# Abstracts of the ICARE 2023 77th SIAARTI National Congress

**DOI:** 10.1186/s44158-023-00111-9

**Published:** 2023-10-25

## Outpatient surgery and Non Operating Room Anesthesia

### **A1 Deep sedation in high-risk patients undergoing Emergency upper GI endoscopy: a retrospective study assessing safety and effectiveness**

#### M. Mariani, A. De Simone, R. Villani

##### Aorn A. Cardarelli, Napoli, Italy

###### **Correspondence:** M. Mariani

*Journal of Anesthesia, Analgesia and Critical Care 2023,* **3(Suppl 1):**A1

Sedation may be defined as a drug-induced depression in the level of consciousness. The purpose of sedation and analgesia is to relieve patient anxiety and discomfort, improve the outcome of the examination, and diminish the patient's memory of the event. Inadequate sedation/analgesia is to be avoided at any cost, given it may result in undue patient discomfort or patient injury because of lack of cooperation. In most cases, it can affect the endoscopist’s ability to successfully complete the procedure.

Overall, there is no consensus on optimal depth of sedation or sedative agents, however, when it comes to routine, low-risk and moderate endoscopic procedures the administration of propofol in unintubated patients, termed “monitored anesthesia care (MAC)”, paired with benzodiazepine and/or opioids, is one of the most common sedation methods for GI procedures in North America and Europe. On the other hand, Emergency upper GI endoscopy is often prolonged and complex, performed during high-risk conditions, like acute upper GI hemorrhage, sub-acute bowel obstruction/ileus, food bolus, achalasia, Morbid obesity and SpO2 < 95% with supplemental oxygen. In these patients, the upper GI endoscopy is intrinsically aggravated by the increasing risk for airway compromise and sedation can, in addition, put patients at risk of entry into general anesthesia, rendering them unconscious and potentially incapable of protecting their airway, patients may require assistance in maintaining a patent airway, and spontaneous ventilation may be inadequate.

Scarce literature can be found providing guidance on the anesthesiologic conduct during upper GI endoscopy in Emergency, and despite the above-mentioned risks, the present retrospective study conducted on 100 patients treated in the Emergency Gastroenterology Unit at AORN Cardarelli aim at demonstrating that deep sedation obtained with the administration of propofol, benzodiazepine and opioids is a safe and viable option when it comes to complex upper GI endoscopy, especially in those patients where the risk-benefit ratio for general anesthesia is not optimal. The present study can address some of the current literature’s shortages on this topic, however further studies are needed to better support these results.

### A2 Oral procedural sedation in an ambulatory service dedicated to the healthcare of people with disabilities

#### E. Valeri ^1^, C. Benassai ^1^, M.E. Berni ^1^, M. Luchini ^1^, D. Coletta ^2^, G. Vannini ^2^, F. Cei ^2^, K. Franchini ^3^, C. Gini ^3^, L. Meini ^3^, V. Barletta ^4^, R. Tarquini ^2^, R. Spina ^1^

##### ^1^ SOC Anestesia e Rinimazione Ospedale S. Giuseppe, Empoli, ITALY, ^2^ SOC Medicina Interna 1 Ospedale S.Giuseppe, Empoli, ITALY, ^3^ Dipartimento infermieristico Ospedale S.Giuseppe, Empoli, Italy, ^4^ DIrezione sanitaria, Empoli, Italy

###### **Correspondence:** E. Valeri

*Journal of Anesthesia, Analgesia and Critical Care 2023,* **3(Suppl 1):**A2

Introduction: Morbidity and mortality of people with disabilities are significantly higher than in the general population, this is due to the difficulty in accessing care. People with disabilities die 20 years earlier than the average of people without disabilities from causes unrelated to the cause of the disability, 30 years earlier in the case of severe intellectual disability.

The difficulty in accessing treatment is often due to the lack of collaboration and low tolerance towards the healthcare environment and for this reason physical restraint has been and is often used to provide services, even non-invasive or minimally invasive. This increases the resistance and fear of patients towards the healthcare environment, with greater difficulties in subsequent accesses.

Since 2000 with the DAMA Project (Disable Advanced Medical Assistance) and since 2017 in Tuscany with the PASS project (Assistance Paths for People with Special Needs) dedicated paths have been created to facilitate access to care for people with disabilities.

Tools: The DAMA-PASS clinic has been active in the ASL Toscana Centro, at the S. Giuseppe hospital in Empoli, managed by a multidisciplinary team made up of anesthesiologists, internists, facilitator nurses, and specialists who are involved case-by-case. The presence of the anesthetist is essential for those patients for whom it is not possible to obtain collaboration in any way except with sedation, absolutely avoiding physical restraint.

With the consent of the legal administrator, non-cooperative patients are subjected to oral sedation in the NORA regimen, this may be necessary for the execution of non-invasive tests or it may be preparatory to obtaining a venous access in order to carry out continuous sedation with intravenous drugs for invasive procedures. Oral sedation is performed with midazolam, mixed with a drink the patient likes, in the range of 0.3 mg/kg to 1.3 mg/kg.

Results: Since 2015 we have taken care of 438 patients (250 males, 188 females). Of these, 164 patients required sedation. Oral sedation involved 64 males (25.5% of the total males) for a total of 103 oral sedations and 21 females (11.2% of the total females) for a total of 28 oral sedations.

The mean midazolam dose used was 0.7 ± 0.2 mg/kg body weight in males, 0.6 ± 0.2 mg/kg body weight in females. We obtained, with an onset of 20-25', a moderate-deep sedation (RASS - 3/ - 5) which allowed the positioning of the venous access and the subsequent procedures without restraint. Complete awakening occurred spontaneously after 60-90' in 50% of the patients, for others flumazenil 0.5-0.8 mg was administered IV.

Side effects are rare: one case of bronchospasm, one case of paradoxical reaction to midazolam and three cases of resistance to midazolam. Only one case required hospitalization for observation.

Conclusions: The DAMAPASS of the San Giuseppe di Empoli hospital has guaranteed the provision of care to patients with specific needs in an appropriate and non-traumatic way, thus restoring the real right to health and dignity.

Oral sedation with midazolam has been shown to be acceptable to the patient, repeatable, predictable, reliable and safe.

### A3 Virtual outpatient sedation and vascular implants in oncological patients

#### P. Cuofano ^1^, A. Mignone ^1^, L. Capozzolo ^1^, V. Capodanno ^1^, L. Druella ^1^, G. Verde ^1^, D. Merlicco ^2^

##### ^1^ Hospice il Giardino dei Girasoli - Medicina del Dolore - Cure Palliative - Centro NAD - DS 64 - Eboli - Asl Salerno - Italy; ^2^ Chirurgia Generale Multidisciplinare del Policlinico Riuniti di Foggia e delle Sale Operatorie, Lucera, ITALY

###### **Correspondence:** A. Mignone

*Journal of Anesthesia, Analgesia and Critical Care 2023,* **3(Suppl 1):**A3

BACKGROUND

Patient pain is usually managed with analgesic therapy. However, recent studies have focused on non pharmacological treatments and, among them, virtual reality hypnosis - VRH - (i.e. digital sedation) is an innovative technique that reduces pain and anxiety in clinical practice. During VRH patients wear virtual lens and headphones through which they are conducted in a virtual reality, an immersive virtual experience that combines a 3D video with music, sounds and/or voice. Immersion session follows standard phases of hypnosis. During the induction phase, subjects are over the surface of the sea. They are invited to induce progressive relaxation in their body. The guidance phase brings the subjects slowly under the water. During the deepening phase, subjects follow a soothing underwater experience. The realtering phase brings the subjects progressively back into reality.

MATHERIALS AND METHODS

Our clinical experience has focused on the positioning of implanted vascular devices (PICC, PICC-PORT, FICC-PORT) in oncological adult patients (30 px control) and the combined use of VRH (30 px study).

We have previously collected informed consent, monitored vital parameters (NIPB, HR), and started VRH in the study group during the positioning of the vascular device.

At the end of the procedure we have collected a satisfactory questionnaire reported to the scale of anxiety (Hamilton Scale Anxiety).

We have also timed the procedure in both groups.

RESULTS

We have noticed that patients in the study group have evaluated virtual reality as a pleasant experience, reducing their feeling of anxiety. Vital parameters presented less variability in study group (HR started from 100 HR average to 65 HR average during the surgical procedure), meanwhile in the control group the average HR was 90 during the procedure.

In the study group the time required to carry out the procedure was shorter than control group (30 minutes in study group vs 45 minutes in control group).

CONCLUSIONS

Virtual Reality Hypnosis can improve performance combined with surgical practice, increasing patients’ compliance and reducing time necessary to carry out the procedures, that can help the cut of healthcare costs.

Authors have no conflict of interest

REFERENCES

- Virtual reality hypnosis in the management of pain: Self- reported and neurophysiological measures in healthy subjects Floriane Rousseaux et al. Eur J Pain. 2022;00:1–15.
- Virtual Reality Distraction durante la chirurgia urologica endoscopica in SpinalAnesthesia: A Randomized Control Trial, Moon J., et al (2018), Journal of Clinical Medicine, 8, 2

### A4 Efficacy of intranasal combination of Dexmedetomidine and Midazolam for procedural sedation in children

#### G. Bortone, C. Ferialdi, M. Cacciapaglia

##### Ospedale SS Annunziata, Taranto, Italy

###### **Correspondence:** G. Bortone

*Journal of Anesthesia, Analgesia and Critical Care 2023,* **3(Suppl 1):**A4

Background

Intranasal application of drugs is widely used in pediatric anesthesia, especially for surgical premedication or diagnostic non-painful procedures [1,2]. Midazolam [3,4] and recently Dexmedetomidine [5-6] are most common drugs used as a single intranasal sedative in pediatric population. This retrospective study seeks to evaluate the efficacy and safety of intranasal dexmedetomidine plus midazolam (IN) as a sedatives for diagnostic Magnetic Resonance Imaging (MRI) examinations in children.

Methods

Children 3 to 10 years of age (Table 1), ASA 1-2, undergoing sedation for brain MRI with contrast were enrolled.

A combination of Dexmedetomidine (4 mcg/kg) plus midalozam (0,2 mg/kg) was administered IN before the procedure. The IN cohort (32 children) was matched and compared to a cohort of subjects (44 children) who underwent sedation with IN dexmedetomidine (4mcg/kg) plus endovenous midazolam (0,05-0,1 mg/kg)(EV). MRI was started when subjects reached a deep sedation, according to University of Michigan Sedation Scale (UMSS). We included only good or excellent image quality, with no or almost absent motion artifacts. We evaluated adverse events, i.e. bradycardia, desaturation, nausea, interventions and procedure times. Children were required to have a UMSS of 0 or 1, an Aldrete score > 9 to discharge.

Results

All 32 children of IN group were successfully sedated; there were no significant differences in the rate of observed adverse events/interventions in comparison to the EV cohort (Table 2).

Furthermore, the IN group had a shorter onset time (Table 3) when compared to the EV cohort (p < 0.003), which had a significant effect on procedure and recovery times (p < 0.001).

Conclusions

Total intranasal sedation may be used for children undergoing MRI scans. The IN combination of Dexmedetomidine and Midazolam is a safe and effective form of sedation in pediatric patients showing faster onset and discharge times.

Written informed consent was obtained from the parents of included children.

References

1. Fantacci C, Fabrizio G C, Ferrara P, Franceschi F, Chiaretti A. Intranasal drug administration for procedural sedation in children admitted to pediatric Emergency Room. European Review for Medical and Pharmacological Sciences. 2018; 22: 217-222
2. Pansini V, Curatola A, Gatto A, Lazzareschi I, Ruggiero A, Chiaretti A. . Intranasal drugs for analgesia and sedation in children admitted to pediatric emergency department: a narrative review . Ann Transl Med 2021;9(2):189 |
3. Chen S, Lang B, Wu L, Zhang W. Efficacy and safety of oral versus intranasal midazolam as premedication in children: a systematic review and meta-analysis Minerva Anestesiologica 2023 Feb 27
4. Gómez-Manzano F J, Laredo-Aguilera J A, Cobo-Cuenca A I, Rabanales-Sotos J , Rodríguez-Cañamero S , Martín-Espinosa N M, Carmona-Torres JM. Evaluation of Intranasal Midazolam for Pediatric Sedation during the Suturing of Traumatic Lacerations: A Systematic Review Children 2022, 9, 644
5. Lee S. Dexmedetomidine: present and future directions Korean J Anesthesiol 2019 August 72(4): 323-330
6. Mondardini M C, Migoni A, Cortellazzi P, Di Palma A, Navarra C, Picardo S G, Puzzutiello R, Rinaldi L, Vitale F , Zito Marinosci G Z, Conti G. intranasal dexmedetomidine in pediatrics: update of current knowledge Minerva anestesiologica 2019 December;85(12):1334-45

**Table 1 (abstract A4).** See text for description

Demographic characteristics	IN	EV	***P***-value
	n = 32	n = 44	
Age (yr) ± SD	5 ± 2.30	4.94 ± 1.41	0.79
Weight ( kg) ± SD	21.4 ± 9.53	21.4 ± 7.22	0.98
Sex M/F (%)	60/40	63/37	0.67

**Table 2 (abstract A4).** See text for description

Observed adverse events	IN	EV	***P***-value
Bradycardia	0	1	0.9
Desaturation	0	0	1
Apnea	0	0	1
Nausea	0	0	1
Vomit	0	0	1
Nasal irritation	0	0	1

**Table 3 (abstract A4).** See text for description

Procedure times	IN	EV	***P***-value
Onset time (min) Median (IQR)	25	33.5	< 0.003
Duration of MRI (min) Median (IQR)	42	45	0.27
Recovery time (min) Median (IQR)	86	100	< 0.001

## Veterinary anesthesia

### **A5 Effect of perfusion index on the ability of oxygen reserve index to estimate arterial partial pressure of oxygen in anaesthetized dogs**

#### F. Zanusso, G.M. De Benedictis, L. Bellini

##### Department of Animal, Production and Health, University of Padova, Legnaro, Italy

###### **Correspondence:** F. Zanusso

*Journal of Anesthesia, Analgesia and Critical Care 2023,* **3(Suppl 1):**A5

Local perfusion on the measurement site affects the accuracy of pulse oximetry to estimate arterial hemoglobin oxygen saturation [1]. Using the ratio of pulsatile to non-pulsatile blood flow detected by the probe, the CO-oximeter measures the Perfusion Index (PI) that quantifies local perfusion [2]. Moreover, multi-wave CO-oximetry can estimate arterial partial pressure of oxygen (PaO2) between 100 to 200 mmHg calculating the Oxygen Reserve index (ORi) based on the simultaneous measurement of arterial and venous blood light absorption using a similar technology of traditional pulse oximetry [3,4]. This prospective observational study investigates if changes in PI affect the ability of ORi to estimate PaO2 in anaesthetized dogs.

Thirty anaesthetized and mechanically ventilated dogs undergoing elective procedures were connected to a multi-wave pulse CO-oximeter by a probe wrapped around the tongue to measure ORi and PI. As the pulse signal was judged stable, an arterial blood sample was analysed to obtain the PaO2. The residuals calculated from the regression model that plotted ORi over PaO2 were analysed with a linear regression to investigate the correlation between the PI measured by the device and the PaO2 measured by the blood gas analyser. To identify a range of PI with the strongest correlation between ORi and PaO2, paired measurements, organized according to ascending values of PI, were subset evenly into 4 groups containing 21 paired values (PI1, PI2, PI3, PI4), and the correlation analysis was recalculated for each.

A total of 84 paired measurements were collected, and PaO2 ranged between 72 and 288 mmHg. The median PI value was 1.41 (0.23-3.40). The correlation between ORi and PaO2 was moderate (r=0.69, p<0.001), and the residuals calculated from the regression line moderately correlated with PI (r=0.47, p<0.001). In groups PI1, PI2, PI3 and PI4, the obtained PI ranges were 0.23-0.80, 0.84-1.40, 1.41-1.90, and 2.00-3.40, respectively. No significant differences were observed in PaO2 values among groups (p=0.836). The strongest correlation between ORi and PaO2 was detected in the PI2 (r=0.89, p<0.001), while in PI4, the correlation was not significant (r=0.42, p=0.060).

In anaesthetized healthy dogs, the ability of ORi to estimate the PaO2 correctly was lower if the CO-oximeter displays a PI >2, likely because changes in the pulsatile and non-pulsatile component of lingual blood flow alter the performances of the probe. The value of PI should be taken into consideration if ORi is used to titrate oxygen therapy.

References

1. Schallom L, Sona C, McSweeney M, Mazuski J Comparison of forehead and digit oximetry in surgical/trauma patients at risk for decreased peripheral perfusion Heart Lung. 2007;36(3):188-94.
2. Gayat E, Aulagnier J, Matthieu E, Boisson M, Fischler M. Noninvasive measurement of hemoglobin: assessment of two different pointofcare technologies. PLoS ONE. 2012;7(1):e30065.
3. Scheeren TWL, Belda FJ, Perel A. The oxygen reserve index (ORI): a new tool to monitor oxygen therapy. J Clin Monit Comput. 2018;32(3):379-389.
4. Bellini L, Dzikitib BT, De Benedictis GM, Sepulveda FRA, Maney JK. Oxygen reserve index as a noninvasive indicator of arterial partial pressure of oxygen in anaesthetized donkeys: a preliminary study. Vet Anesth Analg. 2021;48(3):388-392.

### A6 Preliminary comparison of two common NSAIDs (carprofen and meloxicam) for post-surgical pain management in female rabbits undergoing ovariectomy

#### M. Serpieri ^1^, P. Banchi ^2, 3^, G. Bonaffini ^1^, C. Ottino ^1^, M. Mauthe von Degerfeld ^1^

##### ^1^ Centro Animali Non Convenzionali, Department of Veterinary Sciences, University of Turin, Grugliasco, Italy; ^2^ Department of Internal Medicine, Reproduction and Population Medicine, Faculty of Veterinary Medicine, Ghent University, Merelbeke, Belgium; ^3^ Department of Veterinary Sciences, University of Turin, Grugliasco, Italy

###### **Correspondence:** M. Serpieri

*Journal of Anesthesia, Analgesia and Critical Care 2023,* **3(Suppl 1):**A6

Background

The presence of pain in rabbits leads to increased recovery times, stress, decreased gastrointestinal motility with reduced appetite and fecal output [1]. Composite pain scales, such as CANCRS (Centro Animali Non Convenzionali Rabbit Scale), are useful tools for pain assessment in rabbits [1,2]. NSAIDS such as meloxicam and carprofen have analgesic effects and are commonly used after elective surgical procedures; although meloxicam is a well-tested NSAID in rabbits, carprofen has been evaluated to a lesser extent [3,4]. The aim of this study was the comparison between the post-operative effects of meloxicam and carprofen after ovariectomy in rabbits.

Materials and methods

16 mixed-breed domestic rabbits, undergoing elective ovariectomy, were included in this study and divided in 2 Groups, consisting of 8 subjects each. A signed informed consent was obtained. A mix of ketamine, medetomidine and butorphanol (20, 0.4, and 0.2 mg/kg) was administered IM to obtain anesthesia, and ovariectomy was performed. The NSAIDs were post-operatively administered for three days, SQ SID: Group M, 1 mg/kg meloxicam; Group C, 2 mg/kg carprofen. All rabbits received 5 mg/kg enrofloxacin SQ SID and 1 mg/kg metoclopramide SQ BID for three days.

Rabbits were evaluated using CANCRS at 5 time points (T0: baseline, T1-T4: respectively, 6 h after surgery and at 9 am, 1.30 pm, and 6 pm the day after). Also, time of spontaneous feeding and fecal output after recovery were recorded. Statistical analysis was performed with R (v. 4.3.0), considering a significance level of p<0.05. Anova for repeated measures with Bonferroni post-hoc test was used for the analysis of the CANCRS score over time; Mann-Whitney U test was used for comparisons of CANCRS final scores and for times of spontaneous feeding and fecal output between Groups.

Results

There were no significant differences between Groups in CANCRS final score between groups at any time point (Table 1), and in times of spontaneous feeding and fecal output (both p=0.63) (Table 2).

Conclusions

The absence of differences in final scores between Groups suggests that both NSAIDs had similar effects in alleviating post-operative pain, although maximum time to fecal output was shorter in group C. The greater final score for CANCRS was at T1: however, it was beneath the score indicating pain during rabbit gastrointestinal syndrome. Meloxicam and carprofen are therefore both suitable alternatives for post-surgical pain management in female rabbits undergoing ovariectomy.

References

1. Miller AL, Leach MC. Pain Recognition in Rabbits. Vet Clin North Am Exot Anim Pract, 2023; 26: 187-199.
2. Banchi P, Quaranta G, Ricci A, Mauthe von Degerfeld M. A composite scale to recognize abdominal pain and its variation over time in response to analgesia in rabbits. Vet Anaesth Analg, 2022; 49: 323-328.
3. Benato L, Rooney NJ, Murrell, JC. Pain and analgesia in pet rabbits within the veterinary environment: a review. Vet Anaesth Analg, 2019; 46: 151-162.
4. Leach MC, Allweiler S, Richardson C, Roughan JV, Narbe R, Flecknell PA. Behavioural effects of ovariohysterectomy and oral administration of meloxicam in laboratory housed rabbits. Res Vet Sci, 2009; 87: 336-347.

**Table 1 (abstract A6).** CANCRS final scores (mean ± standard deviation) in Groups

	Group C	Group M
T0	2.4 ± 1.1	2.5 ± 1.3
T1	3.5 ± 1.7	3.0 ± 1.9
T2	2.1 ± 1.0	2.0 ± 1.1
T3	1.8 ± 1.1	1.8 ± 1.0
T4	1.7 ± 1.2	1.4 ± 1.2

**Table 2 (abstract A6).** Times of spontaneous feeding and fecal production [median (range)] in hours after recovery

	Group C	Group M
Spontaneous feeding (h)	5.5 (3 – 12)	6 (3 -24)
Fecal output (h)	7 (4 – 17)	7 (3 -24)

### A7 The effect of methylphenidate on anesthesia recovery in experimental pigs: part 2

#### A. Mirra, C. Spadavecchia, O.L. Levionnois

##### University of Bern, Vetsuisse Faculty, Bern, Switzerland

###### **Correspondence:** A. Mirra

*Journal of Anesthesia, Analgesia and Critical Care 2023,* **3(Suppl 1):**A7

Background

Methylphenidate, an inhibitor of dopamine and norepinephrine reuptake transporters, has been suggested to shorten and improve anesthesia recovery [1-3]. In a previous study, we have demonstrated its lack of effect when administered at the end of anesthesia in experimental pigs. The aim of the present research was to evaluate if its administration at extubation would enhance anesthesia recovery.

Materials and methods

Sixteen healthy pigs, 10.0 ± 0.6-week-old (mean ± standard deviation), weighting 28.9 ± 4.7 kg, and of mixed sex (ten females and six castrated males) were included in this experimental, randomized study. A sole propofol infusion was started at a rate of 10 mg/kg/hour and increased by 10 mg/kg/hour every 15 minutes, until reaching an electroencephalographic suppression ratio (SR; as calculated by the SedLine monitor) >80%. Afterwards, propofol was stopped and the pig let recovery. Immediately after extubation, either saline (group SL) or methylphenidate 20 mg/kg (group MP) was administered IV. The following parameters were assessed at baseline, 5 (TP5), 10 (TP10) and 15 (TP15) minutes after methylphenidate administration: heart rate, respiratory rate, diastolic (DAP), mean (MAP) and systolic (SAP) blood pressure, nociceptive withdrawal reflex thresholds, patient state index (PSI), suppression ratio, spectral edge frequency 95% right (SEF r) and left (SEF l). Moreover, time to return of palpebral reflex and jaw tone, and time to return to a PSI of 50, 60, 70 and 80 was calculated. Comparison among groups was performed using the two-way repeated measure ANOVA followed by the Bonferroni test in case of parametric data and the Kruskal-Wallis test followed by the Dunn's Method in case of non-parametric data.

Results

Significantly higher DAP was found at TP5 in group MP compared to group SL (p=0.046). Significantly higher SEF r values were found at TP5 in group MP compared to group SL (p=0.038). No other significant differences were found.

Conclusions

The present research confirms the results of our previous study. No clinically relevant improvements in anesthesia recovery were achieved when administering methylphenidate at extubation in experimental pigs.

Ethical approval (protocol 32015) was obtained from the committee for animal experiments, canton Bern.

References

1. Chemali JJ, Van Dort CJ, Brown EN, Solt K. Active emergence from propofol general anesthesia is induced by methylphenidate. Anesthesiology. 2012; 116(5): 998–1005.
2. Dodson ME, Fryer JM. Postoperative effects of methylphenidate. Br J Anaesth. 1980; 52(12):1265–70.
3. Macris SG, Kadoglou ON, Cacouri AN, Macris GJ. A clinical comparison of the effectiveness of nikethamide, ethamivan, methylphenidate, and bemegride in postanesthetic arousal. Anesth Analg. 1962; 41:593-8.

### A8 The effect of methylphenidate on anesthesia recovery in experimental pigs: part 1

#### A. Mirra, C. Spadavecchia, O.L. Levionnois

##### University of Bern, Vetsuisse Faculty, Bern, Switzerland

###### **Correspondence:** A. Mirra

*Journal of Anesthesia, Analgesia and Critical Care 2023,* **3(Suppl 1):**A8

Background

Due to the lack of antagonists, recovery from anesthesia relies on drugs clearance. The difficulty of controlling such a delicate phase leads to increased risks for the patient, costs and prolonged recovery. Different than pharmacological antagonization, the inhibitor of dopamine and norepinephrine reuptake transporters methylphenidate has been shown to stimulate arousal pathways [1–3]. The aim of the present study was to evaluate the effect of methylphenidate on anesthesia recovery from propofol in experimental pigs.

Materials and methods

Five healthy pigs, 10.4 ± 0.5-week-old (mean ± standard deviation), weighting 25.8 ± 1.8 kg, and of mixed sex (one female and four castrated males) were included in an experimental, randomized, cross-over study. Each pig was anesthetized three times, ensuring a wash-out period of at least 36 hours. Sole propofol was administered intravenously (IV) to effect until intubation. A continuous IV infusion was simultaneously started at 20 mg/kg/hour and increased every ten minutes by 6 mg/kg/hour, together with an additional IV bolus of 0.5 mg/kg. Once electroencephalographic suppression ratio (as calculated by the SedLine monitor) reached a value between 10% and 30%, propofol was stopped and either saline (group SL), methylphenidate 10 mg/kg (group MP10) or methylphenidate 20 mg/kg (group MP20) was administered IV. The following parameters were assessed at baseline, 5 (TP5), 10 (TP10) and 15 (TP15) minutes after methylphenidate administration: heart rate, respiratory rate, diastolic (DAP), mean (MAP) and systolic (SAP) blood pressure, nociceptive withdrawal reflex thresholds, patient state index (PSI), suppression ratio, and spectral edge frequency 95% right and left. Moreover, time to extubation, time to return of palpebral reflex and jaw tone, and time to return to a PSI of 50, 60, 70 and 80 were calcualted. Comparison among groups was performed using the two-way repeated measure ANOVA followed by the Bonferroni test in case of parametric data and the Kruskal-Wallis test followed by the Dunn's Method in case of non-parametric data.

Results

Significantly higher MAP (p=0.042) was found at TP10, and significantly higher SAP (p=0.049) at TP15 in group MP10 compared to group SL. Significantly longer time was needed in group MP20 compared to group SL (p=0.015) to regain jaw tone, and in group SL compared to group MP10 (p=0.039) to reach a PSI of 80. No other significant differences were found.

Conclusions

Methylphenidate does not shorten or improve anesthesia recovery in experimental pigs. Cardiovascular parameters seem to be only marginally affected.

Ethical approval (protocol 32015) was obtained from the committee for animal experiments, canton Bern.

References

1. Chemali JJ, Van Dort CJ, Brown EN, Solt K. Active emergence from propofol general anesthesia is induced by methylphenidate. Anesthesiology. 2012; 116(5): 998–1005.
2. Dodson ME, Fryer JM. Postoperative effects of methylphenidate. Br J Anaesth. 1980; 52(12):1265–70.
3. Macris SG, Kadoglou ON, Cacouri AN, Macris GJ. A clinical comparison of the effectiveness of nikethamide, ethamivan, methylphenidate, and bemegride in postanesthetic arousal. Anesth Analg. 1962; 41:593-8.

## General anesthesia and Perioperative medicine

### **A9 Non-invasive respiratory support in endoscopic surgery with High Flow Nasal Cannula in patients affected by Legionella Pneumophila: a case report**

#### A. Scalvenzi, M. Del Prete, M.E. Porcelli, F. Coppolino, V. Pota, P. Sansone, M.B. Passavanti, M.C. Pace

##### Università degli Studi della Campania L. Vanvitelli, Napoli, Italy

###### **Correspondence:** A. Scalvenzi

*Journal of Anesthesia, Analgesia and Critical Care 2023,* **3(Suppl 1):**A9

Introduction

Legionella Pneumophila is one of the most important causes of respiratory infections in humans and it is the cause of about 30% of nosocomial infections.

Anastomotic leakage following the intervention of esophageal resection presents one of the surgical complications with the highest morbidity and mortality.

Case reports

For the past year we have faced a case of anastomotic leakage post esophageal resection, treated through the application of an endoprosthesis esophageal in a patient affected by L. Pneumophila.

The main problem for the anesthetist during this procedure is identifying the most inadequate perioperative management.

In this specific case, the patient, affected by L. Pneumophila, presented a PO2/FiO2 ratio between 100mmHg and 200mmHg, giving rise to a moderate ARDS panel according to Berlin Criteria (Image 1).

For this reason, we decided to use a non-invasive periprocedural ventilation mode, specifically the High Flow Nasal Cannula (HFNC), to reduce the risk of postoperative pulmonary complications due to invasive ventilation through endotracheal intubation (ETI).

The parameters set during the entire procedure remained stable: Flow 60 L/min, FiO2 100%, Temperature 37 °C.

The patient, opportunely mixed with the current type of anesthesiological procedure and immediately after signing the Informed Consensus, is premedicated with intravenous Midazolam 0.05mg/Kg, while sedation is effected with an initial bolus of Propofol at 0.5mg/Kg. Successively, sedation is maintained whit Propofol in continuous infusion at 3.5mg/Kg/h.

The approximate duration of the procedure is 25 minutes. The patient's vital parameters are stable for all the duration of the intervention, with a SpO2 > 95%, and a MAP > 70 mmHg.

After the procedure, the patient is returned to the Intensive Care unit where he has completed the course of antibiotic therapy, with a progressive reduction of the need for oxygen therapy.

Conclusion

Airway management in patients with L. pneumophila can be a problem, aggravated by a series of additional risks related to ETI, such as pulmonary damage from mechanical ventilation, contamination of the ventilator, delayed weaning and an increase in hospital stay days. The use of HFNCs in endoscopic procedures allows to avoid these risks, eliminating the operating field conflict with the surgical procedure and guaranteeing a higher oxygen saturation compared to other oxygenation devices.

**Image 1 (abstract A9).**
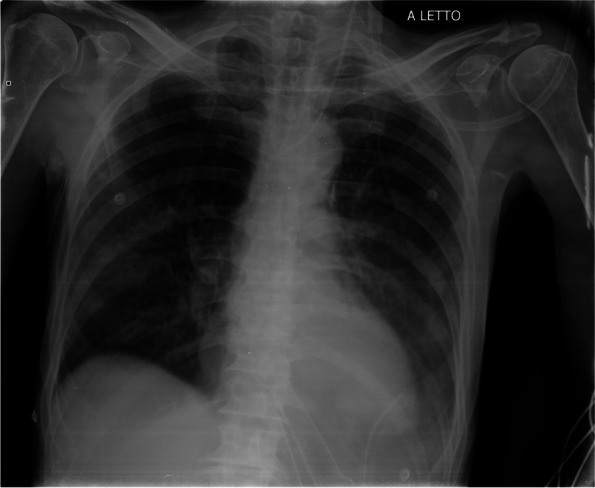
See text for description

### A10 Mortality and thrombotic events in SARS-CoV-2 positive patients after urgent or emergency surgery

#### G. Sudano, M. De Riso, F. Sbaraglia, G. Concina, T.C. Caputo, D.M. Micci, A.M. Scarano, A. Vergari, M. Del Vicario, M. Rossi

##### Fondazione Policlinico Agostino Gemelli IRCCS-Università Cattolica del Sacro Cuore, Roma, Italy

###### **Correspondence:** G. Sudano

*Journal of Anesthesia, Analgesia and Critical Care 2023,* **3(Suppl 1):**A10

Background

The COVID-19 pandemic led to significant changes in the management of surgical patients. Elective surgery underwent a strong reshaping, whereas urgent and emergency procedures continued.

The aim of this retrospective study was to evaluate postoperative mortality rate and thrombotic events in SARS-CoV-2 positive patients undergoing urgent or emergency surgery.

Materials and Methods

After local Ethic Committee’s approval, we reviewed all records of the SARS-CoV-2 positive patients who underwent urgent or emergency surgical procedures between April 2020 and October 2022 in the emergency department of IRCCS Fondazione Policlinico Universitario Agostino Gemelli of Rome.

We included patients who tested positive for both antigenic and molecular SARS-CoV-2 specimens in the time range that goes from 5 days before to 1 day after surgery.

Postoperative mortality rate and the percentage of thrombotic events were evaluated. Mortality rate was specifically assessed on the first postoperative day, between the second and the fifth postoperative day and after 5 postoperative days.

Surgical procedures are summarized in Table 1.

Results

From April 2020 to October 2022, 6387 patients underwent urgent or emergency surgery, 160 of which reported a positive SARS-CoV-2 test. Our patients’ mean (SD) age was 57 (23) years old.

A total of 40 SARS-CoV-2 positive patients died after surgery so the overall mortality rate was 25%: 16 patients died on the first postoperative day (40%), 11 patients died between the second and fifth postoperative day (28%) and 13 patients died after the fifth postoperative day (13%). Dead patients’ mean (SD) age was 73 (15) years old.

Postoperative thrombotic events occurred in 11 patients (7%).

The distribution of fatality and thrombotic events per year are reported in Figure 1 and Figure 2.

Conclusions

Despite the lack of comparison our study provides evidence of what happened during the pandemic period in a high volume COVID-19 hospital.

SARS-CoV-2 positive patients undergoing urgent or emergency surgical procedures during the pandemic had a high risk of mortality and of postoperative thrombotic events.

Even though the highest distribution of SARS-CoV-2 positive patients occurred in 2022, the mortality rate per year resulted higher in 2020, probably due to a lower sensitivity and specificity of the tests used at the beginning of the pandemic and also to the lockdown condition that significantly reduced access to emergency departments.

The results of this study emphasize the importance of carefully evaluating risks and benefits of performing urgent or emergency surgery in SARS-CoV-2 positive patients and the need to implement strict postoperative monitoring to prevent the complications to which these patients are highly susceptible.

**Table 1 (abstract A10).** Demographic and Surgical Characteristics of the Study Population (N=160). Data are presented as mean (SD) and N (%)

Patients characteristics	N=160
Age of SARS-CoV-2 positive patients	57 (23)
Age of dead patients	73 (15)
**Surgical procedures**	
Abdominal-pelvic surgery	67 (42%)
Orthopedic surgery	25 (16%)
Neurosurgery	15 (9%)
Otolaryngology-thoracic surgery	13 (8%)
Endoscopic procedures	13 (8%)
Vascular surgery	9 (6%)
Other procedures	18 (11%)

**Fig. 1 (abstract A10).**
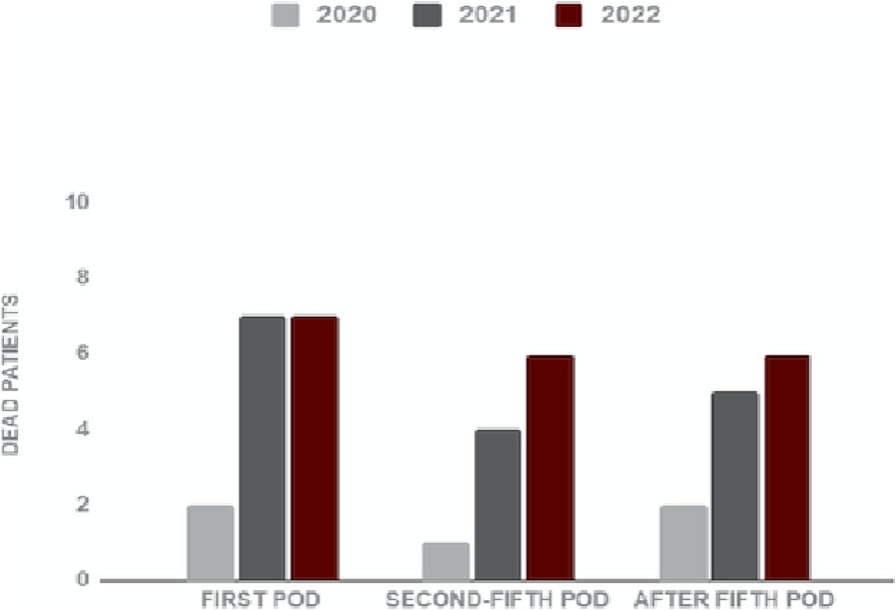
Distribution of mortality per year in SARS-CoV-2 positive patients. PD: postoperative day

**Fig. 2 (abstract A10).**
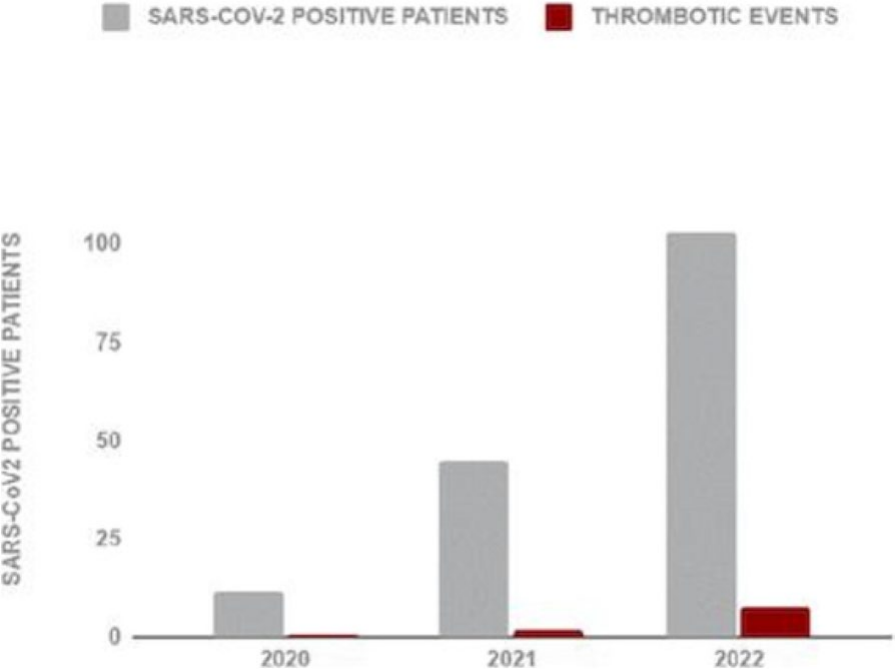
SARS-CoV-2 positive patients and thrombotic events per year

### A11 Preliminary data on the efficacy and safety of intrathecal analgesia within a multimodal analgesia strategy for patients undergoing open liver surgery

#### E. Schirru, L. Fontanarosa, E. Angeli, F. Galasso, V. Brisigotti, A. Cipolletti, S. De Cicco, A. Matarazzo, M. Perfetto, R. Perrucci, C. D'Avino, C. Pacenti, G. Baldini, G. Villa, S. Romagnoli

##### ^1^ Azienda Ospedaliera Universitaria Careggi - Università degli studi di Firenze, Firenze, Italy

###### **Correspondence:** E. Schirru

*Journal of Anesthesia, Analgesia and Critical Care 2023,* **3(Suppl 1):**A11

Background

ERAS guidelines recommend multimodal analgesia strategy for patient undergoing liver surgery and individuate intrathecal opiates as a viable alternative to thoracic epidural analgesia (TEA) with less associated hemodynamic instability [1].

Intrathecal opiates administration reduces the need for systemic opiates that have more undesired side effects, including postoperative nausea and vomiting (PONV), itching and respiratory depression. The use of atropine also seems to contribute to reduction of PONV [2].

Materials and Methods

We conducted an observational retrospective study, including patients over 18 years of age undergoing laparotomic liver surgery. Our objectives were: to compare intrathecal morphine (100-200 mcg) versus TEA and total intravenous analgesia in terms of pain intensity and need for rescue analgesics in the postoperative period; evaluate the incidence of side effects such as PONV, itching and respiratory depression in patients undergoing laparotomic liver surgery after administration of intrathecal morphine.

Data were collected at 0, 3, 6, 12, 24 and 48 hours after surgery. To assess the intensity of pain (static and dynamic) we used the NRS scale, dividing the patients into 4 groups with no (NRS 0), mild (NRS 1-3), moderate (NRS 4-5) and severe pain (NRS>5).

Results

Data from 171 patients were analyzed. Eighty-seven patients received only intravenous analgesia, seventy intrathecal morphine and fourteen TEA.

We found no significant difference in static NRS between the three groups of patients; we recorded significant difference in dynamic NRS between intrathecal opioid group and total intravenous analgesia group, while we didn’t find significant difference in dynamic NRS between intrathecal opioid group and TEA group. There was no significant difference in incidence of side effects (e.g. PONV) between the three groups of patients.

No major complication followed intrathecal morphine administration.

Conclusions

Intrathecal analgesia has shown to be effective in post-operative pain control in the first 24-48 hours after surgery. Dosages of 100-200 mcg of morphine seem to be safe and also it seems that using atropine as adjuvant could be useful to reduce side effects such as PONV.

In conclusion somministration of intrathecal opioid seems to be a valid alternative to TEA and a better analgesia strategy than total intravenous analgesia.

Data were collected from elettronic-database, patients expressed informed consent to data collection in University-hospital

References

1. Joliat et al. Guidelines for Perioperative Care for Liver Surgery: Enhanced Recovery After Surgery (ERAS) Society Recommendations 2022. World J Surg 47, 11–34 (2023).
2. Baciarello et al. Intrathecal atropine to prevent postoperative nausea and vomiting after Cesarean section: a randomized, controlled trial. Minerva Anestesiol. (2011)

### A12 Opioid sparing strategy: intrathecal morphine in major surgery

#### E. Schirru, L. Fontanarosa, E. Angeli, F. Galasso, V. Brisigotti, A. Cipolletti, S. De Cicco, A. Matarazzo, M. Perfetto, R. Perrucci, C. D'Avino, C. Pacenti, G. Baldini, G. Villa, S. Romagnoli

##### Aziende Ospedaliera Universitaria Careggi - Università degli studi di Firenze, Firenze, Italy

###### **Correspondence:** E. Schirru

*Journal of Anesthesia, Analgesia and Critical Care 2023,* **3(Suppl 1):**A12

Background

Opioid administration remains the mainstay of therapy for post-surgical pain; still these drugs have undesired side effects, including postoperative nausea and vomiting (PONV), delayed recovery of bowel function, itching and respiratory depression. Multimodal analgesia is recommended by ERAS guidelines as a strategy to reduce side effects and obtain a better pain control [1]. Spinal analgesia has a high efficacy and relatively low complications [2]. The use of atropine as adjuvant could reduce PONV [3].

Materials and Methods

We conducted an observational retrospective study, including patients over 18 years of age undergoing major abdominal surgery who received intrathecal analgesia with morphine at a dose of 100-200 mcg. Analgesic effects were evaluated by means of the VAS score (NRS scale) for pain intensity and need for rescue analgesics. In addition, the incidence of PONV and respiratory depression were collected.

Data were registered at 0, 3, 6, 12, 24 and 48 hours after surgery. NRS scale was used to divide the patients into 4 groups with no (NRS 0), mild (1-3), moderate (4-5) and severe pain (>5).

Results

Data from 700 patients were analyzed. Graphic 1 and 2 summarize the results on analgesia and Graphic 3 shows the incidence of PONV. No major complications were recorded and 26 patients (3.71%) experienced pruritus.

Conclusions

Intrathecal morphine has shown to be effective in post-operative pain management during the first 48 hours after surgery. Dosages of morphine of 100-200 mcg seem to be safe in terms of respiratory depression.

Data were collected from elettronic-database, patients express informed consent to data collection in University-hospital.

References

1. Tan M, Law LS, Gan TJ. Optimizing pain management to facilitate Enhanced Recovery After Surgery pathways. Can J Anaesth 2015; 62(02):203–218
2. Cook TM, Counsell D, Wildsmith JA et al (2009) Major complications of central neuraxial block: report on the Third National Audit Project of the Royal College of Anaesthetists. Br J Anaesth 102:179–190
3. Baciarello et al. Intrathecal atropine to prevent postoperative nausea and vomiting after Cesarean section: a randomized, controlled trial. Minerva Anestesiol. (2011)

**Graphic 1 (abstract A12).**
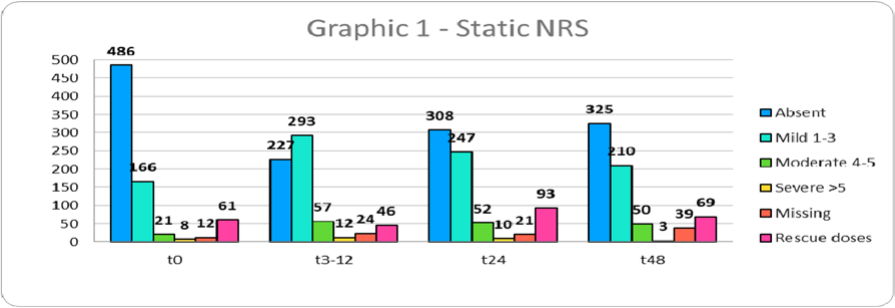
Static NRS

**Graphic 2 (abstract A12).**
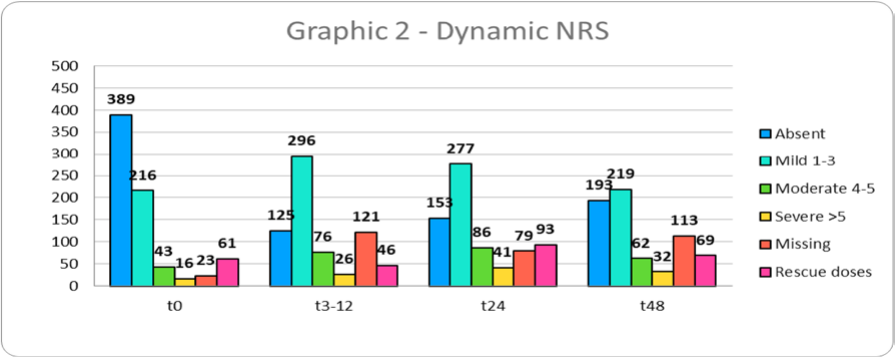
Dynamic NRS

**Graphic 3 (abstract A12).**
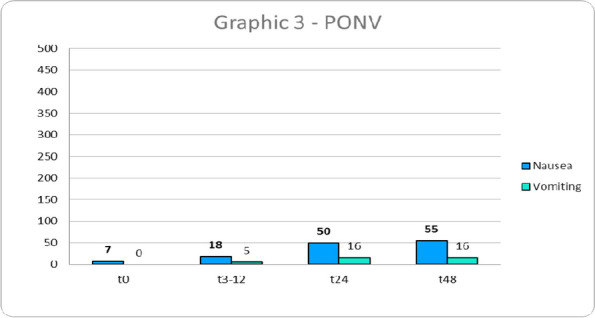
PONV incidence

### A13 Preoperative fasting before pediatric surgery: report on the application of a new protocol

#### M.T. Pizzo ^1^, L. Saccarelli ^2^, E. Schirru ^3^, S. Giacalone ^4^, P. Serio ^4^, D. Colosimo ^4^, Z. Ricci ^3,4^

##### ^1^ Università di Siena, Siena, Italy; ^2^ Università di Perugia, Perugia, Italy; ^3^ Università di Firenze, Firenze, Italy; ^4^ AOU Meyer, Firenze, Italy

###### **Correspondence:** M.T. Pizzo, L. Saccarelli

*Journal of Anesthesia, Analgesia and Critical Care 2023,* **3(Suppl 1):**A13

Current pediatric preoperative fasting guidelines have recommended conservative fasting regimes for many years without significant updates in the last decades. Recent publications have employed more liberal fasting regimes with no evidence of increased aspiration or regurgitation rates. The development of a liberal protocol with a strict application may warrant a shorter period of fasting before pediatric surgery. Essentially, we allowed clear fluid fasting within 1 hour, mother's milk within 3 hours, non-human milk and 'light breakfast' within 4 hours and last meal within 6 hours. The aim of this study was to evaluate the actual times of clear fluid fasting in children undergoing surgery in an institution where fasting was standardized according to the recent European guidelines. A retrospective analysis of fasting times according to local monitoring forms was conducted in a 6 months period. The local ethics committee approved the study. Overall 1000 patients were enrolled in the study. Median (interquartile) age was 7 (3-12) years. All surgeries except cardiac were included. The first 421 children were selected as initial protocol application (group START) in the first 2 months after protocol publication and providers training. START children data were compared to the remaining cases in order to verify if adherence to the protocol tended to improve (group APP). Clear fluid fasting was 180 min (110-342) in START group and 160 (96-320) in group APP (p=0.08) (figure 1).

The improvement was slightly more evident when patients scheduled after the first case (i.e., after 9.30 AM) were analyzed: in this case clear fluid fasting was 207 min (120-345) in START group and 180 (105-306) in group APP (p=0.028). There were 84 adverse events (i.e., hypotension/bradycardia at induction and difficulty to achieve venous access) related to preoperative fasting. At logistic regression these events showed a significant association with duration of clear fluids fasting (OR 0.12 95%CI 0.09-0.17; p=0.02) with a median fasting time in children with presence of adverse events of 377 (210-660) minutes vs 170 (100-351) in children without. Our study showed several important information: 1) in an institution were clear fluids fasting is allowed up to 60 minutes before surgery, median times do not tend to decrease below 2-3 hours. This is essentially due to unwillingness of patients and relatives to provide fluids and to the scarce explanation of the involved healthcare providers to consistently explain this aspect to the patients. 2) Interestingly, some tendency to improvement was observed between START and APP. Then, apparently, fasting times are not influenced by the time of scheduled surgery, even if the APP group seemed to improve with respect to START in the subgroup of 'later' cases. 3) Clear fluid fasting is indeed associated with adverse events related to fasting and this further underlines the importance of respecting protocol times. Further monitoring of fasting times will allow us to appraise if the slight trend to improvement in the APP population will achieve a significant value in the next months and if practices to endorse such improvement will need to be considered.

**Fig. 1 (abstract A13).**
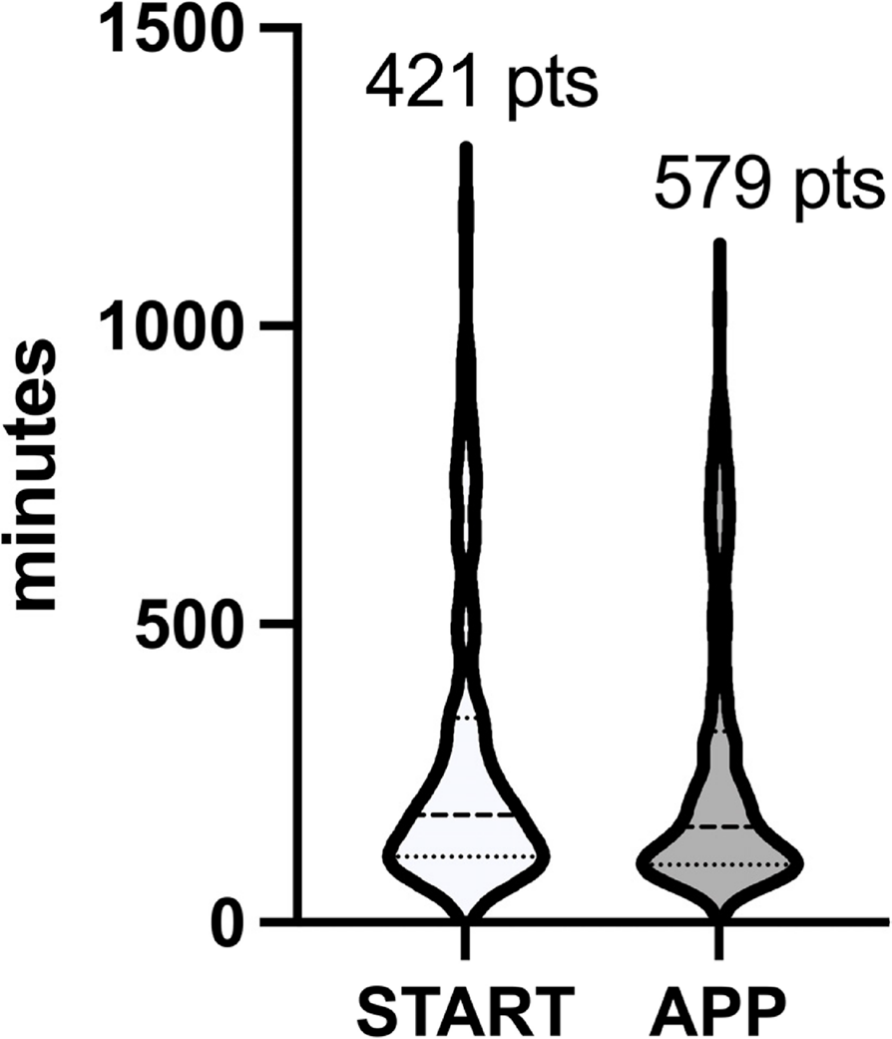
See text for description

### A14 Evaluation of intra-abdominal pressure during postoperative CPAP with helmet

#### P. Priani ^1^, A. Fogagnolo ^2^, M. Verri ^2^, R. Ragazzi ^1,2^, C.A. Volta ^1,2^, S. Spadaro ^1,2^

##### ^1^ Department of Translational Medicine, University of Ferrara, Via Aldo Moro 8, 44121, Ferrara, Italy, Ferrara, Italy; ^2^ Anesthesia and Intensive Care Medicine, Azienda Ospedaliero Universitaria Di Ferrara, Via Aldo Moro 8, 44124, Cona, FE, Ferrara, Italy

###### **Correspondence:** P. Priani (paolo.priani@edu.unife.it)

*Journal of Anesthesia, Analgesia and Critical Care 2023,* **3(Suppl 1):**A14

Background

Postoperative respiratory complications are a major cause of mobidity and mortality during the postoperative period. The PRISM trial investigated the effect of postoperative CPAP via helmet device on PPCs. Although the PRISM trial did not found any statistically significant difference in terms of PPCs [1], CPAP is still widely used in the treatment of respiratory failure during postoperative period. The effect of CPAP via helmet on intra-abdominal pressure still not well investigated.

Materials and Methods

After obtaining informed consent, as a sub-study of the PRISM trial, we enrolled 22 patients that underwent major abdominal surgery and we started CPAP via helmet device according to the PRISM study protocol. We performed arterial BGA and measured intra-bladder pressure through urinary catheter as an indirect measure of intra-abdominal pressure [2]. The measures were obtained before starting CPAP (T0), after 30 minutes (T1) and 4 hours (T2) of CPAP.

Results

All of the patients underwent CPAP via helmet for at least 4 hours. The median level of PEEP administered was 7.5 cmH2O [5-7.625]. At T0, 5 patients (22%) had an IAP equal or grater than 12 cmH2O (cutoff value for defining Intra-abdominal Hypertension [3]). At T1 and T2, 10 (45%) and 12 (55%) patients respectively met the criteria for IAH. 6 patients (27%) with normal IAP values at T0 developed grade 1 or 2 IAH at T1 and/or T2. The mean IAP was 8.57±3.82 at T0, 10.20±4.36 at T1 and 11.16±4.16 at T2. We found a statistically significant increase in the IAP between T0 and T2 (p=0.0012). We found a statistically significant increase in P/F ratio between T1 and T2 (p=0.023) but not between T0 and T1 (p=0.27). No statistically significant variations were found in creatinine levels before and after the CPAP (p=0.48).

Conclusions

IAP was significantly higher after 4 hours of CPAP. In 6 cases, IAP increased from normal values to over 12 cmH2O during CPAP.

References

1. PRISM trial group. Postoperative continuous positive airway pressure to prevent pneumonia, re-intubation, and death after major abdominal surgery (PRISM): a multicentre, open-label, randomised, phase 3 trial. Lancet Respir Med. 2021 Nov;9(11):1221-1230. doi: 10.1016/S2213-2600(21)00089-8. Epub 2021 Jun 18. Erratum in: Lancet Respir Med. 2021 Sep;9(9):e95. PMID: 34153272.
2. Al-Abassi, A.A., Al Saadi, A.S. & Ahmed, F. Is intra-bladder pressure measurement a reliable indicator for raised intra-abdominal pressure? A prospective comparative study. BMC Anesthesiol 18, 69 (2018). https://doi.org/10.1186/s12871-018-0539-z
3. Papavramidis TS, Marinis AD, Pliakos I, Kesisoglou I, Papavramidou N. Abdominal compartment syndrome - Intra-abdominal hypertension: Defining, diagnosing, and managing. J Emerg Trauma Shock. 2011 Apr;4(2):279-91. doi: 10.4103/0974-2700.82224. PMID: 21769216; PMCID: PMC3132369.

### A15 Psychological state assessment in patients undergoing elective abdominal surgery under general anesthesia

#### V. Bellini, S. Celoria, M. Panizzi, M. Badino, T. Domenichetti, E.G. Bignami

##### Anesthesiology, Intensive Care and Pain Medicine Division, Department of Medicine and Surgery, University of Parma, Parma, Italy

###### **Correspondence:** M. Panizzi

*Journal of Anesthesia, Analgesia and Critical Care 2023,* **3(Suppl 1):**A15

Background

Perioperative anxiety is one of the most impacting aspects on the patient's quality of life in the perioperative period. This pilot study, which started on February 7, 2019 and ended on January 29, 2020, has as primary objective to investigate the role of anxiety in the context of general anesthesia during elective abdominal surgery in adult patients, clarifying the correlation between the extent of anxiety state and the level of pain in the postoperative period.

Materials and Methods

This is a prospective, observational, single-centre study. Participants were adults patients undergoing videolaparoscopic cholecystectomy. Informed consent was obtained for each patient. Anxiety before surgery and 1 day postoperatively were measured using the APAIS (Amsterdam Preoperative Anxiety and Information Scale) questionnaire, while pain was investigated by using NRS (Numeric Rating Scale) and VAS (Visual Analogue Scale). Along with these, other clinical variables were recorded (Fig. 1).

Results

31 patients were included in the pilot study, 22 female and 9 male. Mean age was 57 years. An average score of 11.06 on the APAIS scale was observed in the postoperative period versus an average score of 13.38 in the preoperative period, highlighting how patients tended to have less anxiety in the postoperative phase than in the preoperative one. Correlating the APAIS questionnaire score with the postoperative pain by NRS scale and setting a value greater than/equal to 4 as a cut-off, 21 out of 31 patients (67.74%) had an NRS greater than or equal to 4, and of these 15 had an APAIS score greater than or equal to 14 (above the calculated mean); at the opposite, 10 out of 31 patients had an NRS less than or equal to 3, of these 8 had an APAIS score less than or equal to 13 (below the calculated mean).

Conclusions

The results of this study show that highly anxious patients tend to present more pain postoperatively, while moderately anxious patients present less pain postoperatively. It becomes therefore important to create perioperative programs that can not only manage pain in the postoperative phase with the existing pain services but also recognize, investigate, and treat the patient's anxious state preoperatively, in order to create more patient-centered pathways.

In addition, this pilot study may give the basis for further studies to understand the pathophysiological processes that correlate anxiety with pain, such as inflammatory status and/or other perioperative variables.

**Fig. 1 (abstract A15).**
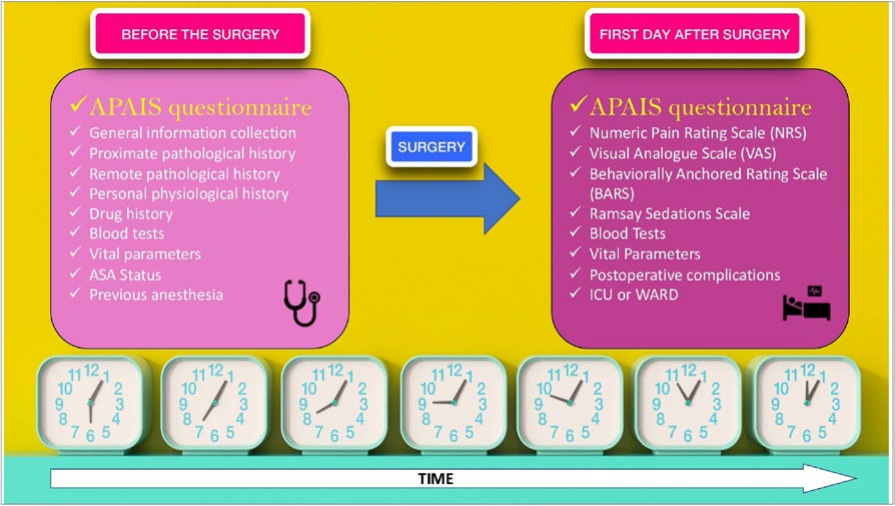
See text for description

### A16 Multimodal prevention strategy for high-risk postoperative nausea and vomiting: a case report

#### V. Nussbaum, F. Sbaraglia, C. Riso, G. Ferrone, A. Catalano, A. Piersanti, D. Del Prete, A. Graziano, D. De Padova, M. Rossi

##### Fondazione Policlinico Agostino Gemelli IRCCS - Università Cattolica del Sacro Cuore, Roma, Italia

###### **Correspondence:** V. Nussbaum

*Journal of Anesthesia, Analgesia and Critical Care 2023,* **3(Suppl 1):**A16

Background

Postoperative nausea and vomiting (PONV) is a condition that can occur in the immediate postoperative period up to 24 hours after surgery. PONV is usually a self-limited adverse event, treated without sequelae, but it could lead even to severe complication [1]. This condition is still underestimated but a comprehensive multimodal approach should be indicated facing high-risk patients.

Case report

A 42-years old woman scheduled for elective bilateral breast implant replacement, ASA score I, BMI 23.03, nonsmoker, reported previous episodes of severe PONV following general and neuraxial anesthesia and motion sickness. She was considered a risk 4 based on simplified Apfel score. She refused a loco regional anesthesia, so a multimodal strategy was proposed to reduce the risk of PONV.

In pre-anesthesia, premedication was given iv with ondansetron 4 mg and dexamethasone 4 mg while magnetic beads were positioned on the surface of both ears’ skin, in antiemetic points (Figure 1).

In the operating room, after multiparametric monitoring, general anesthesia was induced with propofol in Target Controlled Infusion (TCI) 3 mcg/ml and remifentanil TCI 2 ng/ml. Intraoperative maintenance was ensured by a bolus of fentanyl 100 mcg, remifentanil TCI (2-3 ng/ml), ketamine 0.8 mg/kg and midazolam 10 mg both in refracted doses. Towards the end of the surgery, acetaminophene 1g, ketorolac 30 mg, and metoclopramide 10 mg were administered. In the emerging phase, remifentanil was discontinued and propofol was reduced to 1 mcg/ml until the patient resumed spontaneous breathing. The patient was successfully extubated and propofol infusion terminated.

The patient stayed under observation for about 45 minutes in the Recovery Room. She wasn’t agitated and denied PONV.

During the 24 hours after surgery, the patient didn't experienced either nausea or vomiting or pain and was in complete well-being for the discharge.

Conclusion

Preventing PONV in high-risk patients should require a multimodal strategy to reach a safe postoperative recovery. In our case we provide a multimodal strategy based on three cornerstones. Total intravenous anesthesia technique with opioid sparing approach is nowadays considered the gold standard technique [2]. The use of pharmacological prevention and homeostasis maintenance during surgery has been proved as effective in reducing emetic stimulus [3]. We add in this case a non-pharmacological approach using acupuncture technique, useful for PONV managing [4]. In view of this multimodal strategy, we hope that in the future acupuncture approach will be more used as well as the pharmacological approach.

Informed consent to publish had been obtained.

References

1. Sbaraglia F, Saviani M, Timpano JM, Rossi M. Postoperative nausea and vomiting as a cause of tracheal injury: an underestimated life-threatening adverse event? BJA 2019 Sep.
2. Apfel CC, Kranke P, Piper S et al. Nausea and vomiting in the postoperative phase. Expert- and evidence-based recommendations for prophylaxis and therapy. Anaesthesist. 2007 Nov.
3. Haynes GR, Bailey MK. Postoperative nausea and vomiting: review and clinical approaches. South Med J. 1996 Oct.
4. Ongel E, Erdag E, Adiyeke E, et al. Acupressure Versus Ondansetron Usage for Postoperative Nausea and Vomiting After Gynecologic Surgeries. Cureus. March 2023

**Fig. 1 (abstract A16).**
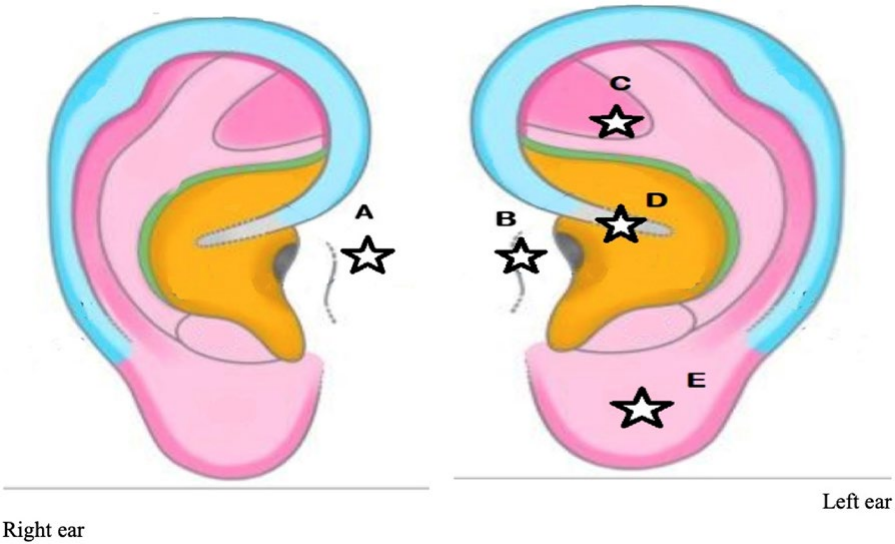
Antiemetic ears points

### A17 Prediction of pain and opiod utilization by nipe in neonates during craniosynthesis: a prospective observational pilot study

#### C. Malatesta, R. Garra, F. Tosi, R. Festa, F. Sbaraglia, A. Pusateri, M. Spanò, F. Antonicelli, M. Rossi

##### Fondazione Policlinico Agostino Gemelli IRCCS - Università Cattolica del Sacro Cuore, Roma, Italy

###### **Correspondence:** C. Malatesta

*Journal of Anesthesia, Analgesia and Critical Care 2023,* **3(Suppl 1):**A17

Background

The Newborn Infant Parasympathetic Evaluation (NIPE) is a Heart rate variability-based technology, developed in 2015, for assessing pain and comfort in neonates under 2-years-old, including premature infants. It is an adaptation of the Analgesia Nociception Index (ANI), developed for monitoring adults and children > 2 years, during surgery under GA. NIPE estimates HR, HF oscillations (> 0.15 Hz), reflecting parasympathetic activity. The monitor displays 2 values, ranging from 0 to 100: the instant-NIPE (NIPEi) and the mean-NIPE (NIPEm). A decrease in NIPE score is interpreted as indicating an increased stress level, whereas an increase in NIPE score indicates improved comfort. According to the manufacturer, NIPE value <50 is indicative of either discomfort, stress, or pain.

Little is known about the performance of the NIPE in anesthetized neonates and infants.

The aim of this study was to investigate the effectiveness of NIPE in predicting painful stimuli, in newborns undergoing GA, and the impact on the need of postoperative analgesia.

Methods

In this single center, prospective, observational pilot study, 28 infants, aged <15 months and weighting <10Kg were recruited for correction of craniosynostosis. Patients were compared in two groups: the NIPE group received Fentanyl (1-2 mcg/Kg) according to the NIPE value (NIPE score< 50); the Control group received Fentanyl (1-2 mcg/Kg) relying on changes in heart rate and blood pressures, together with other clinical signs such as sweating or movements. The primary endpoint was to investigate the ability of the NIPE and heart rate to detect an insufficient antinociception. The secondary endpoint was the correlation between NIPE values and heart rate and the amount of postoperative analgesia.

Results

For statistical analysis Student’s T test was used for unpaired data. A p=0.05was considered statistically significant. A reduction in the use of fentanyl in the NIPE group is evident, with a mean of 12 mcg versus the 22mcg of the control group (Figure 1).

A wide inter-variability was found in the control group. The Pearson’s chi-square test was used for the secondary objectives. NIPE group showed a tendency to match heart rate values and NIPE value reduction at various times of surgery with no significant statistically difference.

Conclusion

This study provisionally suggests the ability of the NIPE to detect insufficient antinociception and is valuable in reducing the amount of opioids really needed by the infant.

References

1. Upton HD, Ludbrook GL. “Intraoperative “Analgesia Nociception Index” guided fentanyl administration during sevoflurane anesthesia in lumbar discectomy and laminectomy: a randomized clinical trial.” Anesth Analg 2017; 125: 81-90.
2. Szental JA, Webb A. “Postoperative pain after laparoscopic cholecystectomy is not reduced by intraoperative analgesia guided by analgesia nociception index (ANI®) monitoring: a randomized clinical trial.” Br J Anaesth 2015; 114: 640-5.
3. Gruenewald M, Herz J. “Measurement of the nociceptive balance by Analgesia Nociception Index and Surgical Pleth Index during sevoflurane-remifentanil anesthesia.” Minerva Anesth 2015; 81: 480-9.

**Fig. 1 (abstract A17).**
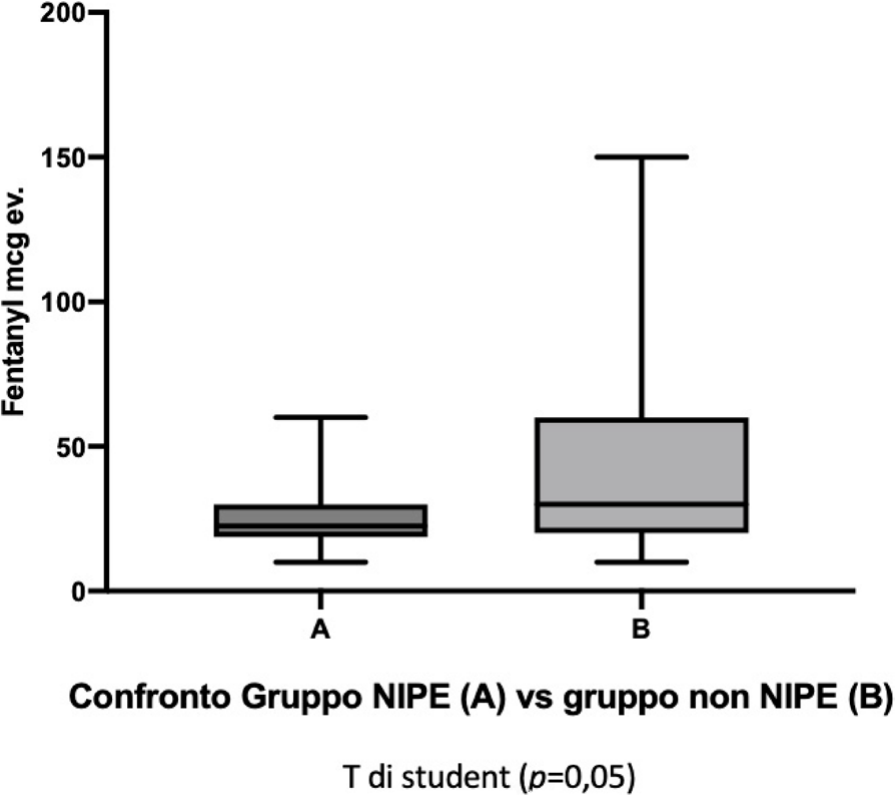
See text for description

### A18 Perioperative cardiocirculatory arrest in a young woman with undiagnosed OSAS: a case report

#### S. Pilloni, A. Busia, E. Lai, M. Muceli, G. Olla, A. Orru', S. Paba, A. Paddeu, M.V. Piroddi, S. Serdino, A. Usai, F.M. Loddo

##### Ospedale nostra signora della mercede - SC Anestesia E Rianimazione - ASL Ogliastra, Lanusei, Italy

###### **Correspondence:** S. Pilloni

*Journal of Anesthesia, Analgesia and Critical Care 2023,* **3(Suppl 1):**A18

Background. Obstructive sleep apnea syndrome (OSAS) is a frequent pathology: it is estimated that 25% of all patients admitted for elective surgery suffer from OSAS and that more than 80% of them are unaware of it. OSAS is known to be responsible of serious postoperative complications, especially respiratory and cardiovascular, including deaths that are often referred generically to cardiac arrest.1 Guidelines propose decision-making flow charts to manage the most critical aspects of the perioperative path, guiding the screening and identification of patients with suspected OSAS and guiding the planning of pre-, intra- and post-operative care levels.2

Case report. A 35-year-old female with grade I obesity (BMI 35.5) and no other comorbidities underwent videolaparoscopic cholecystectomy. The medical history did not report neither snoring nor daytime sleepiness, and the STOP-Bang questionnaire showed a mild risk for OSAS (score 3).

According to the SIAARTI 2019 OSA screening score, she was classified among patients with a mild risk of OSA (score 1), with a BMI > 35 but with gynoid-type obesity, a neck circumference of 37 cm, and a negative SpO2 test (SPO2 >90% in sitting and supine positions). Moreover, the risk associated with the surgical and anesthetic procedures was moderate (interventional procedure under general anesthesia, score 2). Considering these factors, the risk to be attributed to the patient was to be considered not increased (score 3). After the surgical procedure, the patient was discharged to the ward with SpO2 monitoring, and about 10 minutes after discharge she undergoes cardiocirculatory arrest caused by respiratory arrest. ROSC was obtained rapidly and recovery of consciousness as well, without neurological deficits. On the first postoperative day, episodes of arterial desaturation during sleep were observed, and, in the suspicion of OSAS, pneumologist indicated a polysomnography that confirmed a severe OSAS.

Conclusions. STOP-Bang questionnaire is a good screening tool, but it does not identify all undiagnosed cases of OSAS. Therefore, even in patients at not-increased/low risk for OSAS, the measurement of SpO2 is useful during the post-operative time especially if the risk associated with the surgical and anesthetic procedures is moderate/high. In these situations, the increased levels of analgesia/sedation can have a negative impact on predisposed patients.

Informed consent to publish had been obtained.

References

1. Fouladpour N et al. Perioperative complications in OSA patients undergoing surgery: a review of the legal literature. Anesth Analg. 2016; 122(1): 145-51.
2. Corso R et al. Perioperative management of OSA: a systematic review. Minerva Anestesiol.. 2018; 84 (1): 81-93.

### A19 The importance of treatment with perioperative cpap in patients with OSA/OHS candidate for bariatric surgery – a retrospective observational study

#### C. Gobbi ^1^, E. Campane ^1^, L. Botta ^2^, M. Aspesi ^2^, M. Faluomi ^2^, A. Ambrosini ^2^, E.M. Boracchi ^2^, C. Corliano' ^2^, C. Fachinetti ^2^, A. Rizzi ^2^, L. Broggi ^2^, S. Gianazza ^2^, P. Severgnini ^1^

##### ^1^ Scuola di Specializzazione Anestesia, Rianimazione, Terapia Intensiva e del Dolore, Varese, Italy; ^2^ Ospedale L. Galmarini, Tradate, Italy

###### **Correspondence:** C. Gobbi

*Journal of Anesthesia, Analgesia and Critical Care 2023,* **3(Suppl 1):**A19

BACKGROUND

In morbid obesity, central fat accumulation pushes a significant load on the respiratory system, with decreased of lung volumes, lung/chest wall compliance and increased airways resistance.; all contributing to higher breathing work and shorter safe apnea time.

Obstructive Sleep Apnea [1] affects 2/3 of morbidly obese individuals undergoing bariatric surgery. Perioperative continuous positive airway pressure (CPAP) is advised for moderate/severe OSA to avoid respiratory failure.

Obesity Hypoventilation Syndrome [2] is a respiratory consequence of morbid obesity characterized by alveolar hypoventilation during sleep and wakefulness. It involves an interaction between impaired respiratory mechanics, ventilatory drive and sleep-disordered breathing.

AIM

In our Hospital we currently submit the STOP-BANG questionnaire [3] to all candidates for bariatric surgery, to identify moderate/high risk for OSA in order to direct them to specialistic evaluation and start CPAP treatment. Patients with the clinical features of OHS undergo blood gas analysis: if hypoxemia and hypercapnia (>45mmHg) are present, then CPAP treatment should be considered (Figure 1).

METHODS

Retrospective observational study (Jan. 2021 - Dec. 2022). 156 adult patients enrolled (M 33, F 123), undergoing laparoscopic bariatric surgery. Mean BMI for women: 40.96 (min 31,56; max 54,11), for men: 41.86 (min 32,53; max 53,07). Of these subjects, 70 (45%) had or received a diagnosis of OSA/OHS, and perioperatively used CPAP (Table 1).

RESULTS

No patient receiving perioperative CPAP and undergoing LBS (sleeve gastrectomy) had respiratory complications nor showed respiratory issues during the induction of general anesthesia, and none of them was admitted to ICU for postoperative care due to respiratory insufficiency.

CONCLUSIONS

Based on our experience, we can argue that an adequate perioperative ventilation strategy is useful in patients with OHS/OSA candidates for bariatric surgery [4], also for ERAS (Enhanced Recovery After Surgery) protocol applicability [5]. We should preoperatively investigate these pathologies in order to direct patients to the appropriate treatment to minimize the risk of respiratory failure.

Informed consent for the publication of these clinical data has been obtained from the participants.

**Fig. 1 (abstract A19).**
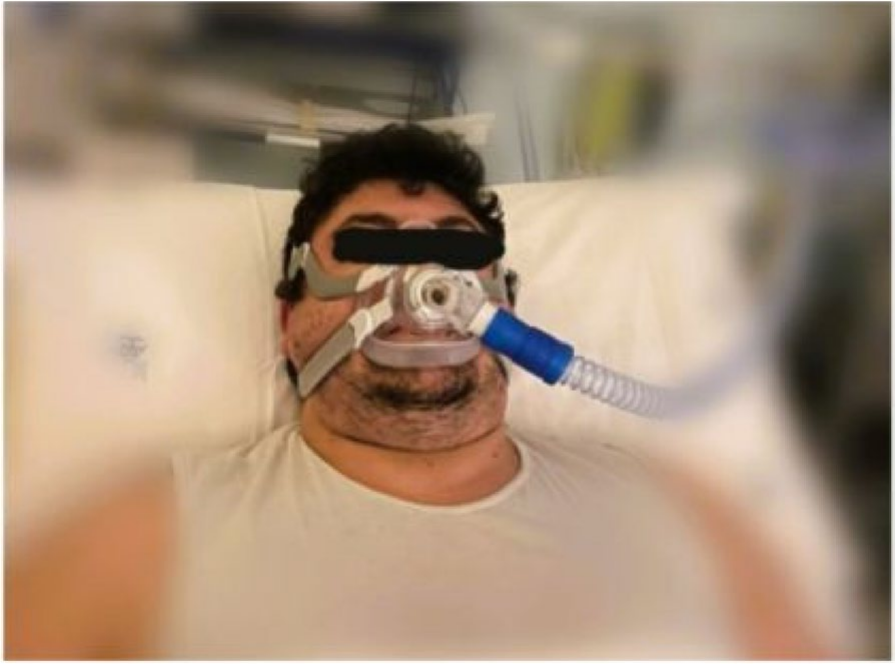
Perioperative CPAP in a patient candidates for bariatric surgery

**Table 1 (abstract A19).**
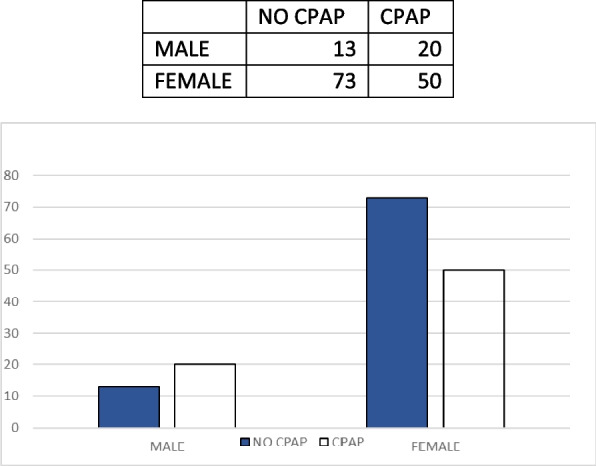
Clinical data

### A20 Perioperative intravenous (I.V.) lidocaine in Laparoscopic Bariatric Surgery (LBS) Improves quality of recovery: an observational retrospective study

#### C. Gobbi ^1^, E. Campane ^1^, L. Botta ^2^, M. Aspesi ^2^, M. Faluomi ^2^, F. Maretti ^2^, S. Passera ^2^, S. Patane' ^2^, E. Serafinelli ^2^, A. Rizzi ^2^, S. Del Ferraro ^2^, F. Lazzarin ^2^, P. Severgnini ^1^

##### ^1^ Scuola di Specializzazione Anestesia, Rianimazione, Terapia Intensiva e del Dolore, Università degli Studi dell'Insubria, Varese, Italy; ^2^ Ospedale L. Galmarini, Tradate, Italy

###### **Correspondence:** C. Gobbi

*Journal of Anesthesia, Analgesia and Critical Care 2023,* **3(Suppl 1):**A20

BACKGROUND

As known in the literature, opioids, due to their breath-depressing effect, can be dangerous for obese patients, who often suffer from disorders such as obstructive sleep apnoea (OSA) and hypoventilation syndrome (OHS) [1,2,3].

At recommended doses [4,5], perioperative i.v. lidocaine is considered safe and effective in ensuring adequate pain control in patients undergoing laparoscopic bariatric surgery (LBS), without the aid of opioids [5,6].

AIM

The present observational retrospective study aims to address the feasibility of opioid free anesthesia OFA with i.v. lidocaine, evaluating its safety and effectiveness in LBS.

METHODS

Clinical data of all patients undergoing LBS at L. Galmarini Hospital (Tradate, Italy) between Jan 2021 and Dec 2022 were retrospectively collected. We entrolled 156 adult patients (M 33, F 123). Mean BMI for women: 40.96 (min 31,56; max 54,11), for men: 41.86 (min 32,53; max 53,07).

We tested a protocol of opioid free anesthesia OFA using i.v. lidocaine (Figure 1).

All patients were evaluated in terms of postoperative pain control according to the Numerical Rating Scale (NRS). Adequate control of postoperative pain was considered reached with NRS lower equal to 4 (24/48 hours).

RESULTS

The protocol used ensured adequate anesthesia and pain control.

Of all patients, 3% did not require post-operative analgesia, 21% required i.v. paracetamol only on the first day at a dose of 1 g every 8 hours, the remaining percentage (76%) required i.v. paracetamol 1g every 8 hours and from 1 to 3 doses of i.v. ketorolac, exclusively during the first day (Graphic 1).

No adverse effects related to i.v. lidocaine were noted during the perioperative time.

CONCLUSIONS

Based on our experience and supported by the literature, we can state that the use of i.v. lidocaine in LBS seems to be safe and effective in reducing consumption of opioids, improving quality of recovery [5].

The results obtained (considering the advantages of an opioid free anesthesia OFA, the easy and safe applicability of the protocol, and the reduced adverse effects associated with the use of i.v. lidocaine) encourage further studies.

The opioid sparing effect of systemic i.v. lidocaine could be a significant criterion to better investigate its efficacy for the applicability of ERAS (Enhanced Recovery After Surgery) protocols [7].

Informed consent for the publication of these clinical data has been obtained from the participants.

**Fig. 1 (abstract A20).**
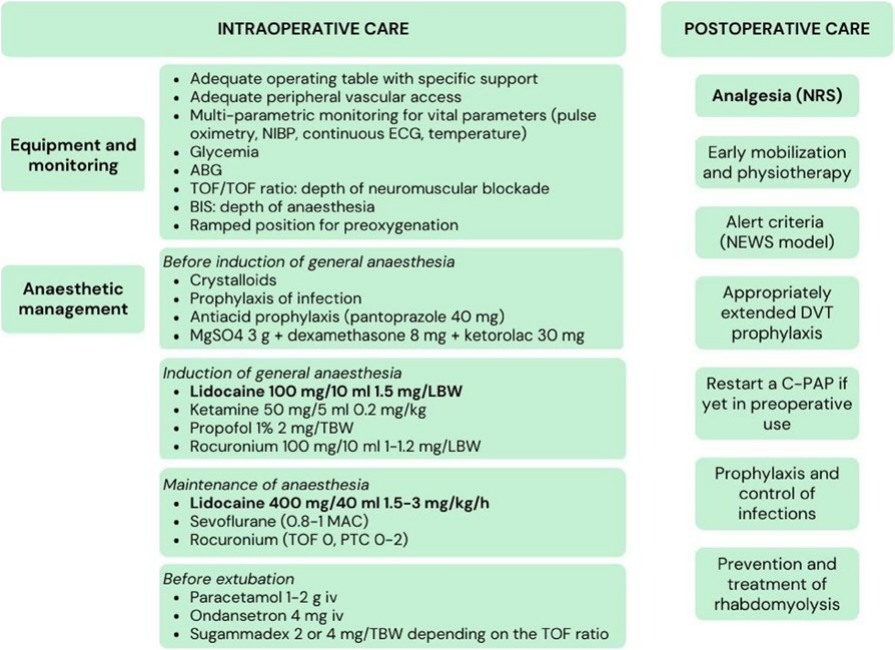
Perioperative management: our protocol of opioid free anesthesia OFA using i.v. lidocaine.

**Graphic 1 (abstract A20).**
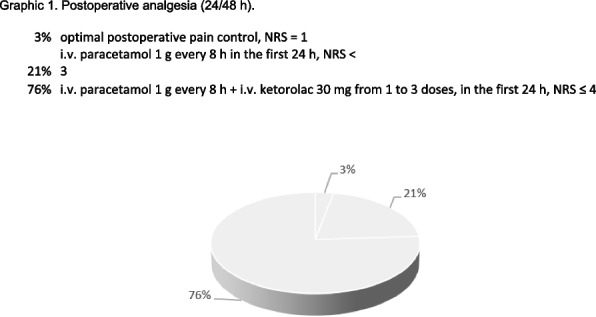
Postoperative analgesia (24/48 h)

### A21 Postoperative severe acute pancreatitis after propofol anesthesia: a case report

#### T.A. Giacon ^1^, F. Carbotti ^1^, N. Asti ^1^, I. Scarpone ^1^, L. Troisi ^1^, S. Congedi ^1^, A. Boscolo ^1^, T. Pettenuzzo ^1^, P. Navalesi ^1^, M. Meggiolaro ^2^

##### ^1^ Institute of Anaesthesia and Intensive Care Unit, Padua University Hospital,, Padova, ITALY; ^2^ Anaesthesia and Intensive Care Unit, Ss. Giovanni e Paolo Hospital, Venezia, Italy

###### **Correspondence:** T.A. Giacon

*Journal of Anesthesia, Analgesia and Critical Care 2023,* **3(Suppl 1):**A21

CASE REPORT

A 75 years old patient whose characteristics are reported in Table 1 was admitted for a programmed laparotomic radical cystectomy due to bladder cancer.

General anesthesia was obtained with target controlled infusion (TCI) of Propofol with Eleved Model [1], TCI of Remifentanil [2], Midazolam, Ketamine and Rocuronium. Other drugs administered are reported in table 1. The patient remained normothermic and hemodynamically stable, even though due to blood loss > 1 l and anemization he was transfused.

At awakening the patient was extubated and transferred to the intensive care unit (ICU) for postoperative monitoring with optimal haemodynamic, neurological and antalgic control.

A few hours after the admission in ICU he reported a blunt abdominal pain, mainly on the right side, nausea and the exams showed increased amylase levels, urologists excluded any surgical involvement of pancreatic tissue and suspected a transient sphincter of Oddi dysfunction [3]. After one night in ICU he was transferred to the urology ward due to his clinical stability, even though Amylase peaked at 2340 U/l. Two days later abdominal pain increased to severe, with characteristics of peritonism and increased inflammatory markers. An abdomen-thorax CT scan revealed acute pancreatitis (AP) (Figure 1) and he was transferred to ICU. He started a standard treatment for acute pancreatitis and antibiotic therapy [4].

On postoperative day nine, due to general deterioration of clinical conditions and further anemization that required blood transfusion, he underwent an emergency laparotomy, in which propofol was not used as an anesthetic drug, that found a completely necrotic pancreas. The following day he faced a multi organ failure (MOF) which rapidly led to death. Consent to publish the case report was accorded by the family.

Acute pancreatitis (AP) Is a complex and severe disease with a high mortality rate [4]. Propofol, is a common anesthetic drug which is widely used in daily practice for sedations and general anesthesia [5], few cases of Propofol Induced AP have been published, in which the diagnosis has been made excluding more common causes [6].

In fact, Propofol is listed as a possible cause of AP, class Ib, based on the classification of Badalov et al. [7]. Following the scheme proposed by the systematic review by Haffar et al for Propofol induced AP [6] we could confirm that our hypothesis is plausible. He satisfied the American College of Gastroenterology criteria for AP [8,9] and it is classified as severe acute pancreatitis according to the Revised Atlanta Classification with peripancreatic necrotic fluid collection [10]. Marshall score [11] after admission was two and Naranjo et al [12] probability scale for drug adverse reaction was three, meaning that the adverse reaction is possible. Latence according to Badalov et al [7] has been short or intermediate. Exclusion of other plausible causes, timing of pancreatitis symptoms and previous cases in literature support our hypothesis of propofol induced pancreatitis.

Informed consent to publish had been obtained.

**Table 1 (abstract A21).** See text for description

Sex	Male
Age (years)	75
Height (cm)	176
Weight (kg)	82
BMI	26.47
Allergies	None
Smoking habit	20 cigarettes/day for 60 years
Alcohol consumption	2 to 3 doses daily, strongly reduced in preoperative period (6 months)
Pathologies	Arterial Hypertension, Dyslipidemia, COPD Gold 1, Low back pain, minimal mitral and tricuspidal rigurgitation, carotid stenosis (50% R, 30% L), mixed anxiety- depressive disorder, irritable bowel syndrome,bladder Cancer stadium T1N0M0 Hg
Previous operations:	Videolaparoscopic Cholecistectomy, appendicectomy, Epigastric Hernia Repair, endovascular prosthesis for subclavian steal syndrome, in situ melanoma exeresis,
Home therapy	Clopidogrel, Perindopril , Indapamide, Symvastatin, venlafaxine and pantoprazol
APACHE II score	10 at first ICU admission
	16 at second ICU admission
	23 after esplorative laparotomy
SAPS II score	22 at first ICU admission
	30 at second ICU admission
	75 after esplorative laparotomy
Charlson Comorbility Index	6
Propofol infusion time (hours)	~ 7
Propofol total infused dose (mg)	~ 3300
Drugs administered intraoperatively	Propofol, Remifentanil, Ketamine, Rocuronium, Midazolam, Cefazolin Dexamethasone, Pantoprazole, Ketorolac, Paracetamol, Sugammadex, MgSO4, Ropivacaine

**Fig. 1 (abstract A21).**
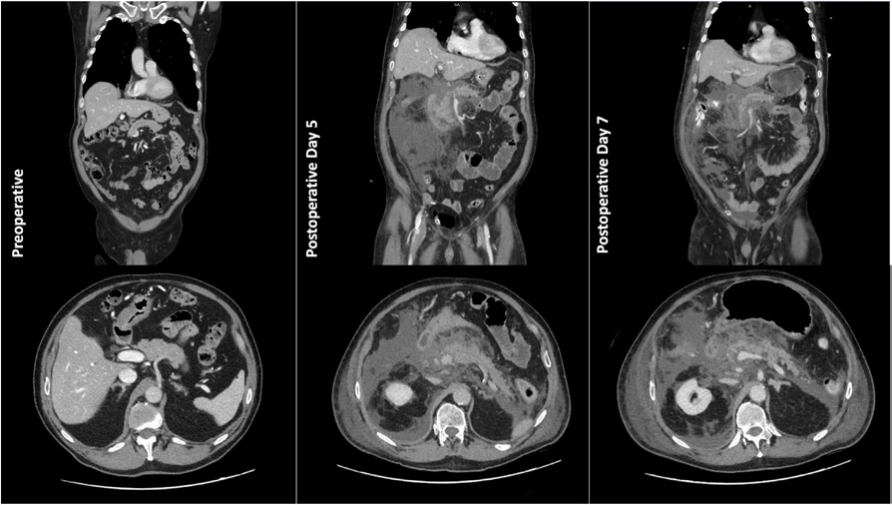
See text for description

### A22 SpO2 reduction, methemoglobinemia and generalized bluish skin discoloration following intradermal administration of patent blue v in breast reconstruction with diep flap: a case report

#### G. Gazzè ^1^, L. Fabbrocile ^2^, C. Coccia ^2^, M. Covotta ^2^, G. Torregiani ^2^, C. Claroni ^2^, E. Forastiere ^2^

##### ^1^ Department of Anesthesia, Intensive Care and Pain Therapy, Policlinico Umberto I, Sapienza University of Rome, Roma, Italy, ^2^ Department of Anesthesia, Intensive Care and Pain Therapy, IRCCS - Regina Elena National Cancer Institute, Rome, Roma, Italy

###### **Correspondence:** G. Gazzè

*Journal of Anesthesia, Analgesia and Critical Care 2023,* **3(Suppl 1):**A22

Background

Patent Blue V (PBV) is used in plastic surgery for sentinel lymph node identification in breast cancers and cutaneous melanomas. Other less allergenic molecules are often preferred over this dye. Currently, propelled by the widespread adoption of lymphatic reconstruction techniques, the use of PBV is strongly re-emerging in clinical practice.

Case report

The patient of our clinical case report is a 60-year-old woman (ASA 2, no history of allergies) admitted to the plastic surgery unit of the IRCCS -Regina Elena National Cancer Institute, Rome, undergoing removal of the left breast prosthesis, debridement of homolateral axillary scar tissue and total breast reconstruction with bipedicled deep inferior epigastric perforator (DIEP) and lymph node flaps. During the procedure, approximately 30 minutes after the intradermal injection of 2 ml of 2.5% PBV sodium salt (50 mg) in the right lower limb, the patient experienced a slight reduction in SpO2 (from 100% to 96%), unresponsive to increased inspiratory fraction of oxygen, and methemoglobinemia was detected in blood gas analysis (maximum value 4.5%), which did not require any treatment. Additionally, a generalized bluish skin discoloration and bluish-greenish urine were observed. No significant hemodynamic, respiratory or hemogasanalytic changes were observed during the procedure; therefore, the decision was made to continue with the surgical intervention. At the end of surgery, the patient was transferred conscious, alert, and cooperative to the Intensive Care Unit (ICU) for postoperative monitoring. The postoperative course in ICU was uneventful and the patient was transferred to the plastic surgery department the following day.

Conclusions

The frequency of allergic reactions to PBV has been estimated to be between 0.2% and 2.7% [1]. Preoperative antiallergic prophylaxis does not prevent anaphylactic reactions but it may reduce their severity.

When administering blue dyes, including PVB, the SpO2 value measured by pulse-oximetry may falsely decrease; therefore, in such cases, serial arterial blood gas analysis is recommended to assess any respiratory exchange abnormalities [2]. Blood gas tests may show falsely elevated methemoglobin values when measured by spectrophotometry [3]. False SpO2 values and methemoglobinemia appear to manifest only when their evaluation is performed in the presence of moderate-to-high blood concentrations of blue dyes using the photometric method. Finally, it is worth noting that in a limited number of cases described in the literature, after the administration of Patent Blue V, the appearance of methaemoglobinemia has occurred, which sometimes has also required treatment.

Informed consent to publish had been obtained.

References

1. Yusim Y, Livingstone D, Sidi A. Blue dyes, blue people: the systemic effects of blue dyes when administered via different routes. J Clin Anesth 2007; 19: 315–321.
2. Pinero A, Illana J, Garcia-Palenciano C et al. Effect on oximetry of dyes used for sentinel lymph node biopsy. Arch Surg 2004; 139: 1204–1207.
3. Howard JD, Moo V, Sivalingam P. Anaphylaxis and other adverse reactions to blue dyes: a case series. Anaesth Intensive Care. 2011;39: 287–292.

### A23 The effect of low-flow anesthesia on emergence agitation in pediatric patients

#### A. Sciusco ^1^, G. Pellico ^1^, C. Belpiede ^1^, M. Ciuffreda ^2^, D. Galante ^1^, C. Piangatelli ^2^, J. Silvestri ^3^

##### ^1^ Department of Anesthesia and Intensive Care, Hospital G. Tatarella, Cerignola (FG), Italy; ^2^ Department of Anesthesia and Intensive Care, ASUR Marche-AV2, Fabriano (AN), Italy; ^3^ Anestesia e Rianimazione Università Politecnica delle Marche, Ancona, Italy

###### **Correspondence:** A. Sciusco

*Journal of Anesthesia, Analgesia and Critical Care 2023,* **3(Suppl 1):**A23

Introduction

We aimed to investigate the positive effects of low-flow sevoflurane anesthesia on the emergence and recovery periods in pediatric patients undergoing adenotonsillectomy.

Material and Methods

The study was performed after obtaining informed consent. Sixty children of ASA I-II aged 2-10 years who were scheduled for adenotonsillectomy were included in the study. Patients were randomly assigned to either low-flow anesthesia or high-flow anesthesia. Heart rate, mean arterial pressure, peripheral oxygen saturation, inspiratory oxygen concentration, end-tidal carbon dioxide, and Bispectral index values of the patients were recorded in the operating room. Spontaneous breathing effort and extubation time were recorded while emergence from anesthesia. Heart rate, mean arterial pressure, peripheral oxygen saturation, pain, and agitation scores were also recorded in the recovery room. The day after surgery, the parents were called to investigate whether the unwanted effects occurred.

Results

Hemodynamic variables were not different between the groups at all times (p> 0.05). There was no difference between the groups in bispectral index values (p> 0.05). Spontaneous respiration time and extubation time were not different between the groups (p> 0.05). Pain and agitation scores were lower in the low flow group (p <0.05). No side effects were observed in any of the groups. Mean arterial pressure and heart rate were similar between groups in our study. However, both of them were decreased during the operation, although they did not drop lower than normal limits. These decreases might have been due to the effect of sevoflurane on cardiorespiratory reflexes, which seemed to be clinically unimportant. Adverse reactions were not detected in any patient in our study indicating that low flow anesthesia does not increase adverse reactions in low flow sevoflurane anesthesia in children.

This study has some limitations. One limitation is the number of participants. Although the numbers are enough to compare groups statistically it would be better if the number of participants had been higher. As we performed routinely in our clinic, we assumed that intravenous paracetamol would be enough for the early postoperative period during which we measured severity of pain which may be another limitation of the study. It could be better if we had planned to give rescue analgesia.

Conclusion

Low flow anesthesia may reduce emergence delirium and postoperative pain even in short-term operations like adenotonsillectomy with no increase in the incidence of adverse events, and with no change in hemodynamic and respiratory parameters in pediatric patients.

### A24 Face mask in the presence of the parents may improve acceptance during inhalation induction induction in pediatric anesthesia

#### C. Belpiede ^1^, G. Pellico ^1^, A. Sciusco ^1^, M. Ciuffreda ^2^, D. Galante ^1^, C. Piangatelli ^2^, J. Silvestri ^3^

##### ^1^ Department of Anesthesia and Intensive Care, Hospital G. Tatarella, Cerignola (FG), Italy; ^2^ Department of Anesthesia and Intensive Care, ASUR Marche-AV2, Fabriano (AN), Italy; ^3^ Anestesia e Rianimazione Università Politecnica delle Marche, Ancona, Italy

###### **Correspondence:** A. Sciusco

*Journal of Anesthesia, Analgesia and Critical Care 2023,* **3(Suppl 1):**A24

Introduction

Face masks are commonly used during the induction phase of pediatric anesthesia. The present study investigated whether the use of a mask in the presence of the parents improved mask acceptance before the slow induction of anesthesia in pediatric patients.

Methods

The study was performed after obtaining informed consent.

This prospective, randomized controlled trial enrolled patients aged 2–10 years who were scheduled to undergo surgery under general anesthesia. Patients were randomly assigned to mask induction with Air/O2/Sevoflurane without the presence of parents (control group) or in the presence of parents (experimental group). The primary outcome was the mask acceptance score, rated on a validated 4-point from 1 point (not afraid; easily accepts the mask) to 4 points (afraid of a mask; crying or struggling). The secondary outcome was heart rate assessed by pulse oximetry in the pediatric ward before transfer to the operating room (OR), at the entrance to the OR, at the patient notification of mask fitting by the anesthesiologist, and after mask fitting.

Results

Seventy-seven patients were accessed for eligibility, with 67 enrolled in the study: 33 in the experimental group and 34 in the control group. Mask acceptance was significantly greater among patients aged 2–3 years in the experimental than in the control group (p < 0.05) (Figure 1).

The mask acceptance score was significantly lower in the experimental than in the control group in patient group 1 (p<0.05)

Conclusions

The use of a mask in the presence of parents can improve acceptance before anesthesia induction with a parental presence in pediatric patients aged 2–3 years.

**Fig. 1 (abstract A24).**
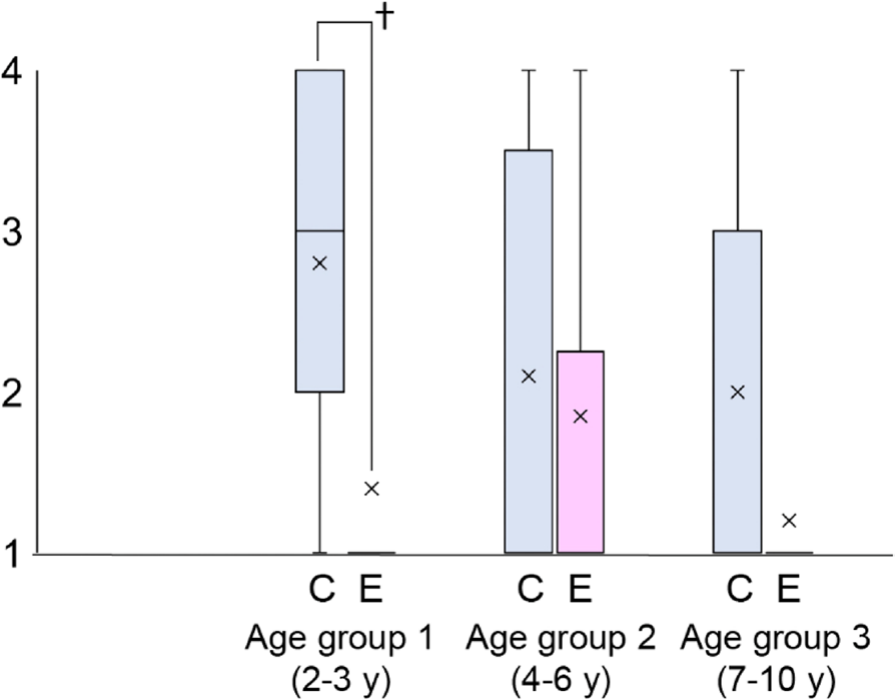
Mask acceptance score

### A25 Implementation of a Multimodal Prehabilitation program for patients undergoing major upper gastrointestinal surgery: the preliminary experience of the University of Florence Prehabilitation Center

#### F. Firenzuoli ^1^, L. Foti ^1^, F. Livi ^1^, F. Barbani ^1^, A.M. Durval ^1^, C. Fiorindi ^2^, C. Tognozzi ^2^, S. Amatucci ^2^, G. Di Testa ^3^, A. Ungar ^3^, F. Staderini ^4^, M. Cricchio ^4^, F. Cianchi ^4^, S. Romagnoli ^1^, G. Baldini ^1^

##### ^1^ Department of Anesthesia and Critical Care, Azienda Ospedaliero-Universitaria Careggi; ^2^ Department of Health Science, University of Florence, Florence, Italy; ^3^ Department of Geriatric and Gerontology, Careggi University Hospital, Florence, Italy; ^4^ Department of Gastroenterological Surgery, Careggi University Hospital, Florence, Italy

###### **Correspondence:** F. Firenzuoli

*Journal of Anesthesia, Analgesia and Critical Care 2023,* **3(Suppl 1):**A25

Background

Multimodal Prehabilitation (MP) before major upper gastrointestinal surgery has shown to improve preoperative functional capacity (FC) and facilitate surgical recovery [1]. Neoadjuvant therapy (NAT), frequently indicated for these patients, is associated with loss of lean body mass (LBM) and deterioration of FC [2]. This cohort study aims at describing the preliminary experience of the University of Florence MP program recently implemented as standard of care for these patients.

Material and Methods

From May 2022 to May 2023, high-risk patients were referred to the University of Florence MPC, in preparation for their surgery. The six-Minute Walking Test, the Fried physical frailty criteria, Patient-Generated Subjective Global Assessment (PG-SGA), bioimpedentiometry, and the Hospital Anxiety and Depression Scale were used to assess physical, nutritional, and psychological status. A personalized MP program was then prescribed for at least 4 weeks, and patients revaluated before and 30 and 90 days after surgery. MP consisted of medical, functional, nutritional, and psychological optimization, including correction of preoperative anemia, optimization of geriatric conditions, and a smoking cessation interventions.

Results

Sixty-two patients were referred to the MPC. Of these, 24 received the intended surgical intervention. Eighteen patients (75%) received NAT (chemotherapy n=14, chemo-radiation n=4) (Table 1).

MP characteristics and interventions are reported in (Table 2).

Overall, there was a clinically meaningful improvement [3] of preoperative FC (six-minutes waking distance, 6MWD), more pronounced in patients who received NAT (Figure 1).

Similarly, preoperative LBM increased; this improvement was mainly driven by an increment of LBM in patients treated with NAT (Figure 1). Compared to baseline, FC decreased after surgery, but return above baseline 90 days after surgery. In contrast, there was a postoperative loss of LBM up to 90 days after surgery. The proportion of sarcopenic, frailty and malnourished patients after MP decreased (baseline vs preoperative: 29% vs 17%, 21% vs 8%, 92% vs 33%, respectively). However, the proportion of anemic patients increased (Figure 2).

Conclusions

MP improved preoperative FC and LBM in oncologic patients undergoing major upper gastrointestinal surgery. FC was maintained throughout the perioperative period, despite the loss of LBM that persisted up to 90 days after surgery. Moreover, in patients treated with NAT, MP prevented the loss of function and LBM commonly observed after NAT. Finally, MP modified several preoperative risk factors associated with postoperative morbidity, mortality and poor functional recovery.

References

1. Minnella EM, Awasthi R, Loiselle SE, Agnihotram RV, Ferri LE, Carli F. Effect of Exercise and Nutrition Prehabilitation on Functional Capacity in Esophagogastric Cancer Surgery: A Randomized Clinical Trial. JAMA Surg. 2018.
2. Jack S, West MA, Raw D, et al. The effect of neoadjuvant chemotherapy on physical fitness and survival in patients undergoing oesophagogastric cancer surgery. Eur J Surg Oncol. 2014;40(10):1313-1320.
3. Antonescu I, Scott S, Tran TT, Mayo NE, Feldman LS. Measuring postoperative recovery: what are clinically meaningful differences? Surgery. 2014;156(2):319-327.

**Table 1 (abstract A25).** Baseline characteristics

	n=24
Age, *yo*	70.96 ±19.98
Gender, *M/F, n*	15/9
BMI	24.95±4.06
6 MWD, *m*	458.41±85.39
6 MWD < 400 *m*	6
PG-SGA A/B/C, *n*	1/16/ 7
Anemia *n (%)*	9 (37.50)
Frailty (Fried phenotype), *n (%)*	5 (20.83)
Type of surgery, *n (%)*	
*Esophagectomy*	11 (45.80)
*Gastrectom*y	13 (54.20)
Neoadjuvant therapy, *n (%)*	18 (75.00)
Active Smokers *n (%)*	4 (16.67)

**Table 2 (abstract A25).** Characteristics of the multimodal prehabilitation program (MP). HIIT=High-Intensity Interval Training; MCT=Moderate Continuous Training; ONS=Oral Nutritional Supplements

	n=24
Duration, days	
Neoadjuvant Therapy	82.29±43.87
No Neoadjuvant Therapy	20.50±47.12
Aerobic exercise, *n (%)*	
HIIT	16 (66.67)
MCT	8 (33.33)
Supervised / Home-based, *n (%)*	18 (75.00) /6 (25.00)
Compliance to aerobic exercise	
Intensity reached/intensity prescribed (%)	89.27
Sessions attended/sessions prescribed (%)	67.00
Whey proteins, *n (%)*	24 (100)
ONS, *n (%)*	17 (70.83)
Immunonutrition, *n (%)*	5 (20.83)

**Fig. 1 (abstract A25).**
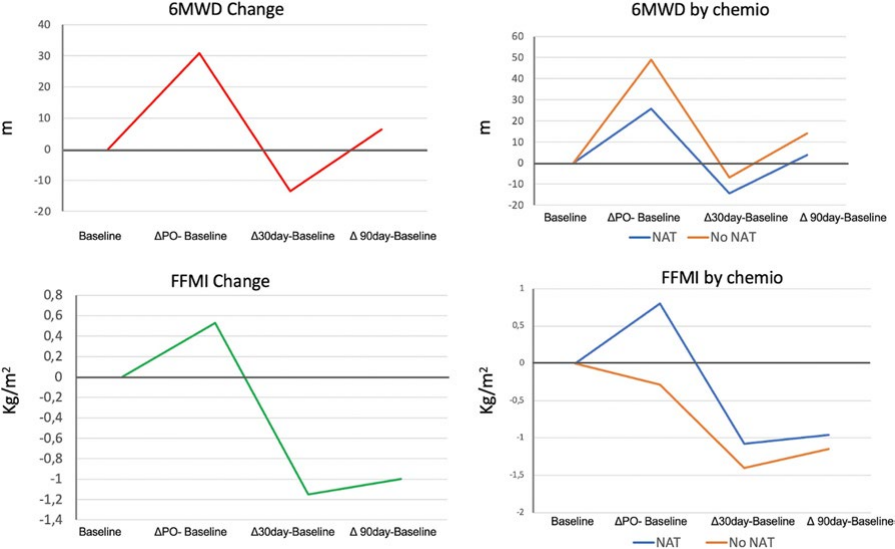
Overall perioperative change of FC and LBM (Fat Free Mass Index, FFMI), in patients treated and not treated with NAT

**Fig. 2 (abstract A25).**
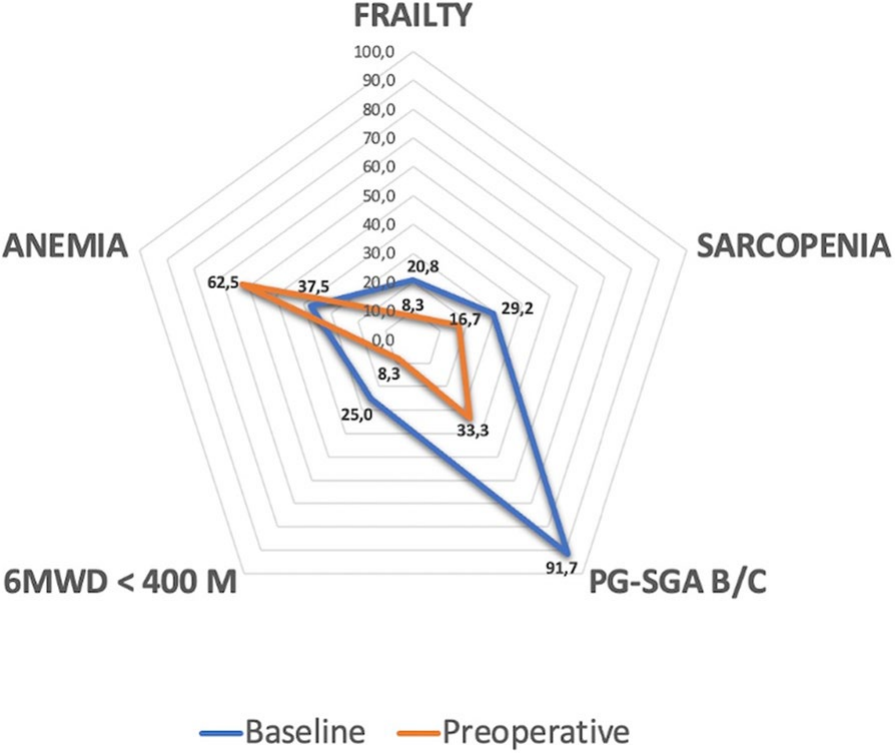
Preoperative change of surgical risk factors

### A26 Rapid sequence induction of anaesthesia: standard procedure vs. high flow nasal oxygen (HFNO)

#### T. Del Santo ^1^, C. Ghelardini ^2^, G. Paladini ^2^, A. Di Filippo ^1^, G. Villa ^1^, G. Baldini ^1^, S. Romagnoli ^1^

##### ^1^ School of Anaesthesia and Critical Care; University of Florence, Azienda Ospedaliero-Universitaria Careggi, Florence, Italy, ^2^ University of Florence, Florence, Italy

###### **Correspondence:** T. Del Santo

*Journal of Anesthesia, Analgesia and Critical Care 2023,* **3(Suppl 1):**A26

Background

Rapid sequence induction (RSI) is an anesthesia technique for tracheal intubation in patients at high risk of aspiration of gastric content. As manual ventilation is not recommended in RSI, pre-oxygenation is a fundamental strategy to ensure an adequate period of safe apnea [1]. The current standard for pre-oxygenation entails spontaneous facemask ventilation (FM) delivering a fraction of inspired oxygen (FiO2) of 100% at 15 L/min flow such as to reach an end tidal O2 (EtO2) of at least 90% prior to the administration of induction medications and myorelaxants. The use of High Flow Nasal Oxygen (HFNO) may offer some advantages over the standard technique [2].

Patients and methods

After obtaining informed consent, patients undergoing anesthesia with indication for RSI were randomized for FM or HFNO. Patients belonging to the FM-group were pre-oxygenated with an anesthesia mask for 5 minutes (15 L/min, FiO2 of 1). After the anesthesia induction, the mask was left in place during the apnea time and removed during the intubation. Patients of the HFNO-group were pre-oxygenated for 5 minutes with HFNO at 40 L/min and FiO2 1. Oxygen flow was increased to 70 l/min after the anesthesia induction and the cannula was left in place during the apnea time and the intubation (figure 1).

PaO2, PaCO2, SpO2 and oxygen reserve index (ORi) were measured before, at 5 minutes of pre-oxygenation and immediately after intubation. Apnea time was measured in each patient. The variables considered were compared. For each patient, the variation of PaO2 and PaCO2 during apnea was related to the apnea time to obtain and compare the mean variation per second during apnea of these variables.

Results

We enrolled 50 patients and randomized 25 for each group. There was no difference in the baseline patient characteristics and basal SpO2, PaO2 and PaCO2. Patients in the HFNO group showed a lower decrease in PaO2 during the apnea time (p=0.0009). In a sub-population with apnea time greater than 300 seconds, PaO2 at intubation was significantly higher in the HFNO group (p=0.046) than in FM-group. Differently, there were no differences between the two groups in PaCO2 (table 1-2).

Conclusions

HFNO might increase the safe apnea compared to the standard method for the pre-oxygenation of patients undergoing RSI offering additional safety especially when the intubation procedure needs more time. In addition, ORi can be a useful non-invasive monitoring of the patient oxygen status during RSI. There was no evidence in CO2 clearance under HFNO.

References

1. El-Orbany M, Connolly LA. Rapid sequence induction and intubation: current controversy. Anesth Analg. 2010; 110:1318–25.
2. Patel A, Nouraei SAR. Transnasal Humidified Rapid-Insufflation Ventilatory Exchange (THRIVE): a physiological method of increasing apnoea time in patients with difficult airways. Anaesthesia. 2015; 70:323–329.

**Fig. 1 (abstract A26).**
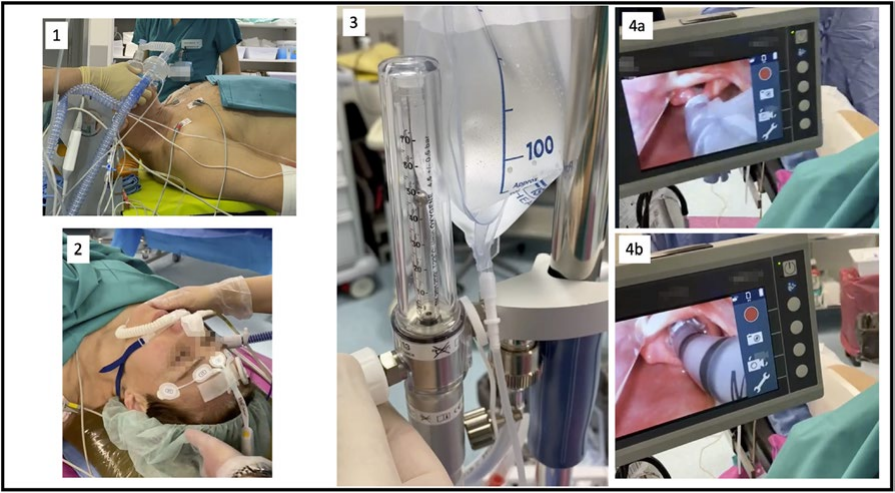
Oxygenation with facemask (1) and with High Flow Nasal Cannula (2-3); during intubation attempts the HFNC is left in place, and airflow continues (4a-4b)

**Table 1 (abstract A26).** Collected data in all the enrolled patients (n=50)

Parameters	FM group	HFNO group	p
PaO_2_ 5’ (mmHg)	414.4 ± 86.3	391.9 ± 98.7	0.430
PaO_2_ ETI (mmHg)	244.0 ± 141.2	312.8 ± 134.5	0.087
PaCO_2_ 5’ (mmHg)	35.3 ± 7.7	34.2 ± 7.2	0.611
PaCO_2_ ETI (mmHg)	48.0 ± 10.4	49.6 ± 6.6	0.512
ORi 5’	0.773 ± 0.198	0.788 ± 0.226	0.825
ORi ETI	0.468 ± 0.324	0.544 ± 0.247	0.356
ΔPaO_2_/t (mmHg/s)	-0.832 ± 0.703	-0.241 ± 0.451	**0.0009**
ΔCO_2_/t (mmHg/s)	0.055 ± 0.028	0.052 ± 0.028	0.713
ΔORi/t (1/s)	-0.001 ± 0.001	0.0007 ± 0.0006	**0.017**

**Table 2 (abstract A26).** Collected data in patients with apnea time > 300 s (n=21)

Parameters	FM group	HFNO group	p
PaO_2_ 5’ (mmHg)	442.2 ± 83.4	398.0 ± 84.5	0.269
PaO_2_ ETI (mmHg)	155.2 ± 82.7	274.2 ± 134.7	**0.046**
PaCO_2_ 5’ (mmHg)	36.6 ± 8.7	32.2 ± 8.0	0.270
PaCO_2_ ETI (mmHg)	55.6 ± 11.6	50.2 ± 8.2	0.237
ORi 5’	0.704 ± 0.226	0.858 ± 0.167	0.092
ORi ETI	0.461 ± 0.413	0.523 ± 0.269	0.681
ΔPaO_2_/t (mmHg/s)	-0.773 ± 0.248	-0.348 ± 0.441	**0.030**
ΔCO_2_/t (mmHg/s)	0.049 ± 0.021	0.050 ± 0.026	0.933
ΔORi/t (1/s)	-0.0006 ± 0.0008	-0.0009 ± 0.0006	0.385

### A27 The NIRS as tissue oxygenation monitoring to optimize blood transfusion in neonates: a prospective observational pilot study

#### C. Cuomo, F. Tosi, R. Garra, R. Festa, F. Sbaraglia, A. Pusateri, M.M. Spanò, C. Malatesta, R. La Macchia, M. Rossi

##### Fondazione Policlinico Agostino Gemelli- Università Cattolica del sacro cuore, Roma, Italy

###### **Correspondence:** C. cuomo

*Journal of Anesthesia, Analgesia and Critical Care 2023,* **3(Suppl 1):**A27

BACKGROUND

Near-infrared spectroscopy (NIRS) is a tool for noninvasive monitoring of regional tissue oxygenation, including splanchnic and cerebral circulation. ^1^

This pilot study aims to analyze the effects of surgical hemorrhages and transfusions on regional oxygen saturation (sSO2). This is detected at the splanchnic level in children undergoing corrective surgery for craniostenosis.

The main purpose of this study is to identify timely transfusion indications. 2

The study also aims to evaluate any correlations between sSO2 and metabolic acidosis markers such as lactate and base excess.

MATERIALS AND METHODS

This no profit observational study involves the Catholic University of the Sacred Heart as the sole participating center.

An INVOS oximeter ( Somanetics, Troy, USA) was used for the study.

This instrument emits light at two wavelengths (730 and 810 nm) in order to measure the concentration of oxygenated and deoxygenated hemoglobin in the microcirculation.

The study examines nineteen patients ages less than 15 months, weighing less than 10 kg, undergoing cranioplasty procedures for the correction of primary craniostenosis. All these patients had a postoperative course in the Pediatric Intensive Care Unit (PICU). Patients with known comorbidities and/or suffering from syndromic craniosynostosis (Crouzon syndrome, Pfeiffer syndrome or Apert syndrome) were excluded from the study.

All patients included in the study underwent the same anesthetic protocol. Plasma expanders were used to replace the lost blood volume. Albumin 5 % was administered in boluses at the initial dose of 20 ml/kg, which could be adjusted according to blood loss.

Conversely, concentrated red blood cells were administered when signs and symptoms of hypoperfusion were observed, considering a hemoglobin cutoff value of 7 g/dL.

At the end of the surgery, the patients were transferred to the Pediatric Intensive Care Unit (PICU), for postoperative monitoring and management of the surgical case.

RESULTS

Data were analyzed using the median and quartiles as the distribution was non-normal (non-Gaussian). From the data analysis, a linear regression was performed between the absolute value (a.v.) values of regional oxygen saturation (rSO2) and hemoglobin (Hb), revealing a significant but weak linear relationship (p=0.001, r2=0.15). On the other hand, the correlation between rSO2 and lactate levels and base excess (BE) values was not statistically significant (p value = 0.97).

CONCLUSIONS

NIRS has proven to be a non-invasive method that could provide valid data for the correct management of perioperative anesthesia. In the pediatric field we could have great benefits in terms of blood saving strategies.

REFERENCES

1. Jobsis FF. Non-invasive, infra-red monitoring of cerebral O2 sufficiency, bloodvolume, HbO2-Hb shifts and blood flow. Acta neurologici Scandinavica Supplementum 1977;64:452-3.
2. Torella F, Haynes SL, McCollum CN. Cerebral and peripheral near-infrared spectroscopy: an alternative transfusion trigger? Vox sanguinis 2002;83:254-7.

### A28 Adherence to Prehabilitation: how is reported and defined? A systematic review of the literature

#### G. Cocci ^1^, C. Donadel ^2^, R. d'Errico ^1^, L. Foti ^1^, F. Barbani ^1^, F. Livi ^1^, T. Piazzini ^3^, S. Romagnoli ^1^, G. Baldini ^1^

##### ^1^ School of Anaesthesia and Critical Care, University of Florence, Azienda Ospedaliero-Universitaria Careggi, Florence, Italy; ^2^ University of Florence, Florence, Italy; ^3^ Biomedical Library, University of Florence, Florence, Italy

###### **Correspondence:** G. Cocci

*Journal of Anesthesia, Analgesia and Critical Care 2023,* **3(Suppl 1):**A28

Background: Prehabilitation (PreHab) refers to individualized preoperative interventions aiming at improving preoperative functional capacity and optimize modifiable risk factors to better overcome surgical stress and better recover to daily activities1. Measuring adherence to a medical intervention is essential for accurately determining its efficacy. It is unclear how adherence to PreHab is currently measured and reported. The aim of this systematic review is to determine how PreHab adherence is measured and reported, and if possible, determine whether high adherence rates are associated with better outcomes.

Materials and methods: PRISMA guidelines for systematic reviews were followed. Medline, Embase, Cochrane Library databases were searched for Randomized Controlled Trial, Clinical Trial, and retrospective studies, from January 1999 to March 2023, and that evaluated PreHab interventions in adult patients awaiting for major or moderate elective surgery. As there is no standardized and unambiguous definition of PreHab, it was decided to search and include trials evaluating any unimodal preoperative intervention including physical exercise, nutritional, and cognitive/psychological support, or multimodal interventions combining at least 2 of the forementioned treatments. Only preoperative treatments that started at least 7 days before surgery were included. Studies aiming at optimizing individual surgical risk factors, such as smoking cessation or anemia were excluded. Included studies were analyzed to investigate 1) how adherence to PreHab interventions was reported and defined 2) the association between adherence to PreHab and preoperative and postoperative outcomes.

Results: Of the 21.313 studies identified from Medline, 246 studies were included for the analysis. PreHab was unimodal in 210 studies (exercise-based PreHab n=127, nutrition-based PreHab n=47, and psychological PreHab n= 36) and multimodal in 36 studies (Table 1).

Different type of prehabilitation interventions were identified (Figure 1).

Overall, adherence to PreHab was reported in 125 of the studies included (50.8%). Of these, 103 (82.4%) defined it. Multimodal PreHab studies that reported adherence to each component of the multimodal intervention prescribed were only 8 (40%); when not entirely reported, adherence to the exercise intervention was the most reported. Parameters used to define adherence were frequency (n=92), intensity/dose (n=1) or duration (n=2) relatively to the PreHab prescription. Sixteen trials used more than 1 of these parameters to define adherence to PreHab (Figure 2).

Due to the high degree of heterogeneity in defining and reporting PreHab adherence it was not possible to assess and establish whether high rates of Prehab adherence are associated with better outcomes.

Conclusions: these preliminary results show that PreHab adherence was not reported in half of the study identified. It was mainly reported describing the frequency of the intervention, relatively to PreHab parameters prescribed. Due to a high degree of heterogeneity defining and reporting PreHab adherence, it was not possible to establish its association with postoperative outcomes. Future studies should better define and report adherence to PreHab, not only describing how frequent the intervention occurred, but also assessing if the intensity/dose and the duration of the intervention are achieved. This will help to better understand the efficacy of PreHab.

References

1. Carli F. Prehabilitation for the Anesthesiologist. Anesthesiology. 2020;133(3):645-652.

**Table 1 (abstract A28).** Studies reporting and defining adherence to prehabilitation

	Adherence to PreHab		
	Reported		Not Reported
	(n=125)		(n=121)
	Defined	Undefined	
	(n=103)	(n=22)	
**Multimodal Prehabilitation,** ***n***	4	16	16
Exercise + psychological intervention, *n*	0	2	2
Exercise + nutritional intervention, *n*	1	2	7
Exercise + nutritional + psychological intervention, *n*	3	12	7
**Unimodal Prehabilitation,** ***n***	18	87	105
Exercise, *n*	14	61	52
Nutrition, *n*	4	15	28
Psychological intervention, *n*	0	11	25

**Fig. 1 (abstract A28).**
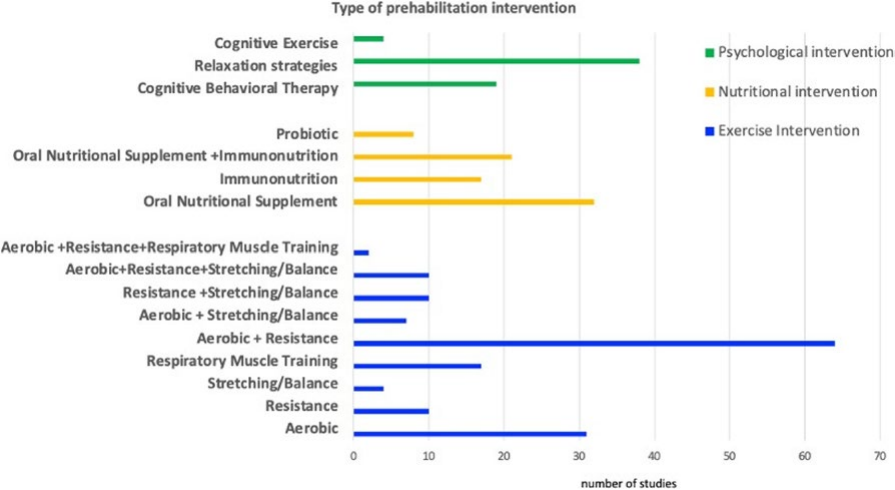
Type of prehabilitation

**Fig. 2 (abstract A28).**
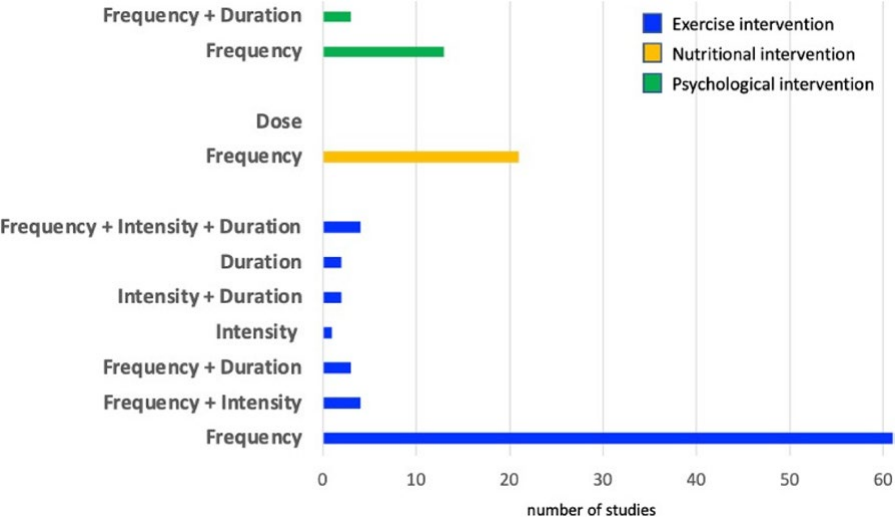
Parameters used to define adherence to prehabilitation

### A29 A randomised controlled trial to assess the efficacy of a dynamic elastance protcol in patientes undergoing major abdominal surgery

#### A. Russo ^1^, P. Aceto ^1^, A.M. Dell'Anna ^1^, L. Cascarano ^1^, L.S. Menga ^1^, B. Romanò ^1^, E. Console ^1^, F. Pugliese ^1^, C. Cambise ^1^, C. Fiorillo ^2^, S. Alfieri ^2^, L. Sollazzi ^1^, M. Antonelli ^1^

##### ^1^ Department of Anesthesiology and Intensive Care Medicine Fondazione Policlinico Universitario A. Gemelli IRCSS, Roma, Italy; ^2^ Department of Digestive Surgery Fondazione Policlinico Universitario A. Gemelli IRCSS, Roma, Italy

###### **Correspondence:** L. Cascarano

*Journal of Anesthesia, Analgesia and Critical Care 2023,* **3(Suppl 1):**A29

Background: Whether a hemodynamic optimization protocol based on dynamic arterial elastance (Eadyn) improves clinical outcomes in patients undergoing major abdominal surgery remains unclear.

Methods: We randomly assigned patients scheduled for major abdominal surgery to Control group (n=23) or Intervention group (n=23) after obtaining written informed consent. We used Hemosphere platform (Edwards Lifesciences, Irvine-California, USA, AcumenIQ sensor) as hemodynamic monitor in both groups. In the Control group, we administered fluids and vasoactive drugs based on Stroke Volume Variation (SVV) and Mean Arterial Pressure (MAP) changes. For the Intervention group, in fluid responders patients, we gave fluid or vasoactive drugs administration according to arterial dynamic Elastance (Eadyn), added to the treatment algorithm. The primary endpoint was the lactate levels at the end of surgery. Secondary endpoints were fluid amount, vasoactive drugs usage and clinical and surgical postoperative complications.

Results: Fourty-six patients were analyzed. No differences beetween the two groups have been noticed in lactate levels at the end of surgery (control group Lac 2.5 mmol/L vs intervention group 2.6 mmol/L p=0.63). The preoperative and intraoperative variables significantly associated with anastomotic leak were: hemodynamic protocol (rho= -0.546; p=0.071), preoperative albumin (rpbi=-0.373; p=0.011), preoperative hemoglobin (rpbi= -0.516; p=0.0001) and total crystalloids amount (rpbi= -0.560; p=0.0001). A logistic regression showed that preoperative hemoglobin (p=0.025) was the only independent predictor of anastomotic leak (Log-Likelihood=-10.01; Likelihood Ratio chi2 =28.14; p<0.0001) (fig. 4). Post-regression receiver operating characteristic analysis (ROC) showed an area under ROC curve of 0.88 and there was a significant association between anastomotic leak and hospital length of stay (rpbi=0.692; p<0.0001).

Conclusions: the addition of arterial dynamic elastance to hemodynamic protocol did not change the lactate levels at the end of major abdominal surgery but it produced a significant effect on fluid balance and vasoactive drugs.

### A30 Clevidipine and inhibition of hypoxic pulmonary vasoconstriction: a case report

#### E. Bertoli ^1^, M. Vissani ^2^, G. Brunelli ^2^, M. Cascelli ^2^, A. Tarquini ^2^, F. Quaranta ^2^, G. Nicoletta ^2^

##### ^1^ Department of Anesthesia and Intensive care Unit, University of Perugia, Perugia, Italy; ^2^ Department of Anesthesia and Intensive Care Unit, San Giovanni Battista Hospital, Foligno, Italy

###### **Correspondence:** E. Bertoli

*Journal of Anesthesia, Analgesia and Critical Care 2023,* **3(Suppl 1):**A30

Background

Clevidipine is a new intravenous calcium channel blocker with favourable pharmacokinetic properties approved for perioperative management of hypertension. We described a case of reversible hypoxemia caused by clevidipine-induced pulmonary vasodilatation in a patient who developed hypertension after abdominal surgery. Written informed consent for the publication was obtained by the patient.

Case report

A 62-year-old man was admitted in our ICU after gastrectomy for spontaneous gastric rupture. His medical history revealed systemic artery hypertension in medical treatment. After weaning from mechanical ventilation and extubation, the patient was treated with high-flow nasal cannula for hypoxemia due to postoperative bilateral lung consolidations diagnosed by computerized tomography. The respiratory function gradually improved until reaching a PaO2/FiO2 ratio of 250 mmHg. The following clinical course was complicated by onset of severe hypertension (190/100 mmHg). Because of esophago-jejunal anastomotic leakage, the patient could not assume oral medications, so we decided to start intravenous clevidipine with a prompt control of pressure (140/80 mmHg) at infusion rate of 2 mg/h. However, immediately after initiation of clevidipine’s infusion the patient developed hypoxemia (PaO2/FiO2 ratio of 130 mmHg). The echographic lung examination excluded new causes of hypoxemia (pneumothorax, pulmonary edema) but confirmed bilateral consolidations similar to previous computerized tomography control. A bedside transthoracic echocardiogram did not revealed right heart dilatation or intracardiac shunting. Having ruled out other obvious causes of hypoxemia, we hypothesized that the clevidipine inhibition of hypoxic pulmonary vasoconstriction (an important compensation mechanism in our patient in consideration of his bilateral lung consolidations) magnified pulmonary shunt and it was the probable explanation of sudden hypoxemia. In fact the following discontinuation of the drug infusion promptly resolved hypoxemia and the patient reached a PaO2/FiO2 ratio of 250 mmHg. We reported this case as a suspected adverse drug reaction.

Conclusion

Based on our case report and analogue ones described in literature [1], we suggest that clinicians must be aware of the possibility of hypoxemia during clevidipine infusion. The probable/possible mechanism involved is the shunt due to clevidipine-induced pulmonary vasodilatation like other dihydropyridine calcium channel blockers [2]. We believe that clevidipine infusion should be used carefully in patients with hypoxic pulmonary vasoconstriction.

References

A) Short JH, Fatemi P, Ruoss S. Clevidipine-induced extreme hypoxemia in a neurosurgical patient: a case report. A A Pract. 2020 Jan 15;14 (2): 60-62.
B) Simonneau G, Escourrou P, Duroux P. Inhibition of hypoxic pulmonary vasoconstriction by nifedipine. N Engl J Med 1981;304: 1582-1585.

### A31 High-flow oxygen therapy vs tracheal intubation during laryngeal microsurgery under general anesthesia: preliminary results of a prospective non-inferiority randomized controlled trial

#### I. Battista, F. Della Sala, A. Piersanti, F. Sbaraglia, G. Spinazzola, D.M. Micci, A. Fabretti, G. Bernardi, C. Memoli, M. Rossi

##### Fondazione Universitaria Policlinico A. Gemelli IRCCS - Università Cattolica del Sacro Cuore, Roma, Italy

###### **Correspondence:** I. Battista

*Journal of Anesthesia, Analgesia and Critical Care 2023,* **3(Suppl 1):**A31

Background

High-Flow Nasal Cannula Oxygenation (HFNCO) is an open-loop oxygenation system that uses flows of up to 70 L/min of 100% oxygen through the Optiflow THRIVE TM apparatus (Fisher and Paykel Healthcare Ltd, Auckland, New Zealand), increasingly used as an alternative to tracheal intubation in patients undergoing short-term general anesthesia.

The efficacy of HFNCO in apnoic patients undergoing laryngeal surgery is debated: on the one hand it allows minimal manipulation of the airways, it expands the surgical field and it proved to be not inferior to tracheal intubation in maintaining oxygen saturation, on the other, it has been associated with a higher incidence of hypercarbia, acidosis and need for rescue maneuvers of airway management.

Methods

Patients aged 18 to 69 years, American Society of Anaesthesiologists (ASA) status I and II undergoing laryngeal microsurgery under general anesthesia and neuromuscular blockade were randomized to either apnoic oxygenation (THRIVE group) or tracheal intubation (Control group).

Primary aim will be evaluation of changes in frontal cerebral tissue oxygen saturation (SctO 2 ) through Near-Infrared Spectroscopy (NIRS) technology in the two groups. Secondary objectives will be the noninferiority of THRIVE compared to tracheal intubation in term of success rate of the technique. Success rate will be defined as partial pressure of carbon dioxide (PaCO 2) <= 65 mmHg and/or peripheral oxygen saturation (SpO 2 ) >= 94% throughout the procedure in the absence of

adverse events.

Results

First 10 patients of 30 expected at completion of the study were included in this preliminary analysis.

Patients characteristics are listed in Table 1;

no significant differences were observed in both groups. Median (IQR) surgery and anesthesia duration, as well as baseline StcO 2, SpO 2, PaCO 2 and hemodynamic parameters were comparable between the two groups (Table 2).

For the primary outcome StcO 2 values were significantly different and higher in the THRIVE group compared to the Control group, consistently with the higher flows of O 2 received (mixed-effect linear regression model, P < 0.001).

For the secondary outcome, success rate of THRIVE was 60% compared to 100% for tracheal intubation. No patient in both groups showed SpO 2 <= 94%; median (IQR) peak PaCO 2 was 61 (60, 61) mmHg in the THRIVE group compared to 43 (41, 45) mmHg in the Control group. Two (40%) patients reached PaCO 2 levels > 65 mmHg and 100% of patients in the THRIVE group developed transient respiratory acidosis with no hemodynamic instability during surgery compared to none in the Control group.

No patient in both group reported dyspnoea at the end of anesthesia; no complication occurred during hospital stay and all patients were discharged on first postoperative day.

Conclusion

THRIVE oxygenation should be used with cautious monitoring during laryngeal microsurgery under general anesthesia and neuromuscular blockade. THRIVE was successful in maintaining oxygen saturation but incidence of hypercarbia and transient respiratory acidosis were more frequent during HFNCO compared with tracheal intubation.

**Table 1 (abstract A31).** See text for description

Baseline characteristics	THRIVE group (n=5)	Control group (n=5)
Age, y	30 (25, 35)	38 (36, 40)
Height, cm	170 (170, 174)	174 (168, 180)
Weight, Kg	80 (74, 80)	75 (67, 82)
Body mass index, Kg/m^2^	25 (24, 27)	24 (23, 25)
ASA status		
1	3 (60)	3 (60)
2	2 (40)	2 (40)
Medical history		
Smokers	2 (40)	3 (60)
Indication to laryngeal microsurgery		
Vocal cord polyp	3 (60)	4 (80)
Vocal cord cyst	1 (20)	0 (0)
Biopsy	1 (20)	1 (20)

Demographic, Baseline and Surgical Characteristics of the Study Population (N=10). Data are presented as median (25th to 75th IQR). *THRIVE* Transnasal Humidified Rapid Insufflation Ventilatory Exchange, *ASA* American Society of Anaesthesiologists

**Table 2 (abstract A31).** See text for description

	THRIVE group (n=5)	Control group (n=5)	***P*** value§
SctO_2_			
Baseline	72 (70, 77)	71 (68, 72)	0.460
At the end of surgery	93 (92, 94)	71 (68, 73)	0.015
SpO2			
At induction of anesthesia	100 (99, 100)	99 (98, 100)	0.100
At the end of anesthesia	100 (99, 100)	98 (98, 99)	> 0.999
Incidence of SpO_2_ ≤ 94%	0 (0)	0 (0)	> 0.999
Lowest pH	7.31 (7.27, 7.36)	7.40 (7.38, 7.43)	< 0.001
Incidence of pH < 7.30	5 (100)	0 (0)	0.008
pH at discharge from PACU	7.42 (7.41, 7.44)	7.40 (7.39, 7.42)	0.387
PaCO_2_, mmHg			
Baseline	35 (34, 36)	34 (33, 37)	> 0.999
At the end of anesthesia	49 (46, 50)	43 (42, 44)	< 0.048
Total monitoring time	48 (46, 48)	39 (36, 42)	0.018
Incidence of PaCO_2_ > 65 mmHg	2 (40)	0 (0)	0.444
Peak PaCO_2_, mmHg	61 (60, 61)	43 (41, 45)	0.019
PaCO_2_ at discharge from PACU, mmHg	36 (36, 41)	40 (37, 42)	0.533
Mean blood pressure, mmHg			
At induction of anesthesia	100 (98, 106)	95 (90, 97)	0.190
At the end of anesthesia	94 (83, 97)	96 (95, 97)	0.533
At discharge from PACU	93 (87, 103)	94 (90, 100)	0.904
Heart rate, bpm			
At induction of anesthesia	96 (80, 100)	72 (65, 77)	0.109
At the end of anesthesia	82 (70, 83)	65 (61. 70)	0.381
At discharge from PACU	75 (71, 82)	74 (66, 79)	0.458
Duration of surgery, min	12 (10, 12)	14 (12, 15)	0.262
Duration of anesthesia, min	21 (20, 24)	26 (24, 28)	0.190
Borg dyspnea score at the end of surgery	0 (0, 0)	0 (0, 0)	> 0.999

Comparison of Randomized Groups on perioperative variables of oxygenation and ventilation. Data are presented as N (%), mean (SD) or median (IQR). *THRIVE* Transnasal Humidified Rapid Insufflation Ventilatory Exchange, *LMA* Laryngeal Mask Airway, *SctO2* cerebral tissue oxygen saturation, *SpO2* pulse oxygen saturation, *PACU* Post-Anesthesia Care Unit, *PaCO2* partial pressure of carbon dioxide. P value corresponded to Wilcoxon rank sum test or Fisher’s exact test

### A32 Systematic review about improvement strategies for the prevention of postoperative AKI in elderly patients undergoing major surgery

#### L. Baccari, A. Fruncillo, F. Barra, D. Giordano, L. D'Angelo, M.R. La Rocca, C. Chiumiento, F. Chiumiento

##### UOC Anestesia E Rianimazione PO Eboli ASL Salerno, Eboli, Italy

###### **Correspondence:** L. Baccari

*Journal of Anesthesia, Analgesia and Critical Care 2023,* **3(Suppl 1):**A32

Background

Acute kidney injury (AKI) is a well-known and frequent complication in elderly patients after major surgery, causing significant morbidity and mortality, as well as increased health care costs. AKI is a frequent complication in elderly surgical population because it presents a reduced renal functional reserve. While preoperative AKI risk factors (hypertension, diabetes mellitus, known chronic kidney disease) and pathophysiological mechanisms of AKI are well understood, the management of perioperative AKI is still a matter of debate because the therapeutic interventions are often implemented late, particularly only after clinical evidence of decreased urine output, elevated serum creatinine levels or decreased glomerular filtration rate. In any case, all the delayed therapeutic approaches act on already existing organ damage.

Materials and methods

This systematic literature review aims to identify relevant literature from 2010 to 2022, published on the English-language PubMed database, gathering quantitative, qualitative and mixed information. The inclusion criteria of the studies examined were based on the collection of AKI prevention strategies to reduce morbidity and mortality in elderly patients undergoing major surgery.

Results

Based on the inclusion criteria, 143 articles were identified including 32 review articles, 18 systematic reviews, 31 randomized trials, 28 meta-analyses, 14 case-control studies, 32 clinical trials of which 18 were multicentres.

The scientific literature reviewed identified preoperative, intraoperative, and postoperative strategies for meaningful prevention of AKI. Among the preoperative ones literature detects:1. evaluation of the pharmacological therapy that the elderly patient takes with particular attention to NSAIDs and ACE inhibitors;2. preoperative fluid therapy plan which must aim at a hydrated and euvolemic patient; 3. encourage patients to drink clear fluids up to 2 hours before induction of general anesthesia. Among the intraoperative strategies, literature detects: 1. goal direct fluid therapy guided by hemodynamic monitoring with study of the patient's reactivity to fluids; 2. hemodynamic stability with MAP > 65 mmHg; 3. use of crystalloids and balanced hydroelectrolyte solutions and reduction of physiological solutions to 0.9%; 4. close monitoring of intraoperative blood glucose, avoiding excessive glycemic variability. Among the postoperative ones:1. postoperative fluid therapy plan with the aim of avoiding postoperative hypotension 2. avoiding the use of contrast media 3. early oral fluid intake 4. function monitoring. Furthermore,the scientific literature has shown that efforts should also be focused on early diagnosis through the use of point of care.

Conclusions

The scientific literature has demonstrated that efforts should be focused on prevention through preoperative, intraoperative and postoperative strategies in association with an early diagnosis of AKI. The KDIGO Care Bundle will be guided by careful monitoring of renal function through the traditional markers of renal function (serum creatinine, eGRF and diuresis),but also through the use of urinary and blood biomarkers which allow for the diagnosis of stress before organ damage has occurred: Insulin-like growth factor binding protein 7 (IGFBP) and tissue inhibitor of metalloproteinase-2 are two urinary biomarkers of cell cycle arrest that can be used to predict risk of developing AKI after major surgery (AKI risk assessment), Proenkephalin A119-159, neutrophil gelatinase-associated lipocalin (NGAL), interleukin-18, proteinCbinding factor and soluble thrombomodulin.

### A33 To be or not to be: a score for the evaluation of surgical and intensive care pathway in the perioperative multi-organ oncological patient

#### L. Andresciani ^1^, F. Galdini ^1^, C. Cariddi ^1^, C. Andresciani ^3^, S. De Summa ^2^, C. Calabrò ^2^, M.G. Lorusso ^2^, R. De Luca ^2^, M. Simone ^2^, G. Napoli ^2^, G. Carravetta ^2^, G. Mastrandrea ^2^

##### ^1^ Università degli Studi di Bari Aldo Moro, Bari, Italy; ^2^ IRCCS Istituto Tumori Giovanni Paolo II, Bari, Italy; ^3^ Università degli Studi La Sapienza, Roma, Italy

###### **Correspondence:** L. Andresciani

*Journal of Anesthesia, Analgesia and Critical Care 2023,* **3(Suppl 1):**A33

Introduction

Multiorgan oncological patient’s therapeutic pathway needs usually both surgical complex techniques and anesthesiologic and intensive-care support due to provide its effectiveness; in this specific setting, also the choice of a mild-high risk surgical approach could be challenged according to patient’s clinical conditions.

Moreover, current literature lack in evaluation’s score useful for the whole perioperative period’s assessment: this need creation and validation of a new tool, named PERIDIA-score, developed by a multidisciplinary team, from the Cancer Institute Giovanni Paolo II in Bari.

Primary Data acquired from PERIDIA01-study, a retrospective-perspective and still ongoing study, showed the cut-off of 13.5/48 points for complications’ onset (1) with a good sensitivity and specificity; statistical analysis performed on a larger sample strongly confirmed this cut-off and strengthened the idea of its predictivity role in the decision-making process.

Materials and methods

A cohort of 199 patients who underwent elective, non-palliative, turaco-abdominal-peri diaphragmatic-sovramesocolic surgery in the surgical ward of our Institute and who needed Post-Operative ICU care was extracted from the 2018-2019 data collection.

According to previous investigation, a ROC analysis was accomplished to confirm both comprehensive PERIDIA-score’s and PERIDIA-partials’ (PERIDIA pre, intra and post) predictability. The same methodology was also applied to single items for their significance assessment and to the sum of PERIDIA-pre and intra for their complications’ onset predictivity evaluation.

Finally, a multivariate logistic regression was implemented which underlined the most significant items.

Results

Primary results highlighted a quite good score’s predictivity of complications’ occurrence during surgical ward stay (NPV 0.765 and AUC 0.805). Also, the ROC analysis performed on the PERIDIA score in the pre intensive period (expressed as PERIDIA-pre + PERIDIA-intra) showed good predictivity value with a threshold of 12.5/32 points for complications’ onset in post-operative ICU stay (NPV 0.722, AUC 0.749).

Moreover, the multivariate logistic regression performed on this sample confirmed statistical significance of HR and Breathing but underlined Frailty as potentially significant item in predicting complications’ onset instead of Nutritional Status.

Discussion

The overall PERIDIA score showed quite good complications’ predictivity for oncological patient in this surgical setting; therefore, this data could support the score application in clinical practice as a useful evaluation’s tool of the whole care pathway in the specific setting of cancer patient.

Moreover, the predictivity of pre intensive PERIDIA could be focused as a guide to support some clinical choice and to achieve tailored treatments in post-operative ICU.

Thus, future steps of this work require single items’ improvement of each assessment (pre intra and post operative phase) to increase their significance and strengthen the overall score’s predictivity, also suggesting a possible role in the decision-making process not only for peri diaphragmatic sovramesocolic oncological surgery, but also for major oncological surgery pathway.

Ethical Approvement

The study was reviewed and approved by the Ethic Committee of IRCCS Istituto Tumori Giovanni Paolo II- Bari, Italy (Prot. N. 326/CE 30/7/2020).

References

1. Andresciani L ., Mastrandrea G. et al. Front oncol. 2021 Oct 11;11:733621

**Fig. 1 (abstract A33).**
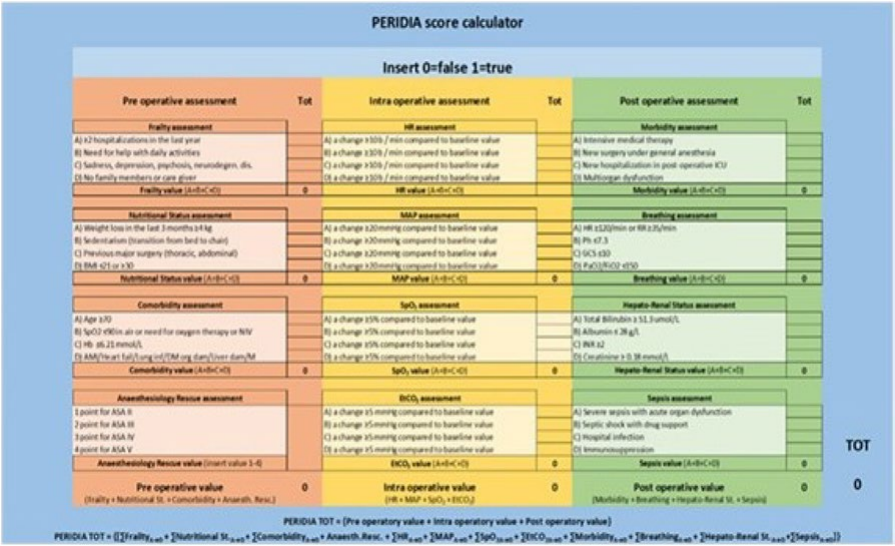
PERIDIA-score calculator. The figure shows the Peridia score calculator. It is divided into 3 scores (pre operatory score, intra operative score, post operative score) each of which expressing a score from 0 to 16 for a maximum of 48 points. Pre operatory score consists in 4 scores, to each of which a result from 0 to 4 points can be assigned. Intra operative score assesses the variation of 4 vital parameters commonly used during the monitoring of general anaesthesia (heart rate or HR, mean arterial pressure or MAP, saturation or SpO2, capnometry or EtCO2). Post operative score concerns the period of hospitalization in the ICU. It consists in 4 scores, to each of which a result from 0 to 4 points can be assigned

**Fig. 2 (abstract A33).**
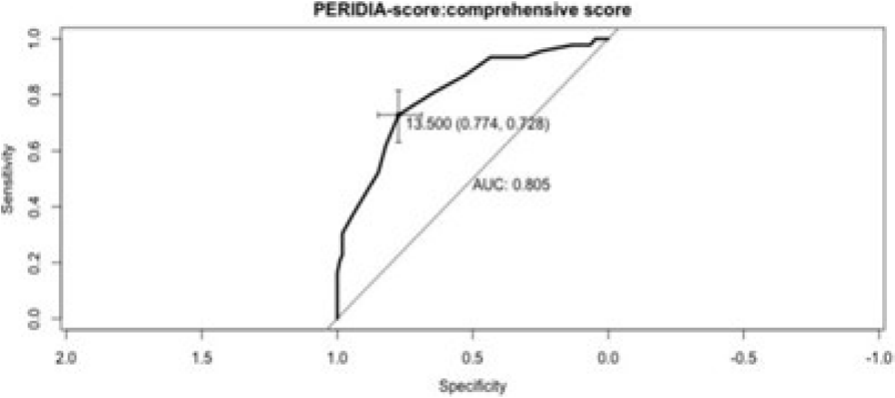
Roc analysis on the comprehensive PERIDIA-score. The figure shows the ROC curve obtained from statistical analysis performed on the complete sample (2018-2019); the threshold of 13.5/48 points (AUC 0.805, sensitivity 0.774 and specificity 0.728)

**Fig. 3 (abstract A33).**
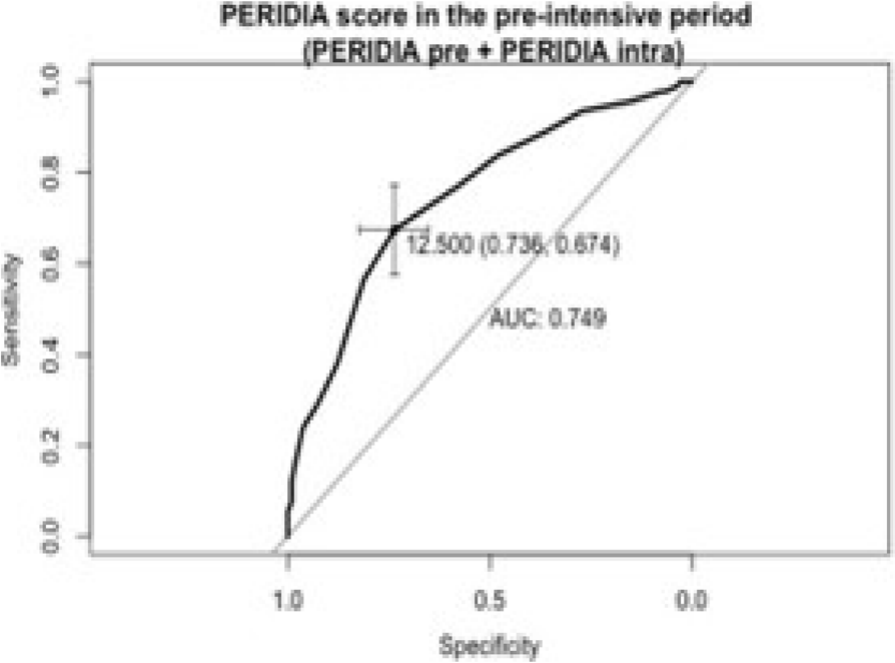
Roc analysis on the pre intensive PERIDIA-score. The figure shows the ROC curve obtained from statistical analysis performed on the sum of PERIDIA pre and PERIDIA intra score; threshold for complications ‘onset is 12.5/32 points (AUC 0.749) with a sensitivity of 0.736 and a specificity of 0.674

## Loco-regional Anesthesia

### A34 Transverse Abdominal Block with Anterior Approach for the treatment of Crural Hernia in SCA patient

#### E. Trimarchi, S. Di Stefano

##### Policlinico Universitario G. Martino, Messina, Italy

###### **Correspondence:** E. Trimarchi

*Journal of Anesthesia, Analgesia and Critical Care 2023,* **3(Suppl 1):**A34

Background. 83-year-old patient, BMI 31, history of hypertension, hypercholesterolemia, aortic-myocardial disease, mild mitral and aortic insufficiency, chest pain exacerbated efforts for about 8 years, in treatment with Prasugrel and Cardio-Asa, reaches the Emergency Department for angina/dyskinetic symptoms, with diagnosis of NSTEMI. Blood chemistry tests show elevation of specific myocardial enzymes: Troponin T 966 pg/ml, Myoglobin 151 ng/ml. Performs coronarography that highlights severe obstructive, calcific and coronary disease, with critical stenosis of the common trunk, multiple critical stenosis in the proximal and middle anterior descending artery and the first marginal branch. During the stay he presents intestinal obstruction from paralytic ileum on left crural hernia clogged, painful and not reducible manually. The surgical evaluation indicates the treatment in an unavoidable emergency by vascular compression of the loops in prison.

Case Report: The patient arrived in S.O. alert and collaborating, in spontaneous breath, VAS 5. HR 92 b/min, NIBP 145/75 mmHg, spo2 94%. Signed the informed consent, we proceed to Trasversus Abdominis Plane Block (TAP block anterior) to anesthetic dosage, performed by ultrasound with linear probe with ultrasonic needle 100 mm, made difficult by large abdominal meteorism. Fast-acting local anaesthetics (AL) (Mepivacaine 1%, 20 ml) and long-acting anaesthetics (Ropivacaine 5%, 20 ml) were used and 4 mg of dexamethasone and 70 mcg of clonidine were added as adjuvants to increase the duration of the sensory block. Verified the effectiveness of the block with both tactile and pain stimulation, the surgeon proceeds with the repair of crural hernoplasty with open technique. Patient alert and collaborating, in spontaneous eupnoic breath in ambient air enriched in O2 through nasal goggles with FiO2 28%. Hr 84 b/min, NIBP 145/75 mmHg, spo2 97%. VAS 3 despite viscera manipulation and numerous attempts at manual bowel reduction. At the end of the procedure, the patient is returned to UTIC for the rest of the stay.

Conclusions. Because of the patient’s comorbidities both neuroaxial and general anesthesia would have required hospitalization in an intensive/resuscitation environment for the risk of massive intra or post-operative cardiac failure. With the single execution of the TAP block the disconfort of the patient was derisory, tied mostly to the manipulation of the intestinal loops, than to the surgical act itself. At 24 hours from the procedure, the patient was hemodynamic stability, with VAS 1, without request for analgesics in the postoperative stay, treatable abdomen with the presence of peristalsis.

Informed consent to publication was obtained together with consent to the anesthesiology procedure.

### A35 Case report: subarachnoid anesthesia with the use of ropivacaine in a pediatric patient with favism

#### U. Tozzi ^1^, D. Arminio ^1^, C. Buonavolonta' ^1^, A. d'Elia ^1^, C.A. Di Lascio ^1^, P.A. Landri ^1^, N. Manzione ^1^, A. Pisapia ^1^, R. Sicilia ^1^, C. Chiumiento ^2^, M. Crisconio ^2^, F. Chiumiento ^1^

##### ^1^ ASL Salerno P.O. Battipaglia, Battipaglia, Italy; ^2^ Scuola Di Specializzazione Anestesia E Rianimazione Universita' Di Salerno, Salerno, Italy

###### **Correspondence:** U. Tozzi

*Journal of Anesthesia, Analgesia and Critical Care 2023,* **3(Suppl 1):**A35

INTRODUCTION

G6PD deficiency is estimated to affect approximately 400 million people.

It is a hereditary disease linked to the X chromosome, therefore more frequent in men. In these subjects the first reaction of the pentose phosphate pathway is not carried out efficiently, resulting in a lack of NADPH (Nicotinamide-Adenine-Dinucleotide-Phosphate).

Patients with G6PD deficiency can be very sensitive and vulnerable to oxidative stress caused by ingestion of broad beans, some drugs, infections, metabolic imbalances. with clinical manifestations such as haemolytic anemia in adults and severe jaundice in newborns.

CLINICAL CASE

Patient of 16 years, female, weight 55 kg, affected by favism, with surgical indication for umbilical hernioplasty.

It was decided to practice subarachnoid anesthesia, performed with a 27G Withacre needle at the L2-L3 level, with Ropivacaine 12 mg in a volume of 2.2 ml since general anesthesia in these patients often makes it difficult to identify a haemolytic crisis because symptoms such as hypotension, could be attributed to other causes.

Furthermore drugs such as Isoflurane, Sevoflurane and Bupivacaine are able to inhibit the G6PDH enzyme in vitro as well as Benzodiazepines; for this reason its use is not recommended.

During the operation, the vital parameters were always stable and normal. The surgery was performed with excellent compliance on the part of the little patient and the duration of anesthesia was estimated to be more than two hours. The post-operative course was also free from complications and the patient was discharged from the hospital on the third day.

Upon discharge, the patient was provided with the telephone number to contact the anesthesiologist in case of problems since the haemolytic crisis can occur up to 7 days. after anesthesia.

DISCUSSION AND CONCLUSIONS

The most effective perioperative management strategy is to prevent hemolysis by avoiding general anesthesia and bupivacaine in the subarachnoid if possible. For postoperative pain, it is advisable to use codeine/codeine derivatives, fentanyl and ketamine.

CONFLICTS OF INTEREST

None of the authors have conflicts of interest.

Informed consent to publish had been obtained

References

1. Cicvaric A et al.: Management of Anesthesia and Perioperative Procedures in a Child with Glucose-6-Phosphate Dehydrogenase Deficiency - J Clin Med . 2022
2. C L Smith, et al.: Anesthesia and glucose-6-phosphate dehydrogenase deficiency. A case report and review of the literature – Anesthesia. 1987 Mar
3. A. Elyassi et al.: Perioperative management of the glucose-6-phosphate dehydrogenase deficient patient: a review of literature - Anesth Prog . 2009

### A36 The effect of intrathecal Midazolam added to Bupivacaine and Morphine for spinal anesthesia in orthopedic patients

#### I. Piccione ^1^, D. Cirillo ^1^, E. Spasari ^1^, I. Russo ^2^, M.S. Barone ^1^, A. D'Abrunzo ^1^, M. Ianniello ^1^, F. Viti ^1^, N. Logrieco ^1^, A. Coviello ^1^

##### ^1^ Department of Neurosciences, Reproductive and Odontostomatological Sciences, University of Naples Federico II, Naples, Italy; ^2^ Department of Public Health, School of Medicine, University of Naples Federico II, Naples, Italy

###### **Correspondence:** I. Piccione

*Journal of Anesthesia, Analgesia and Critical Care 2023,* **3(Suppl 1):**A36

Background

As surgical techniques and pharmacology advance, pain management in orthopedic surgery patients continues to evolve [1]. Multimodal analgesic regimens that target numerous pain pathways may provide the best pain management and reduce opioid use and related side effects [2]. Adjuvants in spinal anesthesia have been used to prolong the quality and duration of its effect, including analgesic one [3,4]. This study aims to prove that intrathecal injections of Midazolam, binding gamma-aminobutyric acid-A (GABAA) receptors in the spinal cord, lead to the potentiation of analgesia.

Materials and methods

This is a retrospective comparative study performed at the Department of Orthopedics and Traumatology of the AOU Federico II in Naples, Italy. Data about patients who underwent spinal anesthesia between September 2022 and April 2023 were retrieved from the department's archive. Inclusion criteria were patients scheduled for elective major orthopedic surgery with spinal anesthesia, aged from 18 years on with a body mass index of 18 – 45 kg/m2 and an American Society of Anesthesiologists physical status classification of I to IV. After obtaining informed consent, patients were clustered into two groups: “Midazolam group” (12 mg Bupivacaine + 150 μg Morphine + 2 mg Midazolam) and “Control group” (12mg Bupivacaine + 150 μg Morphine). In both groups, postoperative pain management included the administration of 1 g intravenous Paracetamol every 8 hours and as rescue analgesia (VAS greater than or equal to 5) 30 mg Ketorolac or, in renal impairment, 10 mg Oxycodone. The study's primary outcome was postoperative pain estimated with Visual Analogue Scale (VAS)[5]. Postoperative pain, itching, nausea, and vomiting were assessed immediately after (T0) the end of the surgery and then after 6 (T6), 12 (T12), 18 (T18), and 24 hours (T24). Furthermore, the level of intraoperative sedation with the Richmond Agitation-Sedation Scale (RASS) were evaluated [6].

Results

In the selected cohort, 20 (57%) patients were included in the Midazolam group (MG) and 15 (43%) patients in the Control group (CG). Average VAS scores were slightly lower in MG (3 at T18) than in CG (4 at T18) (Figure 1). Only 15% of the patients needed rescue analgesia. The level of intraoperative sedation was the same in both groups. The incidence of itching was 30% in both groups, while the incidence of nausea and vomiting was 35% in MG and 40% in CG.

Conclusion

The addition of intrathecal midazolam didn’t lead to statistically significant differences in the management of postoperative pain. The adjuvant neither produce an increase in the level of intraoperative sedation, nor a decrease of the incidence of side effects.

References

1. Giesa, M., Jage, J., & Meurer, A. (2006). Post-operative pain management in orthopaedic surgery and traumatology. Der Orthopäde, 35, 211-222.
2. Savoia, G., Alampi, D., Amantea, B., Ambrosio, F., Arcioni, R., Berti, M., ... & Mattia, C. (2010). Postoperative pain treatment SIAARTI Recommendations 2010. Short version. Minerva anestesiologica, 76(8), 657-667.
3. Suresh, S., Ecoffey, C., Bosenberg, A., Lonnqvist, P. A., De Oliveira, G. S., de Leon Casasola, O., ... & Ivani, G. (2018). The European Society of Regional Anaesthesia and Pain Therapy/American Society of Regional Anesthesia and Pain Medicine recommendations on local anesthetics and adjuvants dosage in pediatric regional anesthesia. Regional Anesthesia & Pain Medicine, 43(2), 211-216.
4. Sun, S., Wang, J., Bao, N., Chen, Y., & Wang, J. (2017). Comparison of dexmedetomidine and fentanyl as local anesthetic adjuvants in spinal anesthesia: a systematic review and meta-analysis of randomized controlled trials. Drug design, development and therapy, 3413-3424.
5. Breckenridge, J. D., & McAuley, J. H. (2011). Shoulder pain and disability index (SPADI). Journal of physiotherapy, 57(3), 197-197.
6. Ely, E. W., Truman, B., Shintani, A., Thomason, J. W., Wheeler, A. P., Gordon, S., ... & Bernard, G. R. (2003). Monitoring sedation status over time in ICU patients: reliability and validity of the Richmond Agitation-Sedation Scale (RASS). Jama, 289(22), 2983-2991.

**Fig. 1 (abstract A36).**
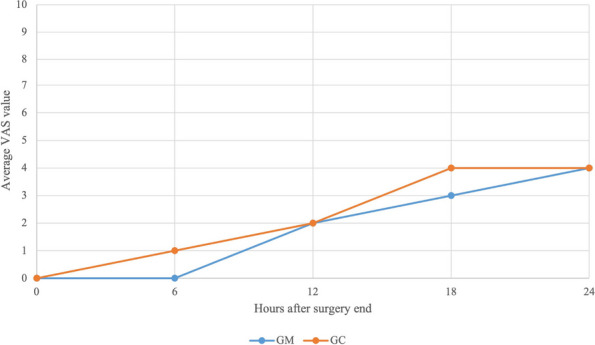
See text for description

### A37 Controlled hypotension during shoulder arthroscopy in beach chair position using Clevidipine

#### E. Pariani, B. Mascia, M. Mazzocchi, R. Pesando, D. Passador, A. Locatelli

##### IRCCS Policlinico San Matteo, Pavia, Italy

###### **Correspondence:** E. Pariani

*Journal of Anesthesia, Analgesia and Critical Care 2023,* **3(Suppl 1):**A37

Background: Arthroscopic shoulder surgery often requires the patient in beach chair position. Compared with open techniques, arthroscopy allows smaller incisions, faster recovery, and pain-free muscle contraction, and has cosmetic benefits. During arthroscopic shoulder surgery, clear visibility is essential(1). Various methods have been used to ensure clear visibility during surgery but controlled hypotensive anesthesia is still the most effective method. However, risks associated with reduction in cardiac output and cerebral perfusion which could result in serious cardiovascular events.

Clevidipine is an ultrashort-acting intravenous dihydropyridine that inhibits L-type calcium channels with high clearance, low distribution and a rapid shift to inactive metabolites by blood esterases. It was developed for short-term intravenous control of blood pressure through a selective effect on arterioles(2).

The purpose of our study is to provide the efficacy and safety of clevidipine to maintain controlled hypotension during shoulder arthroscopy in beach chair position in patients undergoing regional anesthesia and sedation.

Materials and Methods: The study is an open label, interventional study. All patients undergoing shoulder arthroscopy are eligible. Informed consent was collected. Interscalenic US-guided brachial plexus block and sedation (Propofol 0.5 mg/Kg/h) are administered. Information regarding clevidipine include the initial infusion rate, the average infusion rate, and the duration of administration. Hemodynamic information include the MAP, HR and NIRS (to evaluate cerebral oxygenation) during clevidipine infusion. Data were collected every 15 minutes.

Results: 8 patients were enrolled, age 52-76 years. Clevidipine infusion was started at 4 ml/h and tritrated until a target MAP of 60-65 mmHg was reached. The average maintenance infusion rate was 6.9 ml/h.

We found a significant reduction in MAP equal to an average of 20 mmHg from baseline, with no significant changes in HR and NIRS.

Intraoperative clevidipine infusion was stopped in 2 patients for excessive hypotension, with resetting of MAP and NIRS to baseline values almost 15 minutes after discontinuation. In one of these there was a significant decrease in NIRS (from 61 to 48), without neurological syntoms.

Conclusion: Clevidipine results effective and safe in maintaining controlled hypotension during shoulder arthroscopy without significant decreases in NIRS. Its haemodinamic profile allows arteriolar vasodilation without action on venous vasculature, thus maintaining cardiac output that is already reduced by the beach chair position. Both the onset and offset of action on MAP are rapid. These are preliminar results since only a few patients are enrolled in the study at this time, so further enrollments are necessary to confirm our data.

References

1. Kim JY, Song SH, Cho JH, Cho HR. Comparison of clinical efficacy among remifentanil, nicardipine, and remifentanil plus nicardipine continuous infusion for hypotensive anesthesia during arthroscopic shoulder surgery. J Orthop Surg (Hong Kong). 2017 May-Aug;25(2):2309499017716251. doi: 10.1177/2309499017716251. PMID: 28639533.
2. Keating GM. Clevidipine: a review of its use for managing blood pressure in perioperative and intensive care settings. Drugs. 2014;74(16):1947-1960. doi:10.1007/S40265-014-0313-6

### A38 Ultrasound Guided Bilateral Thoracic Erector Spinae Plane Block as the ONLY strategy for analgesia in Major Abdominal Laparotomic Surgery: a case report

#### Michela Limone, Giacomo Torretta, Angelo Storti

##### ^1^ Intensive Care Unit - AORN "San Giuseppe Moscati" Avellino

###### **Correspondence:** M. Limone

*Journal of Anesthesia, Analgesia and Critical Care 2023,* **3(Suppl 1):**A38

Epidural Analgesia (EA) and Paravertebral Block (PVB) are the regional techniques commonly considered as the gold standard for analgesia after abdominal laparotomy, despite of the complexity of the procedures and the high risk of complications.

Therefore, the erector spinae plane block (ESPB) could be a valid alternative. [1]

ESPB is a paraspinal fascial plane block, first described by Forero et al. in 2016.

It’s a relatively easy technique to perform with a lower risk of complications. The spread of the local anesthetic through the connective tissues and towards the spinal nerve roots cranially and caudally from the site of injection provides a multi-dermatomal sensory block. Furthermore, the transforaminal and epidural spread of the local anesthetic during ESP block has been described. It results in a blockage of the dorsal and ventral rami of thoracic and abdominal spinal nerves, thus ensuring both somatic and visceral analgesia and allowing a significant reduction in opioid consumption. [2,3,]

We report a case in which ESPB was successfully used as the only strategy for analgesia in Major Abdominal Laparotomic Surgery.

An 86-years old woman (65Kg;160cm), was scheduled for an open right hemicolectomy whit xifo-pubic incision. Her only comorbid condition were hypertensive heart disease and HBV+.

The patient declined epidural analgesia due to the perceived risks. After a discussion, she agreed to a bilateral ESP blockade.

The ESP block was performed before induction of anesthesia, after institution of IV access, electrocardiogram, invasive blood pressure, pulse-oximetry monitoring, TOF, Entropy and SurgicalPlethIndex (as monitoring of intraoperative pain)

The patient was placed in a sitting position and, using an aseptic technique, a high- frequency (12–15 MHz) linear-array transducer was placed in a longitudinal para- sagittal orientation. A 21G Needle (Pajunk SonoPlex 50mm) was inserted in-plane to the ultrasound beam and in a cranial-to-caudal direction to contact the tip of the T8 transverse process. Twenty milliliters of Ropivacaina 0.5% were injected. This procedure was repeated on the other side.

Anesthesia was induced whit Propofol 130mg, Sufentanil 10mcgr, Rocuronio 50mg and maintained whit Sevoflurane; according to the intraoperative SPImonitoring no more analgesics were needed.

Paracetamol 1g and Morphine 1mg were administered 40 minutes before awakening.

Upon awakening, patient was comfortable and had a numerical rating scale <3 at time 0 and after 6, 12 and 48h.

In summary, this case demonstrates the successful use of Thoracic ESPB for major open abdominal surgery, but more studies need to be conducted.

Informed consent to publish had been obtained.

References

1. Jeong YH, Jung JY, Cho H, Yoon HK, Yang SM, Lee HJ, Kim WH. Transverse abdominis plane block compared with patient-controlled epidural analgesia following abdominal surgery: a meta-analysis and trial sequential analysis. Sci Rep. 2022 Nov 29;12(1):20606. doi: 10.1038/s41598-022-25073-w. PMID: 36446941; PMCID: PMC9709047.
2. Kekul, O., Ustun, Y.B., Kaya, C. et al. Analgesic efficacy of the bilateral erector spinae plane block for colorectal surgery: a randomized controlled trial. J Anesth Analg Crit Care 2, 43 (2022). https://doi.org/10.1186/s44158-022-00073-4
3. Krishnan S, Cascella M. Erector Spinae Plane Block. [Updated 2023 Jan 8]. In: StatPearls [Internet]. Treasure Island (FL): StatPearls Publishing; 2023 Jan-. Available from: https://www.ncbi.nlm.nih.gov/books/NBK545305/

### A39 Laparoscopic hysterectomy under subarachnoid anesthesia

#### G. Grasso ^1^, G. Servillo ^1^, D. Gammaldi ^2^, D.P. Annaclaudia ^1^

##### ^1^ AOU Federico II, Naples, Italy; ^2^ Casa di Cura Tortorella, Salerno, Italy

###### **Correspondence:** D.P. Annaclaudia

*Journal of Anesthesia, Analgesia and Critical Care 2023,* **3(Suppl 1):**A39

In recent decades advances in technology and surgery have led to an increase in the number of operations performed in VLS, a new frontier of minimally invasive surgery with high precision that allows to operate with minimal trauma for organs and tissues through small incisions on the anterior abdominal wall; the images are enlarged and projected onto video.

The main problems are hemodynamic and respiratory alterations related to the Pneumoperitoneum and the Trendelemburg position that determine reduction of venous return and GC, risk of arrhythmias and increased airway pressures. These conditions have contraindicated in recent decades the use of neuroaxial anesthesia in laparoscopic procedures, resorting only to AG.

Anesthesiological strategy can make use of different strategies and the choice of technique depends on many factors: type of patient, preference of the anesthesiologist, expected duration of the intervention, preference of the surgical team.

The aim of this study is to evaluate the possibility of performing laparoscopic gynecological surgery under neuroaxial anesthesia and to evaluate the point of view of the anesthesiologist, surgeon and patient. This is a prospective cohort study comprising sixty-six patients undergoing laparoscopic surgery for benign pathology at the Obstetric Clinic of the Federico II University of Naples.

Patients were assigned to Group A (subarachnoid anesthesia) and Group B (general anesthesia). They were invited to participate in the study during preoperative counseling and it was obtained with written informed consent.

The primary outcome assessed was post-operative pain. Secondary outcomes included mobilization, length of postoperative stay, global patients and surgeons satisfaction.

Immediate postoperative pain was significantly lower in Group A, with no significant differences at 24h in the two groups. Secondary outcomes demonstrated early mobilization of patients, a reduction in post-operative pain with reduced use of opioids in the post-operative period, excellent satisfaction of the surgeon and patients in group A and reduction of hospitalization times (ERAS-protocols). In these patients, the VAS score averaged 3 during surgery.

Regional anesthesia has proven to be an effective choice for conducting anesthesia in benign gynecological surgery, reducing the impact of surgical stress, ensuring faster recovery without compromising surgical outcomes.

In addition, during the Sars-Covid19 pandemic, the ALR has allowed us to avoid airway management by offering greater protection to health workers.

Keywords: post-operative pain, loco-regional analgesia, benign gynecological surgery, Sars-Covid19-pandemic, ERAS-protocols

Laparoscopic gynecological surgery under minimally invasive anesthesia: a prospective cohort study. Giampaolino, Della Corte, Mercorio, Bruzzese, Coviello, Grasso, Del Piano, Bifulco

Laparoscopic surgery for benign adnexal conditions under spinal anaesthesia: Towards a multidisciplinary minimally invasive approach. Raimondo et al

### A40 Skin reducing mastectomy and immediate implant-based reconstruction under regional block in a patient with amyotrophic lateral sclerosis: case report

#### G. Fedele ^1^, M. Alonzi ^1^, C. Bonarrigo ^1^, P.P. Gaglioti ^2^, M. Covotta ^1^, E. Forastiere ^1^

##### ^1^ IRCSS Regina Elena Istituto Nazionale Tumori, Roma, Italy; ^2^ Università La Sapienza, Roma, Italy

###### **Correspondence:** P.P. Gaglioti

*Journal of Anesthesia, Analgesia and Critical Care 2023,* **3(Suppl 1):**A40

Keywords: Mastectomy, Breast Reconstruction, Regional Block, Amyotrophic lateral sclerosis

Background

Amyotrophic lateral sclerosis (ALS) is a neurodegenerative disease that results in progressive loss of upper and lower motor neurons. Weakness of respiratory muscles, difficulty swallowing and susceptibility to muscle relaxants pose challenges for anesthetists. General anesthetics depress ventilation and swallowing reflexes, thereby increasing the risk of regurgitation and hypoxia. Furthermore, ALS patients are highly sensitive to non-depolarizing muscle relaxants. Intravenous opioids may worsen the breathlessness of these patients. Despite the lack of widespread consensus on the ideal anesthetic approach to ALS patients undergoing major breast surgery, there are few noteworthy anesthetic considerations in this cohort.

Case Report

A 60-year-old female patient was scheduled for skin reducing mastectomy and breast reconstruction for ductal carcinoma in situ. Preoperative evaluation revealed a late-stage disease with speech impairment and mouth incompetence with hypersalivation and dysphagia.

After a careful risk-benefit analysis, involving a multidisciplinary team approach,

the patient successfully underwent the surgical procedure with regional block: pectoserratus block T5 with Ropivacaine 0.375% 30 ml and parasternal block T3 with Ropivacaine 0.375% 6 ml were performed with ultrasound-guided technique, adding Clonidine 150 mcg and Dexamethasone 8 mg to the anesthetic mixture.

Pain control was achieved with 1 g paracetamol three time per day and Ketorolac 30 mg as rescue therapy. The patient maintained VAS < 2. Perioperative course was uneventful and the patient was discharged on the second postoperative day without complications.

Conclusion

Patients with ALS present unique challenges for anesthesia providers. Recent studies highlight the benefit of local-regional techniques, discouraging predictable complications of general anesthesia in patients undergoing oncological surgeries.

The decision to perform anesthesia in this high-risk patient population should be based on analyzing patient’s risks against the potential benefits. In conclusion, the regional block allowed the performance of a safely and successful surgery with high patient satisfaction.

Consent to publish

Informed consent to publish had been obtained.

References

1. Carlson, G.W. (2005), Total Mastectomy Under Local Anesthesia: The Tumescent Technique. The Breast Journal, 11: 100-102.
2. Matsumoto, M. et al. Benefits in radical mastectomy protocol: A randomized trial evaluating the use of regional anesthesia. Sci. Rep. 8(1), 7815 (2018).

### A41 Bilateral ESP Block catheter for pain management in a patient with thrombocytopenia undergoing robotic cystectomy

#### F. Della Vecchia ^1^, S. Troili ^1^, S. Tullj ^1^, M. Vespasiano ^2^, A. Piroli ^1^, F. Marinangeli ^1^

##### ^1^ Department of MeSVA, University of L Aquila - San Salvatore Teaching Hospital of L Aquila, L'Aquila, Italy; ^2^ San Salvatore Teaching Hospital of L Aquila, L Aquila, Italy, L'Aquila, Italy

###### **Correspondence:** F. Della Vecchia

*Journal of Anesthesia, Analgesia and Critical Care 2023,* **3(Suppl 1):**A41

-introduction

Postoperative pain management of a patient with platelet deficiency undergoing robotic cystectomy may require a challenging multimodal approach due to concerns around the use of antalgic subarachnoid block (contraindicated with PLT <75x103). Therefore, we used a bilateral ESP Block catheter to manage both intra- and post-operative pain.

We collected the data from a single patient with a background of type 2 diabetes mellitus, new onset atrial fibrillation, splenomegaly, and thrombocytopenia due to increased splenic sequestration. Of note, the relevant drug history for the patient in question includes metformin and Edoxaban.

This study adhered to the STROBE Statement and the Declaration of Helsinki. Written informed consent was obtained from all subjects, or their proxies or legal surrogates

-materials and methods

Upon arrival of the patient in the pre-operating room, after having explained to him the chosen technique, ensuring his maximum collaboration, we begin the practical preparation for the execution of an eco-guided bilateral ESP Block. The patient is asked to assume a prone position. The procedure is carried out in a sterile field. The target area is identified by the transverse process (TP). The linear probe is positioned in a longitudinal parasagittal orientation, approximately 3 cm lateral to the spinous processes, allowing visualization of the adjacent transverse processes in a flat approach. After successful identification of the target, an 18-gauge echogenic needle is inserted using a craniocaudal approach. Once the fascial plane has been recognized, 15ml of local anesthetic (LA), such as Levobupivacaine 0.25% + Dexamethasone 4mg are injected bilaterally (T10- T12 level). After the firsts bolus of LA a bilateral Pajunk E-Cath was inserted in the actual targeted space created from the injected LA.

In the operating room, balanced general anesthesia is performed with Propofol 200mg, Rocuronium 50mg, Remifentanil TCI 0.5-1.5 ng/ml, Sevoflurane 2% (MAC 0.8) plus Morphine 2mg (patient weigh: 80kg). Analgesic starter with Paracetamol 1g and Ondansetron 4mg, thirty minutes before the end of the operation.

Data about intra- and post-operative pain from a single patient undergoing robotic cystectomy were analyzed. Intra-operative pain data included invasive IBP and CR. Data were collected prospectively to evaluate the pain medication needed and pain scores at 1, 4, 12 and 24h after surgery. NRS and Likert Scale was used to assess perceived pain.

-results

Our data, albeit limited, demonstrates that recurrent boluses of Levobupivacaine 0,25% 5ml did not affect IBP or CR significantly, and intravenous Remifentanil TCI was needed to manage intra-operative pain. However, in the post-operative course, the patient appears to respond well to our multimodal approach consisting of a bolus of Levobupivacaine 0,25% 5ml, Paracetamol 1g iv, Morphine 2mg iv. In the immediate post-operative recovery, the patient did not require any further analgesia other than the PCA with levobupivacaine 2.5mg/ml at 6 ml/hr.

-conclusions

Bilateral ESP block catheters seem to be an excellent analgesic option for pain management in robotic cystectomy, at least in those patients where antalgic subarachnoid anesthesia is not advisable (Plt <75*103) and/or there is concern regarding opioid use and side effects (1).

**Fig. 1 (abstract A41).**
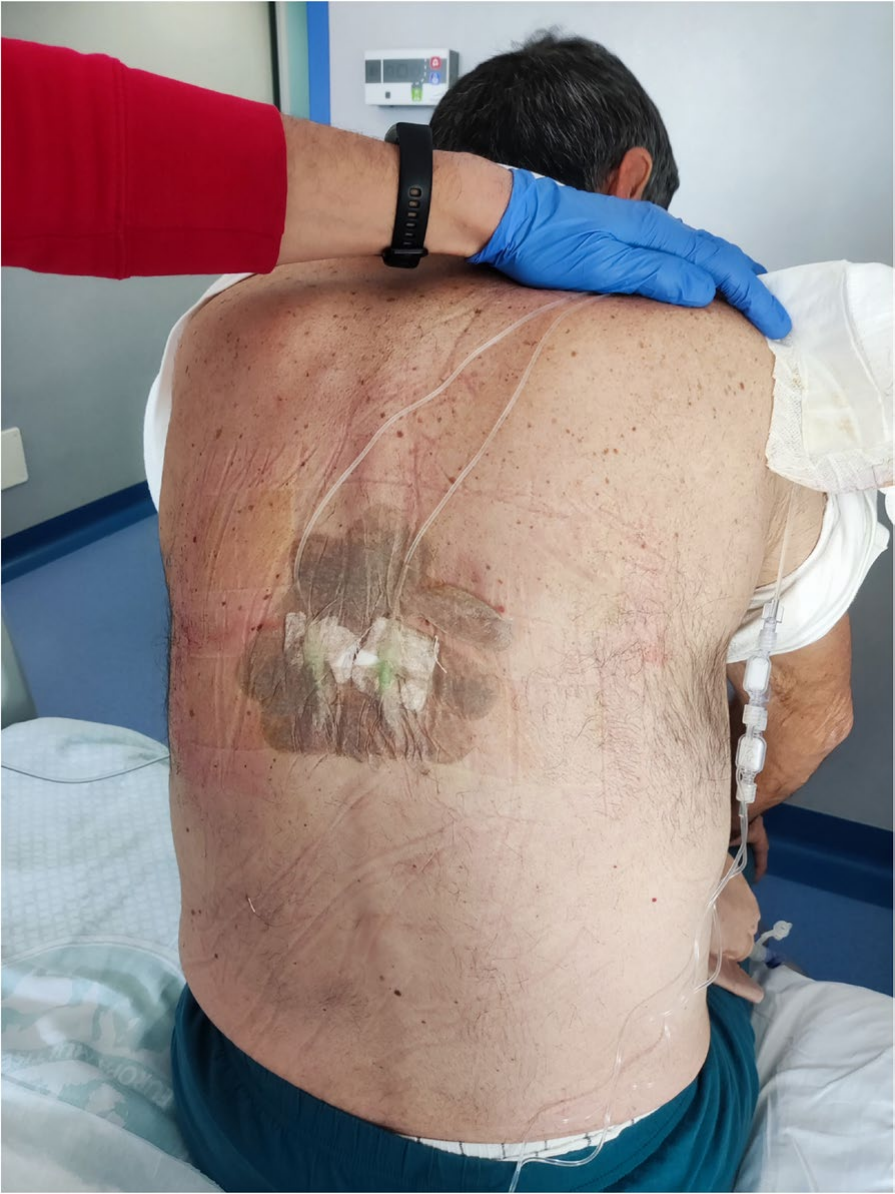
See text for description

### A42 Chronic Post-operatory Pain in oncological breast surgery: can Regional Anesthesia and a transitional pain service make a difference? Preliminary data of a prospective observational multicentric study

#### C. Deli ^1^, G. Abbiati ^1^, G. Belli ^1^, N. Paoletti ^1^, V. Torrano ^2^, R. Fumagalli ^1, 2^

##### ^1^ Department of Medicine and Surgery, University of Milan-Bicocca, Monza, Italy; ^2^ Department of Emergency and Urgency, Anesthesia and Intensive care Unit 1, ASST Grande Ospedale Niguarda, Milan, Italy

###### **Correspondence:** C. Deli

*Journal of Anesthesia, Analgesia and Critical Care 2023,* **3(Suppl 1):**A42

Background

Incidence of Chronic Post-operatory Pain (CPoP) in oncological breast surgery is around 30%^1^ , this percentage rises to 45% in patients who undergo axillary lymph node dissection. ICD-11 definition of CPoP includes all patients with persistent or rising pain after surgery or tissue damage that present pain beyond 3 months after healing. Many strategies are proposed to prevent this complication such as Regional Anaesthesia (RA) as a part of Multimodal Analgesia (MA). Poorly managed preoperative anxiety has reportedly slowed the postoperative recovery of breast cancer patients^2^, therefore the need to also treat this aspect.

The aim of the study is to evaluate the incidence of CPoP following the new perioperative multidisciplinary treatment RA-based and secondary to verify the effectiveness in enhancing patients' psychological and physical conditions.

Materials and methods

Patients are recruited at the pre-operatory evaluation with 3 questionnaires: the Quality of Recovery questionnaire (QoR-15 A-B), the Brief Pain Inventory (BPI) and Pain Catastrophizing Scale (PCS). Rest and incident Numeric Pain Rating Scale (rNRS - iNRS) is collected at 2-6-12-24h after surgery and at the time of discharge Qor-15 is proposed. Then, patients are called at 3-6-9-12 months to assess rNRS – iNRS and BPI.

Results

Preliminary data of 21 patients (Table 1), scheduled with a 3 months follow-up, showed that they had good pain control and a high quality of recovery in the first 24 hours following surgery (median rNRS at 24h= 0 IQR=4 and median iNRS at 24h=1 IQR=4) (Table 2).

Pre- and post-surgery QoR results were similar (Median preop-QoR-A=92 IQR=13 to postop=91 IQR=15 and preop-QoR-B=40 IQR=20 to postop=45 IQR=10) suggesting a good pain, anxiety and nausea and vomiting control with a fast recovery (Image 1). MA with RA permitted a low opioid dose during surgery (Remifentanil mean value for conservatory surgery was 364.5±209.1 mcg and reconstructive surgery was 1442± 434.2 mcg). Furthermore, only 1 patient (4,7%) needed morphine after surgery and that was justified by the fact that this patient underwent reconstructive surgery (285 minutes) and bilateral prosthesis positioning. Anxiety and depression were related to pain perception and 75% of the patients who needed rescue therapy (4; 19%) had PCS>10.

CPoP may be also well controlled months following surgery: patients have reported good pain management (rNRS median=0 IQR=1, iNRS median=0 IQR=3, BPIsev median=0 IQR=12) and full recovery of their daily activities (BPIint median=0 IQR=33). 4 patients (19%) at 3 months had CPoP but 75% of them had axillary lymph node dissection.

Conclusions

Despite further studies are needed to confirm these results, a transitional pain service and a RA-based pain management seems to enhance recovery from surgery and reduce the incidence of CPoP.

Trial registration

Current Controlled Trials ID 603-11102022 AN S00049/2022

References

1. Villa G, et al. Chronic pain after breast surgery: incidence, associated factors, and impact on quality of life, an observational prospective study. Perioper Med (Lond). 2021 Feb 24.
2. Tola YO, et al. Effects of non-pharmacological interventions on preoperative anxiety and postoperative pain in patients undergoing breast cancer surgery: A systematic review. J Clin Nurs. 2021 Dec.

**Table 1 (abstract A42).** Clinic and demographic data

Patients (N 21)	Mean±SD
Age	59.5±14.9
Sex	
Male	1 (4.8%)
Female	20 (95.2%)
BMI (Kg/m^2^)	23.6±4
ASA	
I	1 (4.8%)
II	19 (90.5%)
III	1 (4.8%)
Pain before surgery	2 (9.5%)
PCS >10	10 (47.6%)
Type of surgery	
Mastectomy	10 (47.6%)
Quadrantectomy	10 (47.6%)
Tumorectomy	1 (4.8%)
Plastic reconstructive surgery	4 (19%)
Surgical duration (mins)	
Without reconstructive surgery	96.8±32.6
With reconstructive surgery	281.2±32.5

**Table 2 (abstract A42).** NRS pain scores at rest and movement

Measurement time	Median (Q1-Q3)
Rest	
2h	3 (0-9)
6h	2 (0-9)
12h	0 (0-5)
24h	0 (0-4)
Movement	
2h	3 (2-9)
6h	2 (1-9)
12h	1 (0-5)
24h	1 (0-4)

**Image 1 (abstract A42).**
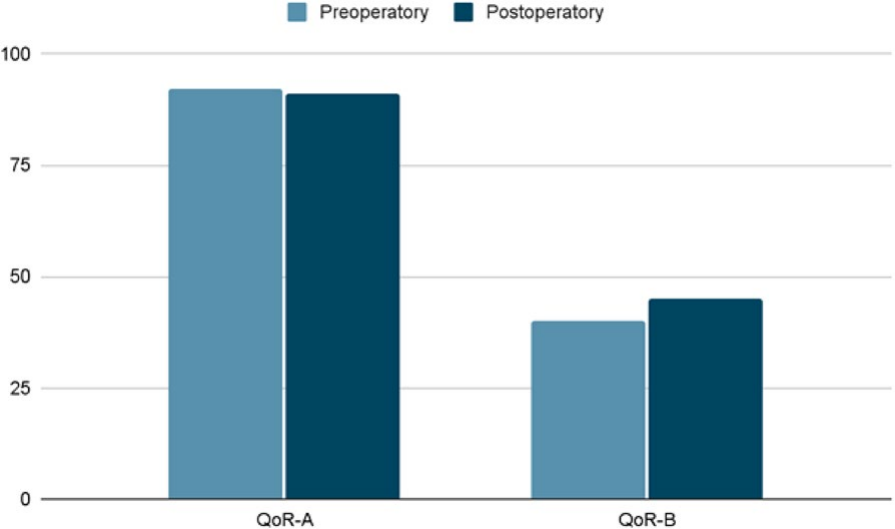
See text for description

### A43 Erector spinae plane block plus Paravertebral block as primary regional anesthesia technique for awake skin level cecostomy in a frail patient: a case report

#### F. Daverio ^1^, L. D'auria ^1^, I. Bitetti ^1^, P. Fugazzola ^2^, A.M. Mori ^1^, G. Ticozzelli ^1^, A. Locatelli ^1^

##### ^1^ Fondazione IRCCS Policlinico San Matteo, Pavia, Italy; ^2^ Fondazione IRCCS Policlinico San Matteo, Pavia, Italy

###### **Correspondence:** F. Daverio

*Journal of Anesthesia, Analgesia and Critical Care 2023,* **3(Suppl 1):**A43

Abstract

A 63-year-old woman (55 kg, 156 cm, BMI 22.6 kg/m2) was admitted to pneumology ward because of her worsening health conditions due to a known IV stage pleural mesothelioma; cancer had spread to all right lung (total opacity at chest X-rays) leading to a compressive effect on the mediastinal structures (superior vena cava, bronchi and pericardial infiltration) (Fig. 1), central nervous system (focal metastasis at left parietal cortex). Moreover, there was also a widespread peritoneal involvement conditioning a large bowel obstruction requiring an emergency skin level cecostomy. The patient had also a history of L4-S2 epidural abscess and L4-L5 osteomyelitis complicated by multiple right iliopsoas abscesses.

Patient-related anesthesiologic risk of mortality was estimated extremely high (ASA 4, POSSUM score 40.4% of predicted mortality, ACS NSQUIP risk score 20.3% of serious complications, 8.8% of death): considering the intrapulmonary shunt due to the tumoral infiltration, the ventilation management could have been very challenging and the hemodynamic instability generated by anesthetic agents, could have led to a dramatic reduction in pre-load needing a huge fluid administration and vasoactive drugs.

A neuraxial block was considered equally risky because of the infectious contraindication, the possible predicted technical difficulty due to the past spinal osteomyelitis and the hemodynamic effect of intrathecal local anesthetic.

Case reports of awake skin level cecostomy conducted with anesthetic blocks like QLB-1, Rectus sheath or TAP block are described and the authors reported good results in terms of pain management and comfort1,2.

In our case we deemed to perform a combined echo-guided monolateral right thoracic erector spinae plane block plus homolateral T8-T9 paravertebral block that, in our opinion, could assure better visceral and peritoneal analgesic coverage (Fig. 2).

A total of 30 ml of 0.33% Ropivacaine, 20 ml for ESP block and 5 ml for each paravertebral block was used to achieve the anesthesiologic plan, adding a light pharmacological intravenous sedation with Midazolam 1 mg and Remifentanil 300 mcg.

During the procedure no hemodynamic instability was observed, spontaneous breathing was maintained, and patient reported comfort without pain during all the surgical stimulation.

At the end of the surgery the patient has been transferred to a surgical ward and no postoperative complications have been reported.

Our experience, although limited to a single case of awake regional anesthesia skin level cecostomy, confirms the possibility of achieving an adequate anesthetic plan, even in frail patients who seem preferable not to be exposed to the systemic effects of general anesthesia.

Informed consent to publish had been obtained.

References

1. I. Vieira, C. Pereira, A. Silva et al., Quadratus Lumborum block as primary anesthetic technique for colostomy procedure: a case report, Brazilian Journal of Anesthesiology, https://doi.org/10.1016/j.bjane.2021.03.0144.
2. A.J. Hughe et al., Awake Colostomy Under Regional Anesthesia in Frail Patients, The American Surgeon 2022, Vol. 0(0) 1–3 DOI: 10.1177/00031348221129507

**Fig. 1 (abstract A43).**
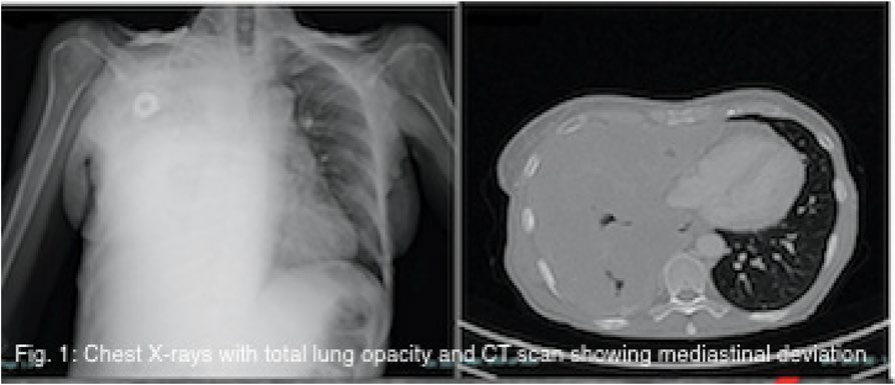
Chest X-rays with total lung opacity and CT scan showing mediastinal deviation

**Fig. 2 (abstract A43).**
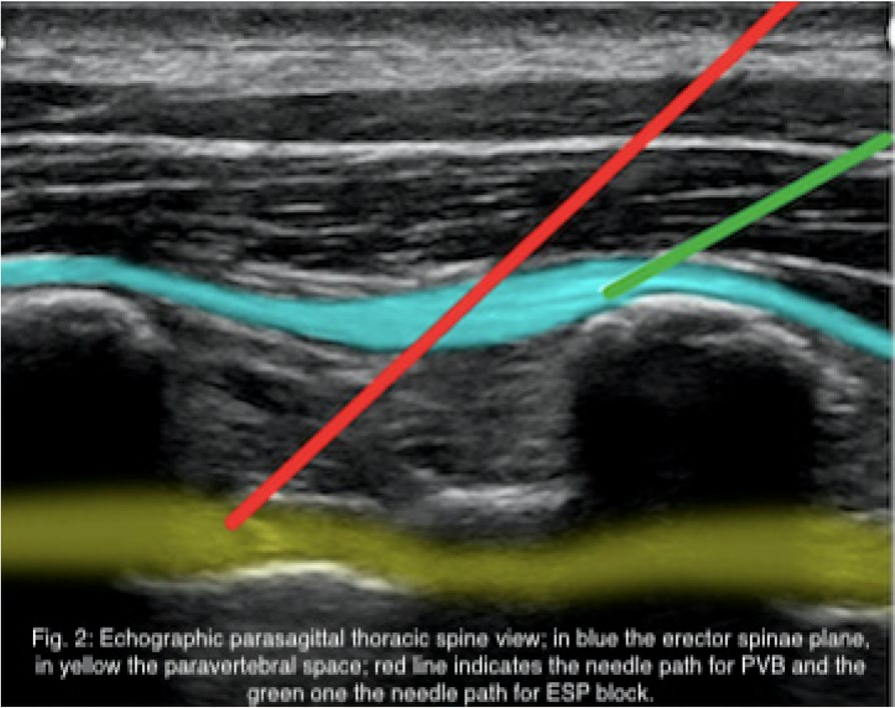
Echographic parasagittal thoracic spine view: in blue the erector spinae plane, in yellow the paravertebral space: red line indicates the needle path for PVB and the green one the needle path for ESP block

### A44 Trans-axillary TAVI in awake surgery: does BRILMA (Blocking the branches of intercostal nerves in the middle axillary line) represents an effective choise?

#### A.A. Petrone, V. Colella, A. Mastroianni, R. Buonomo, C. Marsicano, P. De Negri

##### AORN Sant'Anna e San Sebastiano, Caserta, Italy

###### **Correspondence:** A.A. Petrone

*Journal of Anesthesia, Analgesia and Critical Care 2023,* **3(Suppl 1):**A44

BACKGROUND

The Transcatheter Aortic Valve Implantation (TAVI) is a minimally invasive endovascular procedure aimed to replace a stenotic aortic valve by implanting an aortic bio prothesis inside of a sick native aortic valve.

This procedure is generally performed in local anaesthesia trought femoral artery. It’s an alternative to open heart surgery in patients older than 75 years old or in high-risk patients.

Transapical approach is an alternative to transfemoral technique. However trans-subclavian and trans axillary approach play an important role in patients with poor femoral vascular access.

Alternative approaches are usually performed under general anaesthesia.

We propose a case report of a patient undergoing transaxillary TAVI, treated with BRILMA.

CASE REPORT

A 71 years old man was admitted to ER for cardiogenic pulmonary edema and hospitalized in intensive care unit (ICU). The echocardiografic assessment esteblished a severe aortic stenosis. Past medical history: COPD, CAD (FE 30% - 80% RCA obstruction), hypertension.

The Heart Team decided for a transaxillary TAVI and PTCA on RCA.

We optated for a BRILMA due to high anaesthesiologic risk (ASA IV)

Previous mild sedation with dexmedetomidine (0,7 mcg/kg/m), the block was performed in plain with ultrasound technique, inserting the needle caudal-cranially over middle axillary line (IV-VI intercostal space) and placing the needle tip between the serratus muscle’s fascia and external intercostal muscle’s fascia. An anaesthetic mixture containing 25 mL of ropivacaine 0,5% plus 4 mg dexamethasone was used. The access point was infiltrated with an additional dose of 4 ml lidocaine 2%.

During the procedure the RASS was -2/-1 and no pain or adverse events recorded. At the end he was transferred to ICU for postoperative monitoring.

DISCUSSION

Chest wall blocks are usually used for breast cancer surgery. BRILMA, consists of the block of the cutaneous branches (lateral and anterior) from the II to the VI intercostal nerve, along the middle axillary line and which is commonly used for axillary lymphadenectomy.

It’s easy to perform, carried out under ultrasound guidance, since there are no superficial anatomical finds. It allows to observe real time the diffusion of the local anesthetic along the neurovascular plane, ensuring a lower rate of complications and a high success rate.

CONCLUSIONS

BRILMA can be successfully used in minimally invasive endovascular aortic valve surgery, when axillary or transucclavian approach is required, especially in patients at high anesthesiological risks. It represents a valid alternative to general anesthesia and paravertebral thoracic block.

Authors confirm that informed patient consent has been obtained.

### A45 Hypotension prediction index in elective Total Hip Arthroplasty undergoing neuroaxial anesthesia or peripheral nerve block: our preliminary experience

#### L. Pecora ^1^, S. Tallevi ^2^, V. Biscaccianti ^2^, L. Giovannelli ^3^, D. Tavoletti ^1^, E. Rosanò ^1^, E. Cerutti ^1^

##### ^1^ Anestesia e Rianimazione dei Trapianti e Chirurgia Maggiore, Azienda Ospedaliero Universitaria delle Marche, Ancona, Italy, ^2^ Dipartimento di Scienze Biomediche , Università Politecnica delle Marche, Ancona, Italy, ^3^ Anestesia e Rianimazione in Urgenza, Ospedale San Salvatore Centrale AST 1, Pesaro, Italy

###### **Correspondence:** S. Tallevi

*Journal of Anesthesia, Analgesia and Critical Care 2023,* **3(Suppl 1):**A45

Background

Intraoperative hypotension (IOH) is common during anesthesia and associated with postoperative adverse outcomes. The AcumenTM Hypotension Prediction Index (HPI) (Edwards Lifesciences; Irvine, CA, USA) is an innovative algorithm based on arterial waveform that predicts hypotension up to 15 minutes before it occurs(1). The aim of this study is to evaluate the haemodynamic pattern in patients undergoing neuroaxial anesthesia (NA) or peripheral nerve block (PNB) during elective total hip arthroplasty THA.

Materials and methods

Twelve patients, aged >18 years and consensual for the procedure, undergoing THA have been recruited at our centre. A radial arterial line was placed before anesthesia and connected to Hemosphere platform (Edwards Lifesciences). HPI monitoring started immediately. IOH was defined as a mean arterial pressure (MAP) < 65 mmHg for at least 1 minute. The decision to perform NA or the combination of Sciatic nerve block (SNB), Lumbar plexus block (LPB) and lateral Femoral Cutaneous nerve block (LFCNB) was based on the clinical assessment. A value of HPI of 85 and higher was defined as a trigger to initiate treatment with fluids or vasopressors according to hemodynamic parameters measured by Hemosphere.

The collected data analysis was performed using Acumen Analytics software, Edwards Lifesciences.

Results

The mean age of the patients was 80.2 ± 2.6 years. 7 patients had ASA III status. NA was performed in 5 patients and the combination of PNB in 7. Total number of the hypotension events in patients undergoing NA was 13 and the number of patients that experienced the hypotension was 2; one of them had severe hypotension (MAP under 50 mmHg). The average duration of each hypotensive event was 7.28 minutes. The time-weighted average (TWA) of AUT (area under a MAP of 65 mmHg) per patients was 0.69 mmHg. Hypotension events never occurred in patients undergoing combined PNB (Table 1).

Conclusions

In our experience, the hemodynamic management of patients candidate for THA is complex, because of old age and comorbidities (2). Our preliminary results show that patients undergoing combined PNB benefit of hemodynamic stability when compared with patients submitted to NA. This supports the hypothesis that this technique causes less perturbation due to the absence of sympatholysis and the consequential vasoplegia. Further clinical studies are needed to evaluate if the advantage of PNB in terms of hemodynamic stability is confirmed.

Informed consent to publish had been obtained

Reference

1. Hatib F, Jian Z, Buddi S, Lee C, Settels J, Sibert K, et al. Machine-learning Algorithm to Predict Hypotension Based on High-fidelity Arterial Pressure Waveform Analysis. Anesthesiology. 2018;129(4):663–74.
2. Gragasin FS, Bourque SL, Davidge ST. Vascular aging and hemodynamic stability in the intraoperative period. Vol. 3 APR, Frontiers in Physiology. 2012.

**Table 1(abstract A45).** Cohort comparison hypotension statistics

	NEUROAXIAL ANESTHESIA	PERIPHERAL NERVE BLOCK
**Number of patients**	5	7
**Number of patients with hypotension**	2 of 5 40%	0
**Total number of hypotensive events in dataset**	13 events	0
**Average duration of each hypotensive events**	7.28 ± 6.17 minutes	0
**TWA of AUT (MAP<65 mmHg) per patient**	0.69 ±1.35 mmHg	0
**% of patients who experience an event under 50 mmHg**	1 of 5 20%	
**Vasoactive drugs (number of treated patients)**	2	0

### A46 Regional anesthesia in two patients with congenital factor VII deficiency

#### V. Biscaccianti ^1^, S. Tallevi ^1^, D. Tavoletti ^2^, E. Rosanò ^2^, P. Cerchiara ^2^, L. Pecora ^2^, E. Cerutti ^2^

##### ^1^ Dipartimento di Scienze Biomediche, Università Politecnica delle Marche, Ancona, Italy; ^2^ Anestesia e Rianimazione dei Trapianti e Chirurgia Maggiore, Azienda Ospedaliero Universitaria delle Marche, Ancona, Italy

###### **Correspondence:** V. Biscaccianti

*Journal of Anesthesia, Analgesia and Critical Care 2023,* **3(Suppl 1):**A46

Background

Congenital factor VII (FVII) deficiency is a rare haemorrhagic disorder that causes isolated and prolonged prothrombin time (PT) in the presence of a normal partial thromboplastin time (PTT) [1].

Evidence on the safety of peripheral nerve block (PNB) in patients with coagulopathy is lacking.

We describe the anesthetic management of two high risk patients with FVII deficiency undergoing orthopaedic surgery.

Case report

A 71 year-old female patient, (65 kg, 159 cm, ASA III) with chronic obstructive pulmonary disease, diabetes mellitus, chronic renal failure and a 68 year-old female patient (71 kg, 164 cm, ASA III) with dilated cardiomyopathy, left ventricular systolic dysfunction, and diabetes mellitus were admitted for femoral fracture and humerus fracture respectively. They had no history of pathologic bleeding due to FVII deficiency.

Coagulation studies showed a low FVII activity and a decreased prothrombin activity (PT) with a normal activated partial thromboplastin (APTT).

A first dose of 15 mcg/kg of recombinant form activated FVII (rFVIIa) was given 30 minutes prior to anesthesia.

Anesthesia plan for total hip replacement included lumbar plexus, sciatic, lateral femoral cutaneous and lateral branch of the iliohypogastric nerve blocks, while open reduction and internal fixation of the humerus fracture was managed with ultrasound-guided interscalene and infraclavicular brachial plexus blocks.

No bleeding complications were seen during the surgical procedures and no blood transfusion was required for both.

A second and third dose of rFVIIa were administered 6 and 12 hours after surgery

Coagulation tests were performed every day. Bleeding through the drainage tubes were minimal.

The subsequent course was uneventful, and the patients were discharged on the seventh and fifth

day after surgery respectively.

Conclusion

PNB could be a safe technique in FVII-deficient patients, nevertheless substitution therapy is necessary for anesthestic and surgical procedures to prevent or to manage bleeding episodes.

Written informed consent to publish had been obtained

References

1. E. R. Strauss, M. A. Mazzeffi, B. Williams, N. S. Key, K. A. Tanaka. Perioperative management of rare coagulation factor deficiency states in cardiac surgery. Br. J. Anaesth. 2017; 119:354–68.

**Table 1 (abstract A46).** Haemoglobin and haemostasis parameters before and after surgery

	Before surgery		6 h after surgery		12 h after surgery		Day 1		Day 2		Day 3		Day 4	
	Patient 1	Patient 2	Patient 1	Patient 2	Patient 1	Patient 2	Patient 1	Patient 2	Patient 1	Patient 2	Patient 1	Patient 2	Patient 1	Patient 2
Hb	11,8	12,1	10,8	10,7	10,1	10,2	10,2	10	10,4	10,1	10,2	10,1	10,2	10,3
Plt	181	157	162	154	159	160	161	166	163	160	155	171	153	169
FVII	23	27	-	-	-	-	-	-	-	-	-	-	-	-
PT	49	54	71	68	72	77	60	80	46	60	47	56	48	45
APTT	30	31	31	31	33	32	35	32	32	33	31	34	30	33

*Hb* hemoglobin (gr dl^-1^), *Plt* Platelets (10^9^ L^-1^), *FVII* factor VII (%), *PT* prothrombin time (%), *APTT* activated partial thromboplastin time (s)

### A47 A Modified Approach for Ultrasound-Guided Thoracic Paravertebral Block via Thoracic Intervertebral Foramen: preliminary data from a prospective cadaveric study

#### E. Petrucci ^1^, B. Pizzi ^2^, C. Bianchi ^3^, G. Marrocco ^3^, F. Sciorio ^3^, G. Ceccaroni ^3^, L. Sollima ^4^, D. Rullo ^4^, G. Calvisi ^4^, M. Cascella ^5^, A. Vittori ^6^, F. Marinangeli ^3^

##### ^1^ Department of Anesthesia and Intensive Care Unit, San Salvatore Academic Hospital of L Aquila, L Aquila, Italy; ^2^ Department of Anesthesia and Intensive Care Unit, SS Filippo and Nicola Academic Hospital of Avezzano, L Aquila, Italy; ^3^ Department of Life, Health and Environmental Sciences, Luca Tonini Simulation Center, Department of Life, UnivAQ, L Aquila, Italy; ^4^ Department of Anatomopathology, San Salvatore Academic Hospital of L Aquila, L Aquila, Italy; ^5^ Department of Anesthesia and Critical Care, Istituto Nazionale Tumori, IRCCS, Fondazione Pascale, Napoli, Italy; ^6^ Critical Care, ARCO Roma, Ospedale Pediatrico Bambino Gesù IRCCS, Roma, Italy

###### **Correspondence:** C. Bianchi

*Journal of Anesthesia, Analgesia and Critical Care 2023,* **3(Suppl 1):**A47

Background

We hypothesized an alternative way to perform thoracic paravertebral block. We called this technique thoracic intervertebral foramen (TIF) block. We aimed to verify the spread of dye onto the nervous structures of retropleural space, and into the thoracic paravertebral space (TPVS) and epidural space (ES).

Methods

This is a prospective cadaveric study. Before performing the autopsy, the anesthetic procedure was bilaterally performed at sixth (T6) and at tenth thoracic (T10) vertebra, by using a highfrequency linear-array ultrasound (US) transducer. A Tuohy needle was inserted in-plane to the ultrasound beam in a lateral-to-medial direction gently to contact the spinous process (SP). Subsequently, the needle tip was advanced for 2 mm along with the superior limit of the vertebral pedicle, until losing contact with the bone with 5 ml methylene blue 1% dye (MB) injection. Following, 2 continuous catheter sets were inserted. At the end of the anesthetic procedure, a second US scan of thoracic paravertebral space was performed. A cadaveric dissection was then performed.

Results

We report data from a total of 8 TIF blocks performed in 2 cadavers. In 6 injection sites, we

found dye on both sides of the TPVS and ES, from T4 to T8 and from T9 to T12 levels. In 2 cases, MB was accumulated intramuscularly at T10 level. The ventral rami, the communicating rami, and the

sympathetic trunk (from T4 to T7 and from T8 to T12) were stained by dye in 6 cases. Five intervertebral foramens (IVFs) were filled by dye. The second look US scan documented that in 6 cases, anechoic fluid was found in the TPVS ranging from T4 to T7 and from T8 to T12 levels.

Conclusions

The TIF block achieved a consistent dye spread into the TPVS and ES capturing retropleural organs.

Consent to publish.

Informed consent to publish had been obtained.

### A48 Ultrasound-guided quadratus lumborum block in abdominal robotic surgery: a prospective observational study

#### F. Baccoli ^1^, G. Abbiati ^1^, V. Serafini ^2^, B. Brunoni ^1^, V. Torrano ^2^, R. Fumagalli ^2^

##### ^1^ Department of Medicine and Surgery, University of Milan-Bicocca, Monza, Italy; ^2^ Department of Emergency and Urgency, Anesthesia and Intensive care Unit 1, ASST Grande Ospedale Niguarda, Milan, Italy

###### **Correspondence:** F. Baccoli

*Journal of Anesthesia, Analgesia and Critical Care 2023,* **3(Suppl 1):**A48

Background

Robotic surgery is increasing in various specialties, however, there is still limited knowledge regarding perioperative pain management. Enhanced Recovery After Surgery (ERAS) protocols suggest that quadratus lumborum block (QLB) may be a feasible option for abdominal procedures, although evidence supporting the use of fascial blocks is of low-moderate quality [1]. While Lai et al. has suggested a potential benefit of QLB in robotic-assisted partial nephrectomy (RAPN)[2], conflicting data have emerged from other fields[1,3]. Neuraxial approaches are associated with potential adverse events and have fallen out of recommendations in laparoscopic colorectal surgery since 2012 due to the limited pain caused by less invasive techniques [4].

Therefore, our study aims to evaluate the efficacy of QLB type 3 in providing pain relief, enhancing early recovery, and promoting mobilization.

Materials and methods

We enrolled 11 patients scheduled for abdominal robotic surgery under general anesthesia (two excluded for intraoperative complications). Ultrasound-guided QLB 3 (transmuscular approach) was performed before surgery with ropivacaine 0.375% (Image 1).

Multimodal therapy was administered, consisting of paracetamol and non-steroidal anti-inflammatory drugs (NSAIDs). Pain levels were assessed using the Numerical Rating Scale (NRS) at 2, 6, 12, and 24 hours postoperatively. At 24h, we request patients to complete Quality of Recovery (QoR-15) questionnaire.

Results

Clinic and demographic data are shown in (Table 1).

QLB block was performed before surgery, with ropivacaine 0.375%. NRS scores at rest and during movement are presented in (Table 2).

One patient required 2 mg of morphine and one (with a history of NSAIDs allergy and chronic kidney failure) 100mg of tramadol as rescue therapy. The mean QoR score was 110.89±14.38. Nobody reported nausea following the surgical procedure. Comparing to a group of 22 patients (using chi-square test) receiving intrathecal morphine we found statistically significant difference in terms of nausea (p<0.01), pruritus (p<0.001) and post-operative morphine consumption (p<0.03).

Conclusions

QLB 3 appears to be associated with good pain relief at rest and movement within the first 24 hours post-operatively and may be a useful addition to the multimodal analgesic regimen for a variety of surgeries. We also observed a limited opioid consumption and clinical benefit for nausea and pruritus in comparison to intrathecal analgesia. Data from QoR-15 questionnaire suggest that QLB may be an effective tool to enhance early mobilization, despite the common practice in the department does not allow implementation of these programs.

Our results are encouraging, further research is necessary to confirm preliminary data.

References

1. Ardon A, et al. The Use of Peripheral Nerve Blockade in Laparoscopic and Robotic Surgery: Is There a Benefit? Curr Pain Headache Rep. 2022
2. Lai R, et al. Ultrasound-guided quadratus lumborum block for perioperative analgesia in robot-assisted partial nephrectomy: a randomized controlled trial. Trials. 2021
3. Khater N, et al. Current Strategies in Pain Regimens for Robotic Urologic Surgery: A Comprehensive Review. Anesth Pain Med. 2022
4. Joshi GP, et al. Evidence-based postoperative pain management after laparoscopic colorectal surgery. Colorectal Dis. 2013

**Image 1 (abstract A48).**
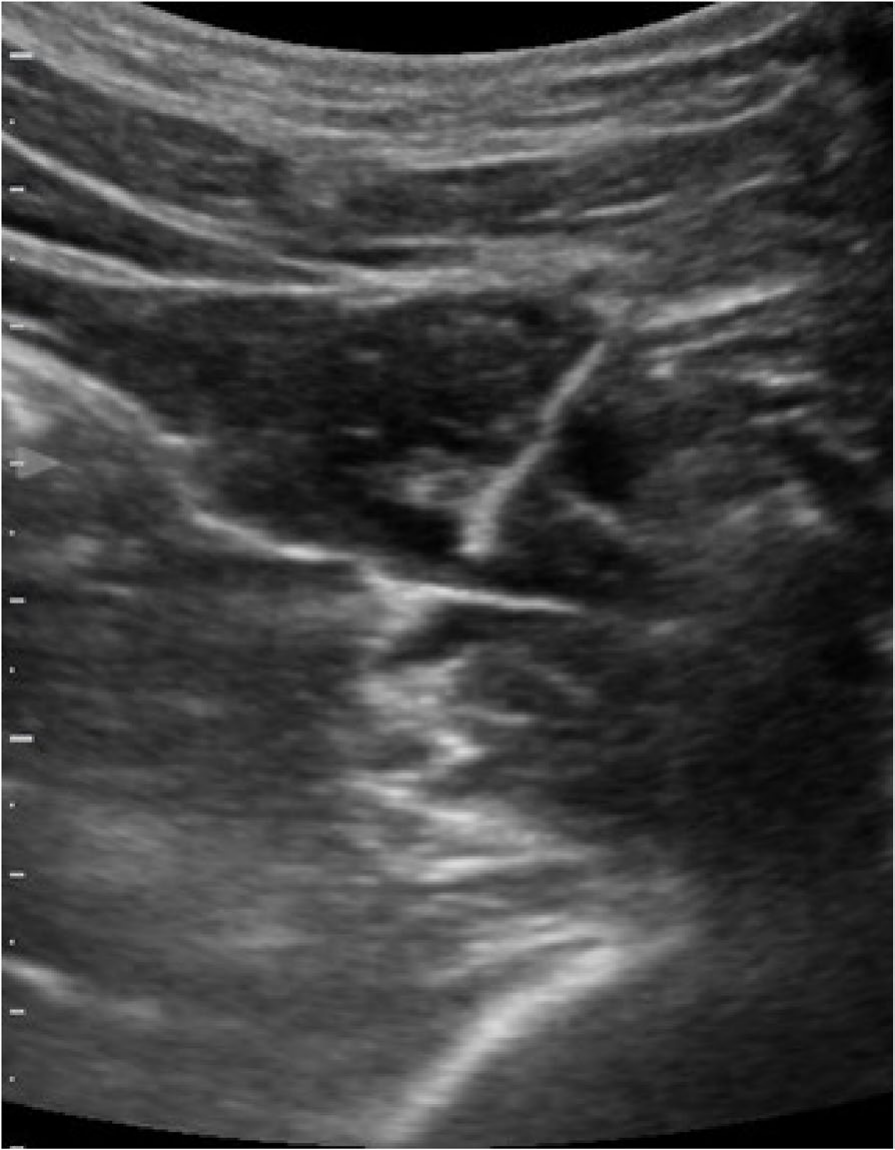
See text for description

**Table 1 (abstract A48).** See text for description

Patients (N 9)	Mean±SD
Age	57.44±14.93
Sex	
Male	6 (66.7%)
Female	3 (33.3%)
BMI (Kg/m^2^)	23.97±3.13
ASA	
I	1 (11.1%)
II	7 (77.78%)
III	1 (11.1%)
Type of surgery	
Renal tumorectomy	2 (22.2%)
Pyeloplasty	2 (22.2%)
Robotic-assisted laparoscopic prostatectomy	3 (33.3%)
Nefrectomy	1 (11.1%)
Right hemicolectomy	1 (11.1%)
Surgical duration (mins)	246.67±39.72

**Table 2 (abstract A48).** See text for description

**Measurement time**	**Mean±SD**
Rest	
2h	2.56±2.41
6h	2.33±1.7
12h	2.89±1.97
24h	2±1.33
Movement	
2h	3.89±2.51
6h	3.33±1.15
12h	3.44±1.89
24h	2.78±1.03

### A49 Transversus Abdominis plane block vs intrathecal analgesia for postoperative pain management in robotic urologic surgery: A Retrospective pilot study

#### S. Gianni^1^, F. Mulazzani ^1^, P. Dell'Oglio ^2^, E. Roselli ^1^, A. Galfano ^2^, R. Fumagalli ^3^, G. Monti ^1^

##### ^1^ Anestesia e rianimazione dei trapianti, ASST Grande Ospedale Metropolitano Niguarda, Milano, Italy; ^2^ Department of urology, ASST Grande Ospedale Metropolitano Niguarda, Milano, Italy; ^3^ Anestesia e rianimazione 1, ASST Grande Ospedale Metropolitano Niguarda, Milano, Italy

###### **Correspondence:** S. Gianni

*Journal of Anesthesia, Analgesia and Critical Care 2023,* **3(Suppl 1):**A49

Background

Robotic-assisted laparoscopic surgery revolutionized the surgical management of localized kidney and prostate cancer, offering advantages over traditional open surgery. Robotic surgery offers less discomfort to patients because of smaller keyhole incisions and less tissue retraction and stretching of fascia and muscular fibers. However, optimal postoperative pain management remains crucia [1]. Analgesia techniques such as transversus abdominis plane (TAP) block [2] and intrathecal administration of morphine [3] have shown promise but lack direct comparison in this surgical context.[4] We performed a retrospective pilot trial comparing TAP block and intrathecal analgesia on postoperative pain control after urologic surgery.

Materials and methods

We conducted a retrospective study at Niguarda Hospital in Milan from September 2022 to January 2023, including patients scheduled for major urologic robotic surgery receiving intrathecal analgesia (IA) with morphine or TAP block. Patient demographics, surgical risk assessment (ASA score), and baseline characteristics were recorded. Data on analgesia techniques, rescue analgesia, pain scores, postoperative nausea and vomiting (PONV), and length of hospital stay (LOS) were collected prospectively. Data are reported as median (interquartile range [IQR]) for continuous variables and as frequencies and proportions for categorical variables.

All the analyses were conducted using R Core Team (version 4.3.0).

Results

We analyzed 59 consecutive patients 44 receiving IA and 15 receiving TAP block as regional analgesia. Population baseline characteristics are summarized in Table 1.

Eight patients underwent robotic nephrectomy, eight partial nephrectomy and 43 radical prostatectomy. We found no significative difference between Numeric pain Rating Scale (NRS) at operative room discharge (p=0.12), and during the first (p=0.63) and second postoperative days (p=0.5) (Figure 1).

Also the need of rescue analgesic therapy during the first (p=1) and second (p=0.32) postoperative day. None of the patients receiving TAP block experienced PONV during the first and second postoperative days while, in the IA group, eight and three patients experienced PONV during postoperative day 1 and 2 respectively. Hospital length of stay did not differ between the two groups.

Conclusion

This pilot observational study suggests that both TAP block and IA effectively manage postoperative pain after major robot-assisted urologic surgery. TAP block emerges as a valid alternative to IA due to the comparable effect on postoperative pain without IA related side effects such as PONV, urinary retention, pruritus and respiratory depression. Larger studies are needed to confirm these findings and further optimize pain management in this surgical population.

References

1. Khater N, Comardelle NJ, Domingue NM, Borroto WJ, Cornett EM, Imani F, et al. Current Strategies in Pain Regimens for Robotic Urologic Surgery: A Comprehensive Review. Anesth Pain Med 2022;12. https://doi.org/10.5812/aapm-127911.
2. Taninishi H, Matsusaki T, Morimatsu H. Transversus Abdominis Plane Block Reduced Early Postoperative Pain after Robot-assisted Prostatectomy: a Randomized Controlled Trial. Sci Rep 2020; 10:3761. https://doi.org/10.1038/s41598-020-60687-y.
3. Shim JW, Cho YJ, Moon HW, Park J, Lee HM, Kim YS, et al. Analgesic efficacy of intrathecal morphine and bupivacaine during the early postoperative period in patients who underwent robotic-assisted laparoscopic prostatectomy: a prospective randomized controlled study. BMC Urol 2021;21. https://doi.org/10.1186/s12894-021-00798-4.
4. Milliken D, Lawrence H, Brown M, Cahill D, Newhall D, Barker D, et al. Anaesthetic management for robotic-assisted laparoscopic prostatectomy: the first UK national survey of current practice. J Robot Surg 2021;15:335-41. https://doi.org/10.1007/s11701-020-01105-3.

**Table 1 (abstract A49).** Descriptive statistics of study population

	Total	Intrathecal	TAP block	p value
	(N=59)	(N=44)	(N=15)	
**Age (years)**				
**Median [IQR]**	65.5 [14.5]	65.0 [13.0]	67.0 [11.5]	
**Missing**	1 (1.7%)	1 (2.3%)	0 (0%)	
**Gender**				
**Femminile**	6 (10 %)	2 (5 %)	4 (27 %)	
**Maschile**	53 (90 %)	42 (95 %)	11 (73 %)	
**BMI (kg/m2)**				
**Median [IQR]**	25.6 [4.70]	25.7 [3.47]	24.6 [5.97]	
**ASA score**				
**Median [IQR]**	2.00 [0]	2.00 [0.500]	2.00 [0]	
**Missing**	1 (1.7%)	1 (2.3%)	0 (0%)	
**Type of surgery**				
**Nephrectomy**	8 (14 %)	3 (7 %)	5 (33 %)	
**Partial nephrectomy**	8 (14 %)	5 (11 %)	3 (20 %)	
**Prostatectomy**	43 (73 %)	36 (82 %)	7 (47 %)	
**Cardiovascular disease**	27 (46%)	17 (39%)	10 (71%)	
**Obstructive Pulmonary disease/Smoke**	9 (16%)	7 (16%)	2 (13%	
**Diabetes/Endocrinologic disorder**	5 (8%)	5 (11%)	0 (0 %)	
**Chronic kidney disease**	5 (8%)	2 (5%)	3 (21%)	
**Neurologic disorder**	1 (2%)	0 (0 %)	1 (7%)	
**Coagulopathy/Anticoagulant therapy**	0 (0%)	0 (0 %)	0 (0 %)	
**Postoperative acetaminophen**				
**No**	1 (2 %)	1 (2 %)	0 (0 %)	
**Yes**	58 (98 %)	43 (98 %)	15 (100 %)	
**Postoperative ketorolac**				
**No**	43 (73 %)	38 (86 %)	5 (33 %)	
**Yes**	16 (27 %)	6 (14 %)	10 (67 %)	
**Postoperative tramadol**				
**No**	58 (98 %)	44 (100 %)	14 (93 %)	
**Yes**	1 (2 %)	0 (0 %)	1 (7 %)	
**NRS OR discharge**				
**Median [IQR]**	0 [0]	0 [0]	0 [0]	*0.12*
**Missing**	25 (42.4%)	19 (43.2%)	6 (40.0%)	
**NRS day 1**				
**Median [IQR]**	0 [1.00]	0 [1.00]	0 [2.00]	*0.63*
**Missing**	6 (10.2%)	4 (9.1%)	2 (13.3%)	
**NRS day 2**				
**Median [IQR]**	0 [0]	0 [0]	0 [0]	*0.51*
**Missing**	10 (16.9%)	10 (22.7%)	0 (0%)	
**Need for rescue analgesia day 1**				
**No**	50 (85 %)	37 (84 %)	13 (87 %)	
**Yes**	9 (15 %)	7 (16 %)	2 (13 %)	*1.00*
**Need for rescue analgesia day 2**				
**No**	53 (90 %)	38 (86 %)	15 (100 %)	
**Yes**	6 (10 %)	6 (14 %)	0 (0 %)	*0.32*
**PONV day 1**				
**No**	51 (86 %)	36 (82 %)	15 (100 %)	
**Yes**	8 (14 %)	8 (18 %)	0 (0 %)	*0.1*
**PONV day 2**				
**No**	56 (95 %)	41 (93 %)	15 (100 %)	
**Yes**	3 (5 %)	3 (7 %)	0 (0 %)	*0.56*
**Hospital LOS**				
**Median [IQR]**	3.00 [1.00]	3.00 [1.00]	3.00 [0]	

**Fig. 1 (abstract A49).**
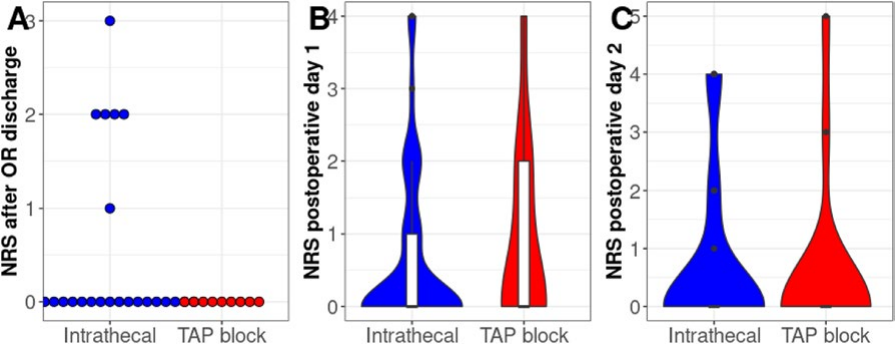
Numeric pain Rating Scale (NRS) at operative room (OR) discharge (Panel A), NRS on postoperative day 1 (Panel B), NRS on postoperative day 2 (Panel C)

## Anesthesia in the fragile patient

### **A50 Tako-Tsubo Syndrome in a Patient with Post-Operative Septic Shock**

#### G. Cosenza, S. de Sarno, A. di Giovanni, F. Coppolino, V. Pota, M.B. Passavanti, P. Sansone, C. Pace

##### Università della Campania Luigi Vanvitelli, Napoli, Italy

###### **Correspondence:** G. Cosenza

*Journal of Anesthesia, Analgesia and Critical Care 2023,* **3(Suppl 1):**A50

Background

Septic shock is characterized by an abnormal host response to infection, resulting in multi organ failure and hemodynamic instability. Guidelines recommend fluid resuscitation with 30 mL/kg of crystalloids within the first three hours of diagnosis and administration of norepinephrine to achieve a MAP > 65 mmHg.

Distributive shock is not the only hemodynamic alteration which can occur in septic patients. The catecholamine stress that characterizes the early stages of the disease is an important trigger for the onset of transient and reversible dysfunction of the left ventricle (LV) with symptoms like myocardial infarction, a condition known as Tako-Tsubo Syndrome (TTS).

TTS is a stress-induced (physical or emotional) cardiomyopathy so called for the characteristic ballooning shape that the left ventricular apex presents on echocardiography.

The diagnostic criteria for TTS according to the 'International Expert Consensus Document on TTS' are:

A) Transient dysfunction of the LV with ballooning of the apex and dyskinesia of the myocardial walls not attributable to the perfusion territory of a particular coronary artery
B) Onset of aspecific electrocardiogram abnormalities
C) Rise in cardiac biomarkers
D) Absence of myocardial infection

Case Report

After obtaining informed consent, a 56-year-old man with dehiscence of the ileocolic anastomosis post-resection surgery was admitted to our intensive unit care. Septic shock was diagnosed and fluid resuscitation and continuous infusion of norepinephrine at a dosage of 0.2 mcg/kg/min were administered. The patient, connected to mechanical ventilation in IPPV mode, was weaned off at 6 hours after admission. Hemodynamic monitoring was established using Acumen HPI software. Norepinephrine therapy was continued, achieving a MAP of 65 mmHg.

On the second day, there was an elevation of cardiac biomarkers and T wave inversion in all leads on the ECG.

Transthoracic echocardiogram (TTE) showed an ejecton fraction (EF) of 30%, halved compared to that found on the pre-operative TTE, meeting the diagnostic criteria for TTS.

In accordance with guidelines, non-catecholamine inotropic infusion was started with levosimendan (0.1 mcg/kg/min) and short-acting beta-blocker infusion with esmolol (60 mcg/kg/min) to reduce catecholamine-mediated myocardial insult.

Conclusion

In our case report, due to peri-operative management, artificial intelligence-based monitoring, and post-operative pharmacological management according to guidelines, we observed an improvement in EF, a reduced need for vasopressors, and an increase in cardiac output until discharge to the referring department.

Informed consent to publish had been obtained

**Fig. 1 (abstract A50).**
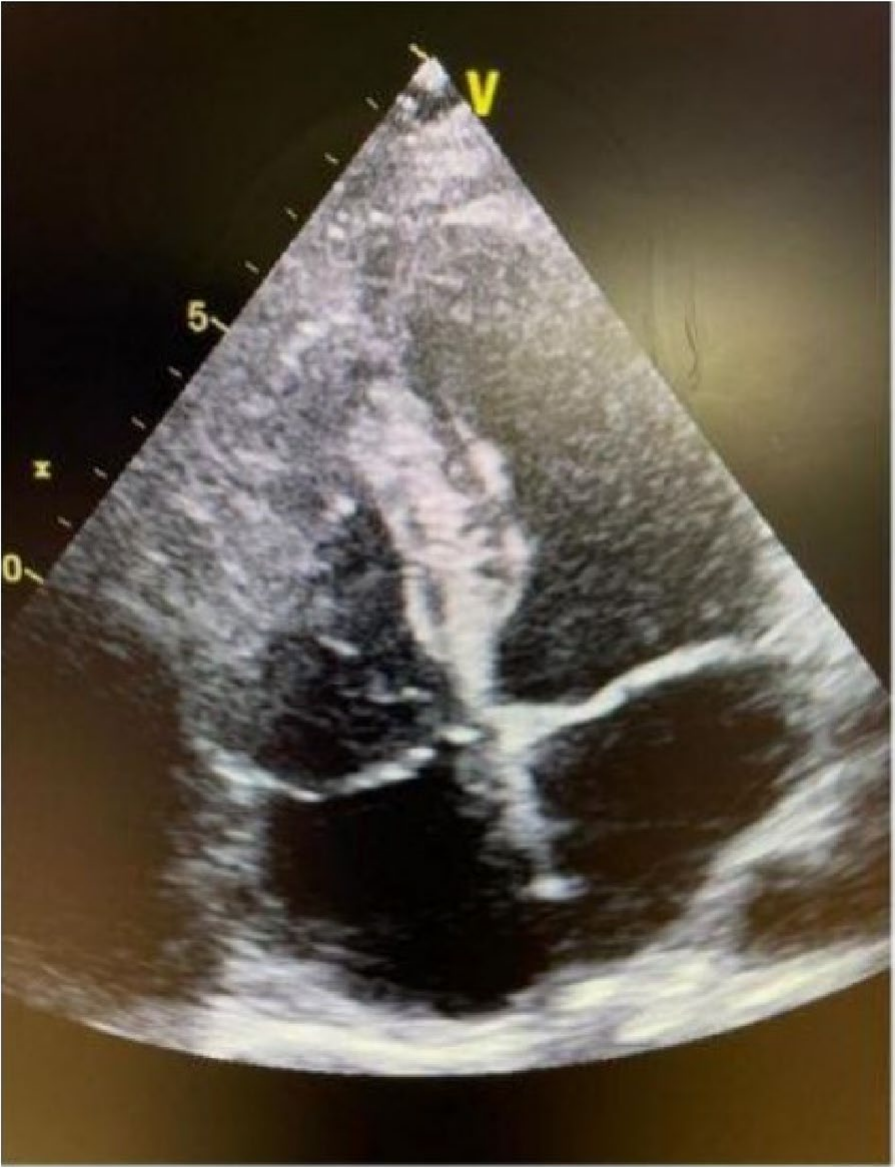
Pre-operative TTE

**Fig. 2 (abstract A50).**
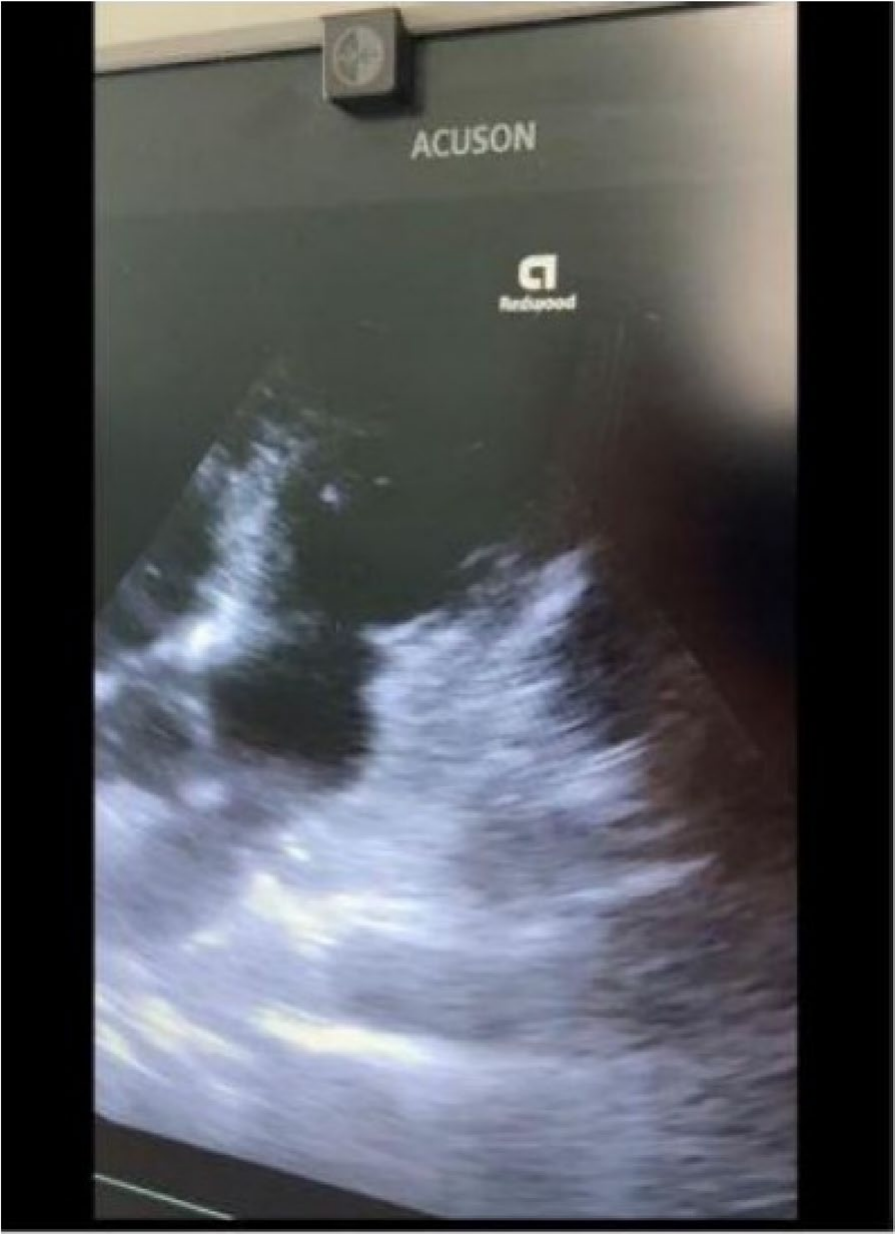
Intensive care TTE

### A51 Postoperative muscle mass depletion in elderly frail patients undergoing major elective surgery: a retrospective analysis of outcome on a prospectively included cohort

#### F. Moretto ^1^, A. Siani ^1^, M. Fracazzini ^1^, A.C. Leonetti ^1^, N. De Vita ^1,2^, L. Scotti ^1^, V. Viarengo ^2^, S. Gentilli ^3^, R. Romito ^4^, A. Volpe ^5^, M. Leigheb ^6^, C. Rigamonti ^7^, A. Carriero ^8^, F. Della Corte ^1,2^, R. Vaschetto ^1,2^

##### ^1^ Università del Piemonte Orientale, Dipartimento di Medicina Traslazionale, Novara, Italy; ^2^ Azienda Ospedaliero Universitaria Maggiore della Carità, Anestesia e Terapia Intensiva, Novara, Italy; ^3^ Azienda Ospedaliero Universitaria Maggiore della Carità, Clinica Chirurgica, Novara, Italy; ^4^ Azienda Ospedaliero Universitaria Maggiore della Carità, Chirurgia Generale 2, Novara, Italy; ^5^ Azienda Ospedaliero Universitaria Maggiore della Carità, Urologia, Novara, Italy; ^6^ Azienda Ospedaliero Universitaria Maggiore della Carità, Ortopedia e Traumatologia, Novara, Italy; ^7^ Azienda Ospedaliero Universitaria Maggiore della Carità, Medicina Interna, Novara, Italy; ^8^ Azienda Ospedaliero Universitaria Maggiore della Carità, Radiologia diagnostica e interventistica, Novara, Italy

###### **Correspondence:** F. Moretto

*Journal of Anesthesia, Analgesia and Critical Care 2023,* **3(Suppl 1):**A51

Background

Elderly frail patients undergoing surgery are increasing nowadays. Sarcopenia is an objective measurement of frailty and has been identified as a predictor of poor postoperative outcomes. Study objectives are to evaluate the impact of sarcopenia on postoperative outcomes in elderly frail patients undergoing major elective surgery and factors affecting postoperative depletion of skeletal muscle mass in the same patients.

Materials and methods

This retrospective analysis was conducted on prospectively included patients 70 years older or more, undergoing major elective surgery in general surgery, urology, orthopedics at Maggiore della Carità Hospital, Novara, for whom preoperative and postoperative computer tomography (CT) scans were available. Total psoas area (TPA) measured at third lumbar vertebra level was adjusted for height to calculate total psoas index (TPI = TPA/height^2^ [cm^2^/m^2^]). Sarcopenia was defined as the lowest sex-specific quartile of TPI. Furthermore, preoperative and postoperative muscle density as Hounsfield Units (HU) was calculated.

Results

Among the 104 patients included from May 2020 to January 2022, 51 patients with preoperative and postoperative CT scans were retrospectively analyzed. Mean age was of 76 (±4) years and 27 (53%) were males. Eighteen patients underwent urologic surgery, 13 upper digestive and 20 lower digestive surgery, with laparoscopic or robotic surgery performed in 31 cases. Surgery was conducted under general balanced anesthesia in all cases and mean anesthesia duration was of 322 (±117) minutes. Thirteen patients were defined as sarcopenic at the preoperative CT scan analysis and mean preoperative TPI was 4.27 (±0.93) cm^2^/m^2^ in males and 2.96 (±0.85) cm^2^/m^2^ in females. Baseline muscle density was 55 (±8) HU in males and 50 (±7) HU in females. Postoperative CT was performed in median 8 (5-15) months after surgery, showing an overall decrease of 14% in TPI (absolute decrease of 0.51 (±0.08) cm^2^/m^2^) and of 13% in muscle density. TPI reduction was more relevant in males than females, 0.62 (±0.03) cm^2^/m^2^ (14%) vs 0.38 (±0.06) cm^2^/m^2^ (12%) respectively. Median hospital length of stay (HLOS) was of 8 (6-13) days, eight patients were admitted to intensive care unit (ICU) after surgery, and 22 experienced some postoperative complications. At univariable regression analysis, preoperative sarcopenia did not influence HLOS, ICU admission and postoperative complications occurrence, while HLOS and ICU admission were significantly associated with postoperative TPI decrease.

Conclusions

Sarcopenia did not affect postoperative outcomes in our cohort, but patients with muscle depletion had longer hospitalization and more ICU admissions.

### A52 The impact of CONOX® on postoperative cognitive dysfunction in elective surgical elderly patient: a prospective observational study

#### A.U. de Siena ^5^, S. Nappi ^4^, C. Visani ^3^, S. Mele ^2^, M. Vargas ^1^, A. Marra ^1^

##### ^1^ Department of Neurosciences, Reproductive and Odontostomatological Sciences, University of Naples, Federico II, Napoli, Italy; ^2^ Department of Neurosciences, Reproductive and Odontostomatological Sciences, University of Naples, Federico II, Napoli, Italy; ^3^ Department of Neurosciences, Reproductive and Odontostomatological Sciences, University of Naples, Federico II, Napoli, Italy; ^4^ Department of Neurosciences, Reproductive and Odontostomatological Sciences, University of Naples, Federico II, Napoli, Italy; ^5^ Department of Neurosciences, Reproductive and Odontostomatological Sciences, University of Naples, Federico II, Napoli, Italy

###### **Correspondence:** A.U. de Siena

*Journal of Anesthesia, Analgesia and Critical Care 2023,* **3(Suppl 1):**A52

Background: Postoperative cognitive dysfunction (POCD) occurs in 40% of elderly patients, during post-operative recovery for elective surgery; POCD can be assessed with Montreal Cognitive Assessment score (MoCA) [1,2]. 36-Item Short Form Health Survey (SF-36) and Quality of Recovery (QoR) are two questionnaires to assess the healthy status and recovery quality, respectively.

Methods: This study was a prospective observational trial. We included all over 60 years old patients, hospitalized for at least 3 days, undergoing general anaesthesia combined with spinal anesthesia for elective surgical procedure lasting at least 2 hours. Exclusion criteria were: history of mental or central nervous disorders, severe hypoacusis or visual loss, assumption of psychotropic drugs, impossibility to obtain written consent. Informed consent was obtained from all partecipants in the study. CONOX® were applied on each patient before the induction of anesthesia. Patients were divided in two groups: CONOX® guided group (C-group), in which anesthesia was guided by CONOX®, or routine care group. MoCA and SF-36 were collected: the day before and the day after the surgery; 7 days and 30 days via telephone after surgery; QoR was collected only the day after surgery. POCD is present if the MoCA score of postoperative periods is 1 or more standard deviations less than the baseline [1]. T-student test was used to analyses the difference between the continuous data and a chi-square test was used to analyse the binomial distributions. The statistical significance was set at a p-value<0.05.

Results: We enrolled 13 patients: 7 in C-guided group and 6 in routine care group. Two patients of C-group dropped out at the follow-up and they were excluded from the analysis. No differences were found in the baseline characteristics (Table 1).

No significant statistical difference was found in all scores at day 1, day 7 and day 30 between the two groups (Table 2).

The intraoperative parameter (i.e. heart rate, halogenated consumption, etc.) did not differ between groups (Table 3).

No differences were found in POCD incidence between the groups (Table 4).

Table 4 - Value of standard deviations from baseline for each patient and comparison of incidence of POCD between groups

Conclusions: Conversely to data reported in literature in our population we found no difference in the 2 groups, probably due to a too small sample size. Our data reported an incidence of about 50% of POCD, with no POCD at 30 days in the C-guided group. However, the application of anaesthesia monitoring, such as bispectral index (BIS), reduces the incidence of POCD [3]. More trials are needed to clarify if also CONOX® can reduce the incidence of POCD.

References

1. Dautzenberg G, Lijmer J, Beekman A. Diagnostic accuracy of the Montreal Cognitive Assessment (MoCA) for cognitive screening in old age psychiatry: Determining cutoff scores in clinical practice. Avoiding spectrum bias caused by healthy controls. Int J Geriatr Psychiatry. 2020 Mar;35(3):261–9.
2. Urits I, Orhurhu V, Jones M, Hoyt D, Seats A, Viswanath O. Current Perspectives on Postoperative Cognitive Dysfunction in the Ageing Population. Turk J Anaesthesiol Reanim. 2019 Dec 11;47(6):439–47.
3. Chan MTV, Cheng BCP, Lee TMC, Gin T. BIS-guided Anesthesia Decreases Postoperative Delirium and Cognitive Decline. J Neurosurg Anesthesiol. 2013 Jan;25(1):33–42.

**Table 1 (abstract A52).** Characteristics of the enrolled patients

N=11 (100%)	C-group	Routine care group	Mean difference	95% CI	t	dF error	p-value
	N=5 (46%)	N=6 (54%)					
	Mean (SD)	Mean (SD)					
Age	65 (4.37)	66.5 (4.23)	-1.5	-7.92/	-0.53	8.03	*0.60
				4.92			
BMI	25.84 (3.29)	24.27 (3.00)	1.56	-3.28/	0.75	7.63	*0.47
				6.41			
Time of anesthesia (minutes)	298.00 (112.19)	190.83 (112.193)	107.16	-82.24/	1.56	4.061	*0.19
				296.58			
Time of surgery (minutes)	281 (110.25)	153.33 (50.66)	127.66	-26.86/	2.11	5.06	*0.087
				282.19			
	N (%)	N(%)			χ^2^		p-value
ASA							
I	0 (0%)	0 (0%)			0.11		**0.74
II	3 (60%)	3 (50%)					
III	2 (40%)	3 (50%)					
IV	0 (0%)	0 (0%)					
Type of surgery							
Robot-assisted prostatectomy	3 (60%)	3 (50%)			0.11		**0.74
Other types	2 (40%)	3 (50%)					
Comorbidity	5 (100%)	6 (100%)			0.00		**1.00

*t-test p-value

**Chi-square test p-value

**Table 2 (abstract A52).** Anesthesia management and awakening evaluation

N=11 (100%)	C-group	Routine care group	Mean difference	95% CI	t	dF error	p-value
	N=5 (46%)	N=6 (54%)					
Heart rate	73.15 (10.20)	76.55 (11.63)	-3.40	-17.57/	-0.54	8.98	0.59
				10.76			
Systolic pressure	104.75 (15.03)	97.61 (15.09)	7.13	-14.15/	0.76	8.51	0.46
				28.43			
Diastolic pressure	70.9 (15.40)	65.55 (16.43)	5.34	-16.45/	0.55	8.83	0.59
				27.14			
Mean blood pressure	72.56 (14.88)	76.24 (14.02)	-3.67	-25.51/	-0.38	8.23	0.70
				18.15			
MAC	0.64 (0.30)	0.48 (0.35)	0.12	-0.25/	0.85	8.68	0.41
				0.55			
Desflurane (%)	3.27 (1.64)	2.62 (1.53)	0.64	-1.59/	0.65	8.49	0.52
				2.88			
NRS awakening	2.00 (0.99)	0.00 (0.00)	2.00	-2.30/	1.29	4.00	0.26
				6.30			
RASS awakening	-0.2 (0.7)	0.33 (0.81)	-0.533	-1.43/	-1.37	7.95	0.20
				0.36			
Awakening time	8.00 (3.43)	6.66 (2.58)	1.33	-4.16/	0.58	6.15	0.57
				6.83			

NRS numeric rate scale RASS Richmond Agitation-Sedation Scale

**Table 3 (abstract A52).** MoCA, SF-36, and QoR at baseline, 1 day, 7 days, and 30 days after surgery

N=11 (100%)	C-group	Routine care group	Mean difference	95% CI	t	dF error	p-value
	N=5 (46%)	N=6 (54%)					
QoR after 1 day	105.2 (24.42)	119.5 (10.7)	-14.3	-56.82/28.22	-0.88	4.63	0.41
MoCA at basaline	23.6 (3.57)	21.00 (3.28)	2.6	-2.30/7.50	1.21	8.14	0.25
MoCA after 1 day	23.4 (25.30)	22.33 (5.57)	1.06	-6.59/8.72	0.31	8.65	0.75
Telephonic MoCA after 7 days	10.80 (2.76)	10.50 (3.27)	0.3	-3.57/4.17	0.17	8.89	0.86
Telephonic MoCA after 30 days	61.3 (2.54)	75.91 (2.87)	-14.61	-48.35/14.62	-1.33	4.45	0.24
SF-36 at baseline	62.99	66.68 (6.23)	-3.69	-37.50/30.11	-0.28	4.94	0.78
SF-36 after 1 day	63.13	66.24 (10.42)	-3.11	34.84-41.06/6	-0.21	4.63	0.83
SF-36 after 7 days	58.74	66.75 (9.53)	-8.00	-1.43/	-0.5	5.26	0.61
				0.36			
SF-36 after 30 days	105.20	119.50 (13.52)	-14.30	-45.87/29.85	-0.88	4.63	0.41

**Table 4 (abstract A52).** Value of standard deviations from baseline for each patient and comparison of incidence of POCD between groups

	MoCA time				MoCA time		
C-group ID	At 1 day	At 7 days	At 30 days	Routine care group ID	At 1 day	At 7 days	At 30 days
1	0.81	**-1.35**	0.81	1	**-2.43**	**-2.74**	-0.30
2	**-1.08**	0.54	0.00	2	0.30	1.52	0.91
3	1.35	1.35	0.27	3	1.83	2.74	2.74
4	**-1.08**	**-1.35**	0.27	4	1.52	**-1.52**	0.91
5	-0.27	-0.91	0.81	5	-0.61	-0.30	**-2.13**
				6	1.83	0.30	2.13
Number of POCD events (%)	2 (18%)	2 (18%)	0 (0%)		1 (6%)	2 (18%)	1 (6%)
MoCA time		χ^2^		p-value			
At 1 day		0.03		0.85			
At 7 days		0.05		0.81			
At 30 days		0.91		0.33			

### A53 Post-operative delirium in elderly patients undergoing major abdominal surgery: incidence of a long-term issue

#### E. Cappellini ^1^, E. Angeli ^1^, G. Calonaci ^1^, C.V. Piccolo ^1^, A. Galardi ^1^, N. Basso ^1^, F. Livi ^1^, L. Foti ^2^, B. Mura ^2^, G. Villa ^1^, S. Romagnoli ^1, 2^

##### ^1^ School of Anaesthesia, Critical Care and Pain medicine, University of Florence, Florence, Italy; ^2^ Department of Oncological Anaesthesia and Intensive Care, Careggi University Hospital, Florence, Italy

###### **Correspondence:** E. Cappellini

*Journal of Anesthesia, Analgesia and Critical Care 2023,* **3(Suppl 1):**A53

Background: Post-operative delirium (POD) is one of the most insidious complication in elderly and is associated with prolonged mechanical ventilation, increase length of stay (LOS) in hospital, long term neurocognitive impairment and an overall increase mortality. In literature, the incidence POD after major abdominal surgery is 25%. The aim of the study is to assess the incidence of POD in elderly patients undergoing major abdominal surgery; adverse events and risk factors for POD have also been recorded.

Materials and methods: We conducted an observational, prospective, monocentric study. After obtaining appropriate informed consent we enrolled patients aged 60 yrs. or older, scheduled for major abdominal elective surgery from august 2019 to October 2019 at department of Oncological Anaesthesia and Intensive Care of Careggi University Hospital. Five days, 1 – 3 months follow-up were recorded. Confusion Assessment Method (CAM) and the CAM–ICU were used as diagnostic tools for POD assessment.

Results: From the 110 patients enrolled, 11 developed POD with an incidence of 10% (C.I 5,1 – 17,2). No differences between the delirium post-operative Group (PODg) and not-POD group (nPODg) was found in the pre-operative data, except for an older age and a higher value of Charlson Comorbidity Index in PODg. PODg experienced higher incidence of intraoperative burst suppression (60% vs 24%; p<0.05) and prolonged amount of intraoperative hypotension (23,7 min vs 6,3 min; p<0,05). PODg experienced more morphine prescription and longer mobilization times, LOS in ICU and higher incidence of post-operative complications (63% vs 17%; p <0.05). PODg showed a worsening of the Short Blessed Test performance at follow-up.

Conclusions: POD is a serious complication associated with morbidity and poor neurocognitive performance still after discharge, with worsening of patient’s quality of life. In addition, it is important to understand how neurocognitive decline of patients who experienced POD impact on care givers quality of life with physical, psychological, and financial burdens. Within this context a multidisciplinary strategy due to reduce the iatrogenic risk factors that can precipitate the fragile patient's compensation is pivotal.

### A54 General versus spinal anesthesia for geriatric patients undergoing fixation for hip fractures

#### L. Al-Husinat ^1^, S. Al Sharie ^2^, Z. Al Modanat ^1^, P. Pelosi ^3^, D. Battaglini ^4^

##### ^1^ Department of Clinical Sciences, Faculty of Medicine, Yarmouk University, Irbid, Jordan; ^2^ Faculty of Medicine, Yarmouk University, Irbid, Jordan; ^3^ Department of Surgical Sciences and Integrated Diagnostics, University of Genoa, Genoa, Italy; ^4^ Anesthesia and Intensive Care, San Martino Policlinico Hospital, IRCCS for Oncology and Neuroscience, 16132, Genoa, Italy

###### **Correspondence:** L. Al-Husinat

*Journal of Anesthesia, Analgesia and Critical Care 2023,* **3(Suppl 1):**A54

Keywords

Hip fractures, Geriatrics, Spinal anesthesia, General anesthesia, Perioperative care.

Abstract

Introduction: Hip fractures are common injuries in geriatric population, which often require surgical intervention. The choice between spinal and general anesthesia requires careful consideration of patient's medical condition, surgical procedure, risks, and benefits of each technique. We aimed to investigate the differences in perioperative care and mortality in patients receiving spinal (SA) and general anesthesia (GA) for hip fractures repair.

Methods: This is a retrospective multicentered study conducted in Jordan. Demographic, clinical, preoperative, operative, and postoperative data of geriatric patients (age more than or equal to 65) who underwent surgical fixation for hip fractures under spinal or general anesthesia were collected.

Results: Overall, 1083 patients were included, of whom 485 were males (44.8%) and 598 were females (55.2%). The median age for the study population was 78-year-old. Diabetes mellitus, hypertension, Alzheimer disease and osteoporosis were the most common comorbidities. 674 patients (37.4%) underwent spinal anesthesia, and 410 patients (62.6%) underwent general anesthesia. The median time to surgery in the overall cohort was 2 (4-1) days. Time to surgery was significantly longer in GA than SA (3 (4-2) days GA and 2 (4-1) GA, p = 0.000).

Cefazolin was the most commonly used class of antibiotics, followed by a combination of Cefazolin and Vancomycin. SA group used more antibiotics than GA group (p = 0.000). antegrade intramedullary nailing (IMN) was the most common technique of fixation, followed by hemiarthroplasty. SA group was fixed with IMN technique more than GA ([443 (62.76%) SA and 264 (64.39%) GA], p=0.390).

Cementation was needed more in the SA group ([164 (81.19%)SA and 72 (61.02%) GA], p=0.000).

Postoperatively, opioids were used more frequently in the SA group [424 (62.91%)] more than the GA group [229 (55.85%). p = 0.021].

Overall, 152 patients died after 1-year postoperatively (14.0%) [98 patients (64.5%) in SA and 54 in GA group, 29.6% of them died 1-year postoperatively, 25.7% after 6 months, and 18.4% in the hospital.

Conclusion: To our knowledge no studies have yet indicated the differences between general anesthesia and spinal anesthesia in elderly patients undergoing hip fracture repair. Our study found that spinal and general anesthesia are both commonly used in geriatric patients undergoing surgical fixation for hip fractures. However, SA was associated with shorter time to surgery, higher use of antibiotics, more frequent use of cementation, and more frequent use of opioids postoperatively. The choice between SA and GA should be made carefully based on patient-specific factors, comorbidities, and surgical requirements.

## Bioethics

### **A55 Would you write it down? - delegation of end of life decision-making to the intensivist**

#### L. Maderna, G.L. Formicola, C.D. Votta, L. Mariconti, L. Rota, G. Russo

##### Ospedale Maggiore di Lodi - ASST Lodi, Lodi, Italy

###### **Correspondence:** L. Maderna

*Journal of Anesthesia, Analgesia and Critical Care 2023,* **3(Suppl 1):**A55

BACKGROUND: Medical progress in the recent years resulted in increased survival of people with chronic organ failures and in longer life expectancy. [1] Since 2017 is possible to express living wills and share plans of care [2]. However, this possibility remains untapped by the majority of people and physicians, as if the “end of life” concept is still a kind of taboo; decisions are delayed longer and longer until it’s no more possible to avoid the conversation [3].

MATERIALS AND METHODS: Retrospective revision of ICU consultancies from 11/01/2022 to 01/31/2023 in a single centre (Lodi Hospital).

RESULTS: We evaluated 182 patients; 22% ended up in a non-indication to ICU admission (“too sick to benefit”); 65% of consultancy requests was received during afternoon or night shifts; 34% was requested from the ED. Mean patients age was 80.3 years; 73% of this was in a very frail condition (dependency from a caregiver or in a nursing home). 26.8% had three or more severe comorbidities. We received one request for “shared decision making” in the absence of an acute necessity and one patient had a living will. We also received 4 doubled “confirmatory” requests.

COMMENTS End-of-life decisions are taken in a situation of acute organ failure (46% respiratory failure in our study) which requires fast decisions that often become irreversible. Almost none of the patients had any living will or planned care in case of acute organ failure, even if affected from chronic diseases. The intensivist is invested with the determining role of decision about completely unknown patients, often alone, quickly, and during understaffed shifts. It seems that other specialists call the intensivist focusing their clinical evaluations more on the single organ failure than on the whole patient condition; this may happen because death may be considered a professional failure, it’s difficult to talk with patients and with shocked, unprepared relatives, or, perhaps, it may represent a sort of self-protection determined by the so-called “defensive medicine”. This condition may generate frustration, increase burnout, and, being a time-consuming activity, it could eventually lead to a disadvantage for people who benefit from ICU care by diverting staff's attention in the absence of a consultancy dedicated specialist. It seems necessary a better application of 219/2017 law; early involvement of “complementary palliative care”; avoidance of “defensive medicine”; a validated and shared decision-making algorithm; education and training in hospitals and general practice. It might also be useful, to take the right time to talk with patients and caregivers, to have a specific, multi-disciplinary outpatient clinic targeted to people with chronic and potentially worsening conditions to promote shared decisions and living wills when not urged by any life-threatening condition.

REFERENCES

1. Giannini A., Gruppo di Studio di Bioetica SIAARTI: “le grandi insufficienze d’organo end stage: cure intensive o cure palliative?” SIAARTI - 2013.
2. https://www.gazzettaufficiale.it/eli/id/2018/1/16/18G00006/sg
3. Elia F Vergano M Di Meglio L “The patient who fell off a skyscraper” Intensive Care Med 2018 Oct;44(10):1770

## Cardiothoracic Vascular Anesthesia

### **A56 Extracorporeal hemadsorption therapy as a potential option for rapid removal of Ticagrelor in a high-risk cardiac surgical patient**

#### L.M. Titherington ^1^, R. Mandarano ^2^, M.F. Ostuni ^2^, S. Bevilacqua ^2^, I. Galeotti ^2^, G. Olivo ^2^

##### ^1^ School of Human Health Sciences, University of Florence, Florence, Italy; ^2^ Cardiac Anaesthesia and Intensive Care, Department of Anaesthesiology, Careggi University Hospital, Florence, Italy

###### **Correspondence:** L.M. Titherington

*Journal of Anesthesia, Analgesia and Critical Care 2023,* **3(Suppl 1):**A56

Background

Cardiac patients on antiplatelets or oral anticoagulation undergoing emergency cardiac surgery are at an increased risk of developing perioperative bleeding due to insufficient discontinuation time[1]. Cytosorb (Cytosorbents, Monmouth Junction, NJ) is a blood purification technology that has been demonstrated to remove the antiplatelet P2Y12 receptor inhibitor, Ticagrelor, from the blood.

Case report

A 69 years old male patient, height 180 cm, weight 85 Kg, presented to the emergency room with myocardial infarction. Dual antiplatelet therapy (DAPT) with loading doses of ticagrelor and aspirin was administered. He underwent percutaneous coronary intervention (PCI) with one drug eluting stent (DES) implantation and subsequently developed cardiogenic shock due to papillary muscle rupture. He required multi-organ support and was brought to emergency surgery.

Considering the high risk of bleeding we decided to implement Cytosorb in parallel to the Cardiopulmonary Bypass (CPB).

Preoperative laboratory assays showed a haematocrit of 39,4%, platelet count 348000/L, INR 1, aPTT 32 sec, and fibrinogen 483 mg/dL. Preoperative VerifyNow P2Y12 PRU (Platelet Reactivity Unit) test value was 6, signifying a low platelet reactivity (LPR). Mitral valve replacement and tricuspid repair via right mini-thoracotomy were performed. Aortic cross-clamping time was 83 minutes, CPB time 130 min, and surgical time was 180 min. Haemostasis was obtained through the use of protamine in a 1:1 ratio to initial heparin, fibrinogen 2 gr, and 1 platelet pool. At the end of the surgery the ROTEM test was normal and the PRU test was 9.

Cytosorb was continued in ICU allowing complete free-ticagrelor adsorption (3 hours).

12 hours after surgery (24 hours after DAPT) PRU test was 178 (OPR optimal-platelet-reactivity range for P2Y12 receptor binding drugs), ARU (Aspirin resistance units) test was 478 (optimal range for aspirin therapy) and total chest tube output was 280 ml. On postoperative day 2 the patient was weaned from IABP and aspirin was restarted. Clopidogrel was added to aspirin on postoperative day 5 according to the platelet function tests, without a loading dose.

Patient’s total hospital stay was 19 days including 11 days in CICU. Total number of pRBCs transfused were 3 with only 1 platelet transfusion required. A coronary angiography one month after surgery showed complete DES patency.

Conclusions

Ticagrelor adsorption with Cytosorb during CPB is a safe and effective method to reduce bleeding complications in high-risk bleeding cardiac surgical patients. Careful haemostasis and serial measurements of platelet function permit the simultaneous management of the thrombotic risk.

A formal written consent was acquired from the patient for data publication.

References

1. Kietaibl, Sibylle; Ahmed, Aamer; Afshari, Arash; Albaladejo, Pierre; Aldecoa, Cesar; Barauskas, Giedrius; De Robertis, Edoardo; Faraoni, David; Filipescu, Daniela C.; Fries, Dietmar; Godier, Anne; Haas, Thorsten; Jacob, Matthias; Lancé, Marcus D.; Llau, Juan V.; Meier, Jens; Molnar, Zsolt; Mora, Lidia; Rahe-Meyer, Niels; Samama, Charles M.; Scarlatescu, Ecaterina; Schlimp, Christoph; Wikkelsø, Anne J.; Zacharowski, Kai. Management of severe peri-operative bleeding: Guidelines from the European Society of Anaesthesiology and Intensive Care: Second update 2022. European Journal of Anaesthesiology 40(4):p 226-304, April 2023. | DOI: 10.1097/EJA.0000000000001803

### A57 Use of landiolol for new-onset atrial fibrillation in ICCU patients

#### M. Rocco ^1^, G. D'Arista ^1^, S. Salafica ^2^

##### ^1^ Emergency and Admissions Department, Sapienza University, Sant’ Andrea University Hospital, Rome, Italy; ^2^ Emergency and Admissions Department, School of Anesthesia, Resuscitation, Intensive Care and Pain, Sapienza University, Rome, Italy

###### **Correspondence:** S. Salafica

*Journal of Anesthesia, Analgesia and Critical Care 2023,* **3(Suppl 1):**A57

Background

Tachyarrhythmias commonly occur in patients admitted to the Cardiac Intensive Care Unit (ICCU), high risk patients are those septic and undergoing cardiac surgery or major surgery [1,2,3]. Atrial fibrillation (AF) may also result in extended hospital stays and can significantly increase morbidity [4,5]. Compared to other beta-blockers, landiolol is an ultra-short acting beta-blocker with a very high beta1-selectivity, with only marginal effects on blood pressure and myocardial contractility [6,7,8]. In particular, landiolol was effective in rapidly controlling heart rate (HR) and could be used in vasopressor-dependent patients without significant effects on blood pressure or as a bridging treatment for adjunctive oral β-blocker in heart failure (HF) patients [9,10,11].

Case report

The patient was a 66-year-old woman with valvular disease. Fifteen days after cardiac surgery she had an episode of AF with HR increased to 150 bpm and systolic blood pressure (SBP) decreased to 70 mmHg. Continuous crystalloids infusion was increased, and norepinephrine was increased up to 0.2 mcg/kg/min without improving hemodynamics. Therefore, continuous landiolol infusion was started with a rate of 10 mcg/kg/min and was increased to 15 mcg/kg/min in three steps, titrated according to HR response without any significant effect on SBP. Six hours later, the patient converted to sinus rhythm and her SBP increased to 120 mmHg with a MAP>70 mmHg, which led to a reduction of norepinephrine (Figure 1).

Two days later landiolol was stopped and norepinephrine was tapered to 0.05 mcg/kg/min. The patient remained in sinus rhythm and her cardiac output (CO) improved, resulting in decreased norepinephrine demand with a stable MAP>75 mmHg.

Conclusion

In this report, we present a critically ill patient with new-onset AF who was successfully treated with a continuous intravenous administration of landiolol for 48h. Patients admitted to ICCUs often require norepinephrine to maintain adequate blood pressure which may induce the development of supraventricular tachyarrhythmias, like AF, in these patients [12]. Landiolol, compared to other beta-blockers, shows faster pharmacokinetics, higher potency and cardioselectivity (β1/β2-selectivity 255:33) with a less potent negative inotropic effect [13]. Accumulating evidence is supporting the use of ultrashort-acting β1-blockers (like landiolol) suitable for the acute management of supraventricular arrhythmias in ICCU patients, without adverse effects on inotropia and blood pressure [14,15,16,17,18].

References

1. Oprea AD, Lombard FW, Kertai MD. Perioperative beta-adrenergic blockade in noncardiac and cardiac surgery: a clinical update. J Cardiothorac Vasc Anesth. 2019;33:817–832.
2. Domanovits H, Wolzt m and Stix G Landiolol: pharmacology and its use for rate control in atrial fibrillation in an emergency setting. Eur Heart J Suppl. 2018;20(Suppl A):A1–A3.
3. Drikite L, Bedford JP, O’Bryan L. Treatment strategies for new onset atrial fibrillation in patients treated on an intensive care unit: a systematic scoping review. Crit Care. 2021;25(1):257.
4. Hao J, Zhou J, Xu W. Beta-Blocker Landiolol Hydrochloride in Preventing Atrial Fibrillation Following Cardiothoracic Surgery: A Systematic Review and Meta-Analysis. Ann Thorac Cardiovasc Surg. 2022;28:18–31.

©Consent to publication collected from the patient's family.

**Fig. 1 (abstract A57).**
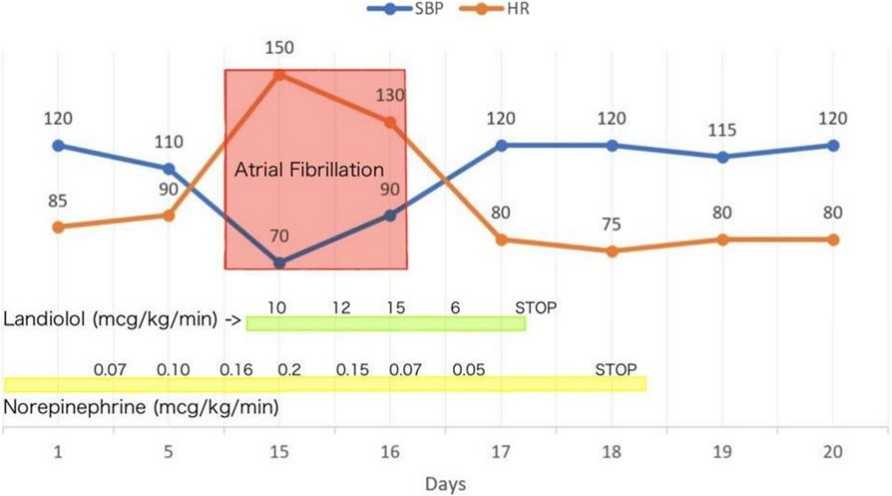
See text for description

### A58 The role of thromboelastography-guided hemostatic therapies during the treatment of aortic dissection

#### P. Raimondo ^1^, G. Di Bari ^2^, E. Rollo ^3^, S. Lenoci ^1^, G. Rubino ^1^, M.A. Villani ^1^, A. Stripoli ^1^, A. Armenise ^1^, G. Colantuono ^1^, G. Fiore ^1^, S. Grasso ^1^

##### ^1^ Department of Precision and Regenerative Medicine and Jonica Area, Anesthesia and Intensive Care II, University of Bari, Bari, Italy; ^2^ Department of Precision and Regenerative Medicine and Jonica Area, Cardiac Surgery, University of Bari, Bari, Italy; ^3^ Interdisciplinary Medicine Department, School of Anesthesia, Resuscitation, Intensive Care and Pain, University of Bari, Bari, Italy

###### **Correspondence:** E. Rollo

*Journal of Anesthesia, Analgesia and Critical Care 2023,* **3(Suppl 1):**A58

BACKGROUND

Although substantial advances in diagnosis and surgical techniques, the acute type A aortic dissection remains associated with high morbidity and mortality rates. Postoperative bleeding, several perioperative transfusions and reoperation for bleeding are the most frequent complications for this condition. The dissection itself activates the coagulation system and consumes platelet, clotting factors, and fibrinogen, even before surgery [1-2]. Blood flow through the false lumen is a powerful activator of the hemostatic system even before the operation. This remarkable activation may influence postoperative outcome of AAD patients [3]. In this consumption coagulopathy setting, literature suggests that hemostatic therapy should focus on the rapid and sufficient supplementation of clotting factor and fibrinogen after hypothermic circulatory arrest [4]. In line with the guidelines recommendations, the aim of the study is to manage the patients’ coagulation corrections through Thromboelastographic (TEG) to improve coagulation and outcome.

MATERIALS AND METHODS

Between 2020 and 2022, we enrolled adult patients with age over 18 years, admitted to the Department of Cardiac Surgery (Policlinico of Bari, Italy) for acute type A aortic dissection. We collected all medical, surgical, CPB and TEG perioperative data. TEG was performed at the end of CPB (post protamine) and after coagulation’s correction.

RESULTS

We enrolled 17 patients (10 man and 7 women) with mean age of 66,7 years, undergoing emergency surgery for acute type A aortic dissection. All patients underwent CPB with a mean duration of 224,4 minutes. Nine patients did not receive any transfusion, the remaining received in mean 278,52 ml of Blood red cells. A mean of packed red blood cell (2,5 pool), Platelets (1,4 pool), fresh frozen plasma (2,4 pool) Fibrinogen (1.75 gr), Tranexamic Acid (1,33 gr), Kedcom (1450 UI), Uman Complex (1750 UI) and Factor VII (600 UI) have been used.

CONCLUSIONS

In our brief case series, TEG early confirms the hypocoagulability, related to several factors: dissection, surgical trauma, cardiopulmonary bypass, and hypothermic circulatory arrest. TEG permits the immediate and bedside evaluation of Reaction Time, K, Angle, Maximum Amplitude, Lysis Time and Coagulation Index and their TEG guided correction (Figures. 1-2). The outcome of surgery for acute aortic dissection is improving, but many debates remain as to the optimal treatment and coagulation management.

REFERENCES

1. Guan X, Li J, Gong M, et al. The hemostatic disturbance in patients with acute aortic dissection: A prospective observational study. Medicine (Balti- more) 2016;95:e4710.
2. Zindovic, I., Sjögren, J., Bjursten, H., Ingemansson, R., Ingimarsson, J., Larsson, M., ... & Nozohoor, S. (2019). The coagulopathy of acute type A aortic dissection: a prospective, observational study. Journal of cardiothoracic and vascular anesthesia, 33(10), 2746-2754.
3. Paparella D, Rotunno C, Guida P, et al. Hemostasis alterations in patients with acute aortic dissection. Ann Thorac Surg 2011;91:1364–9.
4. Liu, Y., Han, L., Li, J., Gong, M., Zhang, H., & Guan, X. (2017). Consumption coagulopathy in acute aortic dissection: principles of management. Journal of cardiothoracic surgery, 12(1), 1-8.

**Fig. 1 (abstract A58).**
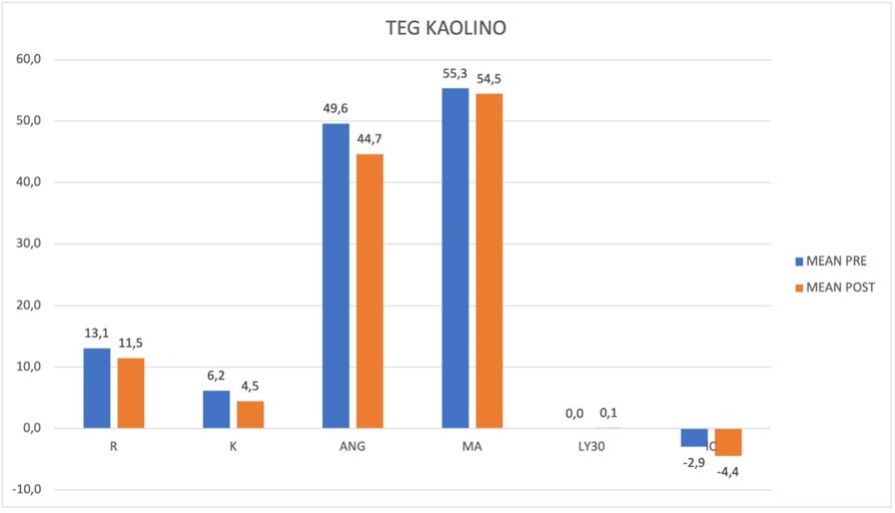
TEG Kaolin Trace: Mean of value Pre and post correction

**Fig. 2 (abstract A58).**
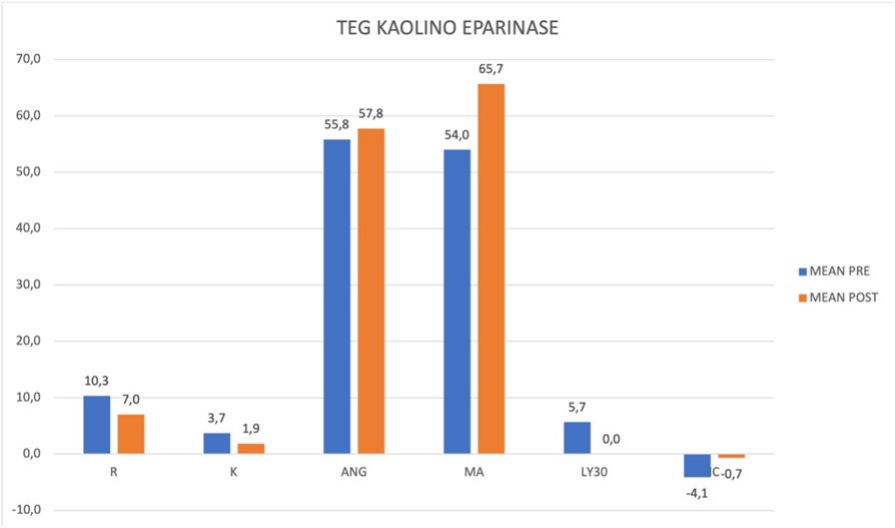
TEG Kaolin – Heparinase Trace: Mean of value Pre and post correction

### A59 Impact Of Tavi on sublingual microcirculation in patients with severe aortic stenosis: an observational study

#### J. Rama, A. Zanin, M. Mastrantoni, E. Bergamini, M. Taiana, A. Russo, L. Gottin

##### Università degli Studi di Verona, Verona, Italy

###### **Correspondence:** J. Rama

*Journal of Anesthesia, Analgesia and Critical Care 2023,* **3(Suppl 1):**A59

INTRODUCTION

The study of microcirculatory bed gained in recent years an increasing interestfor the evaluation different pathological states, particularly of shock. Furthermore recent studies have demonstrated improvement of the microcirculatory coronaric flow after TAVI. Unfortunately, literature about the correlation between these interventions and systemic microcirculation is still lacking.

PURPOSE

The aim of our study consists in evaluating the possible correlation between sistemic microcirculation (evaluated through the analysis of sublingual microcirculation) and TAVI. Our goal was to investigate if TAVI-related changes in hemodynamic could be associated alsowith modifications of sublingual microcirculatory bed. We tried also to investigate the maintenance or loss of the so-called hemodynamic coherence (concordance between macro and microcirculatory parameters) following TAVI.

METHODS

The analysis of the sublingual microcirculation was made using a CYTOCAM© device, a handheld device which exploits the IDF tecnology to produce a real-time image of the sampled field. Measurements were collected in 3 different moments: T0, before TAVI; T1, immediately after TAVI; T2, 24 hours after. We recorded the following perfusion parameters of microcirculation: TVL, TVD, PPV, PVD. Contextually, lactate values were obtained at T0 and T1. Sample was composed of 28 patients undergone Transfemoral TAVI between November 2023 and February 2023 in the hemodynamic lab of our institution. The group comprehended patients with AS both high flow high gradient and low flow low gradient. All the procedures were performed under moderate sedation. Informed consent was collected before the procedure for each patient.

RESULTS

Analysis of our data showedno statistically significant differencefor all the parameters in the T0-T1 and in the T0-T2 intervals. We recorded a trend in deterioration of microcirculation perfusion immediately after the procedure, not reaching the significance (p>0.05). The only parameter showing an improvement was TVL at 24 hours in low flow low gradient patients (p 0.07, Table 1). We have also seen a tendency of loss of hemodynamic coherence in some patients.

CONCLUSIONS

In our study we have tried to find a correlation between TAVI and microcirculatory bed modification. We havenot found a significant relationship in terms of improvement of the microcirculatory perfusion parameters after TAVI. However, we recorded a tendency of improvement of many parameters in the subpopulation of low flow low gradient patients, which could stimulate future researches.

**Table 1 (abstract A59).** See text for description

T0	T2	Media + Dev. Standard T0	Media + Dev. Standard T2	p-value
**TVD T0**	**TVD T2**	**14,9±3,08**	**12,2±3,72**	**0,251**
**PVD T0**	**PVD T2**	**18,8±3,73**	**11,3±3,07**	**0,347**
**PPV T0**	**PPV T2**	**93,8±7,78**	**92,9±6,16**	**0,624**
**TVL T0**	**TVL T2**	**8,96±3,15**	**6,03±3,45**	**0,07**

### A60 Use of levosimendan in difficult weaning due to diastolic dysfunction: case report

#### A. Palma, M. Alfieri, P.F. Marsilia, R. Esposito, F. Cirillo, M. Ciamillo, G. Mercadante, F. Maurelli, M. De Cristofaro

##### Cardarelli Hospital, Naples, Italy

###### **Correspondence:** A. Palma

*Journal of Anesthesia, Analgesia and Critical Care 2023,* **3(Suppl 1):**A60

Introduction: Left ventricular diastolic dysfunction (LVDD) is associated to weaning failure (1). Spontaneus breathing trial (SBT)-induced central hemodynamic changes monitored by critical care echocardiography (CCE) helps guide weaning of patients at high risk of weaning pulmonary edema (WIPO). Levosimendan improves both LV sistolic and diastolic functions and can be used to optimize cardiac function before of SBT in anticipation of a difficult weaning process in patients with LVDD (2).

Methods: An obese 44-years-old man with known dilated cardiomyopathy arrived at the Emergency Department with a pulseless ventricular tachycardia, treated with electrical shock, followed by rapid ventricular response atrial fibrillation, treated with more 4 shocks and subsequent restoration of sinus rhythm. The patient was intubated and admitted in Intensive Care.

Results: After 72h, a SBT failed because of WIPO. A CCE showed an E/A ratio of 1,37 cm/sec with DT of 145,0 ms , E/e’ 26,57 cm/sec and a left atrial Volume Index of 38ml/m2 identifying a grade II LVDD (3). A chest X-ray revealed perihilar and increased vascular markings. Levosimendan was added to standard therapy. 48h later, a CCE demonstrated an improvement in LVDD (Fig.1): E/A ratio was 1,06 cm/sec; DT 210 msec, E/e’ 10,9 cm/sec. Weaning was successfull on day 5 and patient was discharged on day 7.

Conclusion: A combination of clinical parameters with bedside CCE can not only help in setting up therapy, but also facilitate weaning from ventilation. Use of CCE to study LVDD can help to identify patients who can be difficult to wean off the ventilator. Furthermore, levosimendan can be associated to a better weaning outcome in patients with LVDD through its beneficial effect on cardiac function.

Written informed consent for the publication has been obtained by patient after discharge.

Transmitral pattern during weaning ventilation before and after levosimendan therapy

References

1. Goudelin M et al. ICM 46:1371-1381, 2020
2. Ifigeneia K et al. CRIT CARE RES PRACT Vol 2019 Art ID 7169492:8 pages,2019
3. Nagueh SF et al. EHJCI 17:1321-60,2016

**Fig. 1 (abstract A60).**
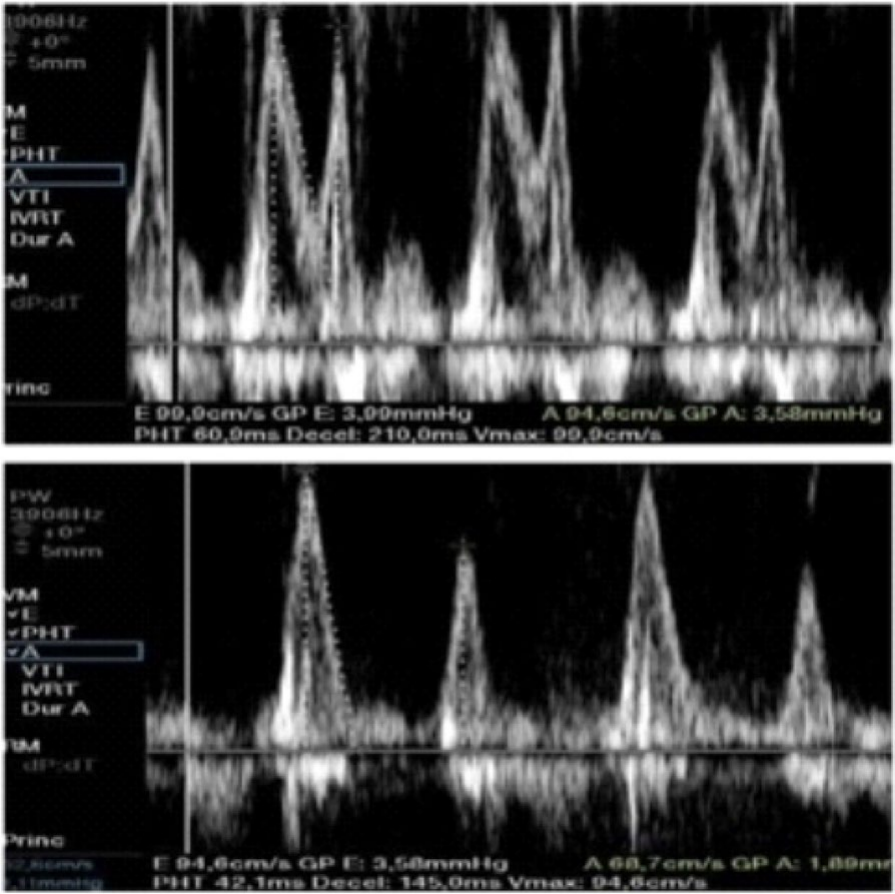
See text for description

### A61 TEG controlled administration of bivalirudin in heparin allergic patient undergoing Carotid Endarterectomy (CEA)

#### F. Lombardi ^1,2^, E.C. Colacchio ^3^, P. Raimondo ^4^, A. Mascia ^4^, E. Rollo ^4^, V. Delmonte ^2^, G. Colacchio ^5^

##### ^1^ U.O.C. Cardiologia, Università Cattolica Sacro Cuore, Roma, Italy; ^2^ U.O.C. Medicina Perioperatoria, Dipartimento di Emergenza e Urgenza, Ospedale Generale Regionale F. Miulli, Acquaviva delle Fonti, Italy; ^3^ Clinica di Chirurgia Vascolare ed Endovascolare, Università degli studi di Padova, Padova, Italy; ^4^ Dipartimento di Emergenza e Trapianti d'organo, Sez Anestesia e Rianimazione II, Policlinico di Bari, Bari, Italy; ^5^ U.O.C. Chirurgia Vascolare, Ospedale Generale Regionale F. Miulli, Acquaviva delle Fonti, Italy

###### **Correspondence:** A. Mascia

*Journal of Anesthesia, Analgesia and Critical Care 2023,* **3(Suppl 1):**A61

BACKGROUND

Heparin is the drug of choice for anticoagulation during Carotid Endarterectomy (CEA) required to minimize dangerous thromboembolic events, but in patients with its contraindication, bivalirudin is frequently used as a short half-life direct thrombin inhibitor. In the vascular surgical and endovascular setting the use of the heparin is codified, but there are no defined protocols for the management of bivalirudin during open surgery. The aim of this case report is to define and evaluate the results of TEG guide administration of bivalirudin during these procedures.

CASE PRESENTATION AND TREATMENT

An 87-year-old lady with severe pulmonary hypertension, ASA III, NYHA III, allergic to sodium heparin and to low molecular weight heparins, was scheduled for an elective CEA, after obtaining the informed consent. Approximately 5 months earlier, the same patient underwent percutaneous correction of tricuspid regurgitation with TriClip (Abbott) and the procedure was successfully completed using bivalirudin for anticoagulation. According to body weight and kidney function, the administration of the bolus has been followed by continuous infusion; bivalirudin was also diluted in the surgery washing solutions during CEA. Immediately before carotid occlusion, bivalirudin was administered as a 60 mg bolus followed by a 1,4 mg/Kg/h infusion. Following a team-agreed protocol, the infusion rate was modulated according to the value of the Activated Clotting Time (ACT), double-checked at close intervals and Thromboelastogram (TEG) before, during and in the hours following the procedure (Fig. 1);

(Fig. 2); (Fig. 3). The patient was discharged home in 4 days without significant complications. Bivalirudin provided adequate anticoagulation and good surgical outcome without any adverse effects in this patient.

DISCUSSION and CONCLUSION

Viscoelastic testing provides a rapid picture of whole blood coagulation dynamics and hemostasis that can be reviewed and evaluated bedside. So, this experience supports the utility of TEG to guide the administration of bivalirudin as an alternative to heparin in surgical vascular procedures. Before, during and after CEA, TEG guided the coagulation state of the patient, monitoring the effect of bivalirudin and the function of coagulation system components, to ensure the possibility of targeted corrective action, in case of hemorrhagic complications, until the end of the drug effect.

Informed consent to publish had been obtained

REFERENCE

- Bivalirudin Use in Carotid Endarterectomy in a Patient with Heparin-Induced Thrombocytopenia Ann Pharmacother 2006;40:340-3. DOI 10.1345/aph.1G307

**Fig. 1 (abstract A61).**
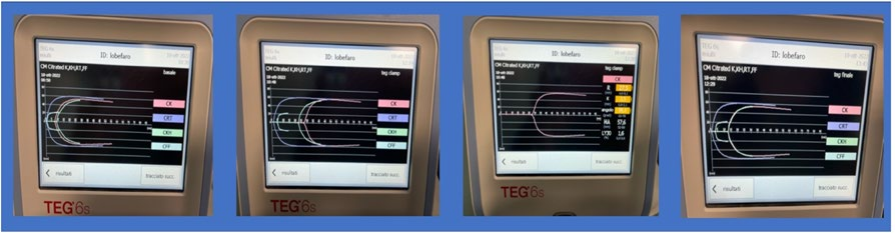
Perioperative Tromboelastogram

**Fig. 2 (abstract A61).**
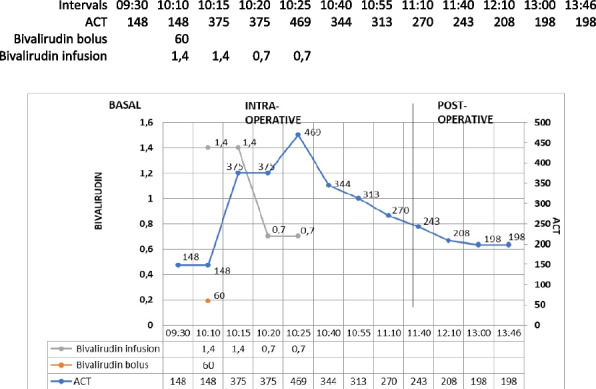
Bivalirudin and ACT data

**Fig. 3 (abstract A61).**
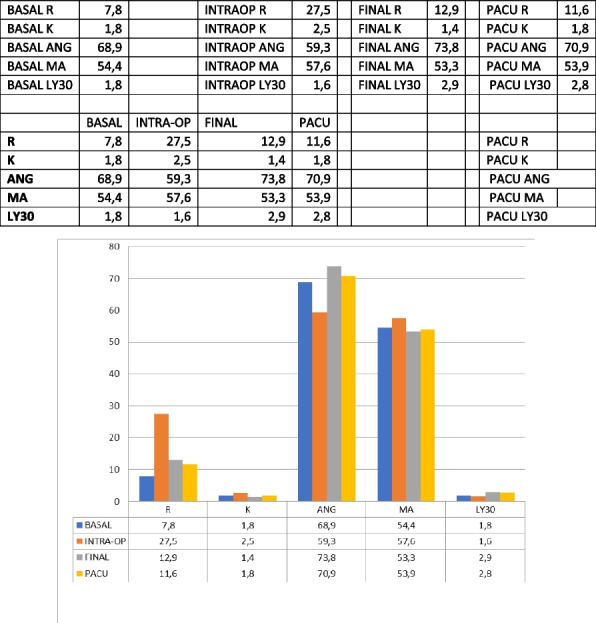
TEG Data

### A62 Inferior vena cava distensibility from subcostal and trans-hepatic imaging using both M-mode or artificial intelligence: a prospective study on mechanically ventilated patients

#### L. La Via ^1^, F. Sanfilippo ^2^, V. Dezio ^1^, P. Amelio ^3^, G. Genoese ^4^, F. Franchi ^5^, A. Messina ^6^, C. Robba ^7^, A. Noto ^8^

##### ^1^ AOU Policlinico G.Rodolico-San Marco, Catania, Italy; ^2^ Dipartimento CHIRMED, Università di Catania, Catania, Italy; ^3^ School of Anaesthesia and Intensive Care, University Magna Graecia, Catanzaro, Italy; ^4^ Division of Anesthesia and Intensive Care, University of Messina, Policlinico G. Martino, Messina, Italy; ^5^ Anesthesia and Intensive Care Unit, University Hospital of Siena, University of Siena, Siena, Italy; ^6^ Humanitas Clinical and Research Center - IRCCS, Milano, Italy, Department of Biomedical Sciences, Humanitas Universit, Milano, Italy; ^7^ Department of Surgical Science and Diagnostic Integrated, University of Genoa, Genova, Italy; ^8^ Department of Human Pathology of the Adult and Evolutive Age Gaetano Barresi, Division of Anesthesia and Intensive, Messina, Italy

###### **Correspondence:** L. La Via

*Journal of Anesthesia, Analgesia and Critical Care 2023,* **3(Suppl 1):**A62

Background

Variation of inferior vena cava (IVC) is used to predict fluid-responsiveness, but the IVC visualization with standard sagittal approach (SC, subcostal) cannot be always achieved. In such cases, coronal trans-hepatic (TH) window may offer an alternative, but the interchangeability of IVC measurements in SC and TH is not fully established [1, 2]. Further, artificial intelligence (AI) with automated border detection may be of clinical value but it needs validation.

Materials and Methods

We performed a prospective observational validation study in mechanically ventilated patients with pressure-controlled mode. Informed consent was obtained before the assessment. Primary outcome was the IVC distensibility (IVC-DI) in SC and TH imaging, with measurements taken both in M-Mode or with AI software (Figure 1).

We calculated mean bias, limits of agreement (LoA), and intra-class correlation (ICC) coefficient. The study was approved by local ethical committee on 21/03/2022 (Reference protocol: 53/2022/PO).

Results

Thirty-three patients were included. Feasibility rate was 87.9% and 81.8% for SC and TH visualization, respectively. Comparing imaging from the same anatomical site acquired with different modalities (M-Mode vs AI), we found the following IVC-DI differences: 1) SC: mean bias -3.1%, LoA [-20.1;13.9], ICC=0.65; 2) TH: mean bias -2.0%, LoA [-19.3;15.4], ICC=0.65. When comparing the results obtained from the same modality but from different sites (SC vs TH), IVC-DI differences were: 3) M-Mode: mean bias 1.1%, LoA [-6.9;9.1], ICC=0.54; 4) AI: mean bias 2.0%, LoA [-25.7;29.7], ICC=0.32 (Table 1).

Conclusions

In patients mechanically ventilated, AI software shows good accuracy (modest overestimation) and moderate correlation as compared to M-mode assessment of IVC-DI, both for SC and TH windows. However, precision seems suboptimal with wide LoA. The comparison of M-Mode or AI between different sites yields similar results but with weaker correlation.

Trial registration: Reference protocol: 53/2022/PO, approved on 21/03/2022

REFERENCES

1. La Via L, Astuto M, Dezio V, Muscarà L, Palella S, Zawadka M, Vignon P, Sanfilippo F, (2022) Agreement between subcostal and transhepatic longitudinal imaging of the inferior vena cava for the evaluation of fluid responsiveness: A systematic review. Journal of critical care 71: 154108
2. Sanfilippo F, La Via L, Dezio V, Santonocito C, Amelio P, Genoese G, Astuto M, Noto A, (2023) Assessment of the inferior vena cava collapsibility from subcostal and trans-hepatic imaging using both M-mode or artificial intelligence: a prospective study on healthy volunteers. Intensive care medicine experimental 11: 15

**Fig. 1 (abstract A62).**
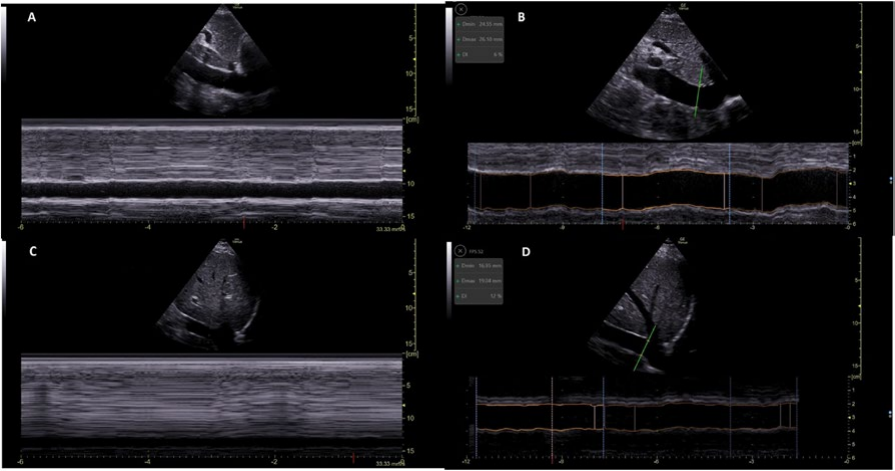
A: Subcostal view in M-mode; B: Subcostal view with AI; C: Trans-hepatic view in M-mode; D: Trans-hepatic view with AI

**Table 1 (abstract A62).** Summary of comparisons between measurement of the inferior vena cava (IVC) in adult patients mechanically ventilated in pressure control mode

Comparison		Variable	ICC	Mean Bias	Lower LOA	Upper LOA
			95%CI	95%CI	95% CI	95% CI
M-SC	**AI-SC**	IVC Min (mm)	0.79;	3.0;	-2.1;	8.1;
			-0.01 to 0.93	2.0 to 4.0	-3.8 to -0.3	6.3 to 9.8
		IVC Max (mm)	0.78;	2.9;	-2.3;	8.1;
			0.02 to 0.93	1.9 to 3.9	-4.1 to -0.5	6.4 to 9.9
		**IVC-DI (%)**	**0.65;**	**-3.1;**	**-20.1;**	**13.9;**
			**0.27 to 0.83**	**-6.4 to 0.3**	**-25.9 to -14.3**	**8.1 to 19.7**
M-TH	**AI-TH**	IVC Min (mm)	0.88;	2.4;	-0.7;	5.6;
			-0.06 to 0.96	1.8 to 3.1	-1.8 to 0.4	4.5 to 6.7
		IVC Max (mm)	0.85;	2.5;	-0.8;	5.9;
			-0.08 to 0.96	1.9 to 3.2	-2.0 to 0.3	4.7 to 7.1
		**IVC-DI (%)**	**0.65;**	**-2.0;**	**-19.3;**	**15.4;**
			**0.25 to 0.84**	**-5.5 to 1.5**	**-25.4 to 13.3**	**9.3 to 21.5**
M-SC	**M-TH**	IVC Min (mm)	0.74;	1.1;	-6.9;	9.1;
			0.41 to 0.88	-0.7 to 2.8	-9.9 to -4.0	6.1 to 12.0
		IVC Max (mm)	0.69;	1.2;	-7.0;	9.5;
			0.30 to 0.86	-0.5 to 3.0	-10.0 to -3.9	6.4 to 12.5
		**IVC-DI (%)**	**0.54;**	**0.1;**	**-19.0;**	**19.3;**
			**-0.09 to 0.80**	**-4.0 to 4.2**	**-26.2 to -11.9**	**12.1 to 26.4**
AI-SC	**AI-TH**	IVC Min (mm)	0.77;	0.4	-6.9;	7.8;
			0.46 to 0.90		-9.9 to -4.9	5.75 to 10.82
		IVC Max (mm)	0.76;	0.9	-6.0;	7.8;
			0.45 to 0.90		-8.9 to -4.1	5.9 to 10.7
		**IVC-DI (%)**	**0.32;**	**2.0**	**-25.7;**	**29.7;**
			**-0.63 to 0.72**		**-35.4 to -19.3**	**23.3 to 39.4**

### A63 Challenging surgery for aorta-to-right atrium fistula with torrential tricuspid valve regurgitation due to endocarditis

#### G. Gaudioso ^1^, A. Caruso ^2^, M. Mazzamuto ^3^, E. Panascia ^4^, S. Lentini ^3^, C. Santonocito ^4^

##### ^1^ School ofl Anesthesia and Intensive Care Unit, University Hospital Mater Domini , Magna Graecia, Catanzaro, Italy; ^2^ School of Anesthesia and Intesive Care A.O.U.Policlinico G. Rodorico- San Marco, Catania, Italy; ^3^ Division of Cardiovascular Surgery and Transplant Unit A.O.U. Policlinico G. Rodorico- San Marco, Catania, Italy; ^4^ Division of Anesthesia and Intensive Care Medicine III CAST -A.O.U. Policlinico G. Rodorico San Marco, Catania, Italy

###### **Correspondence:** G. Gaudioso

*Journal of Anesthesia, Analgesia and Critical Care 2023,* **3(Suppl 1):**A63

Background

Infective endocarditis (IE) remains a serious and even life-threatening disease with substantial morbidity and mortality. IE could have a lot of complications such as: central nervous system emboli, heart failure, abscess formation and systemic embolization. Moreover, the bacterial invasion at the level of periannular valves causes a suffering of tissue until necrosis occurs, and subsequent development of an intracardiac aorta-to-right atrium fistula (AAF) (1).

The case reports the clinical management and a no-previously reported surgical treatment of the AAF and the severe tricuspid valve insufficiency caused by IE.

Case report

This case report describes a 25-year-old man with IE, vegetation, perforation of noncoronary sinus with AAF with high flow left to right shunt and tricuspid valve regurgitation (TVR) first detected by transthoracic echography and after by transesophageal echography (TEE). We describe the clinical management and a no-previously reported surgical treatment of the AAF and the severe tricuspid valve insufficiency caused by IE. The patient was anesthetized with intravenous anesthesia. Cardiopulmonary bypass was started with selective double cava cannulation and return to the ascending aorta. After aortic cross clamp, a transverse aortotomy was performed. Myocardial protection was achieved and the aorta to right atrium fistula was found in the lower portion of the non-coronary Valsalva sinus and was closed with three interrupted U type prolene sutures passed over autologous pericardial patches. The tricuspid valve was severely damaged by the infective endocarditis, with lack of more than half of the anterior leaflet, that was reconstructed by the mean of a large autologous untreated pericardial patch followed by insertion of a single goretex chordae. At the end, a bicuspidalization was performed to improve the leaftes coaptation.

A last TEE was performed, before patient discharge, reporting a mild TVR and a minimal residual aortic-right atrium shunt. Patient is clinically improved post-operatively and laboratories data and radiological exams were normal.

Conclusion

AAF is a rare but very serious complication of IE and predicts a higher mortality. Patients who were treated conservatively had a higher mortality than those underwent surgery or percutaneous closure (53% vs. 12% vs. 3%, p0 <=0,00001). Thus, the in-hospital survival for percutaneous, surgical, and medically managed patients was 97%,88%, and 46% respectively (p=< 0,00001) (2). we reported this case presentation to describe for the first time the successfully surgical treatment with a never described surgical technic, combining the fistula closure and the tricuspid valve repair. This surgical approach, the contribution of Transesophageal echography to the diagnosis and follow-up together with antibiotic therapy could improve prognosis.

Written informed consent was obtained from the patient for publication of this case report and accompanying images.

References

1. Chen MY, Zhong DD, Ying ZQ. Aorta-to-right atrium fistula, an unusual complication of endocarditis. J Zhejiang Univ Sci B. 2009 Mar and 10(3):230-2.
2. Jainandunsing JS, Linnemann R, Bouma W, Natour N, Bidar E, Lorusso R, Gelsomino S, Johnson DM, Natour E. Aorto-atrial fistula formation and closure: a systematic review. J Thorac Dis. 2019 Mar and 11(3):1031-1046.

**Fig. 1 (abstract A63).**
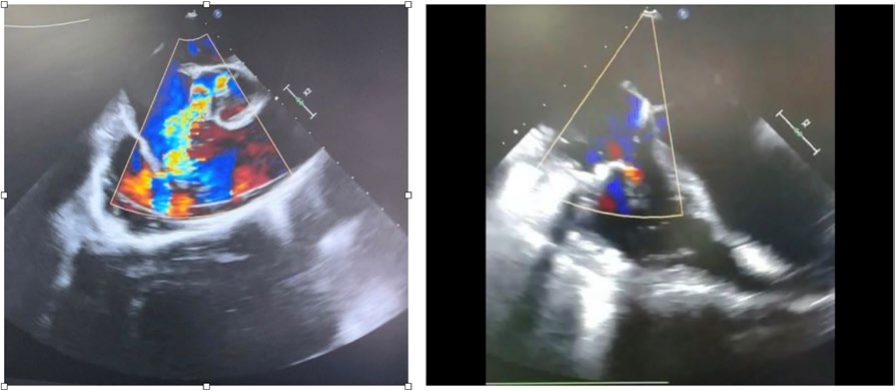
Preoperative and postoperative TEE

### A64 Intraoperative haemoadsorption with CytoSorb® in cardiac surgery

#### G. Ferrara ^1,2^, P. Raimondo ^1^, S. Lenoci ^1^, G. Rubino ^1^, M.A. Villani ^1^, A. Stripoli ^1^, A. Armenise ^1^, G. Colantuono ^1^, G. Fiore ^1^, M. Fiorentino ^3^, N. Di Venosa ^2^, S. Grasso ^1^

##### ^1^ Intensive Care Unit, Department of Emergency and Organ Transplantation (DETO), 'Aldo Moro' University, Bari, Italy; ^2^ Department of Anesthesia and Intensive Care Unit, 'L. Bonomo' Hospital, Andria, Italy; ^3^ Nephrology, Dialysis and Transplantation Unit, Department of Emergency and Organ Transplantation, 'Aldo Moro' University, Bari, Italy

###### **Correspondence:** G. Ferrara

*Journal of Anesthesia, Analgesia and Critical Care 2023,* **3(Suppl 1):**A64

Background

Cardiopathic patients are characterized by chronic inflammation. Cardiac surgery and cardiopulmonary bypass (CBP) cause hyperinflammation with postoperative multiorgan dysfunction and sepsis, which are more common in this surgical setting. The CytoSorb adsorber can bind to a wide range of inflammatory mediators and molecules and the removal of these substances is concentration-dependent. Therefore, the efficiency of cytokines removal rises when their level increases.

Materials and methods

After informed consent, we enrolled 18 patients who underwent cardiac surgery at Cardiac Surgery Department of ‘Policlinico di Bari’ from January 2020 to August 2022. Among these patients only 9 of them received intraoperative haemoadsorption with CytoSorb during CPB. The two groups (CPB with haemoadsorption or CytoSorb group and CPB without haemoadsorption or control group) were homogeneous in age, medical history and kind of surgery. We analyzed these perioperative data: inflammation markers, platelet count, serum lactate, length of stay and outcome.

Results

Inflammation markers (C-reactive protein, procalcitonin and presepsin), platelet count at admission and discharge, serum lactate levels, length of stay and outcome were comparable between the two groups, with no statistically significant difference. In patients who did not receive heart transplantation (4 in CytoSorb group and 4 in control group), the increase of mean value of C-reactive protein (CRP) from the early postoperative phase to the first postoperative day (deltaCRP) is lower in the CytoSorb group compared to the control one and this difference reached statistically significance (p Value 0,023).

Conclusions

This study confirms what the most recent literature reports [1]. Although there is not a statistically significant difference between the two groups in examined data, the statically significance of deltaCRP between the two groups related to the not heart transplanted patients has a great interest. CytoSorb provides a concentration-dependent removal. Hence, the highest removal efficiency is given at the highest concentration of cytokines. This explains our finding in deltaCRP in not transplanted patients who are characterized by a perioperative systemic hyperinflammation in contrast to transplanted patients in which systemic hyperinflammation is lowered by immunosuppressive therapies. Consequently, the use of CytoSorb in cardiac surgery might prevent or significantly reduce systemic inflammation due to CBP, thanks to its broad adsorption spectrum. Further studies on a larger number of patients are needed to better evaluate CytoSorb efficiency in preventing complications of sepsis in critical patients. Although our results are not statistically significant, they might suggest an interesting field of application of CytoSorb in organ perfusion during cardiac transplantation.

References

1. Diab M, Lehmann T, Bothe W, et al. Cytokine Hemoadsorption During Cardiac Surgery Versus Standard Surgical Care for Infective Endocarditis (REMOVE): Results from a Multicenter Randomized Controlled Trial. Circulation. 2022 Mar 29;145(13):959-968.

### A65 No EEG burst suppression by general anaesthesia during carotid body paraganglioma surgical excision: a case report

#### S. Di Franco, R. Giampieri, D. Smaldone, E. Barbato, E. Barone, R. Compagna, M. Boschetti, G. Barba, A. Pucciarelli, S. De Vivo, C. Fittipaldi, L. Durante

##### P.O. Pellegrini Als Napoli 1 Centro, Napoli, Italy

###### **Correspondence:** S. Di Franco

*Journal of Anesthesia, Analgesia and Critical Care 2023,* **3(Suppl 1):**A65

Background

Carotid body paraganglioma (CBP) is a rare non-chromaffin neoplasm with an incidence of 1-2 per 100,000 population. The finding of this neoplasm is greater in women and the 5-7% are revealed to be malignant [1]. The anaesthetic management during surgical removal is complex and aimed at managing hemodynamic complications due to tumour manipulation, secretion of catecholamines and surgical injury of cranial nerves. The main objective of anaesthesia is to ensure hemodynamic stability, optimise cerebral perfusion and facilitate surgeon's work.

Case report

A 69-year-old male patient was hospitalised in March 2023 with a diagnosis of left CBP (Shamblin classification 2), history of hypertension, smoke (about 20 sigs/day) and COPD. At the time of admission, a total body TC angiography was performed which showed the left CBP measuring 26x19x31 mm (Figure 1).

After an initial failure in embolization of the branches supplying the tumour, open surgery was planned by a multidisciplinary team by evaluating general anaesthesia with a reduced intake of hypnosedative drugs and opioids in association with ultrasound-guided superficial cervical plexus block. In the operating room, continuous EEG monitoring, Bispectral index (BIS) monitoring, invasive arterial pressure monitoring, SpO2, heart rate, body temperature, diuresis were guaranteed. Induction of anaesthesia was achieved with intravenous administration of midazolam 0.01mg/kg, fentanyl 1.5mcg/kg, atropine 0.1mg/kg and ketamine 0.7mg/kg. Mio-resolution was obtained with 0.6mg/kg of rocuronium. Anesthesiological maintenance was ensured by continuous infusion of ketamine at 0.35mg/kg/h and sevoflurane with a MAC=0.5. Following orotracheal intubation with a 7.5 mm diameter tube, the block of the superficial cervical plexus was performed with ultrasound guidance. Ropivacaine 0.5% (15ml) was injected in combination with dexamethasone 4mg as an adjuvant. The intervention lasted 180 minutes. There were no drug-induced EEG changes or burst suppression with BIS values between 55 and 60. No hemodynamically relevant changes were detected. It was not necessary to administer vasopressors. Post-operative pain was absent with NRS=0 at T0 (1 hour post surgery) and NRS=3 at 12 hours (post surgery). The patient was discharged within two days post surgery without complications. All the data presented were collected after acquiring the patient's written consent and in accordance with the Declaration of Helsinki.

Conclusion

Loco regional anaesthesia for intraoperative and post-operative pain management and ketamine continuous infusion acting on NMDA receptors may offer a great hemodynamic support in anesthesiological management of surgical removal of CBP not causing burst suppression in EEG monitoring in continous the cerebral activity douring the surgical phases and detecting promptly brain damages related to the surgical procedure.

Reference

1. Ng DW, Yam CI, Wong LT, Koh DL. An anaesthesia perspective on carotid body tumour (CBT) excision: A twenty-year case series at the Singapore General Hospital. J Perioper Pract. 2017;27(10):228-233

**Fig. 1 (abstract A65).**
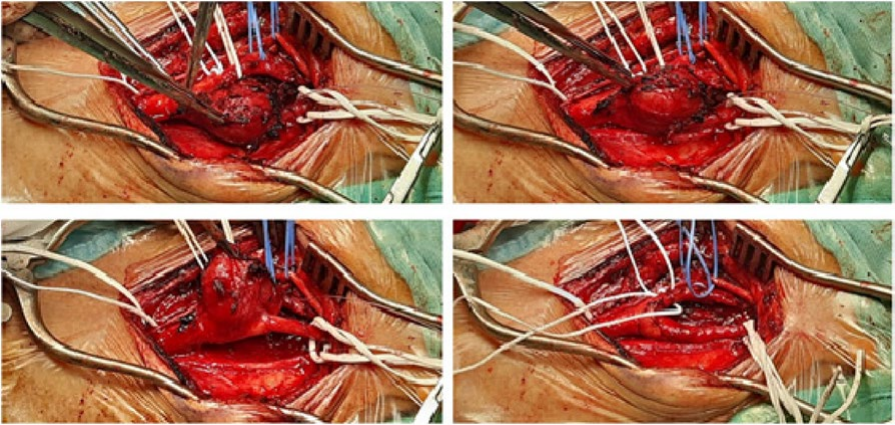
See text for description

### A66 Erector spine plane block versus intravenous post-operative analgesia for pain control after adult minimally invasive cardiac surgery

#### R. Benedetto ^1^, P. Dambruoso ^2^, N. Ceglie ^2^, F. Massaro ^3^, P. Raimondo ^1^, S. Grasso ^1^

##### ^1^ Department of Precision and Regenerative Medicine and Jonica Area, Anesthesia and Intensive Care II, Policlinico di Bari, Bari, Italy; ^2^ Cardiac Anesthesia and Postoperative Intensive Care, Santa Maria Hospital, GVM Care and Research, Bari, Italy; ^3^ Perioperative Medicine, Department of Emergency and Urgency, Regional Hospital F. Miulli, Acquaviva delle Fonti, Italy

###### **Correspondence:** R. Benedetto

*Journal of Anesthesia, Analgesia and Critical Care 2023,* **3(Suppl 1):**A66

Background

Adequate post-operative analgesia also after minimally invasive cardiac surgery is fundamental to reduce post-operative pain and associated complications like hemodynamic instability, arrhythmias, late ventilator weaning, pulmonary infections, and long-lasting hospital stay.

Among the myofascial blocks, the ESP-block promises to achieve an effective thoracic pain control – compared with post-operative systemic use of opioids – through the deposit of local anesthetic in myofascial plane between erector spinae muscle and thoracic transverse processes (Fig. 1; informed consent to publish data and images had been obtained for all patient).

This study compares ESP-block versus post-operative intravenous analgesia to control pain more effectively after minimally invasive cardiac surgery.

Materials and methods

Twenty patients of either sex, undergoing elective minimally invasive cardiac surgery (coronary artery bypass or valve repair/replacement) receiving the same pharmacological treatment intraoperatively, were randomly assigned to Group A (ESP-Block) or Group B (post-operative intravenous analgesia).

Group A received mono-lateral, single shot ESP block, using ropivacaine 0.375% plus dexamethasone 4 mg before anesthesia induction between T5-T6 transverse processes (Fig. 2) while Group B received post-operative intravenous analgesia (tramadol 400 mg/24 hours).

The primary goal of the study was to evaluate differences between ESP-block group and intravenous analgesia group in post-operative pain control at rest and during motion using Numerical Rating Scale (NRS) after minimally invasive cardiac surgery.

Statistical analysis was performed using the independent Student’s T test considering P<0.05 as statistically significant.

Results

NRS score was evaluated considering 0, 3, 6, 9, 12, 24 hours after extubation in both groups at rest and during motion (Table 1-2).

The mean NRS score resulted smaller in Group A and was lower than 4 in both groups (except for the 9th hour post extubation in Group A and 6th hour in Group B). Nevertheless, the NRS score resulted statistically significant only from 6th to 12th hour after extubation, during which ESP block resulted more effective than intravenous analgesia.

In the previous (from 0 to 6th hour) and subsequent hours (from 12th to 24th hour), lower NRS means were registered for Group A even if with no statistical significance.

Furthermore, the first rescue analgesia was requested after 9 hours in Group A and 6 hours in group B.

Conclusion

The ESP block is easy to perform and provide an immediate superior pain control in comparison with intravenous post-operative analgesia and allows to reduce post-operative opioid consumption and possible related complications after minimally invasive cardiac surgery.

**Fig. 1 (abstract A66).**
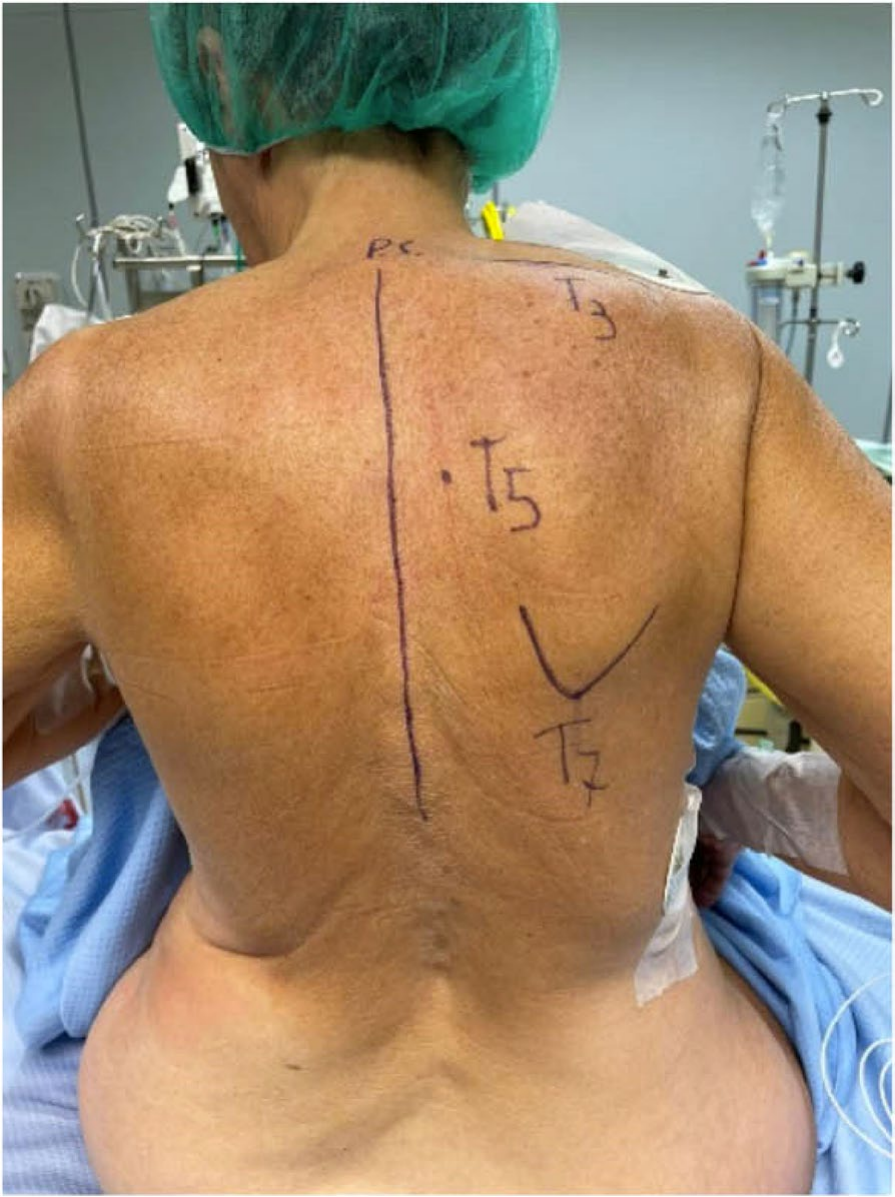
Anatomical landmarks to perform ESP-Block

**Fig. 2 (abstract A66).**
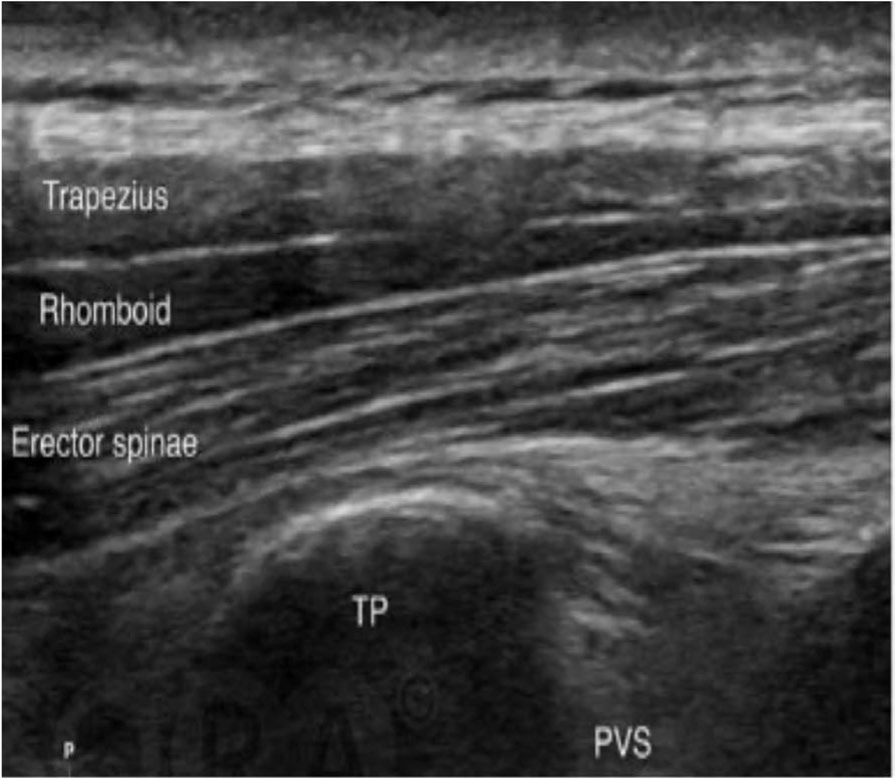
Sonographic anatomy of muscles: trapezius, rhomboid and erector spinae, with transverse process (TP) and paravertebral space (PVS)

**Table 1 (abstract A66).** Numeric Rating Scale comparison between two groups at rest with p-value (T-student test)

	ESP BLOCK	INTRAVENOUS	
		ANALGESIA	
	Mean ± DS	Mean ± DS	p-value
	At rest	At rest	
**Post-extubation**	1.8 ± 1.03	2.6 ± 0.96	0.09
**3 hrs later**	2.3 ± 0.9	3 ±0.81	0.09
**6 hrs later**	2.4 ± 1.77	4.5 ± 2.17	0.029
**9 hrs later**	4.2 ± 2.39	1.9 ± 1.7	0.025
**12 hrs later**	3.2 ± 2.4	0.8 ± 0.13	0.014
**24 hrs later**	0.2 ± 0.4	0.4 ± 0.7	0.45

**Table 2 (abstract A66).** Numeric Rating Scale comparison between two groups during motion with p-value (T-student test)

	ESP BLOCK	INTRAVENOUS	
		ANALGESIA	
	Mean ± DS	Mean ± DS	p-value
	During motion	During motion	
**Post-extubation**	2.5 ± 1.5	3.3 ± 1.15	0.214
**3 hrs later**	2.3 ± 0.94	3.4 ± 1.24	0.09
**6 hrs later**	2.4 ± 1.77	4.5 ± 2.17	0.029
**9 hrs later**	4.4 ± 2.5	1.9 ± 1.7	0.020
**12 hrs later**	2.8 ± 2.14	0.8 ± 0.13	0.021
**24 hrs later**	0.2 ± 0.4	0.4 ± 0.7	0.45

### A67 Coronary artery bypass graft in a patient with Rendu-Osler-Weber Syndrome - A CASE REPORT -

#### A. Caruso ^1^, E. Panascia ^2^, V. Scuderi ^2^, M.M. Giambra ^3^, S. Lentini ^4^, C. Santonocito ^2^

##### ^1^ School of Anesthesia and Intensive Care A.O.U. Policlinico G. Rodolico - San Marco, Catania, Italy, ^2^ Division of Anaesthesia and Intensive Care Medicine III CAST - A.O.U. Policlinico G. Rodolico - San Marco, Catania, Italy, ^3^ School of Vascular Surgery, Division of Vascular surgery A.O.U. Policlinico G. Rodolico - San Marco, Catania, Italy, ^4^ Division of Cardiovascular Surgery and Organ Transplant Unit A.O.U. Policlinico G. Rodolico - San Marco, Catania, Italy

###### **Correspondence:** A. Caruso

*Journal of Anesthesia, Analgesia and Critical Care 2023,* **3(Suppl 1):**A67

Background

Rendu-Osler-Weber syndrome or Hereditary hemorrhagic telangiectasia (HHT) is an autosomal dominant genetic disorder determining alterations in vascular structures. Clinical features are represented typically by recurrent epistaxis and telangiectasis of the nasal and buccal mucosa, tongue, and lips. Arteriovenous malformations (AVMs) could be localized in the cerebral, pulmonary, gastrointestinal, and hepatic circulations leading to severe complications (1). Coagulative disorders and acquired von Willebrand disease could be present and related with the recurrent and chronic bleeding but also with aortic valve stenosis and AVMs. High cardiac output heart failure can manifest due to the combination of large pulmonary AVMs and/or hepatic AVMs and chronic anemia (2). Given the high hemorrhagic risk as perioperative complication in cardiothoracic surgery, this kind of procedures in HHT patients could represent a difficult challenge.

The case report has been managed at a University Hospital and this work has been reported in line with SCARE 2020 criteria (3).

Case report

A 71-year-old male with HHT came to hospital for chest pain and the ECG showed NSTEMI. Coronorographic studies revealed critical stenosis of left anterior descending artery and Diagonal artery for which surgical procedure was required. The patient presented active nasal bleeding the day of the procedure treated with nasal packing prior to surgery. Given the high hemorrhagic risk, prevention of coagulation disorders after CPB was a priority. On pump CABG x two was performed and the careful management with viscoelastic tests combined with the goal-directed transfusions lead to a good outcome and both the surgery and the postoperative period in ICU were uneventful. Further hospitalization was complicated by lung infection, which was treated with antibiotic therapy and the patient was discharged from the hospital after fourty days.

Conclusion

HHT is a condition related to higher postoperative bleeding risk after cardiac surgery. Management of these patients should be done with a multidisciplinary team discussion and planning to opimise patient’s conditions prior to surgery. Diagnostic tools like CT scan it is suggested to investigate on the presence of AVMs.. During surgery it is fundamental to be guided by point of care systems like viscoelastic tests (ROTEM) to reduce the risk of postoperative bleeding.

Written informed consent was obtained from the patient for publication of this case report and accompanying images. A copy of the written consent is available for review by the Editor-in-Chief of this journal on request.

Reference

1. Sabbà, C. A rare and misdiagnosed bleeding disorder: hereditary hemorrhagic telangiectasia. 2005 Oct;3(10):2201-10. : J Thromb Haemost. Epub 2005 May 9. .
2. Kritharis A, Al-Samkari H, Kuter DJ. Hereditary hemorrhagic telangiectasia: diagnosis and management from the hematologist's perspective. s.l. : Haematologica. 2018 Sep;103(9):1433-1443.
3. Agha RA, Franchi T, Sohrabi C, Mathew G, Kerwan A, Dec, SCARE Group. The SCARE 2020 Guideline: Updating Consensus Surgical CAse REport (SCARE) Guidelines. Int J Surg. 2020 and 33181358., 84:226-230.

### A68 Dynamic Arterial Elastance and pulse wave analysis: does exist a mathematical coupling?

#### M. Magrelli ^1^, L. Cardia ^1^, O. Mandraffino ^1^, G. Genoese ^2^, F. Albanese ^2^, R. De Luca ^2^, P. Calì ^2^, M. Giardina ^2^, A. Noto ^1^

##### ^1^ Division of Anesthesia and Critical Care Department of Human Pathology of the adult and evolutive age Gaetano Barresi, Messina, Italy; ^2^ Division of Anesthesia and Critical Care, A. O. U. Policlinico G. Martino, Messina, Italy

###### **Correspondence:** M. Magrelli

*Journal of Anesthesia, Analgesia and Critical Care 2023,* **3(Suppl 1):**A68

Background

The prediction of the increase of the stroke volume (SV) after volume expansion (preload dependency) has been widely studied, nevertheless, it is demonstrated that an increase in cardiac output will not always produce an increase in mean arterial pressure (MAP) [1].

Dynamic arterial elastance (Eadyn), stated as the ratio between Pulse Pressure Variation (PPV) and Stroke Volume Variation (SVV), was proposed as a marker of ventricular-arterial coupling [2] and could be employed to predict the response of mean arterial pressure to volume expansion in hypotensive and preload-dependent patients. A mathematical coupling between PPV and SVV was postulated when the measure of the two variables comes from the same origin (pulse wave analysis), the present study aimed to test the lack of this autocorrelation in patients undergoing vascular surgery.

Materials and methods

A prospective observational study on fluid-responder patients undergone aortic surgery was conducted; in accordance with the Declaration of Helsinki and approved by the “Comitato Etico Interaziendale della Provincia di Messina” (PN125-21-29/09/2021). Written informed consent was obtained from all participants before enrollment. Hemodynamic parameters were simultaneously recorded from pulse wave analysis (FloTrac Sensor, Edwards Lifesciences) and bioreactance (NICOM, Cheetah Medical). EaDyn values were calculated using PPV from the arterial line and SVV from the arterial line (FloTrac) or from bioreactance (NICOM). The Bland-Altmann plot and non-parametric statistical test were used to evaluate any difference between EaDyn.

Results

21 patients were recruited; one patient was excluded because of aortic stenosis. 42 fluid challenges have been carried out in 20 patients; 25 of these fluid tests showed a preload dependency, and, in 14 of these, the increase of MAP was observed (table 1).

The mean EaDyn calculated from FloTrac only and the EaDyn from NICOM didn’t show any statistical difference (0.83±0.37 vs 0.75±0.33 p=0.16) (figure 1).

The Bland and Altmann plot between the two methods showed a mean bias of 0.09 and the limits of agreements between 0.50 and 0.69 (figure 2).

The predictive significance of Eadyn is preserved in both measurement systems: in MAP Responders is always higher than 0.8, which is considered a cut-off of optimal ventricular-arterial coupling [3] (figure 3).

Conclusions

In the computing of Eadyn, mathematical coupling between PPV and SVV detected from the same arterial wave signal can be probably ruled out: the value of Eadyn preserved its ability to predict MAP increasing after fluid challenge. Therefore, Dynamic Arterial Elastance can be easily calculated in the operating room from only an arterial signal.

References

1. Monge García MI, Barrasa González H. Why did arterial pressure not increase after fluid administration? Med Intensiva [Internet]. 2017;41(9):546–9. Available from: http://dx.doi.org/10.1016/j.medin.2017.03.005
2. Lanchon R, Nouette-Gaulain K, Stecken L, Sesay M, Lefrant J-Y, Biais M. Dynamic arterial elastance obtained using arterial signal does not predict an increase in arterial pressure after a volume expansion in the operating room. Anaesth Crit Care Pain Med [Internet]. 2017;36(6):377–82. Available from: http://dx.doi.org/10.1016/j.accpm.2017.05.001
3. Monge García MI, Gil Cano A, Gracia Romero M. Dynamic arterial elastance to predict arterial pressure response to volume loading in preload-dependent patients. Crit Care [Internet]. 2011;15(1):R15. Available from: 10.1186/cc9420

**Table 1 (abstract A68).** Fluid-responders patients characteristics.

**Number of Patients**	**17**
**Sex**	**16M/1F**
**Mean age (SD)**	**70.4 (7.4)**
**Hypertension (n. of cases and %)**	**7 (41.1%)**
**Dyslipidemia(n. of cases and %)**	**8 (47%)**
**Diabetes Mellitus (n. of cases and %)**	**7 (7.4%)**
**Kidney failure (n. of cases and %)**	**2 (11.7%)**
**Inotropic, vasodilators, beta-blockers agents therapy (n. of cases and %)**	**7 (7.4%)**
**Atrial fibrillation (n. of cases and %)**	**2 (11.7%)**

**Fig. 1 (abstract A68).**
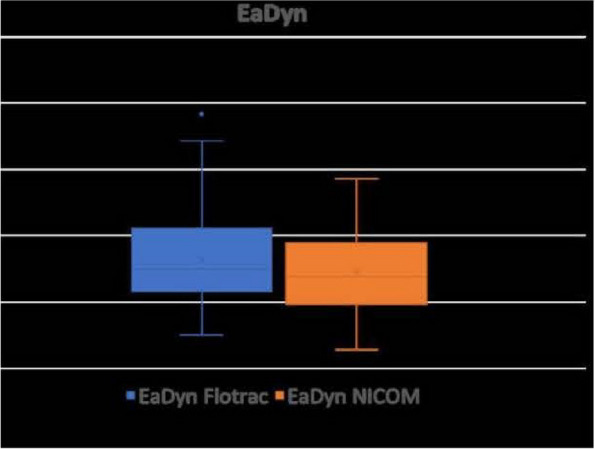
BoxPlot chart. In blue the distribution of Dynamic Arterial values in which PPV and SVV are detected by FloTrac, in orange the distribution of Dynamic Arterial values in which PPV is monitored by FloTrac and SVV is detected with Cheetah Medical. Median values and 1^st^ and 3^rd^ percentile

**Fig. 2 (abstract A68).**
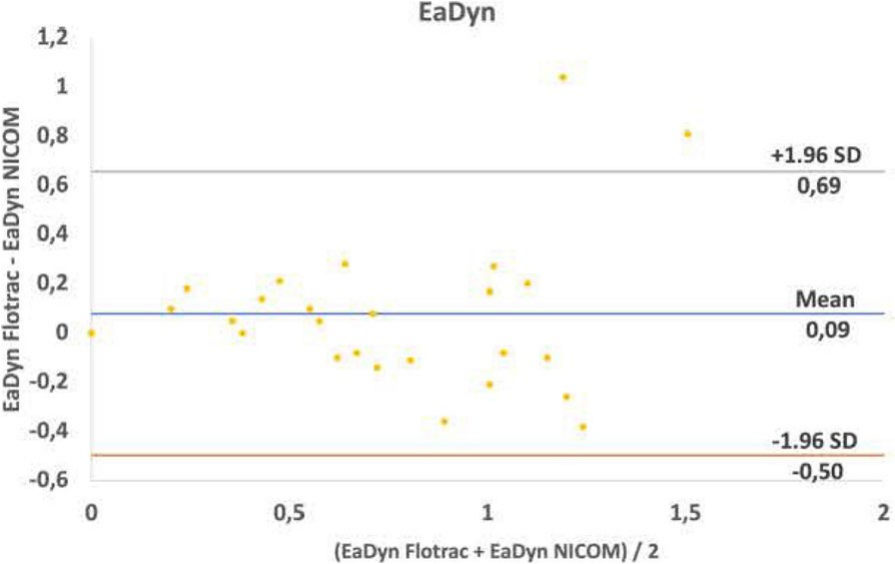
Bland and Altmann Plot: Eadyn values are reported as points whose coordinates are the mean value between EaDyn FloTrac and Eadyn Cheetah at the x-axis and the value of the difference between EaDyn FloTrac and Eadyn Cheetah at the ordinates. Values are expressed in mean and standard deviation

**Fig. 3 (abstract A68).**
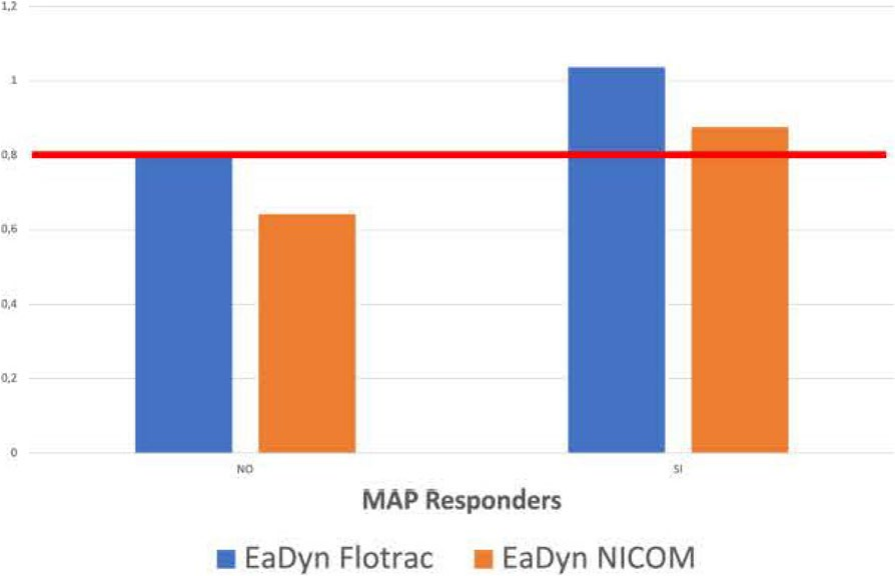
In blue the distributions of Eadyn values in which PPV and SVV are monitored with FloTrac, in orange the distribution of Eadyn values in which PPV and SVV are detected by different wave lines. The red lines represent the commonly accepted lower bound of Eadyn

### A69 CytoSorb hemoadsorption of rivaroxaban during open heart operations in children: a case report

#### A. Barbaria ^1^, S. Todaro ^2^, K. Aiouaz ^3^, M. Cotza ^3^, A. Giamberti ^4^, M. Reali ^4^, M. Ranucci ^3^, U. Di Dedda ^3^, T. Aloisio ^3^

##### ^1^ Anesthesia and Critical Care, Department of Surgical, Pediatric and Diagnostic Sciences, University of Pavia, Pavia, Italy; ^2^ Anesthesia and Critical Care, Department of Pathophysiology and Transplantation, University of Milan, Milano, Italy; ^3^ Department of Cardiothoracic-Vascular Anesthesia and Intensive Care, IRCCS Policlinico San Donato, San Donato Milanese (Milano), Italy; ^4^ Department of Congenital Cardiac Surgery, IRCCS Policlinico San Donato, San Donato Milanese (Milano), Italy

###### **Correspondence:** A. Barbaria

*Journal of Anesthesia, Analgesia and Critical Care 2023,* **3(Suppl 1):**A69

Background

The management of hemostasis during emergency cardiac surgery could be an operative challenge because of the higher risk of bleeding due to the assumption of DOAC [1]. Recently, extracorporeal hemoadsorption of DOAC with CytoSorb has been described as an effective method to reduce perioperative bleeding.[2]

Case report

We herein report a case of a 6-year-old boy (weight 20 kg) with a past diagnosis of osteogenic sarcoma of the left humerus, treated with surgery and chemotherapy. During the follow-up he had the complication of a deep venous thrombosis (DVT) of the left innominate, subclavian and axillary veins. At first LMWH was introduced (Clexane 2000 IU bid), then it was switched to rivaroxaban 5 mg bid. Three days later, the child was admitted to the pediatrics department with fever, high CRP and thrombocytopenia. An echocardiography found a thrombus in the right atrium reaching up to the tricuspid valve. A CT scan was performed showing the extension of the DVT from the left common jugular to the left atrium (Figure 1).

For this reason, the child was transferred at the department of Pediatrics Intensive Care Unit (IRCCS Policlinico San Donato). At the admission a new echocardiography showed that the thrombus had extended throughout the tricuspid valve into the left ventricle. Laboratory tests reported a significant thrombocytopenia (platelet count 32.000/uL), an aPTT of 46,1s and a rivaroxaban anti-Xa level of 30 ng/mL. After a multidisciplinary discussion, the decision to wait until 24 hours from the last somministration of rivaroxaban was taken. The morning after, the child underwent an open-chest intracardiac thrombectomy. The decision to mount a CytoSorb haemoadsorber into the cardiopulmonary bypass (CBP) circuit was taken, with the aim of removing active molecules of rivaroxaban during the CPB time. The treatment was associated with a good control of peri-operative bleeding. A single unit of platelets was administered. The point-of-care viscoelastic tests after CPB were normal, with no other treatment needed.

In the following days, no bleeding complications were reported. After 3 days, anticoagulant enoxaparin was introduced. A week later, the child was dismissed from the hospital in good physical conditions.

Conclusion

This case provides an example of the utility of CytoSorb hemoadsorption in an emergency pediatric clinical setting. Even though several case reports have been published [5], further research is needed to validate this technique on a larger scale in a randomized controlled fashion.

Consent to publication

Informed consent to publish had been obtained

References

1. Milling TJ Jr. A review of oral anticoagulants, old and new, in major bleeding and the need for urgent surgery. Trends Cardiovasc Med. 2020 Feb;30(2):86-90. doi:10.1016/j.tcm.2019.03.004. Epub 2019 Mar 26. PMID:30952383;PMCID:PMC6763385.
2. Hassan K. Cytosorb Adsorption During Emergency Cardiac Operations in Patients at High Risk of Bleeding. Ann Thorac Surg. 2019 Jul;108(1):45-51. doi:10.1016/j.athoracsur.2018.12.032. Epub 2019 Jan23. PMID: 30684482.
3. Buonocore M. CytoSorb haemoadsorption for removal of apixaban-A proof-of-concept pilot case for a randomized controlled trial. J Clin Pharm Ther. 2022 Dec;47(12):2373-2375. doi: 10.1111/jcpt.13802. Epub 2022 Nov 9. PMID:36351749.

**Fig. 1 (abstract A69).**
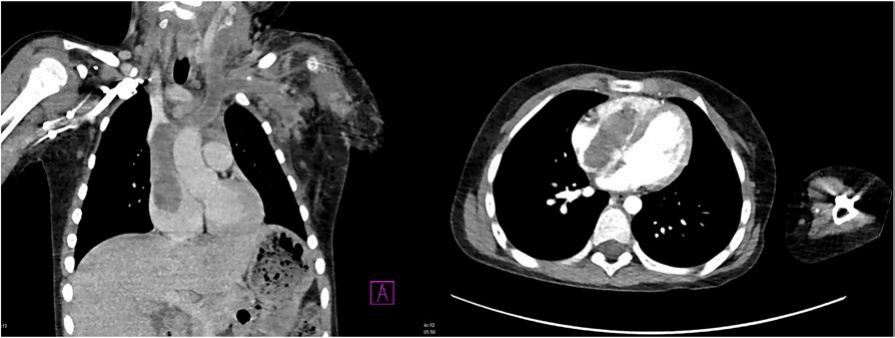
Pre-operative CT scan

## Vascular Anesthesia

### **A70 Veno-venous extracorporeal membrane oxygenation for pyopneumothorax by Strep. Constellatus: a case report**

#### P. Balagna ^1, 3^, A. Cardinale ^1^, N. D'Ettore ^1^, G. Maj ^1^, E. Vokrri ^2^, S. Meda ^2^, F. Pappalardo ^1^

##### ^1^ Cardiothoracic and Vascular Anesthesia and Intensive Care, Azienda Ospedaliera Santi Antonio e Biagio e Cesare Arrigo, Alessandria, Italy; ^2^ Division of Thoracic Surgery, Santi Antonio e Biagio e Cesare Arrigo Hospital, Alessandria, Italy; ^3^ Department of Translational Medicine, University of Eastern Piedmont, Novara, Italy

###### **Correspondence:** P. Balagna

*Journal of Anesthesia, Analgesia and Critical Care 2023,* **3(Suppl 1):**A70

Background

Pleural empyema is a collection of pus in the pleural cavity, most commonly secondary to bacterial pneumonia. Pathogens usually involved are Streptococci if community-acquired, methicillin-resistant Staphylococcus aureus (MRSA), and Pseudomonas if hospital-acquired^1^. It is associated with elevated morbidity and mortality as it can exacerbate acute respiratory distress syndrome. Its pathological features with huge airspace cavities filled with pus make mechanical ventilation challenging as barotrauma might portend lethal complications: VV ECMO might apply in this setting^2^.

Case report

A 65-year-old man presented to the ED of a peripheral hospital with dyspnea, elevated infection markers (WBC 38000/ul, PCR 50 mg/dL, PCT 9 ng/mL), and severe hypoxemia (PaO2/FiO2 80). The CT scan showed centrilobular emphysema with massive hydropneumothorax of the right lung (Fig. 1).

Broad-spectrum antibiotics and non-invasive ventilation had been immediately started but with little benefit and the patient was referred to our Center. Upon admission, the patient was severely dyspnoic and hypoxemic: intubation had not been performed envisioning the potential risks associated with rupture of the airspace cavity. The patient was intubated and a chest tube was placed with initial drainage of approximately 1000 ml of purulent fluid. Despite optimization of mechanical ventilation, gas exchange remained poor with associated hemodynamic instability, and we decided to implant VV extracorporeal membrane oxygenation DualLumenCannula (Avalon 27Fr) in the right internal jugular vein under transesophageal echocardiography guidance (Fig. 2).

The initial setting of the VV ECMO was 2800 rpm, 2.8 L/min, and FiO2 100%-FGF 2 L/min. the patient was immediately started on PSV 10/5/0.4 and sedation was stopped. After two days, the plural fluid was contaminated by Streptococcus constellatus sensible to penicillin. On day 6, we gradually reduced ECMO flow and FiO2 until its removal on day 9. The CT scan showed persistent consolidation of the right lower lobe with clear pleural space and no intraparenchymal cavitation (Fig. 3).

The patient appeared was transferred to another ICU for respiratory physiotherapy.

Conclusion

Pleural empyema due to Strep. Constellatus has unique pathological features (huge cavitation occurring in pats with severe baseline pulmonary disorders) and can lead to severe respiratory failure. VV ECMO, in this light, is a valuable option to treat these patients.

Informed consent to publish had been obtained.

References

1. Dyrhovden R, Nygaard RM, Patel R, Ulvestad E, Kommedal Ø. The bacterial aetiology of pleural empyema. A descriptive and comparative metagenomic study. Clin Microbiol Infect. 2019 Aug;25(8):981-986. doi: 10.1016/j.cmi.2018.11.030. Epub 2018 Dec 21. PMID: 30580031.
2. Combes A, Peek GJ, Hajage D, Hardy P, Abrams D, Schmidt M, Dechartres A, Elbourne D. ECMO for severe ARDS: systematic review and individual patient data meta-analysis. Intensive Care Med. 2020 Nov;46(11):2048-2057. doi: 10.1007/s00134-020-06248-3. Epub 2020 Oct 6. PMID: 33021684; PMCID: PMC7537368.^2^

**Fig. 1 (abstract A70).**
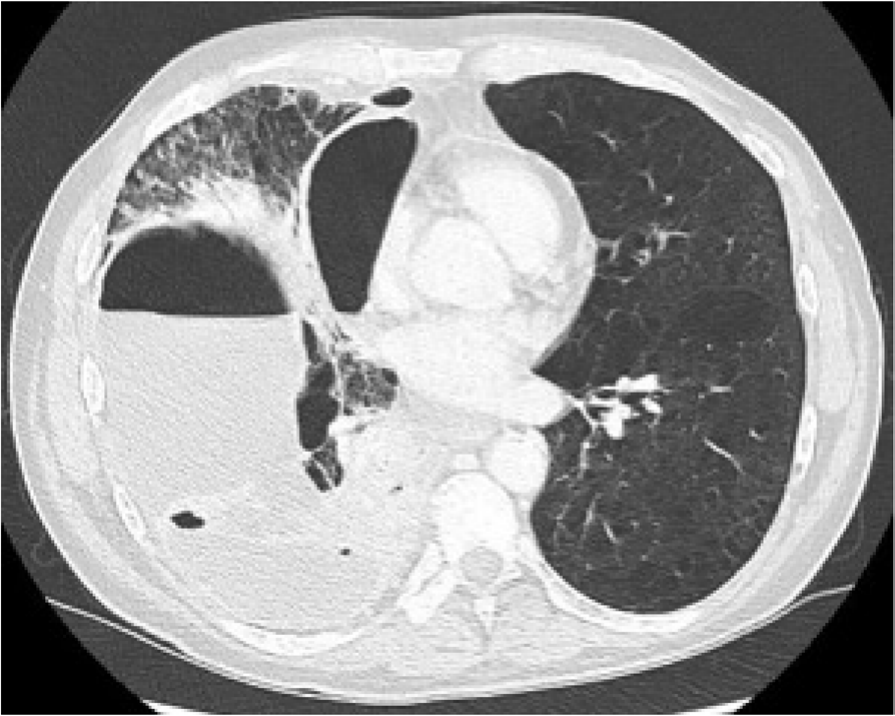
Axial CT image shows hydropneumothorax of the right lung

**Fig. 2 (abstract A70).**
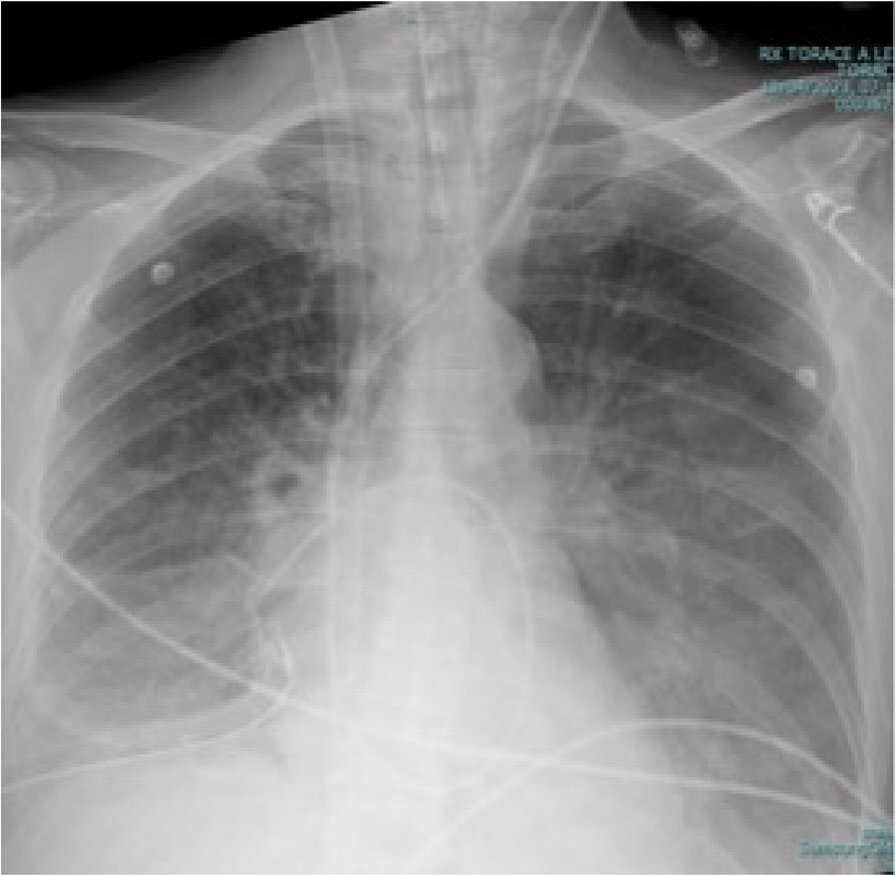
Anteroposterior chest radiograph taken at day 1 after VV ECMO, chest tube, ET tube, CVC and Swan-Ganz catheter placement

**Fig. 3 (abstract A70).**
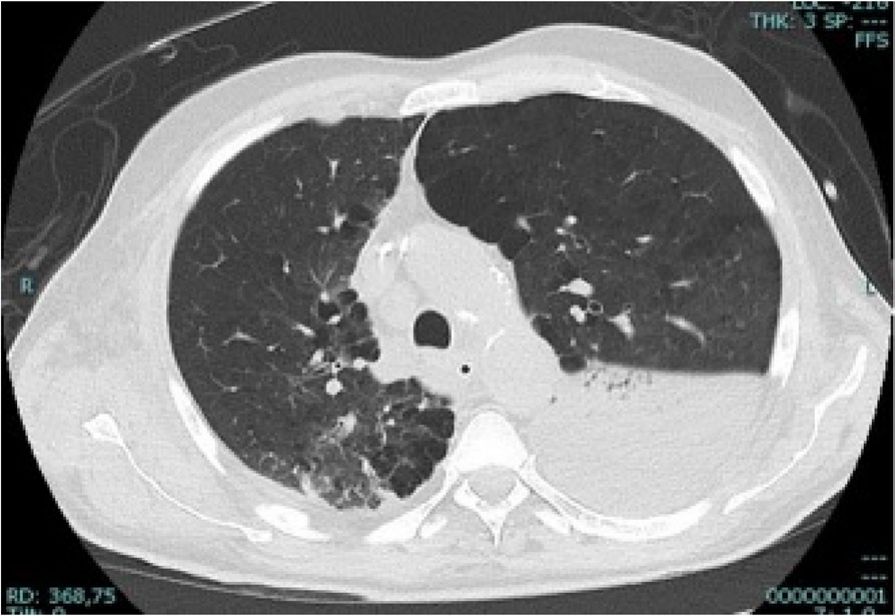
Axial CT image shows resolution of the hydropneumothorax of the right lung

### A71 Rate control in NSTEMI and sepsis scenario: a case report

#### F.I. Adamo, G. Scoccia, G. Manzi, G. Di Pietro, R. Improta, A. Ciuffreda, D. Angotti, S. Valentini, M. Mancone, C.D. Vizza, R. Badagliacca

##### Department of internal medicine, anesthesiological and cardiovascular sciences, La Sapienza University, Rome, Italy

###### **Correspondence:** F.I. Adamo

*Journal of Anesthesia, Analgesia and Critical Care 2023,* **3(Suppl 1):**A71

Background

Tachyarrhythmias are a common feature in NSTEMI patients admitted to the intensive care unit. During hospitalization some patients may experience an infectious complication. In the setting of possible sepsis an ultra-selective beta-blocker therapy is of the utmost importance to ensure valid hemodynamics, since it avoids further vasodilation having no beta2 effects. Landiolol is an intravenous ultra short-acting beta1-blocker. Its 1-minute onset of action determines a rapid heart rate control, and half-life of 4 minutes allows an equally rapid recovery if side effects occur [1]. On the other side its highly cardioselectivity (beta1/beta2 ratio of 255) results in a minor effect on blood pressure compared to other beta-blockers, which is particularly useful in settings where the risk of hypotension is increased. In fact, several observational studies reported the efficacy of landiolol to manage and prevent both perioperative and sepsis-related tachyarrhythmias [2,3].

Case report

A 77-years-old patient was admitted to the emergency department because of weakness and palpitation. On admission to the hospital patient was unrensponsive. Measured blood pressure was 80/40 mmHg. The electrocardiogram showed ventricular tachycardia at 150 beats per minute. Considering the hemodynamic instability, defibrillation was performed with restoration of sinus rhythm and of normal blood pressure values. Since the blood gas analysis showed metabolic acidosis with hyperlactacidemia and respiratory failure, patient was intubated. The electrocardiogram after defibrillation documented ST elevation in V1 and aVR and an echocardiogram showed left ventricular ejection fraction 35% with akinesia of the apex and parapical segments. Therefore, an emergency coronary angiography was performed showing chronic occlusion of the anterior descending artery. Patient underwent a complex revascularization intervention with good angiographic outcome. Since the onset of fever with chest x-ray suggesting pneumonia (SOFA score 3), antibiotic therapy was started. Two days after hospital admission, the onset of atrial fibrillation occured, thus an attempt of electrical cardioversion was made, but without restoration of sinus rhythm. Given the poor heart rate control with metoprolol at the maximum hemodynamically tolerated dose and cordarone and considering the increased risk of hypotension due to sepsis, an ultraselective beta-blocker was chosen, starting landiolol infusion which resulted in a further reduction of the heart rate of approximately 16%, with similar blood pressure values. Once the acute phase of the sepsis was over, the beta-blocker therapy was progressively reduced by suspending the landiolol infusion and switching to oral therapy with bisolprolol.

Conclusion

In our clinical experience rate control with intravenous landiolol proved to be safe and effective in patient with NSTEMI and sepsis. Hypotension or hemodynamic worsening was not observed.

Patient gave his written consent to the writing of this case report.

References

1. Iguchi S et al. Development of a highly cardioselective ultra short-acting beta-blocker, ONO-1101. Chem Pharm Bull (Tokyo). 1992 Jun;40(6):1462-9.
2. Matsuishi Y et al. Evaluating the Therapeutic Efficacy and Safety of Landiolol Hydrochloride for Management of Arrhythmia in Critical Settings. Vasc Health Risk Manag. 2020 Apr 3;16:111-123.
3. Kakihana Y et al. Efficacy and safety of landiolol, an ultra-short-acting beta1-selective antagonist, for treatment of sepsis-related tachyarrhythmia. Lancet Respir Med. 2020 Sep;8(9):863-872.

## Acute pain

### **A72 Use of serratus plane block and esp block in the management of pain associated with rib fractures in chest trauma, case series**

#### F. Schettino ^1^, F. Coletta ^1^, C. Sala ^1^, A. Tomasello ^1^, A. De Simone ^1^, E. Santoriello ^1^, M. Mainini ^1^, S. Cotena ^1^, R. Villani ^1^

##### ^1^ A.O.R.N. Antonio Cardarelli, Napoli

###### **Correspondence:** F. Schettino

*Journal of Anesthesia, Analgesia and Critical Care 2023,* **3(Suppl 1):**A72

Rib fractures associated with chest trauma are characterized by significant pain. Patients with three or more fractured ribs have an increased risk of lung complications. Pain can impair ventilation and the ability to eliminate secretions due to difficulty coughing, which can cause atelectasis and hypoxia. Analgesic options in chest trauma range from systemic analgesia to invasive regional anesthesia techniques. such as thoracic epidurals, paravertebral catheters, intercostal nerve blocks and blockages of the fascial plane. The aim of the study is to evaluate ESP and SAP blocks analgesic efficacy in chest trauma and their ability to improve respiratory parameters. Primary endpoints were: changes in NRS, change in p|f ratio, possible need for rescue therapy and need for oxygen therapy or ventilatory support. Secondary endpoints were changes in respiratory mechanics evident on the objective examination (respiratory rate, heart rate, signs of respiratory fatigue such as paradoxical breathing, accessory muscles activation ) and length of stay in hospital. Fifteen patients with spontaneous breathing (18 years and older) with isolated thoracic trauma associated to rib fractures who received a fascial plane chest wall block were evaluated. In all patients there was an almost immediate reduction of pain with onset around ten minutes after the block; the oxygen flow support generally decreased after the procedure, and only in one case it was necessary to continue oxygen therapy. From our observation it emerges that chest wall anesthetic blocks are safe and effective techniques in the treatment of pain related to rib fractures. They have also proven to be successful and helpful in improving respiratory parameters and reducing oxygen support.

### A73 Progression from first degree atrioventricular block to complete atrioventricular block after bilateral esp block

#### G.M. Petroni ^1^, P. Fusco ^2^, W. Ciaschi ^3^, F. de Sanctis ^4^, L. Giacomino ^4^, A. Sanapo ^4^, R. Commissari ^4^, M. Divizia ^5^, S. Meloncelli ^5^, F. Marinangeli ^1^

##### ^1^ University of L Aquila, Department of Life, Health and Environmental Sciences, L'Aquila, Italy; ^2^ SS. Filippo e Nicola Hospital Avezzano- Italy, Avezzano, Italy; ^3^ Fabrizio Spaziani Hospital , Department of Anesthesia and Intensive Care Unit,, Frosinone, Italy; ^4^ S. Maria Hospital, terni, Italy, Terni, Italy; ^5^ S.A.M.O. Pain Management Center, Roma, Italy, Roma, Italy

###### **Correspondence:** G.M. Petroni

*Journal of Anesthesia, Analgesia and Critical Care 2023,* **3(Suppl 1):**A73

Background

First-degree atrioventricular block (AVB) may lead to complete AVB. We present a case in which a complete AVB occurred in a patient with a first-degree AVB after performing a bilateral erector spinae plane (ESP) block.

A first-degree atrioventricular block (AVB) is defined as the prolongation of the PR interval on an electrocardiogram to more than 200 msec. Due to various triggers, first-degree AVB may evolve into a third-degree AVB. A complete AVB may be triggered by surgical and non-surgical vagal stimuli and could also be associated with cardiomyopathies, sarcoidosis, amyloidosis, rheumatic fever, infections, endocarditis, other conditions and certain drugs. Several cases of complete AVB with epidural ropivacaine infusion in patients with a first degree AVB have been described in the literature, but none of these describe to a progression from a first-degree to a third-degree AVB after an erector spinae plane (ESP) block. [1], [2]

We describe a case in which a complete AVB occurred in a patient with a first-degree AVB (PR > 0.20s and < 0.30s) after performing a bilateral ESP block with ropivacaine

Case report

The case involved a patient in their 80s weighing 75kg who underwent laparotomy surgery for gangrenous cholecystitis. This patient had several comorbidities, including: arterial hypertension, diabetes mellitus, chronic obstructive pulmonary disease (COPD), and a first-degree AVB. After surgery, the patient was admitted to the intensive care unit and immediately extubated. The patient complained of abdominal pain with NRS 8, agitation and tachypnea.

An ultrasound ESP block was performed at T8 level under aseptic conditions. Using an in-plane approach, a 18 G Tuohy needle was inserted in a caudal–cephalad direction, until the tip was below the erector spinae muscle, as evidenced by visible hydrodissection below the muscle plane, and by the injection of a 5 ml normal saline. A 20 G epidural catheter was threaded 4 cm in a cephalad direction. The same procedure was performed on the opposite side. A 20 ml bolus dose of 0.375% ropivacaine was administered through the catheter after a negative blood aspiration.

Pain was assessed using the numerical rating scale (NRS). After 10 minutes, the NRS was 0. About 15 minutes after the execution of the ESP block, the patient developed a third-degree AVB, without hemodynamic instability, but which required placing a temporary pacemaker. The local anesthetic (LA) administered was less than the toxic dose (3mg/kg). [3]

Conclusion

Many studies show that the efficacy of an ESP block is based on the anesthetic spreading partly in the paravertebral space and subsequently through the intervertebral foramina into epidural space. [4]

Based on our experience, the ESP block likely caused a complete AVB a patient with a first degree AVB. The most likely cause of this complete AVB is the sympathetic blockade by the LA spread into the epidural space. We performed the block at the T8 level, introducing the catheter 4 cm deep and injecting the LA in caudal-cranial direction. Human preganglionic sympathetic cardiac neurons originate mainly from the T1-T4 or T5 thoracic spinal segments. [5]

Informed consent to publish had been obtained

## Chronic pain

### **A74 Effectiveness of Dry Needling (DN) for Myofascial Trigger Points (TrPs) Associated with Neck and shoulder Pain: a case report**

#### Roberta C, Alessandro F, Antonella M, Antonella F, Rosa Maria Z, Donatella B

##### Ospedale Civile S. Spirito, Pescara, Italy

###### **Correspondence:** Roberta C

*Journal of Anesthesia, Analgesia and Critical Care 2023,* **3(Suppl 1):**A74

Background

Neck and shoulder pain has a prevalence of 70% in general population [1]. The first therapeutic option usually consists in physical manipulation, rest, analgesics, local anaesthetics or botulinum toxin injection. Neck pain might be caused by the activation of TrPs, defined as “a hypersensitive spot located in a taut band of skeletal muscle which stimulation induces referred pain symptoms and motor phenomena” [2], mostly found in the upper Trapezius Muscle. Inserting a thin needle trough skin in order to reach the TrPs and manipulating it with static and dynamic DN technique might release the muscular contraction and improve the neck range-of-motion (ROM). When a TrPs is reached, a local twitch is elicitated to confirm the right position of the needle [3].

Case report

This report describes the case of a 48 years-old caucasian woman affected by neck and shoulder pain with medical history of failed back-surgery syndrome (L3-L5 arthrodesys). Altough upper-limb sensitive-evoked potentials were normal, cervical column MRI-scan evidenced C5-C6 osteocondrosys and disc dislocation from C4 to C7 with conflict zones on the dura mater. The examination of the trapezius muscle revealed a strong contracture that evoked pain, reduced the ROM and denied resting. The patient referred pain intensity on the numerical rating scale (NRS) as 8-9, localized on neck and shoulders, albeit in therapy with opioids, cannabinoids, miorelaxants and steroids. In addition to that, she underwent ultrasound-guided local anesthetics and steroids perineural injections that led to a brief pain relief. Due to poor outcome of conventionl therapy, she underwent bilateral DN procedure of the upper trapezius muscle branch. After three days from the second treatment, she referred a NRS of 7-8. After eitght days, before the third treatment, NRS was 7. TrPs elicitation in each treatment was significant and bilateral. In the follow-up visit, after one month, the patient referred a NRS of 5, improved ROM and better rest.

Conclusions

DN of the upper-branch of the trapezius muscle can relieve muscular contracture and reduce associated pain if conducted with the right elicitation of TrPs. ROM could also be improved, thus leading to a better quality of life in patients with neck and shoulder pain.

Informed consent to publish had been obtained

References

1. Marcos J Navarro-Santana, Jorge Sanchez-Infante, César Férnandez-de-la-Penas, Joshua A. Cleland, Patricia Martin-Casas, Gustavo Plaza-Manzano. Effectiveness of Dry Needling for myofascial Trigger Points associated with neck pain symptoms: an updated systematic review and meta-analysis. J Clin Med. 2020; 9: 3300
2. David G. Simons, Janet G. Travell. Myofascial pain and dysfunction: the trigger point manual, 3rd ed. 2019
3. César Férnandez-de-la-Penas, Jo Nijs. Trigger point dry needling for the treatment of myofascial pain syndrome: current perspectives within a pain neuroscience paradigm. J Pain Res. 2019; 12:1899- 1911

### A75 Long-term effect of electroacupuncture in fibromyalgia patients: a retrospective study on duration of pain relief and improvement of life quality

#### S. Pitoni, D. Del Prete, C. Riso, A. Scarano, M. Del Vicario, G. Cannelli, L. Zappia, M. Rossi

##### UCSC, Catholic University of the Sacred Heart, Department of Emergency, Anesthesiology and Intensive Care, Rome, Italy, Roma, Italy

###### **Correspondence:** S. Pitoni

*Journal of Anesthesia, Analgesia and Critical Care 2023,* **3(Suppl 1):**A75

Background

Fibromyalgia (FM) is a highly prevalent chronic pain syndrome that affects 2-5% of the population and causes significant morbidity and disability. Currently, non-pharmacological treatments have a central role. Electroacupuncture (EA) determines an improvement of short-term pain in FM patients. The objective of this study is to assess long-term pain relief after EA.

Materials and methods

The present research is a “before-after” retrospective study conducted on 21 adult patients with FM, treated with EA at the Acupuncture Clinic of Gemelli Hospital between 12/2020 and 08/2022. Patient’s consent was obtained before EA. Patients underwent a cycle of 6 EA sessions on a weekly basis. Before and after the EA cycle the following questionnaires were administered: NRS, Brief Pain Inventory (BPI), Revised Fibromyalgia Impact Questionnaire (FIQR), Fatigue Severity Scale (FSS), Nicholson McBride Resilience Questionnaire (NMRQ) and SF-36. Before (T0) and after the last EA session, the FM patients underwent 3 follow-up visits, named respectively T1 (within 7days after the last EA session), T2 (after 30 days) and T3 (90 days).

Results

Patients’ characteristics are presented in Table 1.

After the EA cycle, the NRS, PSS-BPI, PIS-BPI, MH-SF36, RP-SF36 and the FIQR Depression subscale (11-points Likert self-assessed scale) showed a significant improvement from T0 to T1, but it didn’t persist at T2 and T3 (See Graph 1 and 2, T0 vs T1: NRS from 6.5±1.9 to 4.7±2.1, PSS from 6.2±2.0 to 4.6±2.1, PIS from 7.1±2.0 to 5.4±2.2, MH from 40.0±16.2 to 49.4±13.9, RP from 6.3±19.4 to 19.1±32.5, Depression from 6.8±3.3 to 4.6±2.9, p<0.05). Additionally, Anxiety (11-points Likert self-assessed scale), showed an implementation after the end of the EA treatment, reaching the significance at T3 (See Graph 3, T0 vs T3: Anxiety from 5.4±2.4 to 7.2±1.3, p<0.05). Finally, FIQR, FSS and NMRQ didn’t change between T0, T1 and T3.

Conclusions

In FM a single cycle of 6 weekly session of EA reduces pain (NRS, PSS-BPI) and its interference with daily activities (PIS-BPI), thus decreasing patients’ role limitations due to physical health problems (RP-SF36). Moreover, EA improves emotional well-being (MH-SF36) and diminishes depression. However, EA-associated health benefits are not persistent and decay within 3 months after the end of the EA. This phenomenon is associated with an increase of anxiety at T3. Therefore, EA should not be considered as an isolated treatment, but it is valuable within a multimodal approach. Different scoring systems are needed to monitor its effects on mid and long term.

**Table 1 (abstract A75).** See text for description

Patient's characteristics	Mean ± SD, Frequency (%)
Age (y)	52.0± 10.7
BMI (Kg/m2)	25.3± 6.6
Gender	F: 19/21 (90.5%)
	M: 2/21 (9.5%)
Acupuncture naive	14/21 (66.7.%)
Primary FM	14/21 (66.7%)
FM and autoimmune disease	7/21 (33.3%)
Education	Primary and lower secondary (age 5-13 ys): 5/21 (23.8%)
	Upper secondary (age 14-18 ys): 11/21 (52.4%)
	Degree or more(>18ys): 5/21 (23.8%)
Pain therapy	No drugs: 2/21 (9.5%)
	1 drug: 10/21 (47.6%)
	2 drugs: 5/21 (23.8%)
	3 or more drugs: 4/21 (19.1%)
	Muscle relaxants: 6/21 (28.6%)
	Antidepressants: 7/21 (33.3%)
	Antiepileptics: 5/21 (23.8%)
	Paracetamol: 2/21 (9.5%)
	Cox-2 inhibitors: 1/21 (4.8%)
	Opioids: 10/21 (47.6%)
	Tapentadol: 5/21 (%)
	Buprenorphine transdermal patch: 5/21 (%)
	Benzodiazepines: 2/21 (9.5%)
	Cannabis: 1/21 (4.8%)
Rescue medications	No rescue meds: 10/21 (47.6%)
	Paracetamol: 3/21 (14.3%)
	Other drug: 10/21 (47.6%)
WPI+SS (T0)	20.3± 5.3
FIQR (T0)	61.6±22.6

**Fig. 1 (abstract A75).**
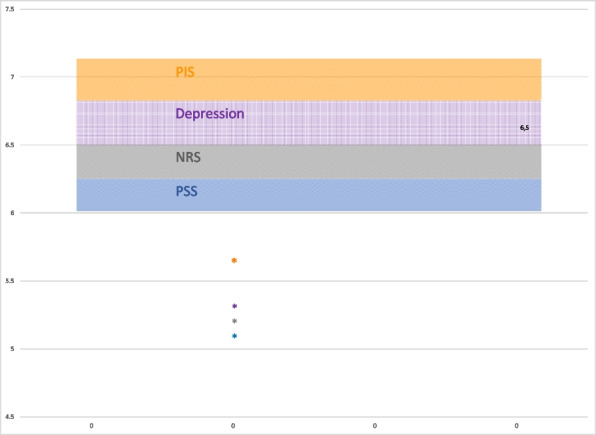
See text for description

**Fig. 2 (abstract A75).**
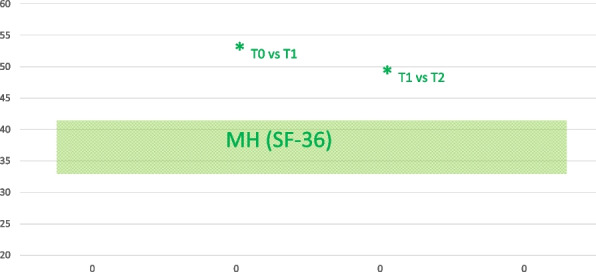
See text for description

**Fig. 3 (abstract A75).**
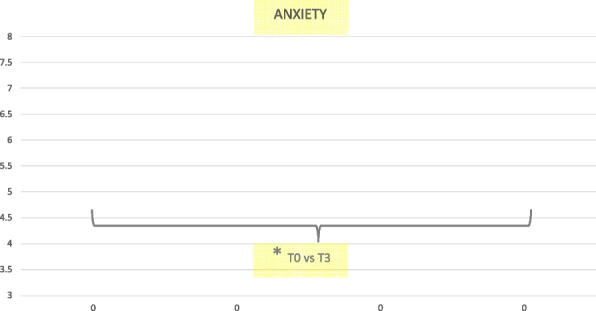
See text for description

### A76 Treatment of persistent neuropathic pain induced by forearm crush injury with pulsed radiofrequency. A case report

#### M. Palazzo ^1^, D. Marelli ^2^, N. Petrucci ^1^

##### ^1^ Asst Garda, desenzano, Italy; ^2^ univesità di Brescia, Brescia, Italy

###### **Correspondence:** M. palazzo

*Journal of Anesthesia, Analgesia and Critical Care 2023,* **3(Suppl 1):**A76

Rationale: The case of a patient with recurrent neuropathic pain caused by a forearm crush injury is described in this article. The patient provided written informed consent for this case report and its associated images to be published. The patient was a 45-year-old man who visited the Pain Therapy Clinic at Desenzano Hospital. Under ultrasound guidance, the patient responded effectively to Pulsed Radiofrequency (PRF).

Background: With a Numeric Rating Scale (NRS) score of 9, the patient complained of neuropathic pain from a crush injury to the left forearm. A nerve conduction study/electromyography confirmed nerve damage in the forearm. After being caught in a press between two glue spreader cylinders during March 2019 with his entire forearm up to his elbow, he sustained a crush injury, compressing the muscles, but no fractures. For more than a year, the medial side of the left elbow, forearm, and hand suffered tingling and piercing pain. His pain was not relieved by oral medication as prescribed by his General Practitioner, laser therapy or physical therapy. We performed ultrasound-guided nerve block of ulnar nerve, radial nerve and medial cutaneous nerve with injection of 4 cc of 0.5 % lidocaine. Pain relief surrounding the nerve block was immediately achieved. Because the effect of peripheral nerve block test with local anesthetics was found to be positive, we considered performing PRF lesioning on all three nerves.

Methods: Under ultrasound guidance, PRF stimulation of the left ulnar nerve, radial nerve, and medial cutaneous nerve of the forearm was conducted at the lateral epicondyle level and medial mid-arm. The patient felt dysesthesia and a tingling sensation at the nerve’s innervation region with 0.4 V stimulation. The PRF treatment was performed at 45 V for 360 seconds, at 5Hz and 5ms pulsed width, with the temperature of the electrode tips not exceeding 42 °C.

Results: The patient’s pain was entirely relieved with an NRS score of 1 at the 1-, 3-, 6- and 9-month follow-up examinations. There were no adverse side effects.

Conclusions: The administration of PRF to the medial cutaneous nerve, radial nerve, and ulnar nerve of the forearm has been shown to effectively reduce neuropathic pain caused by a forearm crush injury.

**Fig. 1 (abstract A76).**
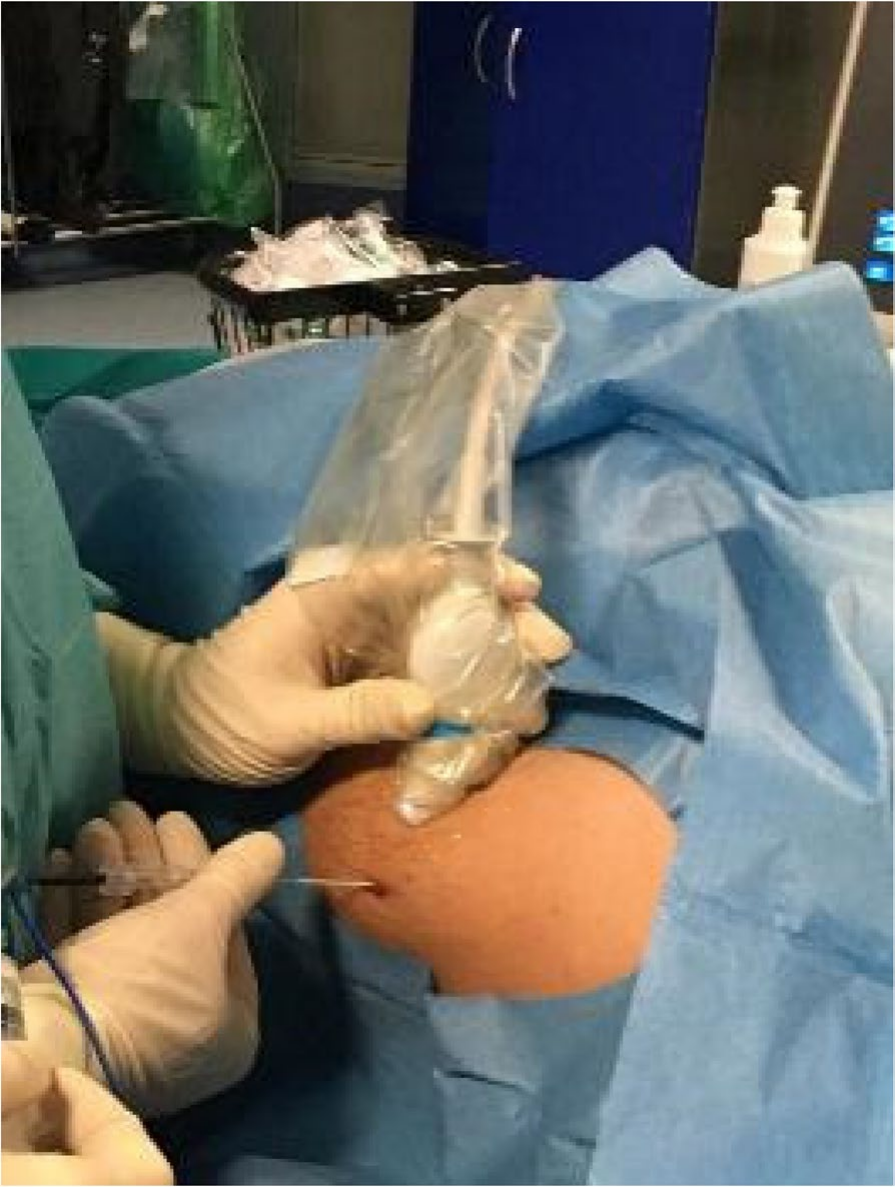
See text for description

### A77 Persistent neuropathic pain treated with Capsaicin patches

#### G. Ferraro ^1^, V. Gagliardi ^2^, A. Butturini ^1^, F. Ceccherelli ^2,3^, G. Gagliardi ^1,2,3^

##### ^1^ Ospedale Santa Maria della Misericordia, Rovigo, Italy; ^2^ Università degli Studi di Padova, Padova, Italy, ^3^ A.I.R.A.S., Padova, Italy

###### **Correspondence:** G. Ferraro

*Journal of Anesthesia, Analgesia and Critical Care 2023,* **3(Suppl 1):**A77

Neuropathic pain is associated with a functional abnormality of the nervous system due to a direct lesion or disease of the somatosensory system. Capsaicin has been used in several clinical settings as a topical medication to treat neuropathic pain. We report three cases of persistent neuropathic pain who underwent this protocol: three applications of the capsaicin 8% patch separated by one month.

Written informed consent was obtained from all patient.

Background. Capsaicin has used in several clinical settings as a topical medication to treat pain derived from different conditions. It selectively stimulates nociceptive neurons and has been widely used to study pain-related events. It has been demonstrated that capsaicin activates TRPV1 (the transient receptor potential vanilloid subtype 1). TRPV1 is mainly expressed in neuronal cells, as trigeminal nerves and dorsal region ganglia. Capsaicin has a selective action on C-polymodal nociceptors, also mediated by reactive oxygen species (ROS). High or repeated doses of capsaicin induce an initial pain sensation, followed by analgesia. After exposure to a high or repeated dose of capsaicin, the TRPV1 receptors begin a refractory state commonly termed as desensitization which leads to inhibition of receptor function [1]. Some studies indicated capsaicin participation in neurotransmission in a TRPV1-independent manner. It seems to be able to modulate the synaptic transmission acting through pre- and postsynaptic mechanisms [2].

Cases report. CASE 1, POST HERPETIC NEURALGIA (PHN) (Figure 1): 41 years-old male patient affected by persistent pain lasting for 1 year, due to post herpetic neuralgia involving the area covered by the supraorbital nerve. At the cellular level, PHN upregulates the receptors typically associated with pain, such as TRPV1. After the tratment the outcome has resulted to a significant reduction of the symptoms: the pain relief has maintained, staggered only by sporadic episodes of itching.

CASE 2 DEAFFERENTATION (Figure 2): 32 years-old male patient has had post-inguinal herniorrhaphy painful consequences due to triple neurotomy for 48 months. The clinical outcome at the end of the therapy consists of a reduction of 50% of the initial pain.

CASE 3, CRPS I (complex regional pain syndrome) (Figure 3): 20-years old female patient presenting persistent pain in the left ankle in CPRS-I consequent to a distorsive trauma. The woman underwent the implantation of a perinervous neurostimulator on sural nerve, with acceptable control of the pain, but without relief from the vasomotor manifestation and the persistence of cutaneous hyperalgesia. After the treatment with Capsaicin patch we have detected a reduction of the intensity of the pain.

Conclusions: The treatment is effective in reducing the intensity of resistant pain, and it consists of a valid therapy for the treatment of the chronic neuropathic pain.

References

A) Fattori V, Hohmann MS, Rossaneis AC, Pinho-Ribeiro FA, Verri WA. Capsaicin: Current Understanding of Its Mechanisms and Therapy of Pain and Other Pre-Clinical and Clinical Uses. Molecules. 2016 Jun 28;21(7):844
B) Braga Ferreira LG, Faria JV, Dos Santos JPS, Faria RX. Capsaicin: TRPV1-independent mechanisms and novel therapeutic possibilities. Eur J Pharmacol. 2020 Nov 15 887:173356

**Fig. 1 (abstract A77).**
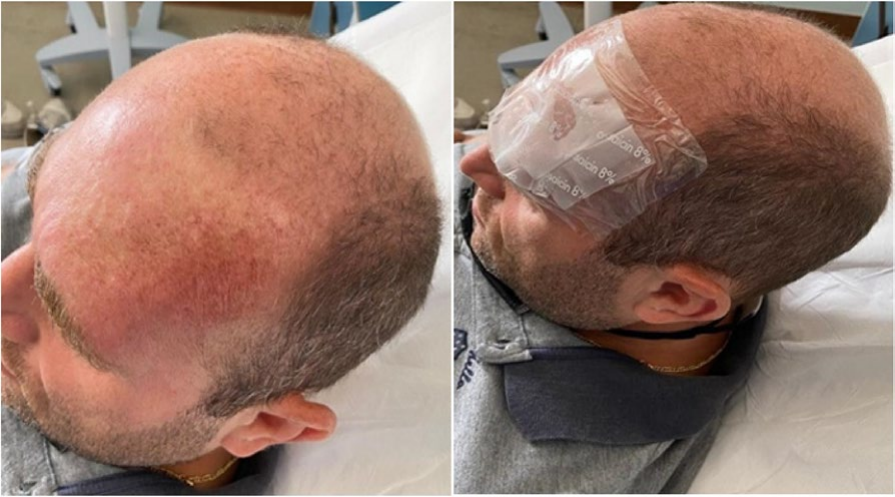
See text for description

**Fig. 2 (abstract A77).**
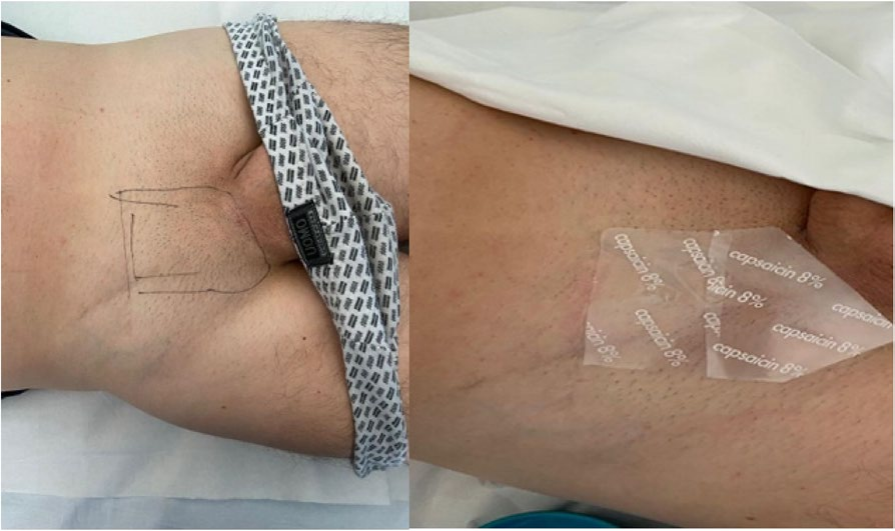
See text for description

**Fig. 3 (abstract A77).**
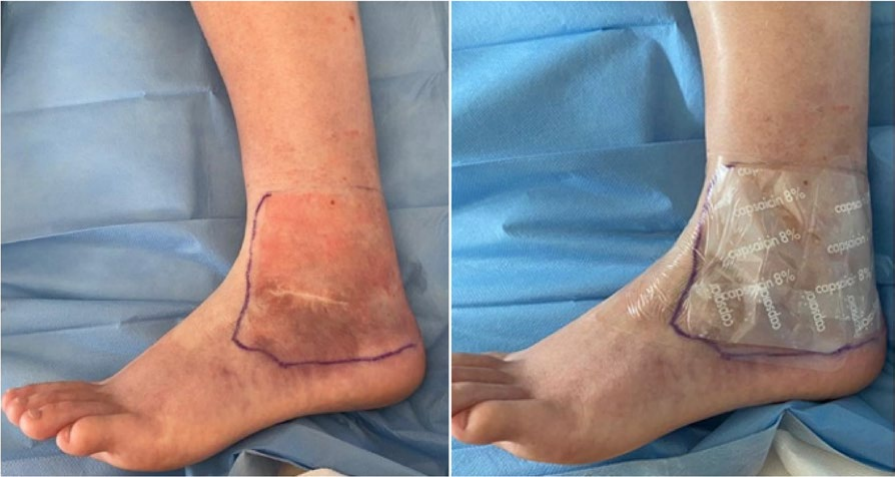
See text for description

### A78 The use of therapeutic cannabis in an italian Hub Center: an evolving landscape of medical treatment

#### L. Baiocchi ^1^, A. Catalano ^1^, A. Scarano ^1^, F. Sbaraglia ^1^, D. Del Prete ^1^, M. Costanzi ^1^, G. Ferrone ^1^, C.F. Riso ^1^, L. Zappia ^1^, M. Rossi ^1^

##### ^1^ Policlinico Universitario Agostino Gemelli, UCSC Gemelli IRCCS, Roma, Italy

###### **Correspondence:** L. Baiocchi

*Journal of Anesthesia, Analgesia and Critical Care 2023,* **3(Suppl 1):**A78

Background: Medical applications of therapeutic cannabis encompass a broad range of conditions. Several studies have shown that the active compounds in cannabis possesses analgesic, anti-inflammatory and neuroprotective properties, making them valuable options for patients with chronic pain or neurodegenerative disorders[1-4].

We retrospectively reviewed the surrounding realities of medical cannabis prescriptions at Pain Center of Policlinico Agostino Gemelli in Rome.

Case Description: Between 2017 and 2023, 169 patients have been treated for chronic pain adding medical cannabis in the therapeutic scheme. According to our protocol, after a positive agreement, psychological status is assessed and a written informed consent is acquired. Pain therapist sets the most suitable treatment plan for the patients and books the first follow up control after 1 month, and then at 2 months intervals, integrated with frequent mail connections and telephone calls. Several formulations with different contents of Tetrahydrocannabinol (THC) and Cannabidiol (CBD) were prescribed during the observational period (Fig. 1) (Tab.1).

The hospital pharmacy, according the regional procedure, communicates the individual code to the external supplying pharmacy, where the patient may collect the personalized medication.

121 patients went at follow up regularly and followed the prescription without any trouble. Forty eight patients (28%) drop out to follow-up: 6 due to inefficacy, 7 due to intolerance, and 4 due to psychiatric indications.

The retrospective analysis showed that many obstacles may negatively affect the regular cannabis administration, not allowing an adequate evaluation about its therapeutic effectiveness. Temporary discontinuation or delay in prescriptions occurred. Inadequate supplies through international allowed channels as well as insufficient production at national level or inadequate support by local pharmacies led us to adequate the therapy basing ourselves to the disposable medications.

Conclusion:

A preliminary analysis of the collected data show how the issue of efficacy and adherence to therapy with medical cannabis has multiple causes, not all related to patient behavior. Availability of different formulations is crucial for defining a tailored therapy, balancing the effects of THC e CBD and allowing personalized and not fixed scheme of administration. Unfortunately, frequent lacking in sourcing negatively affected these therapeutic choices, not least the recent Covid pandemic. Our report describes an integrated care pathway providing a safe and effective system looking after patients on cannabis therapy. The tight follow up allowed us to identify therapy discontinuations and possibly to avoid misconducts.

Despite the progress made in therapeutic cannabis integration, accessibility remains an open issue. Improving supplies and connection framework between sourcing services and hospitals will definitely guarantee a better service for these patients.

References

1. Carroll C, Bain P, Teare L, et al. Cannabis for Dyskinesia in Parkinson Disease a Randomized Double-Blind Crossover Study. Vol 63.; 2004. www.neurology.org
2. Campos AC, Fogaça M V., Sonego AB, Guimarães FS. Cannabidiol, neuroprotection and neuropsychiatric disorders. Pharmacol Res. 2016;112:119-127.
3. Busse JW, MacKillop J. Medical cannabis and cannabinoids for chronic pain: Summary of a Rapid Recommendation. J Mil Veteran Fam Health. 2021; 7:118-122.
4. Black N, Stockings E, Campbell G, et al. Cannabinoids for the treatment of mental disorders and symptoms of mental disorders: a systematic review and meta-analysis. Lancet Psychiatry. 2019;6(12):995-1010.
5. Cannabis Medical use Decree (09/11/2015). Accessed November 30, 2015. https://www.epicentro.iss.it/cannabis-uso-medico/pdf/Decreto%20uso%20medico%20Cannabis%20(GU%2030.11.2015)%20.pdf
6. Bettiol A, Lombardi N, Crescioli G, et al. Galenic preparations of therapeutic Cannabis sativa differ in cannabinoids concentration: A quantitative analysis of variability and possible clinical implications. Front Pharmacol. 2019;9(JAN).

**Fig. 1 (abstract A78).**
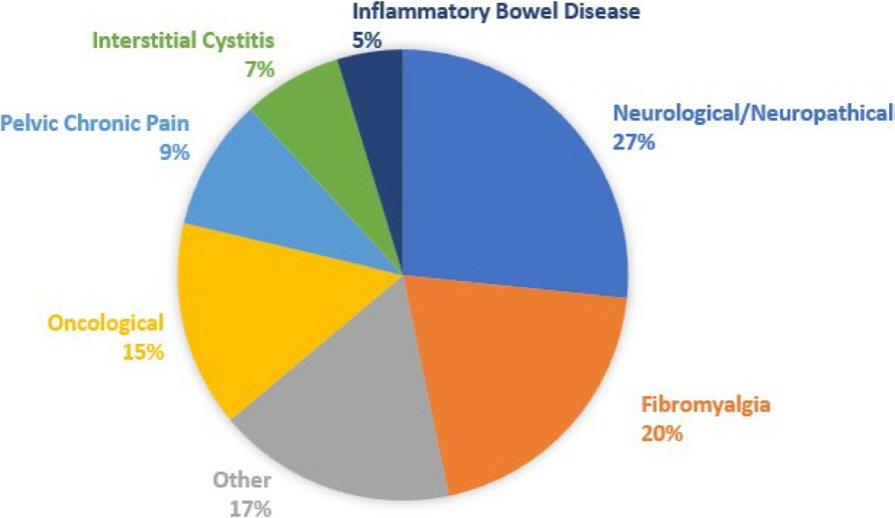
First diagnosis leading to Cannabis treatment

**Table 1 (abstract A78).** Types of used formulations with respective content of Delta-9-tetrahydrocannabinol (THC) and Cannabidiol (CBD)[6]

Phytocannabinoid variety	THC content	CBD content	Patients in therapy (1°prescription)
Bedrocan	>19%	<1%	102
Bediol	>6%	7.50%	14
Bedica	>14%	<1%	11
Bedrolite	<1%	9%	7
FM-2	5-8%	7, 5-12%	4
Pedanios 22-1	22%	<1%	10
Farmalabor	>15%	<1%	21

FM (Farmaceutico militare); THC (tetrahydrocannabinol); CBD (cannabidiol)

## Oncologic pain and palliative care

### **A79 Nutrition and treatments in simultaneous care**

#### G. Acerra, P. Cuofano, E. Falomi, F. Scogliera, A. Cerrone, R. Dell'Orto, A. Mignone

##### Hospice il Giardino dei Girasoli - Medicina del Dolore - Cure Palliative - Centro NAD - DS 64 - Eboli - Asl Salerno - Italy

###### **Correspondence:** P. Cuofano

*Journal of Anesthesia, Analgesia and Critical Care 2023,* **3(Suppl 1):**A79

BACKGROUND

Malnutrition is a state of imbalance between intake of nutrients and energy and body's demand to maintain homeostasis [1].

PreMio observational study, conducted between 2012 and 2014 in Italy, indicates that 42.2 % of cancer patients are at risk of malnutrition already at the first oncological visit, with a higher percentage for gastro-oesophageal cancer cases [2].

Malnutrition tends to worsen with side effects of radio-chemotherapy, especially when they affect the integrity of the mucosa, such as oral candidiasis (common to use local miconazole, which reduces systemic side effects and increases patient compliance [3]. Furthermore, the combination of oral nutritional supplements with the administration of megestrol acetate, the only drug in Europe indicated for the treatment of anorexia and weight loss secondary to neoplasia, stimulates appetite in patients with lung cancer and improves their general condition.

MATERIALS, METHODS AND RESULTS

Il giardino dei Girasoli located in Eboli (SA) is the only Hospice in Italy to have a N.A.D. (Home Artificial Nutrition) center in its structure, this has allowed us to carry out nutritional screening in the early stages of the disease thus associating early nutritional modifications (simultaneous care) with first line oncological treatments. From 2019 to 2022 we enrolled 145 patients and we treated all side effects pre and during chemo with miconazole for candidiasis, local administration of mucoadhesive tablets that allow slow-release over 24 hours. We administered megastrole to 52 patients, for 72 patients we also treated pain either with cannabis alone or in association with oxycodone+naloxone or oxycodone+ paracetamole, for all of them we ensured a caloric intake of 2500 calories/day also through breaks, we noted in all of them almost complete recovery of lost weight except in 9 patients who, however, already had secondarisms.

CONCLUSIONS

As our results show, it is extremely useful to know the nutritional status of the patient before first-line chemo-radiotherapy treatment starts, nutritional status may affect tolerance of chemotherapy and radiotherapy as well as the survival of the patient.

Authors have no conflict of interest.

REFERENCES

1. Definition of malnutrition - https://www.who.int/
2. Muscaritoli M, et al. Prevalence of malnutrition in patients at first medical oncology visit: the PreMiO study. Oncotarget. 2017 Oct 3; 8(45): 79884–79896.
3. Collins DC, et al. Management of oropharyngeal candidiasis with localized oral miconazole therapy: efficacy, safety, and patient acceptability. Patient Prefer Adherence. 2011; 5: 369–374.

## Multiorgan donor and Anesthesia and Intensive care on organ transplant

### **A80 Use of the Cytosorb device in patients with severe graft dysfunction after Liver Transplantation**

#### R. Gaspari ^1,2^, G. Spinazzola ^2^, M. Chioffi ^2^, S. Postorino ^2^, T. Michi ^2^, E. Piervincenzi ^2^, A.W. Avolio ^3,4^, M. Antonelli ^1,2^

##### ^1^ Department of Basic Biotechnological Sciences, Intensive and Perioperative Clinics, Catholic University of the Sacred, Roma, Italy; ^2^ Department of Anesthesia, Emergency and Intensive Care Medicine, Fondazione Policlinico Universitario A. Gemelli IRCC, Roma, Italy; ^3^ Department of Translational Medicine and Surgery, Catholic University of the Sacred Heart, Roma, Italy; ^4^ General Surgery and Liver Transplantation, Fondazione Policlinico Universitario Agostino Gemelli IRCCS, Roma, Italy

###### **Correspondence:** M. Chioffi

*Journal of Anesthesia, Analgesia and Critical Care 2023,* **3(Suppl 1):**A80

Italy.

Background. Cytosorb (CytoSorbents Corporation, Monmouth Junction, USA) is a hemoadsorption device capable of removing molecules up to 60 kda. Cytosorb was mainly used in septic shock to capture cytokines responsible for hyperinflammation. The device is also effective in the removal of bilirubin, bile acids, myoglobin and other toxins, but is never used in patients undergoing liver transplantation (LT) with severe graft dysfunction.

Material and methods. The CytoSorb filter was placed in series on a hemofiltration machine (Gambro Prismaflex Illinois, USA) with a dedicated set of hemoperfusion lines. Patients received two treatments, each lasting 20 consecutive hours with a 4-hour break, without the use of anticoagulant therapy. Biochemical, blood and standard coagulation tests were performed at the beginning and end of each treatment. Informed consent was obtained from the patient or relatives.

Results. Three patients admitted to our Surgical Intensive Care Unit (SICU) following LT from deceased donor were treated with Continuos-Veno-Venous Hemofiltration-CVVH and Cytosorb.

Case 1: 49-year-old male, Body Mass Index-BMI 23.2, affected by primary sclerosing cholangitis, Model for End-Stage Liver Disease-MELD 22, Simplified Acute Physiology (SAPS) II 35. In the postoperative (PO) the patient developed Acute Kidney Injury-AKI and hyperbilirubinemia, which were treated on 5th day with CVVH-Cytosorb. The patient was discharged from the SICU on the 9th PO day.

Case 2: 50-year-old male, BMI 21.9, affected by hepatocarcinoma on liver cirrhosis HCV related, MELD 15, SAPSII 42. In the immediate PO period, there was no sign of initial functional recovery of the graft (elevated transaminases, no bile production, coma state) and additionally the patient developed AKI. A diagnosis of primary nonfunction was made and he was placed on the urgent re-LT list. While awaiting surgical procedure, the patient underwent two treatments CVVH-Cytosorb and the 4th PO day was successfully re-LT. He was discharged from the SICU on the 13th PO day.

Case3: 58-year-old male, BMI 26.9, suffering from alcoholic liver cirrhosis and hepatocarcinoma, MELD 16, SAPSII 27. On the 7th PO day, the patient developed severe hyperbilirubinemia (histological examination: severe canicular cholestasis) and AKI. CVVH-Cytosorb treatment was started and on the 17th PO day he was discharged from SICU.

In the Table 1 are reported the biochemical parameters of each patient at the beginning of the first treatment and at the end of the second extracorporeal treatment.

Conclusions: Cytosorb markedly reduced serum bilirubin and ammonia. In patients who need hemodialytic treatment for AKI the use of Cytosorb does not increase the risks arising from extracorporeal therapy. Further studies are needed to determine whether Cytosorb is effective as a bridge to graft recovery or to support the patient toward re-LT.

References

1. Jansen A, Waalders NJB, van Lier DPT, Kox M, Pickkers P. (2023) CytoSorb hemoperfusion markedly attenuates circulating cytokine concentrations during systemic inflammation in humans in vivo. Crit Care 27(1):117. https://doi.org/10.1186/s13054-023-04391-z
2. Dhokia VD, Madhavan D, Austin A, Morris CG. (2019) Novel use of Cytosorb™ haemadsorption to provide biochemical control in liver impairment. J Intensive Care Soc 20(2): 174-181. doi:10.1177/1751143718772789J

**Table 1 (abstract A80).**
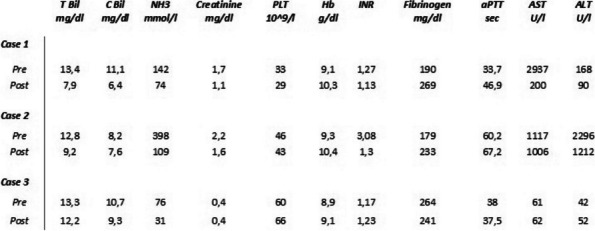
Biochemical parameters of patients at the beginning and end of extracorporeal treatments

*Abbreviations: T Bil* total bilirubin, *C Bil* conjugated bilirubin, *PLT* platelet count, *Hb* hemoglobin, *INR* International Normalized Ratio, *aPTT* activated partial thromboplastin time, *AST* aspartate aminotransferase, *ALT* alanine aminotransferose

### A81 Diagnosis and management of Intensive Care Unit Acquired Weakness (ICUAW) in critically ill patients undergoing liver transplantation

#### R. Gaspari ^1,2^, G. Spinazzola ^2^, M. Delli Compagni ^2^, S. Postorino ^2^, T. Michi ^2^, D.C. Fachechi ^2^, A.W. Avolio ^3,4^, M. Antonelli ^1,2^

##### ^1^ Dep of Basic Biotechnological Sciences, Intensive and Perioperative Clinics,Catholic University of The Sacred Heart, Roma, Italy; ^2^ Department of Anesthesia, Emergency and Intensive Care Medicine, Fondazione Policlinico Universitario A. Gemelli IRCCS, Roma, Italy; ^3^ Department of Translational Medicine and Surgery, Catholic University of The Sacred Heart, Roma, Italy; ^4^ General Surgery and Liver Transplantation, Fondazione Policlinico Universitario Agostino Gemelli IRCCS, Roma, Italy

###### **Correspondence:** M. Delli Compagni

*Journal of Anesthesia, Analgesia and Critical Care 2023,* **3(Suppl 1):**A81

Background. Intensive Care Unit Acquired Weakness (ICUAW) syndrome is characterized by the onset of neuromuscular weakness with no plausible causes other than critical illness [1]. ICUAW is rarely reported in patients undergoing liver transplantation (LT) [2]. Despite the difficulty of diagnosis due to confounding factors such as diabetes, multiple organ failure or sepsis, its early identification and treatment are essential to improve functional recovery and avoid other complications.

Methods and Materials. All patients admitted to the Intensive Care Unit (ICU) after LT surgery who developed severe limb weakness have been analyzed. Electromyography (EMG) and nerve conduction studies (NCS) have been used for the diagnosis of ICUAW. Fibrillation potentials at EMG on the deltoid, brachial and rectus femoris muscles, absence of sensitive potential on sural nerve, and absence of motor response to direct stimulation of median nerve in NCS indicated ICUAW. Informed consent was obtained from patients.

Results. Between January 2017 and January 2023, 197 patients underwent LT from deceased donor at Fondazione Policlinico Universitario A. Gemelli IRCCS Rome. Sixteen of them showed limb weakness. The electrophysiological diagnosis of ICUAW was made in six patients, in the remaining ten patients only muscle deconditioning was found. Clinical and surgical characteristics are reported in Table 1. Patients developed ICUAW 9±4 days after LT. Patients’ age was 46.5±17 years, Body Mass Index 27±5.7, Model for End stage Liver Disease (MELD) 36.5 ± 4.7, Simplified Acute Physiology Score (SAPS) II 60±11, length of surgery 10.1 ± 2.7 hours.

Intraoperative transfusions were: Red Blood Cells 16.7 ± 10.7 units, fresh frozen plasma 10.8 ±6.9 units, and platelets 3.5±3.2 units. ICU length of stay (LOS) was 33.7±26.5 days, and duration of mechanical ventilation (MV) 175.5±86.7 hours. Two patients were tracheostomized, and 2 patients were re-transplanted for primary nonfunction (PNF) of the graft. All patients developed Acute Kidney Injury treated with continuous veno-venous hemofiltration (CVVH) and contracted infections as reported in Table 1. No patient was diagnosed with graft rejection. We treated ICAW with Nicetile 500 mg im twice a day, Thiamine im 100 mg once a day, group B vitamins 1 bottle once a day and intense physiotherapy. All patients had good clinical recovery and are alive 90 days after LT.

Conclusion. ICUAW is rarely reported after LT. In our series early electrophysiological diagnosis, prompt medical therapy, intense daily rehabilitation, glycemic control, strict monitoring and treatment of infections could improve patients’ prognosis.

References

1. Hermans G, Van den Berghe G. Clinical review: intensive care unit acquired weakness. Crit Care. 2015; 19(1): 274. doi: 10.1186/s13054-015-0993-7. PMID: 26242743; PMCID: PMC4526175.
2. Chen WY, Lin PY, Lai CH, Chen YL. Evaluation of Clinical Neuropathy After Living Donor Liver Transplant. Exp Clin Transplant. 2021; 19(7): 664-670. doi: 10.6002/ect.2020.0392. Epub 2021 PMID: 34085916

**Table 1 (abstract A81).** Characteristics of patients affected by Intensive Care Unit Acquired Weakness (ICUAW)

***Parameters***	***Pt 1***	***Pt 2***	***Pt 3***	***Pt 4***	***Pt 5***	***Pt 6***
*Age (y)*	64	56	58	38	17	46
*Gender (M/F)*	M	M	F	F	M	M
*Body Mass Index*	26,6	22,2	34,2	33,5	20,5	25
*Hyperglycemia*	Yes	Yes	Yes	Yes	Yes	Yes
*MELD score*	30	38	40	40	40	31
*D-MELD*	810	912	3105	3160	1062	2139
*SAPS II score*	46	76	49	60	67	67
Etiology*:*						
*Alchool*	No	Yes	No	Yes	No	No
*Virus*	No	Yes	No	No	No	No
*Hepatocarcinoma*	No	Yes	No	No	No	No
*Other*	Cryptogenetic	-	Polycystosis	-	Cryptogenetic	Trauma
*ICU stay before LT (d)*	0	4	5	9	6	13
*PNF*	No	Yes	Yes	No	No	No
*Lenght of surgery (h)*	11,5	6	9	14	11	9
*Duration of MV (h)*	168	75	144	336	150	180
*Tracheostomy*	Yes	No	No	No	No	Yes
*RBC U IO*	36	18	19	11	10	6
*FFP U IO*	23	14	8	5	10	5
*PLT U IO*	9	5	3	2	2	0
*Norepinephrine*	Yes	Yes	Yes	Yes	Yes	Yes
*Mycophenolate*	Yes	Yes	Yes	Yes	Yes	Yes
*Tacrolimus*	Yes	Yes	Yes	Yes	Yes	Yes
*Corticosteroids*	Yes	Yes	Yes	Yes	Yes	Yes
*Serum CPK (U/L)*	74	113	239	< 15	2000	80
*Rejection*	No	No	No	No	No	No
*Infection site*	Blood	Blood+BAL	Blood	Urine	Blood+BAL	Blood+Ascites
*ICUAW (days post LT)*	14	8	8	8	3	13
*ICUAW recovery*	Yes	Yes	Yes	Yes	Yes	Yes
*Pre-LT CVVH*	No	Yes	No	Yes	Yes	Yes
*Post-LT CVVH*	Yes	Yes	Yes	Yes	Yes	Yes
*LOS in ICU (d)*	79	14	20	20	16	53
*LOS in hospital (d)*	187	53	68	85	83	177
*90 days outcome*	Alive	Alive	Alive	Alive	Alive	Alive

*Abbreviations: Pt* patient, *MELD* Model for End stage Liver Disease, *D-MELD* Donor age for MELD, *SAPS-II* Simplified Acute Physiology Score II, *LT* liver transplantation, *PNF* Primary non function, *MV* mechanical ventilation, *RBC U IO* intraoperative red blood cells units, *FFP U IO* intraoperative fresh frozen plasma units, *PLT U IO* intraoperative platelets units, *CPK* Creatine Kinase, *BAL* Broncho-Alveolar Lavage, *PO* post operative, *CVVH* continuous veno-venous hemofiltration, *LOS* length of stay

### A82 A picture of orthotopic liver transplantation candidates with concurrent colonization by multi-drug resistant organisms: a preliminary retrospective study

#### F. Cundari ^1^, M. Monfroni ^1^, R. Taddei ^2^, G. Licitra ^3^, F. Forfori ^4^, G. Biancofiore ^2^

##### ^1^ Scuola di specializzazione anestesia e rianimazione Pisa, Pisa, Italy; ^2^ UO Anestesia e Rianiamazione dei Trapianti AOUP, Pisa, Italy; ^3^ SOD Anestesia e Terapia del Dolore, Pisa, Italy; ^4^ UO Anestesia e Rianimazione Interdipartimentale, Pisa, Italy

###### **Correspondence:** F. Cundari

*Journal of Anesthesia, Analgesia and Critical Care 2023,* **3(Suppl 1):**A82

Background

Patients undergoing orthotopic liver transplantation (OLT) require immunosuppression to prevent graft rejection, raising concerns about the risk of postoperative infections, particularly when they are colonized by multi-drug resistant organisms (MDROs). The detailed impact of MDRO colonization on postoperative outcomes in liver transplantation has not yet been fully determined.

Objectives

We examined the postoperative acquisition rate of bacterial infections and outcomes in patients who were colonized with MDROs at the time of OLT.

Materials and methods

A retrospective analysis was conducted in our center on liver transplant patients between January 2020 and February 2023. The incidence of colonization by MDROs was assessed through surveillance swabs performed on the day of OLT. All the patients with ongoing systemic infections and undergoing re-OLT were excluded. We compared 3 groups: patients who did not develop infections, patients who became infected by the colonizing germ, and patients who developed an infection determined by different germs. The study investigated the differences among the three groups in Model for End-Stage Liver Disease (MELD) score, ICU and in-hospital length of stay (LOS), and in-hospital mortality. Continuous variables were described using median and interquartile range, while categorical variables were presented as numbers and percentage of occurrence. We used Kruskal-Wallis test as a non-parametric test to compare the medians of the 3 independent groups. Given the retrospective nature of the analysis, the need for obtaining informed consent did not arise. Due to the retrospective nature of this study the need for informed consent was waived.

Results

18 of 413 OLT patients were positive for at least one MDRO colonization (4.4%): 3 MRSA, 4 MLSb, 8 CTX-M, 4 VRE, 3 CRE. Among the colonized patients, 4 (22.2%) developed an infection with isolation of the same MDRO during the hospitalization (table 1).

The median ICU stay was homogenous among the groups. Median in-hospital LOS was comparable for patients who did not develop infections and those who became infected with the same MDRO (13.5 vs. 13 days), while it was longer (23.5 days) for patients infected by a different pathogen, although without reaching statistical significance (table 2, figure 1).

Conclusions

This small preliminary study depicts an image of patients colonized by MDROs undergoing OLT in our center. Given the small numbers, the study is not designed or powered to draw conclusions on the safety and the risks of OLT in colonized patients. A larger randomized trial comparing this population with uncolonized patients is needed to obtain further results.

**Table 1 (abstract A82).** Clinical data on liver transplant recipients detected to be colonized by MDROs. Other: Infection from a pathogen other than the one of colonization. MRSA: methicillin resistant staphylococcus aureus, KPC klebsiella pneumoniae carbapenemase, MLSb: Macrolide-lincosamide-streptogramin B, CTX-M CefoTaXime-Munich, VanA/VanB: Vancomycin resistance type A/B, OXA-48: oxacillinase-48, ICU-LOS: intensive care uniti length of stay

Age	Sex	Etiology	MELD	Colonization	Infection	ICU LOS	Hospital LOS
73	M	Hepatitis C-Virus	13	MRSA	Yes (MRSA)	4	14
58	F	Hepatitis B-, hepatitis D-Virus	20	MRSA	Yes (MRSA)	5	12
48	F	Primary Biliary Cholangitis	15	KPC, MLSb	Yes (KPC)	3	16
45	F	Alcohol related	9	CTX-M	Yes (CTX-M)	7	13
64	M	Hepatitis C-Virus	6	MLSb	No	2	8
24	M	Primary sclerosing cholangitis	20	MLSb	No	5	10
59	M	Hepatitis B-Virus	10	KPC	No	7	14
56	F	Hepatitis B-Virus	8	CTX-M	No	6	12
58	M	Non-alcoholic Steatohepatitis	8	CTX-M	No	3	7
50	M	Hepatitis B-Virus	17	CTX-M	No	6	23
70	M	Primary sclerosing cholangitis	12	CTX-M / VanA	No	5	14
42	M	Primary sclerosing cholangitis	30	OXA48, VanA, CTX-M	No	6	26
19	M	Primary sclerosing cholangitis	16	MLSb	Yes (other)	3	23
48	M	Hepatitis B-Virus	7	VanB	Yes (other)	5	11
64	M	Hepatitis C-Virus	24	CTX-M	Yes (other)	5	24
52	M	Caroli disease	9	CTX-M	Yes (other)	3	24
62	M	Non-alcoholic Steatohepatitis	16	MRSA	Yes (other	5	46
65	F	Hepatitis B-, hepatitis D-Virus	11	VanB	Yes (other)	4	14

**Table 2 (abstract A82).** Comparison among the 3 study groups. Data are expressed as the median and the interquartile range

	Infection from MDROs colonization	No infection	Infection by different germs	p-value
**Age**	53 [19]	57 [15.5]	57 [16]	0,995
**MELD**	14 [6,5]	11 [10,5]	13,5 [7]	0,891
**Hospital LOS**	13,5 [2.5]	13 [9,5]	23,5 [10]	0,198
**ICU LOS**	4,5 [2,5]	5,5 [2]	4,5 [2]	0,46

All the patients were discharged, with no in-hospital mortality

**Fig. 1 (abstract A82).**
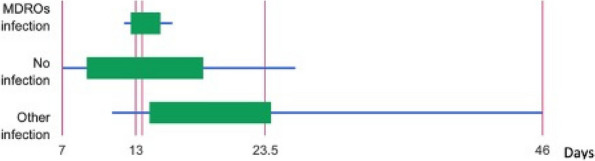
See text for description

## Underwater and Hyperbaric Medicine

### **A83 Carbon monoxide poisoning in eastern Piedmont: preliminary data of a prospective observational study**

#### A. Puggioni ^1^, G.L. Vignazia ^2^, S. Zorzi ^1^, A. Burgener ^1^, G. Romani ^1^, P.P. Miletta ^1^, M. Zuliani ^1^, R. Perucca ^2^, S. Guido ^2^, T. Cena ^2^, S. Bazzano ^2^, F.L. Barra ^2^, C. Antonini ^2^, C. Olivieri ^3^, C. Maestrone ^4^, F. Racca ^5^, F. Della Corte ^1^, G. Cammarota ^1^, A. Giovanniello ^6^, R. Vaschetto ^1^

##### ^1^ Dipartimento di Medicina Traslazionale, Università del Piemonte Orientale, Novara, Italy; ^2^ Anestesia e Rianimazione, A.O.U. Maggiore della Carità, Novara, Italy; ^3^ Anestesia e Rianimazione, Presidio Ospedaliero S. Andrea, Vercelli, Italy; ^4^ Anestesista e Rianimazione, ASL VCO, Domodossola, Italy; ^5^ Anestesia e Rianimazione, AON SS. Antonio e Biagio e Cesare Arrigo, Alessandria, Italy; ^6^ Centro Iperbarico Habilita, Fara Novarese, Italy

###### **Correspondence:** A. Puggioni

*Journal of Anesthesia, Analgesia and Critical Care 2023,* **3(Suppl 1):**A83

Background

Carbon monoxide (CO) poisoning is a common and life-threatening intoxication potentially leading to delayed neurological sequelae (DNS). This study aims to describe incidence, clinical characteristics and outcomes of patients with CO poisoning and indications to hyperbaric oxygen therapy (HBOT).

Materials and Method

This multicentric observational study includes patients with an age >18 years old and indications to HBOT due to acute CO poisoning entering eastern Piedmont hospitals from September 2022 to May 2023. At hospital entry and after hyperbaric treatment, clinical variables, and Pfeiffer’s test were registered. Forty-five-day outcomes were evaluated by phone interviews.

Results

Thirty-nine subjects suffered from symptomatic CO poisoning during the study period. Nine patients were excluded as underage while two for consent withdrawn. One patient, with a past medical history of recurrent pneumothorax, resulting in apicectomy and residual bubbles, was subjected only to normobaric oxygen therapy after balancing risks and benefits.

Patient median age was 54 (44;70) years and 14 (50%) were male. History of respiratory disease (n=9, 32%) and smoke (n=8, 29%) were the most frequent comorbidities. All patients had an accidental poisoning due to solid (n=16, 57%) or gas (n=12, 43%) fuel. Fifteen patients (54%) showed a grade 4 according to the Italian Society of Diving and Hyperbaric Medicine (SIMSI) intoxication scale.

Median CO hemoglobin (COHb) and lactate values were respectively 24.2% (17-28) and 1.9 (1.1-3.8) mmol/L on admission and 1.3% (0.98-1.75) and 0.7 (0.5-0.9) mmol/L after HBOT.

Median time of CO exposure was 12 (3-18) hours and the time from the referring sites to the hyperbaric facility ranged from 3 to 14 hours. Twenty patients (71%) were treated at 2.5 atmosphere absolute pressure (ATA) for 96 min, seven (28%) at 2.8 ATA for 60 min and at 2.5 ATA for other 58 min.

Before HBOT, Pfeiffer test was compatible with intact cognitive function in 20 patients (71%), mild decline in 5 (18%), and severe disfunction in 3 (11 %). Forty-five days after CO poisoning, Pfeiffer test was computed in 20 patients and was compatible with normal cognitive functions in 14 patients (70%) and mild decline in 4 (20%); two patients had language barrier, while one not candidate to HBOT developed DNS within 30 days. Eight patients were lost to follow-up.

Conclusion

Although preliminary, our data show a DNS incidence of 3.5%. Further studies are necessary to evaluate the pathophysiological mechanisms and the real incidence of DNS.

### A84 Hyperbaric Oxygen Therapy in elderly: is it safe enough?

#### A.N. Cracchiolo, D.M. Palma, M. Palmeri, D. Tantillo, R. Lo Bue, A. Teresi, G. Re, B. Bonanno, C. Riccobono, M. Lo Brutto, R. Barbiera, C. Palazzolo, N. Lepore, G. Di Fresco, C. Incandela, L. Venezia, F. Genco

##### ARNAS Civico, Palermo, Italy

###### **Correspondence:** A.N. Cracchiolo

*Journal of Anesthesia, Analgesia and Critical Care 2023,* **3(Suppl 1):**A84

Introduction: Historically, a person who passed the age of 65 is considered elderly. In 2018, the Italian Society of Gerontology and Geriatrics proposed a new dynamic definition of seniority, moving the threshold to 75 years. The increase in the average age of the Italian population has increased the number of patients for whom assessment for suitability and hyperbaric oxygen therapy (HBOT) is required.

Aim: assess in over 75 years old patients indications for HBOT, pre-existing conditions, feasibility of treatment and adverse events that occurred.

Method: Retrospective analysis of data on patients over 75 treated at the multi-site hyperbaric chamber of ARNAS Civico-Palermo, during the period from April 2021 to May 2023. Each patient was required to undergo instrumental assessments (chest X-ray, ECG, echocardiograms when deemed necessary, ENT assessment with impedance measurement) and laboratory tests (blood count, blood glucose, blood electrolytes, azotemia, creatinine) before the start of HBOT. They also underwent a clinical evaluation performed by a physician specialising in hyperbaric medicine. All patients underwent HBOT performed at 2.5 atmospheres absolute (ATA) in adherence to the guidelines of the Italian Society of Underwater and Hyperbaric Medicine (SIMSI). The treatments were performed once a day for five days a week, total oxygen time for each session of 1 hour. Assessment of vital parameters (non-invasive blood pressure, heart rate, transcutaneous oxygen saturation, body temperature) was performed at the start and end of each HBOT session. Subjects with absolute contraindications to HBOT (claustrophobia, previous spontaneous pneumothorax, bullous emphysema, recent uncontrolled epileptic seizures, taking specific drugs) were excluded from treatment.

Results: From April 2021 to April 2023, our hospital's hyperbaric centre treated 220 patients, the number of patients over 75 enrolled was 32 (15% of the whole population, of whom 19 were male), the average age was 79 years old. A total of 4584 HBOT sessions were delivered and the number of sessions performed for this cohort was 955 (20.83% of the total). The indications for HBOT and associated pathologies found in treated patients are shown in figures 1 and 2 respectively.

All patients showed a high level of compliance with the treatment without complaining of problems during the various sessions they underwent with the exception of some sporadic difficulties with the compensation (4 times out of a total of 955 sessions). No adverse events were observed.

Conclusion: As highlighted by other works 1, we may state that the use of rigorous operating protocols, shared with all staff, based on the careful evaluation of the mandatory clinical-instrumental investigations for HBOT to be performed before enrolling the patient, the evaluation of vital parameters before and after each treatment, together with clear comprehensive and peaceful communication with the patients, are the cornerstones that make HBOT a fundamentally safe medical treatment.

I declare toI have collected informed consent from all patients recruited.

References

A) A Hadanny, O Meir, Y Bechor. The safety of hyperbaric oxygen treatment - retrospective analysis in 2,334 patients. UHM 2016, Vol. 43, No. 2

**Fig. 1 (abstract A84).**
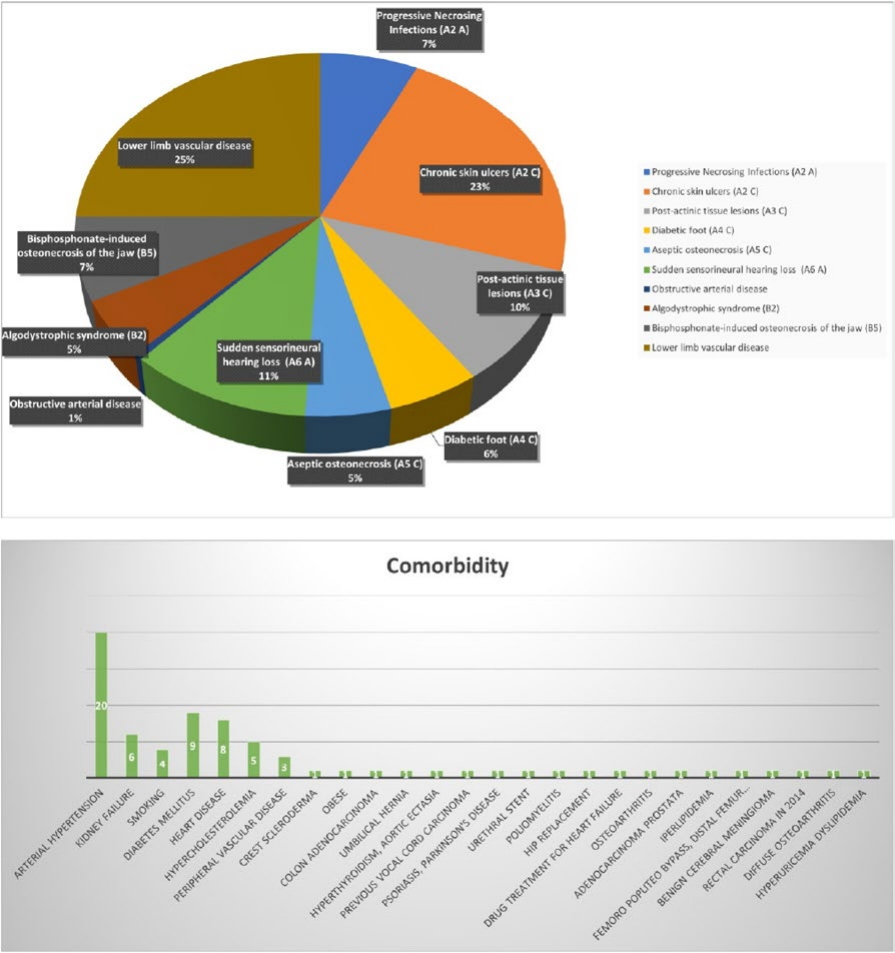
See text for description

### A85 Arterial/Alveolar pO2 ratio in Arterial Blood Gases of SCUBA Divers at Depth

#### T.A. Giacon ^1,2^, M. Paganini ^2^, L. Zucchi ^3^, S. Mrakic-Sposta ^4^, L. Martani ^5^, G. Garetto ^6^, G. Bosco ^2^

##### ^1^ Institute of Anesthesia and Intensive Care, University of Padova, Padova, Italy; ^2^ Department of Biomedical Sciences, University of Padova, Padova, Italy; ^3^ Division of Emergency Medicine, University of Padova, Padova, Italy; ^4^ Institute of Clinical Physiology, National Research Council (CNR), ASST Grande Ospedale Metropolitano Niguarda, MIlano, Italy; ^5^ Anesthesia and Intensive Care Unit, Vaio Hospital, Fidenza, Italy; ^6^ ATIP Hyperbaric Medical Center, Padova, Italy

###### **Correspondence:** T.A. Giacon

*Journal of Anesthesia, Analgesia and Critical Care 2023,* **3(Suppl 1):**A85

Background: current diving physiology postulates that SCUBA divers at depth experience arterial blood gas (ABG) levels variations proportional to environmental pressure. A recent systematic review [1] demonstrated that arterial partial pressures of oxygen (PaO2) under hyperbaric conditions can be predicted from PaO2 measurement at 1 atmosphere absolute (ATA) assuming a constant arterial/alveolar PO2 ratio (a:A). However, regarding SCUBA divers, this systematic review only retrieved simulated dives. This work describes ABGs obtained for the first time in SCUBA divers at depth and aims to verify the validity of a:A ratio in predicting PaO2.

Methods: the study was approved by the local ethics committee. After placing an arterial cannula on the non-dominant limb [2,3], ABG samples were obtained at four steps (Figure 1): at surface before the dive (A); at depth (-15 m or -42 m) before (B) and after (C) a standardized exercise; at surface (D). The underwater exercise consisted of pedaling on a submersed bicycle set at 100 W (plus 50 W to move legs underwater = 150 W total effort) at a rate of 60 rpm for 10 minutes. After calculating alveolar PO2 at surface (1 ATA) as previously reported [1], the a: A was obtained and used to predict PaO2 at depth. The measured PaO2 was plotted against the predicted PaO2, using Spearman’s rho; a linear regression between measured and predicted PaO2 was reported to assess significance of the results, along with the goodness-of-fit F test.

Results: 6 subjects performed the dive at -15 m, and other 2 at -42 m. The PaO2 increased as predicted at both depths, and more at -42 than at -15 m (Figure 1), without differences before and after the exercise at -15 m (p= 0.519). The a:A calculated from the baseline ABG obtained before the dives at rest, out of the water, seemed to adequately predict the PaO2 in the other conditions (r = 0.939, p<0.0001; Figure 2).

Discussion: ABGs have been obtained for the first time in SCUBA divers in real underwater conditions, confirming the predicted rise in PaO2. Also, the a:A ratio showed to accurately predict the PaO2 at depth; however, as showed in the plot (Figure 1) the a:A ratio seems to lose accuracy at higher depths and higher partial pressures, as already emerged in the previous systematic review. Such results should be verified in the future on a wider cohort and possibly during different diving conditions (e.g., using closed-circuit rebreathers or breathing gas mixtures).

**Fig. 1 (abstract A85).**
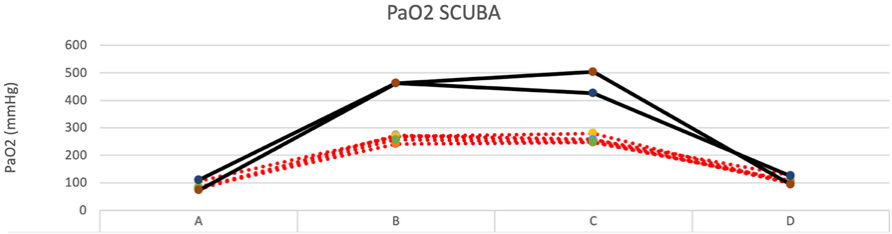
See text for description

**Fig. 2 (abstract A85).**
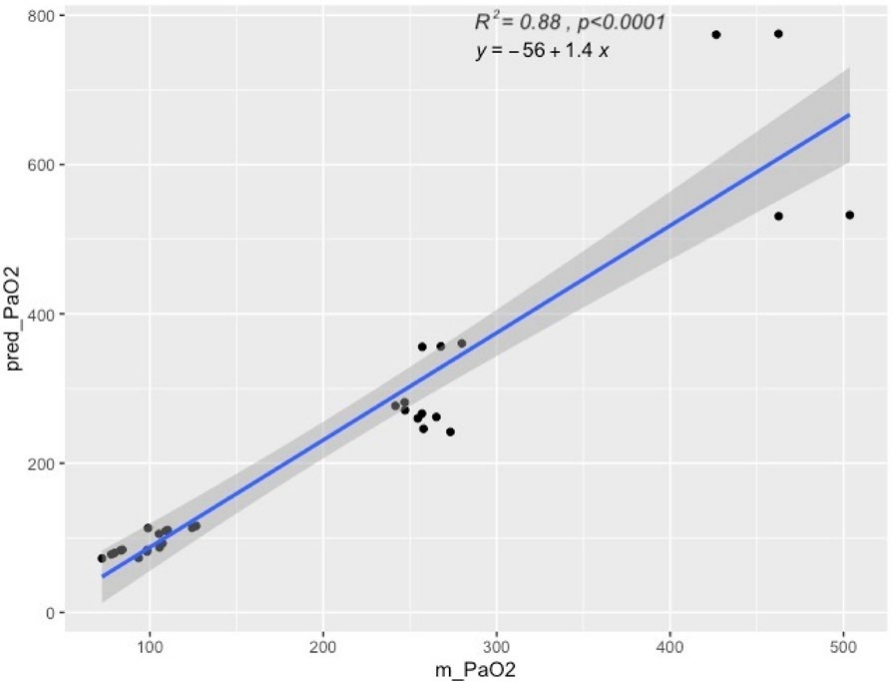
See text for description

## Follow-up/outcomes

### **A86 Changes in incidence of nosocomial infections pre- and post- new bundles adoption**

#### E. Lai, A. Busia, M. Muceli, G. Olla, A. Orru', A. Paddeu, S. Paba, S. Pilloni, M.V. Piroddi, S. Serdino, A. Usai, F.M. Loddo

##### Ospedale Nostra Signora Della Mercede - SC Anestesia E Rianimazione - ASL Ogliastra, Lanusei, Italy

###### **Correspondence:** E. Lai

*Journal of Anesthesia, Analgesia and Critical Care 2023,* **3(Suppl 1):**A86

Background: in ICU, prevalence of infection is estimated to be 30% worldwide, which is a major cause of morbidity and mortality, increased length of hospital stay and hospital costs.1,2

Materials and methods: in our ICU, a bundle to control the spread of infections has been adopted:

isolation for 72 hours from time of admission regardless of the type of patient; systematic cultures, fundamental to obtain evidences for epidemiology, antimicrobial strategies and the control of multidrug-resistant clusters; hand hygiene, personal protective equipment and use of dedicated or disposable equipment; careful monitoring of sanitation processes, correct management of linen and disposal of medical waste according to company guidelines in order to prevent the spread of microorganisms among patients, staff and the environment; health personnel dedicated to the single infected patient.3 Many of these practices were strictly implemented since the advent of SARS-CoV-2 in suspected or infected patients; subsequently, through an analysis of medical records, culture tests results (2021-2022) of the hospitalized patients were evaluated. Data from 2021 and 2022, related to the hospitalization hours (hospitalization > 48 h), were compared. To confirm the statistical significance of the data obtained, the T-test was calculated.

Results: these data show a statistically significant reduction in the incidence of ICU-related nosocomial infections (18 in 2022 vs 36 in 2021, p value < 0,05) following the implementation of these bundles in the absence of a statistically significant difference in the total length of hospitalization (1077 days of hospitalization in 2022 vs 1163 days of hospitalization in 2021, p value > 0,05) between the years 2021 and 2022.

Conclusions: our experience shows how the strictly maintenance of the ICU-bundle practices introduced to fight against Covid-19 pandemic, can lead to the reduction of nosocomial infections, even non-Covid related, in intensive care units.

References

1. Araç E, et al. Evaluation of Infections in Intensive Care Units: A Multicentre Point-Prevalence Stud. Mikrobiyol Bul. 2019 Oct;53(4):364-373.
2. Dror Marchaim, et al. Nosocomial infections in the intensive care unit: Epidemiology and prevention. In: UpToDate, Post TW (Ed), UpToDate, Waltham, MA. Feb 2023.
3. Deverick J Anderson et al. Infection prevention: Precautions for preventing transmission of infection. In: UpToDate, Post TW (Ed), UpToDate, Waltham, MA. Feb 2023.

### A87 Nursing and surgical management in stage IV pressure ulcers: case report

#### S. Paba, A. Busia, E. Lai, M. Muceli, G. Olla, A. Orru', S. Pilloni, A. Paddeu, M.V. Piroddi, S. Serdino, A. Usai, F.M. Loddo

##### Ospedale Nostra Signora Della Mercede - SC Anestesia E Rianimazione - ASL Ogliastra, Lanusei, Italy

###### **Correspondence:** S. Paba

*Journal of Anesthesia, Analgesia and Critical Care 2023,* **3(Suppl 1):**A87

Background: pressure-induced skin and soft tissue injuries are the result of sustained force on soft tissue typically overlying bony prominences, which leads to hypoxia, ischemia, and eventual necrosis.1 They are areas of localized damage to the skin and/or underlying tissue, due to pressure alone or pressure in combination with shear forces. In more than 90% of cases, the pressure-induced injury was not the original cause of admission. The DecubICUs study reported a prevalence of 22,8% with an acquired rate of 13.3% in North American ICUs.2

Case report: a 55-year-old patient was admitted to our ICU after a long hospitalization for cerebral hemorrhage, followed by Covid-19 pneumonia. Upon arrival, the patient presented a stage IV sacral pressure ulcer, surgically treated with a skin graft that failed. The lesion was evaluated through the B-WAT scale and was assigned a score of 49 pt. After a surgical consultation, the graft was removed and the VAC therapy was placed. In addition, a colostomy was performed, which allowed to benefit by improving the toileting quality the wound.1 Following VAC therapy, the lesion improved and presented with abundant granulation tissue, achieving a score of 32 pt on the B-WAT scale. The lesion was treated with Polyurethane foam and Hydrogel and the decubitus was changed every two hours. After surgical consult, we were opted for the paraspinal transposition flap technique. 3 With this technique, the large sacral defect was reconstructed using single flap.

Conclusions: our case shows that this surgical technique, combined with frequent surgical dressings and repositioning every 2 hours, allows a good recovery of the wound. Careful nursing care after the surgery was required, and allowed the lesion to improve. Following these recommendations, the tissue appeared healthy and well vascularized without signs of infection.

Informed consent to publish had been obtained.

References

1. Alan D Rogers, et al. Surgical management of pressure-induced skin and soft tissue injuries. In: UpToDate, Post TW (Ed), UpToDate, Waltham, MA. Aug 26, 2022.
2. Labeau SO, et al. Prevalence, associated factors and outcomes of pressure injuries in adult intensive care unit patients: the DecubICUs study. DecubICUs Study Team, European Society of Intensive Care Medicine (ESICM) Trials Group Collaborators Intensive Care Med. 2021;47(2):160. Epub 2020 Oct 9.
3. Sandipan Guptal, et al. Paraspinal Transposition Flap for Reconstruction of Sacral Soft Tissue Defects: A Series of 53 Cases from a Single Institute. Asian Spine J 2014;8(3):309-314.

## Infections and sepsis

### **A88 Combined antifungal therapy in blood candidiasis in a young street polytrauma**

#### M. Toma, F. Pagliara, D. Puscio, G. Pulito

##### Dipartimento Anestesia e Rianimazione, P.O. Vito Fazzi, Lecce, Italy

###### **Correspondence:** M. Toma

*Journal of Anesthesia, Analgesia and Critical Care 2023,* **3(Suppl 1):**A88

The patient is the victim of a road accident on 25 June 2022 in Basilicata. He is transferred by air ambulance to the San Carlo Hospital in Potenza. In the Total body CT, among other things, a pelvis fracture is reported (pubic symphysis diastasis and fracture of the left iliac wing). On the same day, a T Pod is placed in the operating room. After having performed a consultation with the orthopedics of Lecce, a specialized center for pelvic surgery, the patient is transferred to the CPR of Lecce on 4 July 2022. On admission, the patient's clinical picture appears very complex: present shock, hemodynamics sustained by amines, hyperpyrexia, significant leukocytosis, PCT, CRP and lactate increase (imm 1). A blood sample is then taken for blood culture. A whole body CT with contrast medium is performed again, which highlights a picture of pulmonary embolism. The resulting color Doppler ultrasound of the lower limbs shows a deep vein thrombosis for which a caval filter will then be placed. On 7 July 2022, the blood culture of 4 July 2022 was reported: presence of Candida Albicans in blood isolates taken from central, peripheral and arterial venous catheters with consequent susceptibility test. After consulting with the infectious disease specialist, blood cultures were taken again, Mannano was searched for and therapy was started with Caspofungina 70mg/day after loading dose. On 13 July 2023 the blood culture of 7 July 2023 was reported where Candida Albicans was isolated in the blood sample from the central vein and the Mannano result was borderline. At the umpteenth TT echocardiographic check, the presence of endocarditis confirmed with TE echo was suspected, describing a mass of 0,4 cm adherent to the coronary sinus of possible fungal aetiology. Liposomal Amphotericin B 3mg/kg/h is added to Caspofungin. The blood culture is then repeated and serum candida antigens are monitored. In the following days, the patient's clinical picture improves markedly, the amines to support the hemodynamics are suspended, the white blood cells, CRP and PCT normalize and blood cultures negative for Candida Albicans are then reported (imm 2). The following echocardiographic checks would also be negative. Double antifungal therapy is continued for 4 weeks.

Informed consent to publish had been obtained

**Fig. 1 (abstract A88).**
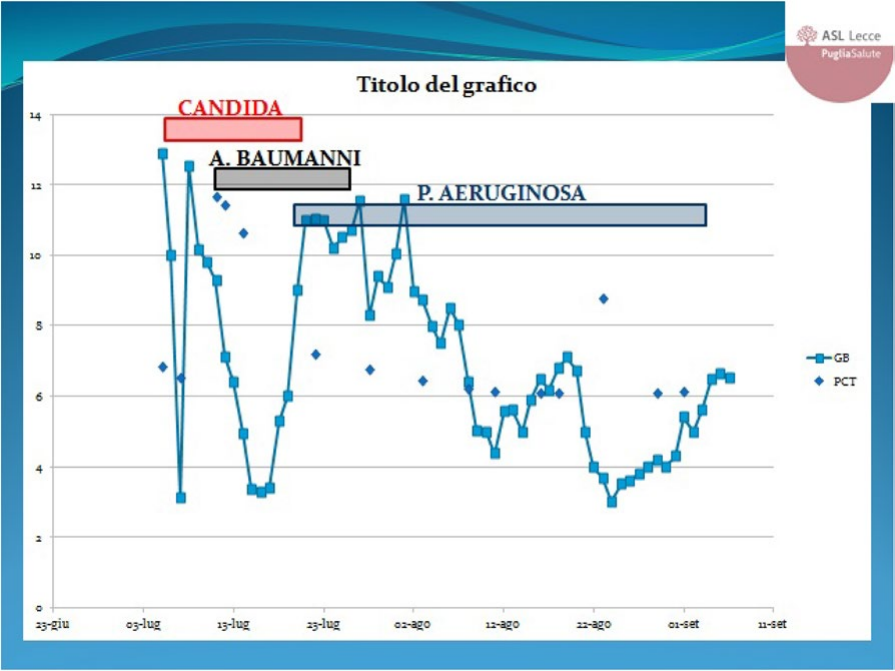
See text for description

### A89 Use of Argipressin in a refractory septic shock – a case report-

#### J.L. Russo, E. Panascia, D. Rinzivillo

##### Azienda Ospedaliera Universitaria Policlinico Gaspare Rodolico San Marco, Catania, Italy

###### **Correspondence:** J.L. Russo

*Journal of Anesthesia, Analgesia and Critical Care 2023,* **3(Suppl 1):**A89

A fifty-nine-years-old female patient came the emercency department for fever and dyspnea, after a chest CT scan diagnosis of left pleural empyema was done and two pleural drainages on the left side were inserted. The patient had a sudden clinical deterioration and she was admitted to ICU with diagnosis of septic shock. Comorbidities included: arterial hypertension cigarette smoking and history of dental absesses. Patient was sedated and intubated, mechanically ventilated with high support and poor gas exchanges. Hemodinamics was supported by noradrenaline 0.33 mcg/kg/min and, as not improving, after argipressin has been introduced at incremental doses from 0.01 UI/min to 0.02 UI/min. Renal disfunction occurred requiring hemofiltration with anticytokines filters. Targeted antimicrobial therapy was started because were positive for central and peripheral line, from bronco aspirate and PTC elevated values. All line were changed and Meropenem- Vaborbactam – Vancomicina and Cancidas has been administered. Five days later slow until wean off of Norepinephrine was achieved and 3 days later argipressin weaning off was possible too. On the 14th days the patient Blue Rhino percutaneous tracheostomy got with Ciaglia Blue Rhino thecnic. After two days cultures and PCT were negative. The patient was clinically improved with no support requiered. Also the CT chest- abdomen showed mediastinal and abdominal abscesses resolution. On the 18th day the patient was transferred to the Respiratory HDU.

I authorize the publication of this case report.

Conclusion

The Use of Argipressin in a case of septic shock refractory to amines has proved effective in the weaning of inotropes.

Informed consent to publish had been obtained

**Fig. 1 (abstract A89).**
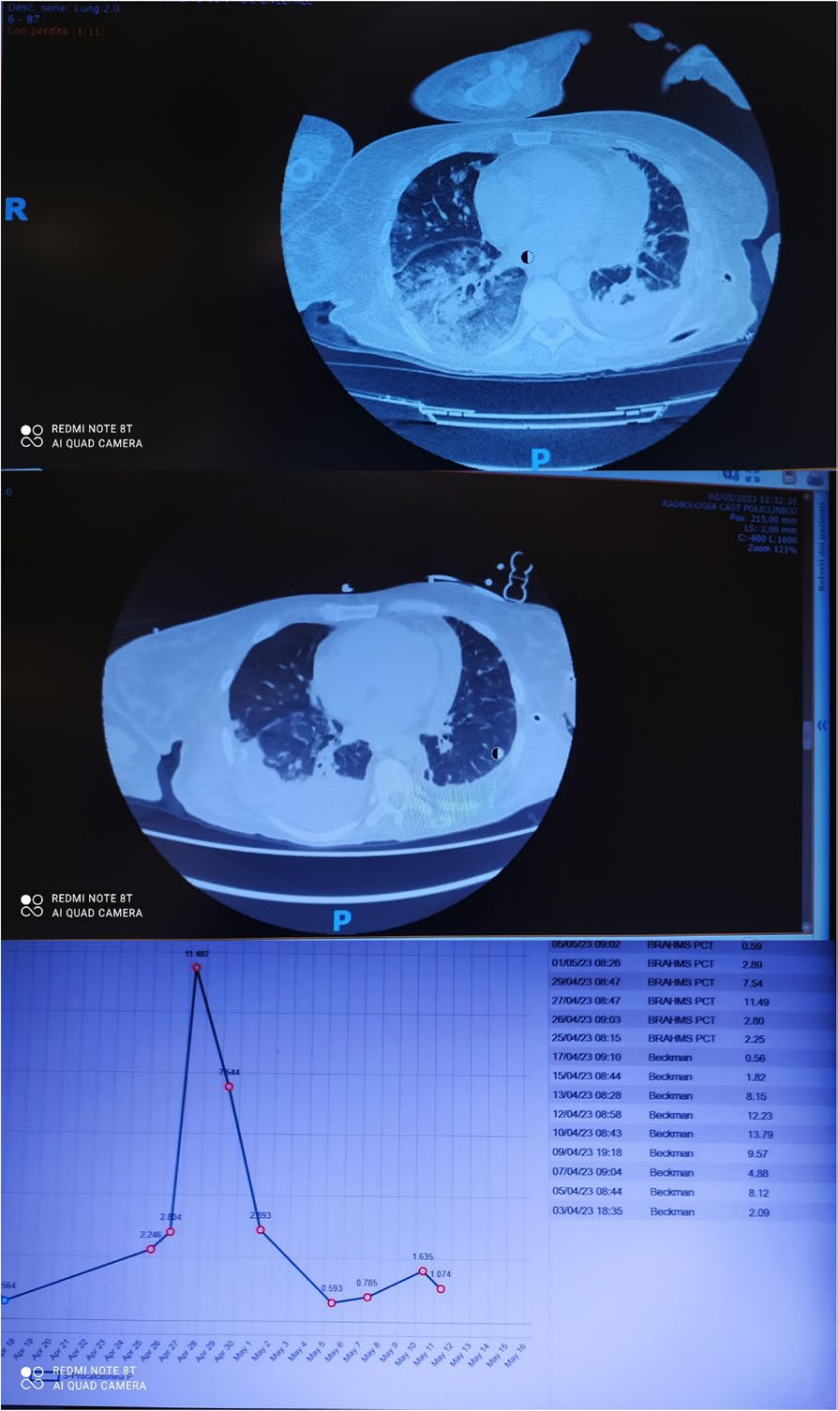
See text for description

### A90 Intensive care unit and medical department: differences in patients with candidemia. A case report and our hospital data

#### V. Quaranta^1^, P. Gnesin^2^, E. Cogi^2^

##### ^1^Università degli Studi di Brescia, Brescia, ITALY, ^2^ASST Franciacorta, Anestesia e Rianimazione, Chiari, ITALY

###### **Correspondence:** V. Quaranta

*Journal of Anesthesia, Analgesia and Critical Care 2023,* **3(Suppl 1):**A90

Background

Candidemia is the most frequently detected type of invasive candida infection. It is the fourth leading cause of nosocomial bloodstream infections in the United States of America (USA) and the seventh in Europe [1]. The overall mortality rate of candidemia is 22–75% [2].

Case report

A 61-year-old woman was admitted to the Emergency Room (ER) for cervicalgia and food vomiting (history of hypertension, diabetes and previous cerebral ischemia); she was discharged with diagnosis of muscular cervicalgia and decompensated diabetes.

The following day she returned for persistence of symptoms; an abdominal ultrasound documented abundant intraperitoneal effusion suspected for ascites in the setting of possible peritoneal carcinosis. The patient, paucisintomatic and normothermal, was admitted to the Medical Department. Few hours later the clinical-laboratory scenario worsened suddenly, an abdominal CT was performed showing an intestinal perforation; she went under surgery to suture a duodenal ulcer. After surgery she was admitted to the ICU due to septic shock. Broad spectrum antibiotic therapy was immediately initiated with piperacillin-tazobactam, amikacin and metronidazole. Also an empirical antimycotic therapy with caspofungine was started; meanwhile, blood cultures documented a candidemia from Candida Glabrata (caspofungine antifungal resistant). Therefore Amphotericin B was started.

The clinical course and the recovery were very slow. A tracheotomy was performed and she had a second surgery due to a duodenal fistula (Candida Glabrata was also present in the peritoneal fluid). A duodenal prothesis and an esophageal stent were placed for a peptic stenosis.

The antifungal therapy had been continued for 30 days since the onset of invasive candidemia, with no endocarditis or eye lesions. The patient was hospitalized for 65 days, 35 of which in ICU.

Conclusion

We analyzed the data of patients admitted to our ICU within 15 months with a diagnosis of invasive candidemia. There were 4 patients out of 305; in 50% of cases candidemia was present at admission (post-surgical patients with intestinal perforation). The observed mortality rate was 50%. Candida was multisensitive in only 25% of cases. Concurrent bacterial isolations were present in 100% of cases. In the same period, in the non-intensive area, we recorded 17 patients with candidemia. In 23% of cases (fragile patients, carriers of devices, non-surgical) the cultures were positive at admission. The antimicogram was multi-sensitive in 94% of cases, with concomitance of bacterial positivity in 47%. However, mortality was at 41%, higher than the mean mortality of the non-intensive area.

The authors certify that they have obtained all appropriate patient consent forms.

References

1. Cuervo G, Garcia-Vidal C, Puig-Asensio M, Merino P, Vena A, Martín-Peña A, Montejo JM Ruiz A, Lázaro-Perona F, Fortún J, et al.. Usefulness of guideline recommendations for prognosis in patients with candidemia. Med Mycol 2019; 57:659–667.
2. Liu f, Zhong L, Zhou F, Zheng C, Zhang K, Cai J, Zhou H, Tang K, Dong Z, Cui W et al.. Clinical features, strain distribution, antifungal resistance and prognosis of patients with non-albicans candidemia: a retrospective observational study. Infect Drug Resist, 2021, 14:3233–3246.

### A91 Role of MR-proADM in critically ill patients: a case series study

#### M. Pisoni, L. Pistidda, A. Mascotti, D. Pasero, P. Terragni

##### Department of Anesthesia and Intensive Care, AOU Sassari, Sassari, Italy

###### **Correspondence:** M. Pisoni

*Journal of Anesthesia, Analgesia and Critical Care 2023,* **3(Suppl 1):**A91

Background

Sepsis and septic shock are still nowadays one of the hardest challenges for intensivist. As a matter of fact, the mortality rate ranges from 15-25% [1]. Sepsis is life-threatening organ dysfunction caused by a dysregulated host response to infection [2]. The key to the treatment is to find a biomarker that could be an early predictor of the prognosis and the severity of organ dysfunction. MR-proADM can potentially reflect the severity of organ dysfunction, even in the first stages of the disease, in the progression of systemic inflammatory response, in the evolution from sepsis to septic shock, and in the mortality risk of septic patients [3,4].

Case series:

Male 80y admitted to our service for septic shock after urgent surgical procedure for bowel obstruction. Empiric antibiotic therapy was started. In the follow

ing days PCT, MR-proADM got worse and an intestinal perforation was detected. After intestinal resection and sepsis source control, MR-proADM sharply declined (Figure 1). In this case the decrease of MR-proADM was inversely proportional to patient recovery.

Female 75y admitted for respiratory failure with acute pulmonary edema in CKD. Empiric antibiotic therapy was started. Despite the patient during recovery didn’t show any infection symptoms, MR-proADM levels stayed high, maybe due to CDK (Figure 2).

Female 52y admitted for respiratory failure after pancreatoduodenectomy. During the recovery, despite the patient had fever and significant PCT rise in absence of surgical complication, MR-proADM stayed always under target level (Figure 3). Inflammatory indexes decreased after antibiotic escalation. In this case we can say that this patient developed an infection without organ failure.

Male 71y admitted for neurologic disease. Although during the recovery he had several thermic and WBC rises, PCT and MR-proADM were negative (Figure 4) so no Antibiotic therapy was started. In this case biomarkers guided physicians on antibiotic stewardship.

Male 74y admitted for epileptic seizures in neurologic unknown disease. During the recovery he had a sudden neurological and respiratory clinical worsening with a rise of MR-proADM and inflammatory indexes. Antibiotic escalation therapy was applied with an improvement of PCT and WBC but an increase of MR-proADM associated to clinical deterioration (Figure 5). Indeed, in the following days the patient developed peritonitis due to intestinal perforation. In this case MR-proADM helped the clinician to detect unknown and concomitant organ failure.

Conclusion

With these case series we show how MR-proADM is useful for the physician to better understand the evolution of sepsis and for antibiotics stewardship. In addition, MR-proADM is able to detect the severity of organs failure and can be also useful as a spy of unknown organ dysfunction when the typical biomarkers are negative.

Written informed consent for publication of their clinical details was obtained from the patient (BioMed Central consent form).

References

1. Piccioni A, et al. Proadrenomedullin in sepsis and septic shock: a role in the emergency Vol. 57, MDPI; 2021.
2. Doi K, Estenssoro et al, Sprung 57 Vol. 49. 2021.
3. Surviving Sepsis Campaign: International Guidelines 2021
4. Enguix-Armada A, et al. Clin Chem Lab Med. 2016 Jan 1;54(1):163–8.

**Fig. 1 (abstract A91).**
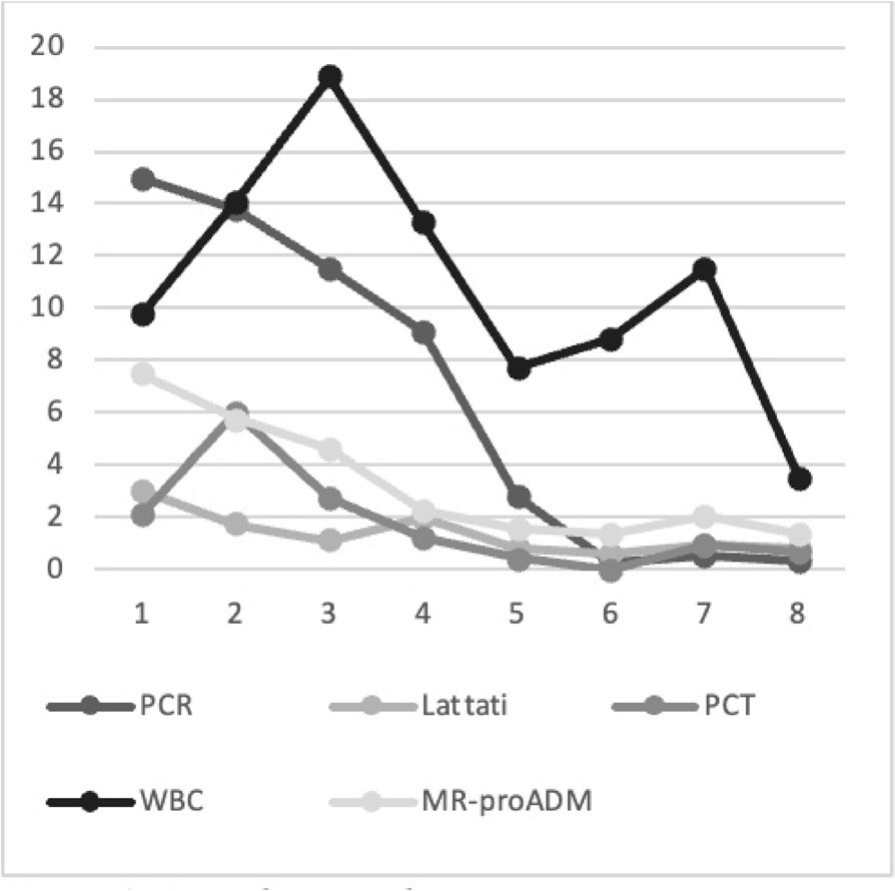
Biomarkers trend

**Fig. 2 (abstract A91).**
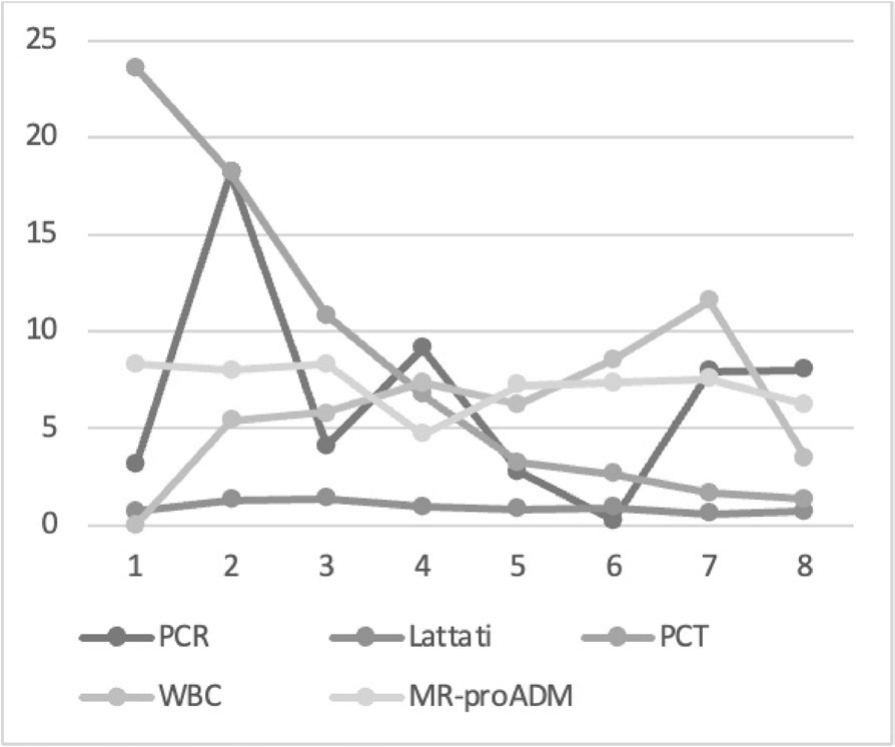
Biomarkers trend

**Fig. 3 (abstract A91).**
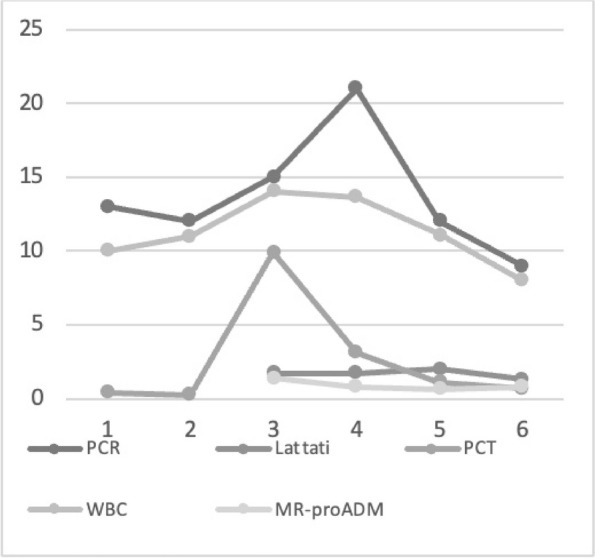
Biomarkers trend

**Fig. 4 (abstract A91).**
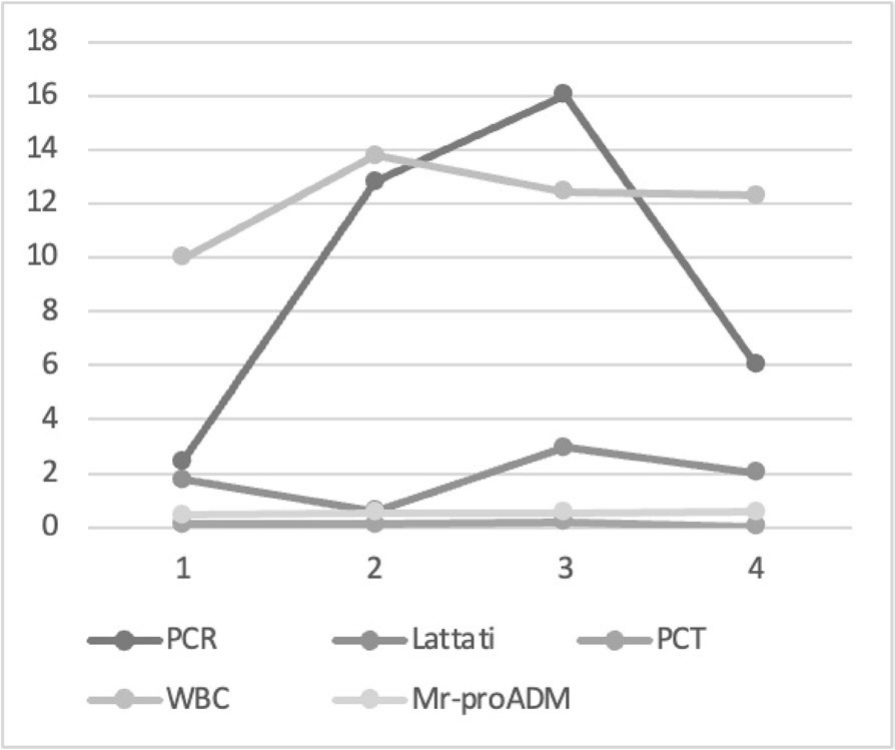
Biomarkers trend

**Fig. 5 (abstract A91).**
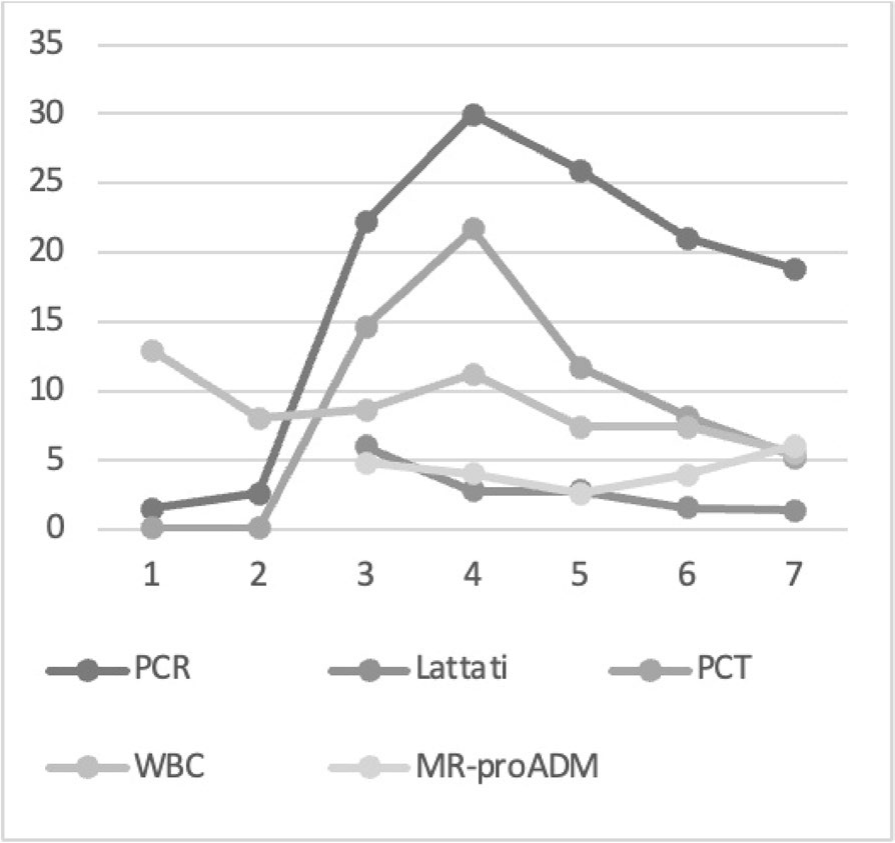
Biomarkers trend

### A92 Landilol in infective endocarditis

#### V. De Angelis ^1^, S. Verrengia ^1^, F. Claro ^1^, C.M. Petrangeli ^1^, P. Vitale ^2^, L. Dori ^2^, F. Leonardis ^1^

##### ^1^ UOSD Terapia Intensiva Policlinico Tor Vergata, Roma, ITALY, ^2^ UOC Malattie Infettive Policlinico Tor Vergata, Roma, Italy

###### **Correspondence:** V. De Angelis

*Journal of Anesthesia, Analgesia and Critical Care 2023,* **3(Suppl 1):**A92

Background

Infective endocarditis (IE) has high mortality rate despite antibiotic therapy and supportive care. It usually affects population at risk. IE is a clinical syndrome with cardiological symptoms but also with other signs and symptoms. It comes with neurological syndrome or septic shock, where the pharmacological approach for non-compensatory tachycardia improves cardiac performance, reducing the heart metabolic oxygen consumption.

Case report

Male, 62ys, weight 90 kg, height 180 cm, BMI: 27.8 kg/mq. Patient without comorbidities, two weeks before admission to ICU the patient underwent knee arthroscopy and in the last three days he had fever and general malaise treated with Paracetamol. He was moved to Tor Vergata University Hospital. The patient presented hemodinamic instability and coronaric acute syndrome. He had tonic clonic seizures with prolonged coma, so he was intubated and ventilated with protective mechanical ventilation (1). The transthoracic echocardiogram showed endocarditis of mitralic valve and severe valve insufficiency and an EF of the 40%. The brain CT scan showed a cerebral hemorrage. The laboratory tests showed neutrophilic leukocytosis (WB: 20.000/mm3; NEU %: 88), Total and direct Bilirubin 7/5.8 mg/dl, RCP 200 mg/L; PCT 12 ng/ml; Creatinine: 4 mg/dl; Adrenomedullin: 2 mmol/l. Hemodinamics was supported by Norepinephrine 0.4 mcg/kg/min with a blood pressure of 50/40 mmHg (MAP<65mmHg). Urinary output was < 0.5ml/kg/hr. The patient presented supraventricular tachycardia at 130 bpm. The Lactate dosage was > 2 mmol/l. Metabolic acidosis: pH 7.28; paCO2 40mmHg, BE: -8; Bicarbonates: 18mmol/l; paO2:100 mmHg (FiO2:0.9), SatO2:91%; SvO2: 60%. SOFA SCORE: 15. Diagnosis: IE with hemorrhagic stroke and septic shock.

The patient was admitted to ICU from the emergency department after ninety minutes. Lactate Ringer was administered at 30 ml/kg (2), improving hemodinamic parameters (CI 2 L/minute/mq, SVV 10%), but the blood pressure was low (50/40 mmHg) with tachycardia (130 bpm). For this reason, argipressin (0.01 UI/min to 0.03 UI/min) was associated. We started the association with norepinephrine at 0.1-0.2 mcg/kg/minute and with landiolol (1 mcg/kg/min to 3 mcg/kg/min in 30 minutes). Empiric antibiotic therapy prescribed: Vancomycin 15-20 mg/kg iv q12 h (dosage of vancocinemia every 48 hours) and Ceftriaxone 2 g q24 h. The blood cultures were performed in the emergency room.

Informed consent to publish had been obtained.

Conclusions

The association of cathecolamines, argipressin and landiolol improves the outcome of patients with septic shock. The use of landiolol reduces the non-compensatory tachycardia. This association improves hemodynamic parameters, reduces lactate, allowing to decrease the dosage of cathecolamines.

References

1. Eddy Fan et al. An Official American Thoracic Society/European Society of Intensive Care Medicine/Society of Critical Care Medicine Clinical Practice Guideline: Mechanical Ventilation in Adult Patients with Acute Respiratory Distress Syndrome. Am J Respir Crit Care Med. 2017; Vol 195: 1253–1263.
2. Surviving Sepsis Campaign Guidelines 2021. Critical Care Medicine. October 2021.

## Infections and sepsis

### **A93 Empiric antibiotic therapy tailored to local bacterial flora in intensive care: our experience**

#### A. Usai, A. Busia, E. Lai, M. Muceli, G. Olla, A. Orru', S. Paba, A. Paddeu, S. Pilloni, M.V. Piroddi, S. Serdino, F.M. Loddo

##### Ospedale Nostra Signora Della Mercede - SC Anestesia E Rianimazione - ASL Ogliastra, Lanusei, Italy

###### **Correspondence:** A. Orru

*Journal of Anesthesia, Analgesia and Critical Care 2023,* **3(Suppl 1):**A93

Background: sepsis exists on a continuum of severity ranging from infection and bacteremia to sepsis and septic shock, which can lead to multiple organ dysfunction syndrome (MODS) and death.1 Ventilator-associated pneumonia (VAP) is one of the most frequent ICU-acquired infections. Reported incidences vary widely from 5 to 40% depending on the setting and diagnostic criteria. VAP is associated with prolonged duration of mechanical ventilation and ICU stay. The estimated attributable mortality of VAP is around 10%, with higher mortality rates in surgical ICU patients and in patients with mid-range severity scores at admission.2 Empiric therapy for patients with sepsis should be directed at the most common organisms causing sepsis, including various MDR pathogens. Thus, the administration of optimal doses of empiric broad spectrum intravenous therapy with one or more antimicrobials is recommended, according to guidelines; if a hospital acquired pneumonia is suspected, guidelines recommend administration of piperacillin/tazobactam, or cefepime, or ceftazidime, or meropenem.3

Materials and methods: we used a specific software for the management of laboratoristic microbiological data to analyze antibiotic sensitivity pattern of bacterial pathogens from various specimen sites such as bronchoalveolar lavage and bronchial aspirate, in a period from January 2018 to December 2022. Data were processed by creation of specific spreadsheets.

Results: lower respiratory infections pathogens were more sensitive to aminoglycosides (sensitivity of 92% and 72% for amikacin and gentamicin, respectively) and fluoroquinolones (sensitivity of 70% for ciprofloxacin) conversely, the antibiotics recommended by the guidelines had lower antibiotic susceptibility rates (sensitivity of 64%, 60% and 55% for cefepime, ceftazidime and piperacillin-tazobactam, respectively) except for carbapenems which maintained acceptable susceptibility rates (70% for meropenem).

Conclusions: The choice of antimicrobials can be complex and should consider the patient's history, comorbidities, immune defects, clinical context, suspected site of infection, presence of invasive devices, Gram stain test. Local prevalence and resistance patterns are relevant elements that can direct empiric therapy, thus antimicrobial choice in ICU should be tailored to local patterns of bacterial flora.1

References

1. Singer M, et al. The Third International Consensus Definitions for Sepsis and Septic Shock (Sepsis-3). JAMA 2016; 315:801.
2. Papazian L et al. Ventilator-associated pneumonia in adults: a narrative review. Intensive Care Med, 2020 May;46(5):888-906.
3. Donati A, Monti G. Percorso Diagnostico Terapeutico Assistenziale per il paziente con Sepsi e Shock Settico. Buone Pratiche Cliniche SIAARTI; v2 del 10/11/2020.

### A94 Hemoperfusion as anti-toxin strategy for Clostridium Perfringens-induced massive hemolysis

#### M. Domini ^1^, A. Ganss ^1^, I. Reffo ^1^, M. Cevolani ^1^, D. Rufolo ^1^, G. Del Fabro ^2^, S. Venturini ^2^, L. Pinciroli ^1^, M. Avolio ^2^, M. Crapis ^2^, M. Balbi ^1^, D. Tonin ^1^, G. Basaglia ^2^, G. Nadalin ^1^

##### ^1^ Azienda Sanitaria Friuli Occidentale, San Vito al Tagliamento, ITALY, ^2^ Azienda Sanitaria Friuli Occidentale, Pordenone, Italy

###### **Correspondence:** M. Domini

*Journal of Anesthesia, Analgesia and Critical Care 2023,* **3(Suppl 1):**A94

Background

Clostridium perfringens can rarely cause severe systemic infections associated with massive hemolysis, usually from an abdominal source and it is most of the time fatal. The consequences of hemolytic anemia and acute multifactorial kidney injury play a determinant role, making this condition a real emergency, requiring multispecialty skills and aggressive multimodal therapies.

Case report

A 55-year-old man was admitted to hospital because of hyperpyrexia and jaundice. Remarkable physical findings were diffuse abdominal tenderness and blackish urine. Blood tests, from grossly hemolyzed serum, showed leukocytosis, severe anemia with spherocytes and ghost cells, acute kidney and liver dysfunction (total bilirubin 7mg/dL, creatinine 3.26mg/dL), C-reactive protein 20mg/dL, procalcitonin >75ng/L; blood gas analysis revealed metabolic acidosis with hyperlactatemia. CTscan showed cholecystitis and multiple liver abscesses. Patient was intubated and placed on hemodynamic support because of rapid development of multiple organ dysfunction syndrome and hemoperfusion-coupled CVVH was started with modified AN69ST membrane oXiris®; multiple hemocomponents were transfused; piperacillin/tazobactam, clindamycin and fosfomycin were administered. Blood cultures became rapidly positive for Clostridium perfringens. Once hemolysis was controlled, cholecystectomy was performed, with rapid improvement of the patient’s condition but he subsequently developed a second septic episode from infected abdominal collections that required percutaneous drainage; at three months, he showed complete recovery.

Conclusion

Clostridium perfringens can rarely cause severe systemic infections with massive hemolytic anemia and kidney injury; its morbidity is due to the action of alpha toxin [1,2]. Continuous renal replacement therapy is of paramount importance to support renal function, manage fluid and electrolyte abnormalities, facilitate the clearance of waste red blood cell products and inflammatory cytokines [3]. Although high-volume ultrafiltration and hemoperfusion techniques have not demonstrated a clear survival benefit, the combination of these two techniques might be useful in this rare condition in which toxins, free hemoglobin and waste products contribute significantly to the pathophysiology of the disease [4,5]. Antibiotic therapy, also crucial, is usually empirical in this setting, and includes ureidopenicillin associated with clindamycin for its anti-toxin action and fosfomycin if polymicrobial origin is suspected [6].

Patient signed informed consent regarding publishing his data.

References

1. Van Bunderen CC, Bomers MK, Wesdorp E. Clostridium perfringens septicemia with massive intravascular hemolysis: a case report and review of the literature. Neth J Med. 2010; 68:343–6.
2. Suzaki A, Komine-Aizawa S, Nishiyama H. Massive intravascular hemolysis is an important factor in Clostridium perfringens-induced bacteremia. Intern Emerg Med. 2022 Oct;17(7):1959-1967.
3. Ankawi G, Neri M, Zhang J, Ronco C. Extracorporeal techniques for the treatment of critically ill patients with sepsis beyond conventional blood purification therapy: the promises and the pitfalls. Crit Care. 2018; 22:262.
4. Broman ME, Hansson F, Vincent JL. Endotoxin and cytokine reducing properties of the oXiris membrane in patients with septic shock: A randomized crossover double-blind study. PLoS One. 2019 Aug 1;14(8):e0220444.
5. Hellman T, Uusalo P, Järvisalo MJ. Renal Replacement Techniques in Septic Shock. Int J Mol Sci. 2021; 22(19), 10238.
6. Brook I. Treatment of anaerobic infection. Expert Rev Anti Infect Ther. 2007 Dec; 5(6):991-1006.

### A95 A sperimental procedure of cycling radical cleaning and disinfection to control nosocomial CRAB in ICU

#### V. Di Nardo, R. Commissari, P. Manzi, S. Cappanera, E. Sensi, B. Tiri, M. Scimmi

##### Azienda Ospedaliera S. Maria, Terni, Italy

###### **Correspondence:** V. Di Nardo

*Journal of Anesthesia, Analgesia and Critical Care 2023,* **3(Suppl 1):**A95

Background

Carbapenem-resistant Acinetobacter baumannii (CRAB) infections have increased over the last ten years in intensive care units (ICUs) [1]. CRAB is difficult to eradicate from the environment due to its ability to persist on surfaces and reduced susceptibility to biocides [2]. Multimodal infection prevention and control (IPC) strategies appear to be highly efective for CRAB prevention and control [3]. Nevertheless, controversy exists about which strategy is most pragmatic, especially in the context of limited economic and logistic resources and with regard to local epidemiology [4].

This work aims to define a set of actions implemented for the management of a cluster of patients with CRAB infection.

Materials and methods

The ICU has 15 beds distributed in two areas: section A composed by one room with three beds, two rooms with 2 beds and 3 in single, closed, isolation rooms; and section B with 5 beds in open space. The bed occupancy rate is of 95%. For the application of the procedure, a single patient room was identified which transitory unit. A new three-bed room (section C) near the ICU has also been identified.

Following the directions of Meschiari et al. [5] each colonized patient was moved from his original unit to the transitory unit. In this unit the patient’s skin was disinfected with 2% leave-on chlorhexidine disposable cloths and he was then transferred to section C for 6 hours. In these hours the patient’s unit was cleaning and disinfection of with 10% sodium hypochlorite for environmental surfaces and hydrogen peroxide in wipes for all medical devices. Air sanitization was also carry out. The common areas in the ICU were also disinfected. Day shifts have been reinforced with an additional nurse.

Results

The entire process took five days, in which the health management authorized the reduction of the number of beds from 15 to 14. An additional 3-bed room was required. The second phase of the study will concern the analysis and dissemination of findings related to infections and patient health.

Conclusions

The WHO-recommended approach to crab control is a multimodal approach with several interventions combined. To date there are very few studies that don’t include the temporary closure or cohort of patients colonized for limit CRAB spread in ICU. The results obtained from the application of this procedure will provide useful elements for the management of crab clusters, in order to avoid the closure of ICU and limit hospitalisations.

References

1. Cassini et al. Attributable deaths and disability-adjusted life-years caused by infections with antibiotic-resistant bacteria (…). Lancet Infect Dis. 2019;19:56–66.
2. Lerner et al. Environmental contami nation by carbapenem-resistant Acinetobacter baumannii (…). Infect Control Hosp Epidemiol. 2020;41:166–71.
3. Wieland et al. Nosocomial outbreaks caused by Acinetobacter baumannii and Pseudomonas aeruginosa in heathcare facilities (…). Am J Infect Control. 2018;46:643–8.
4. Tomczyk et al. Control of carbapenem-resistant enterobacteriaceae, acinetobacter baumannii, and pseudomonas aeruginosa (...). Clin Infect Dis. 2019;68:873–84
5. Meschiari et al. A five-component infection control bundle to permanently eliminate a carbapenem-resistant Acinetobacter baumannii spreading in an itensice care unit. Antimicrob Resist Infect Control. 2021Aug19;10(1):123.

### A96 Pneumonia and meningoencephalitis resulting from reactivation of varicella-zoster virus in an immunocompetent adult: a case report

#### C. De Domenico ^1^, G. Dell'Aglio ^1^, F. Pascucci ^2^

##### ^1^ Università degli Studi di Brescia, Brescia, ITALY, ^2^ ASST Spedali Civili, Brescia, Italy

###### **Correspondence:** C. De Domenico

*Journal of Anesthesia, Analgesia and Critical Care 2023,* **3(Suppl 1):**A96

Background

Varicella-zoster virus (VZV) is one of eight herpesviruses known to cause human infection. Primary VZV infection causes varicella or chickenpox in children. Then, it becomes latent in cranial nerve and dorsal-root ganglia and may reactivate many years later as herpes zoster or shingles, especially in advanced age or in immunocompromised patients.[1] The reactivation of the VZV causes dermatomal herpes zoster (HZ) and more rarely severe disseminated HZ including diffuse rash, encephalitis, hepatitis and pneumonia.[2]

Case Report

A 66-year-old man developed cervicalgia after exertion, which was treated with nonsteroidal anti-inflammatory drugs plus skeletal muscle relaxants, without any improvement of his conditions. A week later, he started suffering from increasing pain, nausea, vomit, vertigo and acute onset hypertension. He was admitted to the Emergency Department with complaints of fever, confusion and fluctuant level of consciousness. Physical examinations revealed nuchal rigidity and a vesicular rash on the face and around the belly scattered in a nondermatomal pattern.

The patient did not know if he had previously had varicella. His past medical history was significant for chronic HBV infection and early stage hepatocellular carcinoma successfully treated with percutaneous ethanol injection.

CT brain and laboratory work-up were unremarkable (except for mild neutrophilic leukocytosis, WBC 11’530/uL). However a FilmArray panel on cerebrospinal fluid detected the varicella zoster virus, treated with intravenous acyclovir. A positive IgG result (3270 mIU/ml, positive when > 165) coupled with a negative IgM result indicated VZV reactivation.

Two days later, he complained of progressively worsening dyspnea leading to acute hypoxic respiratory failure, which required intubation and admission to the ICU. Chest CT showed many small, irregular hyperdense abnormalities and ground-glass opacities with crazy-paving pattern in the right lung (figure 1). CT pulmonary angiography showed small bilateral filling defects in segmental pulmonary arteries managed with Intravenous heparin in continuous infusion.

Empirical antibiotics were initiated using piperacillin-tazobactam due to the potential for aspiration pneumonia.

A lung protective ventilation was applied, which required neuromuscular blocking agents for a week. However, the patient deteriorated with worsening lung compliance and hypoxemia: PaO2 / FiO2 (P/F) ratio remained <100 for about two weeks and the patient displayed recurrent acute desaturations in 100% oxygens. Prone position ventilation did not improve the oxygenation.

As the patient had persistent fever, empirical antibiotics were changed to cefotaxime and a methylprednisolone therapy was started. A tracheostomy was performed because of difficult weaning.

On post-admission day 20, gas exchanged began to improve and on day 35 he was successfully weaned from the ventilator. Eventually, the patient had a complete recovery from neurological and respiratory symptoms.

Conclusion

From this case review, we speculated that varicella pneumonia management is challenging also in healthy patients; however early aggressive management may improve the prognosis.

Informed consent to publish had been obtained.

References

1. Freer G, Pistello M. Varicella-zoster virus infection: natural history, clinical manifestations, immunity and current and future vaccination strategies. New Microbiol. 2018 Apr;41(2):95-105.
2. Lewis DJ, Schlichte MJ, Dao H Jr. Atypical Disseminated Herpes Zoster: Management Guidelines in Immunocompromised Patients. Cutis. 2017;100:321;324;330

**Fig. 1 (abstract A96).**
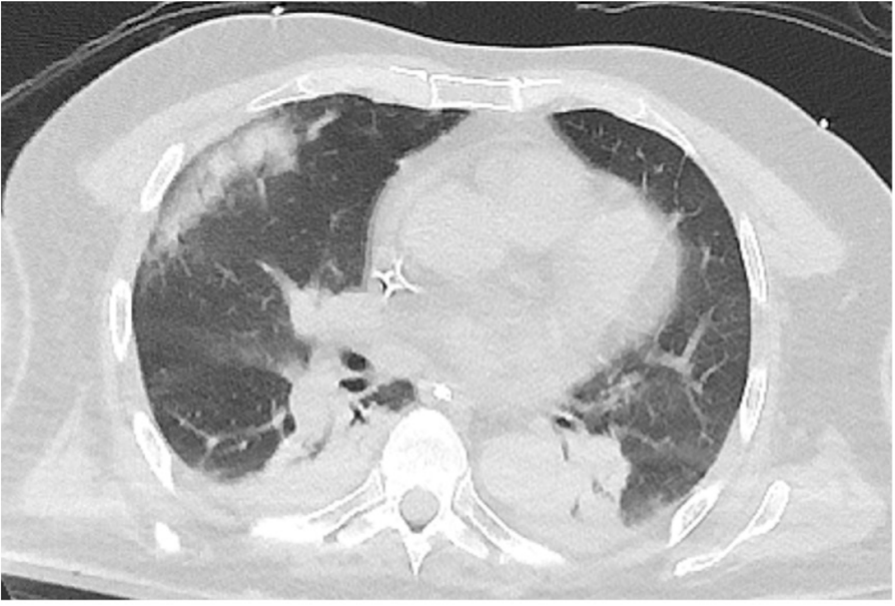
See text for description.

### A97 Decatecholaminisation with landiolol in septic shock: a case report

#### I. Cappellini, L. Zamidei, L. Campiglia, G. Consales

##### Azienda USL Toscana Centro- Ospedale Santo Stefano, Prato, Italy

###### **Correspondence:** I. Cappellini

*Journal of Anesthesia, Analgesia and Critical Care 2023,* **3(Suppl 1):**A97

Introduction

Sepsis is a life-threatening condition characterized by a dysregulated host response to infection, leading to multiple organ dysfunction (1). Sepsis management requires a multidisciplinary approach that includes early recognition, source control, and appropriate antibiotic therapy. In addition, management of hemodynamic instability, including hypotension and tachycardia, is critical in septic shock patients. Decatecholaminisation, a strategy to reduce adrenergic stress, has been proposed to improve outcomes in septic shock patients (2).

Case

We report a case of a 65-year-old male who presented to the emergency department with fever, hypotension, and altered mental status. The patient was diagnosed with septic shock due to a urinary tract infection and was started on broad-spectrum antibiotics and aggressive fluid resuscitation. Despite initial resuscitation, the patient remained hypotensive and tachycardic, requiring high doses of norepinephrine for hemodynamic support.

Given the concern for the potential adverse effects of prolonged adrenergic stress, the decision was made to initiate landiolol, an ultra-short-acting beta-blocker, to control heart rate and reduce adrenergic stress. Landiolol was started at a low dose and titrated up to achieve a target heart rate of 60-80 beats per minute. The patient's hemodynamic status improved, and the dose of norepinephrine was gradually decreased. The patient remained on landiolol for 72 hours with close monitoring of blood pressure and heart rate.

This case illustrates the potential benefit of decatecholaminisation in septic shock patients, particularly in those with persistent tachycardia despite aggressive fluid resuscitation and high-dose vasopressor therapy. Landiolol, with its high selectivity for beta1-adrenergic receptors and ultra-short duration of action, can effectively reduce heart rate without compromising blood pressure and inotropy. Further studies are needed to evaluate the safety and efficacy of landiolol in septic shock patients and to determine optimal dosing strategies.

Conclusion

In conclusion, decatecholaminisation, including the use of landiolol, may be a valuable strategy in the management of septic shock patients. Early recognition of hemodynamic instability and aggressive fluid resuscitation remain the cornerstone of sepsis management, and the addition of decatecholaminisation should be considered in patients with persistent tachycardia despite adequate fluid resuscitation and vasopressor therapy. Further studies are needed to fully evaluate the safety and efficacy of decatecholaminisation in septic shock patients.

Informed consent was obtained from the patient to publish this case report

References

1. Evans L, Rhodes A, Alhazzani W, Antonelli M, Coopersmith CM, French C, et al. Surviving sepsis campaign: international guidelines for management of sepsis and septic shock 2021. Intensive Care Med. 2021;47(11):1181-247.
2. Rudiger A, Singer M. Decatecholaminisation during sepsis. Critical Care. 2016;20(1).

### A98 Polydistrectual resistance index evaluation is an assessment of vascular compliance in patients with septic shock treated with vasopressin

#### A. Barile ^1^, A. Recchia ^2^, G. Paternoster ^3^, M. Copetti ^4^, A. Manuali ^2^, L. Mirabella ^1^, G. Cinnella ^1^, A. Del Gaudio ^2^

##### ^1^ Department of Anesthesia and Intensive Care,University of Foggia,Foggia,Italy, Foggia, Italy; ^2^ Anesthesia and Intensive Care 2,IRCCS Casa Sollievo della Sofferenza , San Giovanni Rotondo,Italy, San Giovanni Rotondo, Italy; ^3^ Cardiovascular Anaesthesia and ICU,Potenza,Italy, Potenza, Italy; ^4^ Unit of Biostatistics,IRCCS Casa Sollievo della Sofferenza ,San Giovanni Rotondo,Italy, San Giovanni Rotondo, Italy

###### **Correspondence:** A. Barile

*Journal of Anesthesia, Analgesia and Critical Care 2023,* **3(Suppl 1):**A98

Background

Surviving Sepsis Campaign recomends using Norepinephrine (NE) as the first-line vasopressor to restore mean arterial pressure. If mean arterial pressure remains inadeguate SSC suggests adding Vasopressin (VA).

Resistance Index (RI) is a Power Doppler ultrasound assessment of vascular compliance to detect organ perfusion.

Materials and methods

Aim of this study is to compare RI in septic shock patients treated with NE (Group1),NE plus VA since the beginning of vasopressor therapy (Group2) and VA plus NE where VA is added if NE dosage was 20 mcg/min (Group3).

RI were measured in Renal Artery (ARE), Radial Artery (AR),Central Retinal Artery (CRA),Superior Mesenteric Artery (AMS) at three different time points (T0) before vasopressor therapy,(T1) at 1 hr,T2 at 24 hrs and T3 at 48hrs.

Results

48 patients were divided into three groups.17 patients Group1;16 Group 2 ,15 Group3.

In Group 1 RI increased from T0 in CRA R[0,90(0,57–1,12)] and ARE L[0,74(0,56-0,92) to T3 in CRA R[0,97(0,97–1,14)] and ARE L[0,96(0,82–1,17)]

In Group2 RI reduced in AMS, from T0[0,84(0.70,1.02)] to T3[0,75(0.59,0.81)],in CRA R, from T0[0,90(0.57,1.09] to T3[0,79(0.58, 0.87)],in CRA L,from T0[0,91(0.43,1.53)] to T3[0,76(0.58, 0.89] and in ARE L,from T0[0,79(0.58, 0.92)] to T3[0,72(0.59, 0.83)].

In Group3 RI reduced in AMS, from t0[0,86(0.71,0.93)] to T3 [0,68(0.64,0.81)],in CRA R,from T0 [0,90(0.75,1.12)] to T3 [0,78(0.66,0.88) ],in CRA L,from T0[0.96(0.76,1.33)] to T3 [0,96(0.76,1.33)],in ARE L,from T0[0.77(0.66, 0.99)]to T3[0,67(0.61,0.85],in ARE R,from T0[0.82[0.64, 0.90]]to T3[0.70(0.62,0.82)] and in AR R,from T0[1,10(0.81,1.30)] to T3 [0.87(0.64,1.22)].(FIGURE 1)

Conclusions

Resistence Index was singnificantly reduced in patients treated with early synergic administration of NE and VA .This strategy optimized multiorgan perfusion.

Reference

1. Evans L. et Al. Surviving Sepsis Campaign: International Guidelines for Management of Sepsis and Septic Shock 2021 Critical Care Medicine November 2021 49(11):p e1063-e1143.

B) Sacha L. et Al. Association of Catecholamine Dose, Lactate, and Shock Duration at Vasopressin Initiation With Mortality in Patients With Septic Shock. Sacha Crital Care Medicine April 2022 1;50(4):614-623.

**Fig. 1 (abstract A98).**
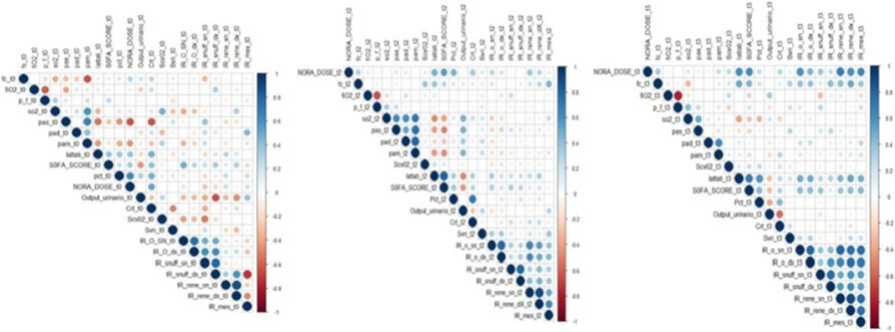
Correlations between all the variables analyzed at T0, T2 and T3 (a,b,c). Intensity of Red indicates Inverse Correlations,Blue indicates Direct. There is a greater direct correlation at T3 between the dose of Norepinephrine and the Resistance Index. The hypothesis that can be made to explain this is linked to the mechanism of Receptor Desensitization. Receptor Desensitization means that the dosage of Norepinephrine must be increased over time to produce the desired effect (MAP>65mmHg). This increase therefore determines, in addition to the desired effect, also an increase in side effects such as tachyphylaxis, vasoconstriction and therefore the increase of the Resistance Indices. -IR MES SUP= AMS;IR O right/left= CRA R/L; KIDNEY IR right/left= ARE R/L; IR snuff right/left=AR R/L ;NORA DOSE= Ne dose

### A99 Decatecholaminizahtion in septic shock and argipressin: an experience

#### Erica Plasmati, Angela Grassi, Monica Armento, Lucia Gaudio, Francesco Zuccaro, Francesco Massim Romito

##### Terapia Intensiva Generale Ospedale Matera Asm Basilicata, Matera, Italy

###### **Correspondence:** Erica Plasmati

*Journal of Anesthesia, Analgesia and Critical Care 2023,* **3(Suppl 1):**A99

BACKGROUND: Septic shock represents a serious health care problem associated with significant morbidity and mortality.

Current guidelines recommend argipressin in addition to catecholamines in refractory septic shock with the aim to rising the MAP to target or to decrease the norepinephrine dosage.

We aimed to analyze the role of argipressin as second line vasopressor in patients with septic shock who already receive norepinephrine evaluating timing of the addition and outcomes in terms of decatecholaminization, improvement of splancnic circulation and reduction in serum lactate levels.

MATERIALS and METHODS: We conducted an observational study in ICU of hospital “Madonna delle Grazie” - Matera. From June to November 2022 data of 9 patients were collected.

The drafted protocol calls for the administration of argipressin in septic shock patients receiving norepinephrine at a rate grater than or equal to 0.2 gamma/kg/min.

A starting infusion rate of 0.25 ml/h was considered, then proceed with subtending doses of 0.25 ml/h every 15-20 minutes up to a maximum of 2.25 ml/h based on the patient’s response.

Once PAM target was reached, the dosage of norepinephrine was first reduced and then the dosage af argipressin was reduced.

An advanced hemodynamic monitoring system (MOSTCARE™) was used.

Attention was focused on the value of PAM, lactates, Systemic Vascular Resistance index (SVRI), 24 h diuresis.

For each individual patient data were collected at TIME 0 (admission in ICU), TIME 1 (start of argipressin) and TIME 2 (discontinuation of argipressin).

Main values over the predefined time intervals was calculated for each parameter.

RESULTS: Our analisys revealed increase in the PAM value, progressive reduction of lactate levels, and augmented 24h diuresis upon discontinuation of argipressin (as shown in Figure).

Systemic vascular resistance index are almost superimposable in the first two time intervals and then decrease significantly at the end of argipressin treatment.

CONCLUSIONS: Further studies are needed but our experience can suggest the addition of vasopressin when norepinephrine administration at a rate greater than or equal to 0.2 gamma/kg/min as a catecholamine sparing strategy.

REFERENCES

1. Julien Demiselle et al Vasopressin and its analogues in shock states : a review Annals of Intensive Care 2020
2. Rui Shi et al Vasopressors in septic shock : which, when and how much? Annals of Translational Medicine 2020

**Fig. 1 (abstract A99).**
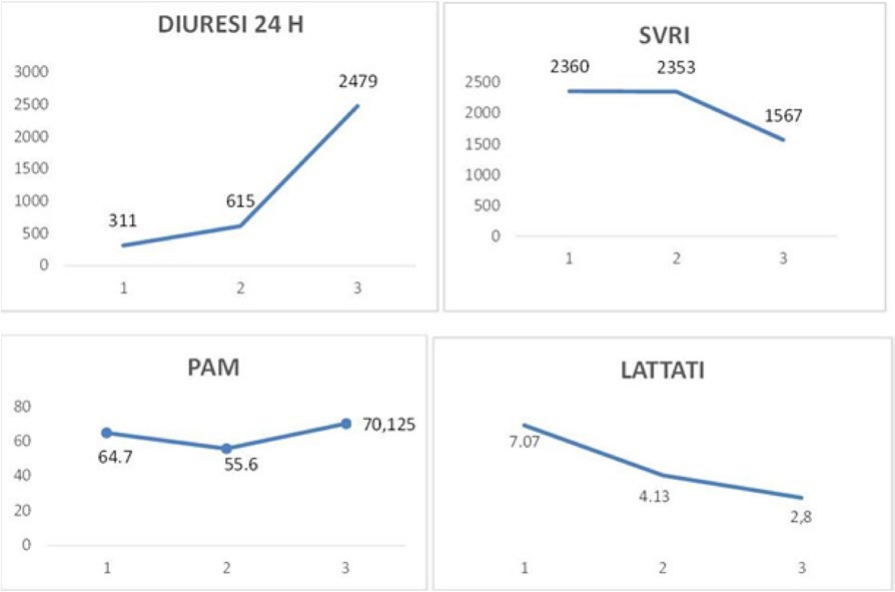
Diuresis, SVRI, PAM, LACTATE variation

## Critical medicine and out-of-hospital emergency

### **A100 Normothermic and hypothermic patient with Out of Hospital Sudden Cardiac Arrest, from the street to the ECLS hospital: a brief report**

#### R. Varutti ^1^, A. Spasiano ^3^, F. Bassi ^1,3^, G. Trillò ^2^

##### ^1^ SOC Anestesia Rianimazione 2, Azienda Ospedaliero Universitaria Santa Maria della Misericordia, Udine, ITALY;^2^ UOC Centrale Operativa SUEM 118 Belluno, Belluno, Italy;^3^ SOSD Elisoccorso Regionale, Azienda Ospedaliero Universitaria Santa Maria della Misericordia, Udine, Italy

###### **Correspondence:** R. Varutti

*Journal of Anesthesia, Analgesia and Critical Care 2023,* **3(Suppl 1):**A100

BACKGROUND:

Asystole and refractory ventricular fibrillation are usually treated by Advanced Life Support (ALS). The use of a mechanical chest compressor is usually applied until ECMO start. No flow and low flow times are considered after sudden cardiac collapse, and they are registered and summarized at transport time. Every ECLS hospital adopt a local protocol to start ECMO as current guidelines recommendation1, but observed outcome are very different.

We present few cases collected from the 118/112 setting in two nearest Italian areas.

MATERIALS AND METHODS:

We collected data from February 2017 until April 2023 retrospectively, from HEMS FVG and HEMS Belluno service. We enrolled 6 normothermic (>35°C) or hypothermic (< 35°C) patient with sudden cardiac arrest out of hospital setting. From each case, we recorded time of no flow, low flow, transport, on site treatment time, mg of administered adrenaline, number of delivered shocks, mechanical chest compressor utilization and outcome (death, alive, organ donor). Clinical consent were obtained.

RESULTS:

The results are shown on table 1. Six patients were enrolled (5 Male), mean age: 48 years (range 34-60 y). Three patients were hypothermic due to avalanche burial. No-flow time were higher in hypothermic patients (due to unearthing times ranging from 20 to 45 min) vs <3 min in normothermic patients, whereas time spent on place by ALS rescue team were lower on hypothermic patient. Hypothermic patient received 1 mg of adrenaline maximum (vs 7 mg mean adrenaline in normothermic patient), no DC shock were administered, and airways have been quickly secured for rapid evacuation (EtCO2> 20 mmHg). The patient who received lower dose of adrenaline and DC shock have had better pH, lactates and potassium at the hospital arrival and needed lower dosage of iv amines for organ support. In 4 cases, the patients were transported to the nearest ECLS hospital by Helicopter.

CONCLUSIONS:

Despite international recommendation regarding ECLS therapy in sudden cardiac arrest and local protocols, the main difference in terms of surviving and outcome is body temperature and the rapid management and transport to the nearest ECLS hospital 2,3. Probably, the lowest dose of adrenaline, mechanical chest compressor and early airway management (sparing time) allow the greater hemodynamic patient stability and the best outcome.

REFERENCES:

1. G.D. Perkins et al. European Resuscitation Council Guidelines 2021. Resuscitation 2021 https://doi.org/10.1016/j.resuscitation.2021.02.003
2. Yan S, Gan Y, Jiang N, et al. The global survival rate among adult out-of-hospital cardiac arrest patients who received cardiopulmonary resuscitation: A systematic review and meta-analysis. Crit Care. 2020;24:61. doi: 10.1186/s13054-020-2773-2
3. De Charrière A, Assouline B, Scheen M, et al. ECMO in Cardiac Arrest: A Narrative Review of the Literature. J Clin Med. 2021; 10(3): 534

**Table 1 (abstract A100).** Data collected from Out of Hospital Cardiac Arrest (OHCA)

	Hypothermic patient (n. 3)	Normothermic patient (n. 3)
No flow time (minutes)	20-60	< 3
Low flow time (minutes)	< 5	Mean 30 (18-32)
First rhythm	Asystole (3)	Ventricular fibrillation (3)
DJ shock	0	Range 2-11
Mechanical chest compressor	2	3
Intermittent manual chest compression	1	0
Time to OHCA and ECMO start (minutes)	180-215	60-120
Alive	0	0
Donor	1 (liver, kidneys)	0
Time to death (h after ECMO start)	3 to 36 h	< 2 h

### A101 Emotions and skill maneuvers cardiopulmonary resuscitation of college students.

#### G. Sciamanna ^1^, N. Tiberii ^1^, M.L. Simonetti ^2^

##### ^1^ Università Politecnica delle Marche, Ascoli Piceno, Italy; ^2^ Università politecnica delle Marche, Ascoli Piceno, Italy

###### **Correspondence:** G. Sciamanna

*Journal of Anesthesia, Analgesia and Critical Care 2023,* **3(Suppl 1):**A101

Introduction: Knowledge of BLS-D maneuvers (Basic Life Support and Defibrillation) is essential for immediate intervention and early defibrillation in the event of cardiac arrest. It is important that the majority of the population know about them. European and international scientific literature has shown that BLS-D performance in students can be improved. The purpose of this study was to investigate the BLS-D skills and emotions related to such emergency interventions of students enrolled at the Polytechnic University of Marche.

Materials and methods: Descriptive observational study carried out by administering a validated anonymous questionnaire, subject to informed consent, to students both in the health sector (e.g. Medicine, Nursing, Physiotherapy, Obstetrics) and in the non-health sector (e.g. Economics, Engineering, Agriculture), with 15 questions regarding CPR following ILCOR 2015 recommendations and ERC guidelines. Data collection was carried out from January to April 2023. The data was processed with SPSS software. The sample analysed is 201 students.

Results: 71.64% are women, 28.36% are men. For both sexes, the older age group is 19-24 years. A cut-off of at least 10 correct answers out of 15 was adopted for an evaluation considered positive. 61.69% of the students obtained a sufficient score on the quiz. Sufficiency was significantly associated with the following variables: attendance of a BLS-D course (p < 0.001), attending a health degree course (p < 0.001), experiencing emotions of security, calm, lucidity if one had to intervene ( p < 0.005) and knowledge of smartphone apps that indicate the presence of defibrillators (p < 0.005). Students who have a course attendance certificate had a good score on the questionnaire compared to those who did not (56.98% vs. 43.02%). 38.31% of the sample resulted in poor preparation with, on the contrary, associated feelings of stress and confusion (p < 0.003). fear of causing harm to the person. (n..92), (p < 0.0012) fear of legal repercussions (n. 98) (p < 0.0023).

On the other hand, the fear of contracting infections from the person in AC was not correlated (n. 27) (p = 0.08) despite the fact that we are in the post-Covid phase.

Conclusions: It is important that in all faculties, both health and non-health, certified simulation laboratories are introduced through virtual reality such as BLSD-VRQ courses which would be optimal and attractive for young people.

### A102 Emergency Team Competencies: scoping review for the development of a tool to support the briefing and debriefing activities of emergency healthcare providers

#### G. Lorenzini ^1^, A. Zamboni ^2^, L. Gelati ^1^, A. Di Martino ^1^, A. Pellacani ^1^, N. Barbieri ^1^, M. Baraldi ^1^

##### ^1^ Azienda USL Modena, Modena, Italy;^2^ SIMANNU Centro di Simulazione

###### **Correspondence:** G. Lorenzini

*Journal of Anesthesia, Analgesia and Critical Care 2023,* **3(Suppl 1):**A102

International figures for the year 2021 report that 10 per cent of patients using healthcare services experience at least one adverse event. Globally, this translates into 3 million deaths due to unsafe care and an expenditure of USD 606 billion per year, just over 1% of the total economic output of OECD countries. Human factors play a crucial role in the development of adverse events. In fact, among the results of surveys included in OECD countries, only 46% of healthcare professionals believe that key information about patient care is transferred effectively between operating units (OUs) and during shift changes. On average, 68% of staff report high levels of teamwork within their O.U., that their organisation shows an improvement in the quality of care following adverse events (65%) and that the changes implemented have been evaluated for effectiveness. It is thus clear that patient safety is one of the most significant hidden issues for public and private healthcare worldwide and that, in order to address this problem, governance based on long-term investments focused on building mechanisms to ensure adequate staffing levels, training and on-the-job support is required. The innovation of curricular pathways linked to the development of human factors, in terms of quality and frequency, represents a focal point that the literature explores, deepens and finally considers as the key to raising the profile of the care provided and the safety of the patients assisted. From this issue stems the aim of this research, namely to introduce an operational tool, built through critical analysis of the literature, that supports the briefing and debriefing activities of teams facing an emergency. The briefing and debriefing tool 'Emergency Team Competencies' is proposed as a universal tool to support briefing and debriefing activities and for all intra- and extra-hospital emergencies.

Briefing and debriefing activities during an emergency support professionals to organise work, resources, plan interventions, recognise mistakes made, strengths and improve future human factors performance in team work. The aim of the research, therefore, is to introduce an operational tool, consisting of a framework of technical and non-technical skills needed to conduct critical patient care, that supports the briefing and debriefing activities of teams in any setting and according to a guideline that includes gold standard team and clinical skills.

The proposed briefing and debriefing tool identifies a framework of key competences to be detected, evaluated and discussed with the team, consisting of 8 Thematic Areas, 32 Building Blocks and 44 Behaviours (Table 1). The lack of literature exploring all critical aspects to be detected, assessed and discussed before and after an emergency is the rationale behind the drafting of the tool. In the near future, the evidence that will emerge from the new scientific literature and the further studies that will be conducted to validate the briefing and debriefing tool, to test the validity of the tool in the objective measurement of practitioners' performance and to measure the change in performance following the implementation of daily briefing and debriefing practice will be important.

**Fig. 1 (abstract A102).**
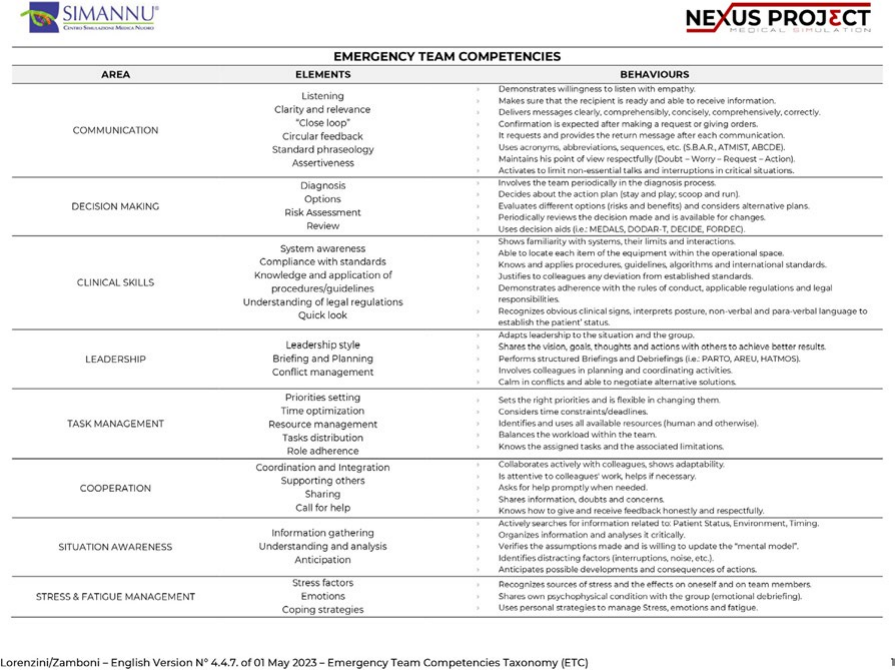
See text for description.

### A103 Out-of-Hospital Cardiac Arrest Occurrence During Five Waves of the Covid-19 Pandemic: Impact on the Activity of the Emergency Medical Service of Treviso

#### A. Graziano ^1^, P. Franceschin ^2^, M. Zagagnoni ^1^, S. Orazio ^1^, M. Carron ^2^, M. Ferramosca ^1^

##### ^1^ Treviso Emergency Medical Service (EMS - 118), Treviso Regional Hospital AULSS 2 Marca Trevigiana, TREVISO, Italy; ^2^ Institute of Anesthesia and Intensive Care, Department of Medicine, University of Padua, PADOVA, Italy

###### **Correspondence:** A. Graziano

*Journal of Anesthesia, Analgesia and Critical Care 2023,* **3(Suppl 1):**A103

Introduction: Out-of-hospital cardiac arrest (OHCA), defined as the cessation of mechanical cardiac activity, remains one of the most common causes of death in developed countries. The management of OHCA requires a swift, intensive support to avoid the irreversible damage caused by hypoxia.

The SARS-CoV-2 (Covid-19) pandemic has had an impact on medical care across the world, including on out-of-hospital emergency services.

Goal of the study: The primary goal of this study is to evaluate the impact of the Covid-19 pandemic on the pre-hospital survival in OHCA cases in which the Treviso emergency medical service (EMS-118) intervened.

Materials and Method: We used an observational retrospective study confronting OHCA cases observed in six different periods, each 3 months long: a pre-pandemic period control (February-April 2019) and five periods relating to five pandemic waves (February-April 2020; November 2020-January 2021; February-April 2021; August-October 2021; November 2021-January 2022).

The data were extracted from the Treviso EMS-118 database.

Results: Overall, 1,188 cases of OHCA were reported. The summary of all the cases is presented in Table 1. We observed that fewer OHCA cases occurred in public places during the pandemic periods compared to the pre-pandemic period as well as fewer cases of ventricular fibrillation. However, we noted an increased number of cases in which asystole was the rhythm of presentation. Moreover, we observed a significant drop in basic life support (BLS) maneuvers practiced by bystanders in the first pandemic wave (16.9% versus 32.3%, p<0.05) and fewer cases where the advance cardiac life support (ACLS) was employed by EMS-118 personnel during the five waves of the Covid-19 pandemic (Table 1).

Compared with the control period, in the five pandemic periods, a stable reduction of pre-hospital survival rates was observed, with a significant decrease of return of spontaneous circulation (ROSC) during the second and third pandemic waves (Table 1).

The multivariate logistic regression analysis shows that the public event (OR [95%CI]: 2.90 [1.46-5.76; p=0.002), ventricular fibrillation as rhythm of presentation (OR [95%CI]: 2.42 [1.03-5.65; p=0.042), BLS (OR [95%CI]: 1.86 [1.09-3.15; p=0.022) and ACLS (OR [95%CI]: 6.02 [2.74-13.20; p<0.001) have a positive impact on OHCA survival observed during the five pandemic waves.

Conclusion: The Covid-19 pandemic significantly reduced OHCA survival during the periods studied.

The adoption of national and international measures to prevent and to contain the spread of Covid-19 contributed to a decrease in the OHCA cases in public, a decrease in the number of patients who benefited from BLS by bystanders, and an improvement in unfavorable clinical settings for the adoption of ACLS.

The negative impact on survival, despite a progressive reduction in containment measures, also suggests a direct role of Covid-19 infection in the OHCA outcome observed during the five pandemic waves.

The BLS and ACLS benefits for pre-hospital survival of OHCA are confirmed.

References

- Gräsner JT, Wnent J, Herlitz J, et al. Survival after out-of-hospital cardiac arrest in Europe - Results of the EuReCa TWO study. Resuscitation. 2020;148:218-26
- McNally B, Robb R, Mehta M, et al. Out-of-hospital cardiac arrest surveillance --- Cardiac Arrest Registry to Enhance Survival (CARES), United States, October 1, 2005--December 31, 2010. Morb Mortal Wkly Rep Surveill Summ Wash DC 2002. 29 luglio 2011;60:1-19
- Merchant RM, Topjian AA, Panchal AR, Cheng A, Aziz K, Berg KM, et al. Part 1: Executive Summary: 2020 American Heart Association Guidelines for Cardiopulmonary Resuscitation and Emergency Cardiovascular Care. Circulation 2020;142:S337-5

**Table 1 (abstract A103).** OHCA cases characteristics, observed during the during the five pandemic waves, compared with the pre-pandemic period (control)

Variable	Control ***F-A 2019***	1^**st**^ wave ***F-A 2020***	2^**nd**^ wave ***N-G 2020/21***	3^**rd**^ wave ***F-A 2021***	4^**th**^ wave ***A-O 2021***	5^**th**^ wave ***N-G 2021/22***	CA ***P-value***
**Total events, n 1,188**	198	195	247	200	155	193	
**Age, years [IQR]**	80 [69-89]	81.5 [71-89]	80 [71-87]	80 [69-87]	82 [69-87.2]	79 [68-87]	0.835
**Gender, n (%)**							0.377
• Male	115 (59.6)	120 (61.5)	157 (63.6)	114 (57)	96 (61.9)	108 (56)	
• Female	78 (40.4)	75 (38.5)	90 (36.4)	86 (43)	59 (38.1)	85 (44)	
**Place, n (%)**							0.124
• Private	172 (86.9)	184 (94.4)	236 (95.5)	188 (94)	139 (89.7)	180 (93.3)	
• Public	**22 (11.1)**	9 (4.6)*	7 (2.8)*	**10 (5.0)**	**12 (7.7)**	9 (4.7)*	
• Not reported	4 (2.0)	2 (1.0)	4 (1.6)	2 (1.0)	4 (2.6)	4 (2.1)	
**Onset, n (%)**							**0.0094**
• VF	**44 (22.2)**	21 (10.8)*	35 (14.2)*	28 (14)*	18 (11.6)*	13 (6.7)*	
• Asystole	**124 (62.6)**	145 (74.4) *	175 (70.9)*	145 (72.5)*	120 (77.4)*	152 (78.8)*	
• PEA	18 (9.1)	15 (7.7)	19 (7.7)	13 (6.5)	6 (3.9)	12 (6.2)	
• Not reported	12 (6.1)	14 (7.2)	18 (7.3)	14 (6.5)	11 (7.1)	16 (8.3)	
**BLSD, n (%)**							0.235
• No	**126 (63.6)**	150 (76.9)*	169 (68.4)	126 (63)	96 (61.9)	133 (68.9)	
• Yes	**64 (32.3)**	33 (16.9)*	65 (26.3)	64 (32)	52 (33.5)	56 (29)	
• Not reported	8 (4)	12 (6.2)	13 (5.3)	10 (5)	7 (4.5)	4 (2.1)	
**ACLS, n (%)**							**0.037**
• No	103 (52)	114 (58.5)	149 (60.3)	115 (57.5)	91 (58.7)	128 (66.3)	
• Yes	87 (43.9)	68 (34.9)	84 (34)	75 (37.5)	57 (36.8))	61 (31.6)	
• Not reported	8 (4)	13 (6.7)	14 (5.7)	10 (5)	7 (4.5)	4 (2.1)	
***Pre-hospital Outcome***							
1-Survilal, n (%)	**40 (20.2)**	22 (11.3)*	22 (8.9)*	18 (9)*	20 (12.9)*	22 (11.4)*	**0.041**
• ROSC	**25 (12.6)**	13 (6.7)	10 (4)*	11 (5.5)*	10 (6.5)	13 (6.7)	0.057
• ACLS till admission	15 (7.6)	9 (4.6)	12 (4.9)	7 (3.5)	10 (6.5)	9 (4.7)	0.392
2-Death, n (%)	158 (79.8)	173 (88.7)	225 (91.1)	182 (91)	135 (87.1)	171 (88.6)	0.270

Data expressed in absolute numbers (percentage, %) or mean value [interquartile range, IQR]. Continuous and absolute values from the pandemic waves with Mann-Whitney U test and χ^2^ test respectively. Variability analyses with ANOVA test and analyses of the proportion trend with the Cochran-Armitage (CA) test. Significance p<0.05. (*) Significance pandemic waves vs. pre-pandemic control period. *BLSD* basic life support defibrillation, *ACLS* advance cardiac life support, *ROSC* return of spontaneous circulation

### A104 Evaluation of the appropriateness of major trauma criteria in centralizing helicopter emergency medical services in the Marche region

#### A. Vito ^1^, A. Salvucci Salice ^2^, R. Antolini ^2^, E. Vitali ^2^, F. Santoni ^2^, G. Perini ^2^, C. Pacini ^2^, A. Donati ^1-2^, A. Carsetti ^1, 2^

##### ^1^ Azienda Ospedaliero Universitaria delle Marche, Ancona, Italy; ^2^ Dipartimento di Scienze Biomediche e Sanità Pubblica, Università Politecnica delle Marche, Ancona, Italy

###### **Correspondence:** A. Vito

*Journal of Anesthesia, Analgesia and Critical Care 2023,* **3(Suppl 1):**A104

Introduction

The major factors impacting mortality and disability in case of Major Trauma are time and adequacy of pre-hospital, intra-hospital, and rehabilitation interventions, for this reason, Major Trauma System (SIAT) was established.

In the Marche region, based on the Regional Diagnostic-Therapeutic Assistance Pathway (PDTA) for the management of severe trauma, helicopter emergency medical services (HEMS) always involve centralization at the Level II Emergency Department of the Marche University Hospital. Major Traumas are classified on clinical criteria (3R code) and situational criteria (2R code).

This system aims to identify all patients with possible serious injuries, even those not immediately apparent, although a certain level of over-triage. According to the guidelines of ACS-COT, the acceptable over-triage rate should range from 25% to 35%.

The purpose of this study is to describe the rate of over-triage among patients centralized through situational criteria using HEMS at the Ancona Trauma Center.

Methods

This retrospective observational single-center study analyzes data from adult patients centralized through HEMS from January 2022 to March 2022. In assessing over-triage, Injury Severity Score (ISS) was calculated, with patients having an ISS >15 considered as major trauma cases. The level of care required and the outcome at discharge, according to the Glasgow Outcome Scale (GOS), were also considered.

Results

63 patients were included in the study, 17 of them were classified as 3R and 46 of them were classified as 2R. Among the 3R patients, 29.14% (5) had an ISS <15. Among the 2R patients, 84.78% (39) had an ISS <15. Table 1 describes the over-triage for each situational criterion. The overall rate of over-triage was 69.84% (44/63).

3R patients required intensive care unit admission more frequently compared to 2R patients (75% vs. 5.7%; p<0.01), while 2R patients primarily stayed in the emergency department and then were discharged at home (47.1%).

The analysis of discharge outcomes using the GOS revealed a significant difference in disabling outcomes between the two groups, favoring the 3R patients.

Conclusions

The over-triage rate in the 2R group exceeds the recommendations provided by ACS-COT as described in the literature. Subjective evaluation of an objective criterion may underlie the high rate of over-triage for situational criteria. Although limited to a short study period, these findings highlight the need for an assessment of appropriateness in utilizing criteria for centralization of major trauma, including comparison with the TRENAU centralization system suggested by recent recommendations from the National Institute of Health.

**Table 1 (abstract A104).** Characteristics of the population

	ISS >15 (number of patients)	ISS <15 (number of patients)	OVER TRIAGE
Interior sheet metal intrusion (roof included) > 30cm on patient side or > 45cm on opposite side	3	15	88,33%
Pedestrian hit and thrown > 3 meters from the point of impact with the vehicle	2	7	77,78%
Vehicle precipitation > 3 meters	1	1	50,00%
Fall from a height > 5 meters (adult); fall > 3 meters, or in any case from 3 times one's height for children aged < 15 years	1	8	88,89%
Cyclist/motorcyclist thrown > 3 meters from the point of impact	0	8	100,00%

## Emergency medicine

### **A105 Treatment of narrow complex tachyarrhythmias in ICU: experience with landiolol**

#### Angela Grassi, Erica Plasmati, Francesca Caniglia, Rosella Nicoletti, Maria Grazia Schievenin, Francesco Massimo Romito

##### Terapia Intensiva Generale Ospedale Matera Asm Basilicata, Matera, Italy

###### **Correspondence:** Angela Grassi

*Journal of Anesthesia, Analgesia and Critical Care 2023,* **3(Suppl 1):**A105

Background: Narrow complex tachyarrhytmias (NCT) are common complications in ICU. Treatments are often focused on control heart rate.

We investigated efficacy and safety of landiolol, an ultrashort acting beta blocker, for treating NCT.

Material and Methods: We conduced an observational study (June 1, 2022 and December 23, 2022), including patients admitted to ICU of “Madonna delle Grazie” Hospital, in Matera (Italy). who developed a NCT.

Inclusion criteria: 18 years or older, hemodinamically stable NCT confirmed by ecg, normalized blood volume (ultrasound of inferior vena cava), apyrexia, absence of electrolyte alterations, use of advanced hemodynamic monitoring (MostCare™).

Starting iv dose of Landiolol was 10 mcg/kg/min, increased to 20-30-40 mcg/kg/min based on the clinical response of the patient.

Primary endpoints: heart rate (HR) reduction, increase in cardiac index (CI). Secondary endpoints: lack of hypotensive effect (by evaluating the main arterial pressure – MAP), reduction of systemic vascular resistance index (SVRI).

After stabilizing the clinical status of the patient, an oral beta blocker was introduced according to clinical practice. The iv infusion of landiolol was reduced by 50% within the first hour of initiating oral therapy and stopped after the second administration of oral therapy, with stable clinical status.

Hemodynamic parameters (HR, CI, MAP, SVRI) were recorded and analyzed at time zero, at the beginning of oral beta blocker (time 1) and the stop of landiolol infusion (time 2). The main values of each parameter (at time zero -1-2) were calculated.

Results: 10 patients were admitted to our protocol.

Middle age was 70.1 years. 3 patients were treated only with iv landiolol, because the occurence of major adverse events did not make it possible to set up the imbrication of oral therapy. Results are shown in Figure 1.

Conclusion: Further investigations are surely needed but we can suggest a global hemodynamic optimization of the patients, obtained by reducing HR, increasing CI, improving MAP and SVRI

References

1. Y. Matsuishi et al Evaluating the therapeutic efficacy and safety of landiolol hydrochloride for management of arrhythmia in critical settings: review of the literature, Vascular health and risk management, 2020: 16, 111-123;
2. R. Poveda Jaramillo, et al Ultra short acting beta blockers (esmolol and landiolol) in the perioperative period and in critically ill patients, Journal of Cardiothoracic and vascular Anesthesia, doi:10.1053/j.jvca.2017.11.039.

**Fig. 1 (abstract A105).**
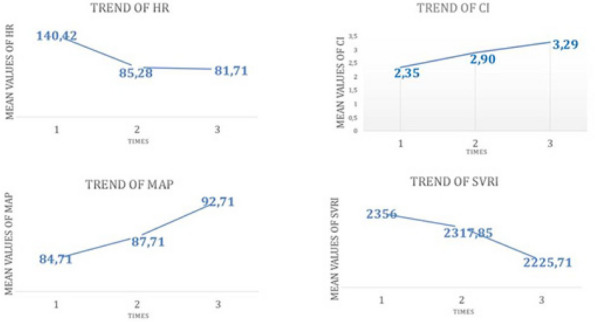
Variation in HR, CI, MAP, SVRI

### A106 Euglycemic diabetic ketoacidosis secondary to dapaglifozin: case report

#### N. Zarrillo ^1^, M. La Vedova ^2^, A. Carbone ^1^, R. Russo ^1^, G. Berlot ^3^

##### ^1^ ASL Caserta- PO San Rocco, Sessa Aurunca, Italy; ^2^ AORN Caserta Sant'Anna E San Sebastiano, Caserta, Italy; ^3^ Azienda Sanitaria Universitaria Integrata Giuliano-Isontina, Trieste, Italy

###### **Correspondence:** A. Carbone

*Journal of Anesthesia, Analgesia and Critical Care 2023,* **3(Suppl 1):**A106

Introduction

Diabetic ketoacidosis (DKA) is a serious and potentially life-threatening acute complication of diabetes mellitus (DM). According to the American Diabetes Association, the diagnostic criteria for DKA include blood glucose (BG) levels > 250 mg/dl, arterial PH < 7.3, anion gap > 12 mEq/L, HCO3- < 15mEq/L and the presence of Ketones in blood and urine. DKA can rarely occur with only mild to moderate glucose elevation, thus featuring the euglycemic DKA (EDKA). As the BG is < 200 mg/dL, the early diagnosis is challenging and the acidosis can be ascribed to causes other than EDKA. Its occurrence has been associated with the sodium-glucose cotransporter 2 (SGLT2)-inhibitors, a relatively new class of antidiabetic agents which achieve glycemic control by promoting glycosuria.

Case report

An obese 46-year-old man, with type2-DM treated with metformin and dapagliflozin, was admitted to the ED with coma and dyspnea; the recent history revealed a fever in the previous 5 days that was attributed to a respiratory infection. The initial vital signs were: temperature 36.1°C, heart rate 80 beats/min, respiratory rate 28 breaths/min, blood pressure 125/75 mmHg. The glycemia was moderately elevated (120.0 mg/dL) and the blood levels of creatinine, BUN and lactate were in the normal range; the arterial blood gases analysis (BGA) showed a severe metabolic acidosis with an elevated anion gap (PH < 7.00, PaCO2 21 mmHg, HCO3- 9.5 mmol/L, anion gap 28 mmol/L); elevated amounts of ketones were present in the urine.

He was promptly admitted to the intensive care unit and treated for EDKA via intravenous rehydration therapy with insulin infusion. Serial BGAs showed the gradual resolution of ketoacidosis with normalized anion gap and the clearance of serum ketones.

Discussion

The occurrence of SGLT2-inhibitors related EDKA has been associated to a number of precipitating factors, including infections, dehydration and surgery but not with the concomitant use of metformin. Although the pathogenetic mechanism of EDKA is not entirely clear, it has been hypothesized that the decrease of insulin secretion caused by the reduction of BG levels caused by SGLT2-inhibitors and the subsequent reduction of the utilization of free fatty acids could be associated an increase production of ketones. As the occurrence of SGLT2-inhibitors associated EDKA is a rare but life-threatening condition, it is important to consider this diagnosis in all patients treated with these agents especially in the presence of possible precipitating factors.

Authors confirm that informed consent to publish had been obtained from the patient.

### A107 Sudden respiratory failure in the operating room: suspected TRALI

#### E. Trimarchi, O. Mandraffino, T. David, G. Mazzeo

##### Policlinico Universitario G. Martino, Messina, Italy

###### **Correspondence:** E. Trimarchi

*Journal of Anesthesia, Analgesia and Critical Care 2023,* **3(Suppl 1):**A107

Background

78 year-old patient, heavy smoker, with myelofibrosis, and polycythemia vera come to the emergency room for multiple episodes of hematemesis.

Case Report

He was vigilant and collaborative, GCS 15, eupnoeic. Unstable hemodynamic; vital signs: HR 103 bpm, BP 91/58 MAP 69 mmHg, SpO2 99%. Ongoing RBC transfusion. After orotracheal intubation he has undergone EGD that was not diagnostic due to the presence of blood clots, so CT angiography was performed. There were diffuse pulmonary thickenings with partial conservation of the left upper lobe and probable blood spreading at the gastric level for which there was an indication to angiography and subsequent ultraselective embolization of the left gastric artery. During the angiography the patient developed acidosis with further reduction of Hb. Tranexamic Acid was administered and additional RBC unit was transfused. During the procedure respiratory exchanges sudden became worse with progressive desaturation; despite the increase of FiO2 up to 90% SpO2 was just about 85-88%. At the next BGA there was marked acidosis with an increase of lactates. He performed Thoracic Ultrasonography that excluded PNX but evidenced a lot of B lines in each lung field. The next chest CT showed the appearance of multiple consolidation. The patient was therefore admitted to ICU for further treatment. In the suspect of aspiration pneumonia, fibreoptic bronchoscopy was performed with evidence of fluid secretions in all explored sections. The suspicion diagnosis of TRALI arose. Thanks to interviews with family members emerged that the patient had carried out numerous transfusions in the previous weeks (about 10). Despite the critical general conditions, after 72 hours there was a progressive improvement in respiratory exchange documented by a reduction in pulmonary thickenings to the chest and an increase in P/F. During the stay, hemodynamic instability persisted and further EGD was performed; it was diagnostic for erosive esophagitis with mild bleeding and gastric stump ulceration. The patient also developed AKI and critical ischemia of the right lower limb with a simultaneous increase in CPK and Myoglobin values therefore he started CRRT.

Conclusions

Haemorrhagic shock due to gastrointestinal bleeding in an already poly-transfused patient who developed Transfusion Related Acute Lung Injury during angiography.

The informed consent to the publication had been obtained together with the consent necessary for hospitalization in ICU.

### A108 A case report of Leptospirosis during Covid-19 pandemic wave

#### E. Trimarchi ^1^, M. Scivoli ^2^, S. Di Stefano ^1^, A. Merendino ^2^, P. Giaquinta ^2^, C. Lo Giudice ^2^, M. Frisina ^2^, V. Brunetto ^2^, A. Barbagallo ^2^, G. Sercia ^2^, G. Terranova ^2^, G. Filoni ^2^, R. Leo ^2^

##### ^1^ Policlinico Universitario G. Martino, Messina, Italy; ^2^ P.O. San Vincenzo Taormina ASP 5 Messina, Messina, Italy

###### **Correspondence:** M. Scivoli

*Journal of Anesthesia, Analgesia and Critical Care 2023,* **3(Suppl 1):**A108

Background: a malaysian patient, indigent grower (living in poor hygienic conditions) came at the Emergency Department of the hospital. He was alert and cooperative, in spontaneous breathing. Good hemodynamic values, the abdomen treatable at superficial palpation, while painful at deep palpation with normal bowel movements and free passage of stool and flatus, body temperature 37°C. He complained of severe chest pain with dry skin and mucous membranes, dysuria and muscle pain. Positive Giordano manoeuvre on the left. The bladder catheter was positioned with approximately 400 ml of hyperchromic urine. He report fever for ten days ago but refused admission.

Case report. They showed direct hyperbilirubinemia and elevation of all hepatic cytolysis indices, marked hypermailasemia, and hight C-reactive protein. The renal parameters were also altered with marked hypercreatininemia and hypermyoglobinemia. In addiction he presented hyperfibrinogenemia and elevated d-dimer. Nasal-pharyngeal swab for the identification of Covid-19 was performed with negative result. Sierologic test was performed with negative result. Chest radiograph showed “extensive and bilateral thickening of cottony appearance partially confluent in the pulmonary parenchyma interesting all the lobes”; Thorax CT showed bilateral lung interstitial involvement with initial alveolar distortion and tendency to parenchymal consolidation in both lower lobes in the declivious pulmonary portions. The patient was admitted to the Internal Medicine Department with suspicion of initial phase of pulmonary sepsi, so he began broad-spectrum antibiotic therapy. Five days later the patient was alert but not more collaborating, agitated (RASS 4), with hypertension and sinus tachycardia. EGA were getting worse with SpO2 78% and P/F ratio < 100 mmHg. VAS 8 with strong chest pain, convulsive cough and emoftoe. The patient was sedate and IOT and transport him to Intensive Care Unit, where he continued meccanic ventilation. According to the patient anamnesis, laboratories parameters and his instrumental examination, we began presumption therapy in diagnostic suspicion of jaundice leptospirosis (Weil disease) with systemic antibiotic Bassado®. The patient had an immediate benefit, already at 24 h from the beginning of the therapy, based on EGA. After progressive reduction of sedation and accurate respiratory weaning, the patient was estubated.

Conclusion: the patient arrived during second wave with a symptomatology similar to Sars-Cov 2 infection; this led the attention of the heathcare professionist to the suspicion for COVID-19.

Thanks to the careful bibliographical research, the clinical history of the patient and the empirical therapy, the ICU team was able to make diagnosis of presumption of Leptospirosis.

Informed consent to publish had been obtained.

### A109 Advanced Hemodynamic monitoring in patients under V-V ECMO using the non – invasive Starling system based on bioreactance

#### G. settesoldi ^1^, F. Alessandri ^1,2^, F. Marinangeli ^3^, F. Pugliese ^1,2^

##### ^1^ Department of General and Specialistic Surgery, Sapienza University of Rome, Rome, Italy., Roma, Italy; ^2^ Department of Emergency Medicine, Critical Care Medicine and Trauma, AOU Policlinico Umberto I, Rome, Italy., Roma, Italy; ^3^ Departments of Anesthesiology and Pain Medicine (F.M., A.C., M.L., L.A., A.M., A.P., G.V.), and Oncology (G.P., P.M.), U, L'Aquila, Italy

###### **Correspondence:** G. settesoldi

*Journal of Anesthesia, Analgesia and Critical Care 2023,* **3(Suppl 1):**A109

Background

Patients on Veno-Venous Extra Corporeal Membrane Oxygenation (V-V ECMO) support are exposed to several alteration of the hemodynamic profile due the extracorporeal blood pump and the critical illness [1].

Several methods have been reported in order to monitor hemodynamics, including echocardiography and thermodilution.

Potential limitations for the use of these systems in patients undergoing an extracorporeal circulation or are not possible to performe in continuous [2].

In our center we used the Starling system based on Bioreactance to perform a continuous monitoring of hemodynamics value of 3 patients under V-V ECMO.

Bioreactance refers to the electrical resistive, capacitive, and inductive properties of blood and biological tissue that induce phase shifts between an applied electrical current and the resulting voltage signal.[3,4]

Case report

We start Starling monitoring on 3 patients at the start of V-V ECMO and we report the hemodynamics values like: CO, CI, SV, SVI, TFC, DO2I, the values of the cardio-help (QB, FdO2), the drug dosage (norepinephrine, furosemide) and every hour urine output.

ECMO treatment lasted 9.3 days (average), the QB never passed the 57,8 % of the CO of the patients, the norepinephrine dosage was never higher than 0.23 mcg/kg/min; in all cases we started with the 100% of FdO2.

Along with the improvement of the clinical conditions, the percentage of ECMO support decreased (68,6% at the start of ECMO support vs 43,2% at the last day), as with the same CO the TFC was reduced with negative balances (TFC medium 62.3 1000/ohm at the first day vs 32.2 1000/ohm at the last day) with a CO medium like 3.42 L/min. At the start of ECMO support the average DO2I was 375 ml/min/m2 vs 485 ml/min/m2 at the last day; we have never passed a balance more negative then -1420 ml/24h to avoid cavitation problems. We have never considerate the SVV because the Bioreactance monitor -like other several non-invasive hemodynamics monitor- needs a pre-established ml/PBW of TV which it was not possible to perform in patients who needs a protective ventilation due the ARDS.

Serial echocardiography were performed by different operators and showed CO values similar to the values detected by bioreactance. (Tab 1,2,3,4,5,6,7,8,9,10)

Conclusions

The bioreactance monitoring performed by Starling is a non – invasive method, easy to apply, accurated and with an important and accurable detection of hemodynamics value – especially in patients undergoing an extracorporeal support- that can guide the clinical practice.

Si esplicita la dichiarazione del consenso alla pubblicazione.

### A110 Use of arginine-vasopressin and landiolol in a patient with bowel perforation and septic shock: a case report

#### V.C. sanda ^1^, M. de rose ^1^, A. allushi ^1^, G. giordano ^1^, P. tozzi ^1^, F. alessandri ^1,2^, F. pugliese ^1,2^

##### ^1^ Department of Emergency Medicine, Critical Care Medicine and Trauma, AOU Policlinico Umberto I,, Roma, Italy; ^2^ Department of General and Specialistic Surgery, Sapienza University of Rome, Roma, Italy

###### **Correspondence:** V.C. sanda

*Journal of Anesthesia, Analgesia and Critical Care 2023,* **3(Suppl 1):**A110

Background

Arginine-vasopressin (AVP) is a synthetic equivalent of the endogenous hormone vasopressin and its hemodynamic effect is exerted mainly due to a vasoconstrictive activity induced by the binding of V1a receptors on vascular smooth muscle cells.[1] In patients suffering from septic shock, current guidelines suggest adding vasopressin if medium artery pressure (MAP) inadequate (i.e.<65 mmHg) despite low-to-moderate dose of norepinephrine, instead of escalating the dose.[2] Landiolol is an ultra short-acting, beta1-superselective adrenergic antagonist and its use has been reported for heart-rate control in patients with supraventricular tachyarrhythmias -including atrial fibrllation (AF)- in patients with sepsis.[3]

Case report

A 61-year old male was admitted to the emergency department of Policlinico Umberto I hospital, Rome, Italy, because of abdominal pain, diarrhea and swelling in the peri-umbilical area that began 5 days earlier. A computed tomography (CT) scan showed bowel perforation and the patient underwent ileal resection with ileoileal anastomosis. The patient was admitted to the intensive care unit sedated and intubated; hemodynamics was supported by norepinephrine 0.45 mcg/Kg/min despite optimal fluid resuscitation and the patient developed AF with high ventricular response (up to 160 bpm). Infusion of AVP was started at 0.03 UI/min, with subsequent reduction in norepinephrine dose (0.12 mcg/Kg/min). Landiolol was started at 10 mcg/Kg/min and escalated up to 30 mcg/Kg/min, with rapid control of heart rate. After 3 days of hemodynamic support and antimicrobial therapy, the improvement of clinical conditions led to the progressive reduction and further interruption of norepinephrine, AVP and landiolol. The patient was successfully weaned from mechanical ventilation and discharged to the surgical ward after a total of 10 days. He was discharged home after a total of 21 days.

Conclusions

During early septic shock several studies reported high endogenous vasopressin concentrations, that decrease to normal values as shock continues: the so-called “relative vasopressin deficiency”.[4] Given its high beta1-selectivity, landiolol has marginal effects on blood pressure and myocardial contractility and it’s effective in reducing heart rate to normal values.[5] In patients with septic shock and supraventricular tachyarrhythmias, studies show that ultrashort-acting beta1-blockers increased stroke volume index, while it reduced heart rate, leading to an improvement of cardiovascular efficiency maintaining the target MAP with lower dosage of catecholamines.[6] In this reported case, the use of AVP in addition to landiolol led to improve patient’s hemodynamics reducing the burden of catecholamines and heart rate. No adverse effects were reported during the combined treatment with AVP and landiolol.

Informed consent to publish had been obtained.

### A111 PRAM Method to investigate COVID-19 heart phenotype: an observational pilot study

#### G. Gaudino, M. Rauseo, A. Becchimanzi, D. Laforgia, D. La Bella, L. Mirabella, L. Tullo, G. Cinnella

##### Azienda Ospedaliero Universitaria Policlinico Riuniti Di Foggia, Universita' Degli Studi Di Foggia, Foggia, Italy

###### **Correspondence:** G. Gennaro

*Journal of Anesthesia, Analgesia and Critical Care 2023,* **3(Suppl 1):**A111

Background: Patients with COVID-19 have multiple reasons to become hemodynamically unstable, from hypovolemia (fever, fluid restriction to prevent the development of pulmonary edema) to vasodilation (sepsis, deep sedation during mechanical ventilation), and right or/and left ventricular dysfunction (mechanical ventilation with high PEEP, pulmonary embolism, circulating cytokines decreasing contractility, myocarditis) (1). However, the hemodynamic phenotype of these patients remains poorly documented (2). We aimed at using uncalibrated pulse wave analysis with the Pressure Recording Analytical Method (PRAM) (3) in order to characterize which of the derived indices may be suitable to detect a poor systolic function.

Material and Methods: We collected consent form and data from all consecutive patients > 18 y-o admitted to ICU from January to April 2021, with no pre-existent/known history of CHF and/or arrhythmia, positive nasal swab for Sars-Cov-2 and confirmed pneumonia by either chest x ray or CT, that were sedated, paralyzed and mechanically ventilated. A radial line was placed for continuos monitoring and ABGs. Data from MostCare Up collected on Day 1° , Day 3° and Day 7° at the same time of the day. Use of inotropes and/or vasoactive drugs was noted on CRF. Continuous variables are reported as mean ± standard deviation (SD) or median (interquartile range [IQR]). Data at different times were compared by analysis of variance (ANOVA) for repeated measures, if significant (P < 0.05).

Results and conclusion(s): We enrolled 11 adults patients, mean age 67 y-o. One patient (#9) died before day 3, five patients died before day 7 (#2,#3,#7,#8,#10). In day 1-3-7, Cardiac Index (CI, L(min*m2)) went from 2.25±0.43 to 2.55±0.41 to 2.48±0.58 (n.s.); Arterial elastance (Ea, mmHg/ml) decreased from 1.43±0.41 to 1.2±0.3 to 1±0.2 (p<0.05 Day 1 vs Day 7). Cardiac cycle efficiency (CCE, units) improved from -0.03±0.5 to 0.03±0.4 to 0.3±0.1 (p<0.05 Day 1 vs Day 3; Day 1 vs Day 7: Day 3 vs Day 7). Based on our observations, it seems that CCE is the most promising tool to detect different phenotypes in association to COVID-19 severity. In Fig. 1, we showed trends of variables coming from the 11 patients. Patients #2-3-4 were influenced by strong vasoconstriction or vasodilation; patients #5-6 had a reduction in both CCE and CI; patient #8, had a significant reduction in CCE, despite CI improved. Interestingly, patient #11 was very sick at the beginning, but then improved until survival. Moreover, while CI and Ea were almost stable, CCE increasing was statistically significant. These results showed that daily observation of cardiac function with PRAM method can provide detailed information about the overall mechanism by which cardiac dysfunction is occurring and that COVID-19 can induce cardiac damage in unique patterns and thus can be studied on a case-by-case basis, day-to-day during infection. This could allow to move toward more personalized cardiovascular medical treatment.

REFERENCES

1. Evrard, B. et al. Crit Care 24, 236 (2020).
2. Michard, F. et al, ICM 2021 Feb;47(2):254-255.
3. Scolletta, S. et al, 39, 1025–1033 (2013).

**Fig. 1 (abstract A111).**
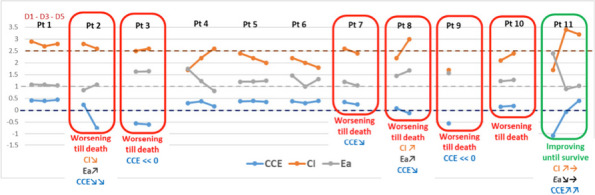
See text for description.

### A112 Bileaflet mitral valve prolapse - near miss sudden cardiac arrest: a case report

#### D. Pisani ^1^, A. Corriero ^2^, P. Ferrara ^1^, C. Ferrari ^1^, F. Puntillo ^2^, M. Ribezzi ^1^

##### ^1^ Anesthesia and Intensive Care Unit I - A.O.U.C. Policlinico, Bari, Italy; ^2^ Department of Interdisciplinary Medicine ICU Section University Aldo Moro, Bari, Italy

###### **Correspondence:** D. Pisani

*Journal of Anesthesia, Analgesia and Critical Care 2023,* **3(Suppl 1):**A112

BACKGROUND

Mitral valve prolapse (MVP) is a benign condition with a prevalence between 1% to 2.4%, more frequently in young females. A close association has been reported between MVP and sudden cardiac death (0,14% of the case) if there are more of these high-risk features [1] such as bileaflet valve, ST-T wave abnormalities in inferior leads and/or frequent complex ventricular ectopy on EKG, characteristic Pickelhaube sign [2] on ultrasound evaluation and papillar muscle/ventricular fibrosis on cardiac MRN.

CASE REPORT

A 33-year-old female patient was admitted to the ICU after sudden cardiac arrest, with a clinical history of mitral valve prolapse with myxomatous bileaflet valve and mild asymptomatic regurgitation. Her EKG history in the past 2 years reported inverted T waves in inferior leads on EKG, no drug intake and past breast augmentation surgery one month before the event.

The patient had collapsed by night as soon as she got out of bed, awakened by a noise.

A neighbour started CPR after five minutes while emergency service was called. After 45 minutes of cardiopulmonary resuscitation and six defibrillation shocks to treat ventricular fibrillation, ROSC occurred. Therefore she was taken to the emergency room, where she was intubated and treated until stabilization of vital signs. Coronary angiography and CT scan of the head were normal.

Subsequently, the patient was admitted to the ICU, in a state of coma (GCS 5/15 - E1V1M3), sedated and artificially ventilated in PRVC (VT 6 ml/kg) with P/F = 206, without electrolyte alterations, valid and spontaneous diuresis, gradual reduction of lactates (2.6 mmol/L), normothermia, increased but not clinically relevant troponins. The patient underwent ECG, with evidence of long QT, and control echocardiography with a post-arrest EF value of 25%, probably due to stunned myocardium. The neuron-specific enolase (NSE) samples reported slightly increasing non-significant values. Approximately 3 days after the acute event, the patient could open her eyes spontaneously without motor and sensory deficit with normal vital parameters, ultrasound and EKG for which she was extubated. Ten days after the event, she was transferred to the Cardiology Department, where an ICD was implanted.

CONCLUSION

MVP's clinical course is heterogeneous, with prognoses ranging from normal life expectancy to fatal cardiac arrest. Patients with high-risk features require careful monitoring.

Informed Consent Statement: Informed consent was obtained from a legally authorized representative of the subject.

References

1. Nalliah CJ, Mahajan R, Elliott AD, Haqqani H, Lau DH, Vohra JK, Morton JB, Semsarian C, Marwick T, Kalman JM, Sanders P. Mitral valve prolapse and sudden cardiac death: a systematic review and meta-analysis. Heart. 2019 Jan;105(2):144-151.
2. Muthukumar L, Rahman F, Jan MF, Shaikh A, Kalvin L, Dhala A, Jahangir A, Tajik AJ. The Pickelhaube Sign: Novel Echocardiographic Risk Marker for Malignant Mitral Valve Prolapse Syndrome. JACC Cardiovasc Imaging. 2017 Sep;10(9):1078-1080.

### A113 Rhabdomyolysis as cause, consequence or mimicker of Myocardial Infarction: a case report

#### M. Nasello ^1^, M. Ippolito ^1,2^, A. Federico ^2^, F. Ronga ^2^, A. Giarratano ^1,2^, A. Cortegiani ^1,2^

##### ^1^ Department of Surgical, Oncological and Oral Science (Di.Chir.On.S.), University of Palermo, 90127 Palermo, Italy;^2^ Department of Anesthesia, Intensive Care and Emergency, Policlinico Paolo Giaccone, University of Palermo, 90127 Palermo, Italy

###### **Correspondence:** M. Nasello

*Journal of Anesthesia, Analgesia and Critical Care 2023,* **3(Suppl 1):**A113

Background

Rhabdomyolysis is characterized by an insult to skeletal muscles, causing the release of intracellular contents into the bloodstream due to myolysis. Potential consequences are electrolytes disturbances and acute kidney injury (AKI). We present a case of temporal association between rhabdomyolysis and myocardial infarction with an unclear pathophysiological relationship.

Case Report

A 65-year-old male was admitted to the emergency department due to fatigue, myalgia and altered mental status. Patient's past medical history included chronic ischemic heart disease, hypertension, diabetes and mild chronic kidney disease. At admission, he was diagnosed with acute myocardial infarction (MI), basing on clinical presentation, EKG and myocardial biomarkers. Coronary angioplasty with drug-eluting stent placement in the right coronary artery was performed with success and a temporary pacemaker was positioned. However, a severe metabolic acidosis persisted, along with an increasingly severe hyperkaliemia (7.5 mmol/L) and hypotension. Further laboratory tests were then performed, showing a creatine phosphokinase >22000 U/L, myoglobin >30000 μg/L and serum creatinine 6.58 mg/dL. A diagnosis of rhabdomyolysis and AKI was posed and the patient was consequently admitted to the ICU. Treatment consisted of hemodynamic optimization with fluids and noradrenaline infusion, early CRRT with adsorbent filter (i.e. CytoSorb ®), bicarbonates and mannitol infusion. The patient stayed in ICU 5 days, with gradual but constant improvement of clinical status and lab test and was then transferred to the Coronary ICU. At 15 days, he is alive and hospitalized, with no residual worsening of kidney injury. The cause primarily triggering his clinical presentation is still unclear, as common triggers (e.g. statins) were ruled out. Association between MI and rhabdomyolysis has been reported, usually occurring after CPR and defibrillation. More reasonably, electrolytic alterations induced by rhabdomyolysis of unknown cause may have mimicked a MI in a patient with history of chronic ischemic heart disease [1] or a single causative factor can have affected myocardium and skeletal muscles simultaneously [2].

Conclusions

Rhabdomyolysis and MI may occur in the same clinical course. Myalgia, trend of cytolysis enzymes and electrolytes may help recognizing rhabdomyolysis in patients with criteria for MI. The trigger and pathophysiological association between the two events may be unknown.

The patient provided informed consent for the publication.

References

1. Patel R, Auraha N, Dyal H, Fernandez J, Nazneen W. Severe rhabdomyolysis induced electrolyte abnormalities mimicking ST elevation myocardial infarction. Chest. 2014; 146:115A.
2. Aisenberg G M, Fred H L. Does Myocardial Necrosis Occur in Rhabdomyolysis? Tex Heart Ist J. 2016; 43(3):203-204.

### A114 Tap-block as a diagnostic and monitoring tool in acute surgical abdomen: a case report

#### S. Tantillo ^1^, I. Sbaraini Zernini ^2^, F. Benvenuti ^1^, M. Guarnera ^1^, L. Giuntoli ^1^, F. Talarico ^1^, D. Mottola ^2^, S. D'Agostino ^2^, P. Peruzzi ^2^, D. Spacca ^2^, E. Gamberini ^1^, V. Rizzelli ^1^, I. Farinelli ^1^, M. Menghini ^1^, N. Cilloni ^1^

##### ^1^ Terapia Intensiva e HUB Maxiemergenze, Ospedale Maggiore Carlo Alberto Pizzardi, Bologna, Italy; ^2^ Dipartimento di Medicina e Chirurgia, Alma Mater Studiorum Università di Bologna, Bologna, Italy

###### **Correspondence:** I. Sbaraini Zernini

*Journal of Anesthesia, Analgesia and Critical Care 2023,* **3(Suppl 1):**A114

Backgorund: The transversus abdominis plane (TAP) block is a regional technique for anterolateral abdominal wall analgesia. It is widely used for postsurgical acute pain management, in the context of a multimodal opioid-sparing analgesia. The cornerstone of major abdominal surgery pain management is continuous epidural analgesia. However, especially in the ICU environment, the insertion of a epidural cathether, in addition to being affected by the coagulative arrangement, could be contraindicated by antiaggregation or anticoagulation therapy. It also required advanced technical skills. Moreover, TAP block presented fewer contraindication and it is a rather simple procedure with a shallow learning curve ant it provides long-lasting analgesia.

Case report: Patient, 67 years-old, admitted to ICU for post-surgical management after a duodenocephalopancreatectomy for cholangiocarcinoma. In 12th day he developed an acute abdominal pain, prevalent in the upper quadrants, radiating to the back, with a progressive anemization. The clinical pain manifestation, described by patient, seemed suggestive for acute post-surgical pancreatitis. We decided to make a TAP block for pain relief and to discriminate between visceral or somatic pain. Within few minutes, the patient was free of pain. So in the suspicion of hemorrhagic complication, as the pain trigger, we performed a FASTUS which revealed free fluid around liver and in the Douglas cavity. The patient was subjected to a CT confirming the US finding and he underwent an abdominal surgical procedure.

The patient's consent was requested for data processing and use for scientific publications.

Conclusions: We described a case report in which TAP block was successfully used in the differential diagnosis of an acute abdomen in critical care setting.

References

1. Dieu A, Huynen P, Lavand'homme P, Beloeil H, Freys SM, Pogatzki-Zahn EM, Joshi GP, Van de Velde M; PROSPECT Working Group of the European Society of Regional Anaesthesia and Pain Therapy (ESRA). Pain management after open liver resection: Procedure-Specific Postoperative Pain Management (PROSPECT) recommendations. Reg Anesth Pain Med. 2021 May;46(5):433-445. doi: 10.1136/rapm-2020-101933. Epub 2021 Jan 12. PMID: 33436442; PMCID: PMC8070600.
2. Niraj G, Kelkar A, Fox AJ. Application of the transversus abdominis plane block in the intensive care unit. Anaesth Intensive Care. 2009 Jul;37(4):650-2. doi: 10.1177/0310057X0903700420. PMID: 19681428.

**Fig. 1 (abstract A114).**
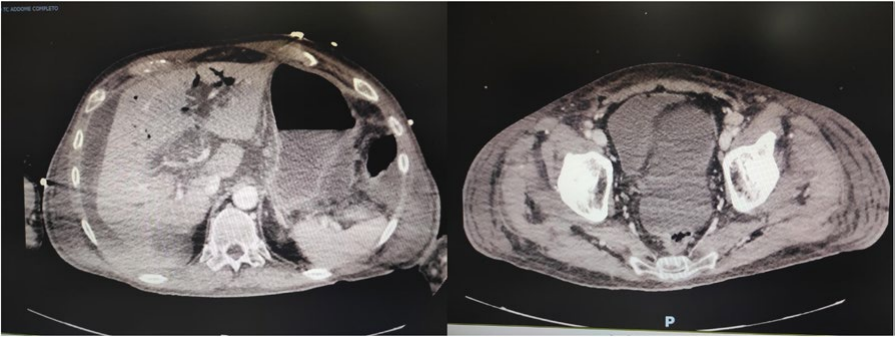
See text for description

### A115 Starvation Ketoacidosis in ICU: a hidden cause of hemodynamics instability

#### S. Tantillo, I. Ottaviani, M. Guarnera, F. Talarico, L. Giuntoli, I. Farinelli, E. Panigas, V. Rizzelli, F. Benvenuti, F. Mazzanti, N. Cilloni

##### Ospedale Maggiore Carlo Alberto Pizzardi, Terapia Intensiva, Bologna, Italy

###### **Correspondence:** M. Guarnera

*Journal of Anesthesia, Analgesia and Critical Care 2023,* **3(Suppl 1):**A115

Case presentation

A 35-years-old man was admitted to our emergency department (ED) after 7 days of fever, dyspnea, vomit and hypotension. At home, he was treated with chronic corticosteroid therapy due to pituitary surgery performed ten years before due to hypothalamic astrocytoma. He had no history of diabete mellitus or other diseases. At hospital admission nasal swab for SARS COV-2 was positive. He was admitted ICU because a suspicion of a septic shock. At the admission the patient was confused, drowsy but arousable, eupneic with minimal oxygen supplement. His hemodynamic state was characterized by tachycardia and mild hypotension. He was sub pyretic. Initial laboratory results revealed normal white blood cell count (7840/l), high level of C reactive proteine (29,13 mg/dl), procalcitonine (4 md/dl). A CT scan was performed revealing small infiltration pneumonia. After arterial catheter insertion an arterial blood gas analysis was performed: pH 7,34, PaCO2 37,8 mmHg, PaO2 94 mmHg, glycemia 103 mg/dl, base excess -5,5, lactic acid 0,7 mmol/l, HCO3- 20 mmol/l. Early management consisted in oxygen support, acetaminophen, empiric antibiotic therapy and fluid administration. During the stay fever and dyspnea rapidly improved while hypotension persisted and norepinephrine was started to maintain adequate mean arterial pressure. Urinary analysis was performed and revealed high urinary ketone bodies level (20 mg/ml). In the suspect of starvation ketoacidosis and related hypotension he was treated with 5% dextrose intravenous infusion at 125 ml/h. After 10 hours of glucose administration ketone bodies in urine were no more detectable, metabolic acidosis improved and norepinephrine was stopped due to normotension and hemodynamic stability. The patients was then discharged to a medical ward.

Written informed consent was obtained.

Discussion

Ketoacidosis is a potentially life-threatening metabolic disorder that occurs due to the accumulation of keto-acids in the body. Starvation ketoacidosis (SKA) has been described in the literature in the context of pregnancy and lactation and in preoperative states and with patients on a very low carbohydrate diet. In contrast to a well-fed state, a diminished supply of glucose stimulates gluconeogenesis, thus diminishing the supply of oxaloacetate and triggering the process of ketogenesis. Enzymes involved in ketogenesis are inhibited by insulin and stimulated by counter-regulatory hormones (glucagon, cortisol, epinephrine and norepinephrine). Management of SKA is focused on providing the substrate (i.e. intravenous glucose) that stimulates endogenous insulin production to restore insulin: glucagon ratio to normalcy. This case report shows that fast recognition of the causes of metabolic acidosis could reduce or avoid complications for the patients and futile admissions in ICU.

References

1. Bashir B, Fahmy AA, Raza F, Banerjee M. Non-diabetic ketoacidosis: a case series and literature review. Postgrad Med J. 2021 Oct;97(1152):667-671. doi: 10.1136/postgradmedj-2020-138513. Epub 2020 Nov 27. PMID: 33246966.
2. Morton A. Review article: Ketoacidosis in the emergency department. Emerg Med Australas. 2020 Jun;32(3):371-376. doi: 10.1111/1742-6723.13503. Epub 2020 Apr 7. PMID: 32266781.
3. Blanco JC, Khatri A, Kifayat A, Cho R, Aronow WS. Starvation Ketoacidosis due to the Ketogenic Diet and Prolonged Fasting - A Possibly Dangerous Diet Trend. Am J Case Rep. 2019 Nov 22;20:1728-1731.

**Fig. 1. (abstract A115).**
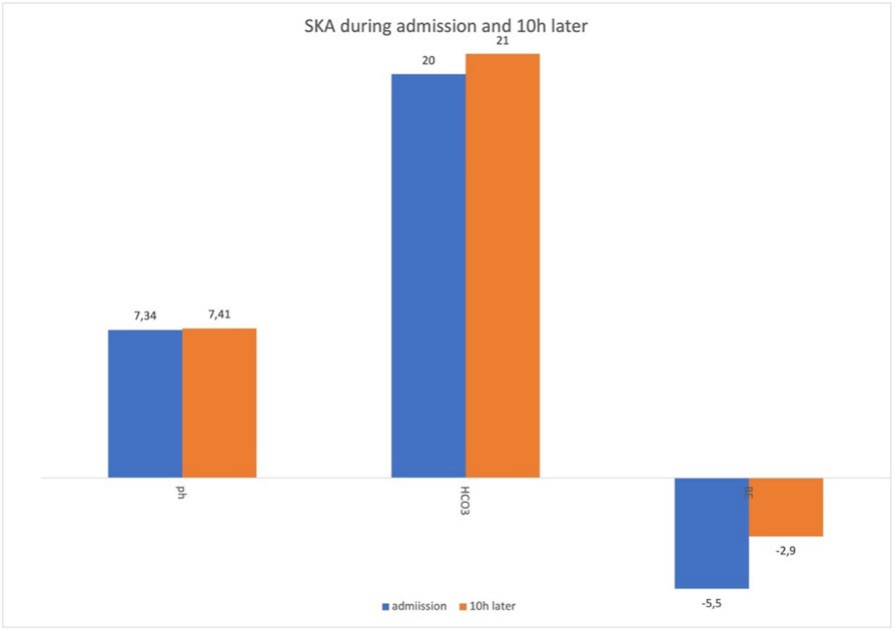
See text for description.

### A116 “Crack lung” ALI/ARDS as a cause of complex weaning from general anaesthesia for minor surgery

#### S. Tantillo, M. Guarnera, L. Giuntoli, F. Talarico, I. Farinelli, C. Della Casa, E. Panigas, A. Lacerenza, F. Moro, V. Rizzelli, F. Benvenuti, N. Cilloni

##### Ospedale Maggiore Carlo Alberto Pizzardi, Terapia Intensiva, Bologna, Italy

###### **Correspondence:** M. Guarnera

*Journal of Anesthesia, Analgesia and Critical Care 2023,* **3(Suppl 1):**A116

A 54-year-old man, ASA 2, underwent general anesthesia for laparoscopic cholecystectomy. After surgery the patient had a violent recovery from anesthesia featured with unconsciousness, psychomotor agitation. The patient was extubated but after a few minutes reintubated because of agitation and desaturation. He was admitted in ICU. Urine toxicology was positive for cocaine. He underwent computer tomography (CT): the brain CT was negative, while lung CT revealed bilateral ground glass opacity (Figure 1).

Echocardiography showed no evidence of cardiac failure. Based on urine toxicology, CT finding and subsequent bronchoalveolar lavage with a typical featuring of ALI/ARDS the patient was diagnosed with an acute pulmonary syndrome triggered by cocaine inhalation (crack lung). Gas exchange impairment was moderate so in the first days after surgery a weaning from sedation and mechanical ventilation was tried but the patient again was violent and unconsciousness. The patient was tracheostomized and treated with corticosteroid; after 1 week the CT shown a healing of the lung damage.

In the 8 days after surgery he was decannulated and completely recovered from neurological and respiratory symptoms (Figure 2).

Written informed consent was obtained from the individual for the publication of any potentially identifiable images or data included in this article.

Discussion

Crack lung it is uncommon in patients submitted to elective surgery. It is as an acute pulmonary syndrome consisting of diffuse alveolar damage and hemorrhagic alveolitis that occurs within 48 hours from smoking crack cocaine.1-2 Many patients require ventilatory support, either noninvasive positive pressure ventilation or endotracheal intubation with mechanical ventilation for respiratory failure. There are reports that patients benefited from corticosteroid in severe cases. This case help to recognize this respiratory disease also in surgical setting in a patient with cocaine abuse, but also shows how the cocaine abuser could have a complex weaning from general anesthesia and sedation; it could depend on the brain damage by the cocaine, non-visible in CT.

References

1. Dolapsakis C, Katsandri A. Crack lung: A case of acute pulmonary cocaine toxicity. Lung India. 2019 Jul-Aug;36(4):370-371. doi: 10.4103/lungindia.lungindia_193_19.
2. Restrepo CS, Carrillo JA, Martínez S, Ojeda P, Rivera AL, Hatta A, et al. Pulmonary complications from cocaine and cocaine-based substances: Imaging manifestations. Radiographics. 2007;27:941–56. doi: 10.1148/rg.274065144.

**Fig. 1 (abstract A116).**
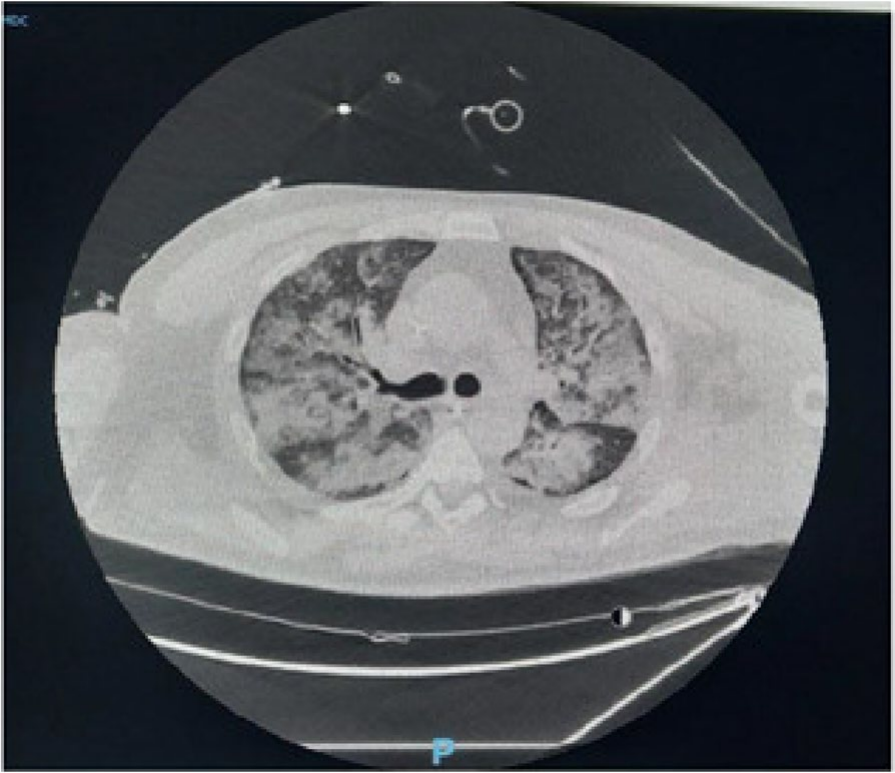
See text for description.

**Fig. 2 (abstract A116).**
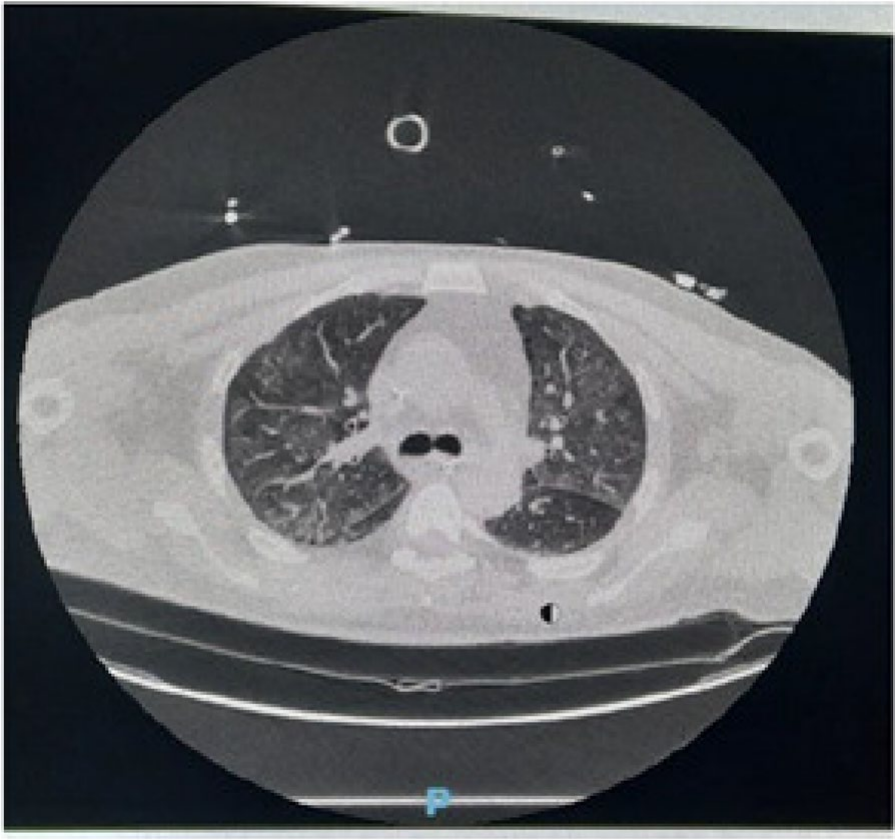
See text for description.

### A117 Diffuse alveolar hemorrhage in a young woman: some possible triggers of a withering evolution

#### E. Franceschi ^1^, F. Platto ^2^, P. Gnesin ^2^, E. Cogi ^2^

##### ^1^ Università degli Studi di Brescia, Brescia, Italy; ^2^ Servizio di Anestesia e Rianimazione, ASST Franciacorta, Chiari, Brescia, Italy

###### **Correspondence:** E. Franceschi

*Journal of Anesthesia, Analgesia and Critical Care 2023,* **3(Suppl 1):**A117

Case Report

A 30-year-old woman was admitted to the emergency room with cough, an episode of self-limited hemoptysis, chest pain and fever for 5 days, treated with NSAIDs and acetaminophen. She had a history of hypothyroidism on hormon replacement therapy. First exams showed SpO2 88% without oxygen, pO2 44 mmHg, an X-Ray revealed extensive consolidation in the left lung, INR was 1.27, PCR was 6.7mg/dl, other values were normal.

The patient was treated with painkillers, antibiotics, and methylprednisolone 40mg. At 12 hours there was a worsening of the pulmonary function; the patient was intubated. Subsequently she had a cardiac arrest (CA) treated with advanced life support (ALS) and a return of spontaneous circulation (ROSC) at 10 minutes. Hematic material was aspirated from the tracheal tube, moreover there was a widespread bronchial bleeding in a condition of hemodynamic shock. Tranexamic acid, fibrinogen, plasma, crystalloids and adrenaline were administered with poor response. Bleeding from rectum and pleural space were observed. Another CA lead to death despite 60’ of ALS.

Post-death analysis showed positivity for: antinuclear antibodies, cannabis (THC), streptococcus pyogenes and influenza A. Autopsy showed lung inflammation with lymphocytes cells, alveolar hemorrhage and thyroiditis.

Informed consent to publish had been obtained.

Background

Diffuse Alveolar Hemorrhage (DAH) is a high mortality (20-50%) condition associated with cough, fever, dyspnea, hemoptysis and ground-glass appearance to the CT evaluation.

The diagnosis is based on clinical criteria. The causes are numerous and can be attributed to infections, rheumatic diseases, drugs or toxins.

High doses of steroids (methylprednisolone 500-1000mg/die ev), immunosuppressants and plasmapheresis are recommended in DAH with inflammatory etiology although a sepsis status is an absolute contraindication. An antimicrobic drug is always recommended. The extracorporeal membrane oxygenation (ECMO) use is controversial due to coagulation disorders.

Conclusion

In this case, multiple co-factors are suspected to have overlapped: the immunoreactive setting in chronic thyroiditis (predisposing for lupus pneumonia or capillaritis), Influenza A and Streptococcus pyogenes infections and cannabinoid use may have lead to progressive and no responsive lung lesion. Due to a rapidly evolution and the absence of a clear cause there weren’t the conditions to save the patient. It remains unclear whether immunosuppressive therapy could has influenced the outcome.

References

- Danoff, S.K., & Hallowell, R. (2023). The diffuse alveolar hemorrhage syndromes. In P. Dieffenbach (Ed.), UpToDate. Retrieved March, 2023
- Endicott-Yazdani, T.R., Gannon, S. & Mora, A. Jr. (2018). The Bleeding Pneumonia - A Review of Diffuse Alveolar Hemorrhage, US Respiratory & Pulmonary Diseases. 3(1):33–6

### A118 Catheter tip in pericardium with unexpected cardiac tamponade : when chest X ray is not enough

#### P.F. Marsilia, M. Esposito, F. Imparato, A. Iodice, E. Capasso, M. Alfieri, M. De Cristofaro

##### AORN Antonio Cardarelli, Napoli, Italy

###### **Correspondence:** M. Esposito

*Journal of Anesthesia, Analgesia and Critical Care 2023,* **3(Suppl 1):**A118

Cardiac tamponade is a rare but potentially fatal CVC insertion complication; it is more frequent and described among pediatric population [1]. Clinicians often fail to consider the diagnosis in patient with typical signs and symptoms, which leads to an increasing mortality, potentially avoidable.

We describe the case of a male patient of 41 years, admitted in ICU for severe blunt chest trauma. CT imaging and eFAST were negative for pericardial effusion at admission. A central venous catheter was placed in the right axillary vein with ultrasound guide, the catheter tip position was confirmed by chest X-Ray. The patient was rapidly treated for pelvic bones stabilization and underwent arterial embolization to stop the bleeding. After 23 hours the patient 's haemodynamics deteriorated, CO dropped , lactate levels rose to 3 mmol/L, there were no signs of active bleeding , haemoglobin levels were stable, and noradrenaline infusion was started at 0.3mcg/kg/h restoring an acceptable Cardiac index ( > 2,5 ml/min/mq). A new echocardiogram showed for the first time a pericardial effusion, but not tamponade according to guidelines [2]. Considering the traumatic nature of the patient's injuries , CT scan was promptly performed, looking for the source of the presumed bleeding . Tomography showed iv contrast only progressing in pericardial space, the catheter was clearly visible outside the heart cavity., which was completely filled with contrast fluid. Right after the contrast dye injection, cardiac arrest occurred, and despite the efforts the patient died.

Cardiac tamponade in adults as a complication of central line insertion is rare and little known among doctors., this case report aims to put a red flag on this rare yet very insidious complication. Tamponade diagnosis furthermore is per se a clinical challenge [3], harder when the aethiology is a condition like a cvc misplacement.

We should all be aware that even chest Xray could be not enough to confirm cvc tip placement, and consider the catheter tip as a cause of pericardial effusion even when other causes can seem more likely.

Written informed consent for publication and /or study use, was obtained from the patient’s next of kin.

References:

A) Goutail- Flaud MF, Sfez M, Berg A, et al. Central venous catheter-related complications in newborns and infants :a 587 case survey. J Pediatr Surg 1991;26:645 – 50.
B) 2015 ESC Guidelines for the diagnosis and management of pericardial diseases: The Task Force for the Diagnosis and Management of Pericardial Diseases of the European Society of Cardiology (ESC). Eur Heart J. 2015 Nov 7;36(42):2921-64.
C) Ahmed Abuzaid, MD1 ; Mohamed Elkashab, MD2 ; Marwan Saad, MD. Cardiac tamponade, a clinical challenge. Journal of Clinical & Invasive Cardiology ISSN 2362-499X Volume 1,Number 1, 2014.

**Fig. 1 (abstract A118).**
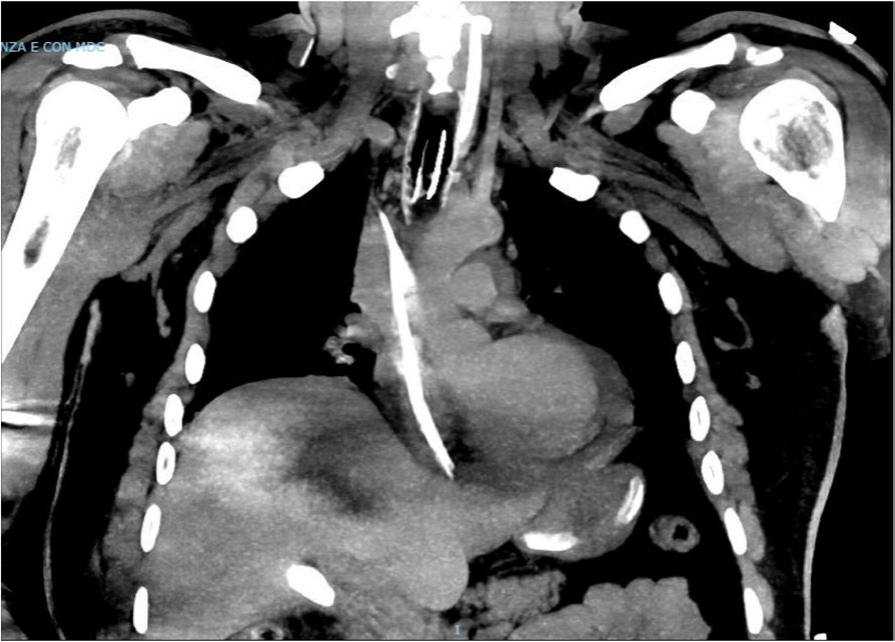
See text for description.

### A119 5-Fluorouracil intoxication: which signs to avoid the breaking point

#### F. Buffoli ^1^, P. Gnesin ^2^, G. Miglio ^2^, E. Cogi ^2^

##### ^1^Università degli Studi di Brescia, Brescia, Italy; ^2^ Presidio Ospedaliero Mellino-Mellini, Chiari, Italy

###### **Correspondence:** F. Buffoli

*Journal of Anesthesia, Analgesia and Critical Care 2023,* **3(Suppl 1):**A119

Background

Fluoropyrimidines, such as Fluorouracil and Capecitabine, are anti-neoplastic drugs commonly used in treatment of several types of malignancies; they have a broad spectrum of adverse effects that varies according to dose and schedule. Serious toxicities are rare but potentially lethal. Cardiac involvement is a poorly defined entity , especially according to the underlying pathophysiolgy and optimal management. Uridine triacetate is the only antidote approved for treatment of early onset, life threatening toxicities.

Case report

A 56 years-old male treated with neo adjuvant chemotherapy for gastric adenocarcinoma and discharged home with continuous infusion of 5-Fluorouracil, no other pathologies in anamnesis, was conducted to the emergency department (ER). The patient experienced neurological

deterioration and dysarthria after vomiting. During the first paramedics assessment the GCS was E4, M4, V4, pupils were physiological and the vital signs were stable. His neurological status of agitation was treated with diazepam, midazolam and anti-psychotics without any benefit. An acute neurological event was excluded by a CT brain; laboratory exams showed only a moderate worsening of the renal function. Few minutes later he developed a ventricular fibrillation (VF) with a return to spontaneous circulation (ROSC) after twenty minutes of advanced life support maneuvers (ALS). Transthoracic echocardiogram revealed severe global disfunction with hypokinesia of inferior, posterior and lateral cardiac walls. Urgent coronary catheterization did not demonstrate any significant stenosis. The patient was transferred to the intensive care unit (ICU) with high doses of inotropes and vasoconstrictors. In ICU a new episode of ventricular fibrillation occurs, which required ALS maneuvers. At post-ROSC ECG a chaotic rhythm persisted and soon evolved again in VF which was refractory to resuscitation maneuvers (delivered 18 shocks and overall 7 mg of adrenaline). Death was declared 8 hours after hospitalization. The patient, after exclusion of all reversible causes of cardiac arrest, was most likely a victim of 5-Fluorouracil intoxication expressed as a neurotoxicity and a cardiotoxicity. 5-Fluorouracil is associated with a wide range of adverse side effects infrequently lethal when identified early; cardiac complications are less common, and the pathophysiology is not fully understood. In this case encephalopathy and cardiomyopathy were rapidly progressive and did not allow to apply adequate therapeutic strategies.

Conclusion

Uridine Triacetate is the only Fluoropyrimidines antagonist available and it is administrable only orally, therefore cannot be given when the patient is severely unstable; furthermore, it is not at disposal in all hospitals. Another therapeutic strategy, unfortunately not available in our center, is the extracorporeal membrane oxygenation (ECMO) despite of a neoplastic disease is normally a contraindication to the procedure. In literature there are few cases report where the utilization of ECMO was used as a bridge therapy while the heart damage resolves spontaneously. Informed consent to publish had been obtained.

## Pediatric and Neonatal Perioperative Medicine

### **A120 Feasibility and safety of early High Frequency Oscillatory Ventilation in PARDS**

#### G. Chidini ^1^, L. Serio ^2^, M. Damiani ^1^, F. Virginia ^1^, L. Ughi ^1^, L. Orlandi ^1^, M.A. Figini ^1^, T. Marchesi ^1^

##### ^1^ Fondazione IRCCS Cà Granda Ospedale Maggiore Policlinico, Milano, Italy; ^2^ Università degli Studi di Milano

###### **Correspondence:** G. Chidini

*Journal of Anesthesia, Analgesia and Critical Care 2023,* **3(Suppl 1):**A120

Background High Frequency Oscillatory Ventilation (HFOV) is still considered as a rescue respiratory to treat children with severe PARDS when Conventional Mechanical Ventilation (CMV) fails to improve gas exchange or results in non-protective ventilation. However its use became even more controversial following two negative clinical trials in adult ARDS and two recent paediatric studies failing to show any benefit of HFOV on patient outcome. It’s still debated whether the outcomes of these studies confirm that HFOV is not beneficial, or even harmful, or that patient outcome was determined by the oscillator management strategy or time of application. Aim of this study was to review clinical data charts of children with PARDS (defined by PALICC criteria) managed with early HFOV during the course of illness. CMV was delivered in a volume controlled mode, tidal volume 6ml/kg, PEEP and FIO2 set according to the low limit of NIH Table. HFOV was delivered with a physiologic individualized open lung approach: the switch from CMV towards HFOV was planned in presence of: Oxygenation Index(OI)>13; Plateau Pressure>28cmH2O after PEEP optimisation. The return from HFOV to CMV was planned if: FIO2<0.4, mean airway pressure<18, normocapnia and no reduction in oxygen saturation during airway suction. We studied the feasibility of our strategy and examined the level and time course of metrics for pSOFA score, use of adjunctive therapies and long term respiratory follow up after hospital discharge.

Materials and methods. The study was approved by the local Ethical Committee, which waived the need for informed consent, as this was a retrospective analysis of prospectively collected data between January 2015 and December 2019. Basic clinical data were extracted from electronic clinical chart (Digistat; United Medical Software, Cerbaia, Italy) and included: demographic and antropometric data at admission followed by daily monitoring of ventilatory setting together with respiratory mechanics, pSOFA and physiological parameters. Data were collected at: 1) Intubation 2) pre-HFOV switch during CMV and 3) return to CMV from HFOV.

Results.In the study period 16 patients were allocated to receive early HFOV. Demographic and outcome measure are reported in Table 1.

HFOV was set at a mean PAW 25±2cmH20, Hz 9±0.5 and Power 45±5. HFOV was delivered for a median period of 3, 1,7-4.25 days. Days on CMV were 7, 6-8.5 with NIV postextubation days 3, 2-5.5. LOS PICU days was 13, 9.7-14.5. PICU and hospital mortality were 6%. Six out of 16 patients needed new hospitalization at six months and 2 out of 16 patients were discharged with home care ventilation. Respiratory mechanics was characterized by extremely low values of static compliance at admission, severe hypoxemia and elevated OI. HFOV resulted in a progressively increase in oxygenation, control of paCO2 and pH. However, HFOV resulted in more severe hemodynamic impairment, higher need of neuromuscular blockade and sedation, need of vasopressors and inhaled nitric oxide.

Conclusions. Early individualized HFOV in PARDS could be considered safe and effective in improving oxygenation and gas exchange in severe PARDS. Further studies are needed to explore potential HFOV advantages on CMV

**Table 1 (abstract A120).** See text for description

Male , n,%	7 (44)
Weight, Kg	4.75, 4.2-7.3
BSA, cm/m^2^	0.26, 0.24-0.36
Prematurity classification	
(<28 week)	3 (18)
(> 28 to 32 weeks)	0 (0)
(>32 to 34 weeks)	2 (12)
(>34 to 37 weeks)	3 (18)
Days before PICU admission	2, 1-3.25
Preintubation NIV Days	0,5-2
pre-HFOV CMV days	1, 0.6-2
HFOV Days	3, 1,7-4.25
CMV, cumulative days	7, 6-8.5
Post extubation NIV Days	3, 2-5.5
LOS PICU, days	13, 9.7-14.5
LOS Hospital, days	19, 13.7-21.7
PICU mortality, n %	1,6
Hospital mortality, n%	1,6
6 months mortality, n%	1,6
Hospitalisation at 6 months, n%	6,37
Discharge on home ventilation, n%	2,12

**Table 2 (abstract A120).** Respiratory mechanics on CMV. Data are expressed as median 1-3IQ Friedman t-test with Bonferroni post-hoc correction * p<0.01 compared to CMV day 1 and pre-HFOV switch

	CMV, day 1	pre HFOV switch	return to CMV
FIO_2_	0.6, 0.5-0.7	0.7, 0.65-0.85	0.6, 0.45-0.65
TV, ml/kg	7, 7-9	6, 5.5-6-7	7, 6.8-7.5
PEEP, cmH_2_O	8, 7-10	10, 8-12	10, 9-11
Pplat, cmH_2_O	25, 23-26	27, 26-29	24,23-25
Driving Pressure cmH_2_O	16, 13-20	17, 15-20	12, 10-14 *
Oxygenation Index	9, 8-11	11, 10-14	8, 6-9 *
Respiratory System Compliance (Crs,ml/Kg cmH_2_O	0.47, 0,31-0,61	0.43, 0.31-0.59	0.8, 0.6-0.9 *
NMB, n%	7,46	12,75	3,15
Prone position, n%	6,40	2,12	7,15
iNO, n%	3,20	6,35	2,12
Vasoactive agents usage, n%	2,12	3,18	-

**Fig. 1 (abstract A120).**
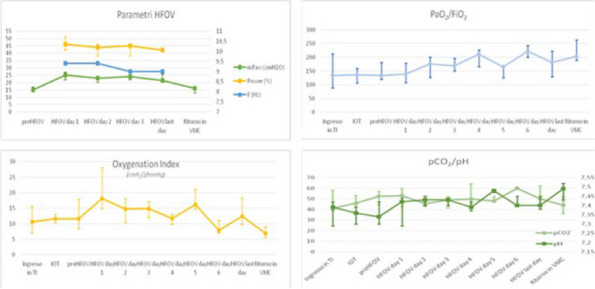
HFOV and physiological parameters

**Fig. 2 (abstract A120).**
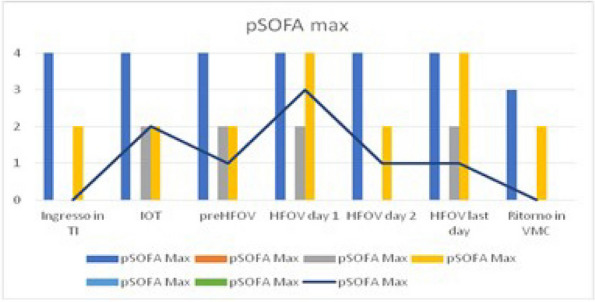
HFOV and pSOFA

### A121 Quantitative ultrasound assessment of gastric content in children undergoing deep sedation for magnetic resonance imaging

#### R. Lombardi ^1^, D. Posa ^2^, S. Sgrò ^3^, A. Vitale ^3^, D. Longo ^1^, S. Picardo ^3^

##### ^1^ Radiologia e Bioimaging - Ospedale Pediatrico Bambino Gesù, Roma, Italy; ^2^ Anestesia e Rianimazione, Terapia Intensiva e del Dolore . Università Cattolica del Sacro Cuore, Roma, Italy; ^3^ Anestesia, Rianimazione e Comparto Operatorio - Ospedale Pediatrico Bambino Gesù, Roma, Italy

###### **Correspondence:** D. Posa

*Journal of Anesthesia, Analgesia and Critical Care 2023,* **3(Suppl 1):**A121

Background

Children undergoing deep sedation need to fast to prevent the risk of aspiration of gastric contents. The ESAIC guidelines stipulate the fasting times to be observed [1]. Unnecessarily prolonged fasting times may increase the risk of post-procedure vomiting and nausea (PONV). Ultrasound (US) is a non-invasive technique capable of measuring the quantity of gastric residue, potentially allowing a reduction in fasting times.

Aims

The aim of the study was to ultrasonographically evaluate the quantity of gastric residue before the fasting times suggested by the ESAIC.

Materials and Methods

In this prospective observational study, children with neurological, neurosurgical or neuropsychiatric conditions, scheduled for magnetic resonance imaging (MRI) under deep sedation at the Bambino Gesù Pediatric Hospital, San Paolo site, were enrolled. Informed consent was obtained from all patients in accordance with local ethical committee guidelines. The study included children aged less than 18 years, whose last meal consisted of cow's milk or formula, breast milk or clear liquids. Patients with slowed gastric motility were excluded. The content in the gastric antrum was quantified in the pre-operative holding area with the patient in the supine and/or right lateral decubitus position, performing sagittal and axial scans in the epigastric region using the SIEMENS ACUSON s3000 ultrasound machine. The volume of the endoluminal material was calculated with the ultrasound system's automated software. After adhering to ESAIC-recommended fasting guidelines, children underwent deep inhalation sedation (oxygen, air and sevoflurane) with a facial mask in spontaneous breathing. No antiemetic drugs were administered. The presence of PONV was recorded.

Results

Twenty-four children aged between 2 months and 12 years were enrolled. Thirteen children consumed water an average (SD) of 67 (28) minutes before the ultrasound evaluation, exhibiting a gastric residue of 0.25 (0.4) ml/kg. Eight children were fed artificial or cow's milk 199 (92) minutes before the ultrasound evaluation, demonstrating a gastric residue of 2.2 (2.1) ml/kg. Three children were fed breast milk 170 (60) minutes before the ultrasound evaluation, presenting a gastric residue of 0.10 (0.2) ml/kg (Table 1). Four children underwent an ultrasound assessment less than 1 hour after their last clear liquid intake; in the absence of any gastric residue, deep sedation was deemed safe [2,3]. Only 2 children experienced PONV, with no complications recorded.

Conclusions

The study confirms that the fasting times stipulated by the guidelines allow for full emptying of gastric contents. Moreover, gastric ultrasound appears to be a feasible technique for assessing gastric residual, potentially enabling the safe reduction of fasting times.

References

1. Frykholm P. et al. Pre-operative fasting in children. Eur J Anaesthesiol 2022; 39:4–25
2. Leviter J. et al. “Full Stomach” Despite the Wait: Point-of- care Gastric Ultrasound at the Time of Procedural Sedation in the Pediatric Emergency Department. EMERGENCY MEDICINE • July 2019, Vol. 26, No. 7
3. Perlas A. et al. Point-of-care gastric ultrasound and aspiration risk assessment: a narrative review. Can J Anesth/J Can Anesth (2018) 65:437–448

**Table 1 (abstract A121).** Time to ultrasound assessment with gastric content volume and total time to procedure start. Data are reported as means (SD)

Type of fluid	Time of fasting at US assessment (minutes)	Gastric fluid volume (ml/kg)	Time of fasting at the start of the procedure (minutes)
**Clear fluid**	67 (28)	0,25 (0,4)	88 (31)
**Breast milk**	170 (60)	0,10 (0,2)	201 (51)
**Milk - Formula milk**	199 (92)	2,2 (2,1)	236 (95)

## Nutrition, metabolism and renal therapy

### **A122 Nutrition under noninvasive ventilation in critically ill patients: a retrospective monocentric analysis**

#### T. Esposito ^1,2^, F. Moretto ^1,2^, F. Verdina ^1,2^, M. Fracazzini ^1^, L. Bertali ^1^, M.L. Donnarumma ^1^, A. Magli ^1^, F. Minelli ^2^, M. Zuliani ^1^, B. Barone ^1^, L. Scotti ^1^, S. Riso ^3^, G. Cammarota ^1,2^, D.C. Francesco ^1,2^, R. Vaschetto ^1,2^

##### ^1^ Università del Piemonte Orientale, Dipartimento di Medicina Traslazionale, Novara, Italy; ^2^ Azienda Ospedaliero Universitaria Maggiore della Carità, Anestesia e Rianimazione, Novara, Italy; ^3^ Azienda Ospedaliero Universitaria Maggiore della Carità, Scienza dell'alimentazione e dietetica, Novara, Italy

###### **Correspondence:** T. Esposito

*Journal of Anesthesia, Analgesia and Critical Care 2023,* **3(Suppl 1):**A122

Background

Critically ill patients in Intensive Care Unit (ICU) are frequently malnourished. Noninvasive ventilation (NIV) often poses the necessity to start artificial nutrition, but data and recommendations about the appropriate nutritional support in NIV are still very limited. We aimed to describe the characteristics and nutritional management of patients undergoing NIV in ICU, and to assess potential associations with patient outcomes.

Methods

We reviewed the electronic records of adults in our ICU undergoing NIV for acute respiratory failure (ARF) for more than 48 hours, from March 2020 to January 2023. Population characteristics and nutritional management were described, as well as NIV settings, complications, need for tracheal intubation (ETI), ICU/hospital length of stay, and mortality. The ethics committee waived the requirement to obtain any informed consent for data collected retrospectively.

Results

A total of 112 patients were included (75 males/37 females), with a mean age of 65 (±12), and a median body mass index (BMI) of 27.76 (25.71-34.44) Kg/m2, with 45 obese subjects. ICU admission diagnosis were ARF (65%), septic (9%) and hypovolemic (9%) shock, while major comorbidities were hypertension (55%), cardiopathy (40%) and respiratory disease (38%). NIV was used in 96 cases for hypoxemic and in 16 cases for hypoxemic-hypercapnic ARF, with a median treatment duration of 69 (42-95) hours over 3 (2-5) days. Patients were on nutritional support for 5 (2-10) days during ICU stay, and time lag between NIV initiation and nutrition start was 1 (0-2) day on average. Enteral nutrition was administered in 25 patients, parenteral in 41, while both routes in 28 patients. Eighteen patients received no nutrition or oral nutritional support. Median caloric intake was 9.65 (5.23-12.91) KCal/Kg/die, with a total protein amount of 0.40 (0.17-0.57) g/Kg/die. Patients on parenteral and on both routes received a higher caloric and protein intake and had a longer NIV treatment duration (p<0.001). Maximal caloric intake reached was 13.62 (±7.74) KCal/Kg, and it was administered for 1 (1-3) day in median, with a time lag between minimal and maximal caloric intake of 1 (0-3) day. Infection occurrence was 25%, while ETI was necessary in 39 (35%) patients. Pressure sores were observed in 17 cases. Lastly, ICU length of stay was 6 (4-10) days on average, and ICU mortality was 21%.

Conclusions

Patients treated with NIV in ICU were given early nutritional support, but median caloric and protein intakes resulted globally low. Further prospective studies are necessary to understand the appropriate nutritional support in NIV.

### A123 Has a “Popeye” lung transplant recipient better outcomes? Retrospective evaluation of nutritional and muscular indexes in a large cohort of lung transplant recipients

#### S. Congedi ^1^, D. Lovison ^1^, M. Biscaro ^1^, M. Nardelli ^1^, C. Legnaro ^1^, A. De Carolis ^1^, T.A. Giacon ^1, 2^, M. Bassi ^1^, I. Lupelli ^1^, P. Navalesi ^1^, A. Boscolo Bozza ^1^

##### ^1^ Institute of Anaesthesia and Intensive Care Unit, Padua University Hospital, via V. Gallucci 13, 35125, Padua, ITALY; ^2^ Department of Biomedical Sciences, Environmental and Respiratory Physiology, University of Padova, Via Marzolo 3, 35131, Padua, ITALY

###### **Correspondence:** S. Congedi

*Journal of Anesthesia, Analgesia and Critical Care 2023,* **3(Suppl 1):**A123

Background: Nutritional status has been shown to play a primary role in influencing short and long-term outcomes also in solid organ transplant recipients [1,2]. However, specific data concerning lung transplants (LT) are lacking. The aims of the study are: i) describing the preoperative nutritional and muscular status before LT and, ii) investigating which nutritional and sarcopenia indices are predictors of postoperative outcomes of interest, as duration of postoperative invasive mechanical ventilation (IMV), need for re -intubation, length of stay in intensive care unit (ICU) and hospital, and rejection at 30 days.

Materials and methods: This single-center retrospective study evaluated for enrollment all consecutive adult patients undergoing bilateral LT at the University Hospital of Padua (between February 2016 and November 2020). The exclusion criteria were: i) admission to ICU before LT, ii) re-transplant, iii) pre-existing muscular or skeletal diseases. For each enrolled patient, the following data, assessed within six months before LT, were collected: a) nutritional indices (Body Mass Index (BMI), serum albumin level, Prognostic Nutritional Index (PNI), Mini Nutritional Assessment Short-Form (MNA-SF)); b) indices of sarcopenia (Creatinine Height Index (CHI), Skeletal Muscle Index (SMI) and densitometry of paravertebral muscles on chest CT); and c) clinical data related to the outcomes of interest. Univariable logistic regression models were used for binary outcomes and univariable Gamma models for continuous outcomes.

Results: 108 patients were included in the study, of which 72 (67%) were male. Nutritional and muscular indexes collected from our cohort were reported in Table 1. Longer duration of IMV was associated with lower preoperative albumin (p-value <0.010), as shown in Figure 1.

The increase of ICU stay was related to lower preoperative albumin levels (p-value <0.010), PNI (p 0.010) and BMI (p-value 0.040), respectively. No nutritional or muscle parameters predicted the need for reintubation, the onset of rejection at 30 days and the length of hospital stay.

Conclusions: Most of the patients undergoing bilateral LT in Our experience presented a nutritional and muscular status within the normal limits. Pre-transplant serum albumin values correlated with the duration of IMV, while serum albumin, PNI and BMI were associated with prolonged ICU stay. On the contrary, the proposed indicators of sarcopenia did not show any correlation with the investigated outcomes. Further studies are necessary to validate our results and to investigate, on a larger sample, newer association between nutritional status and outcomes of interest.

REFERENCE

1. McWhirter JP, Pennington CR. Incidence and recognition of malnutrition in hospital. BMJ. 1994 Apr 9;308(6934):945-8. doi: 10.1136/bmj.308.6934.945. PMID: 8173401; PMCID: PMC2539799.
2. Nosotti M, Ferrari M. Nutritional status and lung transplantation: an intriguing problem. Ann Transl Med. 2020;8(3):44. doi:10.21037/atm.2019.12.62.

**Fig. 1 (abstract A123).**
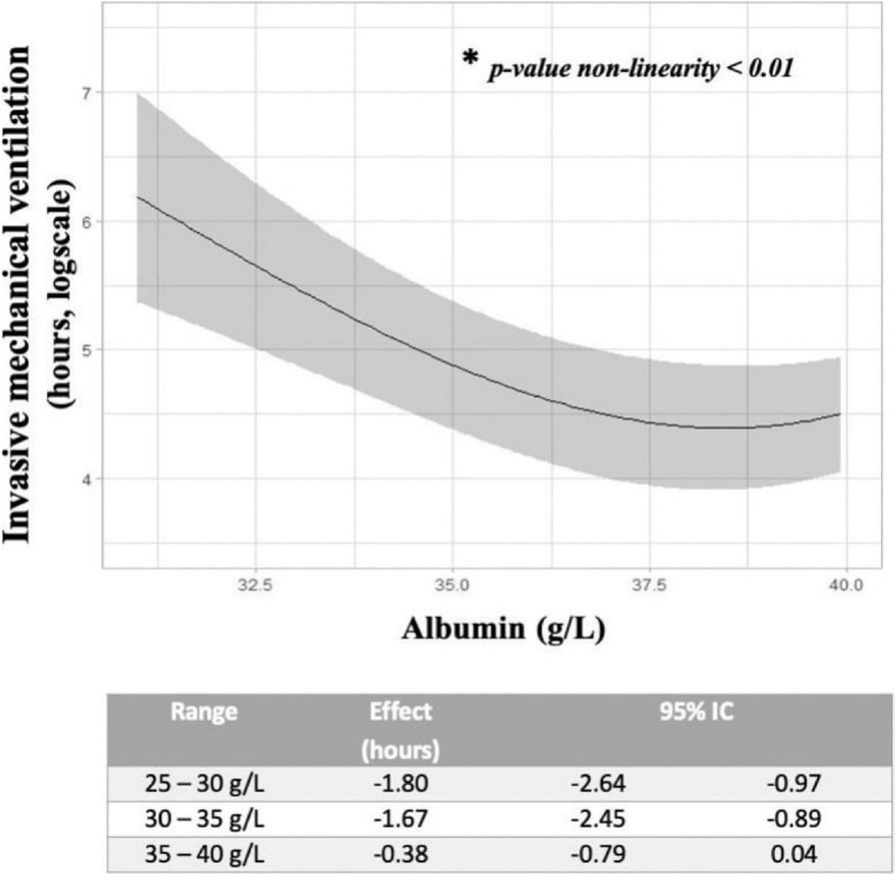
Relation between invasive mechanical ventilation and preoperative serum albumin (non-linearity)

**Table 1 (abstract A123).** Nutritional and muscular indexes in bilateral lung transplant recipients (data are expressed as median and [interquartile range])

Nutritional and muscular indexes	Bilateral lung transplant recipients (n=108)
***BMI, kg/m*** ^***2***^	23.30 [20.70-27.40]
***Albumin, g/L***	39 [36-42]
***Prealbumin, mg/L***	232 [186-276]
***PNI***	50.00 [45.00-54.00]
***MNA-SF score***	13 [12-14]
***CHI (%)***	80 [59-100]
***Muscular density(Hu)***	36.90 [31.80-41.30]
***Muscular volume*** (***mm***^***3***^***)***	65169 [53096-78821]
***SMI (cm*** ^***2***^ ***/BSA)***	36.50 [31.70-40.80]
Sarcopenia*, n (%)	14 (17)

*Abbreviations: BMI* body mass index, *PNI* Prognostic Nutritional Index, *MNA-SF* Mini Nutritional Assessment Short-Form, *CHI* Creatinine Height Index, *SMI* Skeletal Muscle Index

* Sarcopenia was defined as densitometry of paravertebral muscles on chest CT <30 Hu

### A124 A case of reversible metabolic coma in chronic liver disease; can vitamin D make a difference?

#### B. Basta, D. Vailati, F. Della Mura, G. Marino

##### ASST Melegnano Martesana, Vizzolo Predabissi, Italy

###### **Correspondence:** B. Basta

*Journal of Anesthesia, Analgesia and Critical Care 2023,* **3(Suppl 1):**A124

BACKGROUND

Pathogenesis of hepatic encephalopathy (HE) is complex and multifactorial; elevated ammonia levels had been considered as primary cause, but other factors have been advocated. Vitamin D deficiency is common in hepatic patient and it could be correlated to HE gravity according recent findings.

CASE REPORT

We report a case of 73 years man suffering from chronic liver disease (CLD) reversed by vitamin D administration. He presented in Emergency Room with neurologic impairment and hyperammonaemia (187 microg/dl). He was drowsy, able to localize pain stimuli and to say confused words. Lactulose and branched-chain-amino-acids treatment was undertaken; metabolic acidosis was corrected by NaHCO3 infusion. After a few hours epileptic attack was manifested, hence antiepileptic treatment was started (levetiracetam 500 mg bid). A CT scan excluded haemorrhagic or ischemic strokes. Neurologic status got worsen quickly, till coma status; patient was intubated and moved in Intensive Care Unit. After 48 hours treatment, ammonia levels were normalized. Under antiepileptic therapy no epileptic electroencephalogram activity was registered. Electrolytes were in range, acid-base balance was corrected. Any sedation drug was stopped since more of 24 hours, but neurological status was still impaired (RASS+3/-2; CAM-ICU negative for delirium) with evidence of Kussmaul breathing pattern. Vitamin D severe deficiency, detected initially (5,7 ng/ml [severe deficiency<10 ng/ml; insufficiency 10-30 ng/ml; toxicity>100 ng/ml]), but overlooked, was treated with cholecalciferol 100.000 IU IM. After administration, patient recovery was observed in the follow 24 hours; patient was promptly extubated; he was able to fulfil orders and to answer to simple questions; he moved to medical ward in 2 days. After one week from administration, Vitamin D level was increased, defining insufficiency (12,4 ng/ml) state.

CONCLUSION

Vitamin D is a multifunctional steroid hormone; it is involved also in normal functioning on human brain. Vitamin D deficiency was observed in many neuropsychiatric conditions (autism, depression, schizophrenia, cognitive decline, dementia). In CLD, patients with HE have been shown to have significantly lower vitamin D levels than non encephalopathic ones. This correlation suggests that vitamin D deficiency may have an unrecognized role in the development of HE. This case report, limited per se, supports this possible causal association, considering that vitamin D supplementation is like to have determined or at least contributed to neurological recovery of our patient. Maintenance of vitamin D levels within normal range could become part of clinical management of patients with CLD, also in acute setting.

DECLARATIONS

Consent to data collection and publication was obtained from the patient

## Neuroanesthesia and critical care

### **A125 Normal values of the new QPI index from automated pupillometry**

#### S. Zorzi, A. Ayako Minemura Ordinola, S. Vathi, F.S. Taccone

##### Department of Intensive Care, Hôpital Universitaire de Bruxelles (HUB), Université Libre de Bruxelles (ULB), Bruxelles, BELGIUM

###### **Correspondence:** S. Vathi

*Journal of Anesthesia, Analgesia and Critical Care 2023,* **3(Suppl 1):**A125

Background

Automated infrared pupillometry is a reliable, reproducible, and objective measure of the pupillary light reflex (PLR), a valuable tool in the evaluation of potential intracranial pathologies and brainstem dysfunction [1,2]. Moreover, an index derived from one pupillometry, the Neurological Pupil Index (NPi) has important prognostic value in anoxic and non-anoxic brain injury [2]. Recently, another pupillometry device, has proposed a new index called the Quantitative Pupillometry Index (QPI) which is based on the constriction response to light stimulation, ranging from 0 (no response) to 5 (optimal response). However, there is currently no data available on QPI values in healthy subjects or its usefulness in patients with brain injury.

Materials and methods

Adult subjects were included between January and February 2023 at the Hôpital Universitaire de Bruxelles (HUB), Belgium. They were tested in a constant semi-dark environment, after sufficient time to adjust to the dark. Both eyes were sequentially tested; testing was repeated 3 times for each eye, with an interval of 3 minutes between measurements. Together with QPI values, pupil diameter, pupillary constriction, latency and velocity, as well as demographic data, were also collected. Descriptive statistics were computed for all variables.

Results

75 healthy volunteers were included; median age of the study cohort was 30 [28-39] years; 44 (59%) of subjects were female. The most frequent visual defect were myopia (n=32, 43%), astigmatism (n=16, 21%) and hypermetropia (n=6, 8%). At the first measurements, medians pupillary diameter was 6.5 [5.6-7.1] mm in the right eye and 6.2 [5.4-7.0] in the left eye, respectively. All QPI measurements from both eyes scored 5.

Conclusions

The results confirmed consistency in repetitive measurements of the QPI, indicating a value of 5 as normal for this parameter. This information will be useful for future studies that evaluate QPI in brain injured patients, where comparison with normal values is necessary.

References

1. Kardon R. Pupillary light reflex. Curr Opin Ophthalmol 1995; 6(6):20-26.
2. Sandroni C, Citerio G, Taccone FS. Automated pupillometry in intensive care. Intensive Care Med 2022; 48(10):1467-1470.

### A126 Reverse Takotsubo cardiomyopathy after subarachnoid hemorrhage: a case report

#### V. Squillace ^1^, E. Bertoni ^2^, L. Cabrini ^2^

##### ^1^ Dipartimento di Anestesia e Rianimazione Neurochirurgica e Generale, Ospedale di Circolo - ASST Settelaghi, Varese, Italy; ^2^ Università degli Studi del'Insubria, Varese, Italy

###### **Correspondence:** E. Bertoni

*Journal of Anesthesia, Analgesia and Critical Care 2023,* **3(Suppl 1):**A126

Introduction

Takotsubo cardiomyopathy (TTC) affects up to 8% of patients with subarachnoid hemorrhage (SAH), particularly in most severe SAH cases (1). Nearly half of SAH patients presenting TTC develops acute cardiac failure; TTC is associated with an increased risk of in-hospital death or long-term neurological disabilities compared to SAH patients without it (1). It is therefore fundamental to detect the presence of TTC in SAH patients.

Beside the typical left ventricular apical “ballooning” with decreased ejection fraction, SAH patients can present less common TTC patterns not involving the apex nor contractility (1-4).

We present a case of “reverse TTC” (rTTC) associated to SAH, characterized by normal apex function.

Informed consent was obtained for this study.

Case report

A 63 years old woman was found at home confused, with a severe headache. An angio-CT brain scan revealed a widespread SAH after rupture of an aneurysm of the anterior cerebral artery. The Hunt and Hess grade was III [Fig.1].

An external ventricular derivation was placed, then the patient was admitted to the NeuroICU. The following day she underwent embolization of the aneurysm Two days after the event negative T waves where noticed [Fig 2], while hemodynamic deteriorated and norepinephrine was started.

An echocardiography was performed, showing hypokinesia of the middle wall of the left ventricle, with an ejection fraction of 50% [Fig.3]. A diagnosis of rTTC was made and bisoprololo was administered, while norepinerhine was gradually reduced.

The following day both the electrocardiogram and echocardiogram abnormalities were greatly improved. No other complications were observed, and the patient was transferred to a rehabilitation center one month later, after full neurological recovery.

Discussion

One third of TTC presents non-typical patterns, with abnormalities limited to the mid left ventricular wall, or to the basal wall or to focal areas (1). Of note, rTTC seems to be associated with the same prognosis of the typical TTC, even if rTTC might resolve faster (1). rTTC might be triggered by a damage of the cerebral cardiovascular regulatory areas, whereas typical TTC is triggered by a stress release of catecholamine (2). Both patterns share the same therapeutic options, particularly the need to limit vasopressors and inotropic agents.

Conclusion

In SAH patients, in case of electrocardiographic abnormalities above all if associated to hemodynamic deterioration, the absence of a left ventricular apical ballooning does not exclude TTC, as less common patterns are possible and are associated with the same risks.

References

1. Fujita T, Nakaoka Y, Hayashi S et al. Incidence and clinical characteristics of Takotsubo syndrome in patients with subracchnoid hemorrhage. Int Heart J 2022;M 63:517-523.
2. Mohammad SI, Bennson S, Wazim M and Kushak S. cardiac dysfunction in neurocritical care: an autonomic perspective. Neurocrit care 2019; 30:508-521.
3. Kumai T, Inamasu J, Watanabe E et al. Differences between Takotsubo cardiomyopathy and reverse Takotsubo associated with subarachnoid hemorrhage. IJC Heart and vasculature 2016; 11:99-103.
4. Mahanna E, Edwards DA, tarante N et al. Variant neurogenic stunned myocardium in a young female after subarachnoid hemorrhage. AA Case Rep 2016; 6:10-13.

**Fig. 1 (abstract A126).**
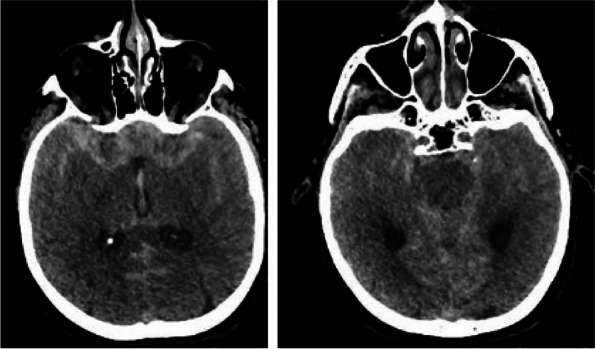
Widespread SAH

**Fig. 2 (abstract A126).**
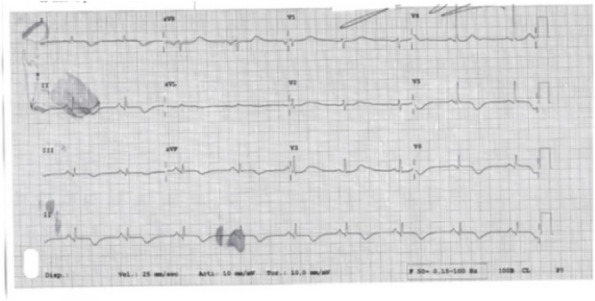
Diffuse negative T waves on the 12-lead electrocardiogram

**Fig. 3 (abstract A126).**
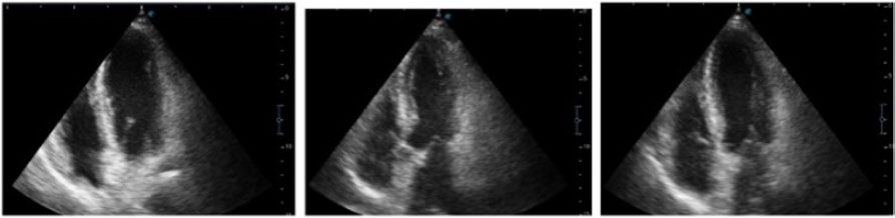
Echocardiography images

### A127 Management of severe traumatic brain injury in a Jehovah's Witness young woman: a case report

#### M. Pillitteri ^1^, L. Vegnuti ^2^, B. Ferro ^2^, P. Roncucci ^2^

##### ^1^ Azienda Ospedaliero Universitaria di Pisa, Pisa, ITALY, ^2^ Ospedali Riuniti di Livorno, Livorno, Italy

###### **Correspondence:** M. Pillitteri

*Journal of Anesthesia, Analgesia and Critical Care 2023,* **3(Suppl 1):**A127

Background

Care for Jehovah's Witness (JW) patients can be challenging and often a dilemma to clinicians because of the patient's religious beliefs against receiving blood products. We present a severe trauma brain injury (TBI) needing emergent neurosurgery, in which, despite severe anemia, an intensive approach with multimodal neurological and hemodynamic monitoring contributed to achieve a good neurological outcome at discharge.

Case Report

A 40 years old JW woman was admitted for severe TBI consisting in acute subdural hematoma (image 1), with calculated GCS of 5 on the scene, needing intubation and emergency surgery (evacuation of hematoma and decompressive craniotomy). Her medical history was negligible except for chronic anemia.

After surgery hemoglobin level decreased to 3,5g/dl determining unstable hemodynamic and increasing lactates. A blood sparing protocol was compulsory, avoiding routine samples except for a daily blood gas-analysis. Hemodynamic was maintained with infusion of amines and low volume cristalloyds, as an attempt of hemoconcentration, with the aim of obtaining a cerebral perfusion pressure of 60mmhg and minimizing the reduction of cerebral oximetry (rSO2)(1). We strictly followed THE MANTLE bundle(2) using multimodal neuromonitoring (qEEG, rSO2, transcranial doppler ultrasound, pupillometry, invasive intracranial pressure) and appling targeted temperature management with mild hypothermia (T 35,5°) associated with high level of sedation. (table 1)

Multidisciplinary hematologist’s support helped us to individualize the therapy to enhance fast autologous red blood cells production (3), consisting in early administration of high dose of intravenous iron (62,5mg/day), plus folic acid 5mg/day, cyanocobalamin 5000mcg/ml weekly and erythropoietin 40000Ul/ml weekly.

Late tracheostomy ultrasound and bronchoscopy guided was performed to reduce risk of bleeding. Weaning started after obtaining a safe hemoglobin level of 7g/dl and absence of sign of hypoperfusion.

After twenty days the patient was weaned from ventilation with a good neurological recovery (GCS 13). Her last hemoglobin level before discharge to the rehabilitation unit was 12,6g/dl. At six months RCS-E scale was 18, LCS scale was 7.

Discussion

JW patients with severe life threatening anemia present an interesting challenge in critical care. Cerebral tissue hypoxia is not uncommon in severe TBI.

Clinical reasoning driven by multimodal neuromonitoring could help with personalized treatment, in order to optimize the match between oxygen delivery and consumption and improve neurological outcome.

This case can be a model for the future to promote the blood sparing approach in the management of severe TBI.

Informed consent to publish the case report had been obtained.

References

A) Leal-Noval SR et al. Red Blood Cell Transfusion Guided by Near Infrared Spectroscopy in Neurocritically Ill Patients with Moderate or Severe Anemia: A Randomized, Controlled Trial. J Neurotrauma. 2017 Sep;34(17):2553-2559. doi: 10.1089/neu.2016.4794.
B) Godoy, D.A., Murillo-Cabezas, F., Suarez, J.I. et al. “THE MANTLE” bundle for minimizing cerebral hypoxia in severe traumatic brain injury. Crit Care 27, 13 (2023). https://doi.org/10.1186/s13054-022-04242-3
C) Posluszny JA Jr, Napolitano LM. How do we treat life-threatening anemia in a Jehovah's Witness patient? Transfusion. 2014 Dec;54(12):3026-34. doi: 10.1111/trf.12888.

**Table 1 (abstract A127).** Multimodal monitoring data (day of arrival and third day)

	Day of arrival	Third day
**ICP**	2 mmHg	3 mmHg
**Pupillometry**	right NPi 1,1	right NPi 0
	left NPi 3,9	left NPi 3,8
**Ocular Echography**	right 5,5mm	not performed
	left 6,5mm	
**rSO2**	right 58%	right 60%
	left 60%	left 62%
**Blood samples**	Hb 3,5 g/dl	Hb 4,5 g/dl
	Lactates 11,5	Lactates 1,8

**Image 1 (abstract A127).**
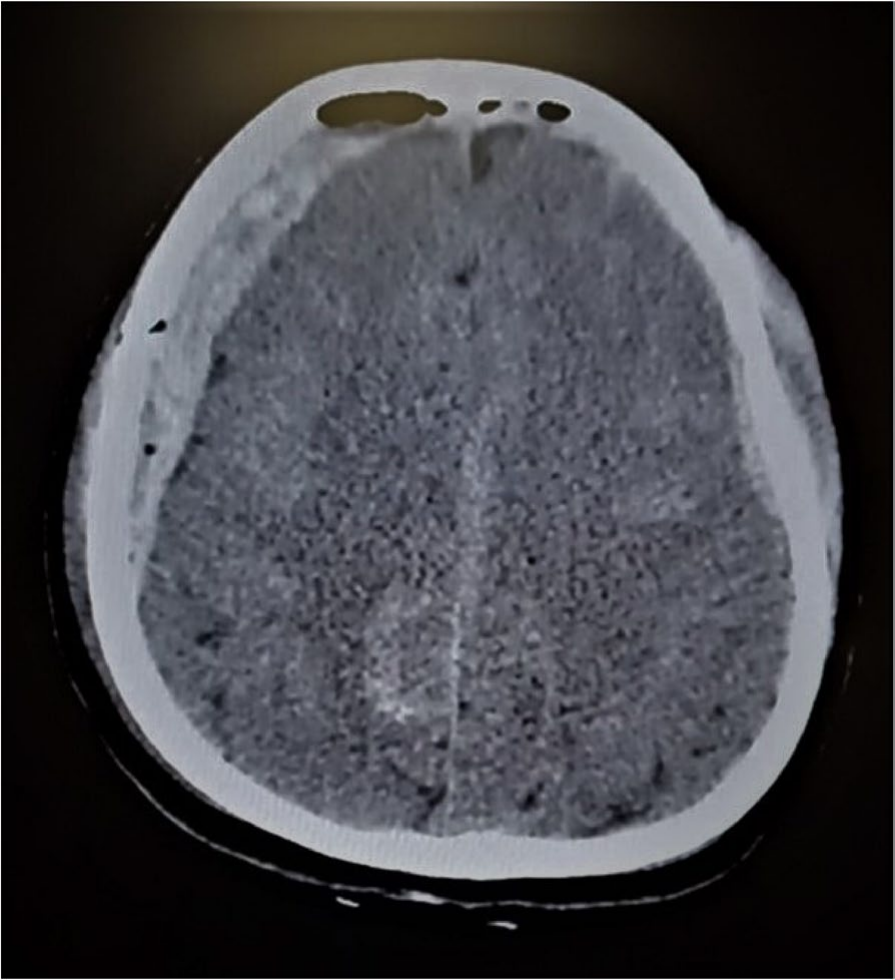
First CT scan performed before treatment

### A128 Preliminary data from Timing of invasive intracranial pressure monitoring between neurosurgeons and intensive care physicians (TIMING-ICP)

#### L. Mariani ^1^, D. Filippi ^4^, L. De Maria ^1,3^, F.A. Rasulo ^1,2^

##### ^1^ Department of Medical and Surgical Specialties, Radiological Sciences and Public Health, University of Brescia, Brescia, Italy; ^2^ Division of Anesthesiology, Intensive Care and Emergency Medicine, University of Brescia at Spedali Civili Hospital, Brescia, Italy; ^3^ University of Brescia Residency School in Neurosurgery, Brescia, Italy; ^4^ Division of Anesthesiology and Critical Care Medicine, Papa Giovanni XXIII Hospital, Bergamo, Italy

###### **Correspondence:** L. Mariani

*Journal of Anesthesia, Analgesia and Critical Care 2023,* **3(Suppl 1):**A128

Background

Intracranial hypertension (IH) has been consistently associated with poor neurological outcome. Many studies show that this is not only related to intracranial pressure (ICP) intensity, but also to the duration of IH episodes [1]. In this setting, promptly detection of IH by invasive monitoring seems to be fundamental to reduce the “dose” of intracranial pressure. Placement of intraparenchymal catheters (IPC), which are associated with an accuracy comparable to the measurements obtained from the gold standard technique, external ventricular drains (EVD), have usually been performed by neurosurgeons. However, placement of IPCs by trained intensivists at the bedside is becoming more common [2]. Preliminary retrospective data suggest that IPC placement performed by the intensivists is a safe procedure, which has the potential of being carried out faster than when it is performed by neurosurgeons, with a similar incidence of complications. The aim of this observational, prospective and multicentric study is to compare timing of invasive intracranial pressure monitoring performed by neurosurgeons and intensivist and to detect differences in the incidence of complications.

Materials and Methods

All adults with acute brain injury with urgent indication for invasive ICP monitoring are being included in 7 different centers in Italy. Exclusion criteria are represented by significative coagulation disorders, non urgent request and the necessity to insert EVD for reasons other than urgent ICP monitoring. Timing of ICP monitoring will be analyzed as follows: T1 represents time when indication to invasive ICP monitoring is stated and T2 is the time in which skin incision is performed. We’ll also consider the place in which the maneuver is carried out (Intensive Care Unit-ICU or Operatory room-OR). Incidence of complications and outcome parameters will also be noted. Written informed consent will be required from all surviving patients as soon as they’ll regain their mental competency.

Results

We are still enrolling patients in both arms. Interim analysis on March 2023 was performed on 40 patients which showed a mean T2-T1 time of 68 minutes in 15 patients treated by intensivists in ICU, 122 minutes in 16 patients treated by neurosurgeons in ICU and 198 minutes in 9 patients treated by neurosurgeons in OR. Complete statistical data analysis requires 32 patients in both arms and will be performed with a linear mixed-effect model to investigate the effect of the operator on timing differences. The incidence of complications will be analyzed through a Generalized Linear Mixed Model.

Conclusions

TIMING-ICP may allow us to assess if ICP monitoring placement by intensivists can be a time-saving procedure compared to when requiring a neurosurgeon for placement, and if it is equally safe to do so.

References

1. Güiza, F. et al. Visualizing the pressure and time burden of intracranial hypertension in adult and
2. paediatric traumatic brain injury. Intensive Care Med. 41, 1067–1076 (2015)
3. Latronico, N. et al. Bedside burr hole for intracranial pressure monitoring performed by anaesthetist-intensive care physicians: Extending the practice to the entire ICU team. Minerva Anestesiol. 69, 159–168 (2003).

### A129 Dexmedetomidine based sedation optimizes conditions for brain mapping in awake craniotomy

#### B. Giammarioli ^1^, G. Hare ^1^, P. Acharya ^1^, S. Das ^2^, M. Cusimano ^2^, K. Ma ^1^, A. Rigamonti ^1^

##### ^1^ Department of Anesthesiology and Pain Medicine, University of Toronto, Toronto, Canada; ^2^ Division of Neurosurgery, Department of Surgery, University of Toronto, Toronto, Canada

###### **Correspondence:** B. Giammarioli

*Journal of Anesthesia, Analgesia and Critical Care 2023,* **3(Suppl 1):**A129

Introduction

Awake craniotomy has been established for years as technique to facilitate intraoperative electrocorticography and cortical mapping to accurately identify those areas of brain which control motor functions and speech to achieve maximal tumor excision. Many different anesthetic care protocols have been described; however, there is still no consensus as to the best anesthetic technique. We have recently developed an approach for awake craniotomy utilizing dexmedetomidine as primary anesthetic agent. However, concerns about the use of dexmedetomidine include the risk of bradycardia and hypotension and the need to support hemodynamics. We report here a case series of 75 patients undergoing awake craniotomy with dexmedetomidine as the primary anesthetic agent. We hypothesize dexmedetomidine can be utilized safely as an anesthetic for awake craniotomy without the risk of airway manipulation and without hemodynamically significant hypotension or bradycardia.

Methods

With research ethics approval, the charts of 75 patients who underwent awake craniotomy

between 2012 and 2023 were reviewed by two authors (GB, GH) 1,2. In the current study, we reported the amount of dexmedetomidine used and the maximum and minimum systolic and diastolic blood pressures and HR throughout the duration of the procedure. We assessed the need for airway manipulation (defined by the use of a laryngeal mask or endotracheal tube during the procedure) and evidence of hypotension (greater than 20%sustained reduction in SBP) and bradycardia (requiring treatment with muscarinic antagonist) during the procedure. We also reported the use of vasopressors (ephedrine, phenylephrine) and glycopyrrolate/atropine. Data are presented as mean and standard deviation.

Results

Patients were 38% female, mean age 46 ± 16; and BMI 27.3 ± 5.5. Length of surgery was 280 ±

73 minutes. No patients required airway intervention. Dexmedetomidine dose was 167±84 mcg and 2 ±0.92 mcg/kg. Maximal and minimal systolic and diastolic blood pressures and HR were 152 ± 19 and 107 ± 14 mmHg; 84± 9 and 56 ± 5 mmHg; 78 ± 14 and 54 ± 10 BPM, respectively. Fig.1

Dexmedetomidine was the primary anesthetic in all cases. Five patients (7%) received ephedrine (10 mg); 3 patients (4%) received phenylephrine, infusions (20 mcg/minute; 30 or 120 minutes). Two patients (3%) received glycopyrrolate (0.2 mg).

Discussion

We have completed a series of 75 patients utilizing dexmedetomidine with favorable outcomes with no need for airway instrumentation. Optimal conditions for brain mapping were obtained in all the cases. We observed no incidence of clinically significant hypotension or bradycardia and use of vasopressors/glycopyrrolate was minimal. We propose that the surgical stimulations and conditions associated with awake craniotomy allow for the use of dexmedetomidine as the primary anesthetic without evidence of clinically significant hypotension or bradycardia.

References

1. McAuliffe N, Nicholson S, Rigamonti A, Hare GMT, Cusimano M, Garavaglia M, Pshonyak I, Das S. Awake craniotomy using dexmedetomidine and scalp blocks: a retrospective cohort study. Can J Anaesth. 2018 Oct;65(10):1129-1137
2. Garavaglia MM, Das S, Cusimano MD, Crescini C, Mazer CD, Hare GMT, Rigamonti A. Anesthetic approach to high-risk patients and prolonged awake craniotomy using dexmedetomidine and scalp block. J Neurosurg Anesthesiol. 2014 Jul;26(3):226-33

**Fig. 1 (abstract A129).**
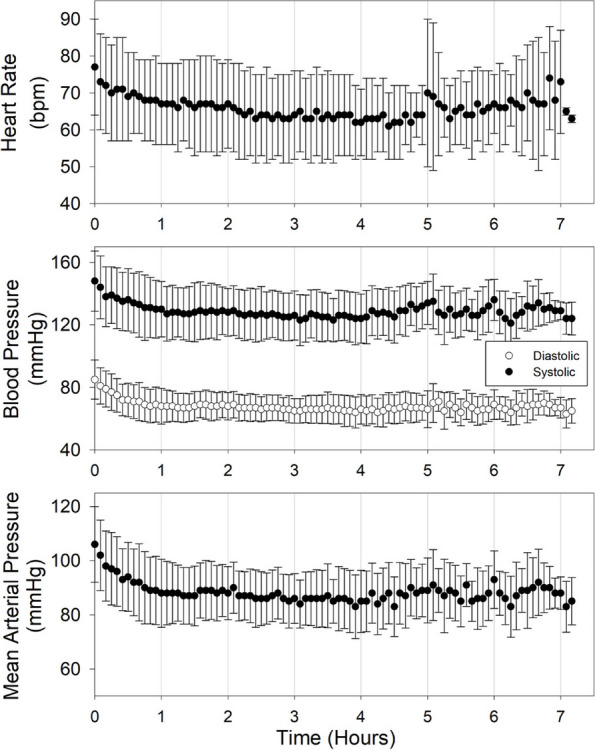
Heart Rate and Blood Pressure Measurements During Awake Craniotomy

### A130 Measuring optic nerve sheath diameter for the rapid detection of severe intracranial hypertension

#### D. Fiume ^1, 2, 3^, A. Armignacco ^2^, S. Carlini ^2^, L. Coen Tirelli ^2^, A. Tiberi ^2^, F. Marchetti ^2^, L. Di Marzio ^2^, B. Baldelli ^2^, P. Picerno ^2^, M. Arciuolo ^1^, M. Peverini ^1^, M. Galletti ^1^

##### ^1^ Sant'Eugenio Hospital, Rome, Italy; ^2^ Tor Vergata University, Rome, ITALY; ^3^ UniCamillus University, Rome, Italy

###### **Correspondence:** A. Armignacco

*Journal of Anesthesia, Analgesia and Critical Care 2023,* **3(Suppl 1):**A130

Background

Intracranial hypertension (IH) is a frequent complication of brain injury, and its severity was correlated with worst outcome. Optic nerve sheath diameter (ONSD) is a non-invasive, cheap, fast and operator-dependent bedside test.

Materials and methods

From May 2022 to March 2023, eighteen adult patients (10M/8F) with traumatic brain injury, subarachnoid/intracerebral haemorrhage and ischaemic stroke were observed in our Post Operative Intensive Care Unit. Clinical management was driven by international guidelines. In these patients clinical monitoring, computed tomography (CT), intraventricular catheter for intracranial pressure (ICP), transcranial doppler (TCD) and ONSD monitoring were all available. Ultrasound examination was performed a 7.5 MHz linear ultrasound probe (ONSD) and traditional 2-MHz transducer (TCD).

Results

In thirteen patients multimodal monitoring has progressed in line with international literature. However, in five patients with ICP > 24 mmHg, IH suspected clinically and CT confirmation, ONSD was constantly >60mm (60-71).

Conclusions

We know that ONSD is sensible but not specific test for IH, and it may help to detect ICP in a multimodal approach [1-2]. We have a clear idea that of the studied ultrasound non-invasive methods, ONSD is the best estimator of ICP [3-4]. Maybe the anatomical features of the optic nerve allow an enlargement of the diameter especially in the case of severe IH. Larger studies are required, but ONSD would be a very useful tool in case of lack of CT (technical problems, overcrowding, ambulance setting...) or while awaiting execution of the same exam.

References

1. Ohle R, McIsaac SM, Woo MY, Perry JJ. Sonography of the Optic Nerve Sheath Diameter for Detection of Raised Intracranial Pressure Compared to Computed Tomography: A Systematic Review and Meta-analysis. J Ultrasound Med. 2015; 34(7):1285-94
2. Fernando SM, Tran A, Cheng W, Rochwerg B, Taljaard M, Kyeremanteng K, English SW, Sekhon MS, Griesdale DEG, Dowlatshahi D, McCredie VA, Wijdicks EFM, Almenawer SA, Inaba K, Rajajee V, Perry JJ. Diagnosis of elevated intracranial pressure in critically ill adults: systematic review and meta-analysis. BMJ 2019; 366:l4225
3. Cardim D, Robba C, Donnelly J, Bohdanowicz M, Schmidt B, Damian M, Varsos GV, Liu X, Cabeleira M, Frigieri G, Cabella B, Smielewski P, Mascarenhas S, Czosnyka M. Prospective Study on Noninvasive Assessment of Intracranial Pressure in Traumatic Brain-Injured Patients: Comparison of Four Methods. J Neurotrauma. 2016; 33(8): 792–802
4. Robba C, Cardim D, Tajsic T, Pietersen J, Bulman M, Donnelly J, Lavinio A, Gupta A, Menon DK, Hutchinson PJA, Czosnyka M. Ultrasound non-invasive measurement of intracranial pressure in neurointensive care: A prospective observational study. PLoS Med. 2017; 14(7): e1002356

### A131 Mean platelet volume to platelet count ratio could be a valid predictor of mortality in critically ill brain injured patients: preliminary data

#### F. Di Pierro, T.G. Zimotti, P.S. Mariotti, G. Cinnella, A. Cotoia

##### Università degli Studi di Foggia, Foggia, Italy

###### **Correspondence:** F. Di Pierro

*Journal of Anesthesia, Analgesia and Critical Care 2023,* **3(Suppl 1):**A131

Background

In various situations, such as Non-ST-Elevation Myocardial Infarction or Severe Sepsis, the ratio between mean platelet volume (MPV) and platelet count (PLT) has shown its prognostic role, way more than MPV and PLT alone.

Purpose

In this study MPV/PLT ratio was calculated to assess its prognostic role in predicting in hospital mortality in intensive care unit (ICU) brain injured patients.

Methods

From May 2021 to May 2023 clinical history of all brain injured patients admitted to the ICU was recorded and hemogram at admission was performed to later calculate the MPV/PLT ratio. Study population was divided in two groups: Group 0 - survivors; Group 1 - not survivors.

Results

67 patients were enrolled: mean age was 61.7±17.4 years, 23 patients (34.3%) were female. Diagnosis at admission was: traumatic brain injury 23.9%, intracranial hemorrhage 37.3%, subarachnoid hemorrhage 14.9%, epidural hematoma 1.5%, subdural hematoma 13.4%, acute ischaemic stroke 9%. 40 patients died during the hospitalization in the ICU (59.7%). Mean MPV/PLT at admission was significantly higher in survivors 0.504 ± 0.317 than in not survivors 0.276 ± 0.281 (p=0.003). Mean MPV at admission was 50.9 ± 3.6 fl in survivors and 72.6 ± 31.4 fl in not survivors (p=0.016), while mean PLT at admission was 217 ± 76.7 10^3/ul in discharged patients and 171 ± 58.5 10^3/ul in patients who died during hospitalization in ICU (p=0.011).

Conclusions

MPV/PLT could be considered an independent predictor of ICU brain injured patients’ mortality, much more reliable than MPV and PLT alone. During ICU stay, hemogram at admission should be performed and MPV/PLT ratio should be calculated from it to have an idea of the brain injured patient's outcome.

Informed consent was obtained from all the enrolled patients.

### A132 Isoflurane and Cerebral Blood Flow in Subarachnoid Hemorrhage

#### I. Depetris ^1^, E. Balzani ^2^, M. Cedrone ^2^, F. Denegri ^3^, C. Geninatti ^4^, O. Morrone ^1^, M. Berardino ^1^

##### ^1^ Department of Anesthesiology, Intensive Care and Emergency, Trauma Center, AOU Città della Salute e della Scienza, Turin, Italy; ^2^ Department of Surgical Sciences, University of Turin, Turin, Italy; ^3^ Neuroradiology Unit, Trauma Center, AOU Città della Salute e della Scienza di Torino, Turin, Italy; ^4^ Radiology department, AOU Città della Salute e della Scienza di Torino, Turin, Italy

###### **Correspondence:** I. Depetris

*Journal of Anesthesia, Analgesia and Critical Care 2023,* **3(Suppl 1):**A132

Background

Inhalation sedation could enhance cerebral perfusion in patients with subarachnoid hemorrhage (SAH). However, concerns regarding the elevation of Intracranial Pressure (ICP) and diversion of blood flow need to be addressed. [1,2] For patients with high-grade SAH requiring sedation, following standard Intensive Care Unit monitoring and treatment, a CT perfusion scan must be conducted starting from day three to detect impending vasospasm.

Materials and methods

Eleven patients with SAH and a World Federation of Neurosurgical Societies (WFNS) score ranging from 3 to 5, along with ICP monitoring, were enrolled in the study. After the CT perfusion scan planned for day three, an isoflurane concentration equivalent to an End Tidal Fraction (Fet%) of 1.1-1.2 was administered using the Mirus System. Once the targeted Fet% was achieved, a second CT perfusion scan was performed. Throughout the administration of the drug, Cerebral Perfusion Pressure (CPP), End Tidal CO2 (EtCO2), ICP, arterial pressure, and oxygen saturation were recorded every five minutes on a Case Report Form (CRF) until the target concentration was reached. Intravenous sedation, catecholamine infusion and respiratory rate were adjusted based on CPP and EtCO2. Approvals from local ethics committee and AIFA were obtained. Informed consent was obtained according to local ethics committee’s indications.

Post-procedural evaluation of CT perfusion was conducted using the GE Healthcare Advantage Workstation. A total of 18 Regions of Interest (ROI) from the frontal, temporal, and occipital-parietal areas were selected for the pre- and post-inhalation study. Cerebral blood flow (CBF), Cerebral Blood Volume (CBV), and Mean Transit Time (MTT) were analyzed. A repeated measures ANOVA was performed, with the inhalation phase and selected cerebral areas as factors. To assess the correlation between flow and volume, a linear regression was conducted.

Results

A repeated measures analysis was performed for descriptive parameters, indicating a statistical significance only for Fet.gas (p<0.1) (Table 1).

A significant difference was found for CBF values (p<0.001). Additionally, the stratified analysis for cerebral areas did not show any differences for the three variables (Figure 1).

The linear regression revealed a significant correlation between volume and flow (p<0.001) with a moderate correlation (Pearson 0.44).

Conclusions

The administration of isoflurane was not associated with increased ICP. Overall, we observed an 8% increase in CBF without any occurrence of stealing in the selected ROIs. The primary goal of the study was to ensure the safety of drug administration, making it reasonable to consider this as a viable treatment option.

**Table 1 (abstract A132).** Repeated measures analysis for descriptive parameters: MAP (Mean Arterial Pressure), HR (Heart Rate) SpO2 (Oxygen Saturation), EtCO2 (End Tidal CO2), ICP (Intracranial Pressure) Fet.gas (End Tidal Fraction of gas)

	Timing 1 (N=11)	Timing 2 (N=11)	Timing 3 (N=11)	p
**MAP,** ***median (IQR)***	89.0 (82.50-92.36)	84.00 (77.50-91.00)	79.00 (75.00-88.50)	0.237
**HR,** ***median (IQR)***	83.00 (69.00-89.00)	77.00 (70.50-90.00)	78.00 (70.00-91.00)	0.997
**SpO2,** ***median (IQR)***	98.00 (97.00-99.00)	98.00 (97.00-99.00)	98.00 (97.00-99.00)	0.764
**EtCO2,** ***median (IQR)***	38.00 (34.50-41.00)	38.00 (35.00-41.00)	37.00 (34.50-39.50)	0.765
**ICP,** ***median (IQR)***	22.00 (3.00-27.00)	22.00 (3.50-31.50)	21.00 (3.00-31.50)	0.970
**Fet.gas,** ***median (IQR)***	0.80 (0.75-0.80) *	0.9 (0.8-0.9)	1.10 (0.95-1.15)	<0.01

**Fig. 1 (abstract A132).**
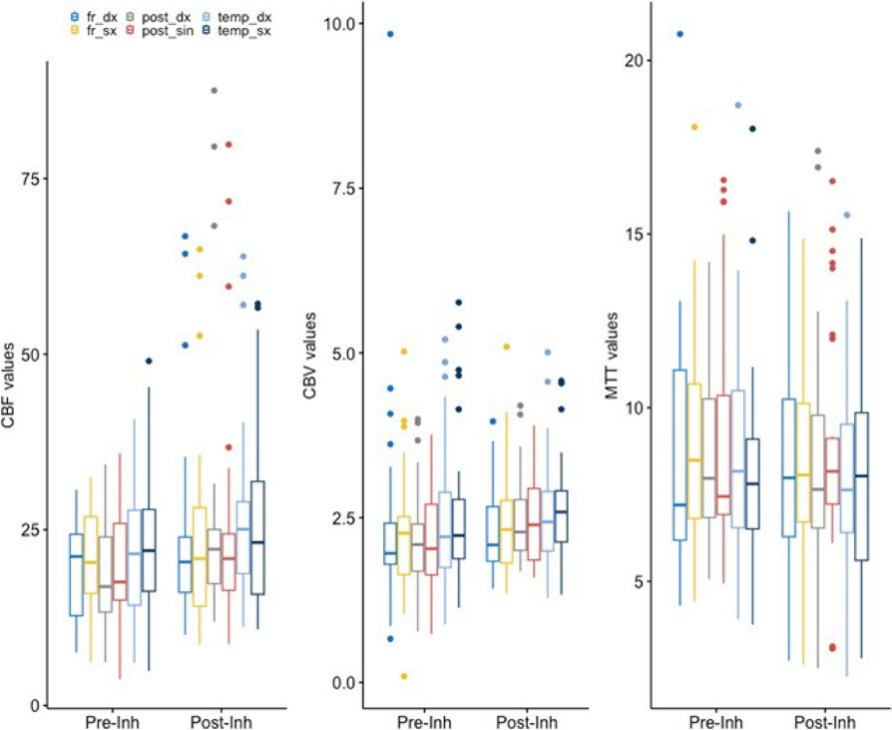
Values of cerebral blood flow (CBF), cerebral blood volume (CBV), and mean transit time (MTT) before and after isoflurane inhalation

### A133 Role of a double lumen cerebrospinal fluid shunt with washing system in treatment of pyogenic meningoventriculitis: a case report

#### E.M. Catanese, T. Tassinati, G. Dallocchio, A.L. PInamonti, M. Vason

##### Azienda Ospedaliera-Universitaria di Ferrara, Ferrara, Italy

###### **Correspondence:** E.M. Catanese

*Journal of Anesthesia, Analgesia and Critical Care 2023,* **3(Suppl 1):**A133

Background

Pyogenic ventriculitis is a severe infection of the central nervous system with serious and often irreversible consequences in the quality of life of patients. Its treatment is difficult due to the impossibility of achieving sterility of cerebrospinal fluid and the physiological characteristics promptly[1], and its mortality rates is close to 60%[2].

Meningitis and ventriculitis may be a complication of a variety of invasive procedures including

spinal surgery[3]. Incidental durotomy when unrepaired increases the risk of developing meningitis[4].

Factors relevant to the nature of CNS infection pose significant challenges to clinicians and ineffective treatments are frequent[5]. Intraventricular administration of antibiotics may offer several benefits over systemic therapy; however, the outcomes and current practices of such treatments are poorly described in literature[6].

Case report

A 72-yr-old male underwent lumbar spine stabilization surgery in a peripheral hospital. Eight days later, he started showing hyperpyrexia and drowsiness, rapidly evolving towards a state of coma, so he has been transferred to our hospital. Exams showed meningitis from Klebsiella pneumoniae with concomitant lumbar paravertebral abscess, for which the patient was initially treated with aspiration and systemic targeted antibiotic therapy, with no significant improvement. A subsequent MRI showed a meningoencephalitis and ventriculitis with initial obstructive hydrocephalus, therefore in fifteenth day a two-lumen ventricular derivation system was placed, in order to manage continuous washing and intraventricular instillation of antibiotics. A few days later the patient underwent neurosurgical surgery to remove vertebral synthesis devices: at that moment surgeons discovered the presence of dural fistula, treated with autologous fat patches. The ventricular washing system was kept in place for 14 days, and we assisted to a progressive until complete cleansing of the purulent material on CT with a synchronous clinical neurological improvement. Continuous monitoring of the ICP has also allowed to observe a progressive lowering of intracranial pressure, spy of a deliquoration. The patient underwent a surgical revision of the fistula and on the eighteenth day all antibiotics infusions were suspended for definitive resolution of the meningoventriculitis.

Conclusions

Combining the ability to directly modify the concentration of the targeted antibiotics in the cerebrospinal fluid while simultaneously removing bacterial mass, the use of a double lumen shunt seems to be a rational choice for patients who poorly respond to systemic antimicrobial therapy alone.

Written informed consent for publication was obtained.

References

1. Ochoa,A.,Argañaraz, R. & Mantese,B. Neuroendoscopic lavage for the treatment of pyogenic ventriculitis in children: personal series and review of the literature. Childs NervSyst 38,597-604(2022)
2. Rezai Jahromi,Behnam MD;Tanskanen. Active Cerebrospinal Fluid Exchange System for Treatment of Pyogenic Ventriculitis. Neurosurgery Open 2(4):okab030
3. Zhou,Jiaming MDa,b; Wang,Rui MDa,b. Incidence of Surgical Site Infection After Spine Surgery:A Systematic Review and Meta-analysis. SPINE 45(3):p208-216
4. Hassanzadeh H,Bell J,Bhatia M. Incidental Durotomy in Lumbar Spine Surgery;Risk Factors, Complications, and Perioperative Management. J Am Acad Orthop Surg.2021 Mar 15;29(6):e279-e286
5. Ippolito M,Giarratano A,Cortegiani A. Healthcare-associated central nervous system infections. Curr Opin Anaesthesiol.2022 Oct 1;35(5):549-554
6. Tunkel AR,Hasbun R,Bhimraj A, et al.2017 Infectious Diseases Society of America's Clinical Practice Guidelines for Healthcare-Associated Ventriculitis and Meningitis. Clin Infect Dis.2017;64(6):e34-e65

### A134 Fast Track in elective neurosurgery: the role of low propofol–high remifentanil BIS-guided general anaesthesia

#### C. Carozzi ^1^, F. Bulica ^2^, M. Manzalini ^3^, D. Martino ^1^, M. Introna ^1^, M. Gemma ^1^

##### ^1^ IRCCS Foundation Carlo Besta Neurological Institute Besta, Milano, Italy; ^2^ University of Milan, Milano, Italy; ^3^ University of Insubria, Varese, Italy

###### **Correspondence:** C. carozzi

*Journal of Anesthesia, Analgesia and Critical Care 2023,* **3(Suppl 1):**A134

Background

An early, reliable and safe neurological examination is crucial in Fast Track program for neurosurgical patients [1].

While processed EEG monitoring avoids oversedation during general anaesthesia (GA), the optimal dosage of hypnotics and analgesics is not yet established [2]. This study was designed to evaluate the effect of propofol and remifentanil on quality of recovery (QR) after neurosurgery.

Materials and methods

After acquiring informed consent, we prospectively collected propofol and remifentanil doses administered by TCI (Target controlled infusion), respectively with Schnider and Minto model, in 50 elective neurosurgical adults, with Glasgow Coma Scale (GCS) 15, during routine GA titrated to BIS (Medtronic, Minneapolis, USA) values 40-60, keeping BP, temperature, pCO2, and SaO2 in normal ranges.

The Recovery Room nurses collected TCI data and scored the QR (Fig. 1). A score <13 defined suboptimal awakening (Aw-No), 13 defined optimal awakening (Aw-Yes).

Univariate variable as gender, age, BMI, duration of surgery, supra vs. infratentorial, intraoperative dosage of propofol (PD) and remifentanil, the effect site concentration of propofol and remifentanil (CeR) pre-extubation were studied in a logistic model, reporting ORs with 95% CIs and ROC curves. Statistical analysis was performed with R (version 4.2.3 https://www.R-project.org/)

Results

27 pts were in the Aw-YES and 23 in the Aw-No group. Only PD and CeR entered the multivariate logistic model for Aw-NO (ORs 1.14 (1.04-1.24) and 0.93 (0.89-0.97) respectively. The regression coefficients were 0.13 for PD and -0.07 for CeR, suggesting that PD increment and CeR decrement are associated with a probability of Aw-No. The best PD cut-off for Aw-No prediction was 4.7 mg/kg/h (Fig. 2).

PD was significantly higher in the Aw-No than in the Aw-Yes (median 4.89 vs 3.96 mg/kg/h; P=0.012), intraoperative Remifentanil dosage didn’t differ (Fig. 3). Pre-extubation CeR was lower in the Aw-No than in the Aw-Yes (media 3,67 vs 6,35 ng/ml; P=0.003) (Fig. 4). Time to extubation was longer in the Aw-No (media 26,4 vs 10,3 min; P=0.001) (Fig. 5).

Conclusions

Low doses of intraoperative propofol and high Ce pre-extubation remifentanil are associated with optimal, early and uneventful awakening after BIS-guided GA and allow a valuable triage in Fast Track Neurosurgery.

References

A) Badenes R, Prisco L, Maruenda A, Taccone FS. Criteria for Intensive Care admission and monitoring after elective craniotomy. Curr Opin Anaesthesiol. 2017 Oct;30(5):540-545.
B) Irwin MG, Chung CKE, Ip KY, Wiles MD. Influence of propofol-based total intravenous anaesthesia on peri-operative outcome measures: a narrative review. Anaesthesia. 2020 Jan;75 Suppl 1:e90-e100.

**Fig. 1 (abstract A134).**
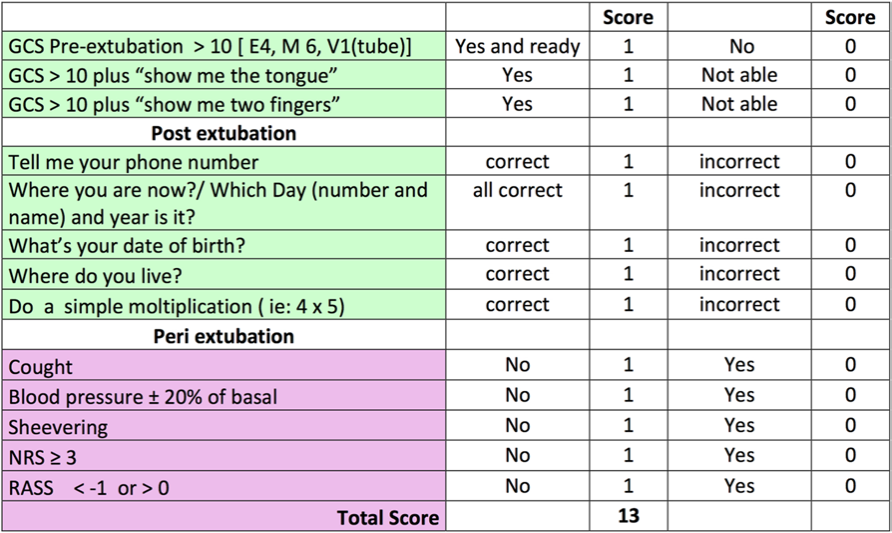
Criteria for Quality of Recovery: Score = 13 -> Awakening-YES (Aw-YES), Score < 13 -> Awakening-NO (Aw-NO)

**Fig. 2 (abstract A134).**
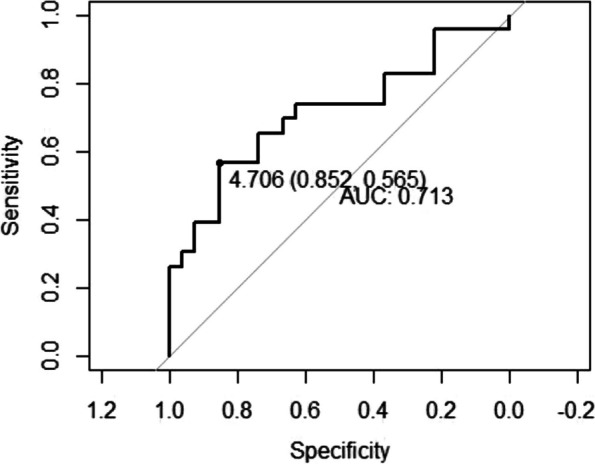
Optimal Intraoperative Propofol dosage for predicting Aw-NO

**Fig. 3 (abstract A134).**
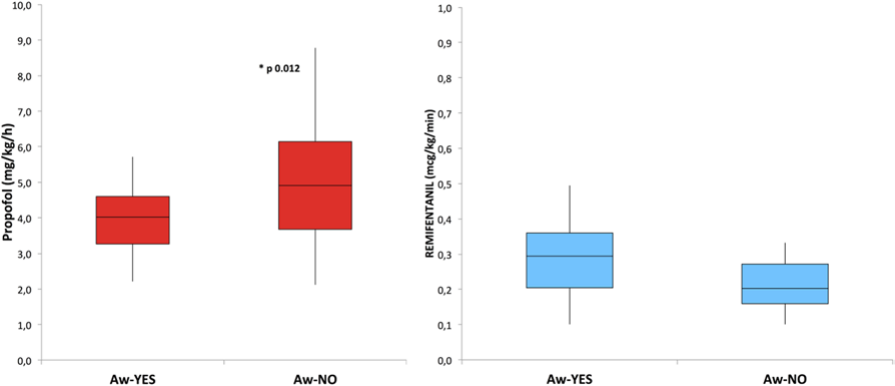
Intraoperative Propofol and Remifentanil dosages

**Fig. 4 (abstract A134).**
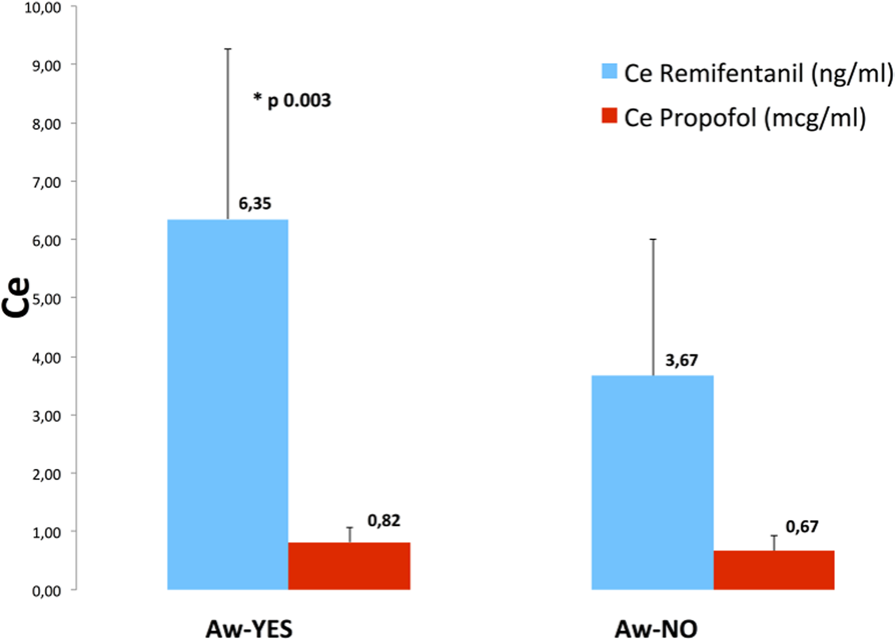
Pre-extubation Ce Remifentanil and Propofol

**Fig. 5 (abstract A134).**
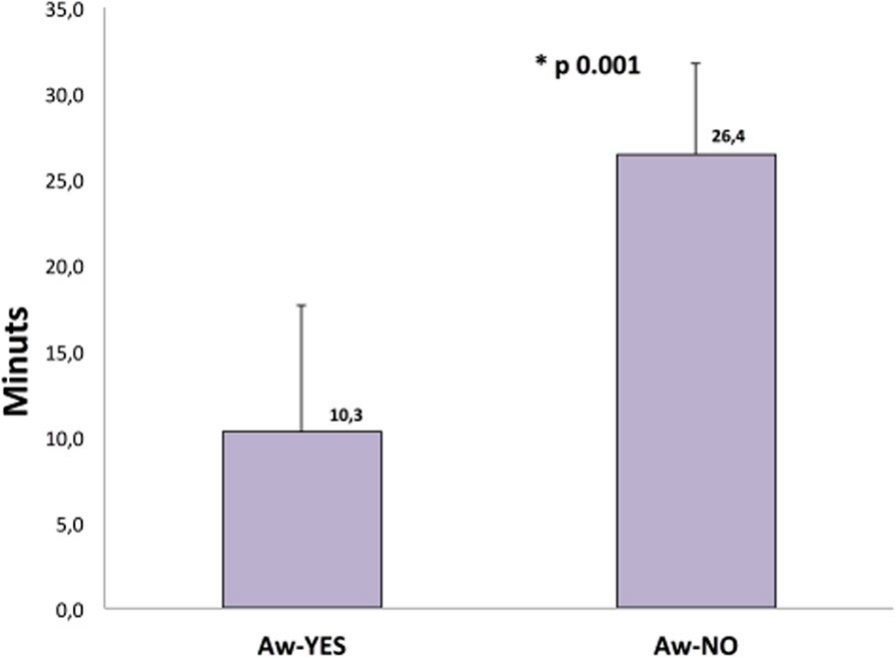
Time to Extubation

### A135 Covid-19 associated autoimmune encephalitis: a case report

#### D. Cappelletto, G. Gagliardi, C. Chiani, A. Boselli

##### Ospedale di Rovigo - ULSS 5 Polesana, Rovigo, Italy

###### **Correspondence:** D. Cappelletto

*Journal of Anesthesia, Analgesia and Critical Care 2023,* **3(Suppl 1):**A135

It is well known that SARS-CoV-2 infection can affect the central and peripheral nervous system with various manifestations: alterations in smell, muscle pain, confusion, delirium and dizziness. The pathological alterations underlying the symptoms can be Guillain-Barré syndrome, meningitis, encephalitis, Miller Fisher syndrome and stroke.

We describe the case of a 61-year-old male patient admitted to our intensive care unit due to a state of drowsiness, disorientation, slurred speech, EEG consistent with epilepsy and encephalitis and positive RT-PCR Covid-19 test. The patient was subsequently sedated and intubated. Cultural investigations were initiated on blood, urine and cerebrospinal fluid, and empirical therapy with dexamethasone, ceftriaxone and acyclovir was started. The epilepsy therapy was continued. During the hospitalization, the patient developed ophthalmoplegia and weakness, especially in the upper limbs. Since all the cultures yielded negative results, in suspicion of autoimmune encephalitis, treatment with intravenous immunoglobulins and low-dose methylprednisolone was initiated, considering the ongoing Sars-Cov2 infection. Therefore, the patient progressively showed gradual clinical improvement with resolution of ophthalmoplegia and recovery of strength, which allowed for extubation and the return to spontaneous breathing with high-flow nasal cannula.

In conclusion, in this case of autoimmune encephalitis associated with COVID-19, we have established an immunomodulatory treatment based on the use of intravenous immunoglobulins, avoiding high doses of corticosteroids that could have worsened the SARS-CoV-2 infection.

Il consenso informato è stato correttame ottenuto - informed consent has been correctly obtained

### A136 Prophylaxis and anti-epileptic therapy following traumatic brain injury: a retrospective observational analysis of a single center

#### R. Antolini ^1^, F. Santoni ^1^, A. Salvucci Salice ^1^, E. Vitali ^1^, C. Pacini ^1^, G. Perini ^1^, A. Donati ^1,2^, A. Carsetti ^1,2^

##### ^1^ Dipartimento di Scienze Biomediche e Sanità Pubblica, Università Politecnica delle Marche, Ancona, Italy; ^2^ Azienda Ospedaliero Universitaria delle Marche, Ancona, Italy

###### **Correspondence:** C. Pacini

*Journal of Anesthesia, Analgesia and Critical Care 2023,* **3(Suppl 1):**A136

Background

Traumatic brain injuries (TBI) are classified based on the initial Glasgow Coma Scale (GCS) score as severe (GCS 3 - 8), moderate (GCS 9-13), or mild (GCS 14-15). A common complication after TBI, especially in the severe ones, are seizures that can be classified as early if they occur within 7 days, or late if they occur after that period. Early seizures can lead to an increase in intracranial pressure with all the related consequences. To prevent early seizures, prophylaxis with antiepileptic drugs is recommended [1]. The aim of our study is to analyze how antiepileptic prophylaxis is managed in patients admitted to our intensive care unit for TBI.

Materials and Methods

We retrospectively analyzed 84 patients admitted to our intensive care unit from January 2022 to December 2022 with a diagnosis of TBI. For each patient, the following variables were investigated: gender, age, pre-intubation GCS, discharge outcome in terms of survival, whether they underwent emergency neurosurgical treatment, any prophylactic and therapeutic anti-epileptic treatment with the respective timing, and the presence of critical activity on EEG during hospitalization.

Results

Of the 84 patients included, the median age was 55 [32; 74] years, 61 (72.6%) were male, with a mean pre-intubation GCS of 9.39 (±4); 19 (22.6%) patients underwent neurosurgical intervention, and 13 (15.4%) died. Of the collected sample, 40 (47.6%) patients underwent antiepileptic prophylaxis with Levetiracetam 500mg x2/die and 24 (28.6%) had a first EEG negative, while 17 (20.3%) had EEG activity indicative of seizure, performed at day 3 [2; 4]. Furthermore, different mono- or poly-pharmacological therapies were analyzed following seizure activity. A second EEG was performed at day 5 [4; 8] (Table 1).

In patients undergoing antiepileptic prophylaxis who had an EEG indicative of critical activity and whose Levetiracetam dosage was subsequently increased, there was a decrease in seizure activity on a second EEG in 3 cases out of 10 (7 patients with critical activity on the second EEG vs 3 without critical activity on the second EEG, p-value = 0.01).

Conclusion

From our preliminary results, despite the small sample size, it appears that increasing the dosage of Levetiracetam in response to a positive EEG is not sufficient to achieve remission of the epileptic activity in a subsequent EEG.

Reference

1. Carney N, Totten AM, O’Reilly C, Ullman JS, Hawryluk GWJ, Bell MJ, et al. Guidelines for the Management of Severe Traumatic Brain Injury, Fourth Edition. Neurosurgery. 2017;80(1):6–15.

**Table 1 (abstract A136).** Demographic and clinical characteristics of patients

		%
Total	84	
Age Median [rangeIQ]	55 [32; 74]	
Sex		
M	61	72,6
F	23	27,3
Pre-intubation GCS Mean (SD)	9.39 (3.93)	
Treatment		
Surgery	19	22,6
Conservative	65	77,3
Outcome		
ICU discharged	71	84,5
ICU dead	13	15,4
Post Traumatic Seizure Prophylaxis	40	47,6
No Post Traumatic Seizure Prophylaxis	44	52,3
First EEG positive	17	20,2
First EEG negative	24	28,5
No EEG	43	51,1
Timing First EEG Median [rangeIQ]	3 [2; 4]	
Timing Second EEG Median [rangeIQ]	5 [4; 8]	

## New technology for point-of-care diagnostics

### **A137 The predictive power of urinary biomarkers [TIMP-2] X [IGFBP7] in the identification of acute kidney injury in patients undergoing major abdominal surgery**

#### V. Tabolli, B. Mura, E. Terreni, L. Turi, F. Magiotti, L.L. Riccitelli, F. Firenzuoli, S. Cipolla, D. Giammarino, G. Villa, S. Romagnoli

##### Department of Health Science, Section of Anaesthesiology and Intensive Care, University of Florence, Florence, Italy

###### **Correspondence:** V. Tabolli

*Journal of Anesthesia, Analgesia and Critical Care 2023,* **3(Suppl 1):**A137

Background

Acute Kidney Injury (AKI) is one of the most common complications after major surgery with severe outcomes in terms of Chronic Kidney Disease (CKD) development, morbidity and mortality [1]. Furthermore, it has a significant impact on Intensive Care Unit (ICU) length of stay [1]. The Kidney Disease Improving Global Outcomes (KDIGO) guidelines recommend early recognition of patients at risk for AKI and their prompt management through supportive measures [2]. Only a minority of patients at high risk are treated according to KDIGO bundles. The identification of patients at higher risk of developing AKI, by testing a urine sample for biomarkers [i.e. Nephrocheck®: Tissue Inhibitor Metalloproteinase-2 (TIMP-2) and Insulin like Growth Factor Binding Protein 7 (IGFBP7)], allows to apply the preventing measures indicated by the KDIGO bundles to reduce the development of AKI [3]. The aim of the study was to assess the early predictive power of Nephrocheck® compared to conventional diagnostic tests for AKI [3].

Materials and methods

The monocentric retrospective observational clinical study was conducted at the Careggi Hospital University of Florence, Italy. The study included adults admitted to ICU after major abdominal surgery, with high risk for AKI development but with no other pre-existing kidney diseases. Written informed consent was previously obtained by all participants recruited. Urine samples were measured by Astute140 Meter Biomerieux analyser® using Nephrocheck® test, which converted [TIMP-2] x [IGFBP7] concentrations into a numerical result, called AKI Risk, at the admission to ICU. The evaluated parameters were preoperative and postoperative serum creatinine, Nephrocheck® values and presence or absence of a decreased urinary output (UO) in the first 6-12 hours after surgery (< 0.5 mL/kg/h) according to KDIGO guidelines. Statistical analysis was performed using Fisher’s exact test.

Results

Eighteen patients were enrolled: 10 patients with positive AKI Risk (0.3 or > 0.3) (Group A) and 8 with negative AKI Risk (< 0.3) (Group B), all patients resulted similar in terms of age and comorbidities. Group A was compared with group B, focusing on the incidence of postoperative AKI, based on a decrease of UO. Ninety percent of those patients belonging to group A vs. fifty percent of group B patients developed postoperative AKI, as shown in Table 1.

Fisher’s exact test returned a p-value of 0.1176 (Table 2).

Conclusions

Nephrocheck® test may be a valid predictor of AKI in patients undergoing major abdominal surgery. The study was not statistically significant, probably because of the small sample.

References

1. Ronco C, Bellomo R, Kellum JA. Acute kidney injury. Lancet. 2019 Nov 23;394(10212):1949-1964.
2. KDIGO AKI Work Group (2012). KDIGO clinical practice guideline for acute kidney injury. Kidney Int Suppl 2(1):1–138.
3. Koyner JL, Shaw AD, Chawla LS, Hoste EA, Bihorac A, Kashani K, Haase M, Shi J, Kellum JA; Sapphire Investigators. Tissue Inhibitor Metalloproteinase-2 (TIMP-2)xIGF-Binding Protein-7 (IGFBP7) Levels Are Associated with Adverse Long-Term Outcomes in Patients with AKI. J Am Soc Nephrol. 2015 Jul;26(7):1747-54.

**Table 1 (abstract A137).** Database with evaluated parameters

	Patients ID	Serum creatinine		Nephrocheck® values at admission to ICU	Decrease in urinary output in the first 6-12h after surgery (yes/no)
		(g/dL)			
		Preoperative	Postoperative		
Group A	1	1.2	1.28	0.35	yes
	2	1.77	1.41	0.36	yes
	3	1.21	1.23	1.02	yes
	4	0.76	0.62	0.6	no
	5	1.17	1.13	0.96	yes
	6	1.01	0.79	1.03	yes
	6	1.03	1.06	1.19	yes
	8	1.1	1.18	0.41	yes
	9	0.54	0.71	2.57	yes
	10	0.56	0.51	0.69	yes
Group B	11	0.76	0.85	0.29	no
	12	1.4	1.91	0.22	yes
	13	0.76	0.83	0.19	no
	14	1.22	1.11	0.19	yes
	15	0.9	0.93	0.06	yes
	16	1.14	1.13	0.17	no
	17	0.88	0.85	0.03	yes
	18	1.64	1.93	0.09	no

**Table 2 (abstract A137).** Fisher’s exact test

	Group A patients	Group B patients	Total
	(n)	(n)	
Postoperative AKI	9	4	13
(n)			
No postoperative AKI	1	4	5
(n)			
Total	10	8	18

### A138 Digital skills in healthcare professionals

#### M.L. Simonetti ^1^, G. Sciamanna ^2^, N. Tiberi ^2^

##### ^1^ Università Politecnica delle Marche, ASCOLI PICENO, Italy; ^2^ Università Politecnica delle Marche, Ascoli Piceno, Italy

###### **Correspondence:** N. Tiberi

*Journal of Anesthesia, Analgesia and Critical Care 2023,* **3(Suppl 1):**A138

Introduction

This study aims to investigate the level of digital competence of physicians and nurses as a whole. These are health professionals who are required to understand digital tools and how to use them effectively. They must consider the increasing widespread implementation of IT platforms for documentation of nursing activities, and the consultation of diagnostic examinations which takes place electronically. Many studies claim that digital technology in the Public Administration brings advantages in terms of speed of acquisition. This leads to an increase in the timeliness and effectiveness of healthcare interventions. Scientific evidence suggests that among the determinants that facilitate the implementation of digital technologies to support the main processes managed on a daily basis are re-fresh distance learning courses available online for both newly hired and experienced staff, offered by their own Company. These extra training courses will ensure that all health professionals are well equipped to use digital tools with efficiency.

Methods

The online data collection was carried out between the months of October to December 2022, by means of a message sent on both email and whatsapp. The message contained an access link with the subject 'The Anonymous revised Questionnaire of Digital Health Literacy (IT-eHEALS) which requires informed consent from the participants. This questionnaire focuses on the assessment of technical knowledge in the use of a representative sample of professionals. These are specifically the employees of San Benedetto del Tronto Hospital. The data collected was extrapolated through the use of a Google Forms application an Excell database. Where the results were compared and analytically re-elaborated with the SPSS

Results

335 questionnaires were analyzed (n.262 nurses, n.73 physicians) for both sexes. The most populous age group was the decade between 26-35 years, accounting for 42.7%. The vast amount of the sample (52.7%) have been active working for at least 15 years, the majority of nurses (51.7%) have a bachelor's degree as their qualification, followed by 25.7% also obtaing a master's degree at university. The maximum score cut-off for the quizzes was 25 points for 25 questions, the average score of the entire sample was 18.5. Between the two professions, nurses average score equarate slightly higher than the score of the doctors (20.3% versus 18.6%).When measured against the question on the importance of digital skills, 76.6% believe that they are very important in everyday working life. regarding the desire to implement digital training courses the majority (71.7%) expressed the desire to attend training courses. Among the factors favoring the level of digital performance, the variables were significant: being < 49 years of age (OR=1.94, 95% CI 1.74-2.15) level of university education (OR=2.06, 95% CI 1.90-2.24) seniority of company service >20 years (OR= 1.17, 95% CI 1.07-1.28). The correlation of the score with the frequency of use of pubmed was significant only for physicians (0.32, P<0.001)

Conclusions

Digital health is not the simple application of an app, but the strategy that underlies its implementation: mobile, hybrid cloud, cybersecurity, big data, artificial intelligence, blockchain, automation and robotics.

### A139 European survey on artificial intelligence and telemedicine in the field of anaesthesiology, intensive care and pain medicine

#### E.G. Bignami , M. Russo, R. Lanza, E. Ori, F. Bezzi, V. Bellini

##### Anesthesiology, Intensive Care and Pain Medicine Division, Department of Medicine and Surgery, University of Parma, Parma, Italy

###### **Correspondence:** M. Russo

*Journal of Anesthesia, Analgesia and Critical Care 2023,* **3(Suppl 1):**A139

Background

The knowledge among physicians of the role of artificial intelligence and telemedicine is under-investigated. For this reason, aims of the study were to explore the main concerns relative to these technologies and understand what actions can be taken to achieve an adequate implementation in clinical practice.

Materials and Methods

This voluntary survey was carried out on behalf of the Board of Directors of ESAIC and SIAARTI (from December 28, 2022, to October 29, 2022). The ESAIC and SIAARTI secretariats emailed the survey link to their active members specialized in anesthesia, resuscitation, intensive care and pain medicine were accepted. The survey consisted of 39 questions and less than 10 minutes were required to complete it.

Results

A total of 4465 e-mails were sent and 220 specialists [age 46.53±10.23; 128 males (58.2%)] responded to the survey. Most part of respondents’ working activity was represented by anesthesia, followed by intensive care and pain management, 55.9% had more than 16 years of work experience. The geographical distribution includes people working worldwide, with most of the replies comin from Italy [104/220 (47.2%)]. (Figure 1) shows the heatmap of italian regions based on the number of responses.

Knowledge about artificial intelligence and machine learning was reported in 207/220 (94.1%) and 180/220 (81.8%) members, respectively. In anaesthesiology, 168/220 (76.4%) and 151/220 (68.6%) have heard of artificial intelligence and machine learning. In intensive care, 154/220 (70.0%) and 133/220 (60.5%) heard of artificial intelligence and machine learning, while these numbers were much lower in pain medicine [artificial intelligence: only 70/220 (31.8%) and machine learning 67/220 (30.5%)]. The main barriers to implementing these tools in clinical practice resulted to be: i) lack of knowledge of algorithms leading to the results; ii) presence of few validation studies available, iii) not enough knowledge on artificial intelligence. Knowledge about telemedicine was reported in 212/220 (96.4%) members. Interestingly, 95.9% believed that training can lead to a greater use of these technologies. 90.0% of respondents declared themselves available to attend these courses and for the 81.4% of participants these new technologies will not replace their work.

Conclusions

The general approach of anesthesiologists, intensivists, and pain management clinicians towards artificial intelligence and telemedicine was overall positive. Ethical and legal issues and the lack of explainability of certain algorithms represented the main deterrents to the application of artificial intelligence in clinical practice. However, most participants did not consider these technologies as a threat to their profession.

**Fig. 1 (abstract A139).**
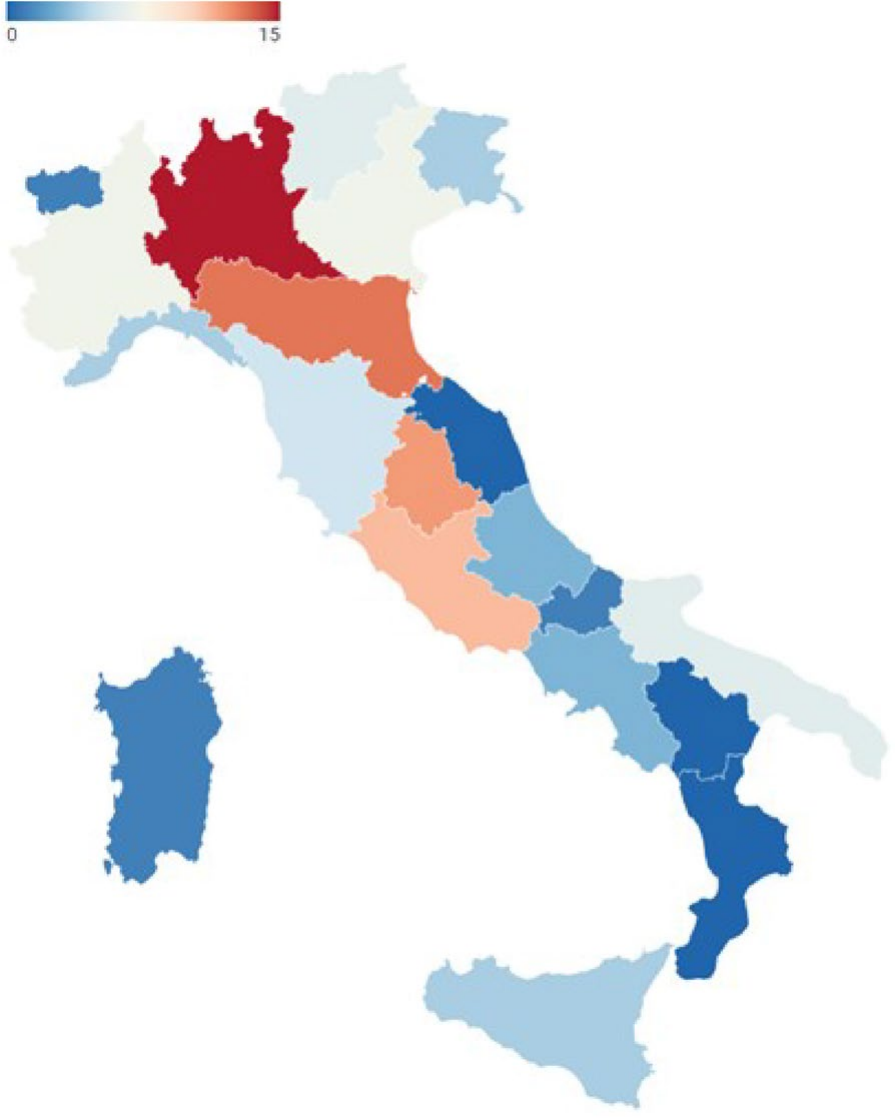
Heatmap representing geographical distribution of the participants’ working centers in Italy.

### A140 The assessment of ventricular-arterial coupling in critically ill patients: A report of three cases

#### D. Petronaci ^1^, C. Marino ^1,2^, F. Vitale ^1^, G. Accurso ^1^, A. Puglisi ^1^, S.M. Raineri ^1,2^

##### ^1^ Department of Anesthesia, Intensive Care, and Emergency, Policlinico Paolo Giaccone, University of Palermo, Palermo, ITALY; ^2^ Department of Surgical, Oncological and Oral Science (Di.Chir.On.S.), University of Palermo, Palermo, ITALY

###### **Correspondence:** D. Petronaci

*Journal of Anesthesia, Analgesia and Critical Care 2023,* **3(Suppl 1):**A140

Background

The assessment of ventricular arterial coupling (VAC) has been shown to provide help in understanding the contribute of circulatory impairment and ventricular disfunction in hemodynamic instability of critically ill patients [1]. VAC-guided hemodynamic optimization protocols have also been proposed for the management of septic shock’ patients [2], improving the outcome and discriminating phisiopatology of septic and cardiogenic shock [1].

We present three cases of bedside assessment of ventricular arterial coupling performed in patients with suspicion of septic shock and admitted to ICU. Consent to publish was obtained from the next of kin.

Case report

Case 1

74-year-old female admitted in ICU for hemodynamic instability after total pancreatectomy surgery, with diagnosis of septic shock. Maximal dose of norepinephrine and terlipressin 0.7 mcg/kg/h were used to support the circulation. Patient underwent a TTE assessment of ecocardiography-derived estimates of EF (39%), left ventricular end-systolic elastance, arterial elastance and VAC in a single-beat determination, that found a VAC 1.4 (Ea 2.1 mmHg/ml, Ees 1.5 mmHg/ml), highlighting excessive peripheral vasoconstriction, impairing cardiac performance. Therapy was optimized by prescribing epinephrine 0.1 mcg/kg/m and a new control was performed: VAC 0.85 (Ea 2.2 mmHg/ml, Ees 2.6 mmHg/ml), demonstrating an optimized hemodynamic and an improved peripherally perfusion (testified by a decreasing value of lactates).

Case 2

46-year-old female admitted in ICU after neurosurgery for intraparenchymal hemorrhage. During the ICU stay, we performed a transesophageal echocardiography in a single-beat determination to estimate necessary parameters for VAC-determination because of hemodynamic instability for suspicious septic shock (circulation mantained by norepinephrine 0.2 mcg/kg/m). The transesophageal echocardiography showed a left ventricular disfunction with aortic valve vegetation, so a new diagnosis of left ventricular disfunction endocarditis-based was done. We calculated VAC of 1.80 (Ea 1.76 mmHg/ml, Ees 0.98 mmHg/ml). Therefore, an isotropic support with dobutamine 5 mcg/kg/m was introduced. At the new TEE echocardiography’s control, the value of VAC was 1.41 (Ea 1.9 mmHg/ml, Ees 1.35 mmHg/ml), showing optimized hemodynamic and perfusion indices.

Case 3

75-year-old male admitted in ICU for post-operative management for ROSC with a history of chronic atrial fibrillation. Since admission, patient presented hemodynamic instability treated with norepinephrine 0.2 mcg/kg/m and atrial fibrillation with high ventricular response, treated with amiodarone and esmolol. We performed a transthoracic echocardiography in a single-beat determination which highlighted an EF of 45% and right ventricular disfunction (TAPSE 12 mm) with moderate tricuspid regurgitation. We found a VAC of 1.91 (Ea 2.31 mmHg/ml, Ees 1.21 mmHg/ml), reason why enoximone therapy was introduced. A few hours later, a new evaluation pointed out a VAC of 1.16 (Ea 1.29 mmHg/ml, Ees 1.12 mmHg/ml), confirming a cardiac performance and hemodynamic optimization.

Conclusion

Ventricular arterial coupling assessment may be an interesting non-invasive tool for a better interpretation of the pathophysiology behind shock in critically ill patients’ hemodynamic instability.

References

1. Guarracino F, Baldassarri R, Pinsky M R. Ventriculo-arterial decoupling in acutely altered hemodynamic states. Crit Care. 2013; 17(2):213.
2. Guarracino F, Bertini P, Pinsky M R. Cardiovascular determinants of resuscitation from sepsis and septic shock. Crit Care. (2019) 23:118.

### A141 Negative Pressure Wound Therapy in crush syndrome: a valuable ally of polytraumatized patients

#### C. Ferrari ^1^, D. Pisani ^1^, P. Ferrara ^1^, P.N.M. Sallustio ^2^, M. Testini ^2^, M. Ribezzi ^1^

##### ^1^ U.O.C. Anestesia e Rianimazione I universitaria A.O.U.C. Policlinico di Bari, Bari, Italy; ^2^ U.O.C. Chirurgia Universitaria V. Bonomo Dipartimento di scienze biomediche ed oncologia umana, Bari, Italy

###### **Correspondence:** C. Ferrari

*Journal of Anesthesia, Analgesia and Critical Care 2023,* **3(Suppl 1):**A141

Trauma is the main cause of death for people aged from 5 to 29 years.

Negative pressure wound therapy is a widespread tool used for wound healing [1].

Negative pressure therapy was applied to a 16th-old boy with crush syndrome.

Informed consent to the publication of this case report was obtained.

A 16th-old boy was hospitalized at Policlinico di Bari Emergency Department, injured in a tractor accident.

The patient showed many wounds all over his body and a large hematoma of the left thigh; enterorrhagia and gross hematuria were also observed.

The CT scan showed “open book” pelvic fracture, medium gluteus haematoma with active bleeding, pneumoperitoneum and transection oh the urethra. The patient underwent colonoscopy with finding of rectal perforation into the ischiorectal space: a balloon catheter was posed into the hole to avoid contamination and a bowel diversion and left colostomy was performed. A suprapubic cystostomy was also carried out.

The patient developed crush syndrome with AKI and rhabdomyolysis, treated with RRT combined with Cytosorb, hepatic acute failure and septic shock.

After 15 days, a CT scan revealed multiple intra-abdominal and pelvic collection and massive left gluteus muscle ischemia occurred; a multidisciplinary team decided to install negative pressure with instillation device. In the next few days, the inflammatory markers got lower, the patient passed through septic shock, nephrostomy was practiced due to the recovery of renal function, and MRI also revealed reduction of the intra-abdominal and pelvic collections. After three months, the patient was moved to general surgery ward and the vacuum device was removed because the scar was completely closed. (Figure 1)

Crush syndrome is a fearsome evolution of trauma and it is associated with a poor outcome because it starts a cascade of events that leads to M.O.F. and death [2]. Negative pressure in case of massive ischemia and necrosis, promotes the healing process, increasing blood supply, stimulating granulation tissue, removing inflammatory cytokines and metalloprotease.

The application of negative pressure with instillation device, together with standard intensive care, may enhance wound healing and may reduce the length to stay, ensuring the discharging from intensive care units without unnecessary delay.

References

1. Kim PJ, Attinger CE, Constantine T. Negative pressure wound therapy with instillation: International consensus guidelines update. Int Wound J. 2020 Feb;17(1):174-186.
2. Long B, Liang SY, Gottlieb M. Crush injury and syndrome: A review for emergency clinicians. Am J Emerg Med. 2023 Apr 25;69:180-187.

**Fig. 1 (abstract A141).**
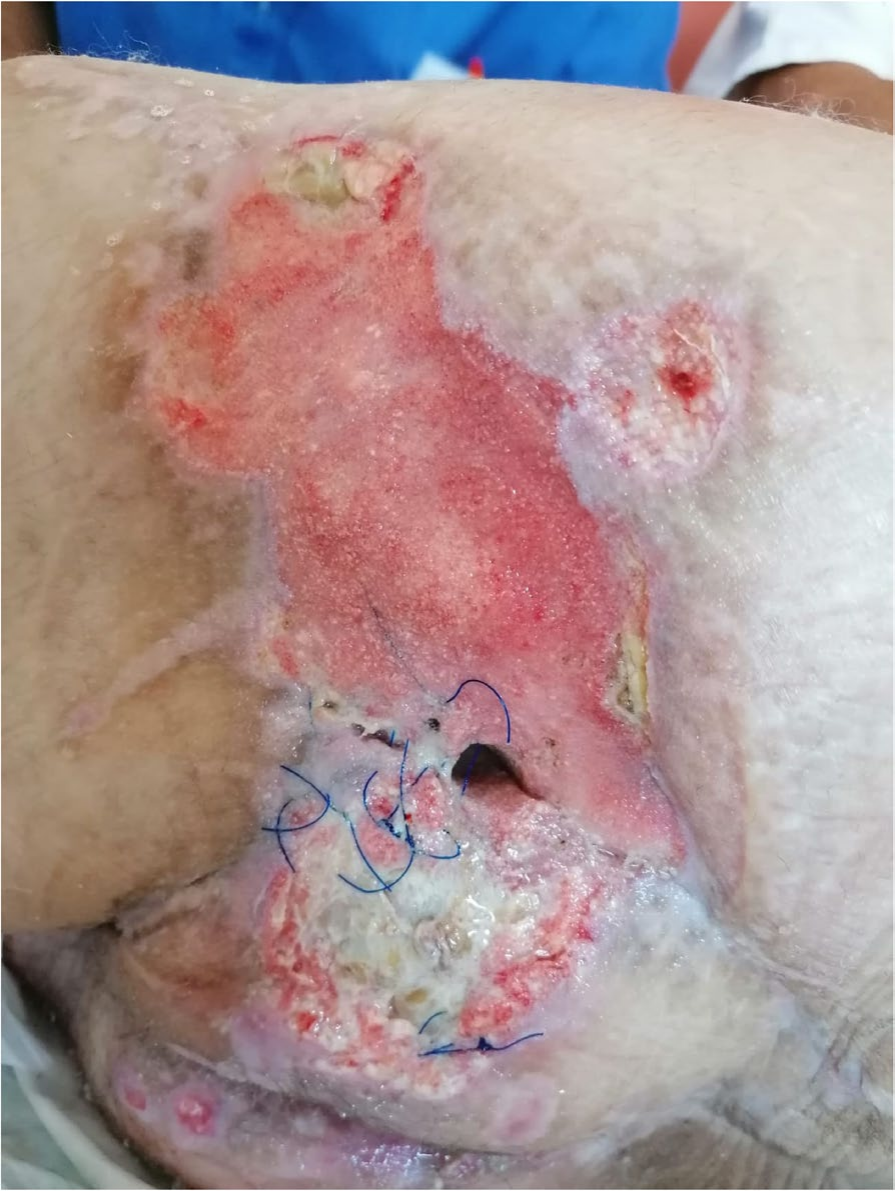
See text for description.

### A142 Use of CytoSorb in mushroom intoxication due to amanita phalloides

#### P. Ferrara ^1^, C. Ferrari ^1^, D. Pisani ^1^, G. Perchiazzi ^2^, F. Puntillo ^3^, M. Ribezzi ^1^

##### ^1^ Anesthesia and Intensive Care Unit I, Bari University Hospital, BARI, Italy; ^2^ Hedenstierna Laboratory and Central Intensive Care Unit, Department of Surgical Sciences, Uppsala University, Uppsala, Sweden; ^3^ Anaesthesia, Intensive Care and Pain Unit, Department of Interdisciplinary Medicine, University of Bari Aldo Moro, Bari, Italy

###### **Correspondence:** P. Ferrara

*Journal of Anesthesia, Analgesia and Critical Care 2023,* **3(Suppl 1):**A142

BACKGROUND

Amanita Phalloides is a poisonous mushroom belonging to Amanitaceae family. The fungus acts mainly by inhibiting the functions of the liver, due to amanitin substance. This molecule interacts with the mRNA of cells, preventing DNA transcription and blocking the activities of the organs1,2.

Symptoms develop in 4 stages: Phase 1: the incubation period of the toxin inside the organism;

Phase 2: the first symptoms are felt at the gastrointestinal level and include nausea, vomiting and diarrhea non-stop, severe dehydration and renal failure;

Phase 3: the liver is affected, with a very profound alteration in bilirubin and transaminase levels, leading to internal bleeding;

Phase 4: the liver goes into necrosis, leading to death in a short time1,2,3;

CASE REPORT

A 53 years old female was admitted to first level hospital in the Bari’s province with vomiting, hypotension, diarrhea and anuria. Tree day before, the patient had eaten mushrooms collected in the Basilicata woods. The patient was resuscitated and transferred to our Intensive Care Unit with cardiovascular insufficiency, metabolic acidosis and hyperlactacidemia. We started broad spectrum antibiotic therapy and contacted the Poison Control Centre while arranging for amanitin determination with ELISA method on urine samples. Laboratory parameters indicated severe leukocytosis plus elevated bilirubin, aspartate aminotransferase and alanine aminotransferase concentrations; prothrombin time and activated partial thromboplastin time were altered platelets were low. We contacted the liver transplantation center to enlist the patient for emergency liver transplantation. After confirmation of the presence of amanitin in urine we started CytoSorb blood purification therapy. The adsorption cartridge was used post hemofilter in combination with CRRT CVVHDF mode. The next day the patient underwent a liver transplant. Preoperative analyses showed amanitin reduction, improvement of inflammation and indices of liver necrosis. CRRT + CytoSorb was maintained during liver transplantation until 2 hours to the end of surgery. The patient was transfused. After 10 hours of surgery the patient returned to the intensive care unit. where after one hour underwent CRRT CVVHDF + CytoSorb. After 2 hours episode of tachycardia followed by malignant arrhythmias and cardiocirculatory arrest.

CONCLUSION

CRRT + CytoSorb probably allowed to stabilize the patient waiting for the liver transplant, lowering the load of aminitin. After about 24 hours of CytoSorb treatment, blood analysis showed a reduction of amantin from 12,22 ng/ml to 8,18 ng/ml, corresponding to a 33% in concentration . During surgery, CRRT + CytoSorb was continued up to the life of filter. Combined CVVHD/CytoSorb therapy was associated with a marked decrease in amanitin concentration and a stabilization of blood parameters.

Informed consent to publish had been obtained

References

1. Garcia J, et al : Amanita phalloides poisoning: Mechanisms of toxicity and treatment. Food Chem Toxicol. 2015 Dec;86:41-55.
2. Kieslichova E et al. Acute Liver Failure due to Amanita phalloides Poisoning: Therapeutic Approach and Outcome. Transplant Proc. 2018 Jan-Feb;50(1):192-197.
3. Ye Y, Liu Z. Management of Amanita phalloides poisoning: A literature review and update. J Crit Care. 2018 Aug;46:17-22.

**Fig. 1 (abstract A142).**
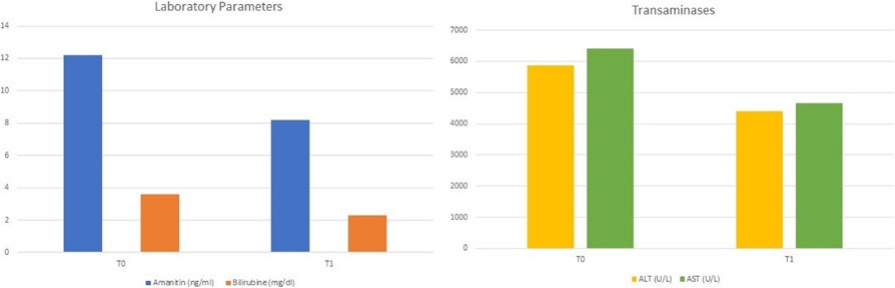
Amanitin, bilirubin and transaminases value value pre CRRT + CytoSorb treatment (T0) and after 24h of CRRT + CytoSorb treatment (T

### A143 Multiparametric telemonitoring in highly complex patients on home ventilation: advantages and limitations in identifying diagnostic therapeutic procedures

#### P. Di Masi, N. Cappellano

##### IRCCS Saverio De Bellis, Castellana Grotte, Bari, Italy

###### **Correspondence:** P. Di Masi

*Journal of Anesthesia, Analgesia and Critical Care 2023,* **3(Suppl 1):**A143

From January 2022 to May 2023, nine patients with neurodegenerative disease (amyotrophic lateral sclerosis), each of them on continuous artificial ventilation, underwent multiparametric telecontrol. The Telecontrol Unit (TU) has been structured to monitor the patients, managed by project referents and present study, contact persons, remotely assessed the oxygen saturation, blood pressure, body temperature, and electrocardiographic activity of the nine patients. Everything was managed through a home-based management system comprising a Web platform with application Sw and an Internet connectivity infrastructure. Alerts at the TU were defined in single form (T°sup 38.5, T°inf 35.5, SaO2 inf 90, SAP sup 170 inf 80, HR inf 50 sup 110) or packet form defined as 'Sepsis' (T°sup 38, SAP inf 90, HR sup 110), 'Acute pulmonary edema' (SaO2 inf 90, SAP sup 165, DAP sup 100), 'airway obstruction desaturation' (HR sup 100 SaO2 inf 90). Package alert was created for assessment of clinical problems with targeted identification of criticality. Package alerts and single alerts allowed real-time intervention with telephone and/or video call contact with the patient's care givers recommending procedures for clinical re-evaluation and possible re-hospitalization. After 17 months of observation (insertions at separate times of patients, with 2 deaths) data analysis allowed us to observe among single alerts as many as 85 for CF inf 50 (interventions to remodulate beta blocker therapies), 43 hypothermia alerts (adapted procedures to warm the patient), 39 for HR sup 110 (remodulated anxiety states and beta blocker therapies). Other single alerts were absent. Instead, package alerts present 2 for sepsis, 5 for airway obstruction. For each initiated measures with antibiotic therapy and airway clearance procedures (failure to drain airway). ECG transmission was operated nine times for further diagnostic investigation with subsequent transmission of the data to cardiology specialist, who in two cases allowed the identification of an atrial fibrillation arrhythmia with subsequent remodulation of ongoing therapy supported by home cardiology consultation. We can conclude how the daily observation operated by the TU allowed the adoption of procedures in a useful timing to prevent the evolution of various criticalities, creating a valuable support to home care givers and physicians in the territory. It's important to stess that everything have been considered in a not easy condition such as that of the home of a complex patient on artificial ventilation. There remains the limitation of a parametric remote sensing carried out not continuously, but with only one morning (or afternoon) 'check-in'. However, the care givers themselves were instructed to resend in real time coinciding with the occurrence of abnormalities detected bedside. Telemonitoring on smartphones by project referrers in such cases enabled with immediacy the alerting of referring physicians and in 7 cases ambulance emergency-emergency specialist. With telemonitoring, extemporaneity of diagnoses and initiation of procedures are important added values in 'home care of complex patients on artificial ventilation.

### A144 TELEHEALTH as a way to bring doctors to motionless fragile children

#### P. Cuofano ^1, 2^, F. Chiumiento ^2^, C. Chiumiento ^2^, L. Fornataro ^1^, M. Montefusco ^4^, P. Vuilleumier ^3^, A. Mignone ^1^

##### ^1^ Hospice il Giardino dei Girasoli - Medicina del Dolore - Cure Palliative - Centro NAD - DS 64 - Eboli - Asl Salerno - Italy; ^2^ UOC Anestesia e Rianimazione DEA Battipaglia-Eboli-Roccadaspide, Battipaglia Eboli Roccadaspide, Italy; ^3^ UOC Pneumologia e UTSIR AO Santobono-Pausillipon, Napoli, Italy; ^4^ UOC Direzione Sanitaria di Distretto 64 Asl Salerno, Eboli, Italy

###### **Correspondence:** P. Cuofano

*Journal of Anesthesia, Analgesia and Critical Care 2023,* **3(Suppl 1):**A144

BACKGROUND

The Hospice “Il Giardino dei Girasoli” - Pain Medicine - Palliative Care - NAD Center Unit located in Sanitary District 64 Eboli - Salerno - covers a district which has a pediatric population of about 10,000 children out of about 104.000 inhabitants and which spans over an area of 925.7 km2, provides home care to 11 fragile children affected by genetic neurodegenerative diseases (such as SMA 1), agenesis of the corpus callosum, epileptic disease, or terminal CRF on dialysis treatment.

Among them, 5 children are tracheostomized, 4 out of them connected to the mechanical ventilation, 8 children have PEG, others need specialist support.

Hospice provides help assistance for the management and periodic replacement of the PEGs and tracheostomy tubes (except for one child who needs assistance during this maneuver at the Pediatric Intensive Care Unit of the Battipaglia Hospital).

Since specialist checks or pneumological visits are also indispensable, children are often required to be transferred to the only specialised Pediatric Center in Campania, the Santobono-Pausillipon Hospital, in Pediatric Pulmonology Unit - Naples.

Unfortunately, due to the often weaker conditions of the young patients, arranging a transfer is not an easy task because children are required to travel in a CMR ambulance for up to 2 hours and for over 250 km (round trip). Moreover, such transfers heavily affect the budget of the NHS from an economic point of view.

MATHERIAL and METHODS

Following an agreement between Pediatric Pulmonology Unity and Hospice, the implementation of the TELEHEALTH enabled the management of virtual medical advice and checks, to remotely adapt the parameters of mechanical ventilation, to prescribe medical therapies, to carry out antibiotic therapy through professional nebulizers (i.e. VAP), all therapies that, without such new technology, would have required the physical presence in the hospital.

RESULTS

Hospital admissions decreased from 79 admissions made in the three-year period 2016-2019 to 18 in the three-year period 2019-2022, with a reduction by 77%.

CONCLUSIONS

The use of TELEHEALTH eases the hospital-territory integration, allows the checks of fragile children with a consequent reduction in hospital admissions and helps the cut of healthcare costs.

Authors have no conflict of interest.

REFERENCES

- Cady R, et al. A telehealth nursing intervention reduces hospitalizations in children with complex health conditions. J Telemed Telecare. 2009; 15:317-20.
- Cohen E, et al. Patterns and costs of health care use of children with medical complexity. Pediatrics. 2012; 130:1463-70.

### A145 Telemedicine for preanesthesia evaluation

#### Valentina Bellini, Christian Compagnone, Michelangelo Craca, Valeria Palermo, Giulia Borrini, Elena Bignami

##### Anesthesiology, Intensive Care and Pain Medicine Division, Department of Medicine and Surgery, University of Parma, Parma, Italy

###### **Correspondence:** M. Craca

*Journal of Anesthesia, Analgesia and Critical Care 2023,* **3(Suppl 1):**A145

Background

The use of televisit is spreading in many areas of medicine, including that of Anesthesia.

In this setting, in particular, the use of televisit seems to fit well with the needs of what is called prenesthesia evaluation. Our research group has developed a project with the aim of introducing telemedicine for pre-perative anesthesiological assessment.

Materials and Methods

The project includes the following phases (Fig 1):

1. Local survey aimed at highlighting doubts and perplexities related to the technique and elaborating a model of televisit that resolves these issues in advance;
2. Simulation sessions with both members of the anesthesia team and healthy volunteers to understand and prevent possible problems during service activation;
3. Development of a flowchart to identify patients eligible for televisit, selection by inclusion criteria and service activation;
4. Economic and organizational impact analysis post service activation.

Results

Currently, phases 1 and 2 of the project have already been addressed. Preliminary data are very encouraging; specifically:

- 96% of survey respondents state a device that can be used for televisiting, demonstrating the preliminary feasibility of the service;
- only 18% think that televisit would not bring any benefits, while 45% expect economic benefits and 44% time savings;
- in the simulation with experts and healthy volunteers, 100% of anesthesiologists say they were able to perform the televisit without difficulty, and only 10% of volunteers said they felt uncomfortable about the modality of the visit.

These findings are allowing the project to continue.

Conclusions

The novel advanced technologies are revolutionizing the healthcare system, increasing the tools available. A well designed digital transformation process is the key to minimize threats and take advantage of the opportunities these technologies offer. We believe that as in pharmacological studies in which a series of phases, design, testing, determination and drug approval, must be respected, the introduction of a new technology must also be tested on several levels before being incorporated into daily clinical practice. For this reason it is important to be able to have models of implementation that can guide in this technological transition. In addition, the proposed study design, adapting from the beginning to the needs of final users, could serve as a model for the activation of telemedicine services exportable to any other health facility.

**Fig. 1 (abstract A145).**
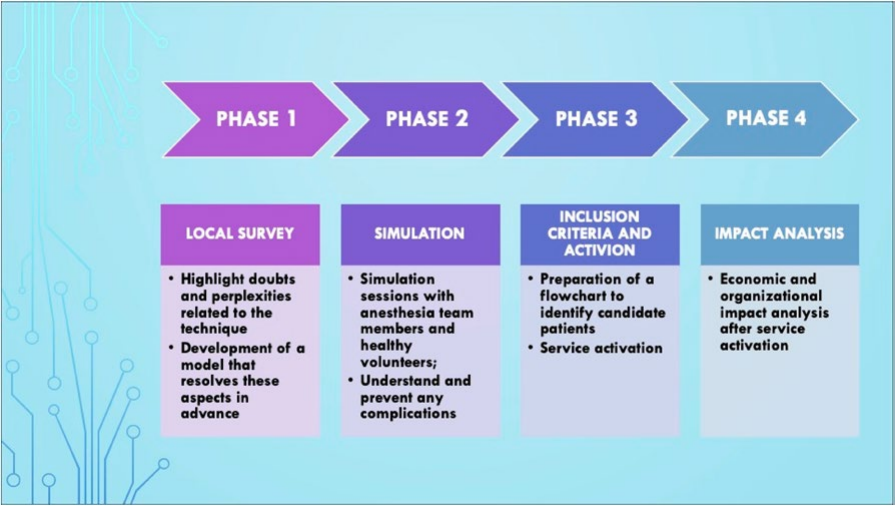
See text for description.

## Obstetrics and perinatal

### **A146 Fetal and maternal outcomes and adverse events during the pandemic: the possible impact of COVID-19 on pregnancy**

#### M. Vettorello ^1,3^, C. Bulfoni ^1^, R. Bienati ^1^, C.G. Achilli ^1^, S. Crippa ^1^, V. Palladio ^3^, M.G. Meroni ^1^, R. Fumagalli ^1,2^

##### ^1^ ASST GOM Niguarda, Milano, Italy; ^2^ Università Milano Bicocca, Milano, Italy; ^3^ Università degli studi di Milano, Milano, Italy

###### **Correspondence:** S. Crippa

*Journal of Anesthesia, Analgesia and Critical Care 2023,* **3(Suppl 1):**A146

Introduction

Coronavirus disease 2019 (COVID-19) has caused a global pandemic. There is a growing body of evidence supporting its effects on organs carrying ACE2 receptors such as the placenta. A higher incidence of pregnancy related complications in SARS-CoV-2 positive mothers such as restricted fetal growth or miscarriage seems to be significative. In this study we aimed to retrospectively analyse the differences in fetal and maternal outcomes and adverse events that may be related to placental involvement by COVID-19 between pre-pandemic, pandemic and early post-pandemic periods.

Methods

Retrospective analysis of original obstetric records of ASST GOM Niguarda, Milano, a tertiary italian hospital, hub for materno-fetal pathology, focusing on pre-pandemic versus pandemic differences in materno-fetal outcomes. Continous data were analysed with one-way analysis of variance (ANOVA) and categorical data with Mann-Whitney U test. Statistical significance was set at a two tailed p-value of <0.05.

Statistical analysis was performed with AnalystSoft, StatPlus Version v8.

Written informed consent for data collection and use was obtained at hospital admission.

Results

Overall fetal morbidity increased between 2020 and 2022 compared to pre-pandemic with an Odds ratio (OR) 2.54 (2019-2021) peaking in 2021 to 9.95% (Table 1).

This increase was mostly due to intrauterine growth retard (IUGR) (1.27% in 2019 to 2.97% in 2021, p<0.05). Preterm birth and fetal death incidences did not differ significantly.

The incidence of preeclampsia was higher in 2021 than in 2019 with an OR 2.48. There was no correlation between covid infection at admission and maternal and fetal outcomes. All the women who tested positive for Sars-Cov2 were asymptomatic or had mild symptoms.470

Conclusions

Access to healthcare facilities was not postponed during the pandemic since gestational weeks at admission (for spontaneous labor, cesarean section or induction) in our records were similar during the pandemic and in 2019.

Nonetheless this study detects an increase in fetal disease, mostly IUGR, in preeclampsia and in pathologic pregnancies (Table 2) generally speaking, during the pandemic with a trend towards normalization early this year.

Since we did not find a direct correlation between testing positive for Sars-Cov2 at admission and adverse outcomes, we suggest that adverse events might depend on Sars-Cov2 infections prior to pregnancy. There are limited data on the impact of COVID-19 on pregnant women and their babies but few studies suggest an increase in miscarriages and preterm births but only in women with Sars during pregnancy.

**Table 1 (abstract A146).** Fetal outcomes and averse events. (IUGR: intrauterine growth retardation; OR:Odds Ratio)ù

	2019 n=1955	2020 n=1900	2021 n=1947	2022 n=2042	2023 n=842	p
**fetal morbidity**	4.24%	8.94%	9.95%	9.05%	7.36%	**<0.0001**
**IUGR**	1.27%	2.47%	2.97%	2.20%	2.13%	**0.008**
**Preterm birth**	6.33%	7.26%	6.57%	6.60%	6.89%	0.83
**cardiac disease**	1.07%	1.74%	1.33%	1.54%	0.95%	0.38
**neurologic disease**	0.10%	0.10%	0.10%	0.15%	0%	0.94
**fetal death (>500mg)**	0%	0.26%	0.05%	0.098%	0%	0.33

**Table 2 (abstract A146).** Mother adverse events and morbility. (PROM: premature rupture of membranes)

	2019 n=1955	2021 n=1954	p
**women age (yrs)**	33 (29-37)	34 (30-37)	**<0.05**
**PROM**	31.8%	32.1%	>0.05
**gestational week**	39.2 (38-40.1)	39 (38-40)	**<0.05**
**physiological pregnancy**	48.4%	40.3%	**<0.0005**
**III labor phase length (min)**	7	5	>0.05
**Preeclampsia**	0.35%	0,87%	**<0.05**
**blood loss (ml)**	300 (200-500)	300 (200-400)	>0.05
**instrumental delivery**	3.8%	4.5%	>0.05
**urgent cesarean sections**	9.2%	9.9%	0.45
**total cesarean sections**	22%	21.3%	0.61
**labor induction**	26.6%	32.6%	**0.0017**
**Intensive care admission**	0.25%	0.26%	>0.05

**Fig. 1 (abstract A146).**
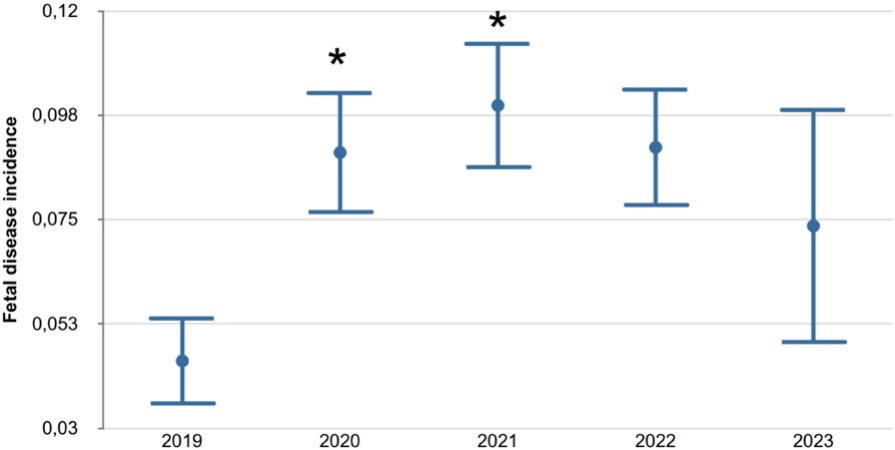
Incidence of fetal disease (2019-2023). *p<0.05

**Fig. 2 (abstract A146).**
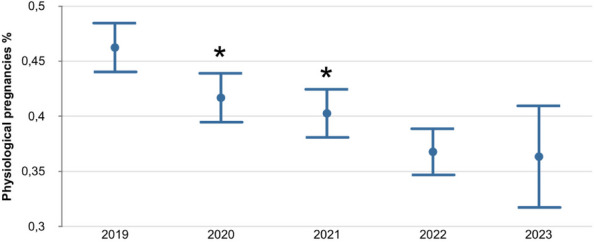
Proportion of physiological pregnancies (2019-2023). *p<0.05

### A147 Influence of epidural analgesia in induced labor course: comparison between prospectively enrolled nulliparous women and historical control group without analgesia

#### V. Palladio ^1^, M. Vettorello ^2,1^, S. Crippa ^2^, C.G. Achilli ^2^, R. Fumagalli ^3,2^

##### ^1^ Università degli studi di Milano, Milano, Italy; ^2^ ASST GOM Niguarda, Milano, Italy; ^3^ Università Milano Bicocca, Milano, Italy

###### **Correspondence:** S. Crippa

*Journal of Anesthesia, Analgesia and Critical Care 2023,* **3(Suppl 1):**A147

Introduction

A recent continuous Cochrane review comparing epidural analgesia for pain relief during labor and no analgesia found that epidural analgesia may influence the course of labor. Prolonged labor, increased use of oxytocin and increased assisted vaginal birth were found. The review included studies who recruited both multiparous and nulliparous women with spontaneous or induced labor. Only a few studies focusing on induced labor in nulliparous women exist comparing epidural analgesia to no analgesia. In this study we aimed to understand if Cochrane results can be extended to this subgroup of patients known for a higher request of analgesia.

Methods

We recruited prospectively 40 nulliparous women with a single cephalic pregnancy who underwent labor induction. Epidural analgesia was performed using ropivacaine supplemented with sufentanil and maintained through on-demand boluses or programmed intermittent boluses (PIEB). Historical control group included 130 nulliparous women with labor induction who did not ask for analgesia, although not restricted to no analgesia.

Informed consent to data recording and use was explicited at hospital admission.

We compared possible mother side-effects and newborn side-effects in both groups (No analgesia (NA) versus epidural analgesia (EA) (Table 1)). Statistical analysis was performed with AnalystSoft, StatPlus Version v8. Incidence comparisons were performed with Chi-square method while numerical variables were compared with Mann-Whitney U test for independent, non gaussian distributions. p values < 0.05 were considered for statistical significativity.

Results

We found an increase in length of first and second stages of labor in EA group (Table 2) and increased incidence in cesarean sections in NA compared to EA, 17.7% vs 2.5%, p=0.01, Odds ratio 8.38. Perineal outcome did not differ, while a trend towards a higher need for instrumental delivery in EA, although not significative can be seen. Newborn side effects as acidosis (arterial pH<7.2), BE reduction, Apgar<7 at 5 minutes did not show differences between EA and NA, while Apgar scores at 1 and 5 minutes were slightly lower in EA group, but without clinical meaning.

Conclusions

Epidural analgesia in nulliparous women with induced labor is associated with a longer duration of both stages but with a similar perineal outcome in terms of lacerations needing suturing and a lower incidence of cesarean sections. The incidence of operative delivery was similar, as well as estimated blood loss. Although early Apgar scores are slightly lower in EA, no differences were found in acidosis incidence, BE, meconium staining and no newborn had an Apgar score below 7 at 5 minutes in either group.

Labor epidural analgesia is therefore safe during induced labor in nulliparous women.

**Table 1 (abstract A147).** Population description (median (25-75 percentile)

	No Analgesia n=130	Epidural analgesia n=40	p value
**age (years)**	33 (29-3)	32 (29-35)	0.55
**gestational week**	39 (38-40)	39 (38-40)	0.28
**BMI**	27.9 (24.6-29.6)	27.6 (25.8-30.3)	0.37
**ASA**	2 (2-2)	2 (2-2)	0.35
**Surgical amniotomy(%)**	39,2%	89,5%	**<0.005**
**Fetal malposition**	6,7%	2.6%%	0.33

**Table 2 (abstract A147).** Outcomes

**Uterine atony (%)**	12%	5%	0.22
**Perineal trauma requiring suturing (%)**	46%	30,0%	0.79
**Perineal tear grade**	1 (0.5-2)	1 (0-2)	0.23
**Instrumental delivery (%)**	5%	13%	0.07
**Cesarean Section (%)**	18%	3%	**0.01**
**Blood loss (ml)**	300 (300-500)	300 (200-500)	0.35

### A148 Retrospective observational study on epidural anesthesia for caesarean section: the experience of tertiary care center

#### M. Pisanti ^1^, M. Loreto ^1^, S. Perna ^1^, G. Carnovale ^2^, L. Baiaino ^2^, R. Villani ^1^

##### ^1^ AORN A. Cardarelli, Napoli, Italy; ^2^ Azienda Universitaria Policlinico Federico II, Napoli, Italy

###### **Correspondence:** G. Carnovale

*Journal of Anesthesia, Analgesia and Critical Care 2023,* **3(Suppl 1):**A148

The paper is original not yet been published, the patients have given their informed consent.

Background

The aim of this study was to assess the efficacy of epidural anesthesia in intraoperative and postoperative pain management after cesarean delivery [1].

Materials and Methods

During an overall period of three years, a total of 1050 women were selected to undergo elective caesarean section under epidural anesthesia (ropivacaine 6.25% + sufentanil 1mcg/ml in 20 ml volume) and receive programmed epidural boluses (PEB) for postoperative analgesia. The adopted protocol foresaw the use of 3 PEBs of 10ml every 8h, with the possibility of adding extra top-ups. Each PEB had the same solution containing: Ropivacaine 0.2%, morphine 2mg and clonidine 75mcg in 10 ml volume.

The primary endpoint of this study was evaluation of intraoperative hemodynamic parameters, incidence of hypotension, and postoperative pain assested by NRS scale (0-10). The secondary outcomes were the total amount of PEB used in the 48h study period, mobilization time, time to first flatus, patient satisfaction and the incidence of complications such as PONV, dural puncture, accidental, catheter displacement,

Results

The incidence of hypotensive phenomena was noted only for 28% of patients, while in 72% there was no presence of signs of hemodynamic instability. The 93% of the patients there was no need of performing an intraoperative top-up. An increase in pain has been observed in the 3-6 hours following the end of the surgery (NRS 3-4 - 80%), with the need of administer the first PEB. The day after surgery and 36 hours later a decrease in postoperative pain was observed up to zero. In the majority of patients (78% of patients) no analgesic bolus was required 48 hours after surgery and it wasn't need to administer more PEBs. The total amount of scheduled PEBs was used in 786 (75%) women, while extra top-ups administration were required in 264 (25%) patients. In the case of insufficient analgesic response to the bolus it was allowed the administration of iv paracetamol 1g as part of our institutional multimodal protocol. A very low incidence (3%) of complications was observed, while the mobilization time after surgery was 55% in the first 8 hours and 100% in 12-18 hours, and the patient satisfaction (1 very poor-5 very good) was above 4 in 88% of the patients.

Conclusions

The epidural anesthesia it’s an extremely effective procedure in the management of intra- and postoperative pain for patients who undergo cesarean delivery.

References

1. Klimek M, Rossaint R, van de Velde M, Heesen M. Combined spinal-epidural vs spinal anaesthesia for caesarean section: meta-analysis and trial-sequential analysis. Anaesthesia. 2018 Jul;73(7):875-888.
2. Maronge L, Bogod D. Complications in obstetric anaesthesia. Anaesthesia. 2018 Jan;73 Suppl 1:61-66.
3. Riveros-Perez E, Wood C. Retrospective analysis of obstetric and anesthetic management of patients with placenta accreta spectrum disorders. Int J Gynaecol Obstet. 2018 Mar;140(3):370-374.

### A149 What type of anesthesia for cesarean delivery? The experience of the Obstetric Anesthesia Service in Rimini

#### Maltoni ^1^, P. Brandolin ^1^, G. Rizzoli ^2^, S. Terenzi ^2^, F. Fracassi ^1^

##### ^1^ AUSL Romagna, Rimini, Italy; ^2^ Universita' Di Ferrara, Ferrara, Italy

###### **Correspondence:** P. Brandolin

*Journal of Anesthesia, Analgesia and Critical Care 2023,* **3(Suppl 1):**A149

Background

Locoregional anesthesia represents the gold standard for cesarean delivery (CD). Many studies have shown an increased risk of adverse events associated with general anesthesia in obstetric patients, therefore one of the goals of modern obstetric anesthesia is to minimize its use. Locoregional techniques for CD are single-shot spinal, epidural and combined spinal-epidural (CSE) anesthesia. Spinal anesthesia is easy to perform, has a rapid onset and provides excellent surgical conditions. Epidural anesthesia is better titratable, thus ensuring greater hemodynamic stability, can be repeated if surgery is prolonged and also be used postoperatively for pain control. Finally, CSE is characterized by a subarachnoid injection followed by the placement of an epidural catheter and subsequent administration of epidural drugs. It offers the advantages of both spinal and epidural techniques while reducing the risk of their complications and the failure rate related to either technique used alone [1,2]. Our aim is to evaluate how the anesthetic choice has changed after the institution of a 24h dedicated Epidural Analgesia Team in 2008 and the introduction of ERAS protocol for pain control after CD using patient-controlled epidural analgesia devices.

Methods

This observational study analyses data collected from computerized operating system Log80 in 2007 and in 2022 at Rimini's Hospital, a level II maternity care center with more than 2500 deliveries/year.

Results

In 2007 spinal anesthesia was the most used technique for CD while in 2022 its use was reduced by more than 50% due to the take hold of epidural and CSE techniques especially for emergent and more complicated CDs. In 2022 epidural anesthesia was practiced in 1/3 of total CDs as conversion from labor analgesia when urgency criteria existed. While in 2007 CSE wasn’t used at all, in 2022 it was used for elective CDs especially in complicated cases (e.g. previous CD, twin pregnancy, comorbid patients) according to ERAS protocols. General anesthesia has always been the least used and was performed only in selected cases where locoregional anesthesia failed or was contraindicated (Table 1) (Fig 1).

Conclusions

Within 15 years the 24h availability of an obstetric anesthesiologist, the growing use of labor analgesia and the introduction of ERAS protocols have determined a significant change in the choice of anesthetic techniques with CSE as the standard technique for elective CDs and epidural for urgent CDs with catheter already positioned. This led to the improvement of maternal postoperative course due to an early mobilization and discharge from hospital.

References

1. Roofthooft E, Rawal N, Van de Velde M. Current status of the combined spinal-epidural technique in obstetrics and surgery. Best Pract Res Clin Anaesthesiol, 2023. Article in press.
2. Klimek M, Rossaint R, van de Velde M and Heesen M. Combined spinal-epidural vs. spinal anaesthesia for caesarean section: meta-analysis and trial-sequential analysis. Anaesthesia 2018, 73, 875–888.

**Table 1 (abstract A149).** Type of anesthesia

	2007	2022
Total CDs/ deliveries	884/2749 (32%)	535/2664 (20%)
Spinal anesthesia	813 (92%)	201 (37%)
Epidural anesthesia	0	180 (34%)
Combinated anesthesia	0	138 (26%)
General anesthesia	71 (8%)	16 (3%)

**Fig. 1 (abstract A149).**
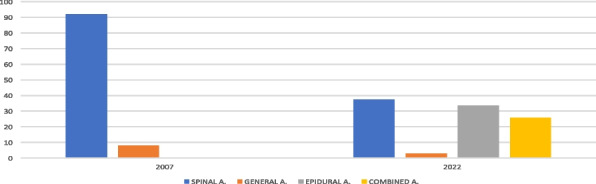
Anesthesia techniques for cesarean delivery in Rimini’s Infermi Hospital

### A150 Moschcowitz syndrome should not be underestimated

#### D. Fiume ^1,2,3^, F. Sciarpelletti ^1^, M. Martorelli ^1^, D. Ferraccioli ^1^, L. Coen Tirelli ^2^, A. Tiberi ^2^, F. Marchetti ^2^, S. Carlini ^2^, A.M. Martini ^1^, M. Peverini ^1^, M. Galletti ^1^

##### ^1^ Sant'Eugenio Hospital, Rome, Italy; ^2^Tor Vergata University, Rome, Italy; ^3^ UniCamillus University, Rome, Italy

###### **Correspondence:** A. Tiberi

*Journal of Anesthesia, Analgesia and Critical Care 2023,* **3(Suppl 1):**A150

BACKGROUND

Thrombotic thrombocytopenic purpura (TTP), also known as Moschcowitz syndrome, was initially described one hundred years ago. TTP is a very dangerous syndrome that results in a low platelet count, low red blood cells and multiorgan failure. Clinically there could be fever, seizures, contusions, weakness, confusion and headache. Often a trigger is identified, developing antibodies inhibiting ADAMTS13 enzyme. Without a diagnosis, or in case of delay, mortality can be more than 90%.

CASE REPORT

We report a case of a 34-old female patient, underwent at emergency caesarean section. Pathological cardiotocography and intrauterine growth restriction were observed. HELLP syndrome was suspected. She arrived at the emergency room with confusion, Hemoglobin: 6.3 g/dl, Platelets: 6.000/yl, stable vital signs. After 72 hours there was no recovery of blood chemistry values. In ICU, TTP was suspected, and apheresis started early. After 7cycles of apheresis patient was discharged from ICU with Hb 9.8 g/dl and PLTs 95.000 y/l, positivity at ADAMTS13.

CONCLUSIONS

Our case further confirms the importance of an early diagnosis in case of TTP.

Informed consent to publish had been obtained.

### A151 A case of HELLP plus Acute Kidney Injury with prolonged coagulopathy after massive obstetric hemorrhage

#### C. De Domenico ^1^, F. Pascucci ^2^

##### ^1^ Università degli Studi di Brescia, Brescia, Italy; ^2^ ASST Spedali Civili, Brescia, Italy

###### **Correspondence:** C. De Domenico

*Journal of Anesthesia, Analgesia and Critical Care 2023,* **3(Suppl 1):**A151

Background

Uncontrolled peripartum bleeding resulting in consumption coagulopathy and disseminated intravascular coagulation (DIC) is one of the leading causes of maternal mortality worldwide. DIC is the endpoint of uncontrolled systemic activation of the hemostatic system, leading to a simultaneous widespread of microvascular thrombosis, that may cause organ failure. The rate of DIC during pregnancy is about 0.03%. However, it often leads to adverse outcome including massive blood product transfusion, hysterectomy and maternal death [1].

Case report

A 36-year-old women (gravida 3 parity 2) at 38+0 week gestation presented to our hospital for induction of labor for a suspected large-for-gestational-age fetus. Her previous children were born through vaginal deliveries and her past medical history was unremarkable except for grade III obesity. After admission the state of both mother and fetus was reassuring; she gave birth to a healthy infant under epidural analgesia and the placenta was delivered spontaneously.

30 minutes later, heavy vaginal bleeding was observed and the patient developed impaired consciousness with seizure requiring intubation and benzodiazepine administration.

We started rapid fluid and blood resuscitation and we administered medications with uterotonic agents and intravenous hemostatic agent (additional oxytocin, sulprostone and tranexamic acid) combined with manual uterine compression. An intrauterine Bakri balloon was placed, but it did not control bleeding. A noradrenaline infusion at 0.6 mcg/kg/min was needed to maintain the arterial systolic pressure at 75 mmHg.

As these treatments failed, it was necessary to perform an embolization of the hypogastric arteries; however, it did not achieve hemostasis and hemodynamic instability persisted. Therefore an emergency hysterectomy was performed as a lifesaving procedure. Total estimated blood loss was 8400 ml in about 5 hours.

At the end, she was admitted in ICU and the laboratory tests showed anemia with schistocytes, thrombocytopenia, prolonged PT and aPTT, low fibrinogen and high AST-ALT-bilirubin levels. The patient also became anuric, with high serum urea and creatinine.

The combination of seizure, hemolysis, thrombocytopenia and elevated liver enzymes suggested a postpartum HELLP syndrome complicated by a DIC.

The treatment was instituted with fresh frozen plasma, red cell transfusion, fresh platelet, fibrinogen, clotting factors. After admission to ICU, persistent diffuse bleeding mainly caused by hyperfibrinolysis (Hb 6.4 g/dl, PLT 54’000/uL, aPTT 144.2 s, LDH 437 U/L) and renal failure occurred. For 15 days the patient required daily blood transfusion and renal replacement therapy as she manifested persistent anuria due to thrombotic microangiopathy resulting in renal cortical necrosis.

She was discharged from the Hospital with hypertension and adequate urinary output a month later.

Conclusion

Women with postpartum hemorrhage need to be treated promptly, pharmacologically, surgically and with blood products including clotting factors to maximize the survival possibilities. Close monitoring of the patients and of their laboratory values as well as restoration of both blood volume and coagulation factors are essential for the survival.

Informed consent to publish had been obtained.

References

1. Erez O, Mastrolia SA, Thachil J. Disseminated intravascular coagulation in pregnancy: insights in pathophysiology, diagnosis and management. Am J Obstet Gynecol. 2015 Oct;213(4):452-63.

## Security, Quality and Clinical Risk

### **A152 Enhanced safety in critical care: how a dedicated clinical pharmacist can improve drug prescription and administration in ICU**

#### A. Lerose ^1^, P. Maimone ^2^, P. Calligaro ^1^, F. Vargiu ^1^, E. Milani ^1^, S. Benedetti ^1^, F. Raffaele ^1^, V. Bertasi ^2^, D.S. Pascu ^3^, M. Carlini ^1^

##### ^1^ UOC Anestesia e Rianimazione, Villafranca (VR), Italy; ^2^ UOC Farmacia Ospedaliera, Villafranca (VR), Italy; ^3^ UOS Risk Management, Villafranca (VR), Italy

###### **Correspondence:** A. Lerose

*Journal of Anesthesia, Analgesia and Critical Care 2023,* **3(Suppl 1):**A152

Background

No clinical setting is error-free in relation to drug prescription. Nonetheless, different studies have shown a higher percentage of mistakes in critical care settings [1]. Risks related to drug prescription and administration come from both the intrinsic properties of the drug but can also be caused by other adverse events. These events can happen all along the prescription and administration process and can concur to a Patient Safety Incident (PSI). The aim of this study was to analyze the issues related to the management of pharmacologic therapy in Intensive Care and verify if these issues could be addressed with the help of a clinical pharmacist.

Materials and Methods

A clinical pharmacist has provided our Intensive Care Unit (ICU) with a booklet and other material containing information on how to handle drug administration safely. After the delivery of this informative material, a survey on clinical risk management regarding drug administration has been given to ICU staff. This survey consisted of 20 questions: 6 related to risk management and 14 related to prescription, transcription, preparation and administration of drugs. Results of this survey were analyzed to evaluate if the staff was prepared enough to handle appropriately pharmacological therapy, as per Ministerial Recommendations n. 7 and 12 [2,3].

Results

During the study period, the informative material was hanged on ICU walls and made available in a shared drive. The material included posters, preparation and administration charts, a list of LASA/SALA (Look-Alike/Sound-Alike) drugs and drugs’ technical sheets. After the study period the survey results (Figure 1) have shown a higher percentage of participation from the nurses (93%) compared to physicians (43%); only 25% of the nursing staff had ICU working experience of more than 5 years. The percentages of right answers were as follows: 82% in the prescription domain; 80% for transcription; 67%for preparation and dilution; 87% for administration and 66% for management of LASA/SALA drugs.

Conclusions

Mistakes caused by wrong administration of drugs have a significant impact on the quality of care and on patients’ safety. The help of a clinical pharmacist, who was deemed useful from the 93% of the staff who was interviewed, has provided active support and made available informative material for standardization of care as per Ministerial Recommendations. If the presence of a clinical pharmacist was to become permanent, it would lead to enhanced standards of safety in ICU and would also be a cost-effective measure [4].

References

1. Kane-Gill S.L., Kirisci L., Verrico M.M., Rothschild J.M. Analysis of risk factors for adverse drug events in critically ill patients*. Crit. Care Med. 2012;40(3):823–828. doi: 10.1097/CCM.0b013e318236f473.
2. Raccomandazione Ministeriale n.7 per la prevenzione della morte, coma o grave danno derivati da errori in terapia farmacologica.
3. Raccomandazione Ministeriale n. 12 per la prevenzione degli errori in terapia con farmaci “LOOKALIKE/ SOUND-ALIKE”.
4. F. Cattel et al., IL RUOLO DEL FARMACISTA IN OSPEDALE The Role of the Pharmacist in Hospital Giornale Italiano di Farmacoeconomia e Farmacoutilizzazione 2014; 6 (2): 16-24

**Fig. 1 (abstract A152).**
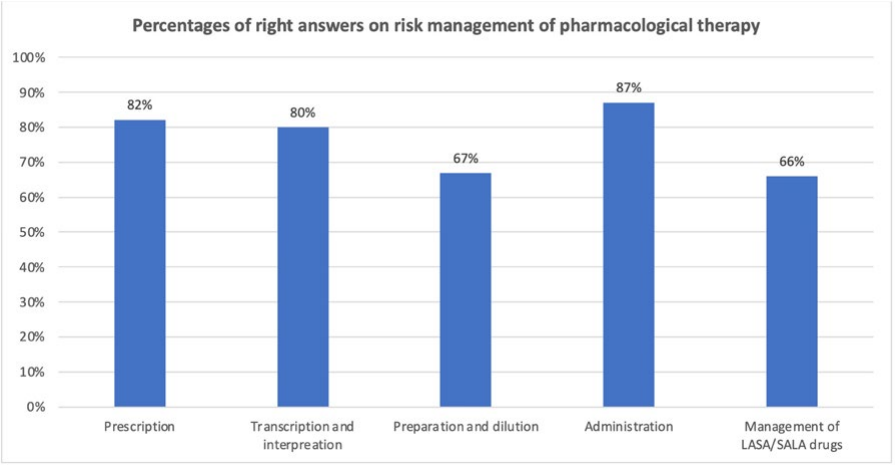
See text for description

### A153 RIsks of nighttime working as perceived by Italian anesthesiologists

#### A.N. Galvano ^1^, M. Ippolito ^1, 2^, A. Noto ^3^, I. Lakbar ^4^, S. Einav ^5^, A. Giarratano ^1, 2^, A. Cortegiani ^1, 2^

##### ^1^ Dipartimento Discipline Chirurgiche, Oncologiche e Stomatologiche - Università degli Studi di Palermo, Palermo, Italy; ^2^ Dipartimento di Anestesia, Terapia Intensiva ed Emergenza - AOUP P. Giaccone - Università degli Studi di Palermo, Palermo, ITALY; ^3^ Divisione di Anestesia e Terapia Intensiva - Policlinico G. Martino - Università di Messina, Messina, Italy; ^4^ Anesthesiology and Intensive Care; Anesthesia and Critical Care Department B, Saint Eloi Teaching Hospital, PhyMedExp, University of Montpellier, INSERM U1046, 1; 80 avenue Augustin Fliche, Montpellier cedex 5, Montpellier, France; ^5^ General Intensive Care Unit of the Shaare Zedek Medical Centre and the Hebrew University Faculty of Medicine, Gerusalemme, Israel

###### **Correspondence:** A.N. Galvano

*Journal of Anesthesia, Analgesia and Critical Care 2023,* **3(Suppl 1):**A153

Background

No data are available on the working conditions and workload of Italian anesthesiologists during perioperative nighttime work, and on the perceived risks.

Materials and methods:

A secondary analysis of an international survey promoted by European Society of Anaesthesiology and Intensive Care (ESAIC) [1] was performed extracting data from responders working in Italy. Descriptive statistics was used to present results.

Results

We analyzed 1085 responses out of the 5292 from the whole dataset. Most of responders (75.21%) declared working a median of 12 consecutive hours during nightshifts, with an irregular nightshift schedule (70.05%). More than half of the responders stated to receive a call 2-4 (40.37%) or 5 times or more (24.88%) to perform emergency procedures and/or ICU activities during nightshifts. More than 70% of the responders declared having relaxation rooms for nighttime work (74%) but none to be used after a nightshift before going back home (82%) and no free meals, snacks or beverages (89%). Furthermore, almost all (95.3%) the surveyed anesthesiologists declared not having received a specifical training or education on how to work at night and that no institutional program has been held by the hospital to monitor fatigue or stress for nightworkers (99.08%). More than half of the responders stated having the possibility, sometimes (38.52%) or always (44.61%), to involve another collegue for difficult medical decisions and to feel comfortable, sometimes (30.6%) or always (34.93%), to call the on-call collegue. Participants declared that nightwork affects their quality of life extremely (13.18%) or significantly (62.58%), and that sleep deprivation, fatigue and current working conditions may reduce performance (67%) and increase risk for the patients (73.18%). Responses to these questions are reported in Figure 1, in form of histograms, and in Table 1 in forms of numbers and percentage.

Conclusions

Italian anesthesiologists perform highly demanding activities during nightshifts, mostly without proper training. They perceive current practice as negatively affecting their quality of life, their performance and patients’ safety. Adequate training and education on nighttime work and institutional fatigue monitoring programs may improve anesthesiologists’ nighttime work conditions. This may reduce perceived risk towards patients’ safety and impact on their wellbeing.

References

1. Cortegiani A, Ippolito M, Lakbar I, Afshari A, Kranke P, Garcia CSR, Myatra SN, Schultz MJ, Giarratano A, Bilotta F, De Robertis E, Noto A, Einav S. The burden of peri-operative work at night as perceived by anaesthesiologists: An international survey. Eur J Anaesthesiol. 2023 May 1;40(5):326-333.

**Fig. 1 (abstract A153).**
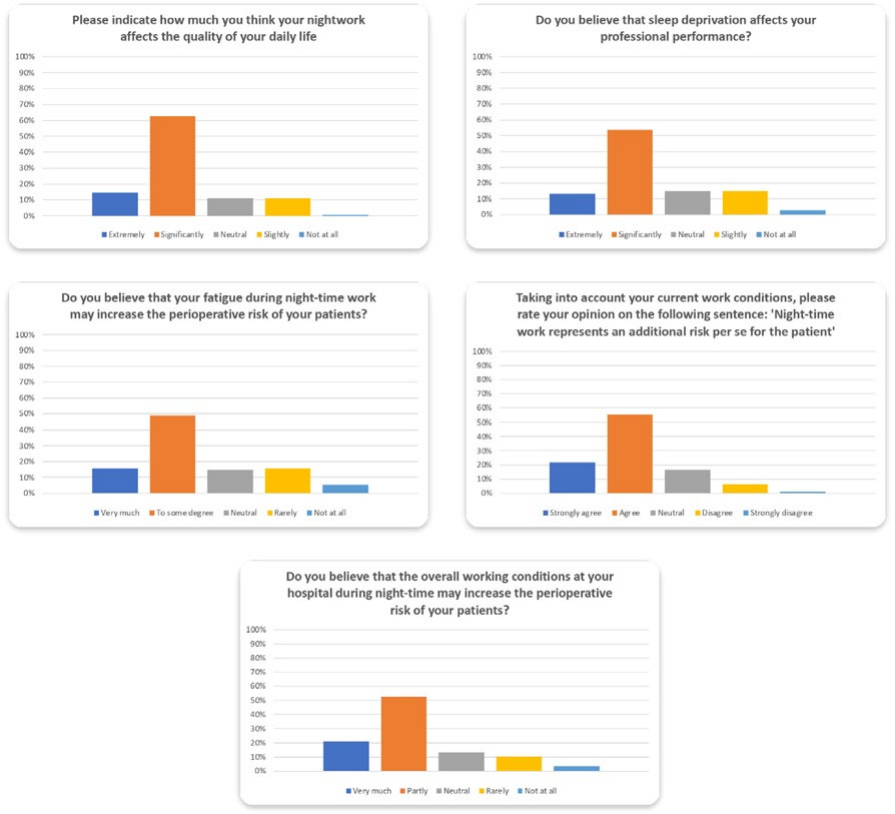
Each panel represents the answer (in percentage) to a single question, that is reported in the title of each panel

**Table 1 (abstract A153).** Results of questions on patients’ safety and doctors’ quality of life

		All
		(n=1085)
		N (%)
**Please indicate how much you think your nightwork affects the quality of your daily life**	*Extremely*	161 (14,84%)
	*Significantly*	679 (62,58%)
	*Neutral*	119 (10,97%)
	*Slightly*	119 (10,97%)
	*Not at all*	7 (0,64%)
**Do you believe that sleep deprivation affects your professional performance?**	*Extremely*	143 (13,18%)
	*Significantly*	584 (53,82%)
	*Neutral*	164 (15,12%)
	*Slightly*	163 (15,02%)
	*Not at all*	31 (2,86%)
**Do you believe that your fatigue during night-time work may increase the perioperative risk of your patients?**	*Very much*	170 (15,67%)
	*To some degree*	531 (48,94%)
	*Neutral*	158 (14,56%)
	*Rarely*	170 (15,67%)
	*Not at all*	56 (5,16%)
**Taking into account your current work conditions, please rate your opinion on the following sentence: 'Night-time work represents an additional risk per se for the patient'**	*Strongly agree*	234 (21,57%)
	*Agree*	600 (55,3%)
	*Neutral*	174 (16,04%)
	*Disagree*	66 (6,08%)
	*Strongly disagree*	11 (1,01%)
**Do you believe that the overall working conditions at your hospital during night-time may increase the perioperative risk of your patients?**	*Very much*	226 (20,83%)
	*Partly*	568 (52,35%)
	*Neutral*	144 (13,27%)
	*Rarely*	112 (10,32%)
	*Not at all*	35 (3,23%)

Data are reported as raw number and percentages

## Simulation

### **A154 Unleashing Excellence: Mastering the Art of Implementing a Sepsis Quality Improvement Program**

#### C. Ebm, S. Brusa, A.C. del Pozo, G. Poli, A. Barbarello, M. Cecconi

##### Humanitas University, Milano, Italy

###### **Correspondence:** A. Barbarello

*Journal of Anesthesia, Analgesia and Critical Care 2023,* **3(Suppl 1):**A154

Introduction: Sepsis remains a significant healthcare challenge, necessitating the implementation of effective improvement programs. However, the successful implementation of such programs requires addressing various challenges, including skepticism towards new educational methods, limited faculty time, and financial constraints. Our study aims to outline the challenges encountered during the implementation and describe the lessons learned from the process.

Methods: The implementation of the sepsis improvement program involved a multidisciplinary approach, involving clinicians, administrators, and other stakeholders. The program was designed to optimize sepsis recognition, timely intervention, and overall patient outcomes. Multiple strategies were employed, including education and simulation-based training, process standardization and risk stratification tools, and continuous data monitoring and analysis. Surveys were applied to track training progress and satisfaction with the program.

Results: Several challenges emerged during the implementation process. It became evident that engaging and continuously motivating faculty members was crucial for the program's success, as their sustained engagement was vital for program sustainability. Incentive programs and recognition initiatives were implemented to foster motivation and foster a culture of continuous improvement. To address this, regular faculty meetings and open communication channels, including direct messaging services, were established, fostering a sense of ownership and promoting collaboration. Additionally, the development of a project playbook helped to clearly define milestones, providing a roadmap for project progression and enabling effective tracking of program outcomes. Furthermore, trialing varied levels of fidelity levels within the simulation-based training significantly reduced costs while still ensuring optimal skill acquisition among hospital staff. Within the first-year knowledge on recognizing and treating a septic patient improved significantly, from 9 [8-10] out of 12 at pre-test to 10 [10-11] out of 12 at post-test (p<0.001).

Conclusion: The challenges encountered during the implementation of a sepsis improvement program emphasized the importance of people over processes, as expertise and commitment proved more influential than mere procedures. Engaging faculty members as active participants, defining milestones through a project playbook, reducing training costs through fidelity variations, and implementing motivation strategies were critical lessons learned. By recognizing and addressing these challenges, healthcare organizations can enhance their sepsis management efforts and improve patient outcomes in the face of this life-threatening condition.

## Invasive and interventional techniques

### **A155 Pulsed Radiofrequency Treatment for Trigeminal Neuralgia: case report**

#### E. Cianciola ^1^, F. Saturno ^1^, G. Monaco ^1^, F. Marino ^1^, S. Palladino ^1^, V. Bellini ^2^

##### ^1^ ASL Salerno-Ospedale dell'Immacolaa di Sapri, Salerno, Italy; ^2^ Azienda ospedaliera universitaria di Parma, Parma, Italy

###### **Correspondence:** F. Saturno

*Journal of Anesthesia, Analgesia and Critical Care 2023,* **3(Suppl 1):**A155

Background

There Trigeminal neuralgia is characterized by intense facial pain, paroxysmal and excruciating, due to an alteration of the fifth cranial nerve’s.

Case report

Patient 76 years old, with a history of surgically unattachable pituitary adenoma, has performed knifene gamma therapy in 2020 (Sterotaxic radiotherapy) for pain control with partial benefit for about one year. The patient came to us for seizures painful that exacerbated with chewing phonation. There patient was taking maximally dosed CBZ, lamotrigine and pregabalin with poor heart rate control painful symptoms and important side effects. For unresponsiveness to pharmacological therapy (VAS 10 diurnal VAS 9 nocturnal continuous need for rescue drugs), the patient decided to undergo surgery pulsed radiofrequency of the ganglion of Gasser (Figure 1).

The patient was positioned supine on a radiolucent bed with moderately extended head placed on donut-shaped cushion and with forehead immobilized by special fixation. The patient's head was rotated 15-20 degrees contralateral to the surgical site. The C arm was placed perpendicular to the patient's head allowing a antero-posterior projection and therefore was inclined in the caudal-cephalic direction of about 25 30 degrees. The foraramen oval was visualized medially to the incisura intercondylar of the mandible and laterally to maxillary sinus. A 10 cm long, 21G gauge RF needle was introduced by puncturing the cheek 2 - 5-3 cm laterally in correspondence with the labial fissure on the affected side. The needle was passed through the masseter muscle until the foramen ovale was reached, in lateral projection; successively, the needle was advanced by overlapping the profile of the clivus sphenoidale. (Figure 2).

The spindle was removed and the CSF leak was observed which attested the transition of the dura mater and the retroganglionic position. (Figure 3).

At the end of the procedure, the patient reported severe pain in the territory of the first trigeminal branch. In the following days, the patient presented paresthesias of the three trigeminal branches, which resolved on the third day (oral therapy with prednisone). At the 1-week post-procedure follow-up, the patient took only 25 mg pregabalin in the evening, VAS 0. After 2 months, the patient still reported no pain and he was not taking any pain medication. We collected adequate consent to participate in the study.

Conclusion

The intervention of pulsed radiofrequency to the ganglion of Gasser is a complex technique burdened by important complications, extensively described in literature. However our case series in three years is comforting, with 19 out of 20 patients reporting marked improvement in symptoms painful. There patient selection must be careful, selecting patients only non-responders to medical therapy and with poor quality of life. Patients must be adequately informed about the adverse events of the procedure and the possibility of treatment failure.

**Fig. 1 (abstract A155).**
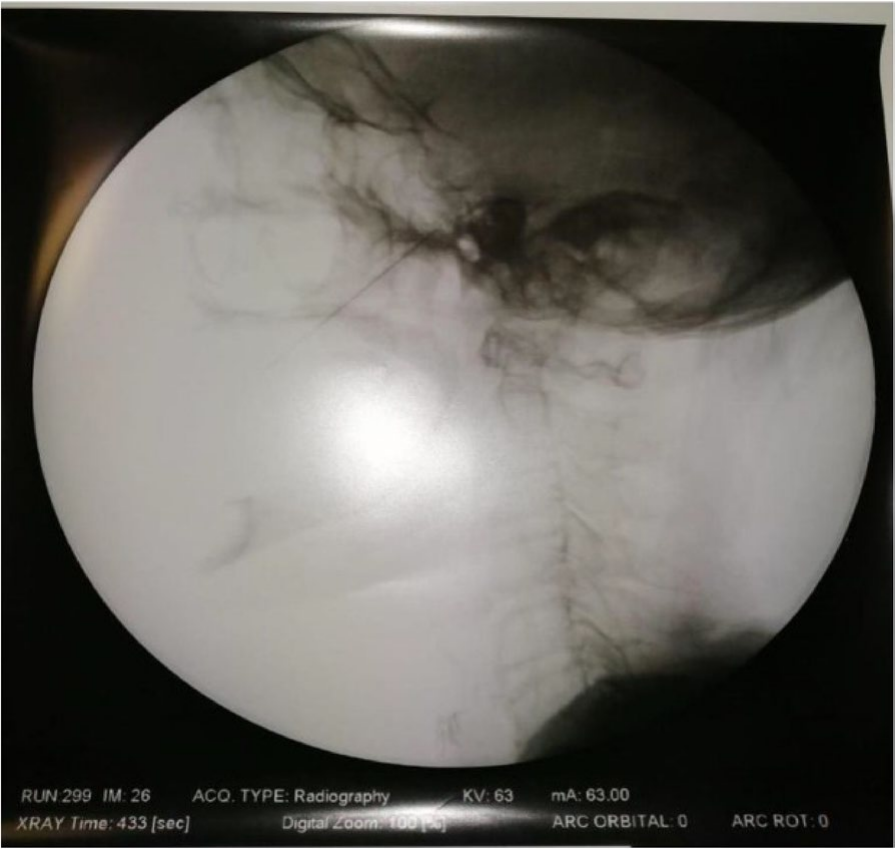
See text for description

**Fig. 2 (abstract A155).**
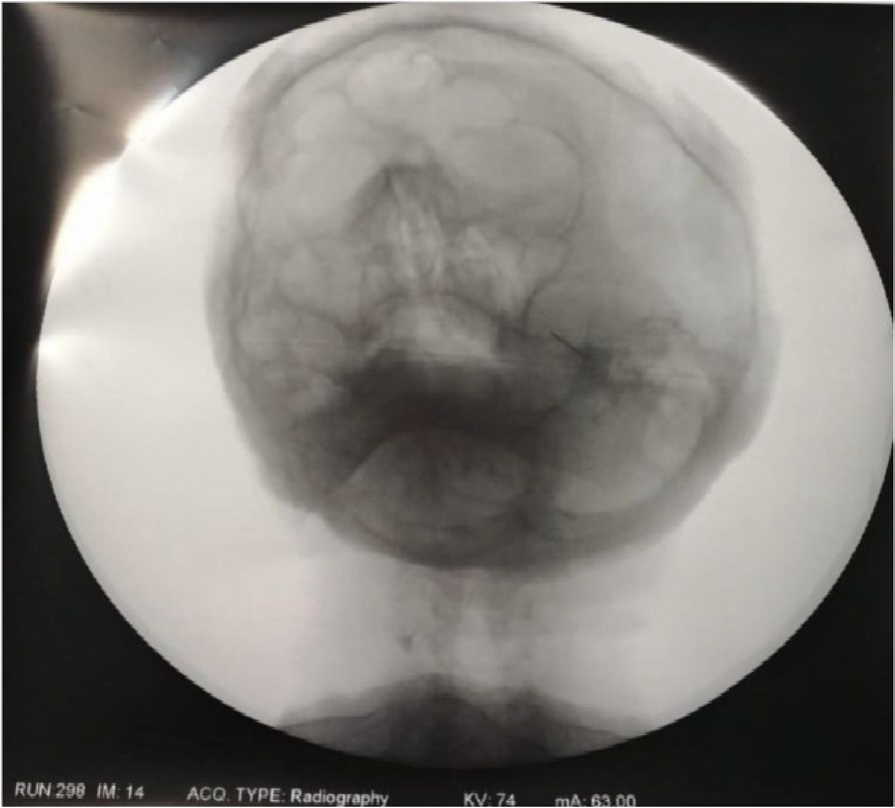
See text for description

**Fig. 3 (abstract A155).**
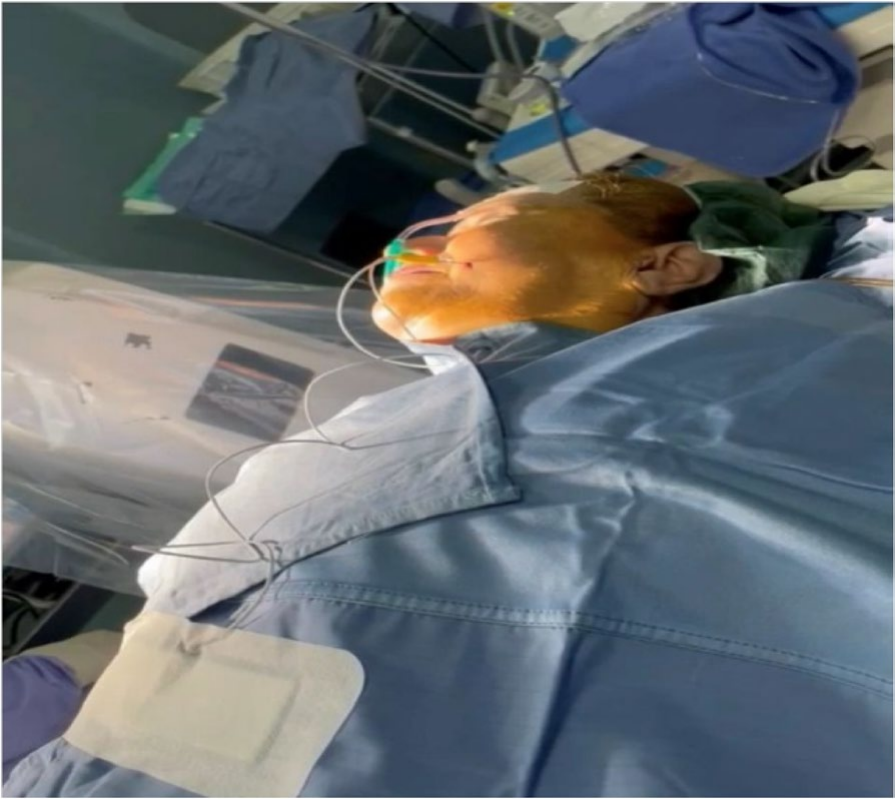
See text for description

### A156 Ultrasound-guided peripheral venous cannulation, a new real-time and dynamic approach

#### I. russo ^1^, M. vargas ^2^, A. marra ^2^, D. cirillo ^2^, I. piccione ^2^, M.S. barone ^2^, A. d'abrunzo ^2^, A.U. de siena ^2^, M. ianniello ^2^, A. coviello ^2^

##### ^1^ Department of Orthopedics and Traumatology, A.O.U. Policlinico Federico II, Naples, Italy, Naples, Italy; ^2^ Department of Anesthesia and Resuscitation, A.O.U. Policlinico Federico II, Naples, Italy, Naples, Italy

###### **Correspondence:** I. russo

*Journal of Anesthesia, Analgesia and Critical Care 2023,* **3(Suppl 1):**A156

Background: Difficult Intra-Venous Access (DIVA) is defined as a clinical condition in which the catheter cannot be inserted into the vein in one attempt but multiple attempts are planned or necessary to reach and maintain peripheral venous access [1,2]. Guidelines recommend the use of ultrasound to search for and puncture the vesse [3,4]. Two techniques for cannulating the vessel are described: the one with only ultrasound-guided puncture and the ultrasound-guided one. In the former, once the angio-catheter tip enters the venous lumen (short axis section out-plane approach), blind cannulation is performed. In the second, the progression of the cannula within the venous lumen is also done under ultrasound guidance then by rotating the probe 90° in-plane cannulation is performed [4,5].

Aim: Our study aims to compare techniques described in the literature versus a new technique used at our institution.

Materials and Methods: Between November 2022 and May 2023 at the Department of Orthopedics and Traumatology of the AOU Federico II in Naples all patients presenting with difficult venous access and signed informed consent were enrolled. Patients were randomized into three groups. First group (G1) peripheral venous access was placed with echo-guided puncture (short axis out-plane) and blind cannulation, in the second group (G2) peripheral venous access was placed with echo-guided puncture (short axis out-plane) and 'echo-guided cannulation (long axis in-plane approach) by rotating the probe 90°. In the third group (G3), peripheral venous access was placed with an echo-guided puncture (short axis out-plane) and cannulation (short axis out-plane) by advancing into the lumen of the vessel with the needle with the tip always under ultrasound vision.

(Figure 1). Procedures were performed by sanitarians experienced in the placement of echo-guided peripheral venous accesses.

Results: Sixty-two patients were recruited and randomly divided into three groups G1 (24 patients) and G2 (18 patients) and G3 (20 patients). The three groups were overlapping in terms of procedural difficulties present in history. Three experienced operators performed a similar number of procedures for each group. The number of first-attempt failures in peripheral venous access placement was 29.3 percent in the first group, 5.6 percent in the second group while no failure occurred in the third group (table 1).

Conclusions: The echo-guided cannulation technique has a lower incidence of failure on the first attempt than the blind technique. Echo-guided cannulation techniques require more manual dexterity. We believe that our technique is less difficult to perform.

References:

1. Sou V, McManus C, Mifflin N, Frost SA, Ale J, Alexandrou EA. Clinical pathway for the management of difficult venous access. BMC Nursing. 2017;16:1-7
2. Witting MD. IV access difficulty: Incidence and delays in an urban emergency department. Journal of Emergency Medicine. 2012;42(4):483-487
3. Van Loon FHJ, Buise MP, Claassen JJF, Dierick-van Daele ATM, Bouwman ARA. Comparison of ultrasound guidance with palpation and direct visualization for peripheral vein cannulation in adult patients: A systematic review and meta-analysis. British Journal Anaesthesia. 2018;121:358-366
4. Pittiruti M, Van Boxtel T, Scoppettuolo G, et al. European recommendations on the proper indication and use of peripheral venous access devices (the ERPIUP consensus): A WoCoVA project. The Journal of Vascular Access. 2023;24(1):165-182. doi:10.1177/11297298211023274
5. Yan-Bing Gao, Jun-Hong Yan, Jian-Min Ma, Xiao-Na Liu, Jing-Yun Dong, Fang Sun, Li-Wei Tang, Jie Li, effects of long axis in-plane vs short-axis out-of-plane techniques during ultrasound-guided vascular access, the American Journal of Emergency Medicine, Volume 34, Issue 5, 2016, pages 778-783, ISSN 0735-6757, https://doi.org/10.1016/j.ajem.2015.12.092

**Fig. 1 (abstract A156).**
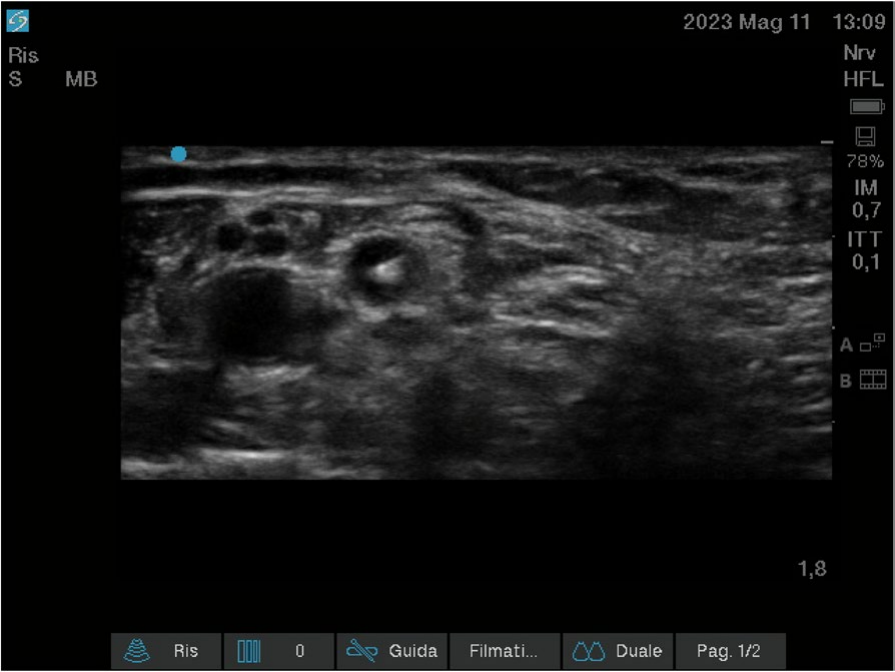
New technique

**Table 1 (abstract A156).**
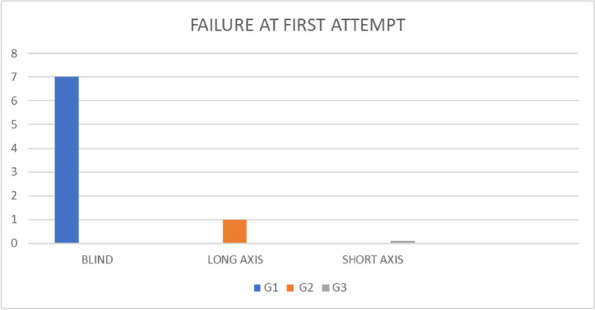
Failure at first attemp

### A157 Epidural blood patch in spontaneous intracranial hypotension, report of two cases

#### Roberta C, Alessandro F, Antonella F, Maria Vittoria D.A, Lucia I, Rosa maria Z, Donatella B

##### Ospedale Civile S. Spirito, Pescara, Italy

###### **Correspondence:** Roberta C

*Journal of Anesthesia, Analgesia and Critical Care 2023,* **3(Suppl 1):**A157

Background

Spontaneous intracranial hypotension (SIH) is a subset of intracranial hypotension and a known cause of headache. It might be caused by cerebrospinal fluid (CSF) leakage and CSF pressure reduction caused by orthostatic position.

SIH might be associated with hearing and sight disorder (due to cranic nerves impairment), back pain and nausea [1].

CSF leakage might be caused by dural weakness (congenital or contrived), ranging from dural tears to meningeal diverticula. Diagnosis is based on the clinical history of the patient, brain and spinal MRI with contrast enhancement, CT/MR myelography and radio-isotope cisternography. In the majority of cases, the leakage is located in the cervical or thoracic tract of the lumbar column, though the exact location of the leak might remain undetermined [2].

Although evidence is anecdotal, first line treatment in conservative, consisting in strict bed rest, hydratation, administration of analgesics, caffeine and theophylline. Steroid use remains controversial. In case of standard care failure, an Epidural blood Patch (EBP) might be indicated to seal off the dural strain.

Cases report

In our Centre, we have performed an EBP in a 47 years-old caucasian woman suffering from orthostatic headache, and a 49 years-old caucasian woman, suffering from orthostatic headache, nausea, tinnitus and lower limb paresthesia. In both patients, cerebral CT-scans and enhanced MRI-scans detected a bilateral fronto-parietal hygroma, and each woman underwent farmacological and conservative treatment, with poor results. No spinal puncture or trauma were reported in medical history.

EBPs were performed in a sterile enviroment, with multiparametric monitoring and a valid intravenous access. The patients were positioned in lateral decubitus. With ultrasound assistance, T11-T12 space was detected, where 20mL of autologous venous blood priorly collected were injected through a 16G thuoy needle. The recumbent position was maintened for 12 hours, while 1000mL of saline were administered. No complications were registered, and a complete recovery, clinical and instrumental (CT/MRI), was reported in both cases.

Conclusions

Epidural blood patch seems a valid and safe therapeutic instrument for treatment of SIH-related orthostatic headache refractory to conservative treatment. Larger studies are needed to completely assess safety and efficacy.

Informed consent to publish had been obtained

References

1. Shin HY. Recent update on epidural blood patch. Anesth Pain Med. 2021;pISSN: 1975-5171
2. Lin JP, Zhang SD, He FF, Liu MJ, Ma XX. The status of diagnosis and treatment to intracranial hypotension, including SIH. J Headache Pain. 2017; 18(1): 4

### A158 COVID-19 ARDS treated with ECMO in peripartum patients: a systematic review

#### L. Muscarà ^1^, S. Palella ^1^, L. La Via ^2^, F. Sanfilippo ^2^

##### ^1^ School of Anaesthesia and Intensive Care, Magna Graecia University, Catanzaro, Italy; ^2^ Department of Anaesthesia and Intensive Care, University Hospital G. Rodolico, Catania, Italy

###### **Correspondence:** L. La Via

*Journal of Anesthesia, Analgesia and Critical Care 2023,* **3(Suppl 1):**A158

Introduction

BACKGROUND: Coronavirus disease 19 (Covid-19) has been one of the most common causes of acute respiratory distress syndrome (ARDS), being responsible for a pandemic with almost 7 million deaths certified. Severe forms of ARDS can be supported with veno-venous extracorporeal membrane oxygenation (VV-ECMO). A specific population where the use of VV-ECMO for Covid-19 ARDS has been repeatedly reported is in young women in the peripartum period.

Objectives

OBJECTIVES: We aimed at performing a systematic review to summarize the results of the current literature on VV-ECMO in peripartum women with Covid-19 ARDS.

Methods

METHODS: We performed web-based search on Pubmed (last update 31.03.2023) to identify relevant articles; the protocol was regularly registered on PROSPERO. We included only articles published in English. Data were inserted independently by two authors (LM, SP) in an Excel database, and cross-checked by two authors (LLV, FS). We primarily focused on maternal and fetal mortality, recording also maternal and gestational age, as well as values of PaO2/FiO2 (P/F) ratio at cannulation, VV-ECMO duration and intensive care unit (ICU) and hospital length of stay (LOS).

Results

RESULTS: Our systematic search retrieved 131 items on PubMed. As reported in Table 1, we finally included 11 studies, including 8 retrospective studies (5 of them being single-center studies, 2 multi-center studies and 1 cohort study), 2 prospective studies (1 of them being single center study and 1 multicenter study), 1 prospective and retrospective cohort study, with a total of 191 patients. Among them, 151 were prepartum and 40 postpartum women (mean age 31 years). Average P/F ratio at cannulation was 72 with a mean duration of VV-ECMO of 22 days. Only 4 studies reported data on ICU and 3 on hospital LOS, with a mean of 43 and 63 days, respectively. The average maternal and fetal mortality were 17.8% (30/168, n=10 studies) and 20.7% (12/58, n=8 studies), respectively. Notably, the largest study did not report data on fetal mortality.

Conclusions

CONCLUSIONS: In our systematic review of case series and studies reporting data from pregnant women supported by VV-ECMO for severe ARDS due to COVID-19, we found that maternal and fetal survival stands around 80%. Such results seem rather good considering these were obtained during previously unexperienced pandemic conditions.

**Table 1. (abstract A158).** See text for description.

Case series	Type of study	Cases	Maternal Age (years)	Prepartum	Postpartum	Pa/FiO2 ratio (mmHg)	ECMO days	Type of ECMO	Lenght of ICU stay (days)	Hospital Length of stay (days)	Maternal mortality	Fetal Mortality
**Kovacevic et al.**	*Retrospective single center*	4	-	4	0	-	-	VV	-	-	3/4	0/4
**Kakar et al.**	*Case series*	5	33	1	4	66,2	59	VV	83	128	5/5	2/5
**Pejù et al.**	*Retrospective multicenter*	15	-	4	11	-	-	VV	-	-	-	-
**Bamasood et al.**	*Prospective single center*	8	28,3	4	4	79,3	22	VV	-	-	0/8	0/8
**Sitter et al.**	*Prospective multicenter*	15	34	11	4	60	25	VV*	38	-	3/15 (6 unknown)	3/15 (1 unknown)
**Yin et al.**	*Case series*	5	33	5	-	94	11	VV	26	30	1/5 (1 unknown)	1/5 (1 unknown)
**Shih et al.**	*Case series*	10	30	5	5	60,5	22	VV	28	31,5	2/10	4/10
**O’Neil et al.**	*Retrospective cohort study*	100	32,4	100		68	16,5	VV	-	-	16/100	-
**Barrantes et al.**	*Case series*	9	30	4	5	62	10	VV	-	-	0/9 (1 unknown)	1/9 (1 unknown)
**Piwowarczyk et al.**	*Retrospective single center*	5	33,2	1	4	93	11	VV	-	-	3/5	1/5
**Fatnic et al.**	*Prospective and Retrospective cohort study*	15	-	12	3	-	-	-	-	-	2/15	-
**Results**		191	31,7	150	40	72,8	22	VV	43,7	63	35/176	12/61

*One case of VA-ECMO

### A159 New block from an old approach for disc pain caused by median erniation

#### P.P. Murdaca ^1^, G. Di Gregorio ^1^, G. Battagin ^1^, L. Frigo ^1^, N. Cucci ^2^, F. Aloi ^2^, M.S. Capria ^2^

##### ^1^ Department of anesthesia, intensive care and pain therapy hospital of Cittadella aulss 6 euganea, Padua, Italy; ^2^ University of Medicine and Surgery of Padua, Padua, Italy

###### **Correspondence:** P.P. Murdaca

*Journal of Anesthesia, Analgesia and Critical Care 2023,* **3(Suppl 1):**A159

The interlaminar, (figure 1) or transforaminal (figure 2) cortisone epidural plays a fundamental role in treatment of lumbosciatica and cervicobrachialgia with predominantly radicular involvement, where the disc impingement mainly affects the foramen or the preforaminal region with an anti-inflammatory neurogenic and anti-edema effect partly also on the still hydrated extruded disc component.

In case of pain median disc herniation the pain symptoms transported by the sinuvertebral nerve remain in largely uncontrolled by a classic injection because the pathogenic noxa is anterior and does not get wet, or marginally, from the anesthetic-cortisone mixture injected posteriorly, i.e. spatially diametrically opposite, in the interlaminar epidural, or periradicular, in the transforaminal (figure 3).

On the other hand, a frankly expelled hernia, with anulus fibrosus irreparably torn and/or black disc, reduced considerably large, chemical or thermal intradiscal ablation/annuloplasty treatments are ineffective on the

nucleus pulposus, as well as surgical ones, in the absence of frank motor-sensory deficits.

In this case a deep paravertebral infiltration in a similar approach could be advantageous

for disc pain, through the safety kambin triangle, placing the tip of the needle on the posterolateral aspect of the vertebral body or disc (figure 4.5).

With this approach, the mixture is poured along the cortical periosteum of the soma or the annulus fibrosus, penetrating inside the vertebral specus anterior, through the conjugation foramen, between two contiguous vertebrae, so as to imbibe the extruded hernia median, dehydrating it and at the same time anesthetizing the spinal nerves, the gray communicating branch and the segmental ganglion of the anterior-lateral chain of the sympathetic system, responsible to central-axial disc back pain jointly. We called deep paravertebral Retrodiscal block

CASE REPORT

36-year-old patient with known disc protrusions all over the lumbar level develops suddenly after one incongruous load a central lumbar pain (more towards the right) radiating to the sacrum, in part, and only occasionally on the thighs, anteriorly, without sensory-motor deficits or involvement of the cauda equina or alteration of the osteotendon reflexes. On magnetic resonance, appearance of central expelled hernia L3-L4 with reduction of corresponding vertebral specus of about 50% (figure 6,7).

Pain continuous worsened by load and by prolonged standing position. After ineffective intramuscular cortisone therapy, he undergoes an epidural median interlaminar cortisone L3-L4 with the same result. So after a week he undergoes a paravertebral prediscal block. It was performed in the left lateral position, in in-plane ultrasound guidance targeting the posterolateral aspect of the L3 vertebral body and L3-L4 disc (figure 8, 9,10). Immediate benefit with approximately 50% pain relief

INFORMED CONSENT TO PUBLISH HAD BEEN OBTAINED

The suitably informed patient has given his free consent to publish the aforementioned abstract.

**Fig. 1 (abstract A159).**
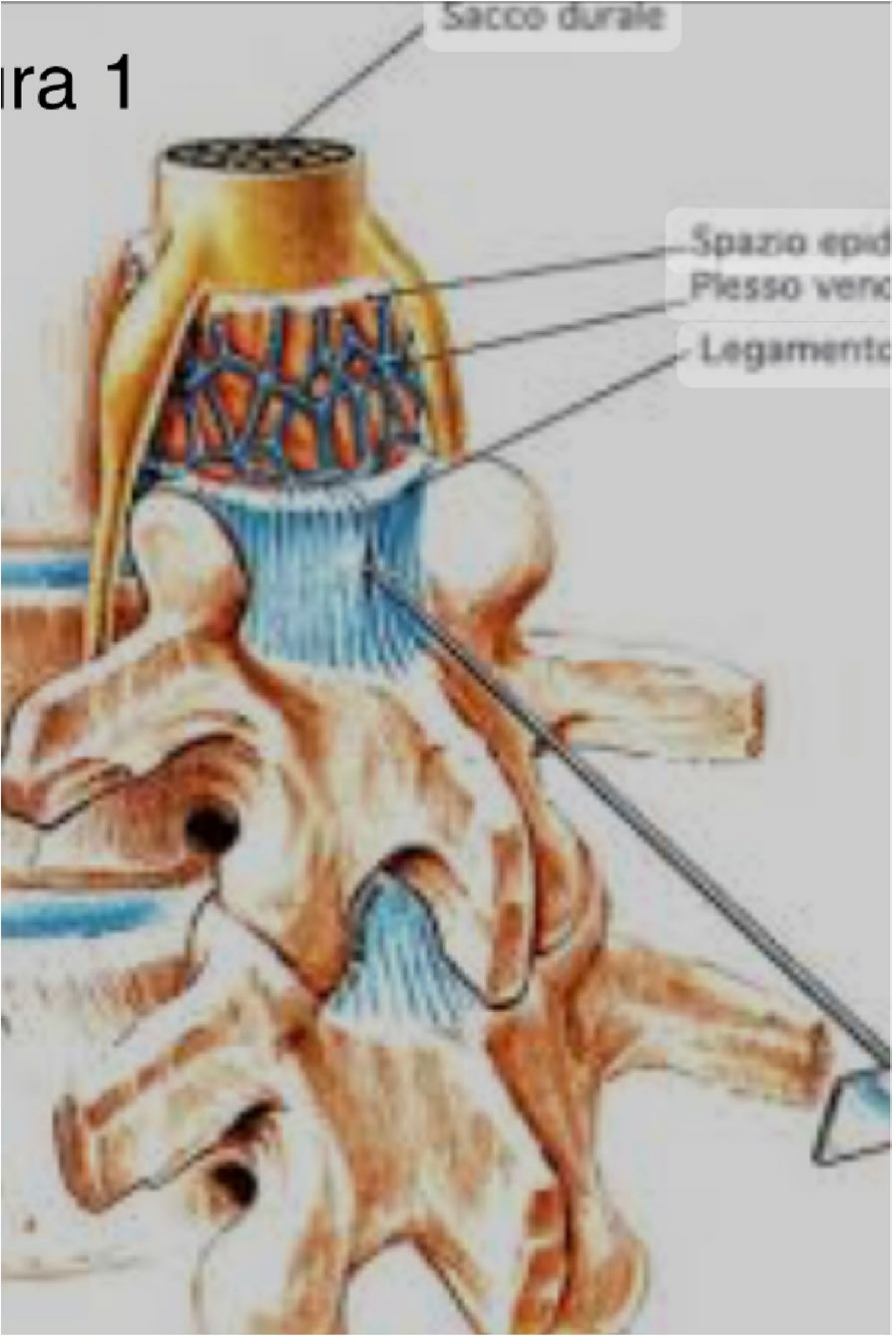
See text for description

**Fig. 2 (abstract A159).**
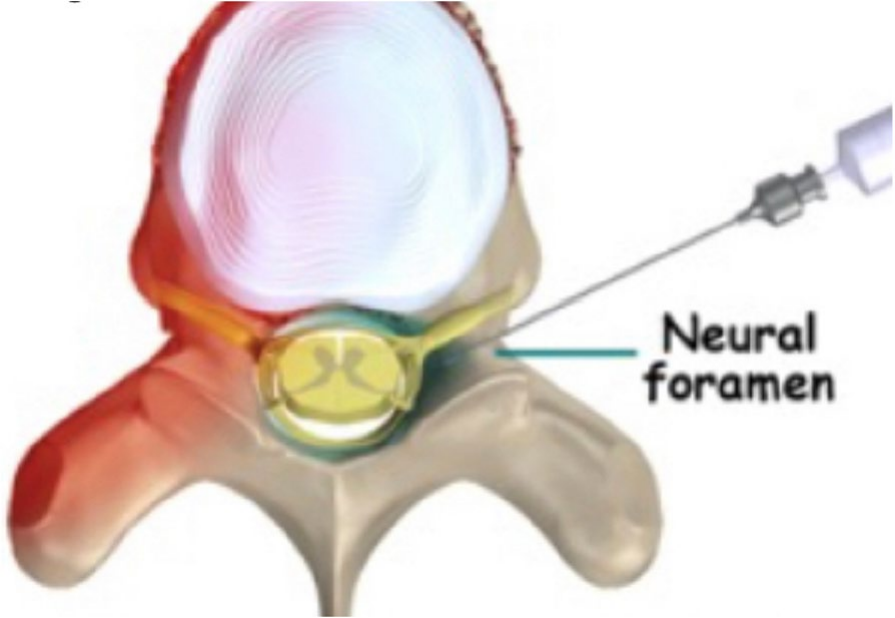
See text for description

**Fig. 3 (abstract A159).**
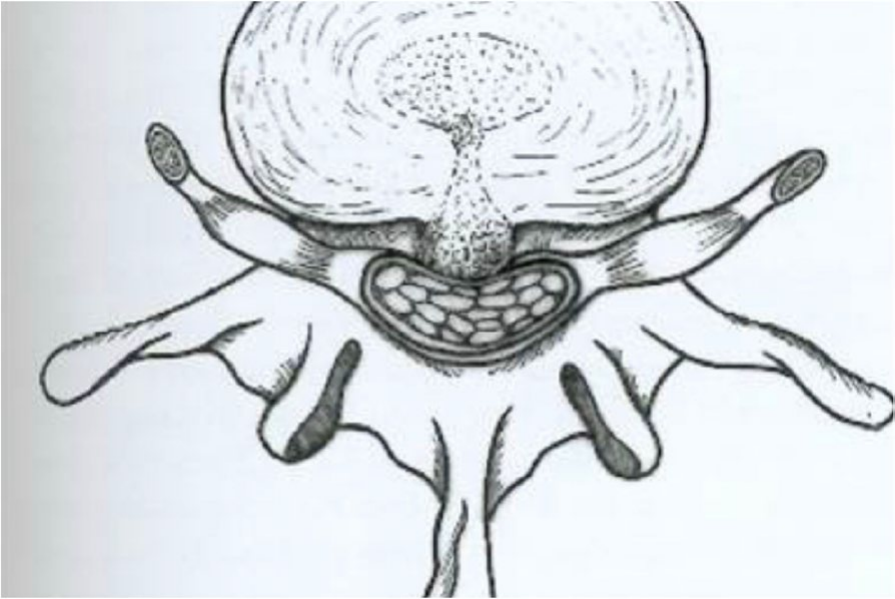
See text for description

**Fig. 4 (abstract A159).**
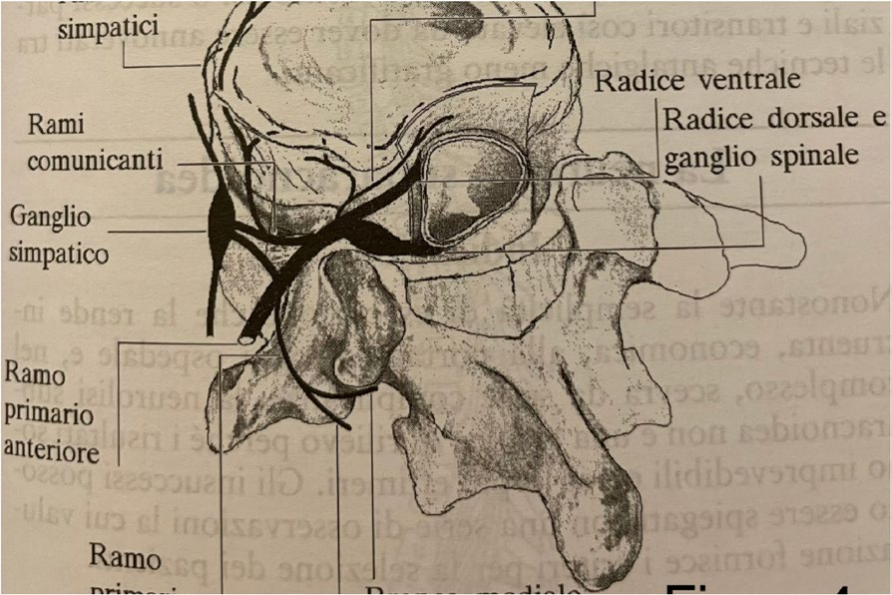
See text for description

**Fig. 5 (abstract A159).**
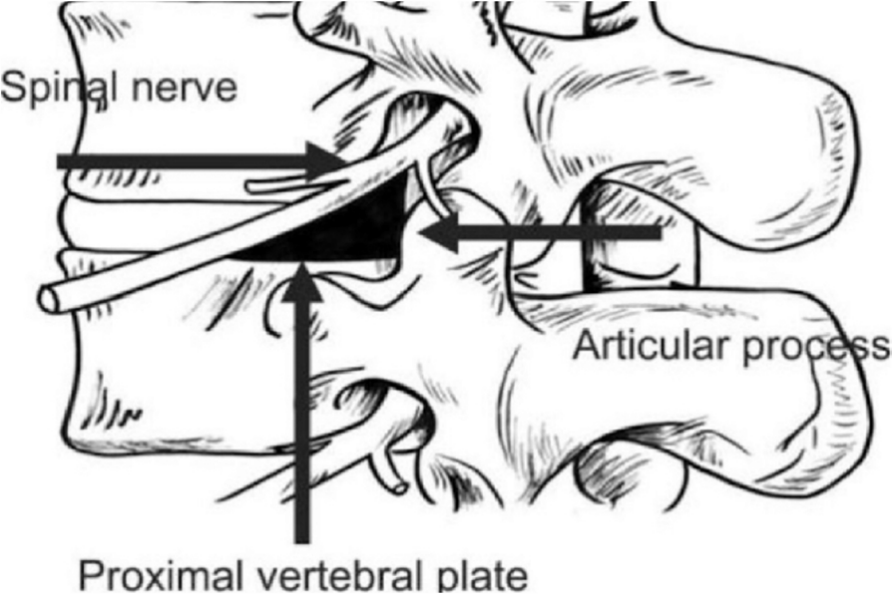
See text for description

**Fig. 6 (abstract A159).**
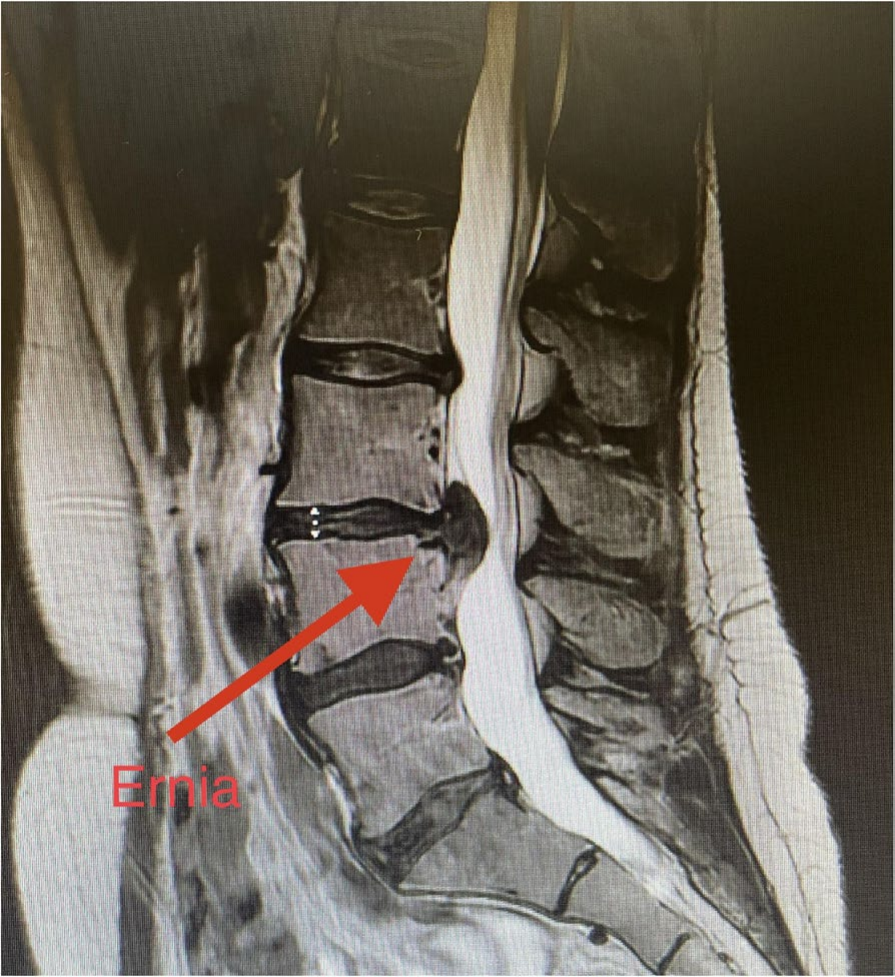
See text for description

**Fig. 7 (abstract A159).**
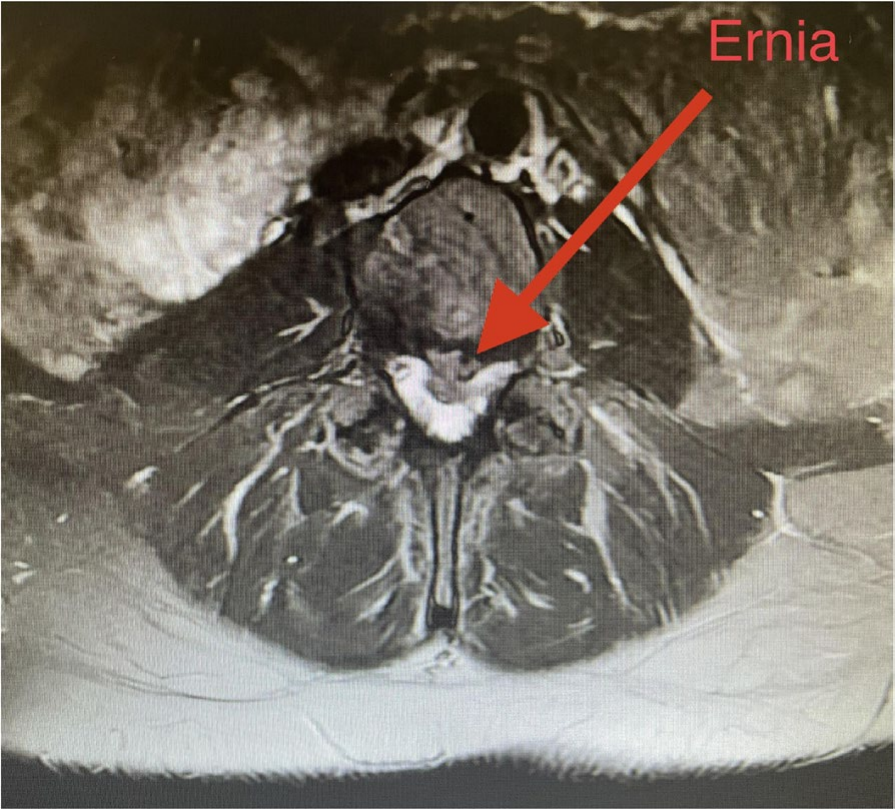
See text for description

**Fig. 8 (abstract A159).**
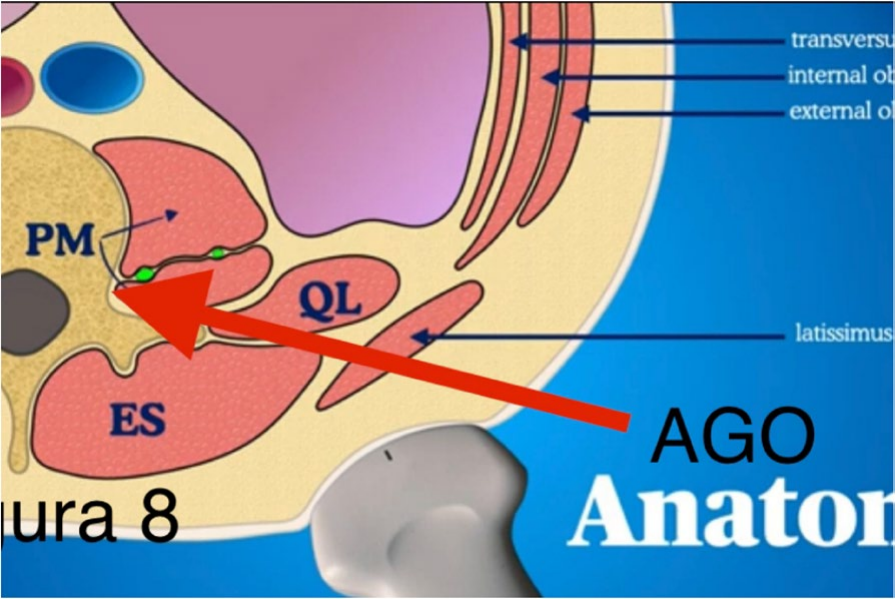
See text for description

**Fig. 9 (abstract A159).**
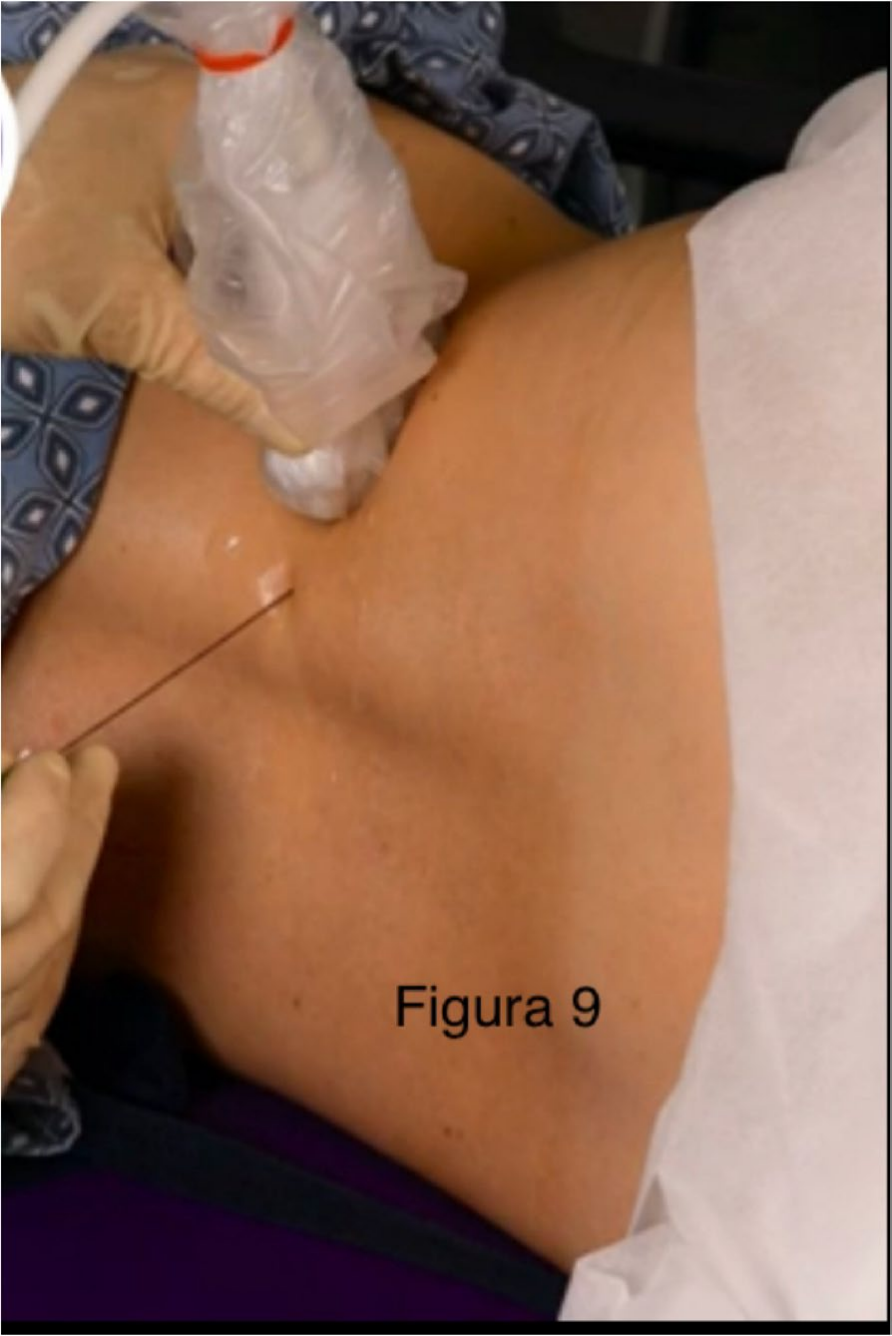
See text for description

**Fig. 10 (abstract A159).**
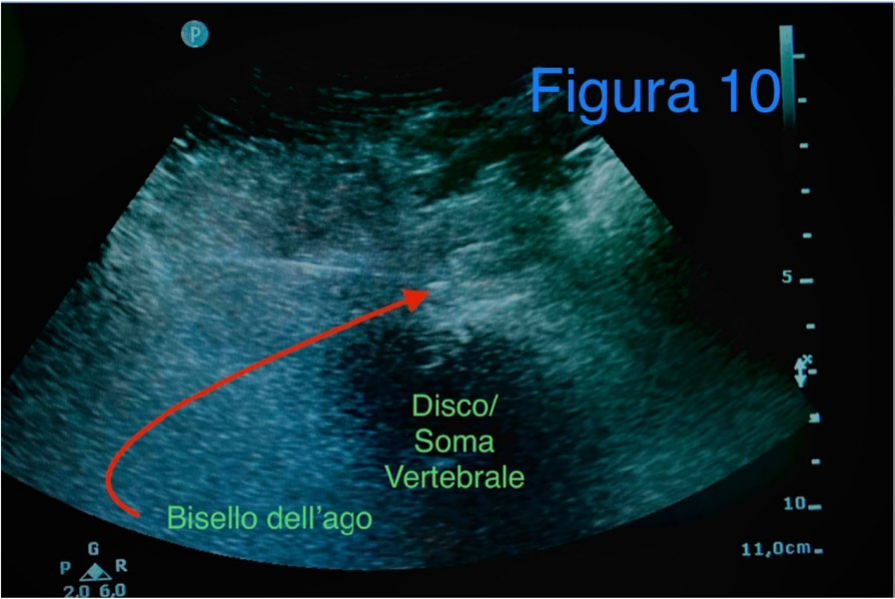
See text for description

### A160 Optimal sedation for treatments on the trigeminal gasser ganglion nerve: the role of dexdor

#### P.P. Murdaca ^1^, G. Di Gregorio ^1^, G. Battagin ^1^, L. Frigo ^1^, M.S. Capria ^2^, N. Cucci ^2^, F. Aloi ^2^

##### ^1^ Department of anesthesia, intensive care and pain therapy hospital of Cittadella aulss 6 euganea, Padua, Italy; ^2^ university of medicine and surgery of Padova, Padova, Italy

###### **Correspondence:** P.P. Murdaca

*Journal of Anesthesia, Analgesia and Critical Care 2023,* **3(Suppl 1):**A160

Pulsed radiofrequency of the intracranial post ganglionic branch of Gasser with transforaminal approach (oval foramen of the middle cranial fossa) represents a validated and effective technique in the treatment of trigeminal neuralgia, however it is painful because it stimulates the nociceptors of the dura mater and, penetrating inside of the branches, the sensory neurons that reside in the trigeminal ganglia. So there are two moments in which sedation-analgesia is fundamental: the positioning of the intracranial needle and the pulsed radiofrequency treatment. Sedation in this case must be:

- deep, in order to avoid hypertensive crises, the cause of the major complication which is cerebral haemorrhage;
- brief to avoid airway management maneuvers that could contaminate the operative field (cheek) or the needle, pushing it into the oral cavity or into sensitive intracranial structures (carotid, pons, medulla, temporal lobe, other cranial nerves) with catastrophic and definitive damage;
- quickly reversible, to allow the patient, once re-emerged from sedation, full collaboration, to verify the correct positioning of the needle tip with neurophysiological tests.

DEXMETHOMIDINE AND ITS TARGET

Dexmedetomidine is a selective alpha-2 adrenergic receptor agonist with a broad range of pharmacological properties. It has a sympatholytic effect through inhibition of norepinephrine release in sympathetic nerve endings. The sedative effects are mediated by the decrease in the discharge activity of the locus coeruleus, the predominant noradrenergic nucleus which is located in the brainstem (figure 1)

TRIGEMINAL NERVE NETWORK

Sensory signals are known to support the background firing of the ascending reticular activating system (ARAS) which includes neurons of the noradrenergic locus coeruleus (LC) and controls the level of attention and alertness. Among the inputs that can drive LC and ARAS neurons, those coming from the trigeminal region seem to be particularly relevant in regulating their activity (figure 2).

SEDATION PROTOCOLS

standard protocol

Acetaminophen+clonidine+opiate+magnesium sulphate+propofol;

dexdor tiva protocol

Paracetamol+opiate+magnesium sulphate+propofol+dexdor tiva 1 gamma/kg/h ;

Dexdor cet dyck protocol

Paracetamol + opiate + magnesium sulphate + propofol + dexdor cet 0.5 ng/ml;

TARGET

Pas<140mmHg and pad<80mmHg

NRS<3

above these ranges propofol and/or dexdor boluses are given

CASE SERIES

Up to now, one case has been treated with the classic dexdor continuous infusion 1 gamma/kg/h and one with the Dyck model cet 0.5 mcg/mL. In both cases no clonidine was used;

in the case of dexdor tiva boluses of both dexdor and propofol were required but with halved dosages compared to the standard treatment;

in the dexdor tci case, neither diprivan nor dexdor boluses were used.

HYPOTHESIS

The locus ceruleus has close connections with the trigeminal nerve and represents the major objective of dexdetomedima, which, perhaps also for this reason, could offer greater respiratory and hemodynamic stability compared to classic sedation, in pulsed radiofrequency treatments of the fifth cranial nerve.

INFORMED CONSENT TO PUBLISH HAD BEEN OBTAINED

Adequately informed patients have given their free consent to publish the above abstract

**Fig. 1 (abstract A160).**
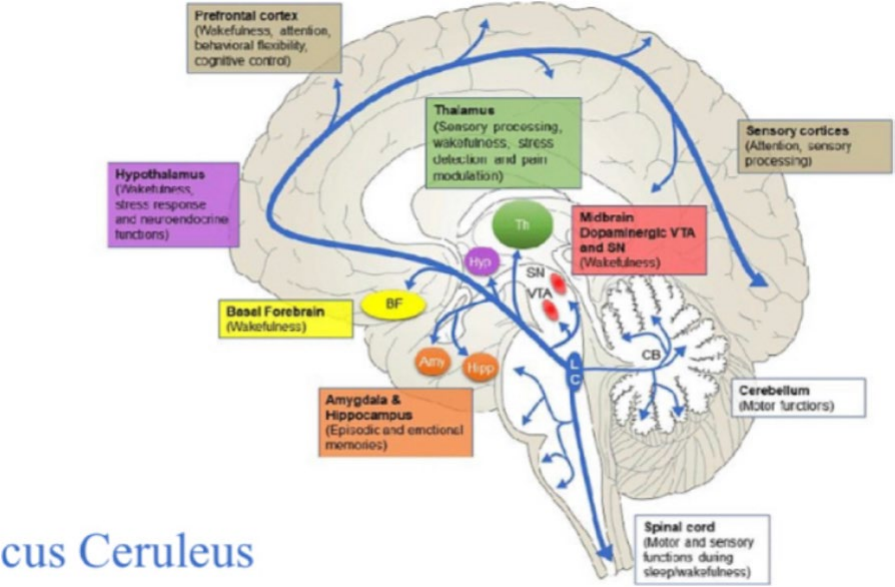
See text for description

**Fig. 2 (abstract A160).**
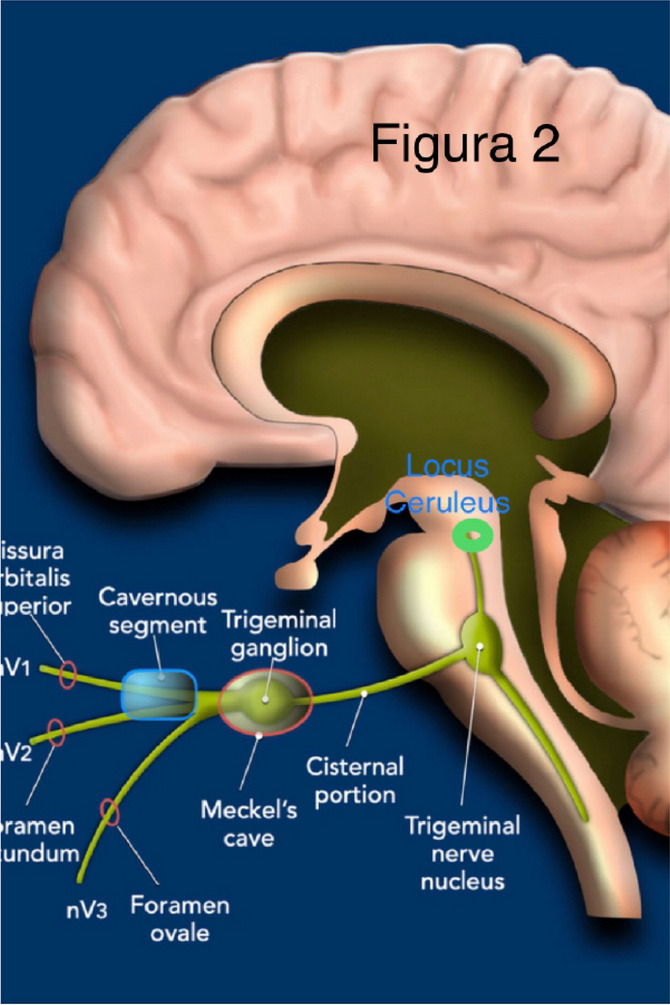
See text for description

**Fig. 3 (abstract A160).**
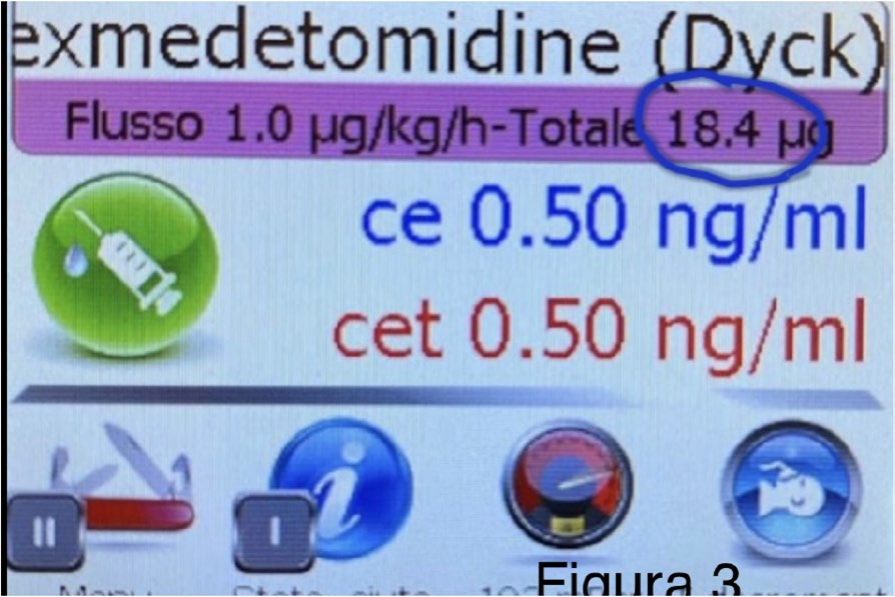
See text for description

### A161 Why the trigeminal Ganglion Meckel Cave isn't perforated in two patients already subjected to

#### P.P. Murdaca ^1^, G. Di Gregorio ^1^, G. Battagin ^1^, L. Frigo ^1^, F. Aloi ^2^, M. Capria ^2^, N. Cucci ^2^

##### ^1^ Department of anesthesia, intensive care and pain therapy hospital of Cittadella aulss 6 euganea, Padua, Italy, ^2^ universities of Padua, Medicine and Surgery, Padua, Italy

###### **Correspondence:** P.P. Murdaca

*Journal of Anesthesia, Analgesia and Critical Care 2023,* **3(Suppl 1):**A161

Trigeminal neuralgia (TN) is a neuropathic pain involving the fifth cranial nerve and is manifested by intense and paroxysmal pain crises similar to electric shocks lasting from a few seconds to two minutes.

Conventional therapy for this pathology is drug treatment with anticonvulsants such as carbamazepine or oxcarbazepine.

When drug therapy has been suspended for various reasons, the solution is to resort to alternative surgical procedures. The most commonly used therapies are:

- Percutaneous retrogasserian radiofrequency
- Percutaneous compression of Gasser's ganglion ('with Fogarty balloon')
- Radiosurgery with gamma knife
- Microvascular decompression.

RADIOFREQUENCY IN TRIGEMINAL NEURALGIA

The root is subjected to a pulsed radiofrequency (PRF), and consists of a neuromodulation and its objective are the post gasserian branches

Conventional/continuous radiofrequency (CRF) produces high temperatures to selectively destroy nerves and targets the pre-Gasser roots near the clivus at the apex of the temporal pyramid.

CASE SERIES AND HYPOTHESIS

The reasons for the benefit or failure in cases in which the patient has already undergone open surgical procedures and even neurolesive techniques are still unclear and unknown. In these two cases, already unsuccessfully subjected to compression with the Gasser balloon (we do not have the duration and pressure of the inflation nor the number of cycles), we noticed that the patients did not benefit from the pulsed radiofrequency but they successfully underwent the thermal thermorhizotomy, however it was not possible to cannulate Meckel's cavity with classic CSF reflux, which is usually a mandatory finding to reach the preganglionic root and trigeminal porus (see Subarachnoid trigeminal meckelography/cisternography with subtentorial cerebellar contrast medium spillage figure 1.2)

Compression with the balloon acts indiscriminately on all branches and probably due to an ischemic effect, destroying the myelin fibers and therefore reducing the tactile sensitivity and the excitability of the trigger points more than the pain sensitivity and therefore has a low incidence of sensory deficits. However, it could induce a crushing of Meckel's cave such as to cause a neuroinflammatory reaction of the meningeal sheets, at least in these two cases, with obliteration of the well-known dural sac containing cerebrospinal fluid and maintain or induce new neuralgia support circuits, with subsequent development of reactions fibrotic similar to the failed back surgery syndrome (Diffusion of the contrast medium in the infra-temporal and probably epidural peripontina region in the absence of opacification of Meckel's cavity and trigeminal cistern; figure 3).

This could explain the ineffectiveness of ganglion pulsed radiofrequency and the need to resort to retroganglionic thermorhizotomy.

INFORMED CONSENT TO PUBLISH HAD BEEN OBTAINED

Adequately informed patients have given their free consent to publish the above abstract

**Fig. 1 (abstract A161).**
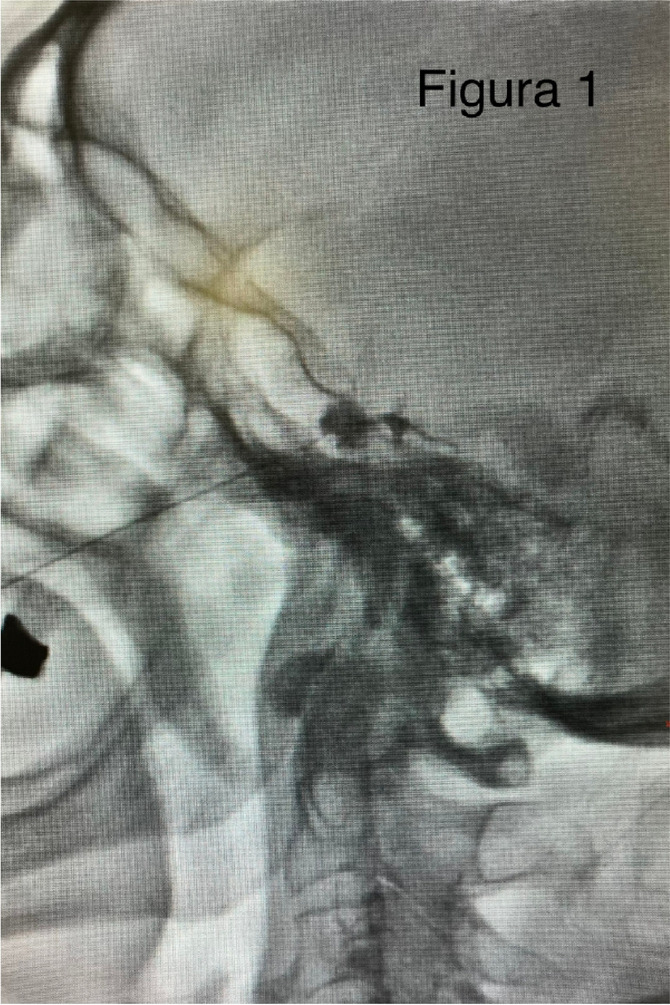
See text for description

**Fig. 2 (abstract A161).**
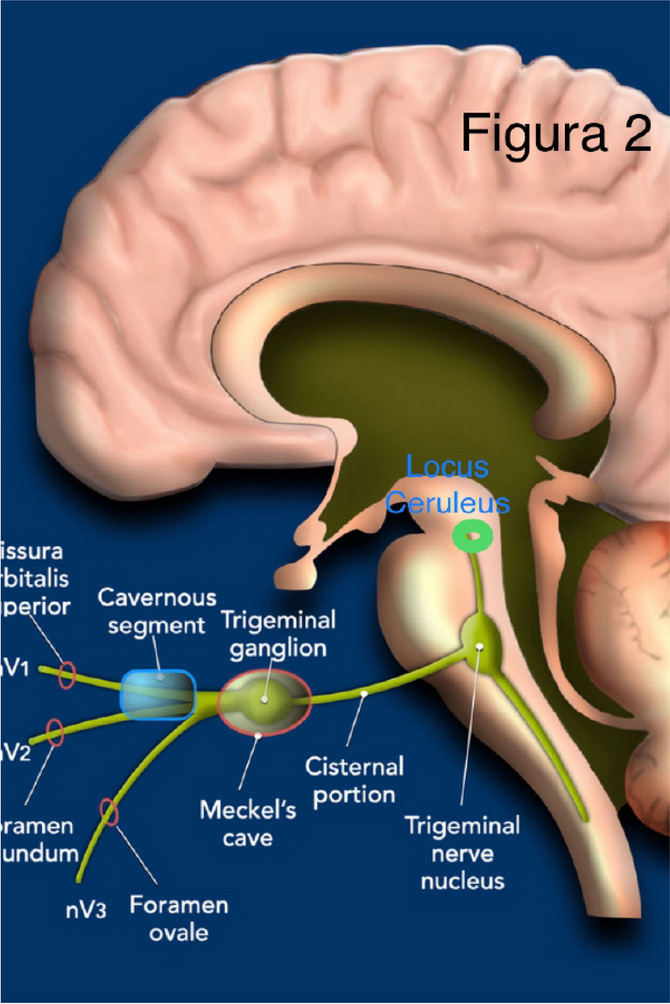
See text for description

**Fig. 3 (abstract A161).**
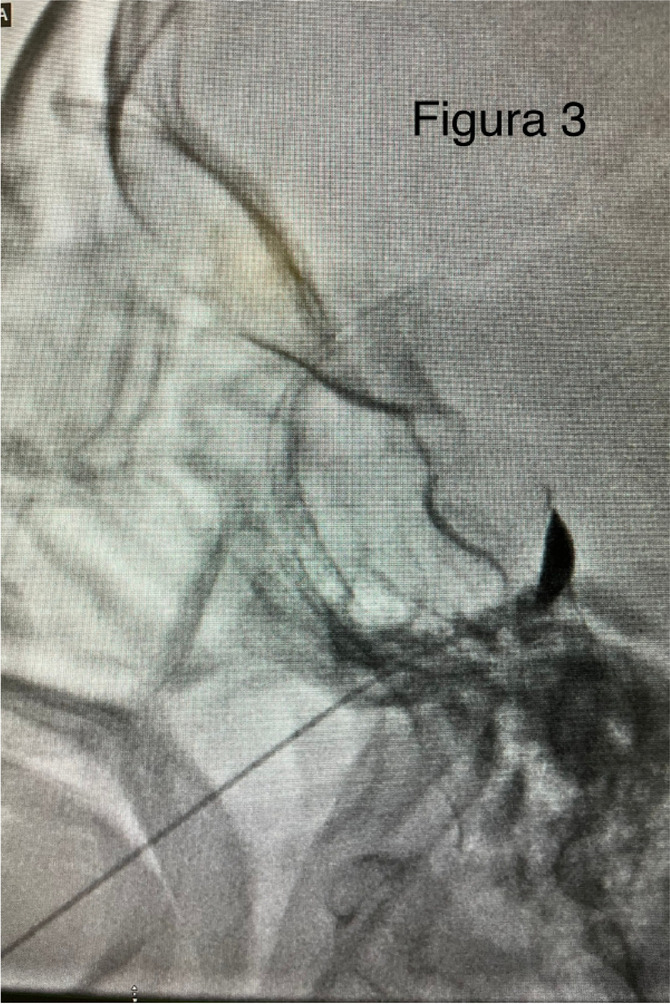
See text for description

### A162 A technical modification for percutaneous dilatational tracheostomy: our experience

#### S. Pilloni, A. Busia, E. Lai, M. Muceli, G. Olla, A. Orru', S. Paba, A. Paddeu, M.V. Piroddi, S. Serdino, A. Usai, F.M. Loddo

##### Ospedale nostra Signora Della Mercede - SC Anestesia E Rianimazione - ASL Ogliastra, Lanusei, Italy

###### **Correspondence:** S. Pilloni

*Journal of Anesthesia, Analgesia and Critical Care 2023,* **3(Suppl 1):**A162

Background: tracheostomy is one of the oldest and most frequently performed procedures on intensive care unit patients.1 In our experience, the most common indication for percutaneous tracheostomy was need for prolonged ventilatory support and difficult weaning. The percutaneous dilatational tracheostomy (PDT) method used in our ICU combines technical principles common to the Percu-Twist and the Griggs-Portex techniques.

Materials and methods: we performed 23 PDTs using a modified Seldinger technique requiring two operators, a Percu-Twist kit, Griggs forceps and continuous bronchoscopic guidance. This approach aimed to prepare the soft tissue to the twisting maneuver, thus reducing the already fewer bleeding events of the percutaneous dilatational technique when compared to surgical tracheostomy.2

Results: we examined all patients subjected to this technique from 2020 to 2022. The total number of patients examined was 23. We followed the patients for 15±4 days after the procedure, analyzing each individual case to check for any complications. None of this patients developed any complications in the observation period.

Conclusions: in our experience, the modified PDT approach can be considered a safe and practical alternative to surgical tracheostomy. The use of routine bronchoscopic guidance for the full procedure, although requiring two operators and thus not being as resource-sparing as other alternatives in current literature3, grants a larger safety profile allowing the early detection of the most common complications, such as tube misplacement, damage to the posterior wall of the trachea and bleeding.

References

1. Joao B Rezende-Neto et al. A technical modification for percutaneous tracheostomy: prospective case series study on one hundred patients. World Journal of Emergency Surgery 2011, 6:35.
2. Omar Naushad et al. Naushad’s Modification of Griggs Percutaneous Tracheostomy: Retrospective Case Series Study on 200 Patients at Subharti Medical College, Meerut, India. MAEDICA – a Journal of Clinical Medicine 2022; 17(1): 64-73.
3. Yashvir Singh Sangwan, Robert Chasse. A modified technique for percutaneous dilatational tracheostomy: A retrospective review of 60 cases, Journal of Critical Care, Volume 31, Issue 1, 2016, Pages 144-149.

### A163 Modified venipuncture technique for insertion of a non-tunnelized picc

#### D. Cirillo ^1^, C. Iacovazzo ^1^, P. Buonanno ^1^, C. D'Errico ^3^, I. Russo ^2^, I. Piccione ^1^, A. Izzo ^2^, L. Marasco ^2^, A. D'Abrunzo ^1^, A. Coviello ^1^

##### ^1^ Department of Neurosciences, Reproductive and Odontostomatological Sciences, University of Naples Federico II, Naples, Italy; ^2^ Department of Public Health, School of Medicine, University of Naples Federico II, Unit of Orthopedics and Traumatology, Naples, Italy; ^3^ Department of Anesthesia and Intensive Care Unit, AORN Cardarelli, Naples, Italy

###### **Correspondence:** D. Cirillo

*Journal of Anesthesia, Analgesia and Critical Care 2023,* **3(Suppl 1):**A163

Background

Insertion of peripherally inserted central catheters (PICCs) is potentially related to the risk of immediate, early and late complications [1]. The highly performed traditional technique involves: ultrasound-guided out-of-plane venipuncture in the short axis of the chosen arm vein, cannulation maneuver using the indirect Seldinger technique, and venous catheter insertion [2].

In our study, we propose a modification of the standard puncture technique for the insertion of an non-tunnelized PICC, with the aim of minimizing the incidence of complications.

Materials and methods

Between November 2022 and April 2023, patients undergoing basilic vein PICC placement in Dawson's Green Zone [3] were enrolled at the Department of Orthopaedics and Traumatology of the AOU Federico II in Naples, Italy. Patients were randomized into two groups. In the first group (G1), the PICC was placed according to the traditional technique in compliance with SIP protocol [4]. In the second group (G2), the PICC was placed with a modified technique, which involved the needle advancement at least 0.5 cm within the vessel lumen with real-time viewing of the needle tip (short-axis section and out-of-plane approach), and insertion of the Seldinger with the needle tip in the center of the vessel lumen (Figure 1). The procedures were performed by experienced anesthesiologists in PICC placement.

Results

52 patients were enrolled, including 25 patients in the G1 group and 27 patients in the G2 group: the two groups overlapped in terms of procedural difficulties present in clinical history. The G1 group presented incidence of multiple punctures for failure to progress Seldinger's by 28%, pain at the puncture site by 16%, hematoma formation by 8% and deep venous thrombosis at 30 days by 8%.

Patients in group G2 never presented multiple puncture need for non-progression of Seldinger's, nor pain at the puncture site, hematoma formation of 3.7% and no incidence of deep vein thrombosis at 30 days (Figure 2).

Conclusions

The modified PICC insertion technique presented a significantly lower incidence of complications than the traditional technique. However, randomized clinical trials are needed to confirm the validity of these findings.

Consent to publish

Written informed consent was obtained from all patients.

References

1. Krein SL, Saint S, Trautner BW, Kuhn L, Colozzi J, Ratz D, Lescinskas E, Chopra V. Patient-reported complications related to peripherally inserted central catheters: a multicentre prospective cohort study. BMJ Qual Saf. 2019 Jul;28(7):574-581.
2. Lamperti M, Biasucci DG, Disma N, Pittiruti M, Breschan C, Vailati D, Subert M, Traškait? V, Macas A, Estebe JP, Fuzier R, Boselli E, Hopkins P. European Society of Anaesthesiology guidelines on peri-operative use of ultrasound-guided for vascular access (PERSEUS vascular access). Eur J Anaesthesiol. 2020 May;37(5):344-376.
3. Dawson RB. PICC zone insertion methodTM (ZIMTM): a systematic approach to determinate the site for PICCs in the upper arm. JAVA 2011;16:156-65.
4. Brescia F, Pittiruti M, Spencer TR, Dawson RB. The SIP protocol update: Eight strategies, incorporating Rapid Peripheral Vein Assessment (RaPeVA), to minimize complications associated with peripherally inserted central catheter insertion. J Vasc Access. 2022 May 27:11297298221099838.

**Fig. 1 (abstract A163).**
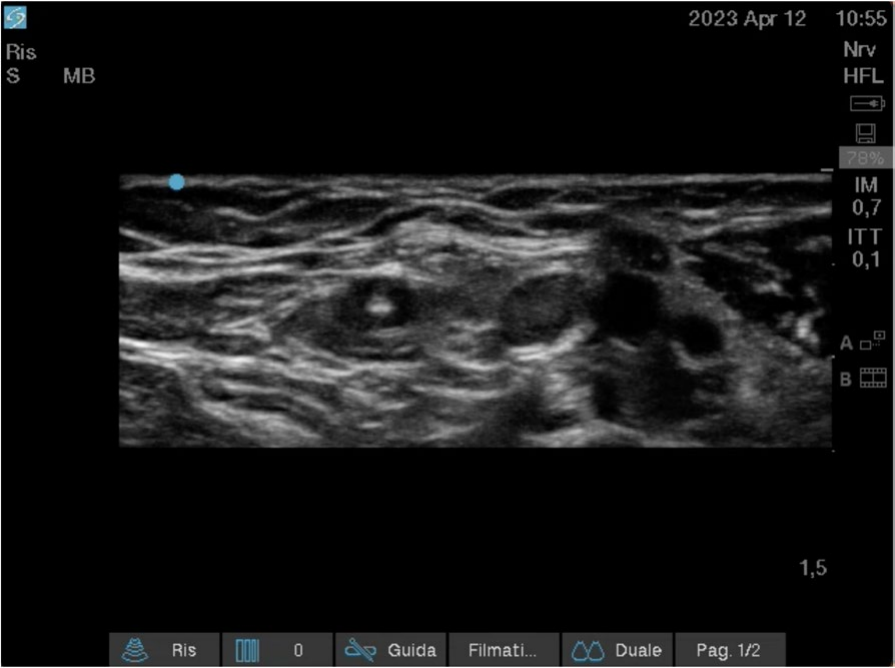
The needle tip in the center of the vessel lumen

**Fig. 2 (abstract A163).**
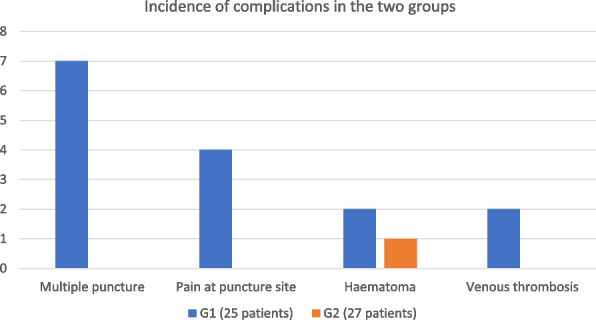
Incidence of complications in the two groups

### A164 Extracorporeal Membrane Oxygenation in hematological patient with MOF and Cyclophosphamide-Induced Heart Failure: a case report

#### C. Carmignan ^1^, N. D'Andrea ^2^, M. Maieron ^1^, A. Brussa ^2^, T. Bove ^1,2^

##### ^1^ Anesthesiology and Intensive Care Clinic, Department of Medicine, University of Udine, Udine, Italy; ^2^ Department of Anesthesia and Intensive Care, ASUFC Santa Maria della Misericordia University Hospital of Udine, Udine, Italy

###### **Correspondence:** C. Carmignan

*Journal of Anesthesia, Analgesia and Critical Care 2023,* **3(Suppl 1):**A164

Background: Chemotherapy-induced cardiac dysfunction is a well-known cause of morbidity and mortality. Particularly Cyclophosphamide may cause severe cardiac toxicity leading to hypotension, cardiomyopathy, tachyarrhythmias, heart failure, cardiogenic shock and consequently Multi Organ Failure (MOF) [1]. Veno-Arterial Extracorporeal Membrane Oxygenation (ECMO A-V) may be an option to support patients if cardiac dysfunction does not recover [2,3]. Few reports show successful weaning from ECMO in patients with hematological malignancies [3, 4]. Mortality is often associated with infections, hyperbilirubinemia and bleeding complications [2, 4].

Case report: A 60-year-old female was admitted in Intensive Care Unit from Hematological Department with clinical presentation of heart failure. Her recent medical history included a Stem Cell Transplantation (SCT) for aplastic anemia and cyclophosphamide administration as conditioning therapy. On 12th day post-transplantation, patient developed cardiogenic shock and severe MOF. Ultrasound assessment showed hypokinetic enlarged ventricles with a 30% ejection fraction (EF), severe tricuspid insufficiency, minimal pericardial effusion, pleural and perihepatic effusion. Clinical, laboratory and echographic evaluations were suggestive of acute cyclophosphamide cardiotoxicity with acute heart failure. In order to restore cardiac function ECMO A-V was implanted with initial mild hyperflow. During recovery, hemodynamics required low doses inotropic support and hemotransfusions. SVO2 values range between 68-85%. Atrial fibrillation was cardioverted. Progressively, acidosis, coagulation status, hemodynamics, liver and kidney disfunction improved, allowing the reduction of ECMO support until complete weaning. On the sixth day transthoracic and transesophageal ultrasound showed improvement of biventricular kinetics and absence of valvular defects (Figure 1).

ECMO A-V was then removed at the seventh day. Hemodynamics remained supported by levosimendan and low doses of adrenaline. Estimated EF was 70%. Three days after decannulation clinical condition were worsening, with temperature and enhanced PCR/PCT: blood stream infection was confirmed. Hyperbilirubinemia and BUN raised, hypotension and septic shock established. On 23th day after SCT the patient died.

Conclusion: ECMO A-V may give potential benefit in improvement clinical conditions and long term prognosis of hematological patients. Good patient selection is necessary. ECMO weaning success as bridge-to-recovery therapy must be pursued. Immunosuppression degree, hematological diagnosis, patient age and ICU admission cause may influence it. Reversible causes of cardiogenic shock (e.g. drug toxicity) may justify A-V ECMO use. In our case, despite extracorporeal support benefit, immunosuppression and bone marrow cyclophosphamide toxicity, with lasting granulocytopenia at 23th days after SCT, may have conditioned patient outcome.

Note: Informed consent to publish had been obtained.

References

1. Dhesi S, et al. Cyclophosphamide-Induced Cardiomyopathy: A Case Report, Review, and Recommendations for Management. J Investig Med High Impact Case Rep. 2013; January-March: 1–7
2. Kang HS, et al. Clinical outcomes of extracorporeal membrane oxygenation support in patients with hematologic malignancies. Korean J Intern Med 2015; 30:478-488
3. Cho S, et al. Extracorporeal Life Support in Adult Patients with Hematologic Malignancies and Acute Circulatory and/or Respiratory Failure. Korean J Thorac Cardiovasc Surg. 2019; 52:25-31
4. Potratz JC, et al. Extracorporeal Membrane Oxygenation in children with cancer or Hematopoietic Cell Transplantation: Single-Center Experience in 20 consecutive patients. Front Oncol. 2021; 11:664928

**Fig. 1 (abstract A164).**
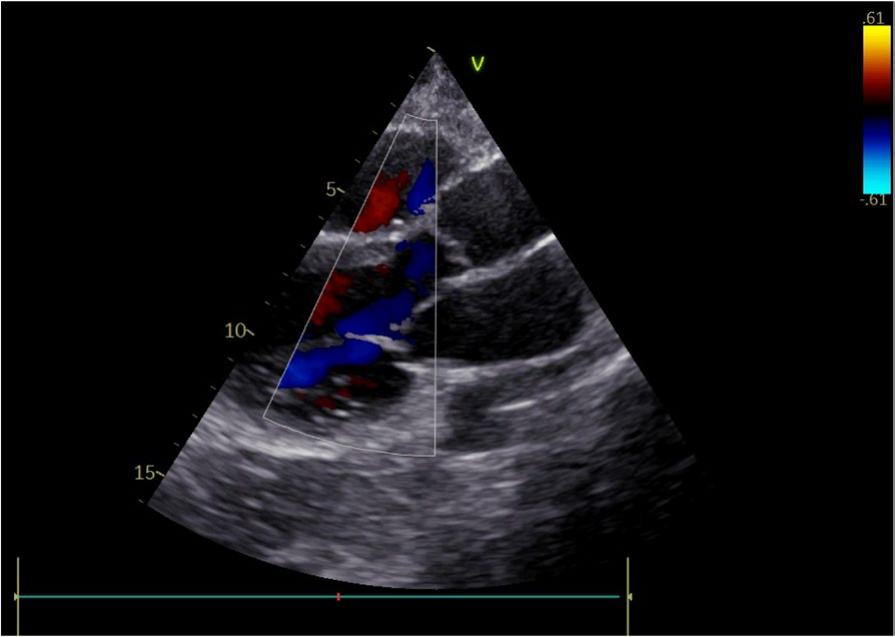
Transthoracic Ultrasound: absence of valvular defects, no effusion, preserved heart chambers size and function

### A165 Intradiscal injection of bone Marrow Concentrate for Lumbar degenerative disc disease: case report

#### E. Cianciola ^1^, F. Saturno ^1^, G. Monaco ^1^, F. Marino ^1^, S. Palladino ^1^, V. Bellini ^2^

##### ^1^ ASL SALERNO -Ospedale di Sapri, Sapri, Italy; ^2^ Azienda Ospedaliera-Universitaria di Parma, Parma, Italy

###### **Correspondence:** F. Saturno

*Journal of Anesthesia, Analgesia and Critical Care 2023,* **3(Suppl 1):**A165

Background

Infiltrative stem cell therapy for degenerative disc disease (DDD) is a relatively new and promising alternative to surgical techniques Chronic low back pain affects approximately 632 million people worldwide with a prevalence of 68% in adults over the age of 60. It has both a social and economic impact on healthcare budgets, making it the most expensive musculoskeletal disorder in the healthcare system.

The exact causes of disc degeneration are complex and difficult to pinpoint; involve aging, genetic predispositions, nutritional factors such as obesity, mechanical trauma, smoking and other related comorbidities.

This is due to a change in collagen synthesis and by NP cells from collagen II to collagen I resulting in loss of proteoglycan and dehydration. The major proteoglycan of the IVD is aggrecan.

Case report

38-year-old patient with lumbosacral pain for about 6 months. Pain characteristics: discogenic pain unresponsive to medical therapy with NSAIDs prescribed by the doctor and to infiltration. He had performed epidural infiltration at another center with partial benefit.

On lumbosacral MRI, there is evidence of dehydration of the intervertebral disc of the L4-5. Vas 6-8 .

Sterile field preparation. Stem cells are taken from the iliac crest using a fluoroscopic technique (Figure 1).

The chosen intervertebral disc is identified by fluoroscopy in A/P projection (Figure 2).

Subsequently, the fluoroscope is positioned in lateral projection and the disc is approached in tunnel vision (Figure 3). Subsequently we proceed to infiltrate the disk as shown in the images.

Once the procedure is completed, the patient reports pain in the infiltration area. Slight increase in back pain in the first three days; then progressive improvement. At the follow-up after 30 days, the patient reports a marked VAS 0 benefit. After 9 months, no discogenic pain, however, he reports new onset lumbar sciatica. You will have to perform a new MRI lumbosacral.

We collected adequate consent to participate in the study

Conclusions

The results of the case report would seem encouraging; however, data in literature are still lacking both in terms of efficacy and safety.

The main concern is the lack of long-term results.

**Fig. 1 (abstract A165).**
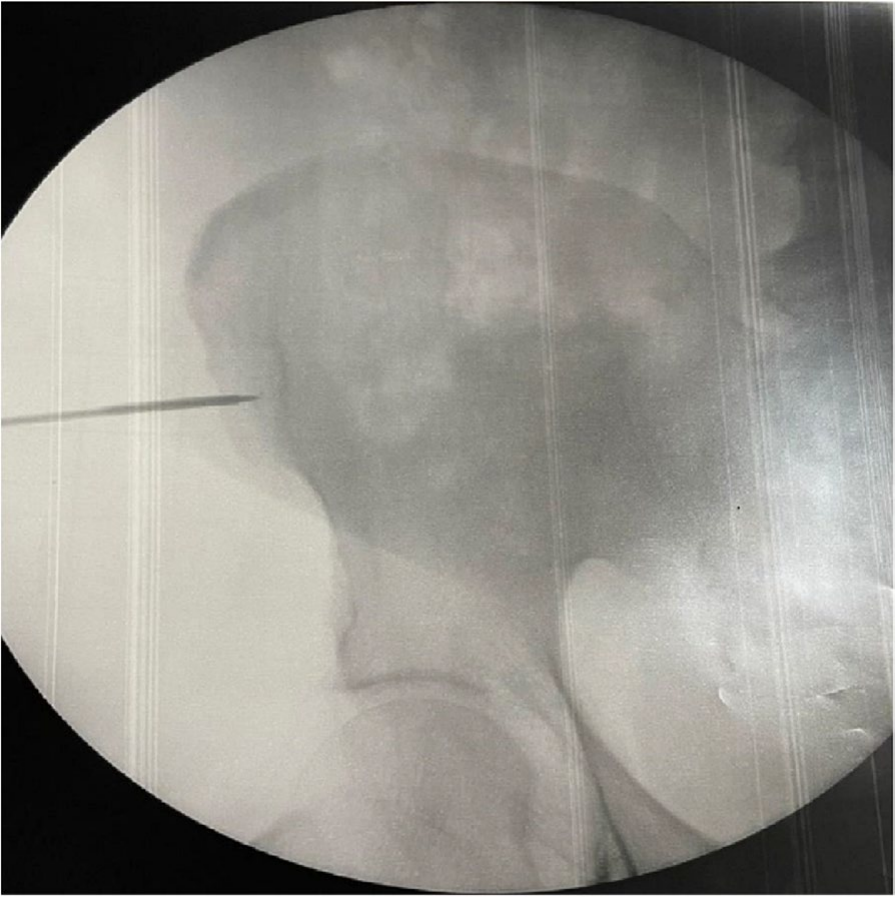
Iliac crest stem cell sampling

**Fig. 2 (abstract A165).**
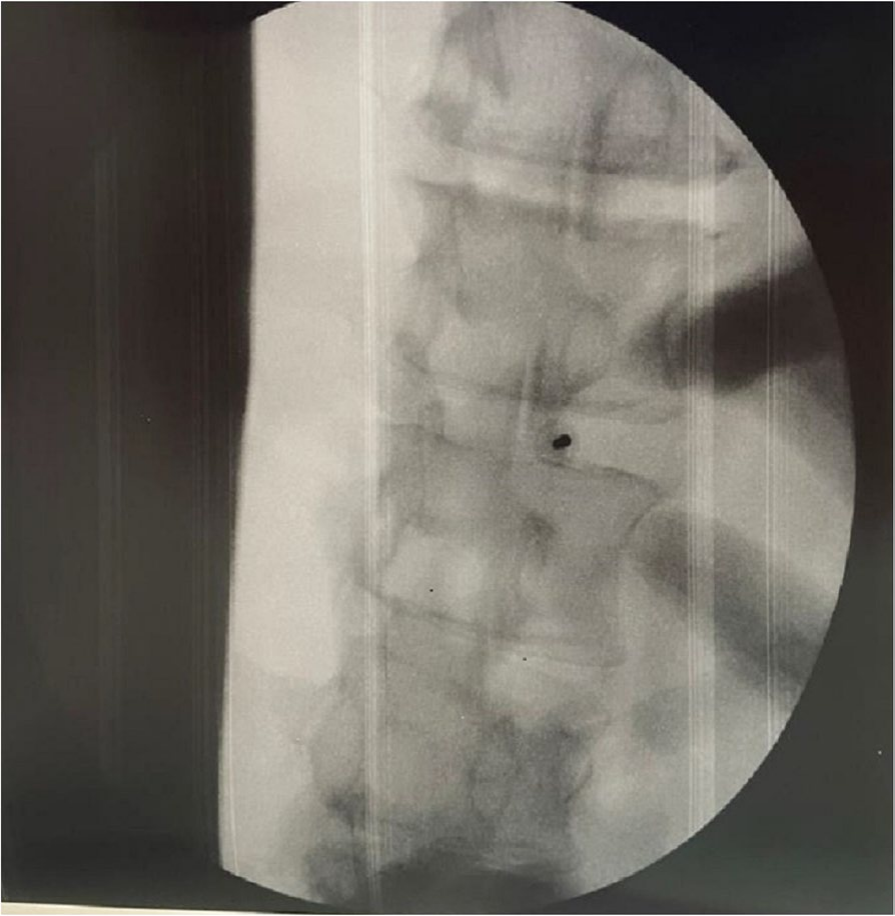
Approach to the intervertebral disc. Antero-posterior view

**Fig. 3 (abstract A165).**
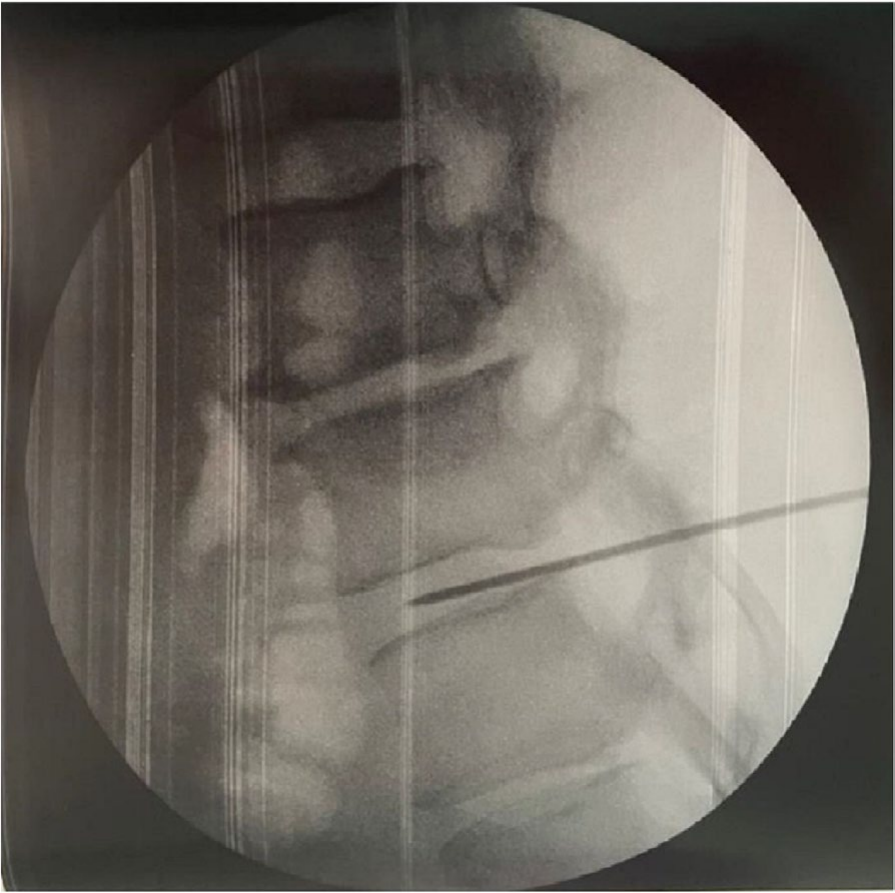
Approach to the intervertebral disc. Latero-lateral view

## Haematology, Haemostasis and Thrombosis

### **A166 The impact of femoral injury on platelets function after discontinuation of clopidogrel treatment**

#### V. Rossi, E. Terreni, A. Fundarò, I. Guerri, A. Bini, L. Gianesello

##### Azienda Ospedaliero-Universitaria Careggi, Firenze, Italy

###### **Correspondence:** E. Terreni

*Journal of Anesthesia, Analgesia and Critical Care 2023,* **3(Suppl 1):**A166

Background

Clopidogrel is P2Y12 ADP platelet receptor inhibitor that is used after coronary stent placement and for stroke prevention [1]. Data from the literature show that about 4% to 30% of patients treated with conventional doses of clopidogrel do not display adequate antiplatelet response [2]. A recent study [3] found that in elderly patients with femoral fracture after 48 hours of clopidogrel discontinuation, 65.7% of patients had a high platelets reactivity. We conducted a retrospective study to determine whether the femoral fracture could impact on platelet function recovery after preoperative clopidogrel discontinuation.

Materials and methods

During a one-year period, after written informed consent, patients that assumed regularly clopidogrel 75 mg per day and underwent orthopedic surgery for femoral fracture (Group 1) o elective surgery for total joint replacement (Group 2), were included in the study. Forty-eight hours after discontinuation of clopidogrel, blood samples of patients were withdrawn into citrated tubes; inhibition of platelet function was determined using Verifynow (Werfen, San Diego, USA) assay and was compared between two groups. Furthermore, demographic and standard laboratory parameters were recorded. A p value <0.05 was considered statistically significant.

Results

A total of sixty patients were enrolled in the study. In the Group 2, 10 patients undergoing total knee replacement, 15 patients undergoing total hip replacement and 5 patients undergoing total shoulder replacement for osteoarthritis, were included. There were no statistical differences into two groups in main demographic and laboratory parameters, apart from age (86.6±9.6 [Group 1] vs. 69.4±6.9 [Group 2], p=0.000042) and glycemia (153.8±60 [Group 1] vs. 83±18.3 [Group 2], p=0.003) (Table 1). At the evaluation time, patients with femoral fracture had a significantly high platelet recovery (Platelet Reaction Unit) than elective surgical patients (244.1±54.5 [Group 1] vs. 113.6±22.2 [Group 2], p<0.00001). However, patients of Group 2 demonstrated a greater reduction in delta hemoglobin between pre and postoperative period compared to Group 1 (14.2±1.6 vs 11±1.6, Group 2 and 12.3±1.8 vs 10.9±1.2, Group 1).

Conclusions

Femoral fracture seems to increase the platelet reactivity 48 hours after preoperative clopidogrel discontinuation, with influence on postoperative hemoglobin. Further large studies are needed to demonstrate the effective recovery time of platelet function in different clinical settings and the impact on need of postoperative transfusions.

References

1. Mohammad RA, et al. Clin Ther 2010; 32: 2265–81; 2. Nguyen AT, et al. JACC 2005;45 (8):1157-64; 3. Gianesello L, et al. Minerva Anestesiol 2022; 88:320-322

**Table 1 (abstract A166).** Demographic and laboratory characteristics of studied population

	Group 1	Group 2	P value
**N. Patients**	30	30	------
**Sex (M/F)**	8/22	12/18	0.27
**Age (years)**	86.6±9.6	69.4±6.9	0.000042
**Creatinine (mg/dL)**	1.7±0,6	0.73±0.02	0.23
**Glicemia (mg/dL)**	153.8±60	83±18.3	0.003
**Hb preoperative (gr/dL)**	12.3±1.8	14.2±1.6	0.37
**Hct preoperative (gr/dL)**	38±5.8	44.0±5.1	0.40
**Platelets preoperative (10** ^**9**^ **/L)**	225.4±66.8	238.2±56.5	0.30
**Hb postoperative (gr/dL)**	10.9±1.2	11.0±1.6	0.30
**Hct postoperative (gr/dL)**	32.7±2.9	33.9±2.7	0.26
**Platelets postoperative (10** ^**9**^ **/L)**	224.8±84	202.8±71.4	0.30
**PRU (platelet reaction unit)**	244.1±54.5	113.6±22.2	<0.00001

Data are expressed as mean±standard deviation or number

*p<0.05

### A167 Use off label of coseal hemostatic glue for treatment of prosthetic bleeding after cardiac surgery - a case report -

#### C. Santonocito ^1^, M. Mazzamuto ^1^, L. Avolio ^1^, M. Giambra ^1^, S. Lentini ^1^, E. Panascia ^1^, A. Caruso ^1,2^

##### ^1^ A.O.U. Policlinico 'G. Rodolico - San Marco', Catania, Italy; ^2^ School of Anesthesia and Intensive Care A.O.U. Policlinico G. Rodolico, Catania, Italy

###### **Correspondence:** A. Caruso

*Journal of Anesthesia, Analgesia and Critical Care 2023,* **3(Suppl 1):**A167

Background: Cardiothoracic surgery is a high risk setting for perioperative complications, mainly bleeding. The etiology of bleeding may be surgical or due to coagulopathy. Unfortunately, it is not always easy to diagnose the etiology of bleeding and if surgical we get several sealants available for helping to solve the bleeding. This case report has been managed at an University hospital and this work has been reported in line with the SCARE 2020 criteria.

Case report: A 37 year old male patient presented with a sudden onset of central chest pain radiating to the back came to the Emergency Department. Imaging investigation showed the presence of type A aortic dissection and he has been referred to our Hospital requiring emergency surgical treatment with prosthetic tube and mechanical prosthetic aortic valve replacement.

His postoperative course in ICU was complicated by severe bleeding requiring five times re-explorations for tamponade. The interesting finding was that the only bleeding site after several surgical re explorations was the prosthetic tube, no other bleeding sites were identified in the whole body (medications, mucosae, skin, nose, mouth, ect…). The clotting tests showed a minor platelet dysfunction and the viscoelastic tests did show a procoagulant pattern. Patient has been polytransfused (over 20 units of RBC, several prothrombin complex concentrates and platelets) with no long lasting results, but the only winning action was an off label use of a large amount of hemostatic glue (COSEAL) not only in the anastomosis and stitches sites but as a “sleeve” around the whole prosthetic tube in a fully bleeding surgical field.

Conclusion: In our experience, after aortic replacement using Vascutek prosthesis tube it is worth to apply the hemostatic glue COSEAL as a “sleeve” all around the whole prosthetic tube before any evidence of bleeding is shown. Moreover, in case of bleeding, even if the viscoelastic tests (ROTEM) did not show coagulopathies it is worth it to check for platelets function or other specific clotting tests and, in case of even minor platelets dysfunction we suggest to transfuse pool of platelets.

Consent to partecipate Written informed consent was obtained from the patient for publication of this case report and accompanying images. A copy of the written consent is available for review by the Editor-in-Chief of this journal on request.

## Invasive and non-invasive ventilation

### **A168 Low-volume extracorporeal carbon dioxide removal is feasible in a non-referral Intensive Care Unit**

#### A. Gattullo, L. Rizzi, A. Toto, A.M. Saponaro, A. Gisotti, N. Di Venosa

##### U.O.C. Anestesia e Rianimazione - Bonomo Hospital, Andria, Italy

###### **Correspondence:** A. Gattullo

*Journal of Anesthesia, Analgesia and Critical Care 2023,* **3(Suppl 1):**A168

BACKGROUNG

Chronic obstructive pulmonary disease (COPD) is the fourth cause of death in Italy and in most countries [1]. It brings irreversible inflammatory injury to the lungs, leading to acute exacerbations that result in respiratory failure and hypercapnia. Tracheal intubation and mechanical ventilation may be necessary, though insufficient. In such cases, extra-corporeal support techniques including CO2 removal (ECCO2R) are an option [2], but often only available in referral centers [3].

CASE DESCRIPTION

Here we describe the case of a 44-year-old male patient who was admitted to our Intensive Care Unit (ICU) after asthma-COPD overlap syndrome exacerbation. The following is reported according to informed consent expressed by the patient after ICU discharge.

He was suffering from severe dyspnea, tachycardia and hypertension, and comatose state prompted immediate intubation. Arterial blood gas analysis (ABG) showed respiratory acidosis (pH 7.21 pCO2 69.3 mmHg) that worsened in the next hours despite conventional therapy and sevoflurane administration, with pCO2 peaking at 146.8 mmHg. We kept tidal volumes as low as 6 ml/kg of predicted body weight and pressures were the following (in cmH2O): peak 46, plateau (Pplat) 25, driving pressure (DP) 20. This caused hemodynamics impairment, that required high dose vasopressor to maintain mean arterial pressure around 70 mmHg. Thus, considering spare oxygenation we decided to start ECCO2R treatment. Haemoperfusion was obtained via a 13.5 Ch bi-lumen catheter using the ProLUNG® System, providing anticoagulation with heparin infusion within the circuit. We managed to keep a low blood flow of 250 ml/min throughout the treatment, a 10 l/min sweep air flow was eventually decreased for weaning, resulting in a VCO2 between 105 and 120 ml/min assumed as half of the patient’s total VCO2 (fig. 1).

Under ongoing sedation and paralysis, ventilation was optimized to keep Pplat <20 cmH2O and DP <15 cmH2O (fig. 2). Vasopressors requirement decreased.

We stopped the treatment after 40 hours and gradual sweep gas weaning. The next day ABG showed pCO2 41.8 and pO2 97.3, allowing paralysis and anaesthetic infusion discontinuation. The patient was able to breath spontaneously after 7 days from the admission, and transferred to ward at day 9.

CONCLUSIONS

Extra-corporeal CO2 removal is helpful in keeping stable blood gases and reduce respiratory acidosis while optimizing mechanical ventilation and its impact on hemodynamics. Moreover, this report reveals it is an easy technique that we managed to successfully administer via simple equipment in our non-refferral ICU.

REFERENCES

1. Christenson SA et al. Chronic obstructive pulmonary disease; Lancet (2022)
2. Fanelli V et al. Feasibility and safety of low-flow extracorporeal carbon dioxide removal to facilitate ultra-protective ventilation in patients with moderate acute respiratory distress sindrome; Crit Care (2016)
3. Augy JL et al. A 2-year multicenter, observational, prospective, cohort study on extracorporeal CO2 removal in a large metropolis area; Journal of intensive care (2019)

**Fig. 1 (abstract A168).**
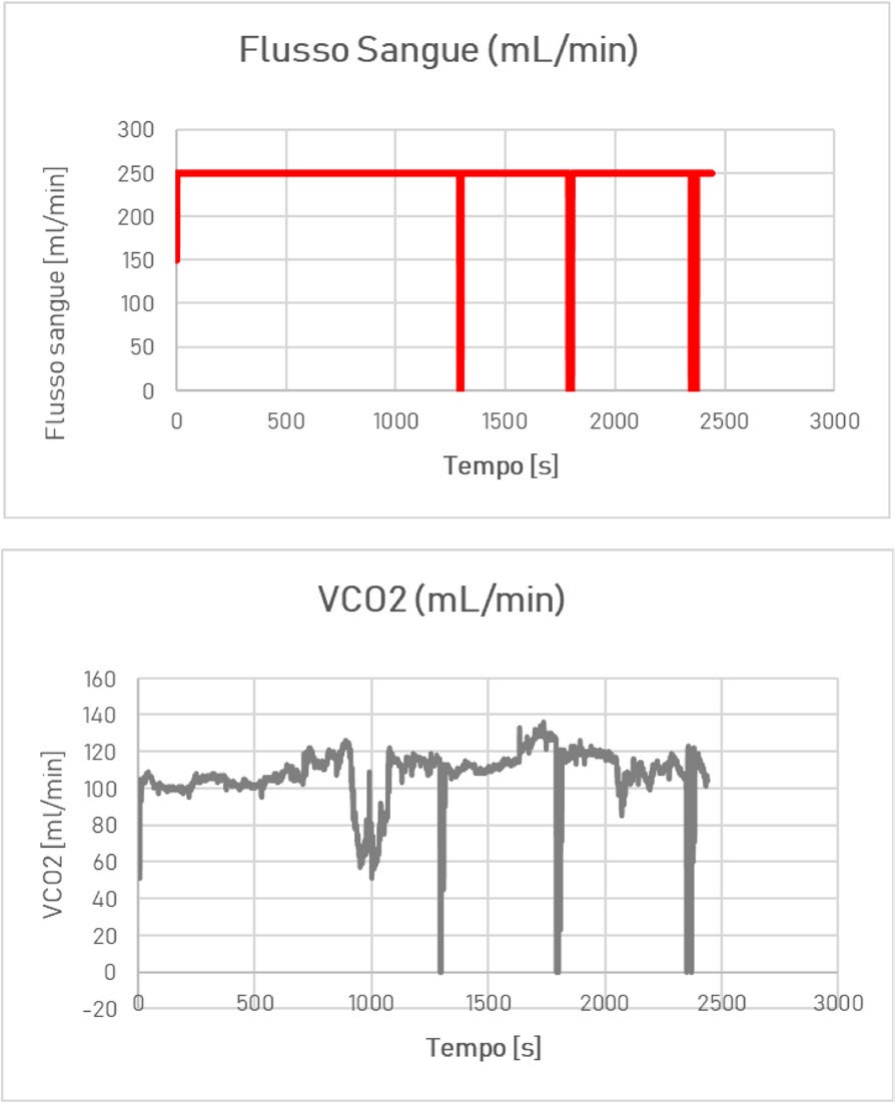
See text for description

**Fig. 2 (abstract A168).**
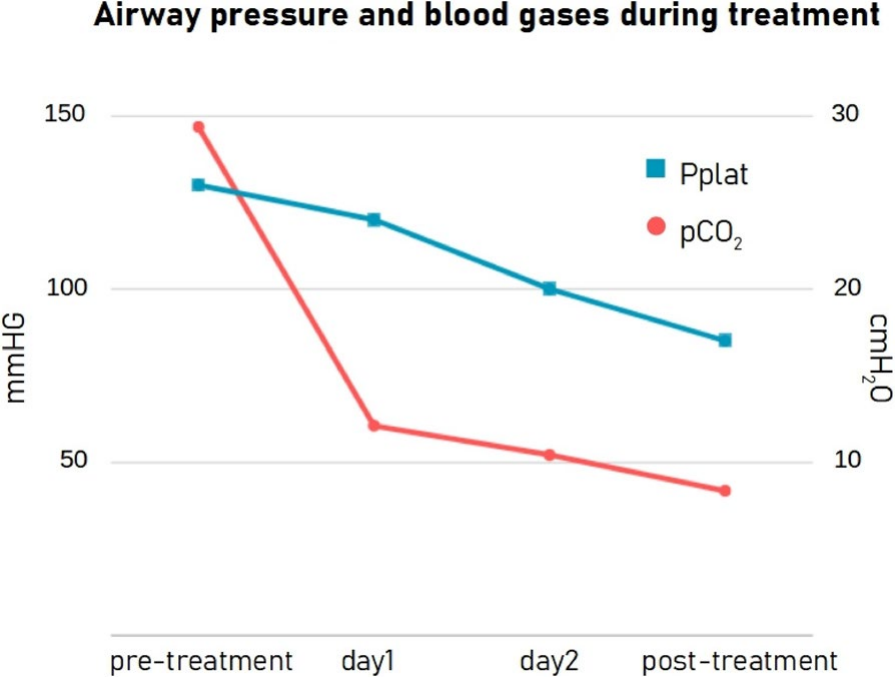
The lines show how plateau pressure (blue, squares) and arterial CO2 (red, dots) improved during extracorporeal carbon dioxide removal

### A169 Effect of pneumoperitoneum and laparoscopy on lung EIT-derived overinflation and collapse in morbidly obese patients

#### P. Priani ^1^, G. Scaramuzzo ^1,2^, R. Ragazzi ^1,2^, R. La Rosa ^1^, V. Chiavieri ^1^, P. Ferrara ^1^, M. Verri ^2^, C.A. Volta ^1,2^, S. Spadaro ^1,2^

##### ^1^ Department of Translational Medicine, University of Ferrara, Via Aldo Moro 8, 44121, Ferrara, Italy, Ferrara, Italy; ^2^ 2Anesthesia and Intensive Care Medicine, Azienda Ospedaliero Universitaria Di Ferrara, Via Aldo Moro 8, 44124, Cona, FE,, Ferrara, Italy

###### **Correspondence:** P. Priani

*Journal of Anesthesia, Analgesia and Critical Care 2023,* **3(Suppl 1):**A169

(scrgtn@unife.it)

Background

Obesity can expose patients to a higher risk of atelectasis during general anesthesia, especially during laparoscopic surgery [1]. PEEP can counteract the tendency of dorsal lung to collapse, but it needs to be personalized to minimize both the risks of overinflation (OI) and collapse (CO). Electrical Impedance Tomography (EIT) can be used to personalize the PEEP level during surgery, but the effect of the surgical phase on the best PEEP provided by EIT in obese patients has not been explored yet.

Materials and Methods

After obtaining informed consent, we enrolled 27 obese (BMI over 30) patients undergoing planned abdominal laparoscopic surgery. A 16-electrodes EIT monitoring (PulmoVista500, Draeger, Germany) was used to evaluate the amount of CO and OI during a PEEP titration trial (PEEP 16 cmH2O - PEEP 6 cmH2O; steps of 2 cmH2O). The best PEEP was defined as the crossing point between CO/OI [2]. The assessment was done after anesthesia induction (BPind), during pneumoperitoneum (BPpneumo) and before extubation (BPext).

Results

We analyzed data from 27 patients, aged 65 [52-82] years old with a BMI of 36±5 kg/cm2. The overall duration of anesthesia was 175 [133-300] minutes, the duration of the laparoscopic time 112±61 minutes. The median [IQR] values of CO and OI after induction (A), pneumoperitoneum (B) and before extubation (C) are reported in figure 1.

We found a statistically significant difference between BPind (10 [9-15] cnmH2O) and BPpneumo (14 [11-16] cmH2O, p<0.0001) while no difference was found between BPind and BPext (10 [8-14] cmH2O, p= 0.63). Pneumoperitoneum reduced the OI at the highest levels of PEEP (p<0.0001) and increased significantly CO at the lowest PEEP (p<0.0001).

Conclusions

In obese patients undergoing laparoscopy, the PEEP able to minimize CO and OI changes dynamically during surgery, with higher levels during the laparoscopic phase. A continuous monitoring is needed to provide the best PEEP levels according to the surgical step.

References

1. Eichenberger A-S et al. Morbid Obesity and Postoperative Pulmonary Atelectasis: An Underestimated Problem. Anesthesia & Analgesia 2002;95:1788–92.
2. Costa ELV et al. Bedside estimation of recruitable alveolar collapse and hyperdistension by electrical impedance tomography. Intensive Care Med 2009;35:1132–7.

**Fig. 1 (abstract A169).**
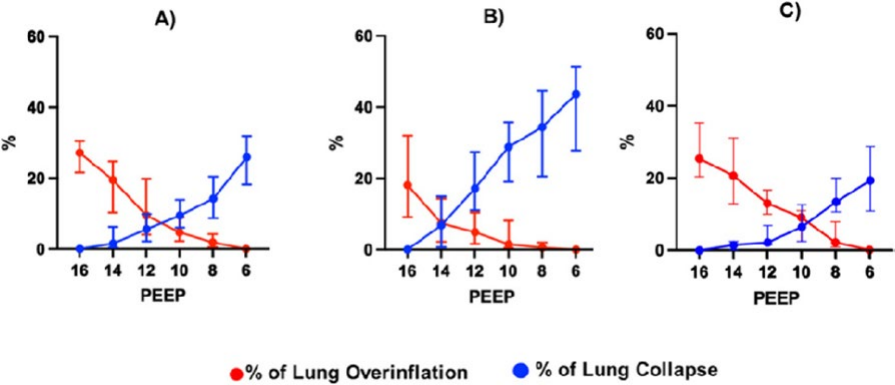
Percentage of lug collapse and overinflation at different PEEP levels in the three steps: after induction (A), during pneumoperitoneum (B) and before extubation (C)

### A170 Effective use of High Flow Nasal Cannula (HFNC) in Abernethy syndrome: a case report

#### D. Pisani ^1^, A. Corriero ^2^, P. Ferrara ^1^, C. Ferrari ^1^, F. Puntillo ^2^, M. Ribezzi ^1^

##### ^1^ Anesthesia and Intensive Care I A.O.U.C. Policlinico di Bari, Bari, Italy; ^2^ Department of Interdisciplinary Medicine - ICU Section University of Bari Aldo Moro, Bari, Italy

###### **Correspondence:** D. Pisani

*Journal of Anesthesia, Analgesia and Critical Care 2023,* **3(Suppl 1):**A170

BACKGROUND

Abernethy syndrome [1], also known as congenital extrahepatic portosystemic shunt (CEPS), is a rare condition of hepatic fibrosis associated with pulmonary hypertension and chronic hypoxemia. There are two variants of the syndrome. CEPS type 1 sees the total absence of a portal vein (PV), while in CEPS type 2 there is a congenital extrahepatic portosystemic shunt with tiny PV radicles.

CASE REPORT

A 26-year-old Caucasian woman arrived at the emergency department alert, with severe abdominal pain, asthenia, hypotension and fever. She had a history of closure of the Botallo duct, celiac disease, polycystic ovary syndrome and CEPS type 2. The usual patient therapy included Tadalafil and Macitentan for pulmonary hypertension diagnosed incidentally in the previous four years during hospitalization for mild chest trauma. Last pulmonary angiography revealed a pulmonary resistance of 10 Woods and 100/60 mmHg sisto/diastolic pulmonary pressure. The patient underwent a chest-abdomen CT scan showing diffuse ground glass parenchymal thickening areas in both lungs, ileal loops distended by liquids and no air-fluid levels. Surgical consultation and cardiological evaluation followed, which prompted no critical situation. The cardiac ultrasound examination showed a slight tricuspid regurgitation and a considerable dilatation of the inferior vena cava with PAPs 95-100 mmHg. Twenty-four hours after admission to the emergency room, the patient presented with tachypnea, desaturation, fever and arterial hypotension (SBP 70 mmHg). Venturi-type oxygen mask (FiO2 50%) was positioned and norepinephrine infusion started. Lab work showed leukocytosis and increased CRP, then the patient developed respiratory alkalosis (pH 7.46 PaO2 61 mmHg PaCO2 33 mmHg) and septic shock. Therefore a CPAP ventilation started, which led to no clinical improvement and patient was admitted to the ICU, where she began a cycle of High-Flow Nasal Cannula (HFNC) [2]. During the ICU stay, an antibiotics cycle was set for the septic state and norepinephrine and fluid support continued. In the following six days after ICU admission, each organ function gradually recovered, resolving tachypnea, acid-base and electrolyte disturbances, and other lab tests. After ten days from ICU admission, the patient was apyretic with minimal asthenia, allowing her transfer to the cardiology department for follow up.

CONCLUSION

This case report shows that patients in good general health generally tolerate mild hypoxemia caused by Abernethy syndrome associated with pulmonary hypertension. However, in conditions of clinical deterioration, the use of HFNC can find a positive response because it does not increase intrathoracic pressures and can guarantee a rapid recovery with good patient tolerance, as demonstrated by our experience.

Informed Consent Statement: Informed consent was obtained from a legally authorized representative of the subject.

References

1. Sahu MK, Bisoi AK, Chander NC, Agarwala S, Chauhan S. Abernethy syndrome, a rare cause of hypoxemia: A case report. Ann Pediatr Cardiol. 2015 Jan-Apr;8(1):64-6.
2. Maggiore, S.M., Grieco, D.L. & Lemiale, V. The use of high-flow nasal oxygen. Intensive Care Med (2023).

### A171 Impedance analysis of lung perfusion in severe ards uses for patient’s tailored bedside therapy with PDE3-Inhibitor enoximone

#### M. Pillitteri ^1^, F. Velasco ^2^, B. Ferro ^3^, L. Luzzi ^3^, P. Roncucci ^3^

##### ^1^ Università di Pisa, Pisa, Italy; ^2^ Ospedale Civile Elba, Portoferraio, Italy; ^3^ Ospedali Riuniti di Livorno, Livorno, Italy

###### **Correspondence:** M. Pillitteri

*Journal of Anesthesia, Analgesia and Critical Care 2023,* **3(Suppl 1):**A171

Introduction

Acute Respiratory Distress Syndrome (ARDS) is a complex and severe pathology secondary to inflammatory damage to lung parenchyma.

The pathophysiological peculiarity of ARDS hypoxemia is the mismatch between alveolar ventilation (V) and pulmonary perfusion (Q) determined by alteration of vasoconstriction hypoxemia with perfusion of collapsed zones and hypoperfusion of spared zones (1).

Patient’s tailored bedside therapy should recognize mismatched V/Q areas using different approaches.

Electrical impedance tomography (EIT) is a non-invasive and radiation-free tool used to monitor bedside and in real-time the distribution of ventilation and perfusion in lungs through the dynamic study of lung distention in a period. EIT permits to estimate alveolar collapse and overdistension, lung recruitment using incremental PEEP and studies lung perfusion (2).

PDE3-inhibitors Enoximone is a well-known medication with vasodilator, bronchodilator, inotropes and anti-inflammatory properties. Its use for reducing cardiac preload and oxygen consumption in hemodynamic instability is common in ICU (3).

During Sars-Cov2 pandemic Enoximone has been used both in aerosolized and endovenous (ev) forms in mechanically and non-invasive ventilated patients, exploiting its vasodilator properties, to act on pulmonary shunt and V/Q mismatch in poor recruitable lungs (4).

Description

We enrolled three patients, a woman and two men, affected by severe Sars-Cov2 ARDS, which necessitated invasive mechanical ventilation and second line therapies because of the poor response in terms of gas exchanges. Due to patients’ critical conditions we couldn’t have obtained informed consent.

EIT perfusion showed marked altered discrepancy between perfusion and ventilation despite tailored PEEP.

We analyzed EIT data before and after ev infusion of enoximone 6 mg/kg/min for 48 hours and we demonstrated a gradual improvement of lung perfusion in 2 patients (responders), a reduction of V/Q mismatch and a decrease of the shunt measured by the reduction of difference in alveolar-arteriosus PaCO2 from 12±3 to 8±3 (p=0,04). One patient did not respond to endovenous treatment.

Discussion

In this report we described the ability of EIT as an aid to monitor altered perfusion in severe ARDS. Furthermore we observed some responsiveness to endovenous treatment with Enoximone with an improvement of the lung perfusion concordant to an increasement of CO2 clearance.

The potential use of impedance tomography to evaluate perfusion and its evolution in ARDS is of interest and could help clinicians in the assessment of tailored therapies and modality of ventilation.

References

A) Slobod D et al. Pathophysiology and Clinical Meaning of Ventilation-Perfusion Mismatch in the Acute Respiratory Distress Syndrome. Biology (Basel). 2022 Dec 30;12(1):67
B) Bachmann MC et al Electrical impedance tomography in acute respiratory distress syndrome. Crit Care. 2018 Oct 25;22(1):263
C) Smith NA et al. Clinical pharmacology of intravenous enoximone: pharmacodynamics and pharmacokinetics in patients with heart failure. Am Heart J. 1991 Sep;122(3 Pt 1):755-63
D) Beute J et al. PDE3-inhibitor enoximone prevented mechanical ventilation in patients with SARS-CoV-2 pneumonia. Exp Lung Res. 2021 Apr;47(3):149-160

**Fig. 1 (abstract A171).**
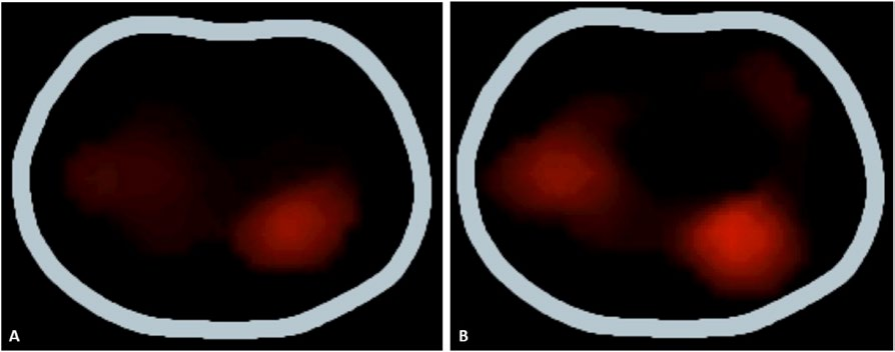
EIT study of lung perfusion in responder patient: A. Before Enoximone infusion; B. After 48h of Enoximone infusion

### A172 Can levosimendan improve quality of respiratory weaning?

#### L. Coen Tirelli ^1^, A. Tiberi ^1^, F. Marchetti ^1^, P. Picerno ^1^, B. Baldelli ^1^, L. Di Marzio ^1^, S. Carlini ^1^, D. Fiume ^2-3^, A.M. Martini ^3^, M. Peverini ^3^, M. Galletti ^3^

##### ^1^ Tor Vergata Hospital, Rome, Italy; ^2^ UniCamillus University, Rome, Italy; ^3^ Sant'Eugenio Hospital, Rome, Italy

###### **Correspondence:** L. Coen Tirelli

*Journal of Anesthesia, Analgesia and Critical Care 2023,* **3(Suppl 1):**A172

BACKGROUND

Levosimendan is a cardiac inotrope that augments the calcium sensitivity of the troponin C complex, that has been shown to improve cardiac muscle contractility and is approved for clinical application worldwide for treatment of heart failure. [1,2]

CASE REPORT

A 77-year-old female patient was admitted to the ICU after emergency surgery for gastric perforation. In addition she reported pulmonary contusions, multiple rib fractures with small pneumothorax, post traumatic subdural haematoma due to an asyncopal episode, and senile dementia.

Diaphragmatic US was performed daily. A weaning attempt was carried out, with an alterned cycles HFNC and NIV-helmet; at the end reintubation was unfortunately necessary. After 48 hours of levosimendan infusion we observed a double increasing of diaphragmatic excursion. Endotracheal tube was removed with efficient weaning.

CONCLUSIONS

More recently, it has been demonstrated that levosimendan improves in vivo contractile efficiency of the diaphragm in healthy subjects. Based on studies in vitro and in vivo, it has been hypothesized that levosimendan improves diaphragm contractile efficiency in patients weaning from mechanical ventilation.[3] Tidal volume and minute ventilation were higher after levosimendan administration, not significately.[4]

In our case report we observed an increased muscle strength, maybe due to levosimendan infusion. Further studies are needed to confirm this hypothesis.

Informed consent to publish had been obtained.

REFERENCES

1. Papp Z, Edes I, Fruhwald S, De Hert SG, Salmenpera M, Leppikangas H, Mebazaa A, Landoni G, Grossini E, Caimmi P, Morelli A, Guarracino F, Schwinger RH, Meyer S, Algotsson L, Wikstrom BG, Jorgensen K, Filippatos G, Parissis JT, Gonzalez MJ, Parkhomenko A, Yilmaz MB, Kivikko M, Pollesello P, Follath F. Levosimendan: molecular mechanisms and clinical implications: consensus of experts on the mechanisms of action of levosimendan. Int J Cardiol 2012;159(2):82–87
2. Follath F, Cleland JG, Just H, Papp JG, Scholz H, Peuhkurinen K, Harjola VP, Mitrovic V, Abdalla M, Sandell EP, Lehtonen L, Steering C, Investigators of the Levosimendan Infusion versus Dobutamine. Efficacy and safety of intravenous levosimendan compared with dobutamine in severe low-output heart failure (the LIDO study): a randomised double-blind trial. Lancet 2002; 360(9328):196–202
3. Doorduin J, Sinderby CA, Beck J, Stegeman DF, van Hees HW, van der Hoeven JG, Heunks LM. The calcium sensitizer levosimendan improves human diaphragm function. Am J Respir Crit Care Med 2012; 185(1):90–95
4. Roesthuis L, van der Hoeven H, Sinderby C, Frenzel T, Ottenheijm C, Brochard L, Doorduin J, Heunks L. Effects of levosimendan on respiratory muscle function in patients weaning from mechanical ventilation. Intensive Care Medicine 2019; 45:1372–1381

**Fig. 1 (abstract A172).**
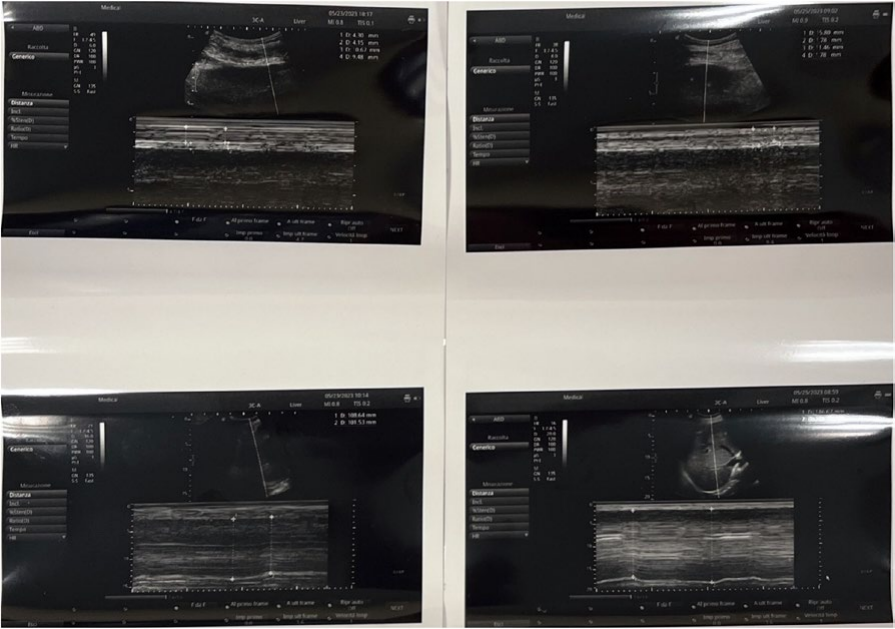
See text for description

### A173 Concurrent aerosol drug delivery high flow nasal therapy in a simulated adult model

#### M. Mac Giolla Eain ^1^, E. Fernández Fernández ^2^, G. Bennett ^2^, R. MacLoughlin ^1^

##### ^1^ R&D - Science and Emerging Technologies, Aerogen Ltd, Galway, Ireland; ^2^ Medical Affairs, Aerogen Ltd, Galway, Ireland

###### **Correspondence:** E. Fernández Fernández

*Journal of Anesthesia, Analgesia and Critical Care 2023,* **3(Suppl 1):**A173

Background

High flow nasal therapy (HFNT) as an alternative to invasive mechanical ventilation (IMV) in spontaneously breathing patients has found increasing adoption in the critical care setting. HFNT allows for concurrent delivery of aerosolised medications to treat patients with respiratory disorders or undergoing respiratory distress. While there are numerous HFNT systems in use, the ability of these systems to effectively deliver aerosolised medications is not always known. This study assessed the ability of a new HFNT system combined with a vibrating mesh nebuliser (VMN) to successfully deliver aerosolised medication to a simulated spontaneously breathing adult patient.

Materials & Methods

2000 μg of salbutamol (TEVA, IRE) was aerosolised using the Aerogen Solo VMN and Aerogen Pro-X controller (Aerogen, IRE). The nebuliser was integrated into the O2FLO High Flow Respiratory Humidifier (Vincent Medical inspired, HK) on the wet-side of the humidification chamber using the Aerogen adult T-piece. The aerosol was delivered to an anatomically correct adult head model (LUCY) via medium nasal cannula (inspired O2Flo, Vincent Medical) at flow rates of 10, 30 & 60 LPM. The head model was connected to a breathing simulator (ASL 5000, IngMar Medical, USA) set to simulate a normal adult breathing pattern (BR: 15 BPM, Vt: 500 mL, I:E: 1:1). A capture filter was placed at the level of the trachea and the quantity of aerosol available for inhalation was determined via UV-spectrophotometry at 276 nm. Testing was completed in independent quintuplicate.

Results

Results for the tracheal dose are expressed as a percentage of the nominal dose placed in the nebuliser’s medication cup and are presented in Figure 1. No equipment alarms or malfunctions were noted during testing.

Conclusions

Here, for the first time, we have shown that the inspired O2FLO High Flow Respiratory Humidifier system can be used in combination with the Aerogen Solo VMN to successfully deliver aerosol to a spontaneously breathing adult patient. Results reported here show that flow rate has a statistically significant impact on tracheal dose (p=0.000) with tracheal dose decreasing with increasing flow rate, and similar levels of drug delivery were achieved to those reported in the literature for other HFNT systems [1].

Reference

1. Murphy, B. et al., ERJ Open; 2022 (8:0020-2022); DOI: 10.1183/23120541.00220-2022.

**Fig. 1 (abstract A173).**
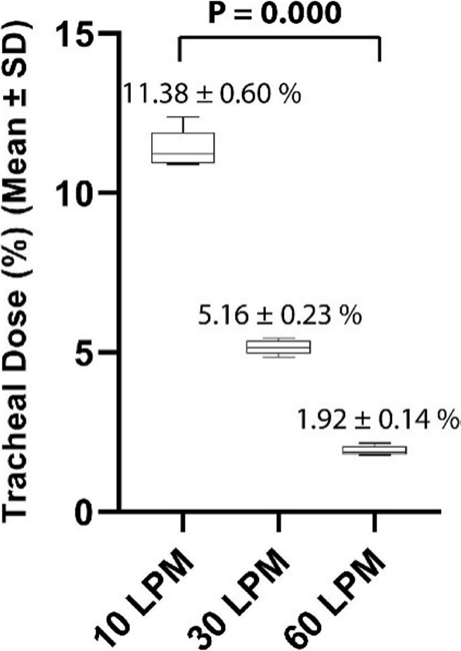
Effects of flow rate (LPM) on the tracheal dose (%) (Mean ± SD) available to a spontaneously breathing simulated adult patient

## Airway management

### **A174 Videolaringoscopy as a standard technique for all tracheal intubations: Has the time for change arrived?**

#### L.M. Titherington ^1^, E. Tur ^1^, G. Baldini ^1^, G. Villa ^1^, E. Angeli ^2^, F. Barbani ^2^, L. Fontanarosa ^2^, S. Romagnoli ^1^

##### ^1^ Department of Anesthesia and Critical Care, Azienda Ospedaliero-Universitaria Careggi; ^2^ Department of Health Science, University of Florence, Florence, Italy

###### **Correspondence:** L.M. Titherington

*Journal of Anesthesia, Analgesia and Critical Care 2023,* **3(Suppl 1):**A174

Background

Standard Macintosh direct laryngoscopy (DL) is the first-line technique for intubation in patients who do not meet pre-operative criteria for a difficult airway and is considered a safe technique in Cormack&Lehane classes I and IIa. Video laryngoscopy (VL) is commonly reserved as a “second line” device in the management of both unpredicted and predicted difficult airways(1). However, more than 90% of all difficult tracheal intubations are unanticipated (2), and multiple intubation attempts are associated with increased risk of adverse events(3). The technological improvement, the decrease in cost, and the progressive diffusion, are pushing us to investigate whether VL should become a standard technique as opposed to a second-line strategy.(4)

Materials and methods

Twenty-four anaesthesia practitioners (10 attendings and 14 residents) were invited to use the McGrath VL device in all the patients scheduled for general anaesthesia with tracheal intubation in 7 theatres of general/urologic surgery during a two-day session. Our aim was to investigate the safety and efficacy profile of VL measured as first-attempt successful intubation ratio and the operators’ satisfaction.

Results

The results can be graphically summarised. See Figures 2, 3 and 4.

Conclusion

Use of VLs as a standard strategy for tracheal intubation could increase the rate of first-attempt successful intubations and thus reduce rates of failed intubation. Our results show that it is a safe and easy to use tool both in hands of experienced attendings and young residents. In light of this, VL-based technique could replace, in the near future, the “traditional” technique as the standard for all intubation procedures. However, further large-scale comparative clinical trials are needed to determine its use a first-line device in routine intubations.

References

1. Frerk C, Mitchell VS, McNarry AF, Mendonca C, Bhagrath R, Patel A, et al. Difficult Airway Society 2015 guidelines for management of unanticipated difficult intubation in adults. Br J Anaesth. 2015;115(6):827–48.
2. Nørskov AK, Rosenstock C V., Wetterslev J, Astrup G, Afshari A, Lundstrøm LH. Diagnostic accuracy of anaesthesiologists’ prediction of difficult airway management in daily clinical practice: A cohort study of 188 064 patients registered in the Danish Anaesthesia Database. Anaesthesia. 2015;70(3):272–81.
3. Hasegawa K, Shigemitsu K, Hagiwara Y, Chiba T, Watase H, Brown CA, et al. Association between repeated intubation attempts and adverse events in emergency departments: An analysis of a multicenter prospective observational study. Ann Emerg Med [Internet]. 2012;60(6):749-754.e2. Available from: http://dx.doi.org/10.1016/j.annemergmed.2012.04.005
4. Penketh J, Kelly FE, Cook TM. Use of videolaryngoscopy as the first option for all tracheal intubations: technical benefits and a simplified algorithm for airway management. Br J Anaesth [Internet]. 2023;130(4):e425–6. Available from: https://doi.org/10.1016/j.bja.2022.12.023

**Fig. 1 (abstract A174).**
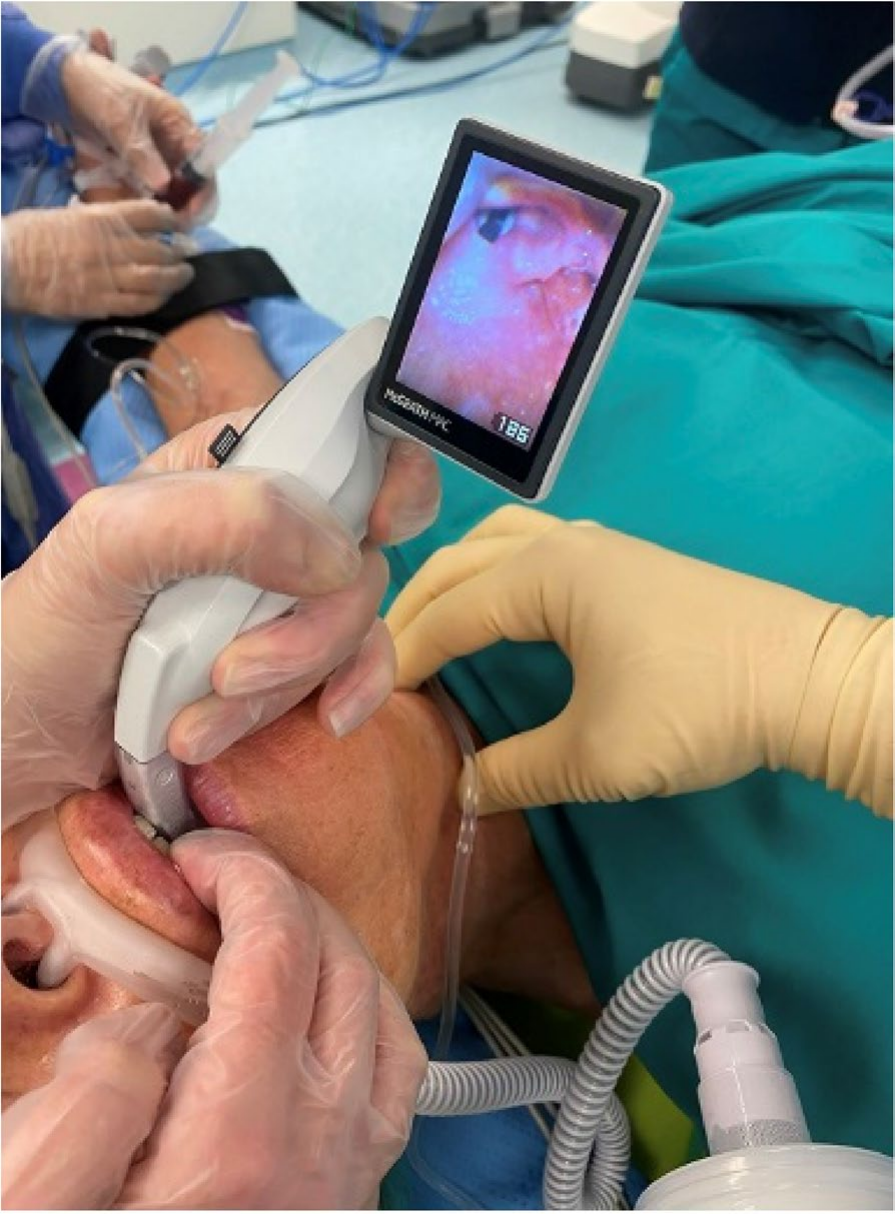
Tracheal intubation with the McGrath X-blade (hyperangulated blade)

**Fig. 2 (abstract A174).**
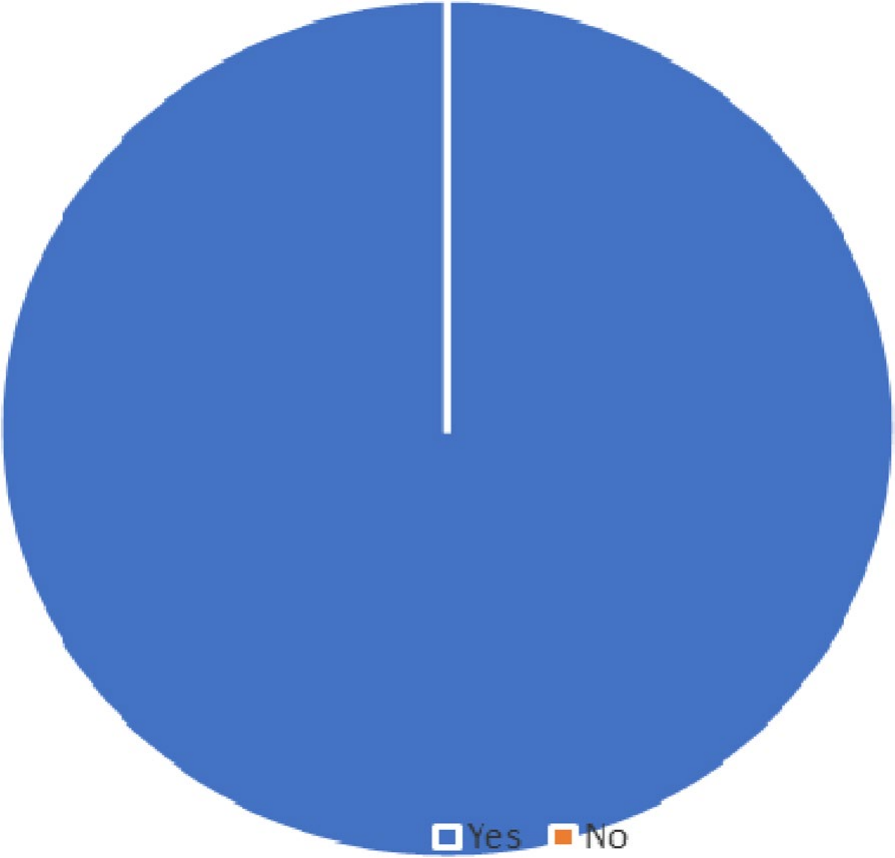
Previous experience with a type of VL

**Fig. 3 (abstract A174).**
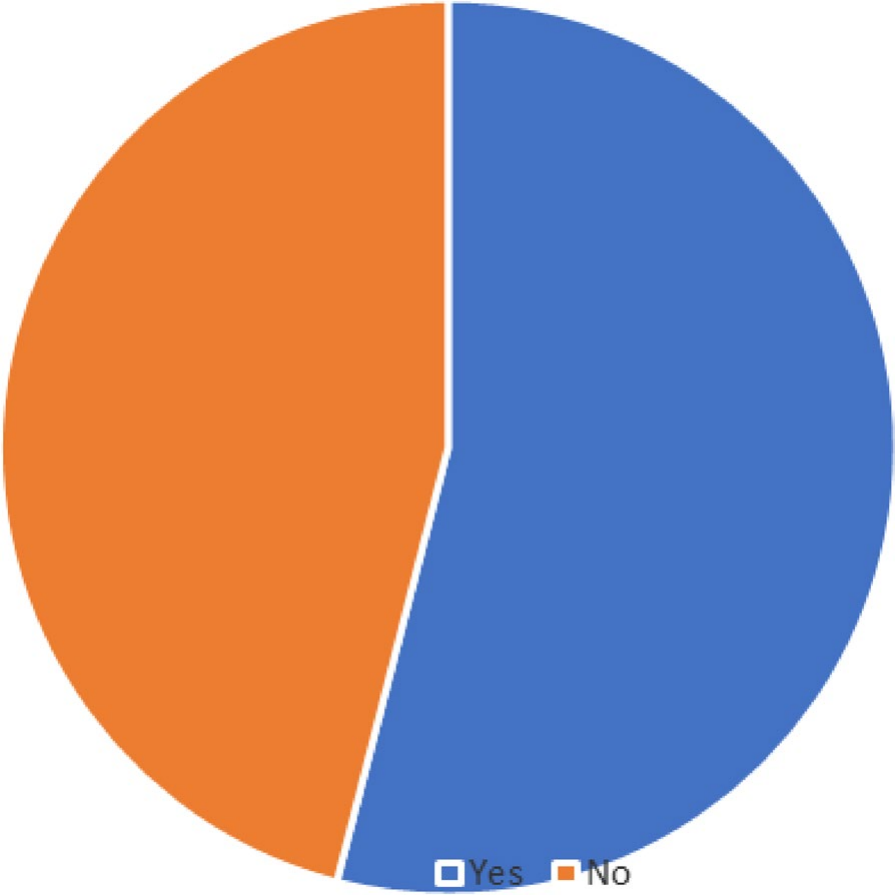
Successful intubation after first attempt

**Fig. 4 (abstract A174).**
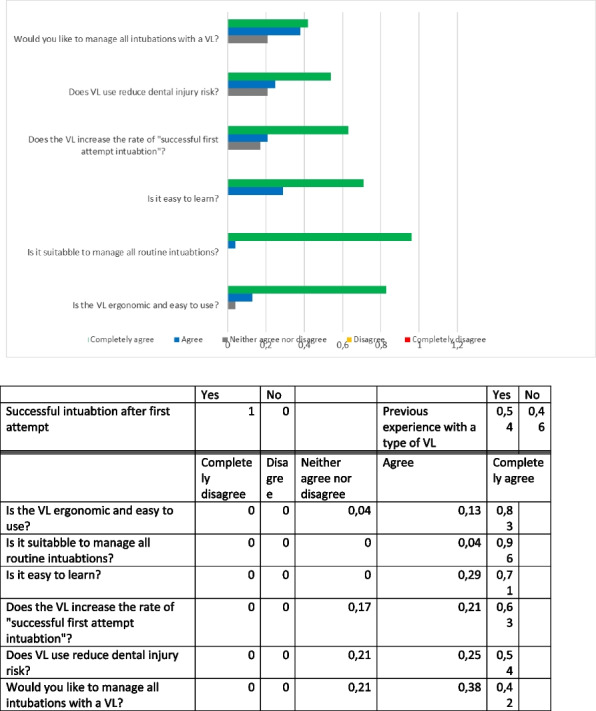
Results of safety and efficacy questionnaire

### A175 Ultrasound evaluation of upper airway in pediatric patients: an obscure field that is worth exploring?

#### J. Silvestri ^1^, M. Ciuffreda ^2^, E. Pisello ^1,2^, C. Chinigioli ^1^, L. Brugiaferri ^1^, S. Sorrenti ^1^, D. Galante ^3^, C. Piangatelli ^2^

##### ^1^ Università Politecnica delle Marche, Ancona, Italy; ^2^ U.O.C. Anestesia e Rianimazione, Terapia Intensiva e del Dolore, Ospedale E. Profili, Fabriano, Italy; ^3^ U.O.C. Anestesia e Rianimazione, Terapia Intensiva e del Dolore, Ospedale G. Tatarella, Cerignola, Italy

###### **Correspondence:** J. Silvestri

*Journal of Anesthesia, Analgesia and Critical Care 2023,* **3(Suppl 1):**A175

Background

Airway management in pediatric patients is one of the main concerns for anesthesiologists and unpredictable difficult intubation remains one of the most important challenges in routine practice. Most of the common preoperative scores predicting difficult intubation have limited predictive value when used on their own. Recently, an ultrasound examination of the upper airway, in particular of anterior cervical soft tissue thickness measuring the distance in centimeters from the epidermis to the epiglottis (DSE, Distance from Skin to Epiglottis), has been proposed as a possible preoperative predictor of difficult intubation in adults, but in pediatric field limited or no data is available.

Objectives

The aim of our preliminary study is to evaluate whether routine use of videolaryngoscope in pediatric patients can reduce risk of difficult intubation in relation to DSE value and to explore if there is a correlation between neck circumference and DSE.

Materials and methods

A total of 72 pediatric patients (3 to 16 years old) undergoing elective surgery under general anaesthesia with tracheal intubation were recruited. Before surgery, DSE with a linear ultrasound transducer placed in the transverse plane at the level of thyrohyoid membrane and the neck circumference were measured for each patient. All patients were routinely intubated using a videolaryngoscope and visual findings were graded according to the Fremantle Videolaryngoscope Scoring System.

Results (Table 1)

- The distribution of DSE values followed a Gaussian curve, with the most frequent one found between 1.6 and 2 cm (52.78%) (Graphic 1).
- Relating DSE values to videolaryngoscopic view, we found a complete vision of vocal cords in 97.22% of cases regardless of DSE values.
- Tracheal intubation occurred in all patients regardless of DSE value, and that was performed at the first attempt in 97.22% of cases and in the rest of cases (2.78%) at the second attempt. There was no impossible intubation.
- In the group of patients with neck circumference between 20 and 25 cm, most (57.14%) had a DSE value between 1.1 and 1.5 cm, in those with circumference between 26 and 30 cm, 65.85% had a DSE of 1.6-2.0 cm, in those with circumference between 31 and 35 cm, 50% had a DSE of 1.6-2.0 cm and 25% of 2.1-2.5 cm, and finally among those with circumference > 35 cm, most (37.5%) had a DSE of 2.1-2.5 cm (Table 2).

Conclusions

DSE evaluation is an ultrasound-based operator-dependent technique that often prolongs preoperative evaluation time of the non-always-cooperating pediatric patients. Routine videolaryngoscopy, optimizing the view of vocal cords and facilitating tracheal intubation, seems to reduce risk of difficult intubation surpassing the DSE evaluation. In addition, a direct proportionality seems to exist between neck circumference and DSE, but certainly further studies are needed in the pediatric field.

**Table 1 (abstract A175).** See text for description

DSE (cm)	N (%)	Neck circumference (cm)	View of vocal cords with videolaryngoscopy (F = full, P = partial, N = none)	Intubation difficulty (1 = at first attempt, 2 = at second attempt or additional devices required, 3 = failed)
**0.6-1.0**	3 (4.17%)	20-25 0	F 3 (100%)	1 3 (100%)
		26-30 1 (33.33%)	P 0	2 0
		31-35 1 (33.33%)	N 0	3 0
		> 35 1 (33.33%)		
**1.1-1.5**	10 (13.89%)	20-25 4 (40.0%)	F 10 (100%)	1 10 (100%)
		26-30 3 (30.0%)	P 0	2 0
		31-35 1 (10.0%)	N 0	3 0
		> 35 2 (20.0%)		
**1.6-2.0**	38 (52.78%)	20-25 1 (2.63%)	F 37 (97.37%)	1 37 (97.37%)
		26-30 27 (71.05%)	P 1 (2.63%)	2 1 (2.63%)
		31-35 8 (21.05%)	N 0	3 0
		> 35 2 (5.26%)		
**2.1-2.5**	17 (23.61%)	20-25 2 (11.76%)	F 17 (100%)	1 17 (100%)
		26-30 8 (47.06%)	P 0	2 0
		31-35 4 (23.53%)	N 0	3 0
		> 35 3 (17.65%)		
**2.6-3.0**	2 (2.78%)	20-25 0	F 2 (100%)	1 2 (100%)
		26-30 1 (50.0%)	P 0	2 0
		31-35 1 (50.0%)	N 0	3 0
		> 35 0		
**3.1-3.5**	1 (1.39%)	20-25 0	F 0	1 0
		26-30 1 (100%)	P 1 (100%)	2 1 (100%)
		31-35 0	N 0	3 0
		> 35 0		
**3.6-4.0**	1 (1.39%)	20-25 0	F 1 (100%)	1 1 (100%)
		26-30 0	P 0	2 0
		31-35 1 (100%)	N 0	3 0
		> 35 0		

**Graphic 1 (abstract A175).**
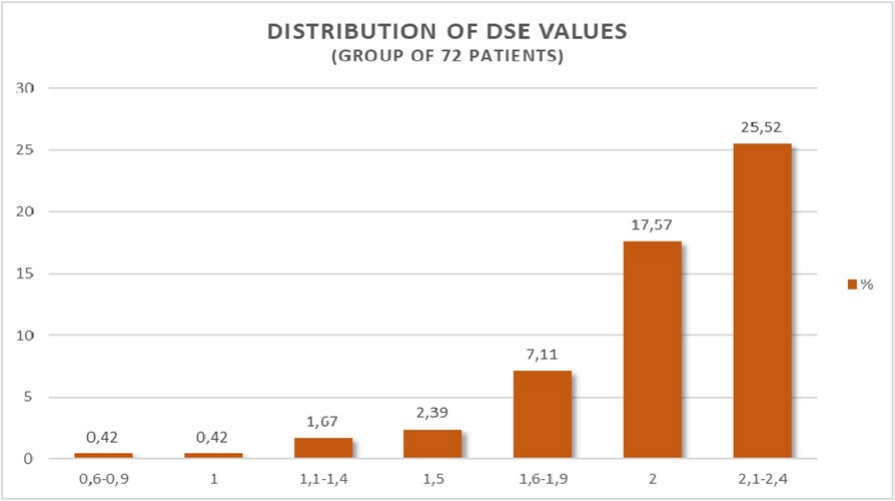
See text for description

**Table 2 (abstract A175).** Correlation between neck circumference and DSE

Neck circumference (cm)	N (%)	DSE (cm)
**20-30**	7 (9.72%)	0.6-1.0 0
		1.1-1.5 4 (57.14%)
		1.6-2.0 1 (14.29%)
		2.1-2.5 2 (28.57%)
		2.6-3.0 0
		3.1-3.5 0
		3.6-4.0 0
**26-30**	41 (56.95%)	0.6-1.0 1 (2.44%)
		1.1-1.5 3 (7.32%)
		1.6-2.0 27 (65.85%)
		2.1-2.5 8 (19.51%)
		2.6-3.0 1 (2.44%)
		3.1-3.5 1 (2.44%)
		3.6-4.0 0
**31-35**	16 (22.22%)	0.6-1.0 1 (6.25%)
		1.1-1.5 1 (6.25%)
		1.6-2.0 8 (50.0%)
		2.1-2.5 4 (25.0%)
		2.6-3.0 1 (6.25%)
		3.1-3.5 0
		3.6-4.0 1 (6.25%)
**> 35**	8 (11.11%)	0.6-1.0 1 (12.5%)
		1.1-1.5 2 (25.0%)
		1.6-2.0 2 (25.0%)
		2.1-2.5 3 (37.5%)
		2.6-3.0 0
		3.1-3.5 0
		3.6-4.0 0

### A176 Can routine use of videolaryngoscopy overcome the predictive role of airway ultrasound for difficult intubation?

#### J. Silvestri ^1^, M. Ciuffreda ^2^, E. Pisello ^1,2^, S. Sorrenti ^1^, L. Brugiaferri ^1^, C. Chinigioli ^1^, D. Galante ^3^, C. Piangatelli ^2^

##### ^1^ Università Politecnica delle Marche, Ancona, Italy; ^2^ U.O.C. Anestesia e Rianimazione, Terapia Intensiva e del Dolore, Ospedale E. Profili, Fabriano, Italy; ^3^ U.O.C. Anestesia e Rianimazione, Terapia Intensiva e del Dolore, Ospedale G . Tatarella, Cerignola, Italy

###### **Correspondence:** J. Silvestri

*Journal of Anesthesia, Analgesia and Critical Care 2023,* **3(Suppl 1):**A176

Background

Unpredictable difficult laryngoscopy is still a challenge for anaesthesiologists and common clinical screening tests have shown a limited predictive value. Ultrasound-based airway assessment has been recently proposed as a tool to predict difficult intubation. In particular, a distance from the epidermis to the epiglottis (DSE, Distance from Skin to Epiglottis) > or = 2.5 cm is considered a predictor of difficult laryngoscopy.

Objectives

The aim of our preliminary study is to evaluate if routine use of the videolaryngoscope could overcome the predictive factors of difficult intubation, particularly a DSE > or = 2.5 cm, and to explore if there is a correlation between DSE and neck circumference.

Materials and Methods

A group of 239 patients, aged 17 to 95 years, undergoing elective surgeries in E. Profili Hospital in Fabriano, were recruited. Pre-operative evaluation was performed before surgery: the DSE with a linear ultrasound transducer placed in the transverse plane at the level of thyrohyoid membrane and the neck circumference were measured for each patient. All patients were routinely intubated using a videolaryngoscope and visual findings have been graded according to the Fremantle Videolaryngoscope Scoring System.

Results (Table 1)

- The most frequently observed value of DSE is between 2.1 and 2.4 cm (25.52%) (Graphic 1).
- Regardless of DSE values, we found a complete view of vocal cords in 80.75%, a partial in 17.99% and none in 1.26% of cases.
- Relating DSE values to videolaryngoscopic view, despite a DSE value > or = 2.5 cm, a complete vision has been reported in 79.25% of cases, partial in 19.81% of cases and none in 0.94% of cases.
- Videolaryngoscopic view allowed tracheal intubation of all patients with DSE > or = 2.5 cm and in 96.23% as a first attempt. There was no impossible intubation in this group of patients.
- Among patients with DSE > or = 2.5 cm, 22.64% presented a neck circumference < 40 cm, while 77.36% > or = 40 cm. 42.86% of patients with DSE < 2.5 cm had a neck circumference < 40 cm and 57.14% > or = 40 cm.

Conclusions

The use of videolaryngoscope seems to allow a complete view of the vocal cords and intubation at the first attempt in most of the cases, regardless of DSE. A directly proportional relationship between DSE and neck circumference was only found from a DSE value greater than 2,5 cm. Further studies are required to assess whether routine use of videolaryngoscope can overcome the predictive role of difficult intubation of DSE.

**Table 1 (abstract A176).** See text for description.

DSE (cm)	N (%)	Neck circumference (cm)	View of vocal cords with videolaryngoscopy (F = full, P = partial, N = none)	Intubation difficulty (1 = at first attempt, 2 = at second attempt or additional devices required, 3 = failed)
**0.6-0.9**	1 (0.42%)	< 40 0	F 1 (100%)	1 1 (100%)
		≥ 40 1 (100%)	P 0	2 0
			N 0	3 0
**1.0**	1 (0.42%)	< 40 1 (100%)	F 1 (100%)	1 1 (100%)
		≥ 40 0	P 0	2 0
			N 0	3 0
**1.1-1.4**	4 (1.67%)	< 40 2 (50.0%)	F 4 (100%)	1 4 (100%)
		≥ 40 2 (50.0%)	P 0	2 0
			N 0	3 0
**1.5**	7 (2.93%)	< 40 4 (57.14%)	F 6 (85.71%)	1 7 (100%)
		≥ 40 3 (42.86%)	P 1 (14.29%)	2 0
			N 0	3 0
**1.6-1.9**	17 (7.11%)	< 40 13 (76.47%)	F 15 (88.24%)	1 17 (100%)
		≥ 40 4 (23.53%)	P 2 (11.76%)	2 0
			N 0	3 0
**2.0**	42 (17.57%)	< 40 22 (52.38%)	F 35 (83.33%)	1 40 (95.24%)
		≥ 40 20 (47.62%)	P 6 (14.29%)	2 2 (4.76%)
			N 1 (2.38%)	3 0
**2.1-2.4**	61 (25.52%)	< 40 15 (24.59%)	F 47 (77.05%)	1 58 (95.08%)
		≥ 40 46 (75.41%)	P 13 (21.31%)	2 3 (4.92%)
			N 1 (1.64%)	3 0
**2.5**	24 (10.04%)	< 40 3 (12.50%)	F 18 (75.0%)	1 24 (100%)
		≥ 40 21 (87.50%)	P 6 (25.0%)	2 0
			N 0	3 0
**2.6-2.9**	51 (21.34%)	< 40 11 (21.57%)	F 44 (86.27%)	1 51 (100%)
		≥ 40 40 (78.43%)	P 7 (13.73%)	2 0
			N 0	3 0
**3.0**	22 (9.21%)	< 40 9 (40.91%)	F 17 (77.27%)	1 20 (90.91%)
		≥ 40 13 (59.09%)	P 4 (18.18%)	2 2 (9.09%)
			N 1 (4.55%)	3 0
**3.1-3.4**	4 (1.67%)	< 40 1 (25.0%)	F 2 (50.0%)	1 4 (100%)
		≥ 40 3 (75.0%)	P 2 (50.0%)	2 0
			N 0	3 0
**3.5**	1 (0.42%)	< 40 0	F 1 (100%)	1 1 (100%)
		≥ 40 1 (100%)	P 0	2 0
			N 0	3 0
**3.6-3.9**	4 (1.67%)	< 40 0	F 2 (50.0%)	1 2 (50.0%)
		≥ 40 4 (100%)	P 2 (50.0%)	2 2 (50.0%)
			N 0	3 0

**Graphic 1 (abstract A176).**
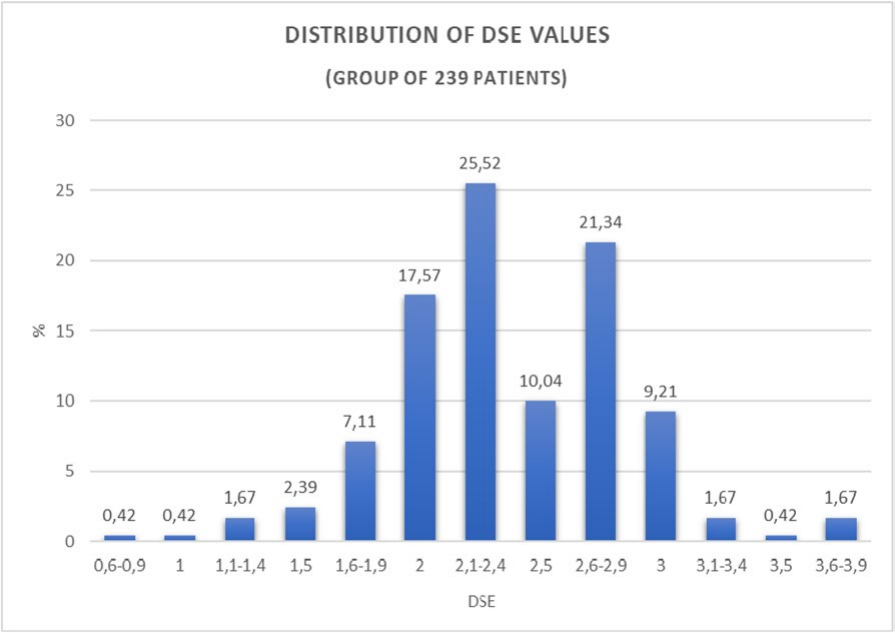
Distribution of DSE values

### A177 Post-intubation tracheobronchial laceration with an esophagus-tracheal fistula conservatively treated

#### F. Mombelli ^1^, P. Gnesin ^2^, C. Enrico ^2^

##### ^1^ Università degli Studi di Brescia, Brescia, ITALY, ^2^ ASST Franciacorta, Chiari, Brescia, Italy

###### **Correspondence:** F. Mombelli

*Journal of Anesthesia, Analgesia and Critical Care 2023,* **3(Suppl 1):**A177

Background

Postintubation tracheobronchial laceration (PITL) is a very infrequent (estimated incidence 0.005% for all endotracheal intubation) but potentially life-threatening disease, with significant morbidity and mortality rates. Conservative treatment is generally reserved for low grade lesions, up to IIIA grade according to Cardillo and colleagues (1), who proposed a risk-stratified morphological classification of PITL, attempting to standardize their management.

Case Report

A 61 years-old female with clinical history of severe obesity (BMI 48 kg/m2), diabetes, chronic kidney disease, personality disorder, chronic heart failure, chronic obstructive pulmonary disease (COPD) and active smoker was conducted to emergency department. The patient experienced a respiratory failure by COPD exacerbation with pneumonia.

Although a not invasive ventilation treatment was performed the patient’s conditions worsened. She was intubated with a not armed 8.0 mm internal diameter (ID) tube and moved to our intensive care unit (ICU).

During the next two days the patient improved respiratory exchanges during invasive ventilation.

A flexible bronchoscopy done for a few episodes of desaturation showed a posterior-wall tracheal lesion (1,5 cm proximal to the tracheal hull), probably iatrogenic.

CT scan of the neck showed a 14 x 7 mm tracheal laceration with esophagus-tracheal fistula (IIIB grade) (Figure 1: neck CT).

During the surgical tracheotomy surgeons placed a 7.0 mm ID tracheal cannula with the cuff proximal to the lesion to avoid hypoventilation of the left lung.

This tracheal lesion was considered suitable for surgical repair.

Ten days after the admission to the ICU, while waiting for the surgical procedure, the patient was re-evaluated with a bronchoscopy which evidenced a partially re-epithelialization of the tracheal lesion with granulation tissue. An esophagogastroduodenoscopy was performed with no evidence of connection between esophagus and trachea.

Follow-up CT scan confirmed that the fistula has been resolved spontaneously.

Patient's clinical conditions gradually improved so she was transferred to a rehabilitation unit two weeks after the hospitalization.

Conclusion

In this case report we describe a patient with a IIIB grade PITL conservatively treated where the anatomical condition could not exclude the tracheal laceration from the mechanical ventilation.

Informed consent to publish had been obtained.

REFERENCES

1. Cardillo G, Ricciardi S, Forcione AR, Carbone L, Carleo F, Di Martino M, Jaus MO, Perdichizzi S, Scarci M, Ricci A, Dello Iacono R, Lucantoni G, Galluccio G. Post-intubation tracheal lacerations: Risk-stratification and treatment protocol according to morphological classification. Front Surg. 2022 Nov 23;9:1049126. doi: 10.3389/fsurg.2022.1049126. PMID: 36504581; PMCID: PMC9727090.

**Fig. 1 (abstract A177).**
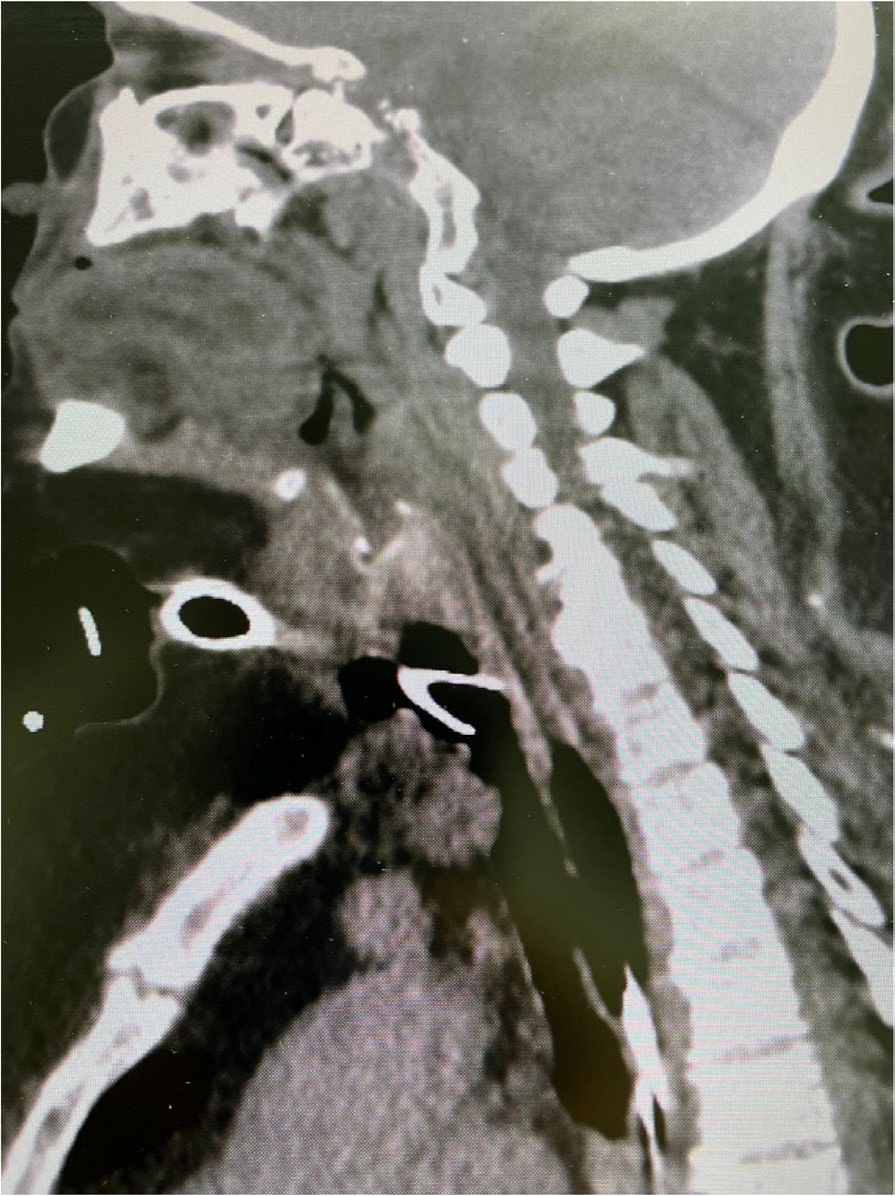
See text for description

### A178 Conservative treatment of tracheal lesion with self-expandable tracheal stent

#### D. Loizzi ^1^, D. Mongiello ^1^, L. Mirabella ^2^, K. Mariano ^2^, A. Cotoia ^2^, R. De Bellis ^1^, N.P. Ardò ^1^, M.T. Bevilacqua ^1^, G. Pacella ^1^, G. Cinnella ^2^, F. Sollitto ^1^

##### ^1^ S.C. di Chirurgia Toracica Policlinico Riuniti- Università degli Studi di Foggia, Foggia, Italy; ^2^ S.C. Anestesia e Rianimazione Policlinico Riuniti - Università degli Studi di Foggia, Foggia, Italy

###### **Correspondence:** D. Loizzi

*Journal of Anesthesia, Analgesia and Critical Care 2023,* **3(Suppl 1):**A178

BACKGROUND

Iatrogenic tracheal rupture (ITR) represents a rare but life-threatening condition requiring timely diagnosis and appropriate treatment. Surgical repair has always been the gold standard approach. Conservative treatment in selected cases is gaining an emerging role. We report a case of a iatrogenic tracheal tear in an adult woman intubated for elective abdominal surgery, conservatively and effectively treated with a self-expandable metal Y-shaped stent.

CASE DESCRIPTION

A 41-Year-old obese woman (BMI= 34.6) was referred to our department, for the suspicion of a tracheal tear, after cholecystectomy in general anesthesia. After extubation, the onset of dyspnea and subcutaneous emphysema led to reintubation and mechanical ventilation. A chest CT revealed bilateral pneumothorax, pneumomediastinum, and the tracheal dilatation lumen in correspondence with the cuff of the orotracheal tube. The endoscopic examination of the airways showed a longitudinal lesion of about 5 cm of the pars membranacea of the distal trachea. Surgical tracheostomy was performed, and two orotracheal tubes were placed through the tracheostomy in each of the main bronchi. On the seventh day, in general anesthesia and rigid bronchoscopy, we placed a Y-shaped tracheal stent. Then a tracheostomy tube was placed with the tip and the cuff inside the stent's lumen, avoiding decubitus of the tracheal wall. The next day the patient was placed in spontaneous breath, keeping the tracheostomy open, and was transferred to the hospital ward. A daily bronchial tree toilette was performed every day. Chest x-ray showed complete re-expansion of lungs. After 40 days, we removed the stent and observed a good resolution of the tracheal lesion.

CONCLUSIONS

The use of a tracheal stent provides a new strategy for the conservative treatment of tracheal tears. For tracheal lesions of the distal trachea, Y-stent placement can be considered a treatment option.

https://www.youtube.com/watch?v=Fx2kyyM2Cjg

I declare the collection of informed consent for the elaboration trachea before and after the treatment with y-stent

**Fig. 1 (abstract A178).**
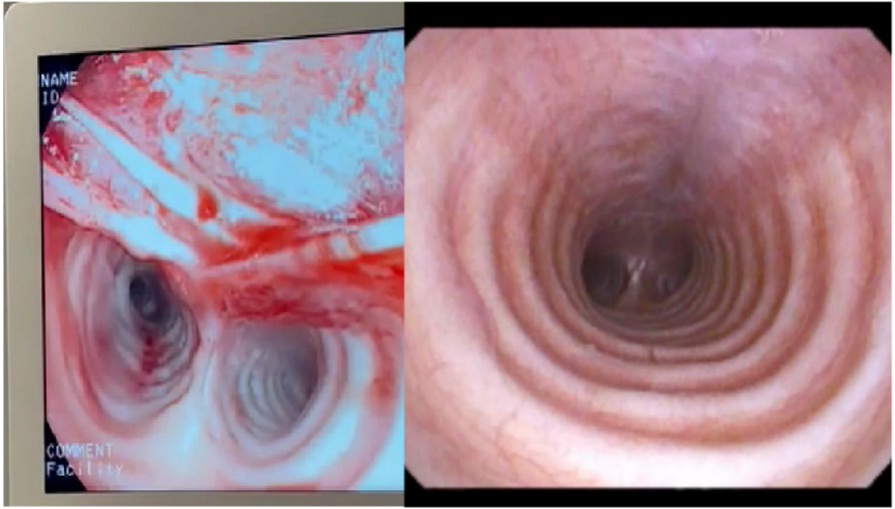
See text for description

### A179 Awake intubation with the McGrath series 5 videolaryngoscope in a newborn with a voluminous and occluding tongue tumor

#### Rosa Gallo ^1^, Francesco Maiarota ^1^, Caterina Grande ^2^*, Massimiliano Sorbello ^3^, Alessandra Pasqua ^2^, Pino Pasqua ^1^

##### ^1^ Azienda Ospedaliera di Cosenza, Cosenza, Italia; ^2^ Università degli Studi Magna Graecia di Catanzaro, Catanzaro, Italia; ^3^ Ospedale Giovanni Paolo II, Ragusa, Italia

###### **Correspondence:** C. Grande

*Journal of Anesthesia, Analgesia and Critical Care 2023,* **3(Suppl 1):**A179

Background

An unexpected partial airways’ obstruction of a newborn, caused by a voluminous neoformation of the tongue, not diagnosed in gynecological checkups, is an extremely critical situation. The neonatal videolaryngoscope, in this context, is an excellent device for managing the airways [1] but unfortunately not available in all birth centers.

The adult-pediatric videolaryngoscope, present in every modern operating room, is not validated in neonatal patients and no studies have been conducted in this population.

Case report

Term newborn weighing 3430 g with a voluminous congenital neoplasm of the tongue obstructing the oral cavity and with partial occlusion of the airways (Figures 1, 2).

With oxygen support, saturation was optimal and we had enough time to devise a strategy. The angiomatous and pedunculated nature of the lesion could rapidly evolve into dramatic scenarios of CICO (can't intubate, can't oxygenate), so orotracheal intubation indication was given for urgent surgery to remove the neoformation. At our hospital we do not have a neonatal pediatric videolaryngoscope with hyperangulated blades, then we decided to keep the little patient breathing spontaneously and managed the situation with the devices in our possession. Once a venous access was found and the little patient monitored, we performed an ultrasound of the neck to identify the cricothyroid membrane, in anticipation of a possible emergency cricothyroidotomy. Denitrogenation was performed with 100% oxygen, positioning the face mask with a two-handed technique. In accordance with the most recent guidelines [2], it was decided not to depress the breath and sedate (Ramsey 2) the patient with inspired Sevorane 4%. While an assistant immobilized the head, we proceeded, sliding along the hard palate, to extremely delicate indirect laryngoscopy with the mcgrath series 5 videolaryngoscope and with the blade positioned on the smallest size (small adult). So a Cormack Lehane grade II vision was obtained and it was possible to intubate with tube number 2.5 expanded and hyperangulated by performing the classic Cloche maneuver in about 3 seconds. The tube was then cuffed and connected to the breathing circuit with ETCO2 monitoring confirming proper placement. Only after confirmation of the ETCO2 was the sedation deepened and rapid myoresolution was acquired by administering rocuronium 1 mg/kg.

Conclusions

In literature there are no studies on the use of the mcgrath series 5 videolaryngoscope for expected difficult management of airways in neonatal population. Our experience confirms latest guidelines, namely that in extremely critical cases it is necessary to proceed with intubation guaranteeing spontaneous breathing and even in the absence of dedicated devices the right strategy can be implemented: target oriented now and no longer device oriented.

**No conflict of interest**

The authors declare, under their own responsibility and in full knowledge of the criminal liability envisaged for false declarations, non existence, even potential, of conflict of interest.

**Consent to publish**

The authors declare that they have been authorized by the parents of the minor and that they have acquired their consent to use the images and data produced so that they can be used in the case report.

Informed consent to publish had been obtained.

References

1. Wald, Keyes, Brown. Pediatric video laryngoscope rescue for a difficult neonatal intubation. Paediatr Anaesth. 2008; 18: 2-790.
2. Apfelbaum, Hagberg, Connis, Abdelmalak, Agarkar, Dutton, Fiadjoe, Greif, Klock, Mercier, Sullivan, Rosenblatt, Sorbello, Tung. 2022 American Society of Anesthesiologists Practice Guidelines for Management of the Difficult Airway. Anesthesiology. 2022; 136: 31-81.

**Fig. 1 (abstract A179).**
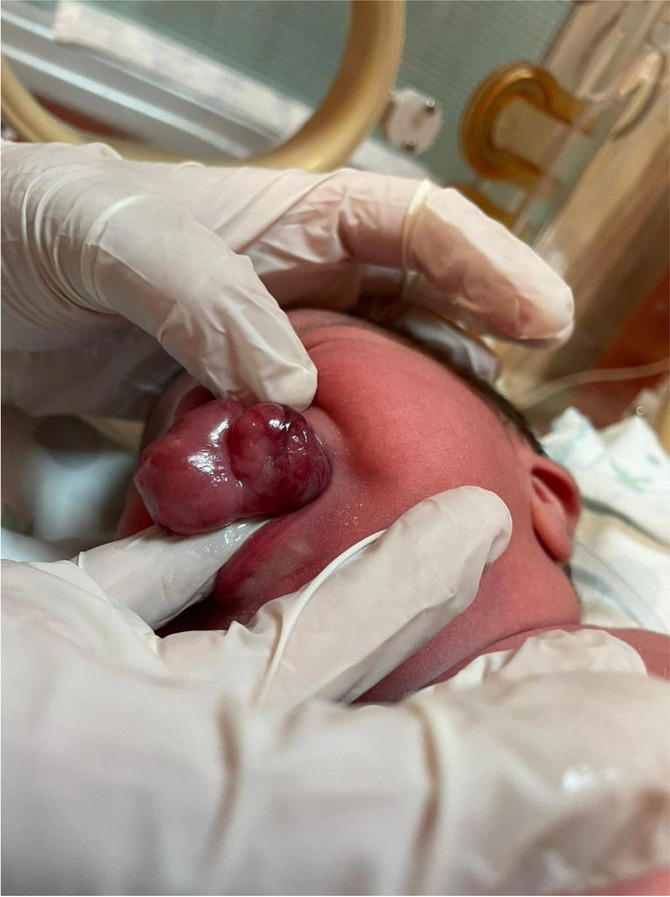
Congenital neoplasm of the tongue

**Fig. 2 (abstract A179).**
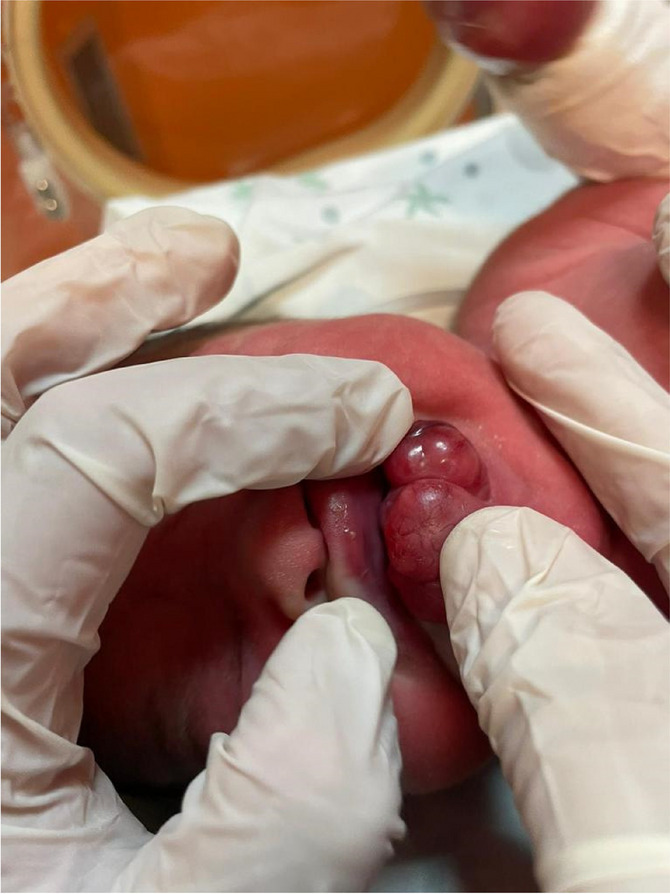
Congenital neoplasm occluding the oral cavity of the newborn

### A180 Airway management in mucopolysaccharidoses type-III: learning from pitfalls

#### C. Negro, S. Todaro, A. Ascari, A. Micalizzi, A.M. Cazzaniga, T. Aloisio, M. Ranucci, U. Di Dedda

##### IRCCS Policlinico San Donato, San Donato Milanese, MIlan, Italy

###### **Correspondence:** S. Todaro

*Journal of Anesthesia, Analgesia and Critical Care 2023,* **3(Suppl 1):**A180

Background

Mucopolysaccharidoses (MPS) are rare lysosomal storage disorders resulting from deficiencies in enzymes responsible for breaking down glycosaminoglycans [1,2]. MPS exhibit a wide range of clinical symptoms, including soft tissue and skeletal abnormalities, hepatosplenomegaly, central nervous system (CNS) disorders, and cognitive impairment. MPS type-III (MPS-III), known as Sanfilippo syndrome, arises from a deficiency in one of the four enzymes involved in heparan sulfate degradation, leading to progressive CNS degeneration. Symptoms include developmental delay, behavioral problems, musculoskeletal manifestations, and respiratory infections [3,4].MPS patients often undergo multiple surgeries requiring general anesthesia and airway management poses challenges due to macroglossia and altered facial physiognomy, particularly in adult patients, for whom limited literature exists [5,6].

Case Report

We present a case of a 36-year-old male MPS-III patient (weight 36 kg, height 130 cm) suffering from severe aortic valve insufficiency with indication for cardiac surgery. The case suggested a potential challenge in airway management due to certain physical characteristics associated with MPS-III, including macroglossia, short neck distances, pathognomonic physiognomy (Figure 1), restrictive chest disease, and profuse drooling. Adult MPS-III patients are rare and no specific guidelines on sedation nor airway management were found. To address expected difficulties, the operating room was equipped with tools for managing difficult airways, such as supraglottic devices, Frova intubating introducer, and an emergency cricothyroidotomy kit. Considering the likelihood of difficult mask ventilation, our initial approach involved awake fiberoptic intubation. Dexmedetomidine 1.2mcg/kg/min, midazolam 0,15 mg/kg and lidocaine 2% spray were used for sedation and local anesthesia purposes. However, due to the patient's cognitive impairment, lack of cooperation, low sedation levels to avoid respiratory depression, copious secretions, and macroglossia, this primary approach failed. To prevent desaturation and patient discomfort, we transitioned to deepening anesthesia using propofol 1% 1 mg/kg. One attempt was needed to secure the airway using videolaryngoscopy with a D-blade and a 6.5 mm cuffed endotracheal tube. Extubation occurred on the first postoperative day in the intensive care unit following a cuff-leak test to rule out upper airway edema. No desaturation, respiratory distress, or incoercible cough occurred within the 24-hour post-extubation observation period.

Conclusion

Limited literature exists on anesthetic management in MPS patients, particularly in adults. Based on our experience, we recommend videolaryngoscopy as the initial approach for non-cooperative adult MPS-III patients. Difficult titration of sedation, intellectual disability, excessive secretions, and challenges in local anesthesia application due to macroglossia hinder successful awake fiberoptic intubation.

INFORMED CONSENT

Written informed consent to participate in the case report has been obtained from patient’s legal guardian (mother). Absolute anonymity of the patient was guaranteed.

References

1. Tomatsu S, et al. Newborn screening and diagnosis of mucopolysaccharidoses. Mol Genet Metab.2013; 110(1-2):42-53.
2. Cimaz R, La Torre F. Mucopolysaccharidoses. Curr Rheumatol Rep.2014;16(1):389.
3. Heon-Roberts R, et al. Molecular Bases of Neurodegeneration and Cognitive Decline, the Major Burden of Sanfilippo Disease. J Clin Med.2020;27;9(2):344.
4. Jakobkiewicz-Banecka J, et al. Glycosaminoglycans and mucopolysaccharidosis type III. Front Biosci (Landmark Ed).2016;21(7):1393-409.
5. Clark BM, et al. Anesthesia for patients with mucopolysaccharidoses: Comprehensive review of the literature with emphasis on airway management. Bosn J Basic Med Sci.2018;18(1):1-7.
6. Moretto A, et al. Anesthesiological risks in mucopolysaccharidoses. Ital J Pediatr. 2018;44 (Suppl 2):116.

**Fig. 1 (abstract A180).**
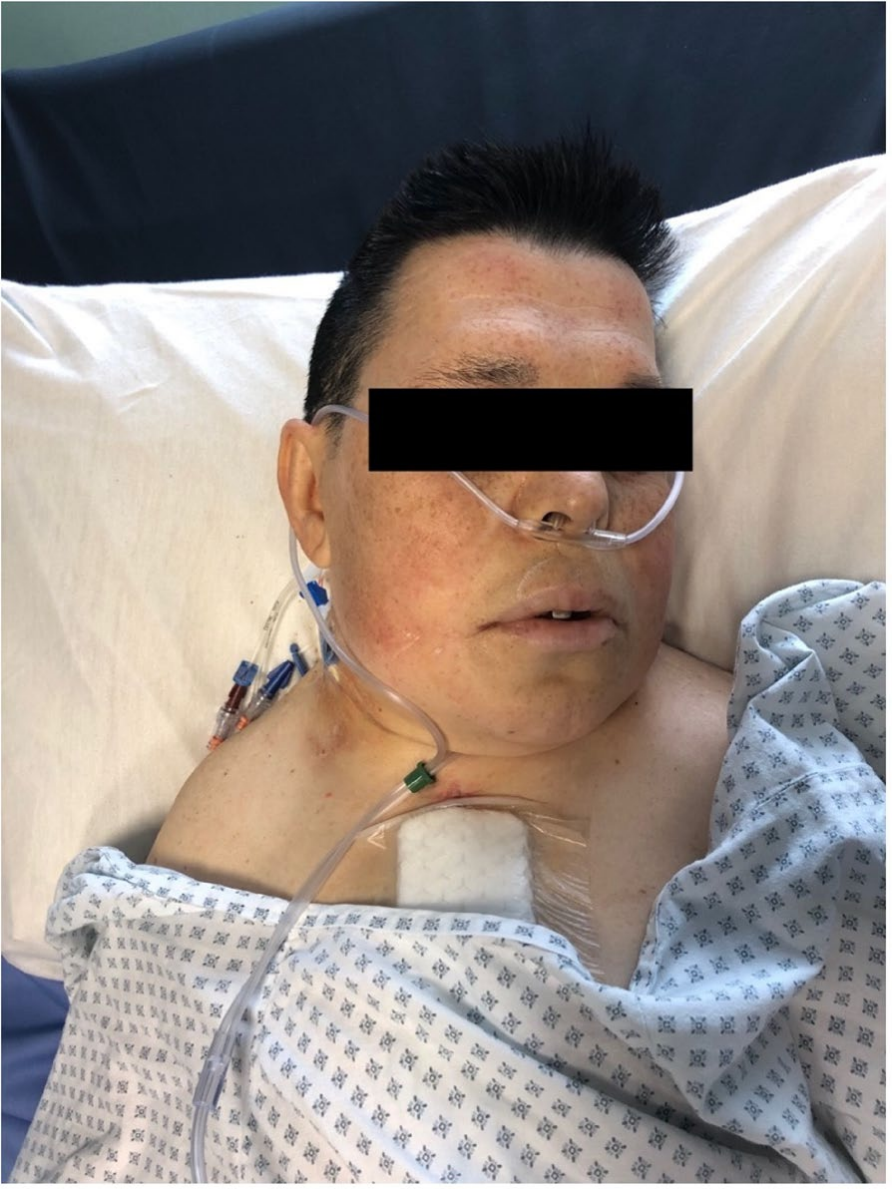
A 36-year-old man with MPS-III; facial, neck and chest characteristics-frontal view

